# Diagnostic accuracy of point-of-care ultrasound in adult non-traumatic emergency medicine: an umbrella review

**DOI:** 10.1186/s12916-026-05094-2

**Published:** 2026-07-25

**Authors:** Claudio Schneider, Nico Frei, Antoine Fernandez, Catherine Frei, Katharina Wahedi, Julius Husarek, Tanya Karrer, Carole Elodie Aubert, Beat Lehmann, Martin Müller

**Affiliations:** 1 Department of General Internal Medicine, Inselspital, Bern University Hospital, University of Bern, Bern, Switzerland; 2 Department of Emergency Medicine, Bern University Hospital, Inselspital, University of Bern, Bern, Switzerland; 3 Department of Emergency Medicine, Lindenhofspital, Bern, Switzerland; 4 Department of Internal Medicine, Spital Emmental, Burgdorf, Switzerland; 5 Medical Library, University Library of Bern, University of Bern, Bern, Switzerland; 6 Institute for Primary Healthcare (BIHAM), University of Bern, Bern, Switzerland

**Keywords:** Point-of-care ultrasound, POCUS, Emergency medicine, Diagnostic accuracy, Umbrella review, Systematic review, Meta-analysis, Non-traumatic emergency presentations, Adult patients, GRADE

## Abstract

**Background:**

Point-of-care ultrasound (POCUS) has become an increasingly used diagnostic tool in adult emergency departments (ED). Although numerous diagnostic test accuracy systematic reviews (DTA-SR) evaluating various POCUS applications are available, clinicians often face uncertainty due to ungraded accuracy estimates and inconsistent evidence. We conducted an umbrella review to (i) summarize diagnostic accuracy evidence for POCUS across adult non-traumatic emergency medicine-relevant conditions, (ii) provide GRADE-based ratings of pooled sensitivity and specificity, and (iii) synthesize findings when multiple DTA-SR addressed the same condition.

**Methods:**

We systematically searched PubMed and Embase from database inception to April 2026 for DTA-SR with meta-analysis evaluating the diagnostic performance of POCUS in adult ED populations for non-traumatic emergency medicine-relevant conditions. DTA-SR were included if they reported pooled sensitivity and specificity against accepted reference standards. The methodological quality was assessed using a DTA-adapted AMSTAR 2 tool. Sensitivity and specificity estimates were graded using the GRADE approach. Results were synthesized using a predefined hierarchical decision framework to account for overlap between primary studies.

**Results:**

A total of 197 DTA-SR were included, most focusing on cardiovascular, gastrointestinal, respiratory, or musculoskeletal conditions. About half were conducted in ED; the rest examined emergency medicine-relevant conditions in specialist settings. Methodological quality was mostly low or moderate. Diagnostic utility varied substantially across conditions. Among 91 evaluated conditions, 12 fulfilled predefined criteria for combined rule-in/rule-out supportive applications, 17 for primarily rule-in supportive applications, and 6 for primarily rule-out supportive applications (defined as pooled sensitivity or specificity ≥ 90% with at least moderate certainty of evidence). The remaining conditions were categorized as adjunctive because these thresholds were not met. Overall, certainty of evidence was frequently low or very low.

**Conclusions:**

POCUS shows promising diagnostic accuracy across adult non-traumatic emergency medicine-relevant conditions, often with moderate to high sensitivity and very high specificity. However, the overall strength and consistency of the evidence are limited. Therefore, diagnostic performance estimates should be interpreted with caution and within the context of each specific clinical condition, and should not be considered directly comparable or additive across different indications. Further high-quality research is needed to better define its role in emergency care.

**Clinical Trial Number:**

Clinical trial number: CRD42024587695.

**Supplementary Information:**

The online version contains supplementary material available at https://doi.org/10.1186/s12916-026-05094-2.

## Background

Emergency departments (ED) face significant challenges due to overcrowding, which can lead to prolonged waiting times, increased patient discomfort, and potentially adverse outcomes due to diagnostic errors [1, 2]. Diagnostic errors occur frequently, leading to longer hospital stays and higher in-hospital mortality rates for affected patients [3], making them a leading cause of death in hospitals [4]. In this high-pressure environment of the ED, clinicians must rely on diagnostic tools that not only expedite patient care but also maintain a low rate of diagnostic errors to ensure patient safety and effective treatment [5, 6].

Point-of-care ultrasound (POCUS), defined as focused ultrasound brought to the patient and performed by the provider in real time, emerges as a powerful diagnostic tool that fulfils these critical needs in the ED [7–13]. However, clinical assessment based on history taking and physical examination remains the cornerstone of initial evaluation. Nevertheless, both have well-recognized limitations, particularly in patients presenting with syndromal conditions such as dyspnea, shock, acute kidney injury, or suspected free fluid [14–17]. In these scenarios, clinical findings may be non-specific, incomplete, or misleading, contributing to diagnostic uncertainty and delays. In this context, POCUS offers real-time imaging possibilities that can rapidly assist in the diagnosis and management of various conditions, reducing the reliance on more time-consuming and resource-intensive imaging modalities [18]. Importantly, it should be understood as an adjunct that complements and augments, rather than replaces, the classical clinical assessment [19, 20]. Its advantages include portability, immediacy of results, and the ability to perform bedside evaluations, which can significantly improve patient flow and outcomes in the ED [18, 21].

A substantial body of systematic reviews (SR) and meta-analyses (MA) exists on the use of POCUS across a variety of diseases and conditions. However, a critical issue is that estimates of diagnostic accuracy, particularly regarding sensitivity and specificity of POCUS, often lack a clear evidence rating, leaving clinicians uncertain about its reliability for safely ruling in or ruling out specific conditions [9, 21–24]. Additionally, for several conditions multiple MA have been conducted, yet to our knowledge no comprehensive synthesis has been conducted to consolidate these findings [25].

In response to these gaps, we conducted an umbrella review with the objectives to: [26] (i) summarize the existing evidence for various specific non-traumatic POCUS conditions, (ii) provide a grading of the reported pooled sensitivity and specificity of POCUS for these conditions in comparison to the respective reference standard, enabling clinicians to confidently use POCUS among adults or mixed population in emergency settings, and (iii) offer a qualitative synthesis where multiple diagnostic test accuracy systematic reviews (DTA-SR) exist for the same condition, ensuring that the best available evidence is accessible and clearly presented in order to guide clinical practice.

## Methods

This umbrella review or overview of reviews is structured according to the Cochrane recommendations [27] and is reported in accordance with the Preferred Reporting Items for Overviews of Reviews (PRIOR) statement [28]. The protocol was registered on PROSPERO prior to the commencement of full-text screening (CRD42024587695) [29]. During the review process, the scope was restricted to non-trauma conditions after initial title and abstract screening due to the large number of eligible reviews. This change was not formally registered as a PROSPERO amendment.

### Study selection

We included all DTA-SR with MA addressing research questions on diagnostic accuracy of ultrasound with potential applicability as POCUS in the ED.

Reviews had to meet the criteria established by Krnic Martinic et al. (2019) [30] to be classified as SR (see Additional file 1: Table S1) [30]. Eligible were DTA-SR on adult or mixed populations (≥ 16 years) with non-traumatic emergency medicine-relevant conditions or acute interventions assessed using ultrasound. Conditions were considered eligible if they were deemed clinically relevant to acute or emergency care, even if the underlying diagnostic accuracy evidence originated from non-ED or specialist settings. Conventional ultrasound (B-mode, Color Doppler) and contrast-enhanced ultrasound (CEUS) applicable in acute care were included. Advanced Doppler applications with direct relevance to emergency medicine and critical care practice, such as spectral Doppler techniques for assessment of stroke volume and fluid responsiveness (e.g., velocity time integral [VTI]) or venous congestion assessment (e.g., the VExUS protocol), were considered within the scope of emergency medicine-relevant POCUS investigations. In contrast, highly specialized cardiologic examinations, including detailed assessment of complex valvular pathology (e.g., tricuspid regurgitation), were considered part of extended ultrasound and were therefore not included. Reviews on specialized methods (e.g., transrectal, endoscopic, elastography) were likewise excluded. Reviews limited to chronic conditions without acute exacerbations, cancer diagnostics, or pediatric-only populations were excluded as well as reviews only including trauma patients. Studies evaluating Artificial Intelligence (AI)-based diagnostic models were excluded, as their reported accuracy reflects model- and dataset-specific performance rather than the intrinsic diagnostic accuracy of a standardized clinical test. This decision was made to ensure conceptual and methodological consistency within the diagnostic test accuracy framework. Only studies comparing ultrasound with accepted reference standards were eligible. If multiple standards were pooled, all were accepted; if stratified, the most accepted reference standard was chosen for reporting and GRADE assessment.

### Search strategy

We conducted a systematic literature search including PubMed and Embase up to April 2026. To enhance search completeness, a limited screening of the most relevant records from Google Scholar and Elicit was performed. In addition, reference lists of included reviews were screened to identify further relevant studies. Studies published in English or German were eligible for inclusion. No dedicated search for grey literature was conducted, as systematic reviews with meta-analyses are typically published in peer-reviewed journals and indexed in major databases, and were therefore expected to be adequately captured by our search strategy. The detailed search algorithm, developed and validated in collaboration with an information specialist, is provided in Additional file 1: Table S2 [27, 31–36].

### Data collection and analysis

#### Selection of reviews

Titles and abstracts were screened in two steps. First, two reviewers performed a sensitive screening of all DTA-SR with MA (Inter-rater agreement, κ = 0.60). Following initial bibliographic screening, two senior emergency physicians with expertise in POCUS independently assessed the conditions and ultrasound applications investigated in the selected reviews for eligibility based on their relevance to emergency medicine POCUS practice. Reviews focusing on conditions or ultrasound applications considered outside the intended scope of emergency medicine POCUS were excluded. Inter-rater agreement was calculated (κ = 0.88), and disagreements were resolved by consensus or consultation with a third reviewer. Applicability was defined as relevance to acute care decision-making in emergency settings, irrespective of whether the underlying studies were conducted exclusively in the ED or in mixed or specialist settings. To ensure a broad and clinically meaningful evidence base, reviews were not excluded solely because a substantial proportion, even the majority, of included primary studies originated from non-ED settings, provided the diagnostic question and POCUS application were considered transferable to emergency care practice. However, the degree of direct ED applicability and setting-specific limitations were subsequently considered during evidence grading and interpretation. Reviews deemed applicable were selected for full-text assessment. Full texts were independently assessed for inclusion by two reviewers, with a third consulted in case of disagreement (κ = 0.77). The number of records excluded at each stage is reported in the PRISMA flow diagram.

#### Data extraction and management

All records were initially managed by a medical information specialist using EndNote for search execution and update handling. Subsequently, records were imported into Zotero for reference management. Duplicate removal was performed using Deduklick. Screening was conducted using Covidence. Data extraction as well as documentation of AMSTAR and GRADE assessments were performed using Microsoft Excel.

Data were independently extracted by two reviewers, with discrepancies resolved by a third. Extracted information included: general study characteristics (author, year, setting, population); condition under study with classification into domain, subgroup, and detailed description; index test and reference standard; and risk of bias of the DTA-SR and included studies. For each condition, pooled sensitivity and specificity with 95% confidence interval (CI) or standard error (SE), number of studies and participants, prevalence, and GRADE domains for sensitivity/specificity were recorded. Subgroup analyses and multiple index tests were extracted when reported.

#### Data synthesis

Included DTA-SR were grouped by condition into 10 domains: cardiovascular, ear/nose/throat, gastrointestinal, genitourinary, gynecological, intervention-related, musculoskeletal, neurological, ocular and respiratory. Each review could appear in several domains if multiple conditions were studied. Within each domain, subgroups and specific conditions were defined.

Each eligible DTA-SR was summarized using a standardized framework (clinical context, index test, reference standard, methodological quality, diagnostic accuracy, certainty of evidence and authors’ conclusions). These summaries were presented as fact sheets. Multiple fact sheets exist for one specific condition if there were multiple (overlapping/not overlapping) DTA with MA on that condition. Primary study overlap across included reviews was formally quantified for conditions including five or more SR using the corrected covered area (CCA) according to the method proposed by Pieper et al. [37] This approach was chosen to focus on conditions with a larger evidence base, where overlap between reviews is most likely to influence the interpretation of results. Because of substantial overlap among primary studies across eligible SRs and considerable methodological heterogeneity, particularly regarding patient populations, index tests, and reference standards, no additional quantitative synthesis was undertaken to avoid double-counting and to preserve the interpretability of pooled estimates. For each fact sheet, one representative pair of pooled sensitivity and specificity was selected, graded, and reported. All additional extracted pooled sensitivity/specificity pairs remained available within the corresponding fact sheet. Selection of the representative pair followed a predefined hierarchy: (i) reviews most focused on ED/POCUS, (ii) multi-organ POCUS prioritized over single-organ ultrasound for complex conditions, (iii) largest number of studies. If no unique pair was available, the highest sensitivity and/or specificity with the narrowest CI were selected.

In a second step, one representative SR MA per condition was selected and identified for presentation in the main summary tables (Tables 2a–c and 3a–b). This selection followed the hierarchy: (i) AMSTAR rating, (ii) GRADE certainty, and (iii) ED focus.

To facilitate clinically oriented interpretation, all conditions were additionally categorized according to their potential diagnostic utility as rule-in supportive, rule-out supportive, or combined rule-in/rule-out supportive applications. Potential rule-out utility was defined as pooled sensitivity ≥ 90% with at least moderate certainty of evidence, whereas potential rule-in utility was defined as pooled specificity ≥ 90% with at least moderate certainty. Conditions not meeting these thresholds were categorized as adjunctive applications.

The following definitions were used to describe/group sensitivity and specificity: very high > 90%, high 80%-90%, moderate 70%-80%, low 50%-70%, very low < 50%.

#### Quality assessment

To assess methodological quality, we used an adapted AMSTAR 2 checklist [38] (see Additional file 1: Table S3 [39]). Reviews were independently rated by two reviewers as “*critically low*”, “*low*”, “*moderate*”, or “*high*”; discrepancies were resolved by discussion or a third reviewer if necessary. In addition to the overall methodological appraisal, AMSTAR 2 items 12 and 13 (the latter being critical) were specifically used as a pragmatic proxy for risk of bias of the included meta-analyses. As AMSTAR 2 is not a dedicated risk-of-bias tool, this approach represents a pragmatic adaptation.

The certainty of evidence for each condition and pooled sensitivity and specificity was independently assessed by two reviewers using the GRADE approach for diagnostic test accuracy. In accordance with GRADE guidance, evidence derived from well-designed cross-sectional or cohort diagnostic accuracy studies, including patients with diagnostic uncertainty and an appropriate reference standard, was considered to start at high certainty. Certainty was downgraded, as appropriate, for risk of bias, inconsistency, indirectness, and imprecision [40]. Publication bias was not formally assessed. In DTA research, conventional approaches for publication bias assessment (e.g., funnel plot asymmetry tests) are not well validated, as sensitivity and specificity are correlated and threshold-dependent measures that violate key assumptions underlying these methods [40]. In line with current Cochrane and GRADE guidance, publication bias was therefore considered indirectly within the overall risk of bias assessment rather than through formal statistical testing. Detailed downgrading criteria are provided in Additional file 1: Table S4. The certainty of evidence for each estimate was rated as high, moderate, low, or very low.

## Results

### Description of included reviews

**Fig. 1:**
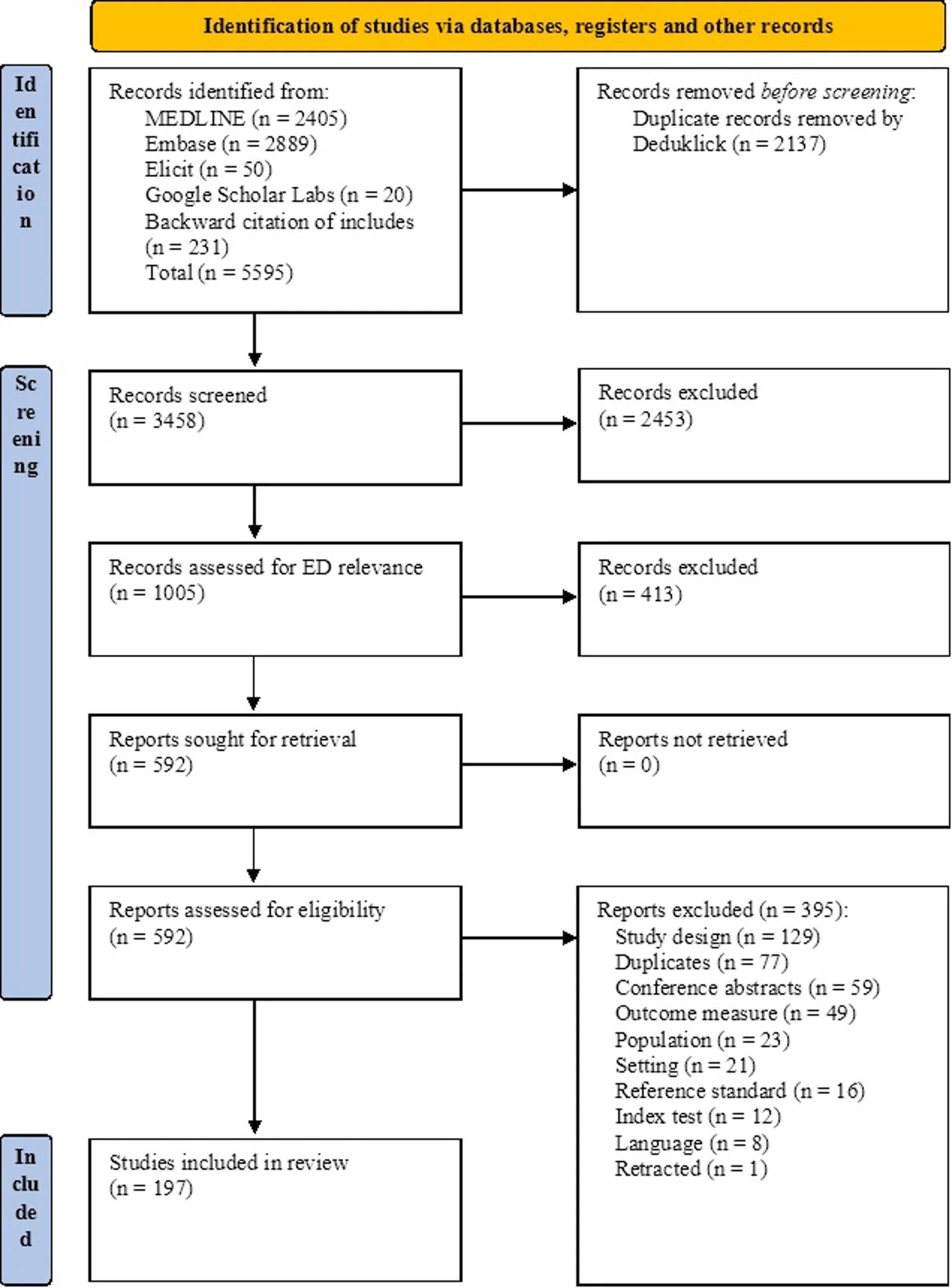
PRISMA flowchart. Source: Page MJ, et al. BMJ 2021;372:n71. doi: https://doi.org/10.1136/bmj.n71. This work is licensed under CC BY 4.0. To view a copy of this license, visit: https://creativecommons.org/licenses/by/4.0/

Figure 1 shows the results of the systematic search and selection of eligible studies. After the initial bibliographic screening, records underwent assessment of the investigated condition and ultrasound application for eligibility within the predefined scope of emergency medicine POCUS. Additional file 1: Table S5 provides an overview of assessed conditions and indicates whether they were considered eligible for inclusion. All studies excluded during this POCUS applicability assessment are listed in Additional file 2: Table S6. Subsequently, full-text records were assessed for eligibility; reasons for exclusion at full-text stage are provided in Additional file 3: Table S7. The search identified 197 non-traumatic DTA-SR. Most reviews were assigned to a single clinical category, while four reviews were classified into more than one category. Overall, reviews were distributed across 10 clinical categories (Table 1). As a result of overlapping classification, the sum of category counts exceeds the total number of included reviews: cardiovascular (58), ear/nose/throat (1), gastrointestinal (33), genitourinary (8), gynecological (11), intervention-related (17), musculoskeletal (21), neurological (18), ocular (3), or respiratory (31). Primary study overlap across conditions including five or more SRs was assessed using the CCA and varied substantially between conditions. Most conditions showed slight to moderate overlap, indicating that the included SRs were largely based on different primary studies despite addressing similar clinical questions. Overlap was low for appendicitis (3.0%), deep vein thrombosis (3.7%), raised intracranial pressure (4.5%), carotid artery stenosis (5.2%), acute cholecystitis (5.6%), acute heart failure (6.5%), and pulmonary embolism (7.6%). Moderate overlap was observed for small bowel obstruction (8.6%) and pneumonia (10.0%). Higher overlap was identified in a smaller number of well-studied conditions, including pneumothorax (12.1%) and endotracheal tube placement (12.6%), whereas very high overlap was observed for gout (20.0%) and cardiac arrest (27.2%), indicating that multiple SRs relied on more similar sets of underlying primary studies.

**Table 1:** Domain, subgroups and detailed diseases/conditions of included POCUS DTA-SR

Main group/subgroup	Detailed conditions (number of DTA pairs)
**Cardiovascular condition**	77 DTA pairs in 58 reviews
Aortic disease	Abdominal aortic aneurysm (3), ruptured abdominal aortic aneurysm (1), aortic dissection (1), aortic dissection type A (2), aortic dissection type B (1), acute thoracic aortic syndrome (1)
CPR	Cardiac arrest (7), PEA (3), post-cardiac arrest neurology (1)
Endocarditis	Infective (1), native (1), prosthetic (1)
Heart failure	Acute heart failure (10), LV diastolic function (1), LV systolic function (1), pulmonary edema (4)
Myocardial ischemia	Myocardial ischemia (1), myocardial infarction (1), acute coronary syndrome (1), significant coronary stenosis (1)
Shock related	Cardiogenic (4), distributive (3), hypovolemic (3), obstructive (4), mixed (2)
Venous thromboembolism	DVT (7), pulmonary embolism (11)
**Ear/Nose/Throat condition**	1 DTA pair in 1 review
Peritonsillar abscess	Peritonsillar abscess (1)
**Gastrointestinal condition**	37 DTA pairs in 33 reviews
Appendicitis	Appendicitis (15)
Biliary system	Acute cholecystitis (7), choledocholithiasis (1), cholelithiasis (2), gallbladder disease (1)
Intestinal obstruction	Small bowel obstruction (5)
Gastrointestinal other	Abdominal tuberculosis (1), acute pancreatitis (1), Budd-Chiari Syndrome (1), diverticulitis (2), gastric content (1)
**Genitourinary condition**	8 DTA pairs in 8 reviews
Testicular torsion	Testicular torsion (3)
Urolithiasis	Urolithiasis (2)
Genitourinary other	Acute kidney injury (1), bladder outlet obstruction (1), hydronephrosis (1)
**Gynecological condition**	11 DTA pairs in 11 reviews
Pregnancy	Cesarian scar pregnancy (1), ectopic pregnancy (3), intrauterine pregnancy (1), retained products of conception (2)
Adnexal torsion	Adnexal torsion (3), ovarian torsion (1)
**Intervention-related condition**	17 DTA pairs in 17 reviews
Airway related	Endotracheal tube placement (7), predicting difficult intubation (4)
Central venous catheter placement	Central venous catheter malposition (4)
Gastric tube placement	Nasogastric tube placement (2)
**Musculoskeletal condition**	21 DTA pairs in 21 reviews
Crystal arthropathies	CPPD (2), gout (6)
Vasculitis	Giant cell arteritis (4), Large vessel vasculitis (2)
Musculoskeletal	Abscess (2), knee effusion (1), necrotizing skin infection (1), retained foreign bodies in skin and soft tissue (1), rotator cuff tear (1), synovial abnormality (1)
**Neurological condition**	23 DTA pairs in 18 reviews
Intracranial artery stenosis	Carotid artery stenosis (5), intracranial artery stenosis (3), large vessel occlusion (1), stroke (Ischemic and hemorrhagic) (1), vertebral artery stenosis (2)
Raised intracranial pressure	Raised intracranial pressure (7)
Neurology other	Cauda equina syndrome (1), intracranial hemorrhage (1), intracranial masses (1), neurodegenerative disease (1)
**Ocular condition**	8 DTA pairs in 3 reviews
Retinal detachment	Retinal detachment (2)
Other ocular condition	Foreign body (1), globe rupture (1), lens dislocation (1), posterior disorder (1), vitreous detachment (1), vitreous hemorrhage (1)
**Respiratory condition**	35 DTA pairs in 31 reviews
Disorder of the pleural space	Hemothorax (1), Iatrogenic pneumothorax (1), pneumothorax (5), pleural effusion (2)
Infectious lung disease	COVID-19 (4), exacerbated asthma/COPD (2), pneumonia (14)
Respiratory other	Acute dyspnea (1), acute respiratory failure (2), acute respiratory distress syndrome (3)

Most reviews were assigned to a single clinical category, while four reviews were classified into more than one category

**Abbreviations**: COPD = chronic obstructive pulmonary disease; COVID-19 = coronavirus disease 2019; CPPD = calcium pyrophosphate deposition disease; CPR = cardiopulmonary resuscitation; DTA-SR = diagnostic test accuracy systematic review; POCUS = point-of-care ultrasound

In total, DTA-SR pairs were identified for 91 different conditions leading to 238 DTA pairs. The majority of DTA-SR pairs have been investigated in a pure adult population (149/238, 62.6%), while 49/238 (20.6%) included both adults and children and 37/238 (15.6%) did not report the age of their population. Three DTA-SR (1.2%) focused exclusively on pregnant women (all addressing appendicitis). In the remaining investigated condition, the study population was not specified. A total of 58.4% (139/238) of the DTA pairs were conducted entirely or partially in an ED environment. Overall, 81.2% (160/197) of the included DTA-SR were published within the last 10 years, reflecting the rapid growth and increasing adoption of POCUS in the past decade.

**Table 2:** (a) POCUS applications with rule-in and rule-out supportive diagnostic performance. (b) POCUS applications with rule-in supportive diagnostic performance. (c) POCUS applications with rule-out supportive diagnostic performance

a							
Condition	Sensitivity		Specificity		Number of reviews	Comment	Representative Study
	Range [%]	Certainty	Range [%]	Certainty			
**Cardiovascular**							
Acute heart failure	73.0–94.1	Moderate	81.8–96.0	Moderate	10	Mostly lung ultrasound. All studies with moderate study quality	Martindale [66] (2016)
Abdominal aortic aneurysm	97.5–99.0	High	98.9–100	High	3	All sensitivities and specificities above 95%	Concannon [41] (2014)
Deep vein thrombosis	89.7–96.1	High	93.8–98.7	High	7	Multiple reviews; range reflects between-review variability. Heterogenous certainty of evidence	Pomero [87] (2013)
Pulmonary embolism	53.0–91.8	High	63.9–99.0	High	11	Multiple reviews; range reflects between-review variability; Wide range across reviews	Melo [95] (2025)
Ruptured abdominal aortic aneurysm	97.8	Moderate	97.0	Moderate	1		Fernando [44] (2022)
Shock Hypovolemic	81.0–91.0	Moderate	91.0–95.0	Moderate	3	Two studies with moderate study quality, one with low	Yoshida [79] (2023)
**Genitourinary**							
Testicular torsion	86.4–95.3	Moderate	95.3–99.0	High	3	Moderate or high certainty of evidence for color Doppler, low for whirlpool sign.	Ota [135] (2019)
**Gynecological**							
Ectopic pregnancy	45.0–97.0	Moderate	76.0–99.0	Moderate	3	Wide range across reviews	Richardson [142] (2016)
**Neurological**							
Carotid artery stenosis	85.0–99.0	Moderate	70.0–98.0	Moderate	4	Doppler ultrasound. Wide range across reviews	Cassola [188] (2022)
Raised Intracranial pressure	82.0–94.0	Moderate	82.0–90.7	Moderate	7	Measurement of optic nerve sheath diameter. Multiple reviews; range reflects between-review variability	Koziarz [200] (2019)
**Respiratory**							
Pleural Effusion	93.0–94.5	Moderate	96.0–97.9	Moderate	2	All sensitivities and specificities above 95%	Zaki [215] (2024)
Pneumonia	37.0–97.0	Moderate	81.0–98.0	High	14	Multiple reviews; range reflects between-review variability; Wide range across reviews	Asmara [52] (2022)
**b**							
**Condition**	**Sensitivity**		**Specificity**		**Number of reviews**	**Comment**	**Representative Study**
	**Range [%]**	Certainty*	**Range [%]**	Certainty*			
**Cardiovascular**							
Acute thoracic aortic syndrome	89.0	Moderate	92.0	High	1		Mani [48] (2025)
Aortic dissection, Type A	58.0–86.7	Low	86.5–94.0	Moderate	2	Wide range across reviews	Yeh [47] (2025)
Pulmonary edema	88.0–97.0	Low	87.0–98.0	High	4	Mostly lung ultrasound	Asmara [73] (2022)
Shock Cardiogenic	75.0–95.0	Very low	94.0–98.0	High	4	Mostly RUSH. High variability in certainty of evidence of pooled sensitivities.	Yoshida [79] (2023)
Shock Distributive	78.0–93.0	Low	97.0–98.0	High	3	Mostly RUSH. High variability in certainty of evidence of pooled sensitivities.	Yoshida [79] (2023)
Shock Obstructive	75.0–94.0	Low	80.0–99.0	High	4	Mostly RUSH. High variability in certainty of evidence of pooled sensitivities.	Stickles [78] (2019)
**Gastrointestinal**							
Acute Cholecystitis	69.0–81.0	Low	79.0–95.6	Moderate	7	All except one specificity estimate had low or very low certainty of evidence	Popat [110] (2024)
Appendicitis	56.0–87.1	Moderate	75.3–97.0	Moderate	15	Multiple reviews; range reflects between-review variability; Wide range across reviews	Kamal [105] (2026)
**Gynecological**							
Intrauterine pregnancy	86.0	Low	98.0	Moderate	1	POCUS by emergency physician. Substantial heterogeneity and inconsistency across sensitivity estimates	Celik [144] (2026)
**Neurological**							
Intracranial artery stenosis	90.0–96.0	Very low	94.0–95.0	Moderate	3	Transcranial Doppler and color-coded Duplex. Certainty of evidence always very low for sensitivity.	Mattioni [192] (2020)
**Ocular**							
Foreign body	100	Low	99.0	Moderate	1	Substantial heterogeneity and inconsistency across sensitivity estimates	Propst [207] (2019)
Globe rupture	100	Low	99.0	Moderate	1	Substantial heterogeneity and inconsistency across sensitivity estimates	Propst [207] (2019)
Lens dislocation	97.0	Low	99.0	Moderate	1	Substantial heterogeneity and inconsistency across sensitivity estimates	Propst [207] (2019)
**Respiratory**							
Acute respiratory failure	89.0–92.0	Very low	94.0–98.0	Moderate	2	Mostly intensive care unit	Jianguo [233] (2026)
Exaz Asthma/COPD	78.0–91.1	Low	88.4–94.0	Moderate	2	BLUE protocol	Staub [69] (2019)
Hemothorax	81.0	Moderate	99.0	High	1	Substantial variability in pooled sensitivity estimates	Staub [209] (2018)
Pneumothorax	79.4–97.4	Low	93.6–99.5	High	5	Multiple reviews; range reflects between-review variability	Asmara [73] (2022)
**c**							
**Condition**	**Sensitivity**		**Specificity**		**Number of reviews**	**Comment**	**Representative Study**
	**Range [%]**	**Certainty**	**Range [%]**	**Certainty**			
**Cardiovascular**							
LV diastolic function	94.0	High	59.0	Low	1	High heterogeneity and inconsistency for the pooled specificity	Huang [71] (2025)
**Gastrointestinal**							
Diverticulitis	92.0–93.9	High	85.9–94.0	Low	2	High heterogeneity in specificities in included studies within the reviews	Celik [130] (2025)
Small bowel obstruction	83.0–95.0	Moderate	77.0–96.0	Low	5	4 of 5 studies with moderate study quality.	Lin [124] (2021)
**Musculoskeletal**							
Abscess	94.8–96.2	High	82.9–85.2	Low	2	Moderate study quality, high inconsistency in both reviews for specificity	Barbic [182] (2017)
**Ocular**							
Posterior disorder	96.0	Moderate	94.0	Low	1		Wu [208] (2025)
Retinal detachment	92.0–94.0	Moderate	91.4–94.0	Low	2	POCUS of the eye, moderate study quality	Propst [207] (2019)

Clinical interpretation categories were assigned based on pooled sensitivity and specificity estimates together with certainty of evidence and heterogeneity across reviews. Potential rule-out applications were defined by sensitivity ≥ 90% with at least moderate certainty of evidence, whereas potential rule-in applications were defined by specificity ≥ 90% with at least moderate certainty. For conditions with multiple reviews, all pooled estimates were considered, and conditions were categorized as rule-in or rule-out supportive if at least one estimate fulfilled the respective criteria.

*Highest certainty of evidence corresponding to pooled sensitivity and/or specificity estimates > 90% is reported according to the respective classification (rule-in, rule-out, or rule-in/rule-out supportive). If multiple estimates fulfilled these criteria, the highest certainty of evidence is shown. If no estimate > 90% was available, the highest certainty of evidence across all available pooled estimates is reported

### Quality of included reviews

**Fig. 2:**
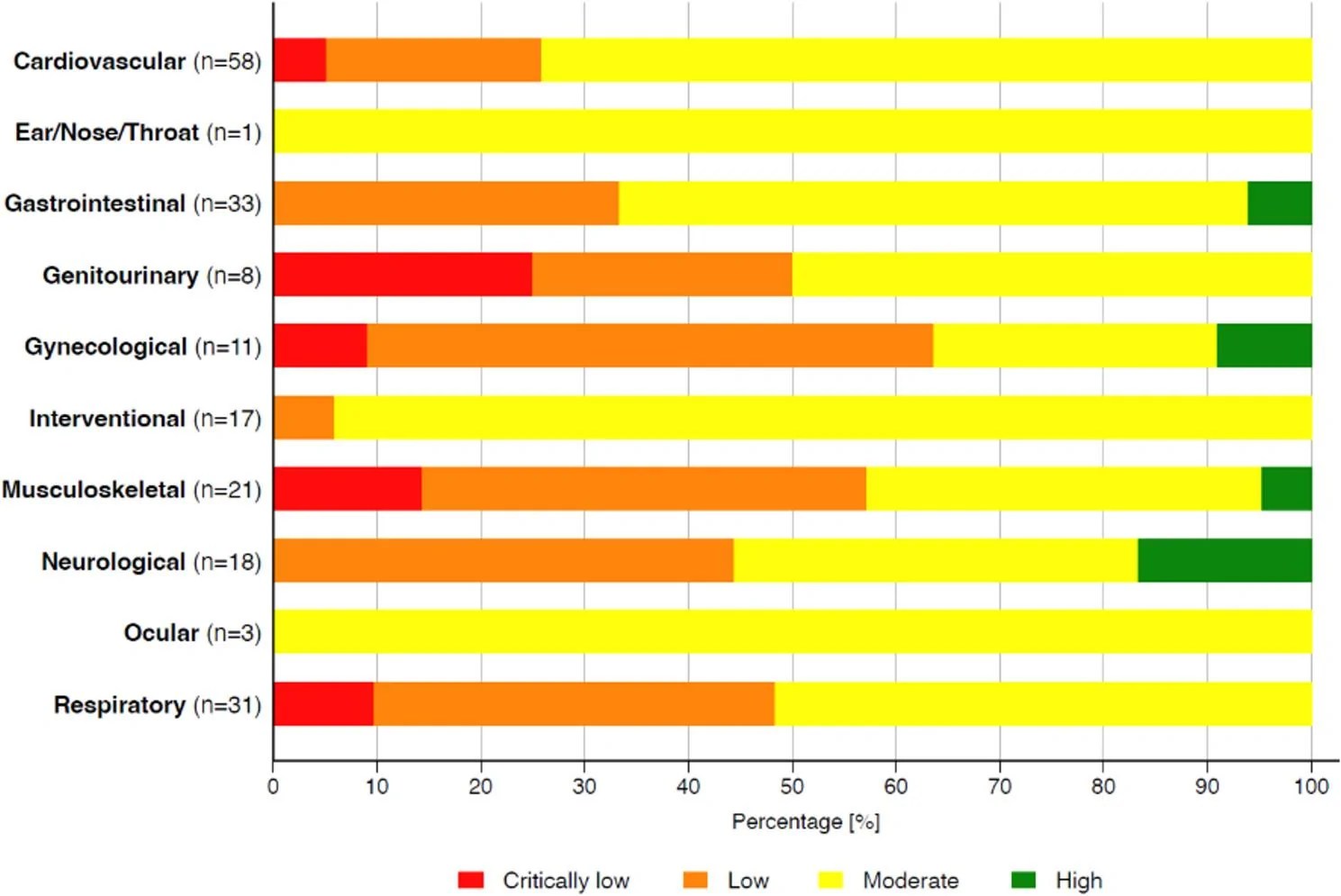
Study Quality (modified AMSTAR 2)

Using the modified AMSTAR 2 tool, 36.6% of the DTA-SR were rated as being of low or critically low, 59.9% of moderate and 3.6% of high quality. AMSTAR 2 item 7, requiring a comprehensive presentation of included and excluded studies with justification for exclusions, was fulfilled by only 18 of 197 included reviews (10.8%), while 149 reviews (89.2%) did not provide a transparent list of excluded studies. In our primary analysis, item 7 was treated as a non-critical domain; however, we additionally performed a sensitivity analysis using the original AMSTAR 2 classification in which item 7 was considered critical. In this sensitivity analysis, 93.4% of reviews were rated as low or critically low quality, 4.6% as moderate quality, and 2.0% as high quality. In 16.5% of the DTA-SR, studies with a high risk of bias were included in the analysis without discussion of the potential implications (critical item 13). No DTA-SR reported the funding sources of the included studies (non-critical item 10). The quality ratings are shown in Additional file 4: Tables S8a to S8j [41–237] with a summary in Fig. 2.

### Data synthesis

**Fig. 3:**
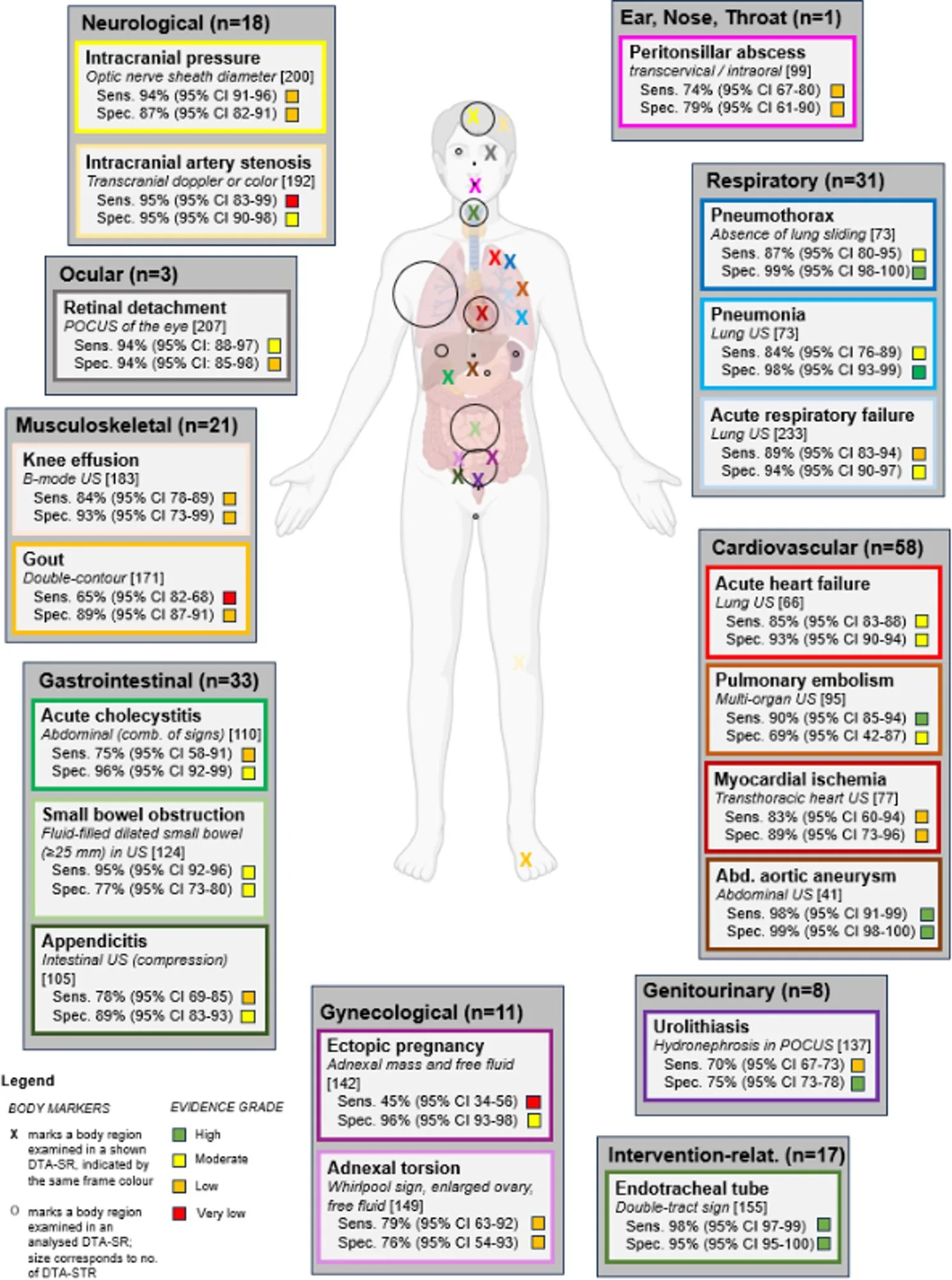
Figure of author selected highly relevant POCUS DTA-SR. Diagnostic accuracy with grading of the evidence of selected POCUS conditions of DTA-SR with MA in each studied domain (colored boxes) as well as examined body regions (circle body markers) of all included DTA-SR (*n* = 197). The number of identified DTA-SR in each domain is shown in brackets. As individual DTA-SR may contribute to more than one domain, the summed total across domains (*n* = 238) exceeds the overall number of included DTA-SR. The size of the circles corresponds to the number of DTA-SR examining these body region (for illustrative purposes, each DTA-SR was mapped to one specific body region). **Abbreviations**: Abd. = abdominal; CI = confidence interval; comb. = combination; DTA-SR = diagnostic test accuracy systematic review; POCUS = point-of-care ultrasound; relat. = related; Sens. = sensitivity; Spec. = specificity; US = Ultrasound

A clinically oriented summary of diagnostic utility across conditions is presented in Tables 2a–c and 3a–b, stratified according to rule-in supportive, rule-out supportive, combined rule-in/rule-out supportive, and adjunctive applications. Figure 3 provides an overview of selected representative conditions and evidence certainty. Among the 238 pooled sensitivities, 182 showed high heterogeneity; similarly, 178 of 238 pooled specificities were highly heterogeneous. In 33.2% (79/238) of the evaluated DTA pairs, the results could only be transferred to the ED setting to a limited extent. Moreover, imprecision was observed in 68.5% (163/238) of pooled sensitivities and 51.7% (123/238) of pooled specificities.

**Table 3:** (a) Adjunctive POCUS applications with moderate or high certainty of evidence. (b) Adjunctive POCUS applications very low or low certainty of evidence

a							
Condition	Sensitivity		Specificity		Number of reviews	Comment	Representative Study
	Range [%]	Certainty	Range [%]	Certainty			
**Cardiovascular**							
Aortic dissection	62.4	Low	87.5	Moderate	1		Sutarjono [45] (2023)
Cardiac arrest	19.4–91.6	Low	70.0–98.0	Moderate	7	Multiple reviews; range reflects between-review variability; Wide range across reviews	Albaroudi [49] (2024)
LV systolic function	89.0	Low	85.0	Moderate	1		Albaroudi [72] (2022)
Shock etiology: mixed	64.0–85.0	Moderate	95–98	Low	2		Stickles [78] (2019)
Significant coronary stenosis	85.0	Low	82.7	Moderate	1		Zarama [77] (2024)
**Gastrointestinal**							
Cholelithiasis	89.8–94.0	Moderate	88.0–93.0	High	2	Overall high certainty of evidence	Ross [122] (2011)
**Genitourinary**							
Hydronephrosis	81.7	Moderate	74.1	Low	1		Popat [110] (2024)
Urolithiasis	70.2–88.2	Low	75.4–79.2	High	2		Wong [137] (2018)
**Intervention related**							
Central venous catheter placement	68.2–82.0	Moderate	98.0-100	Moderate	4	Certainty generally low	Ablordeppey [162] (2017)
Endotracheal tube	78.0–98.7	High	75.0–98.0	High	7	Multiple reviews; range reflects between-review variability; Certainty generally low	Sahu [155] (2020)
Nasogastric tube placement	93.0	High	88.0–97.0	Moderate	2	Certainty generally low	Lin [166] (2017)
**Respiratory**							
COVID-19	86.9–96.0	Moderate	62.4–97.9	Low	4	Most studies with moderate methodological quality	Matthies [218] (2023)
**b**							
**Condition**	**Sensitivity**		**Specificity**		**Number of reviews**	**Comment**	**Representative Study**
	**Range [%]**	**Certainty**	**Range [%]**	**Certainty**			
**Cardiovascular**							
Acute coronary syndrome	93.2	Low	93.2	Low	1		Zarama [77] (2024)
Aortic dissection, Type B	56.8	Low	92.4	Low	1		Zaki [46] (2023)
Infective endocarditis	61.0	Low	94.0	Low	1		Bai [58] (2017)
Myocardial infarction	90.7	Low	67.4	Low	1		Zarama [77] (2024)
Myocardial ischemia	83.1	Low	89.2	Low	1		Zarama [77] (2024)
Native endocarditis	71.0	Very low	80.0	Very low	1		Zarama [77] (2024)
Pulseless electric activity	79.0–89.0	Low	58.0–73.0	Very low	3	All studies with moderate methodological quality	Jian [56] (2025)
Post-cardiac arrest neurology	77.0	Low	98.0	Low	1		Lee [57] (2019)
Prosthetic endocarditis	36.0	Very low	93.0	Very low	1		Habets [60] (2015)
**Ear/nose/throat**							
Peritonsillar abscess	74.0	Low	79.0	Low	1	POCUS, transcervical and intraoral, Overall small population	Kim [99] (2023)
**Gastrointestinal**							
Abd. Tuberculosis	63.0	Very low	68.0	Very low	1		Van Hoving [127] (2019)
Acute Pancreatitis	92.0	Very low	84.0	Very low	1		Fei [128] (2017)
Budd Chiari Syndrome	89.0	Very low	86.0	Very low	1		Gupta [129] (2020)
Choledocholithiasis	73.0	Very low	91.0	Very low	1		Ross [122] (2011)
Gallbladder disease	86.0	Very low	92.0	Very low	1		Wu [120] (2023)
Gastric content	95.0	Very low	88.0	Very low	1		Pan [132] (2024)
**Genitourinary**							
Acute kidney injury	76.0	Very low	79.0	Very low	1		Wei [139](2022)
Bladder outlet obstruction	68.0	Very low	91.0	Very low	1		Chen [138] (2023)
**Gynecological**							
Adnexal torsion	53.0–79.0	Low	76.0–95.0	Low	3	Wide range across reviews; Certainty generally low	Wattar [149] (2021)
Cesarian scar pregnancy	83.0	Low	74.0	Very low	1		Han [140] (2025)
Ovarian torsion	82.0	Very low	81.0	Very low	1		Adu-Bredu [150] (2021)
Retained products of conception	72.0–91.5	Very low	84.3–86	Low	2	High methodological quality	Sundararajan [146] (2024)
**Intervention related**							
Pred. diff. intubation	61.0–82.0	Low	77.0–88.0	Low	4	Certainty generally low	Bhargava [159] (2023)
**Musculoskeletal**							
CPPD	87.9–89	Very low	91.5–94.0	Very low	2	Certainty generally low. Critically low to low study quality.	Filippou [168] (2016)
Giant cell arteritis	67.0–83.0	Low	79.0–95.0	Low	4	Halo sign. Generally, very heterogenous primary studies.	Pouncey [177] (2024)
Gout	60.0–93.0	Very low	73.0–95.3	Low	6	Majority with low study quality. Very low certainty of evidence.	Lee [171] (2018)
Knee effusions	84.0	Low	93.0	Low	1		Meyer [183] (2022)
Large vessel vascul.	77.0–88.0	Very low	96.0	Very low	2	Only low study quality	Bosch [181] (2023)
Necrotizing skin inf.	93.2	Low	87.9	Low	1		Götz [184] (2023)
Retained foreign bodies	72.0	Very low	92.0	Low	1		Davis [185] (2015)
Rotator cuff tear	80.0	Low	85.0	Low	1		Dinnes [186] (2003)
Synovial abnormality	90.0	Very low	86.0	Very low	1		Liu [187] (2024)
**Neurological**							
Cauda equina syndrome	82.3	Very low	65.0	Low	1		Boktor [204] (2023)
Intracranial hemorrhage	88.0	Low	81.0	Low	1		Allen [205] (2023)
Intracranial masses	97.0	Low	99.0	Low	1		Allen [205] (2023)
Large vessel occlusion	87.5	Very low	95.0	Low	1		Jazayeri [194] (2025)
Neurodegenerative	80.0	Very low	71.0	Very low	1		Allen [205] (2023)
Stroke (Ischemic and hemorrhagic)	81.1	Very low	85.5	Very low	1		Pinto-Villalba [195] (2026)
Vertebral artery	70.2	Very low	93.4–97.7	Very low	2		Khan [196] (2007)
**Ocular**							
Vitreous detachment	67.0	Low	89.0	Low	1		Propst [207] (2019)
Vitreous hemorrhage	90.0	Low	92.0	Low	1		Propst [207] (2019)
**Respiratory**							
Acute dyspnea	85.6	Low	80.8	Low	1		Seyala [232] (2026)
Acute respiratory distress syndrome	63.1–84.0	Low	87.0–94.2	Low	3	All studies with moderate methodological quality	Ji [236] (2026)
Iatrog. Pneumothorax	93.0	Very low	97.0	Low	1		Alrajhi [210] (2012)

Clinical interpretation categories were assigned based on pooled sensitivity and specificity estimates together with certainty of evidence and heterogeneity across reviews. Conditions were categorized as adjunctive applications if they did not fulfill predefined criteria for rule-in or rule-out supportive use

*As no pooled sensitivity or specificity estimate fulfilled rule-in or rule-out criteria, the highest certainty of evidence across all available pooled estimates is reported

Detailed information about each category with every extracted pooled accuracy pair is given in Supplement Tables 8a to 8k.

Diagnostic utility varied substantially across conditions. Of 91 evaluated conditions, 12 met criteria for combined rule-in/rule-out supportive applications, 17 for rule-in support only, and 6 for rule-out support only (defined as pooled sensitivity or specificity ≥ 90% with at least moderate certainty of evidence). These categorizations are summarized in Tables 2a–c and 3a–b.

**Fig. 4:**
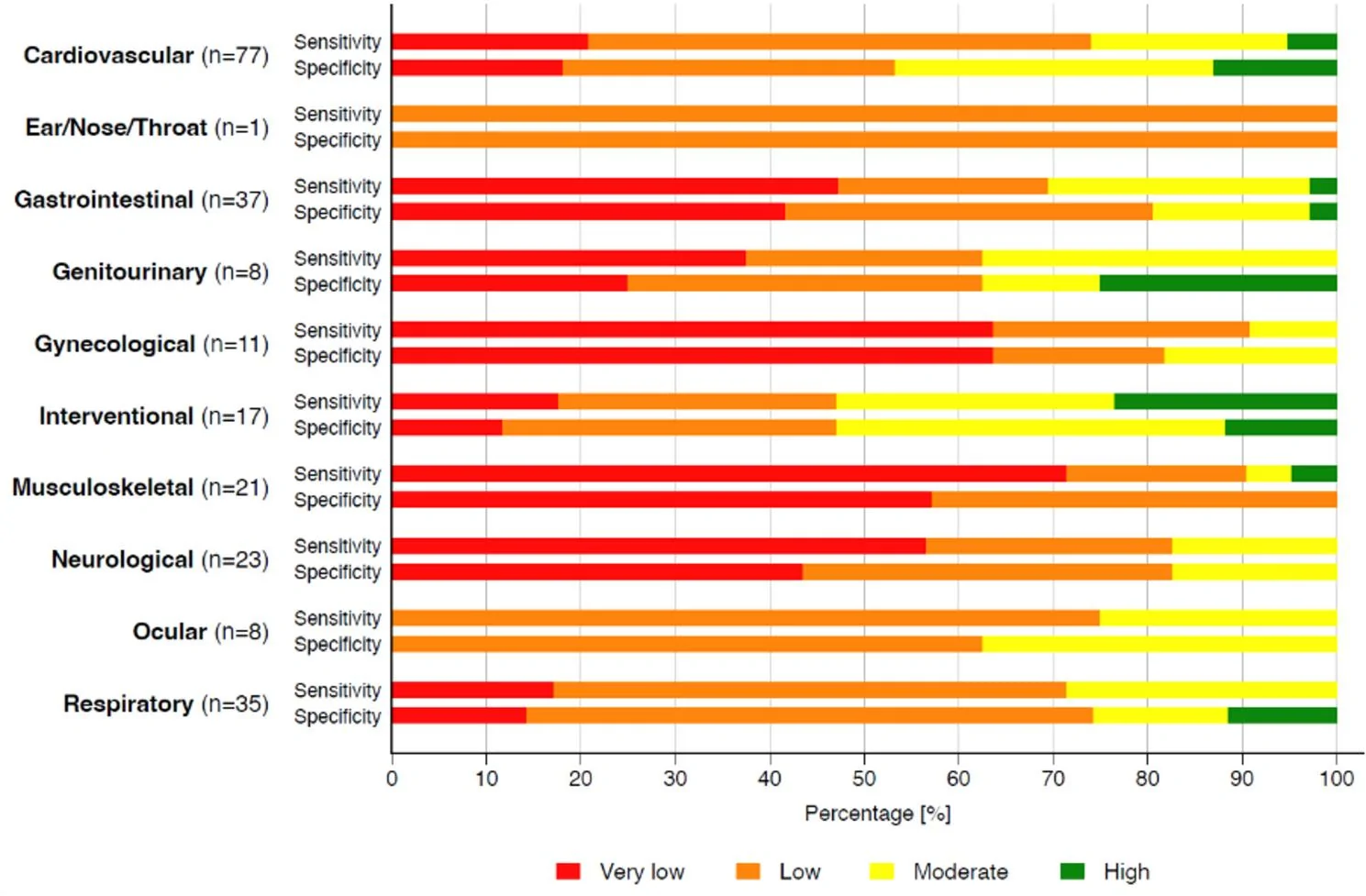
Certainty of evidence for each domain using GRADE. GRADE of pooled sensitivity and specificity per domain with hierarchical selection of DTA pair in case of multiple pairs (ED/POCUS focused, multi-organ US, number of primary studies included)

A critical review of diagnostic accuracy and GRADE assessments for all identified POCUS conditions, especially those examined in multiple DTA-SR, is presented in Additional file 5 [45, 46, 49–56, 59, 60, 63, 65, 71, 72, 76–79, 99, 134–143, 147–169, 188–196, 201, 232, 233]. A fact sheet for each included study condition is shown in Additional file 6 [41–237]. A summary of the GRADE assessment is shown in Fig. 4.

## Discussion

### Summary of main results

This umbrella review synthesized evidence from 197 SR with MA assessing the diagnostic test accuracy of POCUS across 91 non-traumatic, emergency medicine-relevant conditions spanning cardiovascular, respiratory, gastrointestinal, neurological, musculoskeletal, genitourinary, gynecological, ocular, ear/nose/throat, and intervention-related domains. Diagnostic accuracy varied widely depending on the condition assessed, but overall POCUS demonstrated moderate to high sensitivity and high to very high specificity, although the certainty of evidence was frequently low or very low.

Applying predefined thresholds for diagnostic utility to facilitate clinically oriented interpretation (pooled sensitivity or specificity ≥ 90% with at least moderate certainty of evidence) revealed that only a subset of evaluated conditions demonstrated sufficient diagnostic accuracy and certainty of evidence to support rule-in and/or rule-out applications. Specifically, combined rule-in/rule-out supportive potential was identified in 12 conditions, whereas 17 conditions supported primarily rule-in and 6 primarily rule-out applications. However, these categories should not be interpreted as fixed diagnostic recommendations, as clinical utility depends strongly on pre-test probability, disease prevalence, operator expertise, and overall clinical context. Consequently, the same POCUS application may function predominantly as a rule-in tool in selected populations while primarily supporting diagnostic reasoning as an adjunctive application in other settings.

POCUS demonstrated its strongest diagnostic performance in acute, well-defined conditions with clear sonographic targets. Nearly perfect accuracy was consistently reported for abdominal aortic aneurysm and endotracheal tube confirmation, supported by moderate- to high-quality evidence. Similarly, high sensitivities and specificities, often exceeding 90%, were reported for cholelithiasis, small bowel obstruction, deep vein thrombosis, pulmonary edema, pleural effusion, optic nerve sheath diameter for raised intracranial pressure, cerebrovascular stenosis or occlusion, testicular torsion and nasogastric tube placement. In these contexts, POCUS appears to be a reliable tool for rapid rule-in and, in some cases, rule-out decision-making in emergency medicine settings.

In contrast, diagnostic accuracy was consistently lower and more variable in clinically complex, heterogeneous, or diffuse disease entities. Conditions such as aortic dissection, endocarditis, shock etiology, pulmonary embolism, hydronephrosis in urolithiasis, vertebral artery stenosis, neurodegenerative disease, and COVID-19 pneumonia were characterized by limited sensitivity, wide ranges of specificity, and predominantly low or very low certainty of evidence. For these conditions, POCUS may support early diagnostic reasoning or help rule-in disease within structured protocols, but it cannot be relied upon as a stand-alone diagnostic modality.

Observed variability across reviews was likely influenced by differences in patient populations, ultrasound protocols, operator expertise, diagnostic thresholds, and reference standards. Furthermore, our findings should be interpreted in the context of heterogeneous study settings and operator backgrounds, which may differ from real-world emergency medicine department POCUS practice.

### Overall completeness and applicability of evidence

Ultrasound is a versatile adjunct to diagnose many conditions in the ED. Our search identified 91 unique conditions, including those assessed in non-ED or specialist settings. This approach reflects the limited availability of ED-specific diagnostic accuracy data for many POCUS indications and allowed for a broader overview of clinically relevant conditions. However, it also limits direct applicability to emergency practice, as not all included examinations represent true POCUS or were performed by emergency physicians, and may contribute to heterogeneity in diagnostic performance. As a result, a substantial proportion of the included evidence does not fully reflect POCUS as performed in emergency medicine settings, which may limit both the internal consistency of the applicability assessment and the direct clinical applicability of the reported diagnostic accuracy estimates.

### Quality of the evidence

The quality of the included reviews varied. While most of the reviews (59.9%) showed moderate methodological quality using an adapted version of the AMSTAR 2 checklist, the quality of only five (3.0%) reviews was rated as high. A major contributor to lower methodological ratings was the frequent absence of transparent reporting of excluded studies (AMSTAR 2 item 7). However, this finding should be interpreted in the context of evolving reporting standards, as detailed exclusion lists were not consistently required before the introduction of AMSTAR 2 and PRISMA 2020 and appear to have been less commonly reported in DTA reviews than in interventional systematic reviews. Notably, sensitivity analysis using the AMSTAR 2 classification with item 7 retained as a critical domain resulted in a marked shift toward lower methodological quality ratings, highlighting the substantial influence of this adaptation on overall quality assessment. Nevertheless, more transparent reporting of excluded studies in future DTA reviews would be desirable to improve methodological rigor and facilitate interpretation.

An additional limitation is the inclusion of studies with high risk of bias in the analyses. Future reviews should therefore prioritize methodological quality over quantity of included studies. The lack of reporting on funding sources was considered of lesser relevance in diagnostic accuracy research, and the justification for downgrading reviews based on this criterion remains debatable.

For the strength of evidence, we used the GRADE approach. Most DTA pairs showed very low to low certainty of evidence. This is not surprising as the first domain (risk of bias) was informed by the corresponding AMSTAR 2 result, with a critically low or low score leading to downgrading. Many of the included reviews in this overview reported significant between-study heterogeneity. Importantly, heterogeneity in DTA SR should not be interpreted in the same way as heterogeneity in intervention research. Variability in pooled sensitivity and specificity is expected in DTA MA and may reflect threshold effects, differences in patient spectrum (e.g., case mix, disease prevalence, or severity), variation in operator training and experience, ultrasound scanning protocols, use of different reference standards, or differences in clinical setting rather than methodological shortcomings alone. Consequently, heterogeneity in DTA research does not necessarily indicate low-quality evidence but still requires careful exploration and transparent reporting, as inadequate assessment may limit interpretability and confidence in pooled estimates. Only a few reviews assessed the extent and causes of the reported heterogeneity appropriately. White et al. came to a similar conclusion in their review on heterogeneity in medical imaging DTA-SR reporting, showing that many reviews appeared to evaluate heterogeneity incompletely, inconsistently, or methodologically questionable approaches [238].

Some of the included reviews in this overview were downgraded for indirectness. This was mostly due to our attempt to include as many emergency medicine-relevant conditions as possible, and thus including reviews with settings outside the ED. In the cardiovascular domain, an additional source of indirectness arises from the use of formal transthoracic echocardiography (TTE) as the index test in several included reviews. TTE represents a comprehensive, specialist-performed imaging modality and is not equivalent to focused cardiac POCUS. Where possible, we prioritized reviews specifically evaluating POCUS protocols. However, in some cases, primary studies within systematic reviews combined POCUS and comprehensive echocardiography, limiting the ability to isolate POCUS-specific diagnostic performance. This should be considered when interpreting results for cardiovascular conditions.

Most included reviews were downgraded for imprecision, whereas substantial heterogeneity among underlying primary studies frequently contributed to inconsistency ratings. Such heterogeneity may stem from differences in patient spectrum (e.g., case mix, disease prevalence, or severity), variation in index test performance or operator expertise, use of different reference standards, threshold effects, or differences in clinical setting and study design [238, 239].

Traditionally, publication bias is the fifth domain of the GRADE approach, in particular for SR for interventions. However, methods to detect publication bias in DTA studies remain limited [40, 240]. Unlike intervention studies, diagnostic accuracy measures such as sensitivity and specificity are interrelated and threshold-dependent, making conventional approaches such as funnel plot asymmetry testing difficult to interpret and insufficiently validated for DTA research. Current GRADE guidance therefore cautions against inappropriate downgrading based on non-validated publication bias methods in DTA reviews. Accordingly, publication bias was not formally assessed in this overview but was considered indirectly within the overall risk of bias assessment and AMSTAR 2 appraisal.

### Strengths and Weaknesses

This overview has several strengths. First, it adhered to *Cochrane* methodological principles and followed a prespecified protocol, enhancing transparency and reproducibility. Second, a comprehensive systematic literature search was conducted across two major databases, supplemented by screening of the most relevant records retrieved through Elicit and Google Scholar, as well as backward citation searching of all included studies, to minimize the risk of omitting relevant systematic reviews. We did not restrict inclusion to ED-only settings but included reviews addressing conditions relevant to daily emergency practice. This broader approach increased the completeness of identified conditions and was further supported by involvement of content experts during screening. Third, the broad scope of this review, encompassing 91 conditions across ten domains, represents a major strength by providing a comprehensive overview of emergency medicine-relevant POCUS applications. Unlike previous reviews focusing on single organ systems or selected indications, this approach allowed identification of overarching patterns in diagnostic performance, certainty of evidence, and methodological limitations across diverse emergency medicine conditions. At the same time, this breadth constrained the depth of condition-specific appraisal and may have obscured important clinical and methodological nuances. Fourth, dual methodological appraisal using an adapted AMSTAR 2 tool and the GRADE framework allowed structured assessment of both methodological rigor and certainty of evidence. Fifth, categorization of conditions according to their potential rule-in and rule-out utility enhanced the clinical interpretability of findings by moving beyond diagnostic accuracy estimates alone toward a more practice-oriented understanding of POCUS applications.

However, several limitations must be acknowledged. First, the restriction of the scope to non-trauma conditions represents a post hoc deviation from the original protocol, which was not formally documented as a PROSPERO amendment. This decision reduced the comprehensiveness of the review, particularly with regard to trauma-related POCUS applications, and may have influenced the overall representation and balance of POCUS use in emergency care. Second, the applicability assessment was based on pragmatic expert consensus rather than exclusively on predefined eligibility criteria for POCUS indications. Although this approach allowed flexible identification of clinically relevant emergency ultrasound applications, it may have introduced subjectivity into study selection. Third, AMSTAR 2 item 7 (provision of a list of excluded studies) was downgraded from a critical to a non-critical domain, representing a pragmatic deviation from the original tool in light of historically less consistent reporting in DTA-SR than in interventional systematic reviews. Fourth, inclusion of reviews from non-ED or specialist settings limits direct applicability to emergency practice, as not all examinations reflect true point-of-care use. Furthermore, operator competence, including training level, certification status, and ultrasound experience, was inconsistently reported across the included reviews and primary studies, precluding systematic evaluation of its impact on diagnostic performance. Variability in operator expertise may contribute to heterogeneity and limit the generalizability and applicability of findings across clinical settings. Fifth, in the cardiovascular domain, the use of formal TTE in some reviews limits applicability to POCUS, as it is not equivalent to focused bedside ultrasound. Sixth, AI-assisted ultrasound studies were excluded, as their performance reflects model-specific characteristics rather than intrinsic test accuracy, which may limit applicability to emerging AI-supported POCUS. Finally, most included SR were of moderate or low methodological quality, and substantial heterogeneity across populations, protocols, and reference standards limits the strength and generalizability of conclusions. Moreover, restriction to English and German publications and the absence of a grey literature search may have introduced language and publication bias.

### Agreements and disagreements with other studies and/or reviews

To our knowledge, this is the first overview of SR with MA synthesizing diagnostic test accuracy of ultrasound across emergency medicine-relevant conditions while formally assessing methodological quality and certainty of evidence.

The conceptual role of POCUS has been prominently described by Díaz-Gómez et al. (2021), who characterize POCUS as a rapidly deployable bedside tool that enhances diagnostic reasoning and procedural safety, while cautioning against overextension in complex conditions [241]. Our findings align with this conceptual framework. By quantifying pooled accuracy and grading certainty of evidence, we extend this rationale and identify conditions where robust diagnostic performance supports rule-in strategies, and others where performance remains heterogeneous and less certain. Narrative and critical reviews focusing on emergency medicine applications similarly describe POCUS as broadly valuable across acute care indications [9, 242]. In contrast to descriptive syntheses, our overview provides pooled diagnostic accuracy estimates alongside grade certainty of evidence, allowing a more differentiated assessment of clinical reliability. Across organ systems a consistent pattern emerges: specificity is often high, whereas sensitivity is more variable and certainty of evidence is frequently low to very low.

Ceriani et al. highlight implementation constraints, particularly the need for adequate training and quality assurance [242]. In our overview, operator training was frequently insufficiently reported, limiting generalizability and underscoring the importance of structured education and standardized protocols for safe integration into clinical practice. Moreover, numerous guideline and consensus documents recommend indications that closely mirror those identified in our results, while our synthesis adds granularity by delineating diagnostic accuracy and certainty of evidence supporting these recommendations [243–247].

Consistent with this pattern, our pooled estimates consistently show that POCUS performs best for conditions with clear structural signatures and limited interpretive ambiguity. For example, ocular application shows high pooled accuracy for retinal detachment, globe rupture, foreign body or lens dislocation with little heterogeneity [206, 207], a pattern that is echoed in an emergency eye ultrasound narrative review describing POCUS as a rapid adjunct when fundoscopy is limited [248]. Similarly, abdominal aortic aneurysm represents a prototypical “structure-first” emergency diagnosis: both pooled MA estimates in our overview and established clinical reviews and guidelines describe ultrasound as the preferred initial imaging modality, with very high sensitivity and specificity for aneurysm detection [41–44, 249]. This convergence across umbrella evidence, organ-specific reviews, and guideline recommendations supports a practical “rule-in first” paradigm in emergency ultrasound.

Our respiratory summary shows generally high sensitivity and very high specificity for several thoracic conditions (e.g., pneumothorax [73, 209–213], pleural effusion [214, 215]) and pneumonia [69, 73, 220–231] with estimates varying by scanning protocol and reference standard. This is consistent with critical care literature emphasizing that lung ultrasound frequently matches or outperforms chest radiography for common thoracic pathologies such as pleural abnormalities, pneumothorax, pulmonary oedema, and pneumonia, and that protocols such as the BLUE protocol have been widely described for systematic assessment at the bedside [250]. Variation in scanning protocols and comparator tests plausibly explains why specificity estimates fluctuate across studies and settings, consistent with the heterogeneity noted in our pooled respiratory results.

Narrative overviews of point-of-care abdominal ultrasound, such as the comprehensive review by Kameda and Taniguchi, describe POCUS as a promising tool in emergency medicine and critical care for a broad spectrum of intra-abdominal conditions, including gallstone disease, acute cholecystitis, ureterolithiasis, appendicitis, small bowel obstruction, and abdominal aortic aneurysm [251]. Our umbrella findings align with this clinical perspective by demonstrating consistently high diagnostic accuracy for structurally definable targets such as gallstones [120]. At the same time, in concordance with narrative accounts, we confirm that diagnostic performance for inflammatory or multifactorial conditions such as appendicitis [100–114] and cholecystitis [110, 115–120] is more variable and remains limited by heterogeneity and evidence quality. Notably, some authors argue that the distinction between conclusive and non-conclusive ultrasound examinations (e.g. in appendicitis) is pivotal for interpreting diagnostic performance, highlighting the need for more rigorous conceptualization and methodological treatment of conclusive ultrasound findings in future research [252].

Our musculoskeletal findings are consistent with the narrative overview by Ciechomska et al., which describes ultrasound-detected features such as the double contour sign and tophi as morphologically well-defined lesions incorporated into ACR/EULAR classification criteria [253]. This corresponds with our pooled results showing high specificity but variable sensitivity and very low certainty of evidence, supporting a rule-in rather than rule-out role in crystal arthropathies [168–175]. For giant cell arteritis, the same review emphasizes the halo sign within structured, probability-based diagnostic pathways, aligning with our finding of high specificity and moderate sensitivity and supporting an adjunctive application [177–180]. In contrast, for other musculoskeletal conditions, although clinical utility is described, our synthesis reveals considerable heterogeneity and low certainty of evidence, limiting confidence in standalone use.

Our neurological findings are consistent with contemporary Neuro-POCUS concepts, which position transcranial and cranial ultrasound as adjunctive bedside tools rather than replacements for CT or MRI [254, 255]. Reports of good concordance with CT for selected structural targets, such as larger hemorrhage or midline shift, align with our observation of acceptable accuracy for specific indications but overall low certainty of evidence [255]. Likewise, transcranial Doppler is described as valuable in stroke and vasospasm monitoring while remaining complementary to advanced imaging [254]. Our findings of moderate sensitivity for vascular stenosis and heterogeneous performance of optic nerve sheath diameter further support this focused, adjunctive role rather than standalone exclusion.

Importantly, high diagnostic accuracy does not automatically translate into clinical benefit. However, in domains where our overview identified consistently high or very high accuracy, particularly cardiopulmonary and vascular indications, emerging outcome data suggest that this translation can occur. In patients with acute dyspnea, POCUS-guided management has been associated with faster diagnosis, earlier initiation of appropriate therapy, and reduced ICU utilization [256, 257]. In shock, a recent MA of randomized trials demonstrated that POCUS-guided resuscitation likely reduces 28-day mortality (RR 0.88) and shortens the duration of vasoactive support, with moderate certainty of evidence [258].

Diagnostic studies assess discrimination, not effectiveness within care pathways. Translating accuracy into improved outcomes therefore requires identifying which conditions are truly suitable for ultrasound-based rule-in or rule-out strategies. Our findings indicate that clinical benefit is most plausible where sonographic targets are discrete and physiologically decisive, whereas heterogeneous or indirectly defined conditions are less suited for standalone POCUS decision-making. By systematically synthesizing diagnostic performance and grading certainty of evidence, our overview helps delineate where POCUS can be expected to deliver reliable clinical value and where further validation is needed before outcome gains can be assumed.

Thus, although the overall certainty of evidence remains limited across several indications, current outcome data indicate that when diagnostic performance is robust, clinically meaningful benefit can be achieved. This reinforces the promise of POCUS as a rapid, bedside modality in acute care, while highlighting the need for higher-quality research to define its optimal scope and application.

In addition, AI represents an emerging and rapidly evolving field in POCUS, with increasing interest in its potential to support image acquisition, interpretation, and clinical decision-making. Recent evidence suggests that AI-assisted POCUS may reduce operator dependency, improve standardization, and support less experienced users [259, 260]. Across specific applications, AI-assisted POCUS has demonstrated promising diagnostic performance. For example, AI-based assessment of left ventricular ejection fraction showed good agreement with reference standards and acceptable classification performance, with sensitivities ranging from 70% to 93% and specificities from 89% to 100% [261]. Similarly, a recent MA reported high DA for AI-based detection of pleural effusions on ultrasound (sensitivity 92%, specificity 96%), which is comparable to the diagnostic accuracy estimates identified in our umbrella review for pleural effusion (sensitivity 93–94.5% and specificity 96–97.9%) [262]. However, the current evidence base is still limited by early-phase study designs, heterogeneous methodologies, and a lack of external validation. Furthermore, AI-based performance reflects model- and dataset-specific characteristics rather than the intrinsic diagnostic accuracy of a standardized clinical test, limiting comparability with conventional diagnostic test accuracy studies. For this reason, AI-based studies were not included in the present umbrella review. Nevertheless, given the rapid development of this field, AI-assisted POCUS is likely to play an important role in future emergency care, and further high-quality, clinically integrated evidence syntheses are warranted.

Overall, while AI-assisted POCUS is a promising and rapidly evolving field, the current evidence remains insufficient for inclusion within the scope of this umbrella review and highlights an important area for future research and clinical integration.

## Conclusions

### Implication for practice

This overview provides a structured and comprehensive synthesis of existing SR on the diagnostic accuracy of POCUS in emergency medicine comprising evidence from 197 reviews evaluating 91 unique conditions. POCUS shows promising diagnostic accuracy across a wide range of adult emergency medicine-relevant conditions, with oftentimes moderate to high sensitivity and very high specificity. However, the overall strength of evidence remains limited and heterogeneous, and a substantial proportion of the underlying data originates from non-ED or specialist settings. Therefore, diagnostic performance estimates should be interpreted within the context of the specific clinical condition, pre-test probability, overall clinical assessment, and intended diagnostic application, rather than being considered directly comparable across different indications.

### Implication for research

Despite the large and growing body of evidence synthesized in this overview, important uncertainties remain. The underlying evidence was frequently of limited quality, with more than half of DTA-SR graded as low or very low certainty and most reviews demonstrating only moderate methodological quality. Evidence for syndromal presentations was scarce, as most studies focused on well-defined conditions rather than complex clinical scenarios commonly encountered in emergency medicine. Accordingly, key research priorities include high-quality prospective studies in syndromal presentations, standardization of scanning protocols, improved reporting of operator competence, and validation of emerging applications such as Doppler-based and AI-assisted POCUS. Notably, despite increasing clinical interest, we did not identify eligible DTA systematic reviews with meta-analysis for advanced Doppler applications such as venous congestion assessment (e.g., VExUS) or stroke volume estimation (e.g., LVOT VTI), highlighting an important evidence gap. Improving the methodological quality, consistency, and clinical applicability of future studies will be essential to provide more robust evidence guiding POCUS use in emergency medicine practice.

In summary, POCUS remains a highly promising diagnostic modality for emergency medicine. However, given the limited and heterogeneous evidence base, its diagnostic performance cannot yet be generalized across different clinical conditions without careful contextual interpretation. Higher-quality research is required to define its true diagnostic value and optimal role in acute care.

## Supplementary Information

Below is the link to the electronic supplementary material.

Supplementary Material 1: Additional file 1: Table S1–Table S5.

Supplementary Material 2: Additional file 2: Excluded reviews during assessment of emergency medicine POCUS conditions. Lists systematic reviews excluded during assessment of emergency medicine POCUS applicability after methodological eligibility had been established.

Supplementary Material 3: Additional file 3: Characteristics and reasons for exclusion of full-text articles. Provides the characteristics of all reviews excluded after full-text assessment together with the specific reason for exclusion.

Supplementary Material 4: Additional file 4: Table S8a–Table S8j.

Supplementary Material 5: Additional file 5: Critical synthesis of the included reviews. Provides a structured narrative synthesis of the evidence for each clinical condition, highlighting methodological differences between reviews, certainty of evidence, and implications for clinical practice.

Supplementary Material 6: Additional file 6: Fact sheets for each included review. Contains standardized evidence summaries for every included systematic review, including clinical context, index test, reference standard, methodological characteristics, pooled diagnostic accuracy estimates, GRADE certainty of evidence, methodological quality assessment, subgroup analyses, and the original authors' conclusions.

## Data Availability

All references of the included articles after the title and abstract screening can be found in the main manuscript or the supplementary material.

## Abbreviations

AAA: Abdominal Aortic Aneurysm
ACR: American College of Radiology
AMSTAR 2: A Measurement Tool to Assess Systematic Reviews 2
ARDS: Acute Respiratory Distress Syndrome
BLUE: protocol Bedside Lung Ultrasound in Emergency protocol
CCA: Corrected covered area
CEUS: Contrast-Enhanced Ultrasound
CI: Confidence Interval
COPD: Chronic Obstructive Pulmonary Disease
COVID-19: Coronavirus Disease 2019
CPR: Cardiopulmonary Resuscitation
CT: Computed Tomography
CTA: Computed Tomography Angiography
CTPA: Computed Tomography Pulmonary Angiography
DTA: Diagnostic Test Accuracy
DTA-SR: Diagnostic Test Accuracy Systematic Review
DSA: Digital Subtraction Angiography
ED: Emergency Department
GRADE: Grading of Recommendations Assessment, Development and Evaluation
IA: Intra-arterial
ICP: Intracranial Pressure
ICU: Intensive Care Unit
MA: Meta-analysis
MRA: Magnetic Resonance Angiography
MRI: Magnetic Resonance Imaging
NA: Not Available
POCUS: Point-of-Care Ultrasound
RADUS: Radiology-Performed Ultrasound
RUSH: Rapid Ultrasound in Shock
SE: Standard Error
Sens.: Sensitivity
Spec.: Specificity
SR: Systematic Review
TEE: Transesophageal Echocardiography
TTE: Transthoracic Echocardiography
US: Ultrasound
