# Supplementary material for: Diagnostic accuracy of point-of-care ultrasound in adult non-traumatic emergency medicine: an umbrella review
Source: BMC Med. 2026 Jul 25;24:579. doi: 10.1186/s12916-026-05094-2 (PMC13689006; doi:10.1186/s12916-026-05094-2)
Supplement: Supplementary file 1 [file 12916_2026_5094_MOESM1_ESM.docx]

## Additional file 5: Critical synthesis of the included reviews

Details are summarized across reviews; representative studies are cited where relevant.

##### Cardiovascular

We identified 58 diagnostic test accuracy systematic reviews (DTA SRs) assessing the diagnostic accuracy of ultrasound for aortic disease, shock or cardiopulmonary resuscitation related conditions, endocarditis, heart failure, and venous thromboembolisms.

Sensitivity and Specificity of ultrasound for abdominal aortic aneurysms including ruptured abdominal aortic aneurysm (defined as aneurysm on ultrasound plus pain) were very high in all four reviews (97.5-99.0% and 97.0-100%, respectively), making ultrasound an effective tool to both rule-in and -out abdominal aortic aneurysm. Certainty of evidence was moderate to high. The diagnosis of aortic dissection, Sutarjono et al. demonstrated limited sensitivity (62.4%) and high specificity (87.0%).[45] Zaki et al. compared the subgroups of Type A and Type B dissection and found that ultrasound has the higher sensitivity for Type A dissection (86.7% vs 56.8%) and higher specificity for type B dissection (92.4% vs. 86.5%).[46] Certainty of evidence remains low across all subgroups. Ultrasound should therefore not be used exclusively to rule out aortic dissection but is a valuable tool to rule in aortic dissection, especially for type B dissection, when positive findings are present and aligned with clinical suspicion.

Eight reviews evaluated ultrasound in the prediction of survival of cardiac arrest patients. Accuracy measures varied widely with sensitivities from 19.4-91.6% and specificities from 58.0–98.0%, respectively. Certainty of evidence was mostly low. Reasons for these accuracy ranges were differences in population, index test, and reference standards. For example, Blyth et al, Breglia et al, Dudek et al, Jian et al, and Lalande et al. (2019) defined a true positive test result as cardiac activity (outcome return of spontaneous circulation, ROSC) whereas a true positive test for Albaroudi et al, and Lalande et al. (2021) was absence of cardiac activity (outcome death), i.e. the direction of the index test definition differed across reviews.[49–56]

For determination of shock etiology, ultrasound showed varying sensitivity (64.0-85.0% for mixed shock etiology to 78.0-93.0% for distributive shock) and high to very high specificity (80.0-99.0% for obstructive shock to 97.0-98.0% for distributive shock). Stickles et al. generally reported lower pooled sensitivity than Yoshida et al., whereas pooled specificity was similar.[78, 79] This could be explained by Stickles et al. using Rapid Ultrasound for Shock and Hypotension (RUSH) or an equivalent protocol as index test versus RUSH exclusively. Nevertheless, ultrasound can be confidently used as a rule-in tool in all etiologies of shock (given consistently high specificity), while rule-out performance depends on etiology and protocol.

Bai et al. showed poor sensitivity (61.0%) but very high specificity (94.0%) for the diagnosis of infective endocarditis using transthoracic echocardiography (TTE), though certainty of evidence was low. Bonzi et al. found higher sensitivity (71.0%) but lower specificity (80.0%) in patients with native valve endocarditis.[59] For prosthetic valve endocarditis Habets et al. found very low sensitivity (36.0%) and very high specificity (93.0%).[60] Certainty of evidence was very low to low across all three reviews and therefore limits confidence in rule-out strategies.

Ten DTA SRs assessed the diagnostic accuracy of lung ultrasound (LUS) for the diagnosis of acute heart failure. Overall, sensitivity ranged from 73.0-94.1%, and specificity from 81.8-96.0%, respectively. Gartlehner et al. and Lv et al. specifically presented the highest specificity of 96.0% with moderate sensitivity of 76.0% in examinations performed by internists or emergency physicians.[63, 65] Ultrasound protocols and reference standards varied between DTA SRs explaining the differences of accuracy measures. Better accuracy was seen for the diagnosis of pulmonary edema with high to very high sensitivity (89.0-97.0%) and specificity (87.0-98.0%), respectively. Wang et al. showed highest sensitivity and specificity, though methodological quality was rated critically low and certainty of evidence low (suggesting potential overestimation of accuracy).[76]

When looking at left ventricular (LV) systolic function exclusively, Albaroudi et al. found that visual estimation by an emergency physician had high sensitivity and specificity (89.0%, 85.0%) compared to visual estimation by an expert (reference standard).[72] Huang et al. found very high sensitivity (94%) but only low specificity (59%) for diagnosing left ventricular diastolic dysfunction using focused cardiac ultrasound performed by emergency physicians, suggesting that this application may be useful for ruling out clinically relevant dysfunction but has limited utility for confirming the diagnosis.[71]

Zarama et al. reported variable diagnostic accuracy of cardiac ultrasound (TTE, limited TTE, and POCUS) across ischemic cardiac conditions.[77] Myocardial ischemia showed high sensitivity (83.1%) and specificity (89.2%), whereas myocardial infarction demonstrated very high sensitivity (90.7%) but only moderate specificity (67.4%). In contrast, acute coronary syndrome (ACS) showed moderate sensitivity (68.0%) and very high specificity (93.2%), suggesting stronger rule-in than rule-out potential. Significant coronary stenosis demonstrated high sensitivity (85.0%) and specificity (82.7%). Overall, certainty of evidence was low despite moderate methodological quality, supporting the use of cardiac POCUS primarily as an adjunctive diagnostic tool.

For the diagnosis of deep vein thrombosis, seven DTA SRs showed mostly very high accuracy (sensitivity 89.7–96.1%, specificity 93.8–98.7%). Four reviews specifically evaluated point-of-care ultrasound (POCUS) by emergency physicians compared to radiology performed ultrasound (RADUS), angiology, or a vascular department. All of these reviews demonstrated very high sensitivity (90.0-96.1%) and specificity (95.0-98.0%), respectively, making POCUS a suitable diagnostic tool to both rule in and out deep vein thrombosis.

For pulmonary embolism sensitivities and specificities varied widely (sensitivity 65.5–91.8%, specificity 63.9–99.0%) with mostly very low to low certainty of evidence. This is probably explained by diverse ultrasound protocols, and reference standards. There is a trend of higher specificity than sensitivity in DTA SRs using LUS, though certainty of evidence varies widely from very low to high. Given the current evidence, ultrasound cannot be recommended to rule out but could help to rule in pulmonary embolism following certain protocols.

##### Ear/nose/throat

Only one DTA SR was included assessing the diagnostic accuracy of POCUS for peritonsillar abscess in an emergency department or ear/nose/throat clinic.[99] Although the authors conclude that POCUS demonstrates high sensitivity for ruling in and moderate specificity for ruling out tonsillar abscess, this interpretation is not supported by the reported data. The presented point estimates (sensitivity 74.0%, specificity 79.0%) indicate only moderate diagnostic performance for both rule-in and rule-out purposes, and do not justify the stated conclusion.

##### Gastrointestinal

We identified 33 DTA-SR across gastrointestinal conditions, including appendicitis, biliary disease, small bowel obstruction, and other gastrointestinal pathologies. Overall, ultrasound showed variable but often high diagnostic accuracy, with substantial differences between conditions and consistently low certainty of evidence across most reviews.

For appendicitis, 15 reviews including 4–22 studies each reported sensitivities ranging from 56.0% to 87.1% and specificities from 75.3% to 97.0%. Variation was largely explained by differences in patient groups (particularly in pregnant women, where sensitivity fell markedly, whereas in children sensitivity was substantially higher) and setting (emergency department vs. radiological performed ultrasound with a trend toward higher sensitivity for emergency department-performed ultrasound). Reviews using structured criteria such as a 6–7mm diameter cutoff tended to show higher accuracy, while studies with high risk of bias or unstandardized protocols showed lower performance. Methodological quality was usually higher in reviews performed on the emergency departments. Certainty of evidence was mostly low or very low, driven by heterogeneity or indirectness.

In the biliary system, seven reviews reported sensitivities of 69.0–81.0% and specificities of 79.0–95.6% for acute cholecystitis. Although the diagnostic criteria for acute cholecystitis were rarely reported in the included reviews, his limited reporting was associated with variability in accuracy estimates. Most reviews showed similar accuracy estimates, with only moderate sensitivity but high specificity. This may reflect the fact that early-stage cholecystitis is difficult to detect on imaging, particularly before clear sonographic signs develop. Accuracy for cholelithiasis was higher (sensitivity 89.8–94.0%, specificity 88.0–93.0%), reflecting the relative simplicity of gallstone detection compared to the lower sensitivity (73.0%) for detecting stones after migration into the common bile duct. Certainty of evidence again remained predominantly low.

For small bowel obstruction, five reviews (2–7 studies each) consistently showed high sensitivities (83.0–97.0%) and specificities (77.0–96.0%). Differences across reviews mainly reflected variation in diagnostic thresholds (e.g., bowel dilation ≥25 mm), whether absence of peristalsis was required, and inclusion of pediatric patients. Emergency-department studies with clearly defined criteria showed the best performance. Certainty of evidence ranged from moderate for sensitivity to low for specificity. Across reviews, uncertainty in specificity was associated with heterogeneity in diagnostic thresholds and reference standards, particularly regarding the degree of bowel dilatation required to define obstruction.

In summary, diagnostic accuracy in gastrointestinal ultrasound is particularly high for the detection of gallstones. Ultrasound for acute cholecystitis demonstrates very good specificity, whereas its use in diagnosing small bowel obstruction shows high sensitivity. In contrast, ultrasound for appendicitis provides only poor to moderate accuracy, and its performance appears to be highly dependent on the population and clinical setting. Overall, heterogeneity across gastrointestinal reviews was mainly driven by differences in population characteristics, setting or experience of the ultrasound performer. Certainty of evidence ratings were mostly low or very low because of the heterogeneity of the result of the primary studies. Because many examinations were performed by radiologists, the transferability of the results to other clinical settings was as well often limited. Despite this, ultrasound consistently performed well for small bowel obstruction, cholelithiasis, and diverticulitis, while accuracy for appendicitis and acute cholecystitis varied more substantially depending on technique and study quality.

##### Genitourinary

Seven DTA SR assessed genitourinary conditions, specifically testicular torsion, urolithiasis, bladder outlet obstruction, acute kidney injury, and hydronephrosis. The highest accuracy was seen for testicular torsion (sensitivity 86.4–92.0%, specificity 95.3–99.0%) with varying certainty of evidence mostly due to risk of bias and indirectness.[134, 135] For the other genitourinary conditions, accuracy measures vary from low to high with mostly very low to low certainty of evidence and critically low to moderate methodological quality. Most commonly the certainty of evidence was impacted by heterogeneity. Three out of five reviews were rated as high risk of bias.[136, 138, 139] Overall POCUS shows promising accuracy to rule in testicular torsion, but the certainty of evidence is limited.

##### Gynecological

We identified 11 DTA SRs assessing gynecological and early pregnancy related conditions, specifically ectopic pregnancy, cesarean scar pregnancy, intrauterine pregnancy, retained products of conception, and adnexal or ovarian torsion. Overall, ultrasound demonstrated variable but often clinically useful diagnostic accuracy, although certainty of evidence was predominantly low or very low because of heterogeneity, risk of bias, and differences in reference standards and sonographic criteria.

For ectopic pregnancy, diagnostic performance varied substantially depending on the sonographic feature assessed and the clinical setting. Stein et al. reported very high sensitivity (97.0%) but only moderate specificity (76.0%) for emergency physician-performed pelvic ultrasound in patients at risk for ectopic pregnancy, suggesting ultrasound may be particularly useful for ruling out ectopic pregnancy in emergency settings.[143] In contrast, Crochet et al. demonstrated that individual ultrasound findings such as adnexal mass or free pelvic fluid had only moderate sensitivity (47.0–64.0%) but high specificity (91.0–92.0%), indicating that isolated sonographic findings are more useful for ruling in than excluding tubal ectopic pregnancy.[141] For cesarean scar pregnancy, Han et al. found moderate to high sensitivity (83.0%) and moderate specificity (74.0%) for transvaginal ultrasound.[140] Overall, ultrasound appears useful as a first-line imaging modality in suspected ectopic or cesarean scar pregnancy, though certainty of evidence remained mostly low because of heterogeneity in diagnostic criteria and reference standards.

Four reviews assessed adnexal or ovarian torsion.[147–150] Diagnostic accuracy varied considerably across studies and ultrasound signs. Overall pooled sensitivities ranged from 53.0% to 82.0%, whereas specificities ranged from 76.0% to 95.0. Highly specific findings included absent intraovarian Doppler flow, ovarian edema, and the whirlpool sign, whereas adnexal mass and pelvic fluid showed lower specificity. Reviews restricted to Doppler ultrasound generally demonstrated higher specificity compared to ultrasound alone. Across reviews, ultrasound findings for adnexal torsion were consistently more useful for ruling in than ruling out torsion. However, certainty of evidence was uniformly very low, mainly because of heterogeneity, inconsistent sonographic criteria, and methodological limitations.

Overall, gynecological ultrasound demonstrated the best rule-in performance for adnexal torsion and specific ectopic pregnancy findings when characteristic sonographic signs were present. In emergency department settings, pelvic ultrasound also showed promising sensitivity for excluding ectopic pregnancy. Nevertheless, diagnostic performance varied substantially according to the specific ultrasound sign, patient population, and imaging protocol. Most reviews were limited by heterogeneity, variable operator expertise, and low methodological quality, resulting in predominantly low or very low certainty of evidence.

##### Intervention-related

We identified 17 DTA SR assessing the diagnostic accuracy of POCUS for interventions including endotracheal tube placement, prediction of difficult intubation, central venous catheter placement, and nasogastric tube placement. Five out of seven reviews assessing endotracheal tube placement showed high or very high pooled sensitivity and specificity (88.0-98.7% and 94.0–96.0%, respectively) with an almost always high certainty of evidence.[151–155] Soni et al. and Yang et al. showed lower specificity (75.0-78.0%) with low certainty of evidence.[156, 157] Yang et al. assessed only LUS whereas the other six reviews assessed transtracheal ultrasound. Methodological quality was moderate across all reviews.

The diagnostic accuracy for prediction of difficult intubation varied widely with very low to low certainty of evidence. This variability in sensitivities is probably due to varying ultrasound signs used and due to varying populations, both causing high heterogeneity. Three studies excluded obese patients to a varying degree, whereas Ji et al. did not report exclusion due to obesity.[158–161]

All four DTA SR assessing central venous catheter placement showed very high specificity (98.0–100.0%) with varying sensitivity (68.2–82.0%). Certainty of evidence was low to moderate as well as methodological quality. The observed variability in sensitivity may be attributable to differences in study populations and clinical settings. Two studies included exclusively adult patients, whereas Smit et al. enrolled both adults and children, potentially introducing heterogeneity related to age distribution and disease presentation.[162–165] Operator experience did not significantly influence DTA measures according to Smit et al.’ subgroup analysis.

Both DTA SRs assessing nasogastric tube placement showed high to very high sensitivity and specificity (93.0% and 88.0–97.0%, respectively).[166, 167] Certainty of evidence varied from low to high with heterogeneity and imprecision being the reason for lower certainty. Methodological quality was moderate. Reasons for heterogeneity and imprecision were probably low sample sizes and differences in populations.

##### Musculoskeletal

We identified 21 DTA SR assessing the diagnostic accuracy of ultrasound for crystal arthropathies, vasculitis and miscellaneous musculoskeletal conditions. Filippou et al. and Gamon et al. found ultrasound to have high sensitivity (87.9–89.0%) and very high specificity (91.5–94.0%), respectively, in diagnosing Calcium Pyrophosphate Dehydrate Deposition Disease (CPPD) arthritis, though the certainty of evidence was very low in both reviews.[168, 169]

For gouty arthritis accuracy measures were more varied with low to very high sensitivity 60.0–93.0%) and moderate to very high specificity (73.0–95.3%), though newer reviews trend towards higher specificity. Certainty of evidence was predominantly very low reflecting overall low methodological quality, high heterogeneity, wide confidence intervals, and indirectness. Overall, ultrasound shows promising accuracy for the diagnosis of crystal arthropathies but cannot be recommended to be used as a single diagnostic tool due to low certainty of evidence.

Both sensitivity and specificity of ultrasound for giant cell arteritis or large vessel vasculitis varied (67.0–88.0% and 79.0–96.0%, respectively). When compared to clinical diagnosis or temporal artery biopsy specificity was very high (>90%) compared to temporal artery biopsy alone. Certainty of evidence was again very low. Ultrasound is useful as an additional diagnostic tool but cannot be recommended to use singularly for the diagnosis of giant cell arteritis.

Ultrasound showed moderate to very high accuracy for miscellaneous musculoskeletal conditions. Certainty of evidence was predominantly very low to low.

##### Neurological

We identified 18 DTA SRs assessing neurological conditions, specifically carotid artery stenosis, intracranial artery stenosis, vertebral artery stenosis, intracranial pressure, intracranial hemorrhage, intracranial masses, and neurodegenerative disease.

Liu et al. found very high sensitivity and specificity for contrast enhanced ultrasound (CEUS) to diagnose extracranial carotid artery occlusion.[189] Cassola et al. and Wardlaw et al. found varying accuracy depending on the cut-off values.[188, 190] Certainty of evidence was predominantly very low or low across all three reviews limiting conclusions regarding the role of ultrasound for the diagnosis of carotid artery stenosis/occlusion in an emergency medicine setting.

Three studies (Liu et al., Mattioni et al. and Zhu et al.) found very high diagnostic accuracy for intracranial artery occlusion in adults with acute ischemic stroke (sensitivity 90.0–96.0%, specificity 94.0–95.0%) with comparable populations, index tests, and reference standards.[191–193] Methodological quality was moderate to high, but certainty of evidence was still predominantly very low due to heterogeneity, indirectness, and imprecision.

Khan et al. assessed the diagnostic accuracy of both doppler and color doppler ultrasound for vertebral artery stenosis.[196] Both modalities showed moderate sensitivity (both 70.2%) and very high specificity (93.4–97.7%). However, despite specificity estimates exceeding predefined rule-in thresholds, very low certainty of evidence and low methodological quality precluded classification as a clinically supportive rule-in application.

Pinto-Villalba et al. evaluated transcranial Doppler and color-coded duplex ultrasound for ischemic and hemorrhagic stroke, reporting moderate sensitivity (81.1%) and specificity (85.5%). Methodological quality and certainty of evidence were both low to very low, limiting confidence in the diagnostic estimates.[195]

Seven DTA SRs assessed optic nerve sheath diameter to diagnose elevated intracranial pressure. All found comparable sensitivities (82.0–94.0%) and specificities (82.0–90.7%). Importantly, at least one review demonstrated moderate certainty of evidence with diagnostic estimates exceeding predefined rule-in and rule-out thresholds, indicating potential utility of ultrasound assessment of optic nerve sheath diameter to support both exclusion and confirmation of elevated intracranial pressure in appropriate clinical contexts.[201]

##### Ocular

The two reviews on retinal detachment report very similar diagnostic accuracy, with high sensitivities (92.0–94.0%) and specificities (91.4–94.0%). Both analyses are based on moderate-quality evidence.

For foreign bodies, globe rupture, and lens dislocation, diagnostic performance is uniformly excellent. Because these findings rely on only two studies each, using similar methods and the same reference standard, little heterogeneity is observed. In contrast, vitreous detachment and vitreous hemorrhage show more variable results. Their lower or more uncertain sensitivities reflect the subtler and less consistent sonographic appearances of these conditions.

##### Respiratory

In total, we identified 31 DTA-SR across respiratory conditions, covering pleural disorders, infectious lung diseases, acute respiratory distress syndrome and acute respiratory failure. Overall, lung ultrasound demonstrated high to very high diagnostic accuracy, though with substantial variability across topics and reviews.

For pleural pathologies, sensitivities ranged from 79.4% to 97.4% and specificities from 93.6% to 99.0%. Pneumothorax and hemothorax reviews showed excellent specificity but more variable sensitivity, likely because, in the absence of a standardized protocol, loculated effusions or small pneumothoraces could easily be missed.

In COVID-19, sensitivities were consistently high (86.9-96.0%), while specificities varied widely (62.4-97.9%). Lower specificity was mainly associated with reduced scanning protocols, likely because examining fewer lung regions increases the chance of misclassifying nonspecific artefacts as pathological. Nevertheless, ultrasound seems to have more utility for ruling out COVID-19 than for differentiating SARS-CoV-2 infection from other causes of respiratory illness (given limited specificity in abbreviated protocols). Differences in reference standards (RT-PCR, CT, clinical diagnosis) also played a major role: RT-PCR may miss early cases, while CT detects many non-COVID abnormalities.

For pneumonia, sensitivities ranged from 82.0% to 97.0% with Padrao et al. as an outlier (37.0%) and specificities from 81.0% to 98.0%. Higher accuracy was found in reviews using structured protocols or clear diagnostic criteria (e.g., consolidation, focal B-lines). Differences across reviews largely stemmed from heterogeneous reference standards, particularly the use of chest X-ray, which may lead to underdiagnose of pneumonia and could have lowered apparent ultrasound specificity. Variation in operator training and the inclusion of mixed patient populations also may have contributed to the observed heterogeneity.

Overall, variation in scanning protocols, diagnostic definitions, reference standards, and methodological quality explains the differences across reviews. Despite this, lung ultrasound consistently shows strong diagnostic accuracy across respiratory conditions, especially when standardized protocols and clearly defined criteria are applied.

Diagnostic accuracy was moderate for undifferentiated acute dyspnea (sensitivity 85.6%, specificity 80.8%), limiting utility as a stand-alone diagnostic tool.[232] In contrast, acute respiratory failure demonstrated high to very high sensitivity (89.0–92.0%) and specificity (94.0–98.0%), with at least one review showing moderate certainty of evidence for specificity, suggesting potential support for rule-in applications despite predominantly intensive care populations.[233] For acute respiratory distress syndrome (ARDS), diagnostic accuracy was more variable, with sensitivities ranging from 63.1–84.0% and consistently high specificities (87.0–94.2%). Overall, certainty of evidence remained low to very low, supporting lung ultrasound primarily as an adjunctive diagnostic tool in acute respiratory conditions.
