# Supplementary material for: Diagnostic accuracy of point-of-care ultrasound in adult non-traumatic emergency medicine: an umbrella review
Source: BMC Med. 2026 Jul 25;24:579. doi: 10.1186/s12916-026-05094-2 (PMC13689006; doi:10.1186/s12916-026-05094-2)
Supplement: Supplementary file 2 [file 12916_2026_5094_MOESM2_ESM.docx]

## Table S7. Characteristics and reason for exclusion of the excluded reviews screened in fulltext

| **Citation** | **Reason for Exclusion** |
| --- | --- |
| Ablordeppey EA, Drewry AM, Beyer AB, Theodoro DL, Fowler SA, Fuller BM, et al. Diagnostic Accuracy of Central Venous Catheter Confirmation by Bedside Ultrasound Versus Chest Radiography in Critically Ill Patients: A Systematic Review and Meta-Analysis. Crit Care Med. 2017;45(4):715–24. doi:10.1097/CCM.0000000000002188 | duplicate |
| Abram E, Nordstrom A, Shibata J, Sinert R, Paladino L. Diagnosing urolithiasis or nephrolithiasis via emergency ultrasonography and urinalysis. Academic Emergency Medicine. 2016;23:S252–S252. doi:https://dx.doi.org/10.1111/acem.12974 | conference abstract |
| Abreu BFBB de, Scarmagnan GS, Duarte ML, Silva MO da, Iared W. Synovitis detection in rheumatology: a systematic review and meta-analysis of contrast-enhanced ultrasound and magnetic resonance imaging. Sao Paulo Medical Journal = Revista Paulista de Medicina. 2026;144(2):e20253088. doi:10.1590/1516-3180.2025.3088.11112025 | setting |
| Adhikari S, Amini R, Farrell IJ. Bedside ultrasound in the diagnosis of subcutaneous abscess requiring incision drainage. Academic Emergency Medicine. 2013;20(5):S213–S213. doi:https://dx.doi.org/10.1111/acem.12115 | conference abstract |
| Adhikari S, Farrell I, Stolz U. How accurate is ultrasonography in confirming endotracheal tube placement? A meta-analysis. Annals of Emergency Medicine. 2012;60(4):S18–S18. doi:https://dx.doi.org/10.1016/j.annemergmed.2012.06.073 | conference abstract |
| Adhikari S, Farrell I. How accurate is ultrasound in diagnosing pneumonia? A meta-analysis. Academic Emergency Medicine. 2014;21(5):S325–S325. doi:https://dx.doi.org/10.1111/acem.12365 | conference abstract |
| Adhikari SR, Farrell I, Keim S. How accurate is ultrasound in diagnosing pneumoperitoneum? A meta-analysis. Academic Emergency Medicine. 2016;23:S221–2. doi:https://dx.doi.org/10.1111/acem.12974 | conference abstract |
| Al Deeb M, Barbic D, Barbic S, Featherstone R, Dankoff J. Bedside lung ultrasound for the diagnosis of pulmonary EDEMA in patients presenting with acute dyspnea: A systematic review and meta-analysis. Academic Emergency Medicine. 2013;20(5):S16–S16. doi:https://dx.doi.org/10.1111/acem.12115 | conference abstract |
| Al Deeb M, Barbic S, Featherstone R, Dankoff J, Barbic D. Point-of-care ultrasonography for the diagnosis of acute cardiogenic pulmonary edema in patients presenting with acute dyspnea: A systematic review and meta-analysis. Acad Emerg Med. 2014;21(8):843–52. doi:10.1111/acem.12435 | duplicate |
| Al Lawati K, Aljazeeri J, Bates SM, Chan WS, De Wit K. Ability of a single negative ultrasound to rule out deep vein thrombosis in pregnant women: A systematic review and meta-analysis. Journal of Thrombosis & Haemostasis. 2020;18(2):373–80. | outcome measures |
| Al Nufaiei ZF, Alshamrani KM. Comparing Ultrasound, Chest X-Ray, and CT Scan for Pneumonia Detection. Med Devices (Auckl). 2025;18:149–59. Located at: 40061910. doi:10.2147/MDER.S501714 | outcome measures |
| Alanazi MM, Alkhamash JM, Alalawi AH. Ultrasound in the diagnosis and therapeutic management in undifferentiated shock patients in the emergency department. Medical Science. 2024. doi:10.54905/disssi.v28i153.e143ms3470 | study design |
| Alenazi A, Alshibani A. Confirmatory methods for endotracheal tube placement in out-of-hospital settings: A systematic review of the literature. Heliyon. 2024 Apr;10(7):e28479. doi:10.1016/j.heliyon.2024.e28479 | study design |
| Alexander C. Ultrasound for the diagnosis of testicular torsion: a systematic review and meta-analysis of diagnostic accuracy. Journal of Clinical Urology. 2023 Jun;16(1_suppl):4–84. doi:10.1177/20514158231172341 | conference abstract |
| Al-Khafaji MQ, Al-Smadi MW, Al-Khafaji MQ, Aslan S, Al-Khafaji YQ, Bagossy-Blas P, et al. Evaluating Imaging Techniques for Diagnosing and Drainage Guidance of Psoas Muscle Abscess: A Systematic Review. Journal of Clinical Medicine. 2024;13(11):29. | study design |
| Almandoz IB, García JV, Moreno ALP, De Canales Diez JG, Cruz EC, Alcázar Zambrano JL. 2022-RA-1671-ESGO Diagnostic accuracy of different ultrasound signs for detecting adnexal torsion: systematic review andmeta-analysis. In: Diagnostics [Internet]. BMJ Publishing Group Ltd; 2022 [cited 2025 Feb 26]. p. A84.2-A84. Available from: https://ijgc.bmj.com/lookup/doi/10.1136/ijgc-2022-ESGO.186 doi:10.1136/ijgc-2022-ESGO.186 | conference abstract |
| Alomari O, Alrabadi B, Hussein TA, Shtayat S, Hussein RA, Alnahdi R, et al. Ultrasound assessment of peripheral nerve size in Guillain-Barre syndrome: A systematic review and Meta-Analysis. Neuroradiology [Internet]. 2025. Available from: ["https://ovidsp.ovid.com/ovidweb.cgi?T=JS&CSC=Y&NEWS=N&PAGE=fulltext&D=emed28&DO=10.1007%2fs00234-025-03728-5", "https://libkey.io/libraries/528/openurl?genre=article&aulast=Alomari&issn=0028-3940&title=Neuroradiology&atitle=Ultrasound+assessment+of+peripheral+nerve+size+in+Guillain-Barre+syndrome%3A+A+systematic+review+and+Meta-Analysis&volume=&issue=&spage=&epage=&date=2025&doi=10.1007%2Fs00234-025-03728-5&pmid=40782205&sid=OVID:embase"] | outcome measures |
| Al-Sagban A, Algodi M, Saab O, Al-Obaidi H, Graham H, Abuelazm MT, et al. Lung ultrasound-guided decongestion in heart failure patients: A systematic review and meta-analysis of randomized controlled trials. Journal of Critical Care. 2026;93:155435. doi:10.1016/j.jcrc.2026.155435 | index test |
| Alsup C, Davis J, Bates A, Au A, Adhikari S, Farrell I, et al. Accuracy of point-of-care ultrasonography for diagnosing acute appendicitis: A systematic review and meta-analysi. Annals of Emergency Medicine. 2016;68(4):S138–9. | conference abstract |
| Alzahrani SA, Al-Salamah MA, Al-Madani WH, Elbarbary MA. Systematic review and meta-analysis for the use of ultrasound versus radiology in diagnosing of pneumonia. Crit Ultrasound J. 2017;9(1). doi:10.1186/s13089-017-0059-y | duplicate |
| Amini R, Kostura M, Adhikari S, Farrell IJ. Bedside ultrasonography in the diagnosis of symptomatic nephrolithiasis. Academic Emergency Medicine. 2013;20(5):S259–60. doi:https://dx.doi.org/10.1111/acem.12115 | conference abstract |
| Aminlari A, Stone J, McKee R, Subramony R, Nadolski A, Tolia V, et al. Diagnosing Achilles Tendon Rupture with Ultrasound in Patients Treated Surgically: A Systematic Review and Meta-Analysis. The Journal of Emergency Medicine. 2021 Nov;61(5):558–67. doi:10.1016/j.jemermed.2021.09.008 | study design |
| Aminlari A. Ultrasound for diagnosing achilles tendon rupture in patients treated surgically: A systematic review and meta-analysis. Ultrasound in Medicine and Biology. 2015;41(4):S156–S156. | conference abstract |
| Andeweg CS, Wegdam JA, Groenewoud J, van der Wilt GJ, van Goor H, Bleichrodt RP. Toward an evidence-based step-up approach in diagnosing diverticulitis. Scandinavian Journal of Gastroenterology. 2014 Jul 1;49(7):775–84. doi:10.3109/00365521.2014.908475 PubMed PMID: 24874087. | reference standard |
| Anjum ZI, Bacha R, Manzoor I, Gilani SA. Reliability of knee joint sonography in the evaluation of gouty arthritis. J Ultrason. 2021 Nov 29;21(87):300–5. doi:10.15557/JoU.2021.0051 | study design |
| Arida A, Kyprianou M, Kanakis M, Sfikakis PP. The diagnostic value of ultrasonography-derived edema of the temporal artery wall in giant cell arteritis: a second meta-analysis. BMC Musculoskelet Disord. 2010 Dec;11(1):44. doi:10.1186/1471-2474-11-44 | study design |
| Arruzza E, Milanese S, Li LSK, Dizon J. The diagnostic accuracy of computed tomography and ultrasound for acute appendicitis. J of Medical Radiation Sci. 2023 Apr;70(S1):78. doi:10.1002/jmrs.664 | conference abstract |
| Arvig MD, Laursen CB, Jacobsen N, Gæde PH, Lassen AT. Effectiveness of serial focused ultrasound of the lungs and inferior vena cava for monitoring patients with acute dyspnea: a systematic review protocol. JBI Database of Systematic Reviews and Implementation Reports. 2019 Nov;17(11):2317–25. doi:10.11124/JBISRIR-D-19-00027 | study design |
| Asferg C, Usinger L, Kristensen TS, Abdulla J. Accuracy of multi-slice computed tomography for measurement of left ventricular ejection fraction compared with cardiac magnetic resonance imaging and two-dimensional transthoracic echocardiography: A systematic review and meta-analysis. European Journal of Radiology. 2012;81(5):e757–62. | outcome measures |
| Bäcker HC, Wu CH, Strauch RJ. Systematic Review of Diagnosis of Clinically Suspected Scaphoid Fractures. Jnl Wrist Surg. 2020 Feb;09(01):081–9. doi:10.1055/s-0039-1693147 | study design |
| Bakola E, Palaiodimou L, Eleftheriou A, Foska K, Pikouli A, Stefanatou M, et al. Transorbital sonography in idiopathic intracranial hypertension: Single‐center study, systematic review and meta‐analysis. Journal of Neuroimaging. 2024 Jan;34(1):108–19. doi:10.1111/jon.13160 | reference standard |
| Bankole NDA, Dujardin PA, Bala F, Ehrmann S, Desplobain K, Patat F, et al. Transcranial doppler ultrasound for the diagnosis of large vessel occlusion in patients with acute ischemic stroke: A systematic review. Clin Neurol Neurosurg. 2024;245:108506. Located at: 39167933. doi:10.1016/j.clineuro.2024.108506 | study design |
| Barbic D, Chenkin J, Cho D. Correction: In patients presenting to the emergency department with skin and soft tissue infections what is the diagnostic accuracy of point-of-care ultrasonography for the diagnosis of abscess compared to the current standard of care? A systematic review and meta-analysis. BMJ Open. 2017 Sep 14;7(9):e013688corr1. doi:10.1136/bmjopen-2016-013688corr1 PubMed PMID: 28912196; PubMed Central PMCID: PMC5640141. | study design |
| Barbic D, Chenkin J, Cho DD, Jelic T, Scheuermeyer FX. Correction: In patients presenting to the emergency department with skin and soft tissue infections what is the diagnostic accuracy of point-of-care ultrasonography for the diagnosis of abscess compared to the current standard of care? A systematic review and meta-analysis. BMJ Open. 2017 Jan;7(1):e013688. doi:10.1136/bmjopen-2016-013688 | duplicate |
| Barbic D, Chenkin J, Cho DD, Jelic T, Scheuermeyer FX. Correction: In patients presenting to the emergency department with skin and soft tissue infections what is the diagnostic accuracy of point-of-care ultrasonography for the diagnosis of abscess compared to the current standard of care? A systematic review and meta-analysis. BMJ Open. 2017 Jan;7(1):e013688. doi:10.1136/bmjopen-2016-013688 | population |
| Barbic D, Chenkin J, Cho DD, Jelic T, Scheuermeyer FX. In patients presenting to the emergency department with skin and soft tissue infections what is the diagnostic accuracy of point-of-care ultrasonography for the diagnosis of abscess compared to the current standard of care? A systematic review and meta-an. BMJ Open. 2017;7(1):e013688. doi:10.1136/bmjopen-2016-013688 | duplicate |
| Barra L, Kanji T, Malette J, Pagnoux C. Imaging modalities for the diagnosis and disease activity assessment of Takayasu’s arteritis: A systematic review and meta-analysis. Autoimmunity Reviews. 2018 Feb;17(2):175–87. doi:10.1016/j.autrev.2017.11.021 | population |
| Basmaji J, Tang JE, Arntfield R, Desai K, Ball IM, Fiorini K, et al. The diagnostic accuracy of point-of-care ultrasound in shock: a systematic review and meta-analysis. Can J Anesth/J Can Anesth. 2025;72(7):1118–29. doi:10.1007/s12630-025-02997-1 | duplicate |
| Beier L, Davis J, Esener D, Grant C, Fields JM. Carotid Ultrasound to Predict Fluid Responsiveness: A Systematic Review. Journal of Ultrasound in Medicine. 2020;39(10):1965–76. | outcome measures |
| Benabbas R, Zehtabchi S, Wakai A, Allen R, deSouza IS, Richards RJ, et al. Cardiac Biomarkers, Echocardiography, and Outpatient Cardiac Monitoring for Evaluation of Emergency Department Patients With Syncope: A Systematic Review and Analysis of Direct Evidence for SAEM GRACE. Academic Emergency Medicine. 2026;33(2):e70175. doi:10.1111/acem.70175 | outcome measures |
| Berg I, Walpot K, Lamprecht H, Valois M, Lanctôt JF, Srour N, et al. A Systemic Review on the Diagnostic Accuracy of Point-of-Care Ultrasound in Patients With Undifferentiated Shock in the Emergency Department. Cureus. 2022. doi:10.7759/cureus.23188 | duplicate |
| Berg I, Walpot K, Lamprecht H, Valois M, Lanctot JF, Srour N, et al. A Systemic Review on the Diagnostic Accuracy of Point-of-Care Ultrasound in Patients With Undifferentiated Shock in the Emergency Department. Cureus. 2022;14(3):e23188. Located at: 35444920. doi:10.7759/cureus.23188 | outcome measures |
| Berikashvili LB, Kuznetsov IV, Yadgarov MY, Ryzhkov PV, Polyakov PA, Yavorovskiy AG, et al. Assessment of Fluid Responsiveness via Central Venous Ultrasound Measurement: A Network Meta-Analysis. Journal of Clinical Medicine. 2025;14(2):14. | study design |
| Bigio J, Kohli M, Klinton JS, MacLean E, Gore G, Small PM, et al. Diagnostic accuracy of point-of-care ultrasound for pulmonary tuberculosis: A systematic review. Quinn F, editor. PLoS ONE. 2021 May 7;16(5):e0251236. doi:10.1371/journal.pone.0251236 | study design |
| Blyth L, Atkinson P, Gadd K, Lang E. Bedside focused echocardiography as predictor of survival in cardiac arrest patients: A systematic review. Acad Emerg Med. 2012;19(10):1119–26. doi:10.1111/j.1553-2712.2012.01456.x | duplicate |
| Blyth LR, Atkinson P, Gadd K, Lang E. Does cardiac standstill mean we can stop CPR? Focused ultrasound as predictor of survival in cardiac arrest patients: A systematic review. Annals of Emergency Medicine. 2011;58(4):S265–S265. doi:https://dx.doi.org/10.1016/j.annemergmed.2011.06.292 | conference abstract |
| Blyth U, Fourie H, Akinola L, Smith C, Melo P, Granne I. Definition and diagnostic criteria of retained products of conception following first-trimester pregnancy loss: a systematic review. Human Fertility. 2025 Dec 31;28(1):2522054. doi:10.1080/14647273.2025.2522054 PubMed PMID: 40625283. | study design |
| Bobdiwala S, Saso S, Verbakel JY, Al-Memar M, Van Calster B, Timmerman D, et al. Diagnostic protocols for the management of pregnancy of unknown location: a systematic review and meta-analysis. BJOG. 2019 Jan;126(2):190–8. doi:10.1111/1471-0528.15442 PubMed PMID: 30129999. | index test |
| Bosch P, Bond M, Dejaco C, Ponte C, Mackie S, Falzon L, et al. OP0188 IMAGING IN DIAGNOSIS, MONITORING AND OUTCOME PREDICTION OF LARGE VESSEL VASCULITIS: A SYSTEMATIC LITERATURE REVIEW INFORMING THE 2023 UPDATE OF THE EULAR RECOMMENDATIONS. Annals of the Rheumatic Diseases. 2023 Jun 1;82:124–5. doi:10.1136/annrheumdis-2023-eular.1046 | conference abstract |
| Botis I, Efstathiadou A, Papanastasiou CA, Kokkinidis DG, Zegkos T, Efthimiadis G, et al. Evaluation of mitral regurgitation by cardiac magnetic resonance and transthoracic echocardiography: a systematic review and meta-analysis. Rev Cardiovasc Med. 2021 Dec 22;22(4):1513. doi:10.31083/j.rcm2204155 | study design |
| Bou Chebl R, Kiblawi S, El Khuri C, El Hajj N, Bachir R, Aoun R, et al. Use of Contrast‐Enhanced Ultrasound for Confirmation of Central Venous Catheter Placement: Systematic Review and Meta‐analysis. J of Ultrasound Medicine. 2017 Dec;36(12):2503–10. Located at: 227. doi:10.1002/jum.14296 | conference abstract |
| Bouchard MD, Pow C, Gilbert J, Slawaska-Eng D, Vivekanantha P, Fageeh R, et al. Characteristics, Patterns and Optimal Treatment Strategies of Morel-Lavallee Lesions: A Systematic Review. Journal of Orthopaedic Trauma. 2025;6:06. | outcome measures |
| Boumans MMA, Aerts W, Pisani L, Bos LDJ, Smit M, Tuinman P. Diagnostic accuracy of lung ultrasound in diagnosis of ARDS and identification of focal or non-focal ARDS subphenotypes: a systematic review and meta-analysis. Critical Care. 2024. doi:10.1186/s13054-024-04985-1 | duplicate |
| Brockhattingen K, Drejer Thrane M, Gade GV, Smedemark SA. Point-of-care ultrasound and geriatric patients:  a systematic review. Eur Geriatr Med. 2022 Dec 5;13(S1):S167-168. doi:10.1007/s41999-022-00711-8 | conference abstract |
| Burlet KJDE, Ing AJ, Larsen PD, Dennett ER. Systematic review of diagnostic pathways for patients presenting with acute abdominal pain. International Journal for Quality in Health Care. 2018;30(9):678–83. | study design |
| Burnside PR, Brown MD, Kline JA. Systematic review of emergency physician-performed ultrasonography for lower-extremity deep vein thrombosis. Acad Emerg Med. 2008;15(6):493–8. doi:10.1111/j.1553-2712.2008.00101.x | duplicate |
| Burwash-Brennan T, Lalande E, Burns K, Atkinson P, Lambert M, Jarman B, et al. Is point-of-care ultrasound a reliable predictor of outcome during atraumatic, nonshockable cardiac arrest?. In: Academic Emergency Medicine [Internet]. 2019 [cited 2025 Apr 20]. Available from: https://onlinelibrary.wiley.com/doi/10.1111/acem.13756 doi:10.1111/acem.13756 | conference abstract |
| Buttgereit F, Dejaco C, Matteson EL, Dasgupta B. Polymyalgia rheumatica and giant cell arteritis a systematic review. JAMA. 2016;315(22):2442–58. doi:10.1001/jama.2016.5444 | study design |
| Caballero AF, Villarreal K. Ultrasound for central vascular access. A safety concept that is renewed day by day: Review. Colombian Journal of Anesthesiology. 2018;46:32–8. | study design |
| Campbell RE, McGhee AN, Freedman KB, Tjoumakaris FP. Diagnostic Imaging of Ulnar Collateral Ligament Injury: A Systematic Review. Am J Sports Med. 2020 Sep;48(11):2819–27. doi:10.1177/0363546520937302 | study design |
| Cao J, Sun J, Wang Y, Wang L. Diagnostic accuracy of cardiopulmonary ultrasound for pulmonary embolism: A systematic review and meta‐analysis. Echocardiography. 2022;39(2):185–93. doi:10.1111/echo.15280 | duplicate |
| Carroll PJ, Gibson D, El-Faedy O, Dunne C, Coffey C, Hannigan A, et al. Surgeon-performed ultrasound at the bedside for the detection of appendicitis and gallstones: Systematic review and meta-analysis. Am J Surg. 2013;205(1):102–8. doi:10.1016/j.amjsurg.2012.02.017 | duplicate |
| Carroll PJ, Gibson D, El-Faedy O, Dunne C, Coffey C, Hannigan A, et al. Surgeon-performed ultrasound at the bedside for the detection of appendicitis and gallstones: systematic review and meta-analysis. The American Journal of Surgery. 2013 Jan;205(1):102–8. doi:10.1016/j.amjsurg.2012.02.017 | setting |
| Carsetti A, Sorbello M, Adrario E, Donati A, Falcetta S. Airway Ultrasound as Predictor of Difficult Direct Laryngoscopy: A Systematic Review and Meta-analysis. Anesth Analg. 2022;134(4):740–50. doi:10.1213/ANE.0000000000005839 | duplicate |
| Caruso M, Dell’Aversano Orabona G, Di Serafino M, Iacobellis F, Verde F, Grimaldi D, et al. Role of Ultrasound in the Assessment and Differential Diagnosis of Pelvic Pain in Pregnancy. Diagnostics [Internet]. 2022;12(3). Available from: ["https://ovidsp.ovid.com/ovidweb.cgi?T=JS&CSC=Y&NEWS=N&PAGE=fulltext&D=emed23&DO=10.3390%2fdiagnostics12030640", "https://libkey.io/libraries/528/openurl?genre=article&aulast=Caruso&issn=2075-4418&title=Diagnostics&atitle=Role+of+Ultrasound+in+the+Assessment+and+Differential+Diagnosis+of+Pelvic+Pain+in+Pregnancy&volume=12&issue=3&spage=640&epage=&date=2022&doi=10.3390%2Fdiagnostics12030640&pmid=&sid=OVID:embase"] | study design |
| Castro AA, Lima FJC de, Sousa-Rodrigues CF de, Barbosa FT. Accuracy of ultrasound to detect thrombosis in pregnancy: A systematic review. Rev Assoc Med Bras. 2017;63:278–83. doi:https://doi.org/10.1590/1806-9282.63.03.278 | study design |
| Chaijareenont C, Krutsri C, Sumpritpradit P, Singhatas P, Thampongsa T, Lertsithichai P, et al. FAST accuracy in major pelvic fractures for decision-making of abdominal exploration: Systematic review and meta-analysis. Annals of Medicine and Surgery. 2020 Dec;60:175–81. Located at: 104. doi:10.1016/j.amsu.2020.10.018 | outcome measures |
| Chappell FM, Wardlaw JM, Brazzelli M, Best JJK. Doppler ultrasound, CT angiography, MR angiography, and contrast-enhanced MR angiography versus intra-arterial angiography for moderate and severe carotid stenosis in symptomatic patients. Cochrane Database of Systematic Reviews [Internet]. 2008. Available from: ["https://ovidsp.ovid.com/ovidweb.cgi?T=JS&CSC=Y&NEWS=N&PAGE=fulltext&D=emed10&DO=10.1002%2f14651858.CD007423", "https://libkey.io/libraries/528/openurl?genre=article&aulast=Chappell&issn=1469-493X&title=Cochrane+Database+of+Systematic+Reviews&atitle=Doppler+ultrasound%2C+CT+angiography%2C+MR+angiography%2C+and+contrast-enhanced+MR+angiography+versus+intra-arterial+angiography+for+moderate+and+severe+carotid+stenosis+in+symptomatic+patients&volume=&issue=4&spage=CD007423&epage=&date=2008&doi=10.1002%2F14651858.CD007423&pmid=&sid=OVID:embase"] | study design |
| Chau JP, Thompson DR, Fernandez R, Griffiths R, Lo HS. Methods for determining the correct nasogastric tube placement after insertion: a meta-analysis. JBI Library of Systematic Reviewis. 2009;7(16):679–760. | outcome measures |
| Chaudhary R, Sukhi A, Simon MA, Villanueva FS, Pacella JJ. Role of Internal Jugular Venous Ultrasound in suspected or confirmed Heart Failure: A Systematic Review. Journal of Cardiac Failure. 2022;28(4):639–49. | study design |
| Chavez MA, Shams N, Ellington LE, Naithani N, Gilman RH, Steinhoff MC, et al. Lung ultrasound for the diagnosis of pneumonia in adults: A systematic review and meta-analysis. Respir Res. 2014;15(1). doi:10.1186/1465-9921-15-50 | duplicate |
| Chen H, Chen Y, Liu S, Liu G, Liu H, Song B. Lung ultrasound in diagnosis of acute heart failure: A protocol for systematic review and meta-analysis. Medicine. 2022 Dec 9;101(49):e32257. doi:10.1097/MD.0000000000032257 | study design |
| Childs DD, Lalwani N, Craven T, Arif H, Morgan M, Anderson M, et al. A meta-analysis of the performance of ultrasound, hepatobiliary scintigraphy, CT and MRI in the diagnosis of acute cholecystitis. Abdom Radiol. 2024;49(2):384–98. Located at: 37982832. doi:10.1007/s00261-023-04059-w | duplicate |
| Chin B, Ramji M, Farrokhyar F, Bain JR. Efficient Imaging: Examining the Value of Ultrasound in the Diagnosis of Traumatic Adult Brachial Plexus Injuries, A Systematic Review. NEUROSURGERY. 2018 Sep;83(3):323–32. doi:10.1093/neuros/nyx483 | setting |
| Cho SU, Oh SK. Accuracy of ultrasound for the diagnosis of acute appendicitis in the emergency department: A systematic review. Medicine. 2023;102(13):e33397. doi:10.1097/md.0000000000033397 | duplicate |
| Chou E. The Outcome Predictive Power of Focused Echocardiography in Cardiopulmonary Resuscitation: A Meta-Analysis: 88. Academic emergency medicine. 2016;23:S47. | conference abstract |
| Chowalloor PV, Keen HI. A systematic review of ultrasonography in gout and asymptomatic hyperuricaemia. Annals of the Rheumatic Diseases. 2013 May;72(5):638–45. doi:10.1136/annrheumdis-2012-202301 | study design |
| Christopher N, Mehta N, Sinert R. Systematic review: Lung ultrasound for the diagnosis of pneumonia in the emergency department. Academic Emergency Medicine. 2014;21(5):S36–S36. doi:https://dx.doi.org/10.1111/acem.12365 | conference abstract |
| Ciozda W, Kedan I, Kehl DW, Zimmer R, Khandwalla R, Kimchi A. The efficacy of sonographic measurement of inferior vena cava diameter as an estimate of central venous pressure. Cardiovascular Ultrasound [Internet]. 2016;14(1). Available from: ["https://ovidsp.ovid.com/ovidweb.cgi?T=JS&CSC=Y&NEWS=N&PAGE=fulltext&D=emed17&DO=10.1186%2fs12947-016-0076-1", "https://libkey.io/libraries/528/openurl?genre=article&aulast=Ciozda&issn=1476-7120&title=Cardiovascular+Ultrasound&atitle=The+efficacy+of+sonographic+measurement+of+inferior+vena+cava+diameter+as+an+estimate+of+central+venous+pressure&volume=14&issue=1&spage=76&epage=&date=2016&doi=10.1186%2Fs12947-016-0076-1&pmid=27542597&sid=OVID:embase"] | study design |
| Cochon L, Esin J, Baez AA. Bayesian comparative model of CT scan and ultrasonography in the assessment of acute appendicitis: results from the Acute Care Diagnostic Collaboration project. The American Journal of Emergency Medicine. 2016 Nov;34(11):2070–3. doi:10.1016/j.ajem.2016.07.012 | study design |
| Cochon L, Gutfreund Y, Baez AA. Acute care diagnostic collaboration: Bayesian comparative assessment of diagnostic accuracy of CT scan and ultrasonography in the diagnosis of urolithiasis after the application of the STONE score. Annals of Emergency Medicine. 2016;68(4):S140–1. | conference abstract |
| Cochon L, Pena M, Baez A. Comparative assessment of diagnostic accuracy of computed tomography and ultrasonography in the diagnosis of acute appendicitis after the application of the alvarado scoring system. Annals of Emergency Medicine. 2013;62(4):S35–S35. doi:https://dx.doi.org/10.1016/j.annemergmed.2013.07.375 | conference abstract |
| Colò G, Bignotti B, Costa G, Signori A, Tagliafico A. Ultrasound or MRI in the Evaluation of Anterior Talofibular Ligament (ATFL) Injuries: Systematic Review and Meta-Analysis. Diagnostics. 2023 Jul 10;13(14):2324. doi:10.3390/diagnostics13142324 | reference standard |
| Corcoran JP, Wallbridge PD, Rahman NM, Mallett S, Hew M. Looking beyond the pleura-a systematic review of thoracic ultrasonography to diagnose lung consolidation in respiratory failure. Thorax. 2014;69:A23–4. doi:https://dx.doi.org/10.1136/thoraxjnl-2014-206260.47 | conference abstract |
| da Hora Passos R, da Cunha Lyrio RM, Lourenco ID, de Almeida Figueiredo EJ, Flato UAP, Barbas CV, et al. LUS me up: elevating ARDS diagnosis. Critical Care. 2024;28(1). Located at: 2030759589. doi:10.1186/s13054-024-05044-5 | study design |
| Dahm P, Koziarz A, Gerardo CJ, Benipal S, Nishijima DK, Jung JH, et al. MP54-01 A SYSTEMATIC REVIEW OF CLINICAL SIGNS AND SYMPTOMS, PREDICTION RULES AND IMAGING MODALITIES IN PATIENTS WITH SUSPECTED RENAL COLIC. Journal of Urology. 2021 Sep;206(Supplement 3). doi:10.1097/JU.0000000000002084.01 | conference abstract |
| Dahmarde H, Parooie F, Salarzaei M. Accuracy of Ultrasound in Diagnosis of Pneumothorax: A Comparison between Neonates and Adults—A Systematic Review and Meta-Analysis. Canadian Respiratory Journal. 2019 Dec 19;2019:1–16. doi:10.1155/2019/5271982 | population |
| Dams OC, Reininga IHF, Gielen JL, Van Den Akker-Scheek I, Zwerver J. Imaging modalities in the diagnosis and monitoring of Achilles tendon ruptures: A systematic review. Injury. 2017 Nov;48(11):2383–99. doi:10.1016/j.injury.2017.09.013 | study design |
| Das SK, Choupoo NS, Haldar R, Lahkar A. Transtracheal ultrasound for verification of endotracheal tube placement: a systematic review and meta-analysis. Can J Anesth. 2015;62(4):413–23. doi:10.1007/s12630-014-0301-z | duplicate |
| Davis JJ, Baier L, Fields JM, Esener D. 345 Carotid Ultrasound for Fluid Responsiveness: A Systematic Review. Annals of Emergency Medicine. 2019 Oct;74(4):S135–6. doi:10.1016/j.annemergmed.2019.08.304 | conference abstract |
| Davis JJ, Garson L, Vetter I, Tajani A, Au A, Riesenberg LA, et al. Echocardiography for the diagnosis of pulmonary embolism: A meta-analysis. Annals of Emergency Medicine. 2015;66(4):S127–8. | conference abstract |
| De Jesus JO, Parker L, Frangos AJ, Nazarian LN. Accuracy of MRI, MR Arthrography, and Ultrasound in the Diagnosis of Rotator Cuff Tears: A Meta-Analysis. American Journal of Roentgenology. 2009 Jun;192(6):1701–7. doi:10.2214/AJR.08.1241 | study design |
| Detsky ME, Jivraj N, Adhikari NK, Friedrich JO, Pinto R, Simel DL, et al. Will this patient be difficult to intubate? the rational clinical examination systematic review. JAMA. 2019;321(5):493–503. doi:10.1001/jama.2018.21413 | index test |
| Dhar S, Kapoor S, Mahajan S, Kumar P, Bansal R, Aggarwal R. Clinical applications of the RUSH protocol in anesthetic, critical care, and orthopedic emergency settings: a systematic review. International Journal of Life Sciences Biotechnology and Pharma Research. 2025;14(9):826–35. Located at: 2040461684. doi:10.69605/ijlbpr_14.9.2025.135 | study design |
| Di Gennaro F, Pisani L, Veronese N, Pizzol D, Lippolis V, Saracino A, et al. Potential Diagnostic Properties of Chest Ultrasound in Thoracic Tuberculosis-A Systematic Review. International Journal of Environmental Research & Public Health [Electronic Resource]. 2018;15(10):12. | study design |
| Dones VC, Grimmer K, Thoirs K, Suarez CG, Luker J. The diagnostic validity of musculoskeletal ultrasound in lateral epicondylalgia: a systematic review. BMC Med Imaging. 2014 Dec;14(1):10. doi:10.1186/1471-2342-14-10 | reference standard |
| Duanmu Y, Goldsmith AJ, Henwood PC, Platz E, Hoyler JE, Kimberly HH. Visual estimation of tricuspid annular plane systolic excursion by emergency medicine clinicians. Western Journal of Emergency Medicine. 1022;21(4):1022–8. Located at: 2007112478. doi:10.5811/westjem.2020.5.46714 | study design |
| Dubourg J, Javouhey E, Geeraerts T, Messerer M, Kassai B. Ultrasonography of optic nerve sheath diameter for detection of raised intracranial pressure: A systematic review and meta-analysis. Intensive Care Med. 2011;37(7):1059–68. doi:10.1007/s00134-011-2224-2 | population |
| Dudek M, Szarpak L, Peacock FW, Gasecka A, Michalski T, Wroblewski P, et al. Diagnostic performance of point-of-use ultrasound of resuscitation outcomes: A systematic review and meta-analysis of 3265 patients. Cardiol J. 2023;30(2):237–46. doi:10.5603/CJ.a2021.0044 | duplicate |
| Duftner C, Dejaco C, Sepriano A, Falzon L, Schmidt WA, Ramiro S. Imaging for diagnosis, monitoring and outcome prediction of large vessel vasculitis: A systematic review of the literature and meta-analysis informing the EULAR recommendations. Arthritis and Rheumatology. 2017;American College of Rheumatology/Association of Rheumatology Health Professionals Annual Scientific Meeting, ACR/ARHP 2017. San Diego, CA United States.69. | duplicate |
| Duftner C, Dejaco C, Sepriano A, Falzon L, Schmidt WA, Ramiro S. Imaging in diagnosis, outcome prediction and monitoring of large vessel vasculitis: a systematic literature review and meta-analysis informing the EULAR recommendations. RMD Open. 2018;4(1):e000612. | duplicate |
| Ebell MH, McKay B, Guilbault R, Ermias Y. Diagnosis of acute rhinosinusitis in primary care: a systematic review of test accuracy. Br J Gen Pract. 2016 Sep;66(650):e612–32. Located at: 43. doi:10.3399/bjgp16X686581 | study design |
| Ebell MH. Diagnosing Pulmonary Embolism. | study design |
| Ebrahimi A, Yousefifard M, Kazemi HM, Rasouli HR, Asady H, Jafari AM, et al. Diagnostic accuracy of chest ultrasonography versus chest radiography for identification of pneumothorax: A systematic review and meta-analysis. Tanaffos. 2014;13(4):29–40. | duplicate |
| Elfaituri MK, Khaled A, BenGhatnsh A, Elfaituri A, Faraj HAA, Msherghi A. Abstract 275: Point-of-Use Ultrasound in Out-of-Hospital Cardiac Arrest: A Meta-Analysis of Diagnostic Accuracy and Outcome Prediction. Circulation. 2023 Nov 7;148(Suppl_1). doi:10.1161/circ.148.suppl_1.275 | conference abstract |
| El-Sheikh Y, Wong I, Farrokhyar F, Thoma A. Diagnosis of finger flexor pulley injury in rock climbers: A systematic review. Canadian Journal of Plastic Surgery. 2006;14(4):227–31. | study design |
| Entezari P, Jalili M, Seyedhosseini J, Doosti-Irani A, Mirfazaelian H. Accuracy of Ultrasonography in Diagnosis of Shoulder Dislocation: A Systematic Review. Advanced Journal of Emergency Medicine. 2019 Dec 1;4(1):e9. doi:10.22114/ajem.v0i0.285 | study design |
| Falsetti L, Zaccone V, Marra A, Tarquinio N, Viticchi G, Sampaolesi M, et al. Clinical Method Applied to Focused Ultrasound: The Case of Wells’ Score and Echocardiography in the Emergency Department: A Systematic Review and a Meta-Analysis. Medicina. 2021 Jul 28;57(8):766. doi:10.3390/medicina57080766 | outcome measures |
| Fernandez RS, Chau JPC, Thompson DR, Griffiths R, Lo HS. Accuracy of biochemical markers for predicting nasogastric tube placement in adults-A systematic review of diagnostic studies. Int J Nurs Stud. 2010;47(8):1037–46. doi:10.1016/j.ijnurstu.2010.03.015 | index test |
| Fernando S, Tran A, Cheng W, Rochwerg B, Taljaard M, Kyeremanteng K, et al. MP07: Diagnosis of elevated intracranial pressure in critically ill adults – a systematic review and meta-analysis. CJEM. 2019 May;21(S1):S44. doi:10.1017/cem.2019.142 | conference abstract |
| Fernando SM, Tran A, Cheng W, Rochwerg B, Kyeremanteng K, Seely AJE, et al. Necrotizing Soft Tissue Infection: Diagnostic Accuracy of Physical Examination, Imaging, and LRINEC Score: A Systematic Review and Meta-Analysis. Ann Surg. 2019;269(1):58–65. doi:10.1097/SLA.0000000000002774 | duplicate |
| Fernando SM, Tran A, Cheng W, Rochwerg B, Taljaard M, Kyeremanteng K, et al. Diagnosis of elevated intracranial pressure in critically ill adults: Systematic review and meta-analysis. BMJ. 2019;366. doi:10.1136/bmj.l4225 | duplicate |
| Fernando SM, Tran A, Cheng W, Rochwerg B, Taljaard M, Kyeremanteng K, et al. Diagnosis of elevated intracranial pressure in critically ill adults: systematic review and meta-analysis. Bmj. 2019;366:l4225. Located at: 31340932. doi:10.1136/bmj.l4225 | population |
| Ferszt P, Hill J, Larson S. Ultrasonography relative to Cormack-Lehane in predicting difficult laryngoscopy: a systematic review of diagnostic test accuracy. JBI Evidence Synthesis. 2021 Jun;19(6):1444–51. doi:10.11124/JBIES-20-00141 | study design |
| Fields JM, Davis J, Girson L, Au A, Potts J, Morgan CJ, et al. Transthoracic Echocardiography for Diagnosing Pulmonary Embolism: A Systematic Review and Meta-Analysis. J Am Soc Echocardiogr. 2017;30(7):714-723.e4. doi:10.1016/j.echo.2017.03.004 | duplicate |
| Filippou G, Adinolfi A, Iagnocco A, Filippucci E, Cimmino MA, Bertoldi I, et al. Ultrasound in the diagnosis of calcium pyrophosphate dihydrate deposition disease. A systematic literature review and a meta-analysis. Osteoarthritis & Cartilage. 2016;24(6):973–81. | duplicate |
| Franca Neto AH de, Amorim MMR do, Nóbrega BMSV. Acute appendicitis in pregnancy: literature review. Rev Assoc Med Bras. 2015;61:170–7. doi:https://doi.org/10.1590/1806-9282.61.02.170 | study design |
| French C, Cartwright MS, Hobson-Webb LD, Boon AJ, Alter KE, Hunt CH, et al. Evidence-based guideline: Neuromuscular ultrasound for the diagnosis of carpal tunnel syndrome. Muscle and Nerve. 2012;46(2):287–93. | study design |
| Gaebel K, Kaulbk K, Robertson D, Blkhouse G, Xie F, Assasi N, et al. Clinical effectiveness of portable ultrasound in small emergency departments: a systematic review. Value in Health. 2009;12(7):A252–A252. | conference abstract |
| Gamberini L, Scquizzato T, Tartaglione M, Chiarini V, Mazzoli CA, Allegri D, et al. Diagnostic accuracy for hemoperitoneum, influence on prehospital times and time-to-definitive treatment of prehospital FAST: A systematic review and individual participant data meta-analysis. Injury. 2023 Jun;54(6):1421–31. doi:10.1016/j.injury.2023.03.024 | reference standard |
| Gan RK, Sanchez Martinez A, Abu Hasan MA, Castro Delgado R, Arcos Gonzalez P. Point-of-care ultrasonography in diagnosing necrotizing fasciitis-a literature review. Journal of Ultrasound. 2023;26(2):343–53. | study design |
| Gardner KF, Clattenburg EJ, Wroe P, Singh A, Mantuani D, Nagdev A. The Cardiac Arrest Sonographic Assessment (CASA) exam – A standardized approach to the use of ultrasound in PEA. The American Journal of Emergency Medicine. 2018;36(4):729–31. doi:10.1016/j.ajem.2017.08.052 | study design |
| Gasser CRB, Gehri M, Joseph JM, Pauchard JY. Is It Ovarian Torsion? A Systematic Literature Review and Evaluation of Prediction Signs. Pediatric Emergency Care. 2016 Apr;32(4):256. doi:10.1097/PEC.0000000000000621 | population |
| Gentilotti E, Nardo PD, Cremonini E, Górska A, Mazzaferri F, Canziani L, et al. Diagnostic accuracy of point-of-care tests in acute community acquired-lower respiratory tract infections. A systematic review and meta-analysis. Clinical Microbiology and Infection. 2021. doi:10.1016/j.cmi.2021.09.025 | duplicate |
| Gerner-Rasmussen J, Donatsky AM, Bjerrum F. The role of non-invasive imaging techniques in detecting intra-abdominal adhesions: a systematic review. Langenbecks Arch Surg. 2019 Sep;404(6):653–61. doi:10.1007/s00423-018-1732-8 | outcome measures |
| Ghanem G, Haase D, Brzezinski A, Ogawa R, Asachi P, Chiem A. Ultrasound detected increase in optic disk height to identify elevated intracranial pressure: a systematic review. Ultrasound J. 2023 May 25;15(1):26. doi:10.1186/s13089-023-00324-7 | outcome measures |
| Gianola S, Castellini G, Biffi A, Porcu G, Napoletano A, Coclite D, et al. Accuracy of risk tools to predict critical bleeding in major trauma: A systematic review with meta-analysis. J Trauma Acute Care Surg. 2022 Jun;92(6):1086–96. doi:10.1097/TA.0000000000003496 | study design |
| Giljaca V, Nadarevic T, Poropat G, Nadarevic VS, Stimac D. Diagnostic Accuracy of Abdominal Ultrasound for Diagnosis of Acute Appendicitis: Systematic Review and Meta-analysis. World J Surg. 2017;41(3):693–700. doi:10.1007/s00268-016-3792-7 | duplicate |
| Giljaca V, Nadarevic T, Poropat G, Stimac D. Abdominal ultrasound for diagnosis of acute appendicitis: Systematic review and meta-analysis of diagnostic accuracy studies. United European Gastroenterology Journal. 2015;3(5):A216–A216. doi:https://dx.doi.org/10.1177/2050640615601623 | conference abstract |
| Girardi L, Zarka F, Pradier M, Visintini S, Poletti M, Le Gal G, et al. Diagnostic performance of ultrasound, computed tomography and magnetic resonance imaging in portal vein thrombosis: A systematic review with meta-analysis. Thrombosis Research. 2026 Jan 1;257:109560. doi:10.1016/j.thromres.2025.109560 | setting |
| Gomes SH, Simões AM, Nunes AM, Pereira MV, Teoh WH, Costa PS, et al. Useful Ultrasonographic Parameters to Predict Difficult Laryngoscopy and Difficult Tracheal Intubation—A Systematic Review and Meta-Analysis. Front Med. 2021;8. doi:10.3389/fmed.2021.671658 | study design |
| Gonai S, Miyoshi T, da Silva Lopes K, Gilmour S. An umbrella review of systematic reviews and meta-analyses for assessment and treatment of acute shoulder dislocation. American Journal of Emergency Medicine. 2025;87:16–27. | study design |
| Goodacre S, Sampson F, Thomas S, Van Beek E, Sutton A. Systematic review and meta-analysis of the diagnostic accuracy of ultrasonography for deep vein thrombosis. BMC Med Imaging. 2005 Dec;5(1):6. doi:10.1186/1471-2342-5-6 | study design |
| Goodacre S, Stevenson M, Wailoo A, Sampson F, Sutton AJ, Thomas S. Optimal algorithm for evaluating suspected DVT. Journal of Family Practice [Internet]. 2006;55(9). Available from: ["https://ovidsp.ovid.com/ovidweb.cgi?T=JS&CSC=Y&NEWS=N&PAGE=fulltext&D=emed9&AN=44412107", "https://libkey.io/libraries/528/openurl?genre=article&aulast=Goodacre&issn=0094-3509&title=Journal+of+Family+Practice&atitle=Optimal+algorithm+for+evaluating+suspected+DVT&volume=55&issue=9&spage=754&epage=&date=2006&doi=&pmid=&sid=OVID:embase"] | study design |
| Gottesman RF, Sharma P, Robinson KA, Arnan M, Tsui M, Saber-Tehrani A, et al. Imaging Characteristics of Symptomatic Vertebral Artery Dissection: A Systematic Review. The Neurologist. 2012 Sep;18(5):255–60. doi:10.1097/NRL.0b013e3182675511 | outcome measures |
| Gottlieb M, Holladay D, Peksa G. Accuracy of ultrasound for endotracheal tube confirmation: A systematic review and meta-analysis. Acad Emerg Med. 2018 May;2018 Annual Meeting of the Society for Academic Emergency Medicine, SAEM 201825(S1). doi:10.1111/acem.13424 | conference abstract |
| Gottlieb M, Holladay D, Peksa GD. Point-of-Care Ocular Ultrasound for the Diagnosis of Retinal Detachment: A Systematic Review and Meta-Analysis. Acad Emerg Med. 2019;26(8):931–9. doi:10.1111/acem.13682 | duplicate |
| Gottlieb M, Holladay D, Peksa GD. Ultrasonography for the Confirmation of Endotracheal Tube Intubation: A Systematic Review and Meta-Analysis. Ann Emerg Med. 2018;72(6):627–36. doi:10.1016/j.annemergmed.2018.06.024 | duplicate |
| Gottlieb M, Long B, Koyfman A. Evaluation and Management of Aortic Stenosis for the Emergency Clinician: An Evidence-Based Review of the Literature. Journal of Emergency Medicine. 2018;55(1):34–41. | study design |
| Gottlieb M, Peksa GD, Pandurangadu AV, Nakitende D, Takhar S, Seethala RR. Utilization of ultrasound for the evaluation of small bowel obstruction: A systematic review and meta-analysis. Am J Emerg Med. 2018;36(2):234–42. doi:10.1016/j.ajem.2017.07.085 | duplicate |
| Gottlieb RH, Widjaja J, Tian L, Rubens DJ, Voci SL. Calf sonography for detecting deep venous thrombosis in symptomatic patients: Experience and review of the literature. J Clin Ultrasound. 1999 Oct;27(8):415–20. Located at: 21. doi:10.1002/(SICI)1097-0096(199910)27:8%3C415::AID-JCU1%3E3.0.CO;2-6 | study design |
| Griffiths E. Helicopter emergency medical services use of thoracic point of care ultrasound for pneumothorax: a systematic review and meta-analysis. Scand J Trauma Resusc Emerg Med. 2021 Dec;29(1):163. doi:10.1186/s13049-021-00977-0 | reference standard |
| Grimberg A, Shigueoka DC, Atallah ÁN, Ajzen S, Iared W. Diagnostic accuracy of sonography for pleural effusion: Systematic review. Sao Paulo Med J. 2010;128(2):90–5. doi:10.1590/s1516-31802010000200009 | duplicate |
| Habets J, Tanis W, Reitsma JB, van den Brink RBA, Mali WPTM, Chamuleau SAJ, et al. Are novel non-invasive imaging techniques needed in patients with suspected prosthetic heart valve endocarditis? A systematic review and meta-analysis. Eur Radiol. 2015;25(7):2125–33. doi:10.1007/s00330-015-3605-7 | duplicate |
| Haji-Hassan M, Lenghel LM, Bolboaca SD. Hand-held ultrasound of the lung: A systematic review. Diagnostics [Internet]. 2021;11(8). Available from: ["https://ovidsp.ovid.com/ovidweb.cgi?T=JS&CSC=Y&NEWS=N&PAGE=fulltext&D=emed22&DO=10.3390%2fdiagnostics11081381", "https://libkey.io/libraries/528/openurl?genre=article&aulast=Haji-Hassan&issn=2075-4418&title=Diagnostics&atitle=Hand-held+ultrasound+of+the+lung%3A+A+systematic+review&volume=11&issue=8&spage=1381&epage=&date=2021&doi=10.3390%2Fdiagnostics11081381&pmid=&sid=OVID:embase"] | study design |
| Haji-Hassan M, Lenghel LM, Bolboacă SD. Hand-Held Ultrasound of the Lung: A Systematic Review. Diagnostics. 2021 Jul 31;11(8):1381. doi:10.3390/diagnostics11081381 | study design |
| Hamid KA, Hasan R, Mohamed M, Caravallah L, Chandran A, Mohammad D. Sonographic Inferior Vena Cava Assessment to Predict Fluid Responsiveness in Patients with Sepsis. Pediatrics. 2019 Aug 1;144(2_MeetingAbstract):392–392. doi:10.1542/peds.144.2MA4.392 | conference abstract |
| Hamsher C. Whole-leg compression ultrasonography for suspected DVT. American Family Physician 82(2) (pp 203), 2010. 2010;Date of Publication:July 15. Located at: 361802697 | study design |
| Hansell L, Milross M, Delaney A, Tian D, Ntoumenopoulos G. TO 158 Diagnostic accuracy of lung ultrasound: A systematic review. Respirology. 2021 Apr;26(S2):101–101. doi:10.1111/resp.14021 | conference abstract |
| Hansell L, Milross M, Delaney A, Tian DH, Ntoumenopoulos G. Lung ultrasound has greater accuracy than conventional respiratory assessment tools for the diagnosis of pleural effusion, lung consolidation and collapse: a systematic review. Journal of Physiotherapy. 2021 Jan;67(1):41–8. doi:10.1016/j.jphys.2020.12.002 | population |
| Hassankhani A, Amoukhteh M, Jannatdoust P, Valizadeh P, Gholamrezanezhad A. A systematic review and meta-analysis on the diagnostic utility of ultrasound for clavicle fractures. Skeletal Radiol. 2024 Feb;53(2):307–18. doi:10.1007/s00256-023-04396-3 | reference standard |
| Ho YJ, Sung CW, Chen YC, Lien WC, Chang WT, Huang CH. Performance of Intra-arrest Echocardiography: A Systematic Review. The Western Journal of Emergency Medicine. 2024;25(2):166–74. | study design |
| Holladay D, Fullmer R, Peksa G, Gottlieb M. Ultrasound for the Diagnosis of Diverticulitis: A Systematic Review and Meta-analysis. West J Emerg Med. 2019;MEMC Abstracts Special Issue Supplement 2019 (Full Text)20(5.1):S1. | conference abstract |
| Hu QJ, Shen YC, Jia LQ, Guo SJ, Long HY, Pang CS, et al. Diagnostic performance of lung ultrasound in the diagnosis of pneumonia: a bivariate meta-analysis. International journal of clinical and experimental medicine. 2014;7(1):115–21. | duplicate |
| Hunter F, Thomson K. Towards evidence based emergency medicine: Best BETs from the Manchester Royal Infirmary. BET 1: Emergency physician performed 2-point bedside compression ultrasound for deep venous thrombosis. Emerg Med J. 2014;31(11):944–6. Located at: 25326498. doi:10.1136/emermed-2014-204290.1 | study design |
| Huo YL, Jiao D, Yang DY, Zheng B, Song XT. A meta analysis on diagnostic effectiveness of ultrasound in diagnosis of acute pulmonary edema in ICU. International Journal of Clinical and Experimental Medicine. 2017;10(2):3345–51. Located at: 614585825 | setting |
| Hwang ME. Sonography and Computed Tomography in Diagnosing Acute Appendicitis. Radiol Technol. 2018 Jan;89(3):224–37. PubMed PMID: 29298941. | conference abstract |
| Islam N, Salameh JP, Leeflang MM, Hooft L, McGrath TA, van der Pol CB, et al. Thoracic imaging tests for the diagnosis of COVID-19. Cochrane Database Syst Rev. 2020;11:CD013639. Located at: 33242342. doi:10.1002/14651858.CD013639.pub3 | study design |
| Jacobsen N, Pietersen PI, Nolsoe C, Konge L, Graumann O, Laursen CB. Clinical Applications of Contrast-Enhanced Thoracic Ultrasound (CETUS) Compared to Standard Reference Tests: A Systematic Review. Ultraschall in der Medizin. 2022;43(1):72–81. | outcome measures |
| Jafari Khouzani P, Rahmani E, Rezaei M, Pohrbagher Benam S, Moghadam Fard A, Amani-Beni R, et al. Optic Nerve Sheath Diameter in Predicting the Neurological Outcomes of Cardiac Arrest Survivors: A Systematic Review and Meta-analysis. Arch. 2025;13(1):e29. Located at: 40027220. doi:10.22037/aaemj.v13i1.2486 | setting |
| Jahromi AS, Cinà CS, Liu Y, Clase CM. Sensitivity and specificity of color duplex ultrasound measurement in the estimation of internal carotid artery stenosis: A systematic review and meta-analysis. J Vasc Surg. 2005;41(6):962–72. doi:10.1016/j.jvs.2005.02.044 | population |
| Jain A, Mehta N, Schecter J, Pandya S, Secko M, Sinert R. History, physical exam, plus laboratory testing and ultrasonography for the diagnosis of cholecystitis: An evidence-based review. Academic Emergency Medicine. 2014;21(5):S204–5. doi:https://dx.doi.org/10.1111/acem.12365 | conference abstract |
| Jari R, Alfuraih AM, McLaughlan JR. The diagnostic performance of lung ultrasound for detecting COVID ‐19 in emergency departments: A systematic review and meta‐analysis. J of Clinical Ultrasound. 2022 Jun;50(5):618–27. doi:10.1002/jcu.23184 | duplicate |
| Jeffers K, Keim SM, Long B, Gottlieb M, Adhikari SR. What is the Utility of Point-of-Care Ultrasound for Diagnosing Abdominal Aortic Aneurysm? Journal of Emergency Medicine. 2026;82:82–7. doi:10.1016/j.jemermed.2025.12.006 | study design |
| Jeffers K, Keim SM, Long B, Gottlieb M, Adhikari SR. What is the Utility of Point-of-Care Ultrasound for Diagnosis of Soft Tissue Abscess vs. Cellulitis? Journal of Emergency Medicine. 2025;72(pp 121-128). Located at: 2038473684. doi:10.1016/j.jemermed.2024.11.012 | study design |
| Jenkins S, Alabed S, Swift A, Marques G, Ryding A, Sawh C, et al. Diagnostic accuracy of handheld cardiac ultrasound device for assessment of left ventricular structure and function: systematic review and meta-analysis. Heart. 2021 Nov;107(22):1826–34. doi:10.1136/heartjnl-2021-319561 | index test |
| Jiang L, Wu J, Feng X. The value of ultrasound in diagnosis of pneumoperitoneum in emergent or critical conditions: A meta-analysis. Hong Kong Journal of Emergency Medicine. 2019 Mar;26(2):111–7. doi:10.1177/1024907918805668 | reference standard |
| Joyce A, Snelling PJ, Elsayed T, Keijzers G. Point-of-care ultrasound to diagnose acute cholecystitis in the emergency department: A scoping review. Australas J Ultrasound Med. 2024;27(1):26–41. Located at: 38434543. doi:10.1002/ajum.12371 | study design |
| Kagima J, Stolbrink M, Masheti S, Mbaiyani C, Munubi A, Joekes E, et al. Diagnostic accuracy of combined thoracic and cardiac sonography for the diagnosis of pulmonary embolism: A systematic review and meta-analysis. PLoS ONE [Electronic Resource]. 2020;15(9):e0235940. | duplicate |
| Kagima J, Stolbrink M, Masheti S, Mbaiyani C, Munubi A, Joekes E, et al. Diagnostic accuracy of combined thoracic and cardiac sonography for the diagnosis of pulmonary embolism: A systematic review and meta-analysis. PLoS ONE. 2020;15(9 September). doi:10.1371/journal.pone.0235940 | duplicate |
| Kannady J, Siahaan AMP, Harahap MRA, Aprilia E, Ivander A. Operator and Modality-Dependent Limitations of Optic Nerve Sheath Diameter Sonography for Raised ICP in TBI: A Meta-Regression of Diagnostic Accuracy. Neurocritical Care. 2026. doi:10.1007/s12028-026-02493-4 | population |
| Karanasios S, Korakakis V, Moutzouri M, Drakonaki E, Koci K, Pantazopoulou V, et al. Diagnostic accuracy of examination tests for lateral elbow tendinopathy (LET) – A systematic review. Journal of Hand Therapy. 2022 Oct;35(4):541–51. doi:10.1016/j.jht.2021.02.002 | study design |
| Karigowda L, Gupta B, Elkady H, Deshpande K. Diagnostic Accuracy of Ultrasound in Intensive Care Patients with Undifferentiated Shock: A Systematic Review and Meta-analysis. Indian J. 2024;28(12):1159–69. Located at: 39759796. doi:10.5005/jp-journals-10071-24851 | setting |
| Kassaï B, Boissel JP, Cucherat M, Sonie S, Shah N, Leizorovicz A. A systematic review of the accuracy of ultrasound in the diagnosis of deep venous thrombosis in asymptomatic patients. Thromb Haemost. 2004;91(04):655–66. doi:10.1160/TH03-11-0722 | setting |
| Kataria H, Mcvicar J. Break the fast with feast (EFAST)? Journal of Emergency Medicine. 2012;43(5):938–938. doi:https://dx.doi.org/10.1016/j.jemermed.2012.09.114 | conference abstract |
| Kedan I, Ciozda W, Palatinus JA, Palatinus HN, Kimchi A. Prognostic value of point-of-care ultrasound during cardiac arrest: A systematic review. Cardiovasc Ultrasound. 2020;18(1). doi:10.1186/s12947-020-0185-8 | outcome measures |
| Keikha M, Salehi-Marzijarani M, Soldoozi Nejat R, Sheikh Motahar Vahedi H, Mirrezaie SM. Diagnostic Accuracy of Rapid Ultrasound in Shock (RUSH) Exam; A Systematic Review and Meta-analysis. BEAT. 2018 Oct 1;6(4):271–8. doi:10.29252/beat-060402 | study design |
| Khattab E, Schafer J, Balk D, Khuder S, Beals T, Hoffmann B, et al. Accuracy of ocular ultrasonography for the diagnosis of retinal detachment: A meta-analysis. Academic Emergency Medicine. 2019 May;SAEM Annual Meeting Abstracts26(S1). doi:10.1111/acem.13756 | conference abstract |
| Kheir M, Dong V, Roselli V, Mina B. The role of ultrasound in predicting non-invasive ventilation outcomes: a systematic review. Front Med. 2023 Oct 31;10:1233518. doi:10.3389/fmed.2023.1233518 | study design |
| Kim DW, Suh CH, Yoon HM, Kim JR, Jung AY, Lee JS, et al. Visibility of Normal Appendix on CT, MRI, and Sonography: A Systematic Review and Meta-Analysis. American Journal of Roentgenology. 2018 Sep;211(3):W140–50. doi:10.2214/AJR.17.19321 | population |
| Kives S, Gascon S, Dubuc É, Eyk NV. Diagnosis and Management of Adnexal Torsion in Children, Adolescents, and Adults. Journal of Obstetrics and Gynaecology Canada. 2017 Feb 1;39(2):82–90. doi:10.1016/j.jogc.2016.10.001 PubMed PMID: 28241927. | outcome measures |
| Klatkiewicz T, Gawriołek K, Pobudek Radzikowska M, Czajka-Jakubowska A. Ultrasonography in the Diagnosis of Temporomandibular Disorders: A Meta-Analysis. Med Sci Monit. 2018 Feb 8;24:812–7. doi:10.12659/MSM.908810 | setting |
| Ko SY, Ghotb Razmjou S, Haagsma JA, Verdonschot RJCG. Point-of-Care Ultrasound vs. Manual Palpation for Pulse Check in Cardiac Arrest Patients: A Systematic Review. Journal of Emergency Medicine. 2026;80:112–8. doi:10.1016/j.jemermed.2025.09.019 | study design |
| Kok B, Wolthuis D, Bosch F, van der Hoeven H, Blans M. POCUS in dyspnea, nontraumatic hypotension, and shock; a systematic review of existing evidence. European Journal of Internal Medicine. 2022;106:9–38. | study design |
| Koziarz A, Sne N, Kegel F, Nath S, Badhiwala JH, Nassiri F, et al. Bedside Optic Nerve Ultrasonography for Diagnosing Increased Intracranial Pressure: A Systematic Review and Meta-analysis. Ann Intern Med. 2019;171(12):896–905. Located at: 31739316. doi:10.7326/M19-0812 | duplicate |
| Kraaijenhagen RA, Lensing AW, Lijmer JG, Prandoni P, Prins MH, Ginsberg JS, et al. Diagnostic strategies for the management of patients with clinically suspected deep-vein thrombosis. Current Opinion in Pulmonary Medicine. 1997;3(4):268–74. | study design |
| Krill MK, Rosas S, Kwon K, Dakkak A, Nwachukwu BU, McCormick F. A concise evidence-based physical examination for diagnosis of acromioclavicular joint pathology: a systematic review. The Physician and Sportsmedicine. 2018 Jan 2;46(1):98–104. doi:10.1080/00913847.2018.1413920 | study design |
| Kroese LF, Sneiders D, Kleinrensink GJ, Muysoms F, Lange JF. Comparing different modalities for the diagnosis of incisional hernia: a systematic review. Hernia. 2018;22(2):229–42. | outcome measures |
| Lalande E, Burwash-Brennan T, Burns K, Atkinson P, Lambert M, Jarman B, et al. Is point-of-care ultrasound a reliable predictor of outcome during atraumatic, non-shockable cardiac arrest? A systematic review and meta-analysis from the SHoC investigators. Resuscitation. 2019;139:159–66. doi:10.1016/j.resuscitation.2019.03.027 | duplicate |
| Larson J, Lohse M, Bracey A, Reardon L, Fernandes RF, Sagyi S, et al. The diagnostic accuracy of point-of-care ultrasound for small bowel obstruction: A prospective study. Academic Emergency Medicine. 2020 May;SAEM Annual Meeting Abstracts27(S1):S216-217. doi:10.1111/acem.13961 | conference abstract |
| Latham SK, Smith TO. The diagnostic test accuracy of ultrasound for the detection of lateral epicondylitis: A systematic review and meta-analysis. Orthopaedics & Traumatology: Surgery & Research. 2014 May;100(3):281–6. doi:10.1016/j.otsr.2014.01.006 | reference standard |
| Lee SH, Yun SJ. Ankle ultrasound for detecting anterior talofibular ligament tear using operative finding as reference standard: a systematic review and meta-analysis. European Journal of Trauma & Emergency Surgery. 2020;46(1):73–81. | outcome measures |
| Lee SH, Yun SJ. Efficiency of knee ultrasound for diagnosing anterior cruciate ligament and posterior cruciate ligament injuries: a systematic review and meta-analysis. Skeletal Radiol. 2019 Oct;48(10):1599–610. doi:10.1007/s00256-019-03225-w | setting |
| Lewiss RE, Kaban NL, Saul T. Point-of-care ultrasound for a deep venous thrombosis. Global Heart. 2013;8(4):329–33. | study design |
| Li R, Hung K, Wang X, Leung LY, Hui T, Wong R, et al. Diagnostic accuracy of point-of-care ultrasound for the detection of acute aortic syndrome and thoracic aortic aneurysm in the emergency department: systematic review and meta-analysis. European Heart Journal. 2025;46(Supplement_1). doi:10.1093/eurheartj/ehaf784.2963 | conference abstract |
| Li X, Song J, Sun Z, Yang Y, Lin Y, Wang H. Conventional Ultrasound and Contrast‐Enhanced Ultrasound in the Diagnosis of Splenic Diseases: A Systematic Review and Meta‐analysis. J of Ultrasound Medicine. 2020 Sep;39(9):1687–94. doi:10.1002/jum.15291 | reference standard |
| Li Y, Fang H, Xia W, Xu S, Guo M. Diagnostic accuracy of transvaginal ultrasound-measured endometrial thickness and serum hCG levels in detecting retained products of conception: a systematic review and meta-analysis. Abdom Radiol. 2026 Mar 3. doi:10.1007/s00261-026-05424-1 | duplicate |
| Liaghat B, Pedersen JR, Husted RS, Pedersen LL, Thorborg K, Juhl CB. Diagnosis, prevention and treatment of common shoulder injuries in sport: grading the evidence - a statement paper commissioned by the Danish Society of Sports Physical Therapy (DSSF). British Journal of Sports Medicine. 2023;57(7):408–16. | study design |
| Lin KT, Lin ZY, Huang CC, Yu SY, Huang JL, Lin JH, et al. Prehospital ultrasound scanning for abdominal free fluid detection in trauma patients: a systematic review and meta-analysis. BMC Emerg Med. 2024 Jan 7;24(1):7. doi:10.1186/s12873-023-00919-2 | reference standard |
| Lin YC, Yu YC, Huang YT, Wu YY, Wang TC, Huang WC, et al. Diagnostic accuracy of ultrasound for small bowel obstruction: A systematic review and meta-analysis. Eur J Radiol. 2021;136. doi:10.1016/j.ejrad.2021.109565 | duplicate |
| Lindow T, Quadrelli S, Ugander M. Noninvasive Imaging Methods for Quantification of Pulmonary Edema and Congestion: A Systematic Review. JACC: Cardiovascular Imaging. 1469;16(11):1469–84. | study design |
| Liu F, Dong J, Shen WJ, Kang Q, Zhou D, Xiong F. Detecting Rotator Cuff Tears: A Network Meta-analysis of 144 Diagnostic Studies. Orthopaedic Journal of Sports Medicine. 2020 Feb 1;8(2):2325967119900356. doi:10.1177/2325967119900356 | study design |
| Liu K, Li X, Weng Q, Wang Y, Wei J, Zeng C, et al. Diagnostic Accuracy of Ultrasound for Assessment of Synovial Abnormalities Among Patients With Knee Pain: A Meta-Analysis. Arthritis Care Res (Hoboken). 2024;76(2):295–303. Located at: 37522287. doi:10.1002/acr.25205 | duplicate |
| Liu WJ. The diagnosis of intracranial artery stenosis in patients with stroke by transcranial Doppler ultrasound: A meta-analysis. Technol Health Care. 2024;32(2):639–49. Located at: 37661896. doi:10.3233/THC-220844 | duplicate |
| Liu X, Hui BT, Way C, Beese S, Adriano A, Keane PA, et al. Noninvasive Instrument-based Tests for Detecting and Measuring Vitreous Inflammation in Uveitis: A Systematic Review. Ocular Immunology & Inflammation. 2022;30(1):137–48. | setting |
| Llamas‐Álvarez A, Tenza-Lozano E, Latour‐Pérez J. Accuracy of Lung Ultrasonography in the Diagnosis of Pneumonia in Adults: Systematic Review and Meta‐Analysis. Chest. 2017. doi:10.1016/j.chest.2016.10.039 | outcome measures |
| Lochner P, Leone MA, Coppo L, Nardone R, Zedde ML, Cantello R, et al. B-mode transorbital ultrasononography for the diagnosis of acute optic neuritis. A systematic review. Clinical Neurophysiology. 2016;127(1):803–9. | outcome measures |
| Long E, Oakley E, Duke T, Babl FE. Does Respiratory Variation in Inferior Vena Cava Diameter Predict Fluid Responsiveness: A Systematic Review and Meta-Analysis. Shock. 2017 May;47(5):550–9. doi:10.1097/SHK.0000000000000801 | setting |
| Machado PMMC, Koevoets R, Bombardier C, Van Der Heijde DM. The Value of Magnetic Resonance Imaging and Ultrasound in Undifferentiated Arthritis: A Systematic Review. The Journal of Rheumatology Supplement. 2011 Mar 1;87(0):31–7. doi:10.3899/jrheum.101072 | study design |
| Mackie S, Koduri G, Hill CL, Hutchings A, Wakefield RJ, Dasgupta B, et al. The use of imaging in the diagnosis of polymyalgia rheumatica: Systematic literature review and meta-analysis. Arthritis & Rheumatology. 2014 Oct;2014 ACR/ARHP Annual Meeting Abstract Supplement66(S10). doi:10.1002/art.38914 | conference abstract |
| Mackie SL, Koduri G, Hill CL, Wakefield RJ, Hutchings A, Loy C, et al. Accuracy of musculoskeletal imaging for the diagnosis of polymyalgia rheumatica: systematic review. RMD Open. 2015 Aug;1(1):e000100. doi:10.1136/rmdopen-2015-000100 | population |
| MacLeod JR, Kivell MJ, Shivgulam ME, Liu H, O’Brien MW. Accuracy of Duplex Ultrasound for Detecting Renal Artery Stenosis: A Systematic Review. Journal for Vascular Ultrasound. 2024;48(1):15–27. | outcome measures |
| Maconi G, Greco MT, Asthana AK. Transperinealer Ultraschall bei perianalen Fisteln und Abszessen - Systematischer Uberblick und Metaanalyse, Transperineal Ultrasound for Perianal Fistulas and Abscesses - A Systematic Review and Meta-Analysis. Ultraschall in der Medizin. 1980;38(3):265–72. Located at: 623029676. doi:10.1055/s-0043-103954 | outcome measures |
| Malde S, Nambiar AK, Umbach R, Lam TB, Bach T, Bachmann A, et al. Systematic Review of the Performance of Noninvasive Tests in Diagnosing Bladder Outlet Obstruction in Men with Lower Urinary Tract Symptoms [figure presented]. Eur Urol. 2017;71(3):391–402. doi:10.1016/j.eururo.2016.09.026 | outcome measures |
| Malik FA, Raheem A, Selvam SA, Patel UK, Mahmood SN. Diagnostic Accuracy of Point-of-Care Ultrasound (POCUS) in Patients With Respiratory Tract Disorders - A Systematic Review and Meta-analysis. In: A45. ICU PRACTICES, QUALITY IMPROVEMENT, AND MEDICAL EDUCATION [Internet]. American Thoracic Society; 2023 [cited 2025 Jun 29]. p. A1660–A1660. Available from: https://www.atsjournals.org/doi/10.1164/ajrccm-conference.2023.207.1_MeetingAbstracts.A1660 doi:10.1164/ajrccm-conference.2023.207.1_MeetingAbstracts.A1660 | conference abstract |
| Man J, Wang H, Yang L. Re: Cameron E. Alexander, Hannah Warren, Alexander Light, et al. Ultrasound for the Diagnosis of Testicular Torsion: A Systematic Review and Meta-analysis of Diagnostic Accuracy. Eur Urol Focus. In press. https://doi.org/10.1016/j.euf.2025.04.026. European urology focus. 2025. Located at: 648811251. doi:10.1016/j.euf.2025.07.023 | study design |
| Mandeville JC, Colebourn CL. Can transthoracic echocardiography be used to predict fluid responsiveness in the critically ill patient? A systematic review. Critical Care Research & Practice. 2012;2012:513480. | population |
| Mant J, Doust J, Roalfe A, Barton P, Cowie MR, Glasziou P, et al. Systematic review and individual patient data meta-analysis of diagnosis of heart failure, with modelling of implications of different diagnostic strategies in primary care. Health Technol Assess. 2009;13(32):i–207. doi:10.3310/hta13320 | index test |
| Marbach JA, Almufleh A, Di Santo P, Jung R, Simard T, McInnes M, et al. Comparative Accuracy of Focused Cardiac Ultrasonography and Clinical Examination for Left Ventricular Dysfunction and Valvular Heart Disease: A Systematic Review and Meta-analysis. Ann Intern Med. 2019 Aug 20;171(4):264–72. doi:10.7326/M19-1337 | index test |
| Martindale JL, Wakai A, Collins SP, Levy PD, Diercks D, Hiestand BC, et al. Diagnosing Acute Heart Failure in the Emergency Department: A Systematic Review and Meta-analysis. Acad Emerg Med. 2016;23(3):223–42. doi:10.1111/acem.12878 | duplicate |
| Mathias RM, Chu MKW, Edwards S, Day AS, Ma C, Jairath V, et al. Meta-analysis: Intestinal ultrasound to evaluate colonic contents and constipation. Alimentary Pharmacology and Therapeutics. 2024;60(1):6–16. | population |
| Matthew Fields J, Davis J, Alsup C, Bates A, Au A, Adhikari S, et al. Accuracy of Point-of-care Ultrasonography for Diagnosing Acute Appendicitis: A Systematic Review and Meta-analysis. Acad Emerg Med. 2017;24(9):1124–36. doi:10.1111/acem.13212 | duplicate |
| Matthews W, Ellis R, Furness J, Hing W. Classification of Tendon Matrix Change Using Ultrasound Imaging: A Systematic Review and Meta-analysis. Ultrasound in Medicine and Biology. 2059;44(10):2059–80. | study design |
| Matthies A, Trauer M, Chopra K, Jarman R. 1116 Diagnostic accuracy of point-of-care lung ultrasound for COVID-19: a meta-analysis of 19 studies including 3954 patients. Emerg Med J. 2022 Mar 1;39(3):250–1. doi:10.1136/emermed-2022-RCEM.16 | conference abstract |
| Matthies A, Trauer MM, Chopra K, Jarman R. Diagnostic accuracy of point-of-care lung ultrasound for COVID-19: a systematic review and meta-analysis. Emergency Medicine Journal. 2021. doi:10.1136/emermed-2021-212092 | duplicate |
| Maw A, Hassanin A, Sertich AP, Salame G. Accuracy of lung ultrasound versus chest X-ray in the diagnosis of pulmonary edema: A diagnostic test accuracy systematic review with meta-analysis. Journal of general internal medicine : 2018;33:104. | conference abstract |
| Maw AM, Hassanin A, Ho PM, McInnes MDF, Moss A, Juarez-Colunga E, et al. Diagnostic Accuracy of Point-of-Care Lung Ultrasonography and Chest Radiography in Adults With Symptoms Suggestive of Acute Decompensated Heart Failure: A Systematic Review and Meta-analysis. JAMA Netw Open. 2019;2(3):e190703. doi:10.1001/jamanetworkopen.2019.0703 | duplicate |
| McDowall J, Adam A, Gerber L, Enyuma COA, Aigbodion SJ, Buchanan S, et al. The ultrasonographic “whirlpool sign” in testicular torsion: valuable tool or waste of valuable time? A systematic review and meta-analysis. Emerg Radiol. 2018;25(3):281–92. doi:10.1007/s10140-018-1579-x | duplicate |
| McGivery K, Atkinson PR, Lewis D, Taylor L, Gadd K. Point of care ultrasound for lung B-lines in the early diagnosis of acute decompensated heart failure in the emergency department: A systematic review. Canadian Journal of Emergency Medicine. 2016;18:S55–S55. doi:https://dx.doi.org/10.1017/cem.2016.111 | conference abstract |
| McLaren PSM. A systematic review on the utility of ultrasonography in the diagnosis of testicular torsion in acute scrotum patients. Radiography. 2021 Aug;27(3):943–9. doi:10.1016/j.radi.2020.12.012 | study design |
| McRae A, Murray H, Edmonds M. Diagnostic accuracy and clinical utility of emergency department targeted ultrasonography in the evaluation of first-trimester pelvic pain and bleeding: a systematic review. CJEM. 2009 Jul;11(4):355–64. doi:10.1017/s1481803500011416 PubMed PMID: 19594975. | outcome measures |
| Mehta D, Susheela AT, Lei YW, Ganti N, Dirisanala S, Laller S, et al. DIAGNOSTIC VALUE OF POCUS IN ACUTE APPENDICITIS: A SYSTEMATIC REVIEW AND META META-ANALYSIS. Gastroenterology : 2022;162:S-665. doi:10.1016/S0016-5085%252822%252961560-9 | conference abstract |
| Melo RH, Gioli-Pereira L, Lourenço ID, Da Hora Passos R, Bernardo AT, Volpicelli G. Diagnostic accuracy of multi-organ point-of-care ultrasound for pulmonary embolism in critically ill patients: a systematic review and meta-analysis. Crit Care. 2025;29(1). doi:10.1186/s13054-025-05359-x | duplicate |
| Menegozzo CAM, Artifon ELA, Meyer-Pflug AR, Rocha MC, Utiyama EM. Can ultrasound be used as an adjunct for tube thoracostomy? A systematic review of potential application to reduce procedure-related complications. International Journal of Surgery. 2019 Aug;68:85–90. doi:10.1016/j.ijsu.2019.06.012 | study design |
| Mercer CB, Ball M, Cash RE, Rivard MK, Chrzan K, Panchal AR. Ultrasound Use in the Prehospital Setting for Trauma: A Systematic Review. Prehospital Emergency Care. 2021;25(4):566–82. | study design |
| Meyer R, Lin C, Yenokyan G, Ellen M. Diagnostic Utility of Ultrasound Versus Physical Examination in Assessing Knee Effusions: A Systematic Review and Meta-analysis. J Ultrasound Med. 2022;41(1):17–31. doi:10.1002/jum.15676 | duplicate |
| Miksch M, Koch M, Mantsopoulos K, Gostian AO, Mueller SK, Rupp R, et al. Sonografische Diagnostik tonsillärer Abszesse: Eigene Erfahrungen und eine systematische Übersicht. Laryngorhinootologie. 2022 Sep;101(09):706–12. doi:10.1055/a-1744-6305 | study design |
| Miladi S, Boussaa H, Fazaa A, Sellami M, Zakraoui L, Abdelghani K, et al. Performance of ultrasonography in diagnosing gout. Indian J Rheumatol. 2022;17(4):405. doi:10.4103/injr.injr_83_21 | study design |
| Miller LE, Hammert WC, Rekant MS, Fowler JR. Diagnostic Accuracy of Neuromuscular Ultrasound vs. Electrodiagnostic Studies for Carpal Tunnel Syndrome: Systematic Review and Meta-analysis of Paired Accuracy Studies. Hand. 2024;15589447241278972. Located at: 39324685. doi:10.1177/15589447241278972 | population |
| Mills L, Morley EJ, Soucy Z, Vilke GM, Lam SHF. Ultrasound for the Diagnosis and Management of Suspected Urolithiasis in the Emergency Department. The Journal of Emergency Medicine. 2018 Feb;54(2):215–20. doi:10.1016/j.jemermed.2017.09.020 | study design |
| Milsom SA, Sweeting JA, Sheahan H, Haemmerle E, Windsor JA. Naso-enteric Tube Placement: A Review of Methods to Confirm Tip Location, Global Applicability and Requirements. World Journal of Surgery. 2015;39(9):2243–52. | study design |
| Minnigan HJ, Snead GR, Vrablik ME, Kirschner JM, Seupaul RA. The diagnostic accuracy of bedside ocular ultrasound in the diagnosis of retinal detachment: A systematic review and meta-analysis. Academic Emergency Medicine. 2013;20(5):S215–S215. doi:https://dx.doi.org/10.1111/acem.12115 | conference abstract |
| Mishra L, Rath C, Wibrow B, Anstey M, Ho K. Acoustic Shadowing to Facilitate Ultrasound Guided Arterial Cannulation: A Systematic Review and Meta-analysis of Randomized Controlled Trials. Indian J. 2024;28(7):677–85. Located at: 38994254. doi:10.5005/jp-journals-10071-24751 | setting |
| Mohseni-Bandpei MA, Nakhaee M, Mousavi ME, Shakourirad A, Safari MR, Vahab Kashani R. Application of ultrasound in the assessment of plantar fascia in patients with plantar fasciitis: A systematic review. Ultrasound in Medicine and Biology. 2014;40(8):1737–54. Located at: 53123346. doi:10.1016/j.ultrasmedbio.2014.03.001 | setting |
| Nakajima E, Moon FH, Junior NC, Macedo CR, De Souza AWS, Iared W. Accuracy of Doppler ultrasound in the diagnosis of giant cell arteritis: a systematic review and meta-analysis. Adv Rheumatol. 2023 Feb 8;63(1):5. doi:10.1186/s42358-023-00286-3 | study design |
| Naraghi L, Beals T, Schafer J, Balk D, Lee C, Hoffmann D. Point-of-care cardiopulmonary ultrasound for the diagnosis of acute decompensated heart failure: A systematic review and meta-analysis. Academic Emergency Medicine. 2018 May;25(S1):1553–2712. doi:10.1111/acem.13424 | conference abstract |
| Nederkoorn PJ, Van Der Graaf Y, Hunink MGM. Duplex ultrasound and magnetic resonance angiography compared with digital subtraction angiography in carotid artery stenosis: A systematic review. Stroke. 2003;34(5):1324–31. doi:10.1161/01.STR.0000068367.08991.A2 | study design |
| Neto ES, Scapin M, Lazaro-Paulina F, Campbell RL, Molinari DF, Kummer T. Duration of resuscitation interruption using point-of-care ultrasound versus traditional manual pulse check: A systematic review and meta-analysis. Am J Emerg Med. 2025;98:145–52. Located at: 40876216. doi:10.1016/j.ajem.2025.08.049 | study design |
| Netterström-Wedin F, Bleakley C. Diagnostic accuracy of clinical tests assessing ligamentous injury of the ankle syndesmosis: A systematic review with meta-analysis. Physical Therapy in Sport. 2021 May;49:214–26. doi:10.1016/j.ptsp.2021.03.005 | index test |
| Newberry SJ, FitzGerald J, Maglione MA, O’Hanlon CE, Han D, Booth M, et al. Diagnosis of Gout [Internet]. 2016. Available from: https://ovidsp.ovid.com/ovidweb.cgi?T=JS&PAGE=reference&D=medp&NEWS=N&AN=26985540https://libkey.io/libraries/528/openurl?genre=article&aulast=Newberry&issn=&title=%3D%3D%3D&atitle=Diagnosis+of+Gout&volume=&issue=&spage=&epage=&date=2016&doi=&pmid=26985540 | duplicate |
| Newberry SJ, FitzGerald J, Maglione MA, O’Hanlon CE, Han D, Booth M, et al. Diagnosis of Gout. Agency for Healthcare Research and Quality (US). 2016;15(16):02. Located at: 26985540 | outcome measures |
| Odedra D, Mellnick VM, Patlas MN. Imaging of Blunt Pancreatic Trauma: A Systematic Review. Can Assoc Radiol J. 2020 Aug;71(3):344–51. doi:10.1177/0846537119888383 | study design |
| Ohle R, McIsaac SM, Woo MY, Perry JJ. Sonography of the Optic Nerve Sheath Diameter for Detection of Raised Intracranial Pressure Compared to Computed Tomography: A Systematic Review and Meta‐analysis. J of Ultrasound Medicine. 2015 Jul;34(7):1285–94. doi:10.7863/ultra.34.7.1285 | reference standard |
| Ohle R, McIsaac SM, Woo MY, Perry JJ. Sonography of the optic nerve sheath diameter for detection of raised intracranial pressure compared to computed tomography: A systematic review and meta-analysis. J Ultrasound Med. 2015;34(7):1285–94. doi:10.7863/ultra.34.7.1285 | duplicate |
| Omar M, Jabir A, Khan I, Novelli E, Xu J. Diagnostic Test Accuracy of Lung Ultrasound for Acute Chest Syndrome in Sickle Cell Disease: A Systematic Review and Meta-analysis. Chest. 2022. doi:10.1016/j.chest.2022.11.042 | duplicate |
| Omar M, Jabir AR, Khan I, Novelli EM, Xu JZ. Diagnostic Test Accuracy of Lung Ultrasound for Acute Chest Syndrome in Sickle Cell Disease: A Systematic Review and Meta-analysis. Chest. 2023;163(6):1506–18. doi:https://dx.doi.org/10.1016/j.chest.2022.11.042 | population |
| Orso D, Fonda F, Bove T. Lung ultrasound vs chest radiography for pneumonia diagnosis in critically ill patients in the intensive care unit: a systematic review and Bayesian network diagnostic meta-analysis. EClinicalMedicine. 2025;89:103553. doi:10.1016/j.eclinm.2025.103553 | population |
| Orso D, Guglielmo N, Copetti R. Lung ultrasound in diagnosing pneumonia in the emergency department: A systematic review and meta-analysis. Eur J Emerg Med. 2018;25(5):312–21. doi:10.1097/MEJ.0000000000000517 | duplicate |
| Orso D, Paoli I, Piani T, Cilenti FL, Cristiani L, Guglielmo N. Accuracy of Ultrasonographic Measurements of Inferior Vena Cava to Determine Fluid Responsiveness: A Systematic Review and Meta-Analysis. J Intensive Care Med. 2020 Apr;35(4):354–63. doi:10.1177/0885066617752308 | reference standard |
| Osawa T, Nakayama N, Ishizu T, Kondo T, Nakashima T, Yamamoto T, et al. Diagnostic Accuracy of Point-of-Care Ultrasound for Patients With Cardiogenic Shock　― A Meta-Analysis and Systematic Review ―. Circ Rep. 2025;7(9):727–34. doi:10.1253/circrep.cr-25-0105 | duplicate |
| Paliwoda ED, Newman-Plotnick H, Buzzetta AJ, Post NK, LaClair JR, Trandafirescu M, et al. Acute Management of Nasal Bone Fractures: A Systematic Review and Practice Management Guideline. American Surgeon [Internet]. 2025;31348251358429. Available from: ["https://ovidsp.ovid.com/ovidweb.cgi?T=JS&CSC=Y&NEWS=N&PAGE=fulltext&D=emed28&DO=10.1177%2f00031348251358429", "https://libkey.io/libraries/528/openurl?genre=article&aulast=Paliwoda&issn=0003-1348&title=American+Surgeon&atitle=Acute+Management+of+Nasal+Bone+Fractures%3A+A+Systematic+Review+and+Practice+Management+Guideline&volume=&issue=&spage=00031348251358429&epage=&date=2025&doi=10.1177%2F00031348251358429&pmid=40638849&sid=OVID:embase"] | study design |
| Parekh P. A systematic review: the past, present and future of biliary imaging. Clinical Radiology. 2013 Sep;68:S2–3. doi:10.1016/j.crad.2013.05.007 | conference abstract |
| Parenti N, Palazzi C, Parenti M, D’Addato S. Could internal jugular vein ultrasound be useful in the assessment of patients with heart failure? A systematic review. Italian Journal of Medicine [Internet]. 2024;18(2). Available from: ["https://ovidsp.ovid.com/ovidweb.cgi?T=JS&CSC=Y&NEWS=N&PAGE=fulltext&D=emed27&DO=10.4081%2fitjm.2024.1726", "https://libkey.io/libraries/528/openurl?genre=article&aulast=Parenti&issn=1877-9344&title=Italian+Journal+of+Medicine&atitle=Could+internal+jugular+vein+ultrasound+be+useful+in+the+assessment+of+patients+with+heart+failure%3F+A+systematic+review&volume=18&issue=2&spage=1726&epage=&date=2024&doi=10.4081%2Fitjm.2024.1726&pmid=&sid=OVID:embase"] | study design |
| Parenti N, Scalese M, Palazzi C, Agrusta F, Cahill J, Agnelli G. Role of Internal Jugular Vein Ultrasound Measurements in the Assessment of Central Venous Pressure in Spontaneously Breathing Patients: A Systematic Review. Journal of Acute Medicine. 2019;9(2):39–48. | study design |
| Patel PN, Prajapati S, Vaghela GJ. Exploring Non-Invasive Imaging Techniques in Budd-Chiari Syndrome. International Journal of Current Pharmaceutical Review and Research. 2025;17(2):99–102. | study design |
| Phuah Y, Tan YX, Zaghloul S, Sim S, Wong J, Usmani S, et al. A systematic review and meta-analysis of transthoracic echocardiogram vs. cardiac magnetic resonance imaging for the detection of left ventricular thrombus. Eur Heart J Imaging Methods Pract. 2023;1(2):qyad041. Located at: 39045058. doi:10.1093/ehjimp/qyad041 | population |
| Pitasi C, Lamarca F, Vilela V, Vargas-Santos AB, Aschwanden M, Imfeld S, et al. Accuracy of the intima-media thickness by ultrasound for the diagnosis of giant cell arteritis: a systematic review. Semin Arthritis Rheum. 2025;73:152725. Located at: 40273742. doi:10.1016/j.semarthrit.2025.152725 | outcome measures |
| Polena V, Huchon C, Ramos CV, Rouzier R, Dumont A, Fauconnier A. Non-Invasive Tools for the Diagnosis of Potentially Life-Threatening Gynaecological Emergencies: A Systematic Review. PLOS ONE. 2015 Feb 27;10(2):e0114189. doi:10.1371/journal.pone.0114189 | study design |
| Pomero F, Dentali F, Borretta V, Bonzini M, Melchio R, Douketis JD, et al. Accuracy of emergency physician-performed ultrasonography in the diagnosis of deep-vein thrombosis: A systematic review and meta-analysis. Thromb Haemost. 2013;109(1):137–45. doi:10.1160/TH12-07-0473 | duplicate |
| Popat A, Harikrishnan S, Seby N, Sen U, Patel SK, Mittal L, et al. Utilization of Point-of-Care Ultrasound as an Imaging Modality in the Emergency Department: A Systematic Review and Meta-Analysis. Cureus. 2024. doi:10.7759/cureus.52371 | duplicate |
| Popat A, Yadav S, Pethe G, Rehman A, Sharma P, Rezkalla S. The role of POCUS in diagnosing acute heart failure in the emergency department: A meta-analysis. J Cardiol. 2025;22:22. Located at: 40555279. doi:10.1016/j.jjcc.2025.06.012 | study design |
| Popat A, Yadav S, Pethe G, Rehman A, Sharma P, Rezkalla S. The role of POCUS in diagnosing acute heart failure in the emergency department: a meta-analysis. Journal of Cardiology. 2025. | reference standard |
| Poulsen E, Aagaard R, Bisgaard J, Sørensen HT, Juhl-Olsen P. The effects of ultrasound guidance on first-attempt success for difficult peripheral intravenous catheterization: a systematic review and meta-analysis. European Journal of Emergency Medicine. 2023 Jan 20. doi:10.1097/MEJ.0000000000000993 | outcome measures |
| Pouncey AL, Yeldham G, Magan T, Lucenteforte E, Jaffer U, Virgili G. Halo sign on temporal artery ultrasound versus temporal artery biopsy for giant cell arteritis. Cochrane Database Syst Rev. 2024;2:CD013199. Located at: 38323659. doi:10.1002/14651858.CD013199.pub2 | study design |
| Querol F, Rodriguez-Merchan EC. The role of ultrasonography in the diagnosis of the musculo-skeletal problems of haemophilia. Haemophilia. 2012;18(3):e215–26. | study design |
| Quinn AC, Sinert R. What is the utility of the Focused Assessment with Sonography in Trauma (FAST) exam in penetrating torso trauma? Injury. 2011 May;42(5):482–7. doi:10.1016/j.injury.2010.07.249 | study design |
| Radzina M, Biederer J. Ultrasonography of the Lung. RoFo Fortschritte auf dem Gebiet der Rontgenstrahlen und der Bildgebenden Verfahren. 2019;191(1):909–23. | study design |
| Rai Y, You-Ten E, Zasso F, De Castro C, Ye XY, Siddiqui N. The role of ultrasound in front-of-neck access for cricothyroid membrane identification: A systematic review. Journal of Critical Care. 2020;60:161–8. | outcome measures |
| Raine-Fenning N, Hopkisson J. Management of ectopic pregnancy: a clinical approach. Obstetrics, Gynaecology and Reproductive Medicine. 2009;19(1):19–25. | study design |
| Ransome W, Dizon J, Chau S, Guerrero K. Diagnostic accuracy of imaging modalities for adultswith acute pulmonary embolism: systematic review andmeta-analysis. J of Medical Radiation Sci. 2022 May;69(S1):93. doi:10.1002/jmrs.587 | conference abstract |
| Ransome WJ, Dizon J, Guerrero K, Chau M. Diagnostic test accuracy of imaging modalities for adults with acute pulmonary embolism: A systematic review and meta-analysis. Journal of Medical Imaging and Radiation Sciences. 2023 Mar;54(1):178–94. doi:10.1016/j.jmir.2022.11.003 | study design |
| Razakh A, Uzor A, Htoon A, Valko M. Utilizing POCUS in the diagnosis of small bowel obstruction and the barriers to its implementation in resource-limited settings: a systematic review. Journal of Ultrasound. 2026;29(1):43–56. doi:10.1007/s40477-025-01110-y | study design |
| Rehman M, Duarte Silva F, Chhabra A. Diagnostic efficacy of posterior tibialis tendon dysfunction: a systematic review of literature. Eur Radiol. 2024;34(5):3513–23. Located at: 37889271. doi:10.1007/s00330-023-10364-1 | study design |
| Reiman MP, Thorborg K, Goode AP, Cook CE, Weir A, Hölmich P. Diagnostic Accuracy of Imaging Modalities and Injection Techniques for the Diagnosis of Femoroacetabular Impingement/Labral Tear: A Systematic Review With Meta-analysis. Am J Sports Med. 2017 Sep;45(11):2665–77. doi:10.1177/0363546516686960 | setting |
| Reynolds JC, Nicholson T, O’Neil B, Drennan IR, Issa M, Welsford M. Diagnostic test accuracy of point-of-care ultrasound during cardiopulmonary resuscitation to indicate the etiology of cardiac arrest: A systematic review. Resuscitation. 2022;172:54–63. doi:10.1016/j.resuscitation.2022.01.006 | study design |
| Richardson A, Gallos I, Dobson S, Campbell BK, Coomarasamy A, Raine-Fenning N. Accuracy of first-trimester ultrasound in diagnosis of intrauterine pregnancy prior to visualization of the yolk sac: a systematic review and meta-analysis. Ultrasound in Obstetrics & Gynecology. 2015;46(2):142–9. doi:10.1002/uog.14725 | duplicate |
| Richardson L, Loubani O, Atkinson P. MP05: Diagnostic accuracy of point of care ultrasound in undifferentiated hypotension presenting to the emergency department: a systematic review. CJEM. 2019;21(S1):S43–4. Located at: Cambridge Core. doi:10.1017/cem.2019.140 | conference abstract |
| Rinagel M, Chatelus E, Jousse-Joulin S, Sibilia J, Gottenberg JE, Chasset F, et al. Diagnostic performance of temporal artery ultrasound for the diagnosis of giant cell arteritis: a systematic review and meta-analysis of the literature. Autoimmun Rev. 2019;18(1):56–61. doi:10.1016/j.autrev.2018.07.012 | duplicate |
| Robba C, Santori G, Czosnyka M, Corradi F, Bragazzi N, Padayachy L, et al. Optic nerve sheath diameter measured sonographically as non-invasive estimator of intracranial pressure: a systematic review and meta-analysis. Intensive Care Med. 2018 Aug;44(8):1284–94. doi:10.1007/s00134-018-5305-7 | outcome measures |
| Robba C, Santori G, Czosnyka M, Corradi F, Bragazzi N, Padayachy L, et al. Optic nerve sheath diameter measured sonographically as non-invasive estimator of intracranial pressure: a systematic review and meta-analysis. Intensive Care Med. 2018;44(8):1284–94. doi:10.1007/s00134-018-5305-7 | duplicate |
| Robinson A, Light D, Kasim A, Nice C. A systematic review and meta-analysis of the role of radiology in the diagnosis of occult inguinal hernia. Surg Endosc. 2013 Jan;27(1):11–8. doi:10.1007/s00464-012-2412-3 | reference standard |
| Robinson A, Light D, Nice C. Meta-analysis of Sonography in the Diagnosis of Inguinal Hernias. Journal of Ultrasound in Medicine. 2013 Feb;32(2):339–46. doi:10.7863/jum.2013.32.2.339 | outcome measures |
| Roldan M, Abay TY, Kyriacou PA. Non-Invasive Techniques for Multimodal Monitoring in Traumatic Brain Injury: Systematic Review and Meta-Analysis. Journal of Neurotrauma. 2020;37(23):2445–53. | study design |
| Roll SC, Case-Smith J, Evans KD. Diagnostic accuracy of ultrasonography vs. electromyography in carpal tunnel syndrome: a systematic review of literature. Ultrasound in Medicine & Biology. 2011;37(10):1539–53. | study design |
| Romosan G, Valentin L. The sensitivity and specificity of transvaginal ultrasound with regard to acute pelvic inflammatory disease: a review of the literature. Arch Gynecol Obstet. 2014 Apr 1;289(4):705–14. doi:10.1007/s00404-013-3091-6 | study design |
| Ron E, Alattar Z, Hoebee S, Kang P, vanSonnenberg E. Current Trends in the Use of Ultrasound Over Chest X-Ray to Identify Pneumothoraces in ICU, Trauma, and ARDS Patients. J Intensive Care Med. 2022 Jan;37(1):5–11. doi:10.1177/0885066620987813 | study design |
| Roth D, Pace NL, Lee A, Hovhannisyan K, Warenits AM, Arrich J, et al. Bedside tests for predicting difficult airways: an abridged Cochrane diagnostic test accuracy systematic review. Anaesthesia. 2019;74(7):915–28. doi:10.1111/anae.14608 | index test |
| Rubano E, Caputo W, Mehta N, Paladino N, Sinert R. Systematic review: Emergency department bedside ultrasonography for diagnosing an abdominal aortic aneurysm. Academic Emergency Medicine. 2011 May;18(s1):S29-30. doi:10.1111/j.1553-2712.2011.01073.x | conference abstract |
| Rubano E, Mehta N, Caputo W, Paladino L, Sinert R. Systematic review: Emergency department bedside ultrasonography for diagnosing suspected abdominal aortic aneurysm. Acad Emerg Med. 2013;20(2):128–38. doi:10.1111/acem.12080 | duplicate |
| Ruddox V, Mathisen M, Baekkevar M, Aune E, Edvardsen T, Otterstad JE. Is 3D echocardiography superior to 2D echocardiography in general practice? A systematic review of studies published between 2007 and 2012. International Journal of Cardiology. 2013;168(2):1306–15. | setting |
| Saji SZ, Murga O, Khurana S, Hung Phan B, Khalil B, Nagra AM, et al. Utilization of left ventricular outflow tract velocity time integral in the assessment of fluid responsiveness in adult patients with sepsis or septic shock - a systematic review. J. 2025;23:23. Located at: 40849404. doi:10.1007/s40477-025-01072-1 | outcome measures |
| Sakellariou G, Scirè CA, Adinolfi A, Batticciotto A, Bortoluzzi A, Delle Sedie A, et al. Differential Diagnosis of Inflammatory Arthropathies by Musculoskeletal Ultrasonography: A Systematic Literature Review. Front Med. 2020 May 7;7:141. doi:10.3389/fmed.2020.00141 | outcome measures |
| Sakellariou G, Scire CA, Adinolfi A, Batticciotto A, Bortoluzzi A, Delle Sedie A, et al. Differential Diagnosis of Inflammatory Arthropathies by Musculoskeletal Ultrasonography: A Systematic Literature Review. Frontiers in Medicine. 2020;7:141. | study design |
| Salameh JP, Leeflang MM, Hooft L, Islam N, McGrath TA, van der Pol CB, et al. Thoracic imaging tests for the diagnosis of COVID-19. Cochrane Database Syst Rev. 2020;9:CD013639. Located at: 32997361. doi:10.1002/14651858.CD013639.pub2 | duplicate |
| Scarvelis D, Wells PS. Diagnosis and treatment of deep-vein thrombosis. CMAJ Canadian Medical Association Journal. 1087;175(9):1087–92. | study design |
| Schneiders AG, Sullivan SJ, Hendrick PA, Hones BDGM, McMaster AR, Sugden BA, et al. The Ability of Clinical Tests to Diagnose Stress Fractures: A Systematic Review and Meta-analysis. Journal of Orthopaedic & Sports Physical Therapy. 2012 Sep;42(9):760–71. doi:10.2519/jospt.2012.4000 | study design |
| Shaban E, Yiğit Y, Alkahlout B, Shaban A, Shaban A, Ponappan B, et al. Enhancing clinical outcomes: Point of care ultrasound in the precision diagnosis and Management of Abdominal Aortic Aneurysms in emergency medicine: A systematic review and meta‐analysis. Journal of Clinical Ultrasound. 2024. doi:10.1002/jcu.23850 | duplicate |
| Shaban EE, Yigit Y, Ponappan B, Shaban A, Ahmed MH, Abdelaal YO, et al. Evaluating the Role of Point-of-Care Ultrasound in Central Venous Pressure Monitoring for Critically Ill Patients. A Comprehensive Systematic Review and Meta-analysis. Ultrasound Med Biol. 2025;21:21. Located at: 40404525. doi:10.1016/j.ultrasmedbio.2025.04.008 | setting |
| Shah RG, Novaro GM, Blandon RJ, Whiteman MS, Asher CR, Kirsch J. Aortic valve area: Meta-analysis of diagnostic performance of multi-detector computed tomography for aortic valve area measurements as compared to transthoracic echocardiography. International Journal of Cardiovascular Imaging. 2009;25(6):601–9. | index test |
| Shah VP, Oliveira J. E Silva L, Farah W, Seisa M, Kara Balla A, Christensen A, et al. Diagnostic accuracy of neuroimaging in emergency department patients with acute vertigo or dizziness: A systematic review and meta‐analysis for the guidelines for reasonable and appropriate care in the emergency department. Academic Emergency Medicine. 2023 May;30(5):517–30. doi:10.1111/acem.14561 | outcome measures |
| Shea JA, Berlin JA, Escarce JJ, Clarke JR, Kinosian BP, Cabana MD, et al. Revised estimates of diagnostic test sensitivity and specificity in suspected biliary tract disease. Archives of internal medicine. 1994;154(22):2573–81. | study design |
| Sheraton M, Snitzer J, Guttman J. 226 Transthoracic Echocardiography for Diagnosing AorticDissection in the Emergency Department: A Systematic Review. Academic Emergency Medicine. 2023 May;30(S1):112–3. doi:10.1111/acem.14718 | conference abstract |
| Shokoohi H, Peksa GD, Hutchison A, Al Jalbout N, Montoya K, Westrick JC, et al. Ultrasound accuracy in acute diverticulitis: A systematic review and Meta-analysis. Am J Emerg Med. 2025;92:96–103. Located at: 40088707. doi:10.1016/j.ajem.2025.03.002 | study design |
| Shook SJ, Ginsberg M, Narayanaswami P, Beekman R, Dubin AH, Katirji B, et al. Evidence-based guideline: Neuromuscular ultrasound for the diagnosis of ulnar neuropathy at the elbow. Muscle & Nerve. 2022;65(2):147–53. | outcome measures |
| Shujaa AS, Buhumaid R, Alabdulkareem NF, Alharbi MS, Sulaiman NAB, Alotabi LT. The Use of Point-of-Care Ultrasound (POCUS) in Non-Trauma Cardiac Arrest: A Systematic Review and Meta-Analysis of Diagnostic Accuracy and Therapeutic Impact. SJEM. 2026. doi:10.24911/sjemed.72-1751851270 | outcome measures |
| Sinert R, Martin J, Abdel-Naby R, Mehta N. Systematic review: Emergency department bedside renal ultrasonography for diagnosing nephrolithiasis. European Journal of Emergency Medicine. 2011;18(5):301–301. doi:https://dx.doi.org/10.1097/01.mej.0000405160.99362.7c | conference abstract |
| Sivera F, Andres M, Falzon L, van der Heijde DM, Carmona L. Diagnostic value of clinical, laboratory, and imaging findings in patients with a clinical suspicion of gout: a systematic literature review. Journal of Rheumatology - Supplement. 2014;92:3–8. | outcome measures |
| Sivera F, Andres M, Falzon L, Van Der Heijde DMFM, Carmona L. Diagnostic Value of Clinical, Laboratory, and Imaging Findings in Patients with a Clinical Suspicion of Gout: A Systematic Literature Review. The Journal of Rheumatology Supplement. 2014 Sep 1;92(0):3–8. doi:10.3899/jrheum.140456 | study design |
| Smith S. Gangrenous cholecystitis: Does contrast-enhanced ultrasound improve diagnostic accuracy? A systematic review and meta-analysis. Ultrasound. 2019 May;27(2):NP32-33. doi:10.1177/1742271X19840271 | conference abstract |
| Song J, Jiang H, Wang X, Gong K. Evolution of the Diagnostic Paradigm for Giant Cell Arteritis: From Histopathology to Multimodal Imaging Integration. Journal of Rheumatology. 2026. doi:10.3899/jrheum.2025-1205 | study design |
| Søreide K, Weiser TG, Parks RW. Clinical update on management of pancreatic trauma. HPB. 2018 Dec;20(12):1099–108. doi:10.1016/j.hpb.2018.05.009 | study design |
| Sotoodehnia M, Rafiemanesh H, Mirfazaelian H, Safaie A, Baratloo A. Ultrasonography indicators for predicting difficult intubation: a systematic review and meta-analysis. BMC Emerg Med. 2021;21(1). doi:10.1186/s12873-021-00472-w | outcome measures |
| Squizzato A, Rancan E, Dentali F, Bonzini M, Guasti L, Steidl L, et al. Diagnostic accuracy of lung ultrasound for pulmonary embolism: a systematic review and meta‐analysis. Journal of Thrombosis and Haemostasis. 2013. doi:10.1111/jth.12232 | duplicate |
| Staub LJ, Mazzali Biscaro RR, Kaszubowski E, Maurici R. Lung Ultrasound for the Emergency Diagnosis of Pneumonia, Acute Heart Failure, and Exacerbations of Chronic Obstructive Pulmonary Disease/Asthma in Adults: A Systematic Review and Meta-analysis. J Emerg Med. 2019;56(1):53–69. doi:10.1016/j.jemermed.2018.09.009 | duplicate |
| Stengel D, Bauwens K, Porzsolt F, Rademacher G, Mutze S, Ekkernkamp A. Emergency Ultrasound for Blunt Abdominal Trauma - Meta-analysis Update 2003. Zentralblatt fur Chirurgie. 2003;128(12):1027–37. doi:https://dx.doi.org/10.1055/s-2003-44850 | outcome measures |
| Stewart S, Su I, Gamble GD, Dalbeth N. Diagnostic value of different imaging features for patients with suspected gout: A network meta-analysis. Seminars in Arthritis & Rheumatism. 2021;51(6):1251–7. | duplicate |
| Stickles SP, Carpenter CR, Gekle R, Kraus C, Theodoro D, Scoville C, et al. The diagnostic accuracy of the rapid ultrasound in shock (RUSH) exam for shock etiology: A systematic review and meta-analysis. Academic Emergency Medicine. 2018 May;25(S1):p59. doi:10.1111/acem.13424 | conference abstract |
| Stickles SP, Carpenter CR, Gekle R, Kraus CK, Scoville C, Theodoro D, et al. The diagnostic accuracy of a point-of-care ultrasound protocol for shock etiology: A systematic review and meta-analysis. Can J Emerg Med. 2019;21(3):406–17. doi:10.1017/cem.2018.498 | duplicate |
| Stoianov AG, Patrascu JM, Hogea BG, Andor B, Florescu S, Misca LC, et al. Point-of-Care Ultrasound for the Evaluation and Management of Posterior Cruciate Ligament Injuries: A Systematic Review. Diagnostics. 2023 Jul 12;13(14):2352. doi:10.3390/diagnostics13142352 | study design |
| Storti M, Musella L, Cianci V. [Nurse-performed FAST ultrasound in the emergency department: a systematic review]. Professioni infermieristiche. 2013;66(1):5–16. doi:https://dx.doi.org/10.7429/pi.2013.661005 | language |
| Strauss SA, Ma GW, Seo C, Siracuse JJ, Madassery S, Truesdell AG, et al. Ultrasound-guided versus anatomic landmark-guided percutaneous femoral artery access. Cochrane Database Syst Rev. 2025;3:CD014594. Located at: 40152297. doi:10.1002/14651858.CD014594.pub2 | outcome measures |
| Strøm JJ, Haugen PS, Hansen MP, Graumann O, Jensen MBB, Aakjær Andersen C. Accuracy of lung ultrasonography in the hands of non-imaging specialists to diagnose and assess the severity of community-acquired pneumonia in adults: a systematic review. BMJ Open. 2020 Jun;10(6):e036067. doi:10.1136/bmjopen-2019-036067 | study design |
| Stubbings N, Smith T. Diagnostic test accuracy of clinical and radiological assessments for medial patella plica syndrome: A systematic review and meta-analysis. The Knee. 2014 Mar;21(2):486–90. doi:10.1016/j.knee.2013.11.001 | study design |
| Subramaniam S, Bober J, Chao J, Zehtabchi S. Point‐of‐care Ultrasound for Diagnosis of Abscess in Skin and Soft Tissue Infections. Carpenter CR, editor. Academic Emergency Medicine. 2016 Nov;23(11):1298–306. doi:10.1111/acem.13049 | population |
| Sultan SR, Bashmail FT, Alzahrani NA, Alharbi SI, Anbar R, Alkharaiji M. Contrast-enhanced ultrasound for the evaluation of symptomatic and asymptomatic carotid plaques: A systematic review and meta-analysis. Echocardiography. 1032;39(8):1032–43. | outcome measures |
| Taheri O, Samain J, Mauny F, Puyraveau M, Desmettre T, Marx T. Contribution of point-of-care ultrasound in the prehospital management of patients with non-trauma acute dyspnea: a systematic review and meta-analysis. Eur J Emerg Med. 2025;32(2):87–99. Located at: 39630617. doi:10.1097/MEJ.0000000000001205 | outcome measures |
| Tang S, Xiang C, Yang Q. Ultrasound and MRI in diagnosis of appendicitis in pregnancy: Meta-analysis. Chinese Journal of Medical Imaging Technology. 2021;37(3):411–5. doi:https://dx.doi.org/10.13929/j.issn.1003-3289.2021.03.023 | language |
| Tenuta M, Sesti F, Bonaventura I, Mazzotta P, Pofi R, Gianfrilli D, et al. Use of contrast enhanced ultrasound in testicular diseases: A comprehensive review. Andrology. 1369;9(5):1369–82. | study design |
| Thomas F, Flint N, Setareh-Shenas S, Rader F, Kobal SL, Siegel RJ. Accuracy and Efficacy of Hand-Held Echocardiography in Diagnosing Valve Disease: A Systematic Review. American Journal of Medicine. 2018;131(10):1155–60. | study design |
| Thomas F, Flint N, Setareh-Shenas S, Rader F, Kobal SL, Siegel RJ. Accuracy and Efficacy of Hand-Held Echocardiography in Diagnosing Valve Disease: A Systematic Review. The American Journal of Medicine. 2018 Oct;131(10):1155–60. doi:10.1016/j.amjmed.2018.04.043 | index test |
| Tian H, Zhang T, Zhou Y, Rastogi S, Choudhury R, Iqbal J. Role of emergency chest ultrasound in traumatic pneumothorax. An updated meta-analysis. Med Ultrason. 2023 Mar 30;25(1):66. doi:10.11152/mu-3309 | study design |
| Tsou PY, Wang YH, Ma YK, Deanehan JK, Gillon J, Chou EH, et al. Accuracy of point-of-care ultrasound and radiology-performed ultrasound for intussusception: A systematic review and meta-analysis. The American Journal of Emergency Medicine. 2019;37(9):1760–9. doi:10.1016/j.ajem.2019.06.006 | population |
| Tsujimoto Y, Kataoka Y, Banno M, Anan K, Shiroshita A, Jujo S. Ultrasonography for confirmation of gastric tube placement. Cochrane Database Syst Rev. 2024;7:CD012083. Located at: 39051488. doi:10.1002/14651858.CD012083.pub3 | study design |
| Ullah N, Bacha R, Manzoor I, Gilani SA, Gilani SMYF, Haider Z. Reliability of Focused Assessment With Sonography for Trauma in the Diagnosis of Blunt Torso Trauma. Journal of Diagnostic Medical Sonography. 2022 Jan;38(1):60–70. doi:10.1177/87564793211029849 | study design |
| Unal D, Hazır MS, Ambarcıoglu P. No: 8144 Airway ultrasound assessment of the pre-epiglottic distance to epiglottis-vocal cords distance ratio for the prediction of difficult laryngoscopy: a diagnostic test accuracy meta analysis. Trends in Anaesthesia and Critical Care. 2023 Feb 1;48:S15–6. doi:10.1016/S2210-8440(23)00040-0 | conference abstract |
| Van Der Geest KSM, Sandovici M, Brouwer E, Mackie SL. Diagnostic Accuracy of Symptoms, Physical Signs, and Laboratory Tests for Giant Cell Arteritis: A Systematic Review and Meta-analysis. JAMA Intern Med. 2020;180(10):1295–304. doi:10.1001/jamainternmed.2020.3050 | index test |
| van der Ham DP, van Teeffelen ASP, Mol BWJ. Prelabour rupture of membranes: overview of diagnostic methods. Current Opinion in Obstetrics and Gynecology. 2012 Dec;24(6):408. doi:10.1097/GCO.0b013e328359825c | study design |
| Van Hoving DJ, Griesel R, Meintjes G, Takwoingi Y, Maartens G, Ochodo EA. Abdominal ultrasound for diagnosing abdominal tuberculosis or disseminated tuberculosis with abdominal involvement in HIV-positive individuals. Cochrane Infectious Diseases Group, editor. Cochrane Database of Systematic Reviews. 2019 Sep 30;2019(9). doi:10.1002/14651858.CD012777.pub2 | population |
| Vannucci A, Cavallone LF. Bedside predictors of difficult intubation: A systematic review. Minerva Anestesiol. 2016;82(1):69–83. | outcome measures |
| Varndell W, Topacio M, Hagness C, Lemon H, Tracy D. Nurse-performed focused ultrasound in the emergency department: a systematic review. Australasian Emergency Care. 2018;21(4):121–30. | study design |
| Varonen H, Mäkelä M, Savolainen S, Läärä E, Hilden J. Comparison of ultrasound, radiography, and clinical examination in the diagnosis of acute maxillary sinusitis: a systematic review. | study design |
| Vetsheva NN, Reshetnikov RV, Leonov DV, Kulberg NS, Mokienko OA. Diagnostic value of lung ultrasound in COVID-19: systematic review and meta-analysis. Digit Diagn. 2020;1(1):13–26. doi:10.17816/DD46834 | language |
| Vrablik ME, Snead GR, Minnigan HJ, Kirschner JM, Emmett TW, Seupaul RA. The diagnostic accuracy of bedside ocular ultrasonography for the diagnosis of retinal detachment: A systematic review and meta-analysis. Ann Emerg Med. 2015;65(2):199-203.e1. doi:10.1016/j.annemergmed.2014.02.020 | outcome measures |
| Vredeveld T, van Benten E, Beekmans R, Koops MP, Ket JCF, Mollema J, et al. Reliability and validity of assessment methods available in primary care for bladder outlet obstruction and benign prostatic obstruction in men with lower urinary tract symptoms: a systematic review. BMJ Open. 2022;12(4):e056234. | outcome measures |
| Waitayangkoon P, Weilg-Espejo P, Kissin EY. Periosteal Manifestations of Osteomyelitis and Arthritis on Ultrasound: A Systematic Review. Journal of Medical Ultrasound. 2023 Sep 2. doi:10.4103/jmu.jmu_16_23 | study design |
| Wakrim S, Ziouziou I, Ralph D, Khabbal Y. Penile Doppler ultrasound study in priapism: A systematic review. Progrès en Urologie. 2022 Jan;32(1):61–9. doi:10.1016/j.purol.2021.03.009 | study design |
| Walker SCD, Lipszyc AC, Kilmurray M, Wilding H, Akhlaghi H. Questioning the Role of Carotid Artery Ultrasound in Assessing Fluid Responsiveness in Critical Illness: A Systematic Review and Meta-Analysis. Crit Care Res Pract. 2024;2024:9102961. Located at: 38716052. doi:10.1155/2024/9102961 | setting |
| Wang J, Wu H, Dong F, Li B, Wei Z, Peng Q, et al. The role of ultrasonography in the diagnosis of anterior cruciate ligament injury: A systematic review and meta‐analysis. European Journal of Sport Science. 2018 May;18(4):579–86. doi:10.1080/17461391.2018.1436196 | study design |
| Wang L, Wang M, Zhou Q. Diagnostic value of ultrasound for fracture of the distal radius and ulna: A systematic review. Chinese Journal of Evidence-Based Medicine. 2017;17(2):213–20. doi:https://dx.doi.org/10.7507/1672-2531.201611013 | language |
| Wang MK, Piticaru J, Kappel C, Mikhaeil M, Mbuagbaw L, Rochwerg B. Internal jugular vein ultrasound for the diagnosis of hypovolemia and hypervolemia in acutely ill adults: a systematic review and meta-analysis. Intern Emerg Med. 2022 Aug;17(5):1521–32. doi:10.1007/s11739-022-03003-y | reference standard |
| Wang X, Yang L, Liu X, Yao N, Dai Y, Lu R. Ultrasonic measurement of adult optic nerve sheath diameter for diagnosis of intracranial hypertension: Meta-analysis. Chinese Journal of Medical Imaging Technology. 1464;38(10):1464–9. Located at: 2020890629. doi:10.13929/j.issn.1003-3289.2022.10.005 | language |
| Wang X, Yang L, Liu X, Yao N, Dai Y, Lu R. Ultrasonic measurement of adult optic nerve sheath diameter for diagnosis of intracranial hypertension: Meta-analysis. Chinese Journal of Medical Imaging Technology. 2022;38(10):1464–9. doi:https://dx.doi.org/10.13929/j.issn.1003-3289.2022.10.005 | language |
| Wang Y, Li N, Qu Y. Diagnostic accuracy of lung ultrasound for transient tachypnea: a meta-analysis. Jornal de Pediatria. 2022 Jul;98(4):329–37. doi:10.1016/j.jped.2021.10.003 | population |
| Wang Y, Nie F, Wang T, Li Y, Xu P. Systematic reviews of the accuracy of musculoskeletal ultrasound in the diagnosis of lateral epicondylalgia. Chinese Journal of Tissue Engineering Research. 2019;23(32):5233–9. doi:https://dx.doi.org/10.3969/j.issn.2095-4344.1263 | language |
| Weston AR, Jackson TJ, Blamey S. Diagnosis of appendicitis in adults by ultrasonography or computed tomography: A systematic review and meta-analysis. Int J Technol Assess Health Care. 2005;21(3):368–79. doi:10.1017/S0266462305050488 | duplicate |
| Wetterslev M, Haase N, Johansen RR, Perner A. Predicting fluid responsiveness with transthoracic echocardiography is not yet evidence based. Acta Anaesthesiol Scand. 2013 Jul;57(6):692–7. doi:10.1111/aas.12045 | study design |
| Wilson S, Thavanathan R, Cheng W, Stuart J, Kim D, Shorr R, et al. PO009 Test characteristics of point-of-care ultrasound for the diagnosis  of acute cholecystitis: a systematic review and meta-analysis. Can J Emerg Med. 2023 May 1;25(1):S52. doi:10.1007/s43678-023-00508-z | conference abstract |
| Wilson SJ, Thavanathan R, Cheng W, Stuart J, Kim DJ, Glen P, et al. Test Characteristics of Emergency Medicine-Performed Point-of-Care Ultrasound for the Diagnosis of Acute Cholecystitis: A Systematic Review and Meta-analysis. Ann Emerg Med. 2024;83(3):235–46. Located at: 37855790. doi:10.1016/j.annemergmed.2023.09.005 | duplicate |
| Winkler MH, Touw HR, van de Ven PM, Twisk J, Tuinman PR. Diagnostic Accuracy of Chest Radiograph, and When Concomitantly Studied Lung Ultrasound, in Critically Ill Patients With Respiratory Symptoms: A Systematic Review and Meta-Analysis. Crit Care Med. 2018;46(7):e707–14. doi:10.1097/CCM.0000000000003129 | population |
| Wong C, Teitge BD, Ross M, Young P, Robertson H, Lang E. The Accuracy and Prognostic Value of Point‐of‐care Ultrasound for Nephrolithiasis in the Emergency Department: A Systematic Review and Meta‐analysis. Academic Emergency Medicine. 2018. doi:10.1111/acem.13388 | duplicate |
| Wu C, Zheng Z, Jiang L, Gao Y, Xu J, Jin X, et al. The predictive value of bedside ultrasound to restore spontaneous circulation in patients with pulseless electrical activity: A systematic review and meta-analysis. Erdoes G, editor. PLoS ONE. 2018 Jan 24;13(1):e0191636. doi:10.1371/journal.pone.0191636 | outcome measures |
| Wu C, Zheng Z, Jiang L, Gao Y, Xu J, Jin X, et al. The predictive value of bedside ultrasound to restore spontaneous circulation in patients with pulseless electrical activity: A systematic review and meta-analysis. PLoS ONE [Internet]. 2018;13(1). Available from: ["https://ovidsp.ovid.com/ovidweb.cgi?T=JS&CSC=Y&NEWS=N&PAGE=fulltext&D=emed19&DO=10.1371%2fjournal.pone.0191636", "https://libkey.io/libraries/528/openurl?genre=article&aulast=Wu&issn=1932-6203&title=PLoS+ONE&atitle=The+predictive+value+of+bedside+ultrasound+to+restore+spontaneous+circulation+in+patients+with+pulseless+electrical+activity%3A+A+systematic+review+and+meta-analysis&volume=13&issue=1&spage=e0191636&epage=&date=2018&doi=10.1371%2Fjournal.pone.0191636&pmid=29364925&sid=OVID:embase"] | outcome measures |
| Wu C, Zheng Z, Jiang L, Gao Y, Xu J, Jin X, et al. The predictive value of bedside ultrasound to restore spontaneous circulation in patients with pulseless electrical activity: A systematic review and meta-analysis. PLoS ONE. 2018;13(1). doi:10.1371/journal.pone.0191636 | duplicate |
| Wu X, Li K, Kou S, Zhang Z. The Accuracy of Point-of-Care Ultrasound in the Detection of Gallbladder Disease: A Meta-analysis. Acad Radiol. 2024;31(4):1336–43. Located at: 37838525. doi:10.1016/j.acra.2023.09.029 | duplicate |
| Xia Y, Ying Y, Wang S, Li W, Shen H. Effectiveness of lung ultrasonography for diagnosis of pneumonia in adults: A systematic review and meta-analysis. J Thorac Dis. 2016;8(10):2822–31. doi:10.21037/jtd.2016.09.38 | duplicate |
| Xingxing H, Tianyi C, Xiaolong Y. Non-invasive Assessment of Rheumatoid Arthritis Cardiac Involvement: A Systematic Review of Echocardiography. Current Medical Imaging [Internet]. 2025;21. Available from: ["https://ovidsp.ovid.com/ovidweb.cgi?T=JS&CSC=Y&NEWS=N&PAGE=fulltext&D=emexb&DO=10.2174%2f0115734056375528250701165335", "https://libkey.io/libraries/528/openurl?genre=article&aulast=Xingxing&issn=1573-4056&title=Current+Medical+Imaging&atitle=Non-invasive+Assessment+of+Rheumatoid+Arthritis+Cardiac+Involvement%3A+A+Systematic+Review+of+Echocardiography&volume=21&issue=&spage=E15734056375528&epage=&date=2025&doi=10.2174%2F0115734056375528250701165335&pmid=40660436&sid=OVID:embase"] | study design |
| Xiong X, Xiong Y, Liu G. Systematic review and meta-analysis: safety of ultrasound-guided peripheral venipuncture and catheterization. Annals of Palliative Medicine. 2021;10(11):11721–32. | study design |
| Yan T, Xie W, Xu M. Effect of chest ultrasound compared with pericardial window for the diagnosis of occult penetrating cardiac wounds in hemodynamically stable subjects with penetrating thoracic trauma: A meta-analysis. International Wound Journal. 2023;20(6):2483–91. | retracted |
| Yang J, Huang J, Zhang Y, Zeng K, Liao M, Jiang Z, et al. Contrast-enhanced ultrasound and contrast-enhanced computed tomography for differentiating mass-forming pancreatitis from pancreatic ductal adenocarcinoma: a meta-analysis. Chinese Medical Journal. 2023;136(17):2028–36. | population |
| Yang Q, Zhao F, Qi J, Su L. The comparison of accuracy and practicability between ultrasound and spiral CT in the diagnosis of intestinal obstruction: A protocol for systematic review and meta-analysis. Medicine. 2021 Jan 29;100(4):e23631. doi:10.1097/MD.0000000000023631 | study design |
| Yefei H, Jiayong O, Jieruo G. Diagnostic value of color doppler ultrasonic for gouty arthritis in China : A meta-analysis. International Journal of Rheumatic Diseases. 2017;19th Asia Pacific League of Associations for Rheumatology Congress, APLAR 2017. Dubai .20:71. doi:https://dx.doi.org/10.1111/1756-185X.13178 PT - Conference Abstract | conference abstract |
| Yinghao P, Yang Y, Ying F, Shuyin H, Jiang Z, Hua J, et al. Diagnostic accuracy of artery peak velocity variation measured by bedside real-time ultrasound for prediction of fluid responsiveness: A Meta-analysis. Chinese Critical Care Medicine. 2020;32(1):99–105. doi:https://dx.doi.org/10.3760/cma.j.cn121430-20191213-00018 | language |
| Yoshida T, Yoshida T, Noma H, Nomura T, Suzuki A, Mihara T. Diagnostic accuracy of point-of-care ultrasound for shock: a systematic review and meta-analysis. Crit Care. 2023;27(1). doi:10.1186/s13054-023-04495-6 | duplicate |
| Yuan X, Liu L, Chang W, Wu Z, Huang L, Chao Y, et al. Diagnosis Accuracy of Lung Ultrasound for ARF in Critically Ill Patients: A Systematic Review and Meta-Analysis. Front Med. 2021;8. doi:10.3389/fmed.2021.705960 | duplicate |
| Yuen. The utility of ultrasonography in the diagnosis of testicular torsion-A systematic review. Ultrasound. 2020 May;28(2):NP1–47. doi:10.1177/1742271X20912390 | conference abstract |
| Zabotti A, Bandinelli F, Batticciotto A, Scire CA, Iagnocco A, Sakellariou G. Musculoskeletal ultrasonography for psoriatic arthritis and psoriasis patients: a systematic literature review. Rheumatology. 2017;56(9):1518–32. | study design |
| Zaki HA, Albaroudi B, Shaban EE, Elgassim M, Almarri ND, Basharat K, et al. Deep venous thrombosis (DVT) diagnostics: gleaning insights from point-of-care ultrasound (PoCUS) techniques in emergencies: a systematic review and meta-analysis. The Ultrasound Journal. 2024;16(1):37. | duplicate |
| Zaki HA, Ponappan B, Shaban EE, Shaban A, Shaban A, Khan A, et al. Enhancing rapid diagnosis of pulmonary embolism in the emergency department: the critical role of point-of-care ultrasound (POCUS)—a systematic review and meta-analysis. Egyptian Journal of Radiology and Nuclear Medicine. 2025;56(1):1–17. | duplicate |
| Zamudio Burbano M, Castro Berrío F, Prada Escobar D. Ultrasound identification of the cricothyroid membrane. Systematic review and meta-analysis. Colomb J Anesthesiol. 2023 May 11;51(3). doi:10.5554/22562087.e1071 | outcome measures |
| Zawadka M, La Via L, Wong A, Olusanya O, Muscarà L, Continella C, et al. Real-Time Ultrasound Guidance as Compared With Landmark Technique for Subclavian Central Venous Cannulation: A Systematic Review and Meta-Analysis With Trial Sequential Analysis*. Critical Care Medicine. 2023 May;51(5):642–52. doi:10.1097/CCM.0000000000005819 | outcome measures |
| Zhang Q, Gao F, Sun W, Ma J, Cheng L, Li Z. The diagnostic performance of musculoskeletal ultrasound in gout: A systematic review and meta-analysis. PLoS ONE. 2018;13(7). doi:10.1371/journal.pone.0199672 | duplicate |
| Zhang Y, Xia H, Wang Y, Chen L, Li S, Hussein IA, et al. The rate of missed diagnosis of lower-limb DVT by ultrasound amounts to 50% or so in patients without symptoms of DVT: A meta-analysis. Medicine. 2019 Sep;98(37):e17103. doi:10.1097/MD.0000000000017103 | setting |
| Zhang Z, Xu X, Ye S, Xu L. Ultrasonographic Measurement of the Respiratory Variation in the Inferior Vena Cava Diameter Is Predictive of Fluid Responsiveness in Critically Ill Patients: Systematic Review and Meta-analysis. Ultrasound in Medicine & Biology. 2014 May;40(5):845–53. doi:10.1016/j.ultrasmedbio.2013.12.010 | setting |
| Zheng Y, Zhou HJ, Tao N, Tian Y, Qin SW, Qin BY, et al. Prevention of catheter tip malposition with an ultrasound-guided finger-pressure method to block the internal jugular vein during PICC placement: a meta-analysis. Wideochirurgia i Inne Techniki Maloinwazyjne. 2022;17(2):289–98. | study design |
| Zhou J, Shan Y, Hu P. A systematic review and meta-analysis on transcranial Doppler in diagnosing ischemic cerebrovascular disease. Ann Palliat Med. 2021 Aug;10(8):8963–71. doi:10.21037/apm-21-1759 | setting |
