# Supplementary material for: Diagnostic accuracy of point-of-care ultrasound in adult non-traumatic emergency medicine: an umbrella review
Source: BMC Med. 2026 Jul 25;24:579. doi: 10.1186/s12916-026-05094-2 (PMC13689006; doi:10.1186/s12916-026-05094-2)
Supplement: Supplementary file 3 [file 12916_2026_5094_MOESM3_ESM.docx]

# Supplementary information

## Additional methodological information

### Table S1. Detailed eligibility criteria

|  | **Inclusion criteria** | **Exclusion criteria** |
| --- | --- | --- |
| **Type of studies** | DTA-SR including a MA with a specific research question related to the DTA of a POCUS examination in ED settings | No SR^1^  No DTA of an ultrasound examination with applicability as a POCUS examination in an ED setting  No MA |
| **Population** | DTA-SR focusing on adults (>16 years) including populations containing children as well | DTA-SR that focused primarily on children |
| **US techniques (index test)** | Reviews focusing on the use of conventional ultrasound and/or CEUS | Specialized ultrasound techniques other than CEUS, such as endosonography, elastography |
| **Clinical setting** | *Emergency setting:* The ultrasound examination must be applicable and used within ED (POCUS)  *Patient complaints:* SR addressing acute onset chief complaints or related to an acute intervention (e.g., intubation) where ultrasound is used as a diagnostic tool in the ED setting | *Non-emergency settings:* SR with a strong focus on US use in other settings such as ICU/perioperative  *Chronic conditions:* Reviews focusing on the disease course of chronic or specific diseases that are typically monitored in specialized clinics, e.g., inflammatory bowel diseases  *Cancer diagnostics:* SR that focus on cancer screening, grading and diagnostics |
| **Reference standard** | Accepted reference standard for the condition |  |
| **Outcome measures** | Pooled sensitivity & specificity for the studied condition | If no pooled sensitivity or specificity is presented graphically or numerically, e.g., a SROC curve without a summary estimate due to high heterogeneity |
| **Other** | Publications in English and German | Publications in other languages |

^1^ Criteria suggested by Krnic Martinic et al. (2019)[1] specific research question, ii.) a reproducible search strategy (naming of databases, naming of search platforms/engines, search date and complete search strategy), iii.) reporting of inclusion and exclusion criteria, iv.) presentation of selection/screening methods, v.) critically appraisal and report of the quality/risk of bias of the included studies, vi.) information about data analysis and synthesis that allows the reproducibility of the results.

**Abbreviations:** CEUS = contrast-enhanced ultrasound; DTA = diagnostic test accuracy; ED = emergency department; ICU = intensive care unit; MA = meta-analysis; POCUS = Point-of-Care Ultrasound; SR = systematic review; SROC = summary receiver operating characteristic

### Table S2. Search strategies, A) Ovid MEDLINE and B) Embase

To identify diagnostic test accuracy (DTA) studies on point-of-care ultrasound (POCUS) in emergency settings, a medical information specialist designed and conducted systematic literature searches in MEDLINE (Ovid) and Embase (Ovid).[27]

The search strategy was structured around two main conceptual domains: (i) point-of-care ultrasound, with an emphasis on ultrasound as a diagnostic imaging modality and echocardiography; and (ii) diagnostic performance parameters, including sensitivity, specificity, and accuracy.[31]

An initial search strategy was developed in MEDLINE and iteratively refined, including peer review. The final strategy combined free-text terms, synonyms, acronyms, and controlled vocabulary (MeSH and Emtree terms), and was subsequently translated and adapted for use in Embase.

For the purposes of this umbrella review, a filter for systematic reviews and meta-analyses was applied. This filter was composed of publication types, Emtree terms, and relevant free-text terms. Unwanted study designs were excluded using publication type filters. Studies involving animals only, as well as those primarily addressing oncological or chronic conditions, were excluded using a double-negative filtering approach.

The searches were first conducted in May 2024 and last updated on April 21, 2026. Update records were managed using the Bramer method[32] in EndNote, and de-duplication was performed using Deduklick.[33]

In addition to the systematic database search, supplementary search strategies were employed to identify potentially relevant studies. These included screening of the 50 most relevant records retrieved via Elicit,[34] screening of the 20 most relevant results from Google Scholar (Google Scholar Labs),[35] and backward citation searching of all included studies.

For backward citation searching, all included studies were entered into Scopus to retrieve their reference lists. The resulting records were exported to EndNote and filtered for publication types indicative of evidence synthesis (i.e., systematic reviews or meta-analyses) prior to screening.

These approaches were used to complement the database search and to enhance the comprehensiveness of the search. All records identified through supplementary methods were screened according to the same eligibility criteria as records identified through database searches. Searches conducted in Elicit and Google Scholar were limited to the most relevant results as ranked by the respective algorithms and are therefore not fully reproducible.

No dedicated search for grey literature was conducted. Given that this umbrella review focused on systematic reviews and meta-analyses, which are predominantly published in peer-reviewed journals, grey literature sources were not expected to contribute substantially. However, the potential for publication bias cannot be fully excluded.

The final set of unique records was screened using Covidence.[36]

| **A) Ovid MEDLINE(R) ALL <1946 to April 20, 2026>** |  |
| --- | --- |

| 1 | (pocus or ultrasound* or sonograph* or (focused cardiac ultras* or cardiac ultras* or ((transthoracic or trans-thoracic) and echocardiogra*) or tte)).ti,ab,kw. | 448175 |
| --- | --- | --- |
| 2 | exp "Sensitivity and Specificity"/ or sensitivity.tw. or specificity.tw. or ((pre-test or pretest) adj probability).tw. or post-test probability.tw. or predictive value$.tw. or likelihood ratio$.tw. or (accurac* or diagnos* test?).ti,ab,kw. or false negative reactions/ or false positive reactions/ or (false adj (negative$1 or positive$1)).ti,ab. | 2654532 |
| 3 | (meta analysis or "systematic review").pt. or (systematic adj review$).ti,ab. or (metaanalys* or meta-analys*).ti. | 581111 |
| 4 | 1 and 2 and 3 | 3224 |
| 5 | (exp animals/ or exp animal experimentation/ or exp models, animal/ or exp plants/ or exp fungi/) not humans/ | 5921395 |
| 6 | (clinical study or clinical trial or comment or congress or editorial or letter or preprint).pt. | 3059355 |
| 7 | ((cancer? or chronic or perioperativ* or carcino* or neoplas*) not (acute* or critical or nonperioperativ* or non perioperativ* or emergen*)).ti,ab,kf. | 4554784 |
| 8 | 4 not 5 not 6 not 7 | 2405 |

| **B) Embase <1974 to April 21, 2026>** | |  |
| --- | --- | --- |

| 1 | (pocus or ultrasound* or sonograph* or (focused cardiac ultras* or cardiac ultras* or ((transthoracic or trans-thoracic) and echocardiogra*) or tte)).ti,ab,kw. | 740144 |
| --- | --- | --- |
| 2 | exp "Sensitivity and Specificity"/ or exp false positive result/ or exp false negative result/ or exp diagnostic accuracy/ or sensitivity.tw. or specificity.tw. or ((pre-test or pretest) adj probability).tw. or post-test probability.tw. or predictive value$.tw. or likelihood ratio$.tw. or (accurac* or diagnos* test?).ti,ab,kw. or (false adj (negative$1 or positive$1)).ti,ab. | 3223033 |
| 3 | systematic review/ or meta analysis/ or (systematic review* or meta-analys* or metaanalys*).ti,ab,kw. | 925072 |
| 4 | 1 and 2 and 3 | 5562 |
| 5 | (exp animal/ or exp invertebrate/ or nonhuman/ or animal experiment/ or animal tissue/ or animal model/ or exp plant/ or exp fungus/) not (exp human/ or human tissue/) | 8607260 |
| 6 | (Conference Abstract or Conference Paper or Conference Review or preprint or Clinical Trial or note or letter).pt. | 10107539 |
| 7 | ((cancer? or chronic or perioperativ* or carcino* or neoplas*) not (acute* or critical or nonperioperativ* or non perioperativ* or emergen*)).ti,ab,kf. | 6316647 |
| 8 | 4 not 5 not 6 not 7 | 2889 |

### Table S3. Adapted AMSTAR 2 rating tool. The changes of the tools are highlighted through underlining or striking through. An asterixis indicates critical domains

**1. Did the research questions and inclusion criteria for the review include the components of PICO?**

Population

~~Intervention~~ Index test

~~Comparator group~~ Reference standard

Outcome

**2. Did the report of the review contain an explicit statement that the review methods were
established prior to the conduct of the review and did the report justify any significant deviations from the protocol?**

The authors state that they had a written protocol or guide that included ALL the following:

For Partial Yes:

Review question(s)

A search strategy

Inclusion/exclusion criteria

A risk of bias assessment

For Yes:

A meta-analysis/synthesis plan, if appropriate, and

A plan for investigating causes of heterogeneity

Justification for any deviations from the protocol

**~~3. Did the review authors explain their selection of the study designs for inclusion in the review?~~**

~~For Yes, the review should satisfy ONE of the following: Explanation for including only RCT, NRSI or both~~

**4.* Did the review authors use a comprehensive literature search strategy?**

For Partial Yes (all the following):

Searched at least 2 databases (relevant to research question)

Provided key word and/or search strategy

Justified publication restrictions (e.g. language)

For Yes, should also have

Searched the reference lists / bibliographies of included studies

Conducted search within 24 months of completion of the review

~~searched trial/study registries~~

included/consulted content experts in the field

~~where relevant, searched for grey literature~~

**5. Did the review authors perform study selection in duplicate?**

At least two reviewers independently agreed on selection of eligible studies and achieved consensus on

which studies to include

***OR*** two reviewers selected a sample of eligible studies and achieved good agreement (at least 80 percent),

with the remainder selected by one reviewer

**6. Did the review authors perform data extraction in duplicate?**

At least two reviewers achieved consensus on which data to extract from included studies

***OR*** two reviewers extracted data from a sample of eligible studies and achieved good agreement (at least

80 percent), with the remainder extracted by one reviewer.

**7. Did the review authors provide a list of excluded studies and justify the exclusions?**

For Partial Yes:

Provided a list of all potentially relevant studies that were read in full-text form but excluded from the

review

For Yes, must also have:

Justified the exclusion from the review of each potentially relevant study

**8. Did the review authors describe the included studies in adequate detail?**

For Partial Yes (**ALL** the following):

Described populations

Described ~~interventions~~ index test

Described ~~comparators~~ reference standard

Described outcomes

Described research designs

For Yes, should also have **ALL** the following:

Described population in detail

Described ~~interventions~~ index test in detail

Described ~~comparators~~ reference standard in detail

Described study’s setting

Timeframe for follow-up

**9.* Did the review authors use a satisfactory technique for assessing the risk of bias (RoB) in individual studies that were included in the review?**

For Partial Yes, must have assessed RoB:

From patient selection, index test, reference standard ~~confounding,~~ **~~AND~~**

~~From selection bias~~

For Yes, must also have assessed RoB:

~~Methods used to ascertain exposures and outcomes~~ flow and timing **AND**

Selection of the reported result from among multiple measurements or analyses of a specified outcome

**10. Did the review authors report on the sources of funding for the studies included in the review?**

Must have reported on the sources of funding for individual studies included in the review. Note: Reporting that the reviewers looked for this information but it was not reported by study authors also qualifies

**11.* If meta-analysis was performed did the review authors use appropriate methods for statistical combination of results?**

The authors justified combining the data in a meta-analysis ***AND*** they used an appropriate weighted

technique to combine study results, adjusting for heterogeneity if present

***~~AND~~*** ~~they statistically combined effect estimates from NRSI that were adjusted for confounding, rather than~~

~~combining raw data, or justified combining raw data when adjusted effect estimates were not available~~

***~~AND~~*** ~~they reported separate summary estimates for RCTs and NRSI separately when both were included in~~

~~the review~~

**12. If meta-analysis was performed, did the review authors assess the potential impact of RoB in individual studies on the results of the meta-analysis or other evidence synthesis**

Included only low risk of bias ~~RCTs~~ DTA studies

**OR**, if the pooled estimate was based on ~~RCTs and/or NRSI~~ DTA studies at variable RoB, the authors
 performed analyses to investigate possible impact of RoB on summary estimates of effect.

**13.* Did the review authors account for RoB in individual studies when interpreting/ discussing the results of the review?**

Included only low risk of bias ~~RCTs~~ DTA studies

**OR**, if ~~RCTs~~ DTA studies with moderate or high RoB~~, or NRSI~~ were included the review provided a
 discussion of the likely impact of RoB on the results

**14. Did the review authors provide a satisfactory explanation for, and discussion of, any heterogeneity observed in the results of the review?**

There was no significant heterogeneity in the results

**OR** if heterogeneity was present the authors performed an investigation of sources of any heterogeneity in
 the results and discussed the impact of this sources of any heterogeneity in the results and discussed the
 impact of this on the results of the review

**15. If they performed quantitative synthesis, did the review authors carry out an adequate investigation of publication bias (small study bias) and discuss its likely impact on the results of the review?**

Performed graphical or statistical tests for publication bias and discussed the likelihood and magnitude of impact of publication bias

**16. Did the review authors report any potential sources of conflict of interest, including any funding they received for conducting the review?**

The authors reported no competing interests **OR**

The authors described their funding sources and how they managed potential conflicts of interest

**Rating overall confidence in the results of the review**

**High:** No or one non-critical weakness

**Moderate:** More than one non-critical weakness: the systematic review has more than one weakness but no critical flaws. Multiple non-critical weaknesses may diminish confidence in the review and it may be appropriate to move the overall appraisal down from moderate to low confidence

**Low:** One critical flaw with or without non-critical weaknesses

**Critically low:** More than one critical flaw with or without non-critical weaknesses

*Critical domains (item: 4, 9, 11, 13)*

**Reasons for adaptions:**

- Domain 1: In contrast to interventional studies, in a DTA-SR the index test and reference standard is essential.
- Domain 2: Was changed to a non-critical weakness as the potential bias of a missing protocol in DTA compared to an interventional study is lower.
- Domain 3: The study designs for inclusions in DTA-SR is not critical. The question was removed.
- Domain 4: Trial registries and grey literature search are less important in DTA-SR.
- Domain 7: Was changed to a non-critical weakness as we consider publication bias to be less relevant in DTA studies compared to intervention studies, for which AMSTAR 2 was originally developed.
- Domain 8: see Domain 1; timeframe.
- Domain 9: Special tools, e.g., QUADAS-2, have been developed to assess the risk of bias in DTA studies.[39] These domains are well respected, thus used here over those in SR of interventional studies.
- Domain 11: Removable of RCTs and NRSI part.
- Domain 12/13: Study designs were adapted.
- Domain 15: Was changed to a non-critical weakness as publication bias is of lower importance in DTA-SR.

**Abbreviations:** DTA-SR = diagnostic test accuracy - systematic review; RoB = risk of bias.

### Table S4. The GRADE procedure in this umbrella review

| Pooled sensitivity respectively specificity with 95% confidence interval starts from a *high* level of evidence. The evidence level has been downgraded (from high, *moderate, low,* to *very low*) by evaluating each of the following GRADE domains. | |
| --- | --- |
|  |  |
| **GRADE domain** | **Downgrading evaluation criteria** |
| Risk of bias | A moderate adapted AMSTAR 2 rating may lead to a downgrade, if evidence is compromised; a low/critical low rating will lead to a downgrade of the evidence. |
| Inconsistency | Downgrade if the results of the included DTA studies in the original DTA-SR have a high heterogeneity. This will be evaluated through i) visual inspection of the forest plot, ii) an I²-Statistic, and iii) a Chi² Test (Cochran's Q) test  If there is no data reported to assess the inconsistency in the study (including supplement material), the study will be downgraded in this domain. |
| Indirectness | Downgrading if the evidence is not directly transferable to the research question of this umbrella review, e.g., most studies were performed in a specialist setting or the execution and interpretation of the ultrasound examination require extensive practice. |
| Imprecision | Downgrading if the confidence interval of the pooled accuracy measure is wide (defined as >0.10) or the confidence interval encompasses the clinically relevant thresholds for diagnostic accuracy (defined as <0.70). |

**Abbreviations:** DTA-SR = diagnostic test accuracy - systematic review.

The results were presented in a summary of findings table, which included the assessments for each domain and the overall quality rating for each outcome.

### Table S5. List of predefined conditions that we considered relevant or irrelevant to the emergency department.

| **Conditions considered to be included** | **Conditions considered to be excluded** |
| --- | --- |
| **Cardiovascular conditions** | |
| Abdominal aortic aneurysm  Acute aortic syndrome  Acute heart failure  Acute myocardial ischemia  Acute thoracic aortic syndrome  Aortic dissection  Atrial fibrillation  Cardiac arrest  Deep vein thrombosis  Endocarditis  Hemodynamic instability  Hypovolemic shock  Infective endocarditis  Intracardiac thrombus  Left ventricular systolic dysfunction  Left ventricular diastolic dysfunction  Pericardial effusion  Pericardial tamponade  Pulmonary edema  Pulmonary embolism  Pulmonary hypertension  Right ventricular strain  Ruptured abdominal aortic aneurysm  Septic shock  Shock | Abdominal aortic aneurysm surveillance  Complex valve assessment  Cardiac allograft vasculopathy  Cardiac tumors  Cardioaortic embolic sources in stroke  Cardiovascular imaging indication  Carotid atherosclerotic plaque  Carotid web  Endoleak after EVAR  Fluid responsiveness  Internal thoracic artery graft patency  Intracardiac thrombus  Left ventricular thrombus  Microemboli in carotid disease  Atrial fibrillation  Patent foramen ovale / right-to-left shunt  Peripheral arterial disease  Perioperative cardiac assessments  Prosthetic valve obstruction  Pulmonary hypertension  Renal artery stenosis |
| **Ear/Nose/Throat conditions** | |
| Deep neck space infection  Difficult airway  Dysphagia aspiration  Epiglottitis  Facial cellulitis  Foreign body in upper airway  Laryngeal edema  Neck abscess  Parapharyngeal abscess  Peritonsillar abscess  Post-extubation stridor  Retropharyngeal abscess  Salivary gland abscess  Sialolithiasis  Soft tissue neck infection  Tonsillitis  Upper airway obstruction  Vocal cord dysfunction  Vocal cord paralysis | Cervical lymph node metastasis  Dysphagia aspiration / pharyngeal residue  Hyperparathyroidism  Parathyroid adenoma  Post-extubation stridor  Salivary gland disease  Sjögren syndrome  Thyroid nodules  Thyroid cancer  Vocal cord paralysis  Vocal fold immobility |
| **Gastrointestinal condition** | |
| Abdominal abscess  Abdominal free fluid  Abdominal tuberculosis  Acute cholecystitis  Acute pancreatitis  Appendicitis  Biliary colic  Bowel ischemia  Budd–Chiari syndrome  Choledocholithiasis  Cholelithiasis  Diverticulitis  Gallbladder disease  Gastric content assessment  Gastrointestinal perforation  Hemoperitoneum  Intra-abdominal infection  Intestinal obstruction  Mesenteric ischemia  Pancreatitis complications  Small bowel obstruction | Cholangiocelular carcinoma  Colorectal cancer  Esophageal varices  Focal liver lesions  Gallbladder polyps / adenoma  Gastric cancer  Hepatic steatosis / fatty liver disease  Hepatocellular carcinoma  Inflammatory bowel disease  Liver fibrosis / cirrhosis  Liver metastases  Liver transplant vascular complications  Pancreatitis  Pancreatic cancer  Pancreatic lesions  Portal hypertension  Splenic malignancy  TIPS dysfunction |
| **Genitourinary conditions** | |
| Acute kidney injury  Bladder outlet obstruction  Fournier gangrene  Hematuria  Hydronephrosis  Pyelonephritis  Renal colic  Scrotal abscess  Scrotal pain  Testicular torsion  Urinary retention  Urolithiasis | Asymptomatic microhematuria  Bladder cancer  Kidney transplant complications  Prostate cancer  Renal cancer / renal masses  Testicular abnormalities |
| **Gynecological conditions** | |
| Adnexal masses  Cesarean scar pregnancy  Ectopic pregnancy  Gynecologic hemorrhage  Hemorrhagic ovarian cyst  Ovarian torsion  Pelvic inflammatory disease  Retained products of conception  Ruptured ovarian cyst  Tubo-ovarian abscess  Vaginal bleeding in pregnancy | Adnexal masses  Breast cancer / breast lesions  Endometrial cancer  Hydatidiform mole  Idiopathic granulomatous mastitis  Obstetric cardiac disorders  Ovarian cancer  Ovarian endometrioma  Ovarian tumors / adnexal masses  Posterior pelvic floor disorders  Preeclampsia prediction  Retained products of conception  Uterine anomalies  Uterine leiomyoma / uterine sarcoma  Vulvar cancer inguinal lymph node metastasis |
| **Intervention-related conditions** | |
| Arthrocentesis guidance  Central venous catheter malposition  Central venous catheter placement  Endotracheal tube placement  Epidural space confirmation  Lumbar puncture guidance  Nasogastric tube placement  Paracentesis guidance  Pericardiocentesis guidance  Pleural drainage guidance  Thoracentesis guidance  Tracheal/mainstem intubation  Vascular access guidance | Anterolateral thigh flap perforator mapping  Endotracheal tube placement / one-lung ventilation  Epidural space confirmation |
| **Musculoskeletal conditions** | |
| Abscess  Achilles tendon rupture  Cellulitis  CPPD  Giant cell arteritis  Gout  Joint effusion  Knee effusion  Large vessel vasculitis  Muscle injury  Necrotizing fasciitis  Necrotizing soft tissue infection  Retained soft tissue foreign body  Rotator cuff tear  Septic arthritis  Soft tissue abscess  Soft tissue foreign body  Soft tissue infection  Tendon rupture  Tenosynovitis | Adhesive capsulitis  Anterior inferior iliac spine impingement  Chronic lateral ankle ligament injury  Inclusion body myositis  Inflammatory arthritis  Morton neuroma  Osteoporosis  Rheumatoid arthritis  Sarcopenia  Shoulder pathology  Soft tissue lipoma  Soft tissue masses / tumors  Spondylarthritis / sacroiliitis |
| **Neurological conditions** | |
| Cauda equina syndrome  Cerebral vasospasm after subarachnoid hemorrhage  Elevated intracranial pressure  Intracranial artery stenosis  Intracranial hemorrhage  Intracranial mass lesion  Large vessel occlusion  Midline shift  Optic nerve sheath diameter elevation  Stroke  Traumatic brain injury  Vertebral artery stenosis | Amyotrophic lateral sclerosis  Carpal tunnel syndrome  Cerebral vasospasm after subarachnoid hemorrhage  Depressive disorders  Epilepsy surgery functional mapping  Infiltrative glioma  Parkinson’s disease / movement disorders  Progressive supranuclear palsy  Traumatic nerve injury  Ulnar neuropathy / cubital tunnel syndrome  Ventriculoperitoneal shunt malfunction |
| **Ocular conditions** | |
| Globe rupture  Lens dislocation  Ocular foreign body  Ocular trauma  Papilledema  Posterior vitreous detachment  Retinal detachment  Retrobulbar hematoma  Vitreous hemorrhage | Chronic retinal disease monitoring  Glaucoma assessment  Cataract evaluation  Ocular tumor characterization  Diabetic retinopathy screening  Macular degeneration  Chronic papilledema follow-up  Orbital vascular imaging  Advanced ophthalmologic biometry |
| **Respiratory conditions** | |
| Acute respiratory distress syndrome  Acute respiratory failure  Asthma exacerbation  COPD exacerbation  COVID-19 pneumonia  Hemothorax  Interstitial syndrome / B-lines  Pleural effusion  Pneumonia  Pneumothorax  Pulmonary consolidation  Respiratory distress | Interstitial lung disease  Malignant pleural effusion  Obstructive sleep apnea  Pleural adhesions  Pulmonary atelectasis  Tuberculosis |

The list was developed during and after completion of the expert-based screening process in order to transparently document which conditions and ultrasound applications were interpreted as being within or outside the scope of emergency medicine-relevant POCUS according to the predefined eligibility criteria. The list was not intended to be exhaustive, but rather to provide an overview of the applicability decisions made during the screening process.
