# Supplementary material for: Diagnostic accuracy of point-of-care ultrasound in adult non-traumatic emergency medicine: an umbrella review
Source: BMC Med. 2026 Jul 25;24:579. doi: 10.1186/s12916-026-05094-2 (PMC13689006; doi:10.1186/s12916-026-05094-2)
Supplement: Supplementary file 4 [file 12916_2026_5094_MOESM4_ESM.docx]

## Table S8a: Summary of pooled *cardiovascular* DTA pairs (index test vs reference standard), including GRADE certainty of evidence for sensitivity and specificity, AMSTAR 2 assessments, and information on the diagnostic procedure. For each POCUS indication, one DTA pair with pooled estimates from the included studies is presented.

| **Topic^1^**  n patient (n studies) | | **Sensitivity [%] (95% CI)** | **Specificity [%] (95% CI)** | **GRADE^2^ (Sens./Spec.)** | **Method. quality (AMSTAR 2)** | | **Index test** | **Reference standard** | | **Remark** | | **First author (year)** |
| --- | --- | --- | --- | --- | --- | --- | --- | --- | --- | --- | --- | --- |
| **Aortic disease** | |  |  |  |  |  | |  |  | |  | |
| X | Abd. aortic aneurysm  944 (11) | 97.5  (91.2; 99.2) | 98.9 (97.9; 99.5) | High ⊕⊕⊕⊕  High ⊕⊕⊕⊕ | Moderate | | Ultrasound (diameter ≥3 cm) | CT, RADUS, surgery, autopsy | | Non radiologist performed index Ultrasound | | Concannon[41] (2014) |
|  | Abd. aortic aneurysm  655 (7) | 99.0 (95.0; 100.0) | 99.0 (97.0; 99.0) | Moderate ⊖⊕⊕⊕  Moderate ⊖⊕⊕⊕ | Low | | Ultrasound (diameter ≥3 cm) | CT, Angiography, MRI, Surgery, RADUS | | ED physicians performed index US | | Rubano[42] (2013) |
|  | Abd. aortic aneurysm  NA (9) | 98.0 (96.0; 100) | 100  (99.0; 100) | Moderate ⊖⊕⊕⊕ Moderate ⊖⊕⊕⊕ | Low | | POCUS | CT, RADUS, MRI, angiography, laparotomy, autopsy | | Emergency department | | Shaban[43] (2025) |
| X | Ruptured AAA  628 (5) | 97.8 (95.4; 100) | 97.0 (93.9; 100) | Moderate ⊕⊖⊕⊕  Moderate ⊕⊖⊕⊕ | Moderate | | POCUS | CTA, surgical findings | | ED physicians performed index US | | Fernando[44] (2022) |
| X | Aortic dissection  9602 (14) | 62.4 (60.8; 64.0) | 87.5 (86.7; 88.3) | Low ⊕⊖⊕⊖  Moderate ⊕⊖⊕⊕ | Moderate | | TTE | CT/CTA, TEE, MRI, surgical findings, autopsy | | Population and setting not adequately reported | | Sutarjono[45] (2023) |
|  | Aortic dissection, Type A, 2240 (8) | 86.7 (80.5; 92.8) | 86.5 (77.4; 95.6) | Low ⊕⊖⊕⊖  Low ⊕⊖⊕⊖ | Moderate | | TTE | CXR, CT/CTA, CA, MRI, surgery, autopsy | | Not all studies report both sens and spec | | Zaki[46] (2023) |
| X | Aortic dissection, Type A, 2560 (8) | 58.0 (38.0; 76.0) | 94.0 (89.0; 97.0) | Low ⊕⊖⊕⊖  Moderate ⊕⊖⊕⊕ | Moderate | | TTE | CT, MRA, TEE, aortography, autopsy | |  | | Yeh[47] (2025) |
| X | Aortic dissection, Type B, NA (4) | 56.8 (32.5; 81.1) | 92.4 (81.6; 100) | Low ⊕⊖⊕⊖  Low ⊕⊖⊕⊖ | Moderate | | TTE | CXR, CT/CTA, CA, MRI, surgery, autopsy | | Not all studies report both sens and spec | | Zaki[46]  (2023) |
| X | Acute thoracic aortic syndrome NA (7) | 89.0 (82.0; 84.0) | 92.0 (88.0; 95.0) | Moderate ⊕⊕⊖⊕ High ⊕⊕⊕⊕ | Moderate | | Bedside focused TTE, direct sign of dissection, Type A dissection | CTA, TEE, MRI angiography, surgery, autopsy, follow-up | | Only low risk of bias studies | | Mani[48] (2025) |
| **CPR** | |  |  |  |  |  | |  |  | |  | |
| X | Cardiac arrest  2239 (15) | 87.0 (75.0; 94.0) | 70.0 (56.0; 82.0) | Low ⊕⊖⊕⊖  Low ⊕⊖⊕⊖ | Moderate | | POCUS  (absence of cardiac activity) | Termination / no return of circulation | | Medical cardiac arrest  Primary endpoint: death | | Albaroudi[49] (2024) |
|  | Cardiac arrest  568 (8) | 91.6 (84.6; 96.1) | 80.0  (76.1; 83.6) | Low ⊕⊖⊕⊖  Moderate ⊕⊖⊕⊕ | Moderate | | POCUS  (presence of cardiac activity) | Survival (ROSC) | | traumatic and medical Primary endpoint: ROSC | | Blyth[50] (2012) |
|  | Cardiac arrest  3684 (24) | 86.5 (78.9; 90.4) | 79.0 (67.1; 87.4) | Low ⊕⊖⊕⊖  Low ⊕⊖⊕⊖ | Moderate | | POCUS  (presence of cardiac activity) | Clinical outcome (ROSC, death) | | Medical and traumatic | | Breglia[51] (2026) |
|  | Cardiac arrest  NA (9) | 19.4 (13.9; 26.0) | 73.5 (58.9; 85.1) | Very low ⊖⊖⊕⊖  Very low ⊖⊖⊕⊖ | Critically low | | POCUS  (presence of cardiac activity) | Survival (ROSC) | | only out of hospital Primary endpoint: ROSC | | Dudek[52] (2023) |
|  | Cardiac arrest  1174 (6) | 60.3 (38.1; 78.9) | 91.5 (80.8; 96.5) | Low ⊕⊖⊕⊖  Low ⊕⊖⊕⊖ | Moderate | | POCUS  (presence of cardiac activity) | Survival (ROSC) | | Medical cardiac arrest  Primary endpoint: ROSC | | Lalande[53] (2019) |
|  | Cardiac arrest  82 (5) | 91.0 (67.0; 98.0) | 98.0 (13.0; 100) | Low ⊕⊖⊕⊖  Low ⊕⊖⊕⊖ | Moderate | | POCUS  (absence of cardiac activity) | Termination / no return of circulation | | Traumatic cardiac arrest  Primary endpoint: death | | Lalande[54] (2021) |
|  | Cardiac arrest  NA (10) | 90.0 (83.0; 94.0) | 78.0 (64.0; 88.0) | Very low ⊖⊖⊕⊖  Very low ⊖⊖⊕⊖ | Low | | POCUS  (absence of cardiac activity) | Survival to admission | | Primary endpoint: survival to hospital admission | | Tsou[55] (2017) |
|  | PEA 732 (10) | 86.0 (67.0; 95.0) | 64.0 (51.0; 75.0) | Very low ⊕⊖⊖⊖  Very low ⊕⊖⊖⊖ | Moderate | | TTE | Survival (ROSC) | |  | | Jian[56] (2025) |
| X | PEA 975 (13) | 89.0 (80.0; 94.0) | 73.0 (63.0; 81.0) | Low ⊕⊕⊖⊖  Very low ⊕⊖⊖⊖ | Moderate | | TTE | Follow-up (sudden hemodynamic arrest) | |  | | Jian[56] (2025) |
|  | PEA 820 (9) | 79.0 (58.0; 91.0) | 58.0 (47.0; 61.0) | Low ⊕⊕⊖⊖  Very low ⊕⊖⊖⊖ | Moderate | | TTE | Follow-up (sever hemodynamic deterioration) | |  | | Jian[56] (2025) |
| X | Post-cardiac arrest neurology, NA (3) | 77.0 (53.0; 100) | 98.0  (83.0; 100) | Low ⊕⊖⊕⊖  Low ⊕⊖⊕⊖ | Moderate | | Optical US | CPC, GOS | | Absolute values and free-text results don’t match | | Lee[57] (2019) |
| **Endocarditis** | |  |  |  |  |  | |  |  | |  | |
| X | Infective endocarditis  NA (7) | 61.0  (45.0; 75.0) | 94.0 (85.0; 98.0) | Low ⊕⊕⊖⊖  Low ⊕⊕⊖⊖ | Moderate | | TTE, harmonic | TEE | | Included patients with and without endocarditis | | Bai[58]  (2017) |
| X | Native endocarditis  2209 (11) | 71.0  (56.0; 82.0) | 80.0  (58.0; 92.0) | Very low ⊖⊖⊖⊖  Very low ⊖⊖⊖⊖ | Low | | TTE, fundamental or harmonic | TEE | |  | | Bonzi[59] (2018) |
| X | Prosthet. endocarditis  172 (9) | 36.0 (27.0; 46.0) | 93.0  (84.0; 97.0) | Very low ⊖⊖⊖⊖  Very low ⊖⊖⊖⊖ | Low | | TTE, periannular complications | Surgery, autopsy, or clinical follow-up | |  | | Habets[60] (2015) |
| **Heart failure** | |  |  |  |  |  | |  |  | |  | |
|  | Acute heart failure  1075 (7) | 94.1 (81.3; 98.3) | 92.4 (84.2; 96.4) | Low ⊕⊖⊕⊖  Low ⊕⊖⊕⊖ | Moderate | | LUS, B-Lines | Clinical follow-up | | Includes ICU and ward patients | | Al Deeb[61] (2014) |
|  | Acute heart failure  2563 (8) | 91.8 (80.5; 96.7) | 92.3 (86.6; 95.7) | Low ⊕⊖⊕⊖  Moderate ⊕⊖⊕⊕ | Moderate | | LUS, B-Lines | Medical record audit | |  | | Chiu[62] (2022) |
|  | Acute heart failure  708 (5) | 76.0 (48.0; 91.0) | 96.0 (90.0; 98.0) | Low ⊕⊖⊕⊖  Moderate ⊕⊖⊕⊕ | Moderate | | LUS | Chart review | | Internists or emergency physicians | | Gartlehner[63] (2021) |
|  | Acute heart failure  3309 (15) | 85.0 (84.0; 87.0) | 91.0 (89.0; 92.0) | Moderate ⊕⊖⊕⊕  Moderate ⊕⊖⊕⊕ | Moderate | | LUS, ultrasound lung comets | Final diagnosis | |  | | Lian[64]  (2018) |
|  | Acute heart failure 4390 (18) | 92.0 (81.0; 97.0) | 96.0 (93.0; 98.0) | Very low ⊕⊖⊖⊖ Low ⊕⊖⊖⊕ | Moderate | | POCUS (LVEF, B-lines, IVC) | Final diagnosis, expert consensus | |  | | Lv[65] (2024) |
| X | Acute heart failure  1918 (8) | 85.3 (82.8, 87.5) | 92.7 (90.0, 94.3) | Moderate ⊕⊖⊕⊕  Moderate ⊕⊖⊕⊕ | Moderate | | LUS, B-Lines | Clinical chart review (blinded) | |  | | Martindale[66] (2016) |
|  | Acute heart failure  1861 (7) | 82.5 (66.4; 91.8) | 83.6 (72.4; 90.8) | Low ⊕⊖⊕⊖  Moderate ⊕⊖⊕⊕ | Moderate | | LUS, Volpicelli method | Chart review | |  | | McGivery[67] (2018) |
|  | Acute heart failure  692 (8) | 86.7 (70.8; 94.6) | 87.5 (78.2; 93.2) | Low ⊕⊕⊖⊖  Low ⊕⊕⊖⊖ | Moderate | | LUS, varying protocols | Final hospital/ed diagnosis | |  | | Russel[68] (2024) |
|  | Acute heart failure  1208 (8) | 73.0 (48.0; 89.0) | 84.0 (76.0; 90.0) | Low ⊕⊖⊕⊖  Low ⊕⊖⊕⊖ | Moderate | | LUS, diffuse interstitial syndrome | Final diagnosis by experts including radiology | |  | | Staub[69] (2018) |
|  | Acute heart failure  591 (7) | 79.1 (86.5; 86.8) | 81.8 (75.0; 87.0) | Low ⊕⊖⊕⊖  Moderate ⊕⊕⊕⊖ | Moderate | | VCI assessment | ≥ 2 criteria for heart failure (ESC) | |  | | Squizzato[70] (2021) |
| X | LV diastolic function  298 (4) | 94.0 (87.0; 97.0) | 59.0 (42.0; 74.0) | High ⊕⊕⊕⊕ Low ⊕⊖⊕⊖ | Moderate | | Focused cardiac ultrasound | TTE, review by cardiologist, invasiv | | Emergency physician | | Huang[71] (2025) |
| X | LV systolic function  688 (7) | 89.0  (80.0; 94.0) | 85.0 (80.0; 89.0) | Low ⊕⊖⊕⊖  Moderate ⊕⊖⊕⊕ | Moderate | | TTE, visual estimation of LVEF by EP | TTE, visual estimation of LVEF by expert | |  | | Albaroudi[72] (2022) |
| X | Pulmonary edema  770 (5) | 89.0 (81.0; 93.0) | 94.0 (89.0; 96.0) | Moderate ⊕⊕⊕⊖  High ⊕⊕⊕⊕ | Moderate | | LUS, BLUE protocol | Echo, radiography, clinical parameters | |  | | Asmara[73] (2022) |
|  | Pulmonary edema  2097 (9) | 92.0 (84.0; 97.0) | 87.0 (82.0; 91.0) | Very low ⊖⊖⊕⊖  Low ⊖⊖⊕⊕ | Low | | LUS, B-Lines | Discharge diagnosis, expert panel | |  | | Dong[74] (2023) |
|  | Pulmonary edema  1301 (8) | 88.0 (75.0; 95.0) | 90.0 (88.0; 92.0) | Low ⊕⊖⊕⊖ Moderate ⊕⊖⊕⊕ | Moderate | | LUS | Chart review, TTE, BNP, Chest x-ray | | Emergency department and medical ward | | Maw[75]  (2019) |
|  | Pulmonary edema  1301 (8) | 97.0 (96.0; 98.0) | 98.0 (97.0; 99.0) | Low ⊖⊖⊕⊕  Low ⊖⊖⊕⊕ | Critically low | | LUS, B-Lines | Final hospital/emergency diagnosis | |  | | Wang[76] (2018) |
| **Myocardial ischemia** | |  |  |  |  |  | |  |  | |  | |
| X | Myocardial ischemia  1790 (6) | 83.1  (60.2; 94.1) | 89.2  (73.4; 96.1) | Low ⊕⊖⊕⊖  Low ⊕⊖⊕⊖ | Moderate | | Cardiac ultrasound (TTE, Limited TTE, POCUS) | final diagnosis, coronary angiography | | Emergency physicians | | Zarama[77]  (2024) |
| X | Myocardial infarction  2138 (8) | 90.7 (78.3; 96.3) | 67.4  (49.4; 81.4) | Low ⊕⊖⊕⊖  Low ⊕⊖⊕⊖ | Moderate | | Cardiac ultrasound (TTE, Limited TTE, POCUS) | final diagnosis, coronary angiography | |  | | Zarama[77]  (2024) |
| X | ACS 2433 (16) | 68.0 (53.3; 79.8) | 93.2 (88.2; 96.2) | Low ⊕⊖⊕⊖  Low ⊕⊖⊕⊖ | Moderate | | Cardiac ultr  asound (TTE, Limited TTE, POCUS) | final diagnosis, coronary angiography | |  | | Zarama[77]  (2024) |
| X | Sig coronary stenosis 472 (5) | 85.0 (65.4; 94.4) | 82.7 (81.5; 91.2) | Low ⊕⊖⊕⊖  Moderate ⊕⊖⊕⊕ | Moderate | | Cardiac ultrasound (TTE, Limited TTE, POCUS) | final diagnosis, coronary angiography | |  | | Zarama[77]  (2024) |
| **Shock related** | |  |  |  |  |  | |  |  | |  | |
|  | Cardiogenic Shock  357 (4) | 83.0 (71.0; 92.0) | 97.0 (95.0; 99.0) | Low ⊕⊖⊕⊖  High ⊕⊕⊕⊕ | Moderate | | RUSH or identical POCUS protocol | Chart review, expert consensus | |  | | Stickles[78] (2019) |
| X | Cardiogenic Shock  743 (7) | 75.0  (49.0; 90.0) | 94.0  (88.0; 97.0) | Moderate ⊕⊕⊕⊖  High ⊕⊕⊕⊕ | Moderate | | RUSH | Chart review | |  | | Yoshida[79] (2023) |
|  | Cardiogenic Shock  1217 (14) | 95.0  (84.0; 98.0) | 98.0  (97.0; 99.0) | Very low ⊖⊖⊕⊖  Very low ⊖⊖⊕⊖ | Low | | RUSH or identical POCUS protocol | Chart review, expert consensus | |  | | Basmaji[80] (2025) |
|  | Cardiogenic Shock  783 (8) | 86.0  (72.0; 94.0) | 96.0 (94.0; 97.0) | Moderate ⊖⊕⊕⊕ Moderate ⊖⊕⊕⊕ | Low | | POCUS | Final diagnosis | |  | | Osawa[81] (2025) |
|  | Distributive Shock  357 (4) | 93.0 (68.0; 100) | 98.0 (96.0; 99.0) | Low ⊕⊖⊕⊖  Moderate ⊕⊖⊕⊕ | Moderate | | RUSH or identical POCUS protocol | Chart review, expert consensus | |  | | Stickles[78] (2019) |
| X | Distributive Shock  509 (6) | 78.0 (69.0; 84.0) | 98.0 (95.0; 99.0) | Moderate ⊕⊕⊕⊖  High ⊕⊕⊕⊕ | Moderate | | RUSH | Chart review | |  | | Yoshida[79] (2023) |
|  | Distributive Shock  1327 (15) | 78.0 (69.0; 85.0) | 97.0 (94.0; 99.0) | Very low ⊖⊖⊕⊖  Low ⊖⊕⊕⊖ | Low | | RUSH or identical POCUS protocol | Chart review, expert consensus | |  | | Basmaji[80] (2025) |
|  | Hypovolemic Shock  357 (4) | 81.0 (73.0; 88.0) | 91.0  (87.0; 94.0) | Low ⊕⊖⊕⊖  Moderate ⊕⊖⊕⊕ | Moderate | | RUSH or identical POCUS protocol | Chart review, expert consensus | |  | | Stickles[78] (2019) |
| X | Hypovolemic Shock  602 (7) | 91.0 (84.0; 95.0) | 92.0 (85.0; 96.0) | Moderate ⊕⊕⊕⊖  Moderate ⊕⊕⊕⊖ | Moderate | | RUSH | Chart review | |  | | Yoshida[79] (2023) |
|  | Hypovolemic Shock  1397 (16) | 90.0 (81.0; 95.0) | 95.0 (90.0, 97.0) | Very low ⊖⊖⊕⊖  Very low ⊖⊖⊕⊖ | Low | | RUSH or identical POCUS protocol | Chart review, expert consensus | |  | | Basmaji[80] (2025) |
|  | Obstructive Shock  239 (3) | 75.0 (53.0; 90.0) | 80.0 (73.0; 85.0) | Moderate ⊕⊕⊕⊖  High ⊕⊕⊕⊕ | Moderate | | RUSH or identical POCUS protocol | Chart review, expert consensus | |  | | Stickles[78] (2019) |
| X | Obstructive Shock  725 (7) | 83.0 (65.0; 92.0) | 97.0 (87.0; 99.0) | Moderate ⊕⊕⊕⊖  Moderate ⊕⊕⊕⊖ | Moderate | | RUSH | Chart review | |  | | Yoshida[79] (2023) |
|  | Obstructive Shock  1132 (13) | 94.0 (85.0; 97.0) | 99.0 (98.0; 100) | Low ⊖⊕⊕⊖  Low ⊖⊕⊕⊖ | Low | | RUSH or identical POCUS protocol | Chart review, expert consensus | |  | | Basmaji[80] (2025) |
|  | Obstructive Shock  863 (8) | 78.0 (63.0; 88.0 | 98.0 (94.0; 99.0) | Low ⊖⊕⊕⊖ Moderate ⊖⊕⊕⊕ | Low | | POCUS | Final diagnosis | |  | | Osawa[81] (2025) |
| X | Shock etiology: mixed  332 (3) | 64.0 (56.0; 72.0) | 95.0 (91.0; 98.0) | Moderate ⊕⊕⊕⊖  Low ⊕⊖⊕⊖ | Moderate | | RUSH or identical POCUS protocol | Chart review, expert consensus | |  | | Stickles[78] (2019) |
|  | Shock etiology: mixed  929 (8) | 85.0 (77.0; 91.0) | 98.0 (91.0; 100) | Very low ⊖⊖⊕⊖  Very low ⊖⊖⊕⊖ | Low | | RUSH or identical POCUS protocol | Chart review, expert consensus | |  | | Basmaji[80] (2025) |
| **Venous thromboembolism** | | | | |  |  | |  |  | |  | |
|  | DVT  936 (6) | 95.0 (87.0; 99.0) | 96.0 (87.0; 99.0) | Low ⊕⊖⊕⊖  Low ⊕⊖⊕⊖ | Moderate | | Ultrasound of one re both legs | RADUS or vascular department | | Emergency physicians | | Burnside[82] (2008) |
|  | DVT  NA (100) | 89.7 (88.8, 90.5) | 93.8 (93.1; 94.4) | Moderate ⊕⊕⊖⊕  Moderate ⊕⊕⊖⊕ | Moderate | | Ultrasound (compression, color doppler, cw) | Venography | |  | | Goodacre[83] (2006) |
|  | DVT  212 (5) | 92.5  (81.8; 97.9) | 98.7 (95.5; 99.9) | Very low ⊖⊕⊖⊖  Very low ⊖⊕⊖⊖ | Critically low | | Ultrasound of all deep veins in the calf | Venography | |  | | Gottlieb[84] (1999) |
|  | DVT  2311 (15) | 90.0 (82.0; 95.0) | 95.0 (91.0; 97.0) | Low ⊕⊖⊕⊖  Moderate ⊕⊖⊕⊕ | Moderate | | 2-point and 3-point compression ultrasound | RADUS or angiology | | Emergency physicians | | Hercz[85] (2024) |
|  | DVT  1337 (9) | 91.0 (68.0; 98.0) | 98.0 (96.0; 99.0) | Low ⊕⊖⊕⊖  Low ⊕⊖⊕⊖ | Moderate | | 2-point compression ultrasound | Contrast venography, follow up RADUS | | Emergency physicians | | Lee[86]  (2019) |
| X | DVT  2379 (16) | 96.1 (90.6; 98.5) | 96.8 (94.6, 98.1) | High ⊕⊕⊕⊕  High ⊕⊕⊕⊕ | Moderate | | Ultrasound (duplex, proximal venous, whole leg) | RADUS or angiology lab, angiography | | Emergency physicians | | Pomero[87] (2013) |
|  | DVT 1689 (12) | 92.0 (88.0; 97.0) | 97.0 (95.0; 99.0) | Moderate ⊕⊖⊕⊕ Moderate ⊕⊖⊕⊕ | Moderate | | 2-point ultrasound | RADUS, venography | | Emergency department | | Zaki[88] (2024) |
|  | Pulmonary embolism  643 (2) | 65.5 (31.3; 99.6) | 99.0 (98.3; 100) | Low ⊕⊖⊕⊖  High ⊕⊕⊕⊕ | Moderate | | LUS, BLUE Protocol | Echo, radiography, clinical parameters | |  | | Asmara[73] (2022) |
|  | Pulmonary embolism  426 (10) | 77.0 (50.0; 92.0) | 99.0 (97.0; 100.0) | Very low ⊖⊖⊕⊖  Low ⊖⊖⊕⊕ | Low | | Cardiopulmonary ultrasound | CTPA or clinically derived diagnostic criterion/expert | |  | | Cao[89]  (2022) |
|  | Pulmonary embolism  2158 (18) | 80.0 (71.0; 86.0) | 87.0 (81.0; 92.0) | Very low ⊕⊖⊖⊖  Very low ⊕⊖⊖⊖ | Moderate | | LUS (various, mostly including typical lesions) | CT, MRI, angiography, V/Q scan | |  | | Du[90]  (2024) |
|  | Pulmonary embolism  281 (3) | 91.8 (84.5; 96.4) | 63.9 (56.5; 70.9) | Low ⊕⊖⊕⊖  Low ⊕⊖⊕⊖ | Moderate | | Multiorgan US, RV dilation or DVT | CT, MRI, angiography, V/Q scan, autopsy | |  | | Falster[91] (2021) |
|  | Pulmonary embolism  1986 (16) | 53.0 (45.0; 61.0) | 83.0 (74.0; 90.0) | Very low ⊖⊖⊕⊖  Very low ⊖⊖⊕⊖ | Low | | TTE (right heart strain) | CT, Angiography, V/Q scan, surgery, autopsy | |  | | Fields[92] (2017) |
|  | Pulmonary embolism  1356 (13) | 85.0 (78.0; 90.0) | 83.0 (73.0; 90.0) | Very low ⊖⊖⊕⊖  Very low ⊖⊖⊕⊖ | Low | | LUS | CTPA, V/Q scan, combination | |  | | Jiang[93] (2015) |
|  | Pulmonary embolism  453 (2) | 91.0 (84.0; 95.0) | 81.0 (77.0; 85.0) | Low ⊕⊖⊕⊖  Moderate ⊕⊖⊕⊕ | Moderate | | Multi-organ POCUS, cardiac and LUS | CTPA, clinically derived diagnosis | |  | | Kagima[94] (2020) |
| X | Pulmonary embolism  594 (4) | 90.0 (85.0; 94.0) | 69.0 (42.0; 87.0) | High ⊕⊕⊕⊕ Moderate ⊕⊕⊕⊖ | Moderate | | Multi-organ ultrasound (lung, cardiac and venous POCUS) | CTPA and/or V/Q scan | |  | | Melo[95] (2025) |
|  | Pulmonary embolism  352 (5) | 80.0 (72.0; 86.9) | 93.0 (90.1; 95.8) | Low ⊕⊖⊕⊖  High ⊕⊕⊕⊕ | Moderate | | LUS | CT, MRI or CT/US/D-Dimers | |  | | Niemann[96] (2009) |
|  | Pulmonary embolism  887 (10) | 87.0 (79.0; 92.0) | 81.0  (71.0; 89.0) | Low ⊖⊖⊕⊕  Low ⊖⊖⊕⊕ | Low | | LUS | Composite reference / any imaging test | |  | | Squizzato[97] (2013) |
|  | Pulmonary embolism NA (10) | 79.0 (64.0; 94.0) | 78.0 (68.0; 90.0) | Low ⊕⊖⊕⊖ Low ⊕⊖⊕⊖ | Moderate | | Multiorgan ultrasound, overall | CT, CTPA, MRA, autopsy | | Emergency department | | Zaki[98] (2025) |

^1^ “X” indicates the DTA pair selected as the representative for the condition presented in main Tables 2 or 3. For conditions with multiple eligible DTA pairs, selection followed a predefined hierarchy prioritizing: (i) the highest AMSTAR rating, (ii) the highest GRADE certainty, and (iii) the strongest emergency department focus. ^2^ GRADE is detailed as i) Risk of Bias (⊝/⊕), ii) Consistency (⊝/⊕), iii) Directness (⊝/⊕), and iv) Precision (⊝/⊕).

**Abbreviations**: AAA = abdominal aortic aneurysm; ACS = acute coronary syndrome; BLUE protocol = bed lung ultrasound in emergency protocol; CA = coronary angiography; CPC = Glasgow-Pittsburg Cerebral Performance; CT = computed tomography; CTA = computed tomography angiography; CW = continuous wave Doppler; CXR = chest X-ray; GOS = Glasgow Outcome Scale; DTA = diagnostic test accuracy; DVT = deep vein thrombosis; ED = emergency department; EP = emergency physician; ESC = European Society of Cardiology; ICU = intensive care unit; IVC = inferior vena cava; LUS = lung ultrasound, LVEF = left ventricular ejection fracture; MRI = magnetic resonance imaging; n = number; PEA = pulseless electrical activity; POCUS = point of care ultrasound; RAD = radiology; RADUS = radiology performed ultrasound; ROSC = return of spontaneous circulation; RUSH = Rapid Ultrasound for Shock and Hypotension; RV dilation = right ventricular dilation; Sens. = sensitivity; Sig = significant; Spec. = specificity; US = ultrasound; TEE = transesophageal echocardiography; TTE = transthoracic echocardiography; VCI = inferior vena cava; V/Q scan = ventilation-perfusion scan

## Table S8b: Summary of pooled *ear / nose / throat* DTA pairs (index test vs reference standard), including GRADE certainty of evidence for sensitivity and specificity, AMSTAR 2 assessments, and information on the diagnostic procedure. For each POCUS indication, one DTA pair with pooled estimates from the included studies is presented.

| **Topic^1^**  n patient (n studies) | | **Sensitivity [%] (95% CI)** | **Specificity [%] (95% CI)** | **GRADE^2^ (Sens./Spec.)** | **Method. quality (AMSTAR 2)** | **Index test** | **Reference standard** | **Remark** | **First author (year)** |
| --- | --- | --- | --- | --- | --- | --- | --- | --- | --- |
| X | Peritonsillar abscess 452 (6) | 74.0 (67.0; 80.0) | 79.0 (61.0; 90.0) | Low ⊕⊖⊕⊖ Low ⊕⊖⊕⊖ | Moderate | POCUS, transcervical and intraoral | Drainage or CT | Overall small population except one study | Kim[99]  (2023) |

^1^ “X” indicates the DTA pair selected as the representative for the condition presented in main Tables 2 or 3. ^2^ GRADE is detailed as i) Risk of Bias (⊝/⊕), ii) Consistency (⊝/⊕), iii) Directness (⊝/⊕), and iv) Precision (⊝/⊕).

**Abbreviations:** CI = confidence interval; DTA = diagnostic test accuracy; n = number; POCUS = point of care ultrasound; Sens. = sensitivity; Spec. = specificity

## Table S8c: Summary of pooled *gastrointestinal* DTA pairs (index test vs reference standard), including GRADE certainty of evidence for sensitivity and specificity, AMSTAR 2 assessments, and information on the diagnostic procedure. For each POCUS indication, one DTA pair with pooled estimates from the included studies is presented.

| **Topic^1^**  n patient (n studies) | | **Sensitivity [%] (95% CI)** | **Specificity [%] (95% CI)** | **GRADE^2^ (Sens./Spec.)** | **Method. quality (AMSTAR 2)** | **Index test** | **Reference standard** | **Remark** | **First author (year)** |
| --- | --- | --- | --- | --- | --- | --- | --- | --- | --- |
| **Appendicitis** | |  |  |  |  |  |  |  |  |
|  | Appendicitis  2549 (16) | 82.1 (73.8; 88.2) | 85.9  (72.7; 93.3) | Low ⊕⊝⊕⊝  Low ⊕⊝⊕⊝ | Moderate | NA | Pathology, surgery, follow-up | Mostly emergency department | Arruzza[100]  (2022) |
|  | Appendicitis  2735 (21) | 81.0 (78.0, 83.0) | 87.0  (85.0, 88.0) | Moderate ⊕⊝⊕⊕  Moderate ⊕⊝⊕⊕ | Moderate | B-mode ultrasound | Pathology, surgery, follow-up | Emergency department | Cho[101]  (2023) |
|  | Appendicitis  1334 (10) | 80.0 (76.0; 83.0) | 92.0 (90.0; 94.0) | Moderate ⊕⊝⊕⊕  Moderate ⊕⊝⊕⊕ | Moderate | B-mode ultrasound | Pathology, surgery, follow-up, CT | Emergency department | Fields[102]  (2017) |
|  | Appendicitis  4600 (18) | 77.2 (75.4; 78.9) | 80.0 (60.0; 92.0) | Moderate ⊕⊝⊕⊕  Low ⊕⊝⊕⊝ | Moderate | B-mode ultrasound | Pathology, surgery, follow-up, CT |  | Fu[103]  (2021) |
|  | Appendicitis  2841 (17) | 69.0 (59.0; 78.0) | 81.0 (73.0; 88.0) | Very low ⊝⊝⊝⊕  Very low ⊝⊝⊝⊕ | Low | B-mode ultrasound | Pathology, surgery |  | Giljaca[104]  (2017) |
| X | Appendicitis  2021 (15) | 78.0 (69.0; 85.0) | 89.0 (83.0; 93.0) | Low ⊕⊖⊕⊖ Moderate ⊕⊖⊕⊕ | High | B-mode ultrasound | Pathology, surgery, follow-up, CT, MRI | Emergency physicians | Kamal[105] (2026) |
|  | Appendicitis  2385 (17) | 84.0 (72.0, 92.0) | 91.0 (85.0, 95.0) | Low ⊕⊝⊕⊝  Moderate ⊕⊝⊕⊕ | Moderate | B-mode ultrasound (Cut-off diameter: 6 or 7mm) | Pathology, surgery, follow-up | By emergency physician; all studies high risk of bias | Lee[106]  (2019) |
|  | Appendicitis  521 (5) | 62.0 (43.0; 78.0) | 91.0 (74.0; 97.0) | Very low ⊝⊝⊝⊝  Very low ⊝⊝⊝⊝ | Low | NA | Pathology, surgery | Only Pregnant women, mostly radiological | Li[107]  (2022) |
|  | Appendicitis  1593 (8) | 77.6 (75.4; 79.6) | 75.3 (73.1; 77.5) | Very low ⊝⊝⊝⊕  Very low ⊝⊝⊝⊕ | Low | NA | Pathology, surgery, CT | Only Pregnant women | Moghadam[108]  (2022) |
|  | Appendicitis  NA (12) | 82.0 (70.0; 90.0) | 97.0 (92.0; 99.0) | Very low ⊕⊖⊖⊖  Low ⊕⊖⊖⊕ | Moderate | Color Doppler | Pathology |  | Montero[109]  (2026) |
|  | Appendicitis  NA (11) | 65.3 (52.68; 77.9) | 88.9 (83.2; 94.6) | Low ⊕⊝⊕⊝  Low ⊕⊝⊕⊝ | Moderate | B-mode ultrasound | Pathology, surgery, RADUS, CT | Emergency department | Popat[110]  (2024) |
|  | Appendicitis  1516 (14) | 86.0 (83.0; 88.0) | 81.0 (78.0; 84.0) | Moderate ⊕⊖⊕⊕ Moderate ⊕⊖⊕⊕ | Moderate | B-mode ultrasound | Pathology, surgery, follow-up | Emergency department, medical ward | Terasawa[111] (2004) |
|  | Appendicitis  239 (4) | 56.0 (45.0; 65.0)) | 88.0  (64.0; 97.0) | Very low ⊝⊝⊝⊝  Very low ⊝⊝⊝⊝ | Low | Compression in B-mode ultrasound | NA | Only Pregnant women | Wang[112]  (2023) |
|  | Appendicitis  4801 (21) | 87.1 (84.9, 88.9) | 92.8 (91.5, 93.9) | Very low ⊝⊝⊝⊕  Very low ⊝⊝⊝⊕ | Low | Compression in B-mode ultrasound | Pathology, surgery |  | Weston[113]  (2005) |
|  | Appendicitis  2643 (22) | 86.7 (85.4; 88.0) | 90.0 (88.9; 91.2) | Very low ⊝⊝⊝⊕  Very low ⊝⊝⊝⊕ | Low | Compression in B-mode ultrasound | Pathology, surgery, follow-up |  | Yu[114]  (2005) |
| **Biliary system** | |  |  |  |  |  |  |  |  |
|  | Acute Cholecystitis  5714 (18) | 69.0 (62.0; 76.0) | 79.0 (71.0; 85.0) | Low ⊕⊝⊕⊝  Low ⊕⊝⊕⊝ | Moderate | B-mode ultrasound | Surgery, follow-up |  | Childs[115]  (2023) |
|  | Acute Cholecystitis  7123 (25) | 77.4 (69.9; 83.5) | 88.4  (83.1; 92.2) | Very low ⊝⊝⊝⊝  Very low ⊝⊝⊕⊝ | Low | B-mode ultrasound | Surgery, follow-up | Excluded POCUS | De Oliveira[116]  (2024) |
|  | Acute Cholecystitis  2638 (14) | 71.0 (67.0; 74.0) | 92.0 (90.0; 93.0) | Very low ⊝⊝⊕⊝  Low ⊝⊝⊕⊕ | Low | B-mode ultrasound | Pathology, surgery, RADUS, follow-up |  | Huang[117]  (2023) |
|  | Acute Cholecystitis  2847 (26) | 81.0 (75.0; 87.0) | 83.0 (74.0; 89.0) | Low ⊕⊝⊕⊝ Low ⊕⊝⊕⊝ | Moderate | B-mode ultrasound | Surgery, follow-up | Emergency department | Kiewiet[118]  (2012) |
| X | Acute Cholecystitis  NA (4) | 74.7 (58.1; 91.4) | 95.6 (92.2; 99.0) | Low ⊕⊝⊕⊝  Moderate ⊕⊝⊕⊕ | Moderate | B-mode ultrasound | Pathology, surgery, RADUS, follow-up | Emergency department | Popat[110]  (2024) |
|  | Acute Cholecystitis  2356 (10) | 78.6 (67.8, 86.5) | 94.9 (89.7, 97.5) | Very low ⊝⊝⊕⊝  Very low ⊝⊝⊕⊝ | Moderate | B-mode ultrasound | Pathology, surgery, RADUS, follow-up | Many pediatric patients included as well | Wilson[119]  (2024) |
|  | Acute Cholecystitis  1320 (5) | 73.0  (55.0, 86.0) | 93.0  (82.0, 88.0) | Low ⊝⊕⊕⊝  Low ⊝⊕⊕⊝ | Low | B-mode ultrasound | Pathology, surgery, RADUS, CT |  | Wu[120]  (2023) |
| X | Choledocholithiasis  523 (5) | 73.0 (44.0; 90.0) | 91.0  (84.0; 95.0) | Very low ⊕⊝⊝⊝  Very low ⊕⊝⊝⊝ | Moderate | B-mode ultrasound | Surgery, follow-up |  | Gurusamy[121]  (2015) |
| X | Cholelithiasis  710 (8) | 89.8  (86.4; 92.5) | 88.0  (83.7, 91.4) | Moderate ⊕⊕⊕⊖  High ⊕⊕⊕⊕ | Moderate | B-mode ultrasound | Surgery, RADUS, CT, MRI | By emergency physician | Ross[122]  (2011) |
|  | Cholelithiasis  144 (2) | 94.0 (91.0; 98.0) | 93.0  (91.0; 95.0) | Very low ⊝⊝⊝⊝  Very low ⊝⊝⊝⊕ | Low | B-mode ultrasound | Pathology, surgery, RADUS, CT |  | Wu[120]  (2023) |
| X | Gallbladder disease  1464 (7) | 86.0  (75.0; 93.0) | 92.0  (87.0; 96.0) | Very low ⊝⊝⊝⊝  Very low ⊝⊝⊝⊝ | Low | B-mode ultrasound | Pathology, surgery, RADUS, CT |  | Wu[120]  (2023) |
| **Intestinal obstruction** | | |  |  |  |  |  |  |  |
|  | Small bowel obstruct.  625 (5) | 93.0 (89.0; 95.0) | 96.0 (86.0; 99.0) | Moderate ⊕⊝⊕⊕  Low ⊕⊝⊕⊝ | Moderate | B-mode ultrasound, fluid filled small bowel ≥ 25 mm | Enteroclysis, surgery, diagnosis, follow-up, CT | Pediatric pop included  Emergency department | Gottlieb[123]  (2018) |
| X | Small bowel obstruct.  1004 (7) | 95.0 (92.0; 96.0) | 77.0 (73.0; 80.0) | Moderate ⊕⊝⊕⊕  Moderate ⊕⊝⊕⊕ | Moderate | B-mode ultrasound, small bowel ≥ 25 mm, no peristalsis | Surgery, follow-up, CT | Emergency department | Lin[124]  (2021) |
|  | Small bowel obstruct.  NA (6) | 93.2 (88.8; 97.5) | 81.8 (69.3; 94.3) | Moderate ⊕⊝⊕⊕  Low ⊕⊝⊕⊝ | Moderate | B-mode ultrasound | Pathology, Surgery, CT | Pediatric population included | Popat[110]  (2024) |
|  | Small bowel obstruct.  433 (5) | 83.0 (71.7; 90.4) | 93.0 (55.3; 99.3) | Low ⊕⊝⊕⊝  Low ⊕⊝⊕⊝ | Moderate | B-mode ultrasound, fluid filled small bowel ≥ 25 mm | Surgery, CT | Ultrasound by emergency physician | Shokoohi[125]  (2023) |
|  | Small bowel obstruct.  250 (2) | 97.0 (92.0; 99.0) | 90.0 (84.0; 95.0) | Moderate ⊝⊕⊕⊕  Low ⊝⊝⊕⊕ | Low | B-mode ultrasound, small bowel ≥ 25 mm, no peristalsis | Surgery, CT | Emergency department and other | Taylor[126]  (2013) |
| **Gastrointestinal other** | | |  |  |  |  |  |  |  |
| X | Abd. Tuberculosis  879 (5) | 63.0 (43.0; 79.0) | 68.0 (42.0; 87.0) | Very low ⊕⊖⊖⊖ Very low ⊕⊖⊖⊖ | High | B-mode ultrasound of the abdomen | Bacteriological confirmation of M. tuberculosis |  | Van Hoving[127] (2019) |
| X | Acute Pancreatitis  421 (7) | 92.0 (88.0; 95.0) | 84.0 (78.0; 90.0) | Very low ⊝⊝⊝⊕  Very low ⊝⊕⊝⊝ | Low | Contrast-enhanced ultrasound (CEUS) | CT |  | Fei[128]  (2017) |
| X | Budd Chiari Syndrome  461 (6) | 89.0 (81.0; 94.0) | 86.0 (3.0; 99.0) | Very low ⊕⊕⊝⊝  Very low ⊕⊝⊝⊝ | Moderate | Doppler ultrasound | Surgery, catheter venography |  | Gupta[129]  (2020) |
| X | Diverticulitis  1420 (6) | 93.9 (92.0; 95.3) | 85.9 (72.0; 93.6) | High ⊕⊕⊕⊕  Low ⊕⊖⊕⊖ | Moderate | POCUS | CT, follow-up |  | Celik[130] (2025) |
|  | Diverticulitis  2999 (12) | 92.0 (86.0; 96.0) | 94.0 (88.0; 97) | Moderate ⊕⊕⊝⊕  Low ⊕⊝⊝⊕ | Moderate | B-mode ultrasound | Pathology, surgery, follow-up, CT | Emergency department and other | Vijfschagt[131]  (2024) |
| X | Gastric content  523 (9) | 95.0 (84.0; 99.0) | 88.0 (72.0; 95.0) | Very low ⊕⊝⊝⊝  Very low ⊕⊝⊝⊝ | Moderate | B-mode ultrasound of the antrum in supine and right | Randomized grouping, upper endoscopy |  | Pan[132]  (2024) |

^1^ “X” indicates the DTA pair selected as the representative for the condition presented in main Table 2 or 3. The selection followed a hierarchical order based on: (I) AMSTAR, (II) GRADE, and (III) emergency focus. ^2^ GRADE is detailed as i) Risk of Bias (⊝/⊕), ii) Consistency (⊝/⊕), iii) Directness (⊝/⊕), and iv) Precision (⊝/⊕).

**Abbreviations:** CI = confidence interval; CPPD = Calcium Pyrophosphate Dehydrate Deposition Disease; CT = computer tomography; DCS = double contour sign; DTA = diagnostic test accuracy; n = number: NA = not documented; POCUS = point of care ultrasound; RADUS = radiology performed ultrasound; Sens. = sensitivity; Spec. = specificity

## Table S8d: Summary of pooled *genitourinary* DTA pairs (index test vs reference standard), including GRADE certainty of evidence for sensitivity and specificity, AMSTAR 2 assessments, and information on the diagnostic procedure. For each POCUS indication, one DTA pair with pooled estimates from the included studies is presented.

| **Topic^1^**  n patient (n studies) | | **Sensitivity [%] (95% CI)** | **Specificity [%] (95% CI)** | **GRADE^2^ (Sens./Spec.)** | **Method. quality (AMSTAR 2)** | | **Index test** | **Reference standard** | | **Remark** | | **First author (year)** |
| --- | --- | --- | --- | --- | --- | --- | --- | --- | --- | --- | --- | --- |
| **Testicular torsion** | | |  |  |  |  | |  |  | |  | |
|  | Testicular torsion,  4422 (42) | 95.3 (91.4; 97.5) | 98.3 (96.2; 99.3) | Moderate ⊕⊖⊕⊕ Moderate ⊕⊖⊕⊕ | Moderate | | Color Doppler ultrasound | Surgery, follow-up | |  | | Alexander  [133](2025) |
|  | Testicular torsion,  147 (4) | 92.0  (70.0; 98.0) | 99.0  (95.0; 100.0) | Very low ⊝⊝⊝⊝  Low ⊝⊕⊝⊕ | Low | | Whirlpool sign in ultrasound | Not mentioned | |  | | McDowall  [134](2018) |
| X | Testicular torsion,  2116 (26) | 86.4 (79.4; 91.3) | 95.3 (92.3; 97.2) | Moderate ⊕⊕⊕⊝  High ⊕⊕⊕⊕ | Moderate | | Color Doppler ultrasound | Surgery / follow-up | | Color Doppler after 2010: 95% sensibility, 98% specificity | | Ota[135]  (2019) |
| **Urolithiasis** | | |  |  |  |  | |  |  | |  | |
|  | Urolithiasis,  4005 (21) | 88.2  (87.1; 89.2) | 79.2 (74.3; 84.3) | Low ⊝⊝⊕⊕  Low ⊝⊝⊕⊕ | Critically low | | Twinkling artefact | CT, DSV | | Power doppler: 64% sensitivity, 90% specificity | | Laher[136]  (2020) |
| X | Urolithiasis,  1773 (5) | 70.2 (67.1; 73.2) | 75.4  (72.5; 78.2) | Low ⊕⊝⊕⊝  High ⊕⊕⊕⊕ | Moderate | | Hydronephrosis in POCUS | CT, DSV, surgical finding | | Only high-quality studies included | | Wong[137]  (2018) |
| **Genitourinary other** | | |  |  |  |  | |  |  | |  | |
| X | Bladder outlet obstruction, 1204 (10) | 68.0  (56.0; 78.0) | 91.0  (82.0; 96.0) | Very low ⊝⊝⊝⊝  Very low ⊝⊝⊝⊕ | Critically low | | Detrusor wall thickness | Urodynamic pressure flow studies | |  | | Chen[138]  (2023) |
| X | AKIN,  2400 (23) | 76.0  (70.0; 81.0) | 79.0  (72.0; 85.0) | Very low ⊝⊝⊝⊝  Very low ⊝⊝⊝⊕ | Low | | Renal Resistive Index | Development AKIN | |  | | Wei[139]  (2022) |
| X | Hydronephrosis NA (8) | 81.7 (76.6; 86.9) | 74.1 (59.9; 88.3) | Moderate ⊕⊝⊕⊕ Low ⊕⊝⊕⊝ | Moderate | | Hydronephrosis in POCUS | CT, radiology performed ultrasound | | Emergency department | | Popat[110] (2024) |

^1^ “X” indicates the DTA pair selected as the representative for the condition presented in main Tables 2 or 3. For conditions with multiple eligible DTA pairs, selection followed a predefined hierarchy prioritizing: (i) the highest AMSTAR rating, (ii) the highest GRADE certainty, and (iii) the strongest emergency department focus. ^2^ GRADE is detailed as i) Risk of Bias (⊝/⊕), ii) Consistency (⊝/⊕), iii) Directness (⊝/⊕), and iv) Precision (⊝/⊕).

**Abbreviations**: AKIN = acute kidney injury; CI = confidence interval; CT = computer tomography; DSV = direct stone visualization; DTA = diagnostic test accuracy; n = number: POCUS = point of care ultrasound; Sens. = sensitivity; Spec. = specificity

## Table S8e: Summary of pooled *gynecological* DTA pairs (index test vs reference standard), including GRADE certainty of evidence for sensitivity and specificity, AMSTAR 2 assessments, and information on the diagnostic procedure. For each POCUS indication, one DTA pair with pooled estimates from the included studies is presented.

| **Topic^1^**  n patient (n studies) | | **Sensitivity [%] (95% CI)** | **Specificity [%] (95% CI)** | **GRADE^2^ (Sens./Spec.)** | **Method. quality (AMSTAR 2)** | **Index test** | **Reference standard** | **Remark** | **First author (year)** |
| --- | --- | --- | --- | --- | --- | --- | --- | --- | --- |
| **Pregnancy** | |  |  |  |  |  |  |  |  |
| X | Cesarian scar pregnancy  713 (9) | 83.0 (77.0; 87.0) | 74.0 (63.0; 83.0) | Low ⊕⊕⊖⊕ Very low ⊕⊕⊖⊖ | Low | Transvaginal ultrasound | Surgery, follow-up | Women with abdominal pain or vaginal bleeding during early pregnancy | Han[140]  (2025) |
|  | Ectopic pregnancy  6885 (3) | 88.0 (52.0; 69.0) | 99.0 (96.0; 100) | Very low ⊖⊖⊖⊖  Very low ⊖⊖⊖⊕ | Low | Transvaginal ultrasound | Surgery, follow-up | Women with abdominal pain or vaginal bleeding during early pregnancy | Crochet[141] (2013) |
| X | Ectopic pregnancy  1023 (7) | 45.0 (34.0; 56.0) | 96.0 (93.0; 98.0) | Very low ⊕⊝⊝⊝  Moderate ⊕⊕⊝⊕ | Moderate | Transabdominal or -vaginal ultrasound (adnexal mass and free fluid) | Surgery, final diagnosis | Women with pain and vaginal bleeding or positive urinary pregnancy test, but without a living embryo located outside the uterus | Richardson[142] (2016) |
|  | Ectopic pregnancy  2057 (10) | 97.0 (92.0; 99.0) | 76.0 (60.0; 80.0) | Moderate ⊖⊕⊕⊕  Very low ⊖⊖⊕⊖ | Low | Transabdominal or -vaginal ultrasound | Surgery, final diagnosis |  | Stein[143] (2010) |
| X | Intrauterine pregnancy  1716 (7) | 86.0  (76.0; 93.0) | 98.0 (87.0; 100) | Low ⊕⊖⊕⊖ Moderate ⊕⊕⊕⊖ | Moderate | POCUS by emergency physician | Ultrasound by obstetrician or radiologist | Emergency department | Celik[144]  (2026) |
|  | Retained products of conception  3268 (15) | 72.0 (67.0; 77.0) | 86.0 (82.0; 89.0) | Very low ⊖⊖⊖⊕ Low ⊖⊕⊖⊕ | Low | Transabdominal or -vaginal Cultrasound (Endometrial thickness) | Pathology |  | Li[145] (2025) |
| X | Retained products of conception  1237 (9) | 91.5 (84.4; 95.5) | 84.3 (61.5; 94.7) | Very low ⊕⊖⊖⊖  Very low ⊕⊖⊖⊖ | High | Transvaginal ultrasound (echogenic mass) | Pathology |  | Sundararajan[146] (2024) |
| **Adnexal torsion** | |  |  |  |  |  |  |  |  |
|  | Adnexal torsion  1742 (14) | 53.0 (34.0; 72.0) | 95.0 (86.0; 98.0) | Very low ⊖⊖⊖⊖ Very low ⊖⊖⊖⊖ | Low | Ultrasound (transvaginal, transrectal, abdominal) using ovarian doppler-flow | Surgery |  | Garde[147]  (2023) |
|  | Adnexal torsion  1187 (12) | 79.0 (63.0; 92.0) | 76.0 (54.0; 93.0) | Very low ⊖⊖⊖⊖  Very low ⊖⊖⊖⊕ | Critically low | Whirlpool sign, enlarged ovary, mass, free fluid, unclear if transabdominal or transvaginal | Clinical analysis, clinical or radiological follow-up | No quality assessment | Shakeel[148] (2021) |
| X | Adnexal torsion  1187 (12) | 79.0 (63.0; 92.0) | 76.0 (54.0; 93.0) | Low ⊕⊖⊕⊖  Low ⊕⊖⊕⊖ | Moderate | Whirlpool sign, enlarged ovary, mass, free fluid, unclear if transabdominal or transvaginal | Surgical exploration with / without follow up |  | Wattar[149] (2021) |
| X | Ovarian torsion  1023 (7) | 82.0 (78.0; 86.0) | 81.0  (70.0; 90.0) | Very low ⊖⊖⊖⊖ Very low ⊖⊕⊖⊖ | Low | Transabdominal or -vaginal ultrasound (Whirlpool sign) | Surgery, follow-up | Children and women (including pregnants) | Adu-Bredu[150] (2021) |

^1^ “X” indicates the DTA pair selected as the representative for the condition presented in main Tables 2 or 3. For conditions with multiple eligible DTA pairs, selection followed a predefined hierarchy prioritizing: (i) the highest AMSTAR rating, (ii) the highest GRADE certainty, and (iii) the strongest emergency department focus. ^2^ GRADE is detailed as i) Risk of Bias (⊝/⊕), ii) Consistency (⊝/⊕), iii) Directness (⊝/⊕), and iv) Precision (⊝/⊕).

**Abbreviations:** CI = confidence interval; DTA = diagnostic test accuracy; n = number: NA = not documented; POCUS = point of care ultrasound; Sens. = sensitivity; Spec. = specificity

## Table S8f: Summary of pooled *interventional* DTA pairs (index test vs reference standard), including GRADE certainty of evidence for sensitivity and specificity, AMSTAR 2 assessments, and information on the diagnostic procedure. For each POCUS indication, one DTA pair with pooled estimates from the included studies is presented.

| **Topic^1^**  n patient (n studies) | | **Sensitivity [%] (95% CI)** | | **Specificity [%] (95% CI)** | | **GRADE^2^ (Sens./Spec.)** | | **Method. quality (AMSTAR 2)** | | | **Index test** | **Reference standard** | | | **Remark** | **First author (year)** | |
| --- | --- | --- | --- | --- | --- | --- | --- | --- | --- | --- | --- | --- | --- | --- | --- | --- | --- |
| **Airway related** | | |  | |  | |  | |  |  | | |  |  | | |  |
|  | Endotracheal tube  2588 (30) | 91.0  (82.0; 95.0) | | 98.0  (96.0; 99.0) | | Low ⊕⊖⊕⊖  Moderate ⊕⊖⊕⊕ | | Moderate | | | Transtracheal ultrasound, emergency intubations | Capnography, chest auscultation, chest X-ray | | |  | Alsabri[151]  (2026) | |
|  | Endotracheal tube  665 (9) | 88.0  (76.0; 94.0) | | 98.0 (96.0; 99.0) | | Moderate ⊕⊕⊕⊖  High ⊕⊕⊕⊕ | | Moderate | | | Transtracheal ultrasound, emergency intubations | Capnography, chest auscultation, chest X-ray | | | Emergency department | Chou[152]  (2015) | |
|  | Endotracheal tube  713 (8) | 98.0  (97.0; 99.0) | | 94.0  (86.0; 98.0) | | High ⊕⊕⊕⊕  Moderate ⊕⊕⊕⊖ | | Moderate | | | Transtracheal ultrasound, emergency intubations | Capnography | | |  | Das[153]  (2015) | |
|  | Endotracheal tube  1152 (13) | 98.7  (97.6; 99.3) | | 96.0  (89.8; 98.5) | | High ⊕⊕⊕⊕  Moderate ⊕⊖⊕⊕ | | Moderate | | | Transtracheal ultrasound | Visualization, capno-graphy, auscultation | | | Emergency physicians performed ultrasound | Gottlieb[154]  (2018) | |
| X | Endotracheal tube  2534 (20) | 98.0  (97.0; 99.0) | | 95.0  (90.0; 100) | | High ⊕⊕⊕⊕  High ⊕⊕⊕⊕ | | Moderate | | | POCUS, any sign | Capnography or fiberoptic bronchoscopy | | | Emergency physicians performed ultrasound | Sahu[155]  (2020) | |
|  | Endotracheal tube  2112 (8) | 78.0 (60.0; 89.0) | | 81.0 (63.0; 91.0) | | Low ⊕⊕⊖⊖ Low ⊕⊕⊖⊖ | | Moderate | | | B-mode ultrasound (distance from skin to epiglottis) | Cormack Lehane score, Endotracheal tube | | | Pre- and intraoperative | Soni[156] (2025) | |
|  | Endotracheal tube  617 (9) | 93.0  (87.0; 96.0) | | 75.0  (57.0; 87.0) | | Moderate ⊕⊖⊕⊕  Low ⊕⊖⊕⊖ | | Moderate | | | LUS | Fiberoptic bronchoscopy, chest radiography | | | Adults and children | Yang[157]  (2022) | |
|  | Pred. diff. intubation  544 (4) | 61.0  (45.0; 75.0) | | 88.0  (77.0; 94.0) | | Very low ⊕⊖⊖⊖  Very low ⊕⊖⊖⊖ | | Moderate | | | Hyomental-distance ratio | Direct laryngoscopy (Cormack-Lehane ≥3) | | | Obese patients in 3 studies excluded | Benavides[158]  (2023) | |
| X | Pred. diff. intubation  NA (23) | 76.0  (71.0; 81.0) | | 77  (72.0; 82.0) | | Low ⊕⊖⊖⊕  Low ⊕⊖⊖⊕ | | Moderate | | | Ultrasound anterior neck soft tissue thickness domain | Direct laryngoscopy (Cormack-Lehane ≥3) | | | Most studies exclude morbidly obese patients | Bhargava[159]  (2023) | |
|  | Pred. diff. intubation  2097 (15) | 82.0  (74.0; 87.0) | | 79  (70.0; 86.0) | | Very low ⊕⊖⊖⊖  Very low ⊕⊖⊖⊖ | | Moderate | | | Upper airway US, distance from skin to epiglottis | Direct laryngoscopy (Cormack-Lehane ≥3) | | | Obese patients excluded in some studies | Carsetti [160]  (2022) | |
|  | Pred. diff. intubation  3485 (8) | 69.0  (63.0; 74.0) | | 84.0  (82.0; 85.0) | | Very low ⊕⊖⊖⊖  Low ⊕⊖⊖⊕ | | Moderate | | | Upper airway US, multiple signs | Direct laryngoscopy (Cormack-Lehane ≥3) | | |  | Ji[161]  (2018) | |
| **Central venous catheter placement** | | | | | | |  | |  |  | | |  |  | | |  |
| X | CVC placement  1469 (13) | 82.0  (77; 86) | | 98.0  (97.0; 99.0) | | Moderate ⊕⊖⊕⊕  Moderate ⊕⊖⊕⊕ | | Moderate | | | Cardiac and vascular ultrasound and/or CEUS | Chest radiography | | |  | Ablordeppey[162]  (2017) | |
|  | CVC placement  572 (5) | 72.0  (44.0; 91.0) | | 100  (99.0; 100) | | Low ⊖⊕⊕⊖  Moderate ⊖⊕⊕⊕ | | Low | | | CEUS | Chest radiography | | |  | Bou Chebl[163]  (2017) | |
|  | CVC placement  2548 (25) | 68.2  (54.4; 79.4) | | 98.9  (97.8; 99.4) | | Low ⊕⊖⊕⊖  Moderate ⊕⊖⊕⊕ | | Moderate | | | Vascular ultrasound, TTE and/or CEUS | Chest radiography, TEE +/- intrafluoroscopy | | | Malposition wasn’t defined equally | Smit[164]  (2018) | |
|  | CVC placement  801 (8) | 81.0 (50.0; 95.0) | | 99.0 (97.0; 99.0) | | Moderate ⊕⊕⊕⊖ Moderate ⊕⊖⊕⊕ | | Moderate | | | Vascular ultrasound, TTE, LUS and/or CEUS | Chest X-ray, CT | | | Emergency department | Zhu[165]  (2026) | |
| **Gastric tube placement** | | | | | | |  | |  |  | | |  |  | | |  |
| X | Nasogastric tube placement 420 (5) | 93.0 (87.0; 97.0) | | 97.0 (23.0; 100) | | High ⊕⊕⊕⊕  Low ⊕⊖⊕⊖ | | Moderate | | | Ultrasound of stomach, neck, and esophagus | Chest or abdominal radiography | | |  | Lin[166]  (2017) | |
|  | Nasogastric tube placement 3 (168) | 93.0 (88.0; 97.0) | | 88.0 (47.0; 100) | | Moderate ⊕⊖⊕⊕  Low ⊕⊖⊕⊖ | | Moderate | | | Ultrasound of stomach, neck, and esophagus | Chest or abdominal radiography | | | Adults and children | Peng[167]  (2022) | |

^1^ “X” indicates the DTA pair selected as the representative for the condition presented in main Tables 2 or 3. For conditions with multiple eligible DTA pairs, selection followed a predefined hierarchy prioritizing: (i) the highest AMSTAR rating, (ii) the highest GRADE certainty, and (iii) the strongest emergency department focus. ^2^ GRADE is detailed as i) Risk of Bias (⊝/⊕), ii) Consistency (⊝/⊕), iii) Directness (⊝/⊕), and iv) Precision (⊝/⊕).

**Abbreviations:** NA = not documented; CI = confidence interval; CVC = central venous catheter; DTA = diagnostic test accuracy; n = number; Sens. = sensitivity; Spec. = specificity; US = ultrasound; ED = emergency department; POCUS = point of care ultrasound; LUS = lung ultrasound; CEUS = contrast-enhanced ultrasound

## Table S8g: Summary of pooled *musculoskeletal* DTA pairs (index test vs reference standard), including GRADE certainty of evidence for sensitivity and specificity, AMSTAR 2 assessments, and information on the diagnostic procedure. For each POCUS indication, one DTA pair with pooled estimates from the included studies is presented.

| **Topic^1^**  n patient (n studies) | | **Sensitivity [%] (95% CI)** | **Specificity [%] (95% CI)** | **GRADE^1^ (Sens./Spec.)** | **Method. quality (AMSTAR 2** | **Index test** | **Reference standard** | **Remark** | **First author (year)** |
| --- | --- | --- | --- | --- | --- | --- | --- | --- | --- |
| **Crystal arthropathies** | |  |  |  |  |  |  |  |  |
| X | CPPD,  NA (5) | 89.0 (72.0; 97.0) | 94.0 (87.0; 98.0) | Very low ⊝⊝⊝⊝  Very low ⊝⊝⊝⊝ | Low | Ultrasound of fibrocartilage or hyaline cartilage | Synovial fluid analysis, microscopic analysis | Only high-quality studies included | Filippou[168] (2016) |
|  | CPPD  597 (8) | 87.9 (80.9, 94.9) | 91.5 (85.5, 97.2) | Very low ⊝⊝⊝⊝  Very low ⊝⊝⊝⊕ | Critically low | B-mode and color Doppler ultrasound | Monosodium urate crystal in synovial fluid analysis |  | Gamon[169]  (2015) |
|  | Gout  299 (6) | 93.0 (90.0; 96.0) | 73.0  (67.0; 78.0) | Very low ⊝⊝⊝⊝  Very low ⊝⊝⊝⊕ | Low | Double contour sign, tophus | Monosodium urate crystal in synovial fluid analysis |  | Chen[170]  (2017) |
| X | Gout  1726 (11) | 65.1 (62.0; 68.2) | 89.0 (86.6; 91.1) | Very low ⊕⊝⊝⊝  Low ⊕⊝⊝⊕ | Moderate | Double contour sign | Monosodium urate crystal in synovial fluid analysis | Tophus: 54% sensitivity, 93% specificity | Lee[171]  (2018) |
|  | Gout  363 (5) | 83.0 (72.0, 91.0) | 76.0 (68.0, 83.0) | Very low ⊝⊝⊝⊝  Very low ⊝⊝⊝⊝ | Low | Double contour sign | Monosodium urate crystal in synovial fluid analysis | Tophus: 65% sensitivity, 80% specificity | Ogdie[172]  (2015) |
|  | Gout  NA (11) | 70.0 (58.0; 79.0) | 95.0  (87.0; 98.0) | Very low ⊝⊝⊝⊝  Very low ⊝⊝⊝⊝ | Critically low | Double contour sign | Monosodium urate crystal in synovial fluid analysis | Double contour sign + tophus: 84% sensitivity; 84% specificity | Shang[173]  (2022) |
|  | Gout  1363 (7) | 60.0 (35.0, 82.0) | 94.0  (89.0, 97.0) | Very low ⊝⊝⊝⊝  Very low ⊝⊝⊝⊕ | Low | Tophus | Monosodium urate crystal in synovial fluid analysis | Any ultrasound feature: 90% sensitivity; 84% specificity | Stewart[174]  (2021) |
|  | Gout  1272 (6) | 86.4 (79.4; 91.3) | 95.3 (92.3; 97.2) | Very low ⊝⊝⊝⊝  Very low ⊝⊝⊝⊕ | Low | Double contour sign | Monosodium urate crystal in synovial fluid analysis | Person-based vs joint-/location-based assessment | Zhang[175]  (2018) |
|  | **Vasculitis** |  |  |  |  |  |  |  |  |
|  | Giant cell arteritis  2766 (31) | 83.0 (79.0; 87.0) | 88.0 (84.0; 91.0) | Low ⊕⊝⊝⊕  Low ⊕⊝⊝⊕ | Moderate | Color Doppler (different signs) | Temporal artery biopsy and clinical diagnosis |  | El Yaman[176]  (2026) |
| X | Giant cell arteritis  1479 (15) | 78.0 (74.6; 81.3) | 91.0  (89.0; 92.9) | Low ⊕⊝⊝⊕  Low ⊕⊝⊝⊕ | High | Halo sign  (temporal artery ultrasound) | Clinical diagnosis |  | Pouncey[177]  (2024) |
|  | Giant cell arteritis  1061 (11) | 78.0  (64.0, 87.0) | 79.0  (73.0, 85.0) | Very low ⊝⊝⊝⊝  Very low ⊝⊝⊝⊝ | Low | Any ultrasound sign  (temporal artery ultrasound) | Temporal artery biopsy |  | Rinagel[178]  (2019) |
|  | Giant cell arteritis  1502 (18) | 67.0 (51.0; 80.0) | 95.0  (89.0; 98.0) | Very low ⊕⊝⊝⊝  Low ⊕⊝⊝⊕ | Moderate | Halo sign  (temporal artery ultrasound) | Clinical diagnosis or Temporal artery biopsy |  | Sebastian[179]  (2021) |
|  | Large vessel vascul.  605 (8) | 77.0  (62.0; 87.0) | 96.0  (85.0; 99.0) | Very low ⊝⊝⊝⊝  Very low ⊝⊝⊝⊝ | Low | Halo sign  (temporal artery ultrasound) | Clinical diagnosis |  | Duftner[180]  (2021) |
| X | Large vessel vascul.  584 (8) | 88.0 (82.0; 92.0) | 96.0 (86.0; 99.0) | Very low ⊝⊝⊝⊕  Very low ⊝⊝⊝⊕ | Low | Halo sign with/without compression (axillary, carotid, facial and mostly temporal) | Clinical diagnosis or temporal artery biopsy | Only low risk of bias studies | Bosch[181] (2023) |
|  | **Musculoskeletal other** | |  |  |  |  |  |  |  |
|  | Abscess  NA (5) | 94.8 (90.6; 98.9) | 85.2 (72.2; 98.1) | Moderate ⊝⊕⊕⊕  Low ⊝⊕⊕⊝ | Moderate | POCUS | Needle aspiration or incision-drainage | Emergency department | Popat[110]  (2024) |
| X | Abscess  747 (8) | 96.2 (91.1; 98.4) | 82.9 (60.4; 93.9) | High ⊕⊕⊕⊕  Low ⊕⊝⊕⊝ | Moderate | POCUS | CT, purulent discharge, follow-up | Emergency department | Barbic[182] (2017) |
| X | Knee effusions  381 knees (8) | 84.0 (78.0, 89.0) | 93.0 (73.0, 99.0) | Low ⊕⊝⊕⊝  Low ⊕⊝⊕⊝ | Moderate | B-mode Ultrasound for synovial effusion | MRI | Many pediatric patients included as well | Meyer[183]  (2022) |
| X | Necrotising skin inf.  2522 (13) | 93.2 (90.3; 96.2) | 87.9 (84.4; 91.1) | Low ⊖⊖⊕⊕  Low ⊖⊖⊕⊕ | Critically low | POCUS | Surgery |  | Götz[184]  (2023) |
| X | Ret. foreign bodies  NA (17) | 72.0 (57.0; 83.0) | 92.0  (88.0; 95.0) | Very low ⊖⊖⊕⊖  Low ⊖⊖⊕⊕ | Low | B-mode ultrasound | CT, surgery, follow-up |  | Davis[185]  (2015) |
| X | Rotator cuff tear  NA (29) | 80.0  (78.0, 83.0) | 85.0  (82.0, 87.0) | Low ⊝⊕⊕⊝  Low ⊝⊕⊕⊝ | Moderate | Signs of rotator cuff tear in B-mode ultrasound | Arthrography, arthroscopy, surgery, combination, other | Full-thickness tear: 87% sensitivity, 96% specificity | Dinnes[186]  (2003) |
| X | Synovial abnorm.  640 knees (8) | 90.0 (81.0; 95.0) | 86.0 (77.0; 92.0) | Very low ⊕⊝⊝⊝  Very low ⊕⊝⊝⊝ | Moderate | B-mode Ultrasound for synovial effusion | MRI | Pleuritis: 88% sensitivity; 70% specificity | Liu[187]  (2024) |

^1^ “X” indicates the DTA pair selected as the representative for the condition presented in main Tables 2 or 3. For conditions with multiple eligible DTA pairs, selection followed a predefined hierarchy prioritizing: (i) the highest AMSTAR rating, (ii) the highest GRADE certainty, and (iii) the strongest emergency department focus. ^2^ GRADE is detailed as i) Risk of Bias (⊝/⊕), ii) Consistency (⊝/⊕), iii) Directness (⊝/⊕), and iv) Precision (⊝/⊕).

**Abbreviations:** CI = confidence interval; CPPD = Calcium Pyrophosphate Dehydrate Deposition Disease; CT = computer tomography; DTA = diagnostic test accuracy; n = number: NA = not documented; POCUS = point of care ultrasound; Sens. = sensitivity; Spec. = specificity

## Table S8h: Summary of pooled *neurological* DTA pairs (index test vs reference standard), including GRADE certainty of evidence for sensitivity and specificity, AMSTAR 2 assessments, and information on the diagnostic procedure. For each POCUS indication, one DTA pair with pooled estimates from the included studies is presented.

| **Topic^1^**  n patient (n studies) | | **Sensitivity [%] (95% CI)** | **Specificity [%] (95% CI)** | **GRADE^2^ (Sens./Spec.)** | **Method. quality (AMSTAR 2)** | **Index test** | **Reference standard** | **Remark** | **First author (year)** |
| --- | --- | --- | --- | --- | --- | --- | --- | --- | --- |
| **Artery stenosis** | | | | | |  |  |  |  |
|  | Carotid artery  1007 (5) | 97.0 (95.0; 98.0) | 70.0 (67.0; 73.0) | Moderate ⊕⊕⊖⊕  Low ⊕⊕⊖⊖ | High | Doppler ultrasound | DSA | > 50% stenosis | Cassola[188] (2022) |
| X | Carotid artery  2770 (9) | 85.0  (77.0; 91.0) | 98.0 (74.0; 90.0) | Very low ⊕⊖⊖⊖  Very low ⊕⊖⊖⊖ | High | Doppler ultrasound | DSA | > 70% stenosis | Cassola[188] (2022) |
|  | Carotid artery  NA (8) | 99.0 (83.0; 100) | 97.0 (90.0; 99.0) | Low ⊕⊕⊖⊖  Moderate ⊕⊕⊖⊕ | Moderate | CEUS | Surgery, Angiography, DSA, CTA | Occlusion, extracranial High applicability concerns | Liu[189] (2023) |
|  | Carotid artery  62 (NA) | 36.0 (25.0; 49.0) | 91.0  (87.0; 94.0) | Very Low ⊖⊖⊖⊖  Very low ⊖⊖⊖⊕ | Low | Doppler ultrasound | IAA | 50 - 69% stenosis | Wardlaw[190] (2006) |
|  | Carotid artery 316 (11) | 89.0 (85.0; 92.0) | 84.0  (77.0; 89.0) | Low ⊖⊕⊖⊕  Very low ⊖⊕⊖⊖ | Low | Doppler ultrasound | IAA | > 70% stenosis | Wardlaw[190] (2006) |
|  | Intracranial artery 812 (12) | 96.0 (88.0; 99.0) | 94.0 (82.0; 98.0) | Very low ⊕⊖⊖⊖  Very low ⊕⊖⊖⊖ | Moderate | Transcranial Doppler and color-coded Duplex | IA, CTA, MRA | Adults with acute ischaemic stroke | Liu[191] (2023) |
| X | Intracranial artery 493 (9) | 95.0 (83.0; 99.0) | 95.0 (90.0; 98.0) | Very low ⊕⊖⊖⊖  Moderate ⊕⊕⊖⊕ | High | Transcranial Doppler and color-coded Duplex | IA, CTA, MRA | Adults with acute ischaemic stroke | Mattioni[192] (2020) |
|  | Intracranial artery 1496 (17) | 90.0 (82.0; 95.0) | 95.0 (91.0; 98.0) | Very low ⊖⊖⊖⊖ Low ⊖⊖⊖⊕ | Low | Transcranial Doppler and color-coded Duplex | IA, CTA, MRA |  | Zhu[193] (2025) |
| X | Large vessel occlusion  1343 (6) | 87.5 (79.0; 92.8) | 95.0  (91.8; 97.0) | Very low ⊕⊖⊖⊖ Low ⊕⊕⊖⊖ | Moderate | Transcranial Doppler and color-coded Duplex | CTA, MRA | Adults with acute ischaemic stroke | Jazayeri[194] (2025) |
| X | Stroke (Ischemic and hemorrhagic)  802 (13) | 81.1 (73.1; 87.2) | 85.5 (71.4; 93.3) | Very low ⊖⊖⊖⊖ Very low ⊖⊖⊖⊖ | Low | Transcranial Doppler and color-coded Duplex | MRA, DSA, CTA |  | Pinto-Villalba[195] (2026) |
| X | Vertebral artery  322 arteries (2) | 70.2 (54.2; 83.3) | 97.7 (95.2; 99.1) | Very low ⊖⊕⊖⊖  Very low ⊖⊖⊖⊕ | Low | Color Doppler ultrasound | IAA | > 50% stenosis No formal bias assessment | Khan[196] (2007) |
|  | Vertebral artery  269 arteries (3) | 70.2 (56.6; 81.6) | 93.4 (89.2; 96.1) | Very low ⊖⊕⊖⊖  Very low ⊖⊖⊖⊕ | Low | Doppler ultrasound | IAA | > 50% stenosis No formal bias assessment | Khan[196] (2007) |
| **Raised intracranial pressure** | | |  |  |  |  |  |  |  |
|  | Intracranial pressure 619 (16) | 90.0 (85.0; 94.0) | 85.0 (80.0; 89.0) | Low ⊖⊕⊖⊕  Low ⊖⊕⊖⊕ | Low | Optic nerve sheath diameter | Invasive measurements of ICP | ICU | Aletreby[197] (2022) |
|  | Intracranial pressure  779 (14) | 85.5 (77.9; 90.8) | 90.7 (84.6; 94.5) | Very low ⊖⊖⊖⊖ Very low ⊖⊖⊖⊕ | Low | Optic nerve sheath diameter and/or optic disc elevation | Follow-up | Case-control studies | Bakola[198] (2024) |
|  | Intracranial pressure 222 (8) | 82.0 (75.0; 88.0) | 82.0 (71.0; 90.0) | Very low ⊖⊕⊖⊖  Very low ⊖⊕⊖⊖ | Low | Optic nerve sheath diameter | Invasive measurements of ICP | Traumatic brain injury | Chen[199]  (2023) |
| X | Intracranial pressure 4551 (71) | 94.0 (91.0; 96.0) | 87.0  (82.0; 91.0) | Low ⊕⊖⊖⊕  Low ⊕⊖⊖⊕ | High | Optic nerve sheath diameter | CT, ICP Monitor, Lumbar puncture | Traumatic and atraumatic brain injury | Koziarz[200] (2019) |
|  | Intracranial pressure 1484 (18) | 92.0 (85.0; 95.0) | 90.0 (84.0; 94.0) | Moderate ⊕⊕⊖⊕ Moderate ⊕⊕⊖⊕ | Moderate | Optic nerve sheath diameter | CT, MRI, ICP Monitor, Lumbar puncture |  | Melo[201]  (2025) |
|  | Intracranial pressure 3213 (42) | 87.7 (83.1; 91.5) | 86.3 (81.9; 90.1) | Moderate ⊕⊖⊕⊕ Moderate ⊕⊖⊕⊕ | Moderate | Optic nerve sheath diameter | Invasive measurements of ICP, lumbar punction | 5.1 mm defined as threshold | Wang[202] (2026) |
|  | Intracranial pressure 512 (10) | 85.0 (79.0; 89.0) | 88.0 (80.0; 93.0) | Moderate ⊕⊕⊖⊕  Low ⊕⊕⊖⊖ | Moderate | Optic nerve sheath diameter | Invasive measurements of ICP | ICU, traumatic brain injury | Xu[203] (2024) |
| **Neurology other** | | |  |  |  |  |  |  |  |
| X | Cauda equina syndrome 3 (487) | 82.3 (72.0; 90.0) | 65.0 (60.0; 70.0) | Very low ⊖⊖⊕⊖  Low ⊖⊕⊕⊖ | Low | Post-void residual volume (200ml as threshold) | CT, MRI | ICU, neurology, emergency department | Boktor[204] (2023) |
| X | Intracranial hemorrhage 803 (18) | 88.0  (74.0; 100) | 81.0 (50.0; 100) | Low ⊕⊖⊕⊖  Low ⊕⊖⊕⊖ | Moderate | Transcranial Ultrasound | CT, MRI | ICU, neurology, emergency department | Allen[205] (2023) |
| X | Intracranial masses 113 (2) | 97.0 (92.0; 100) | 99.0 (96.0; 100) | Low ⊕⊖⊖⊕  Low ⊕⊖⊖⊕ | Moderate | Transcranial Ultrasound | CT, MRI | ICU, neurology, emergency department | Allen[205] (2023) |
| X | Neurodegenerative 1402 (20) | 80.0  (72.0; 89.0) | 71.0  (59.0; 82.0) | Very low ⊕⊖⊖⊖  Very low ⊕⊖⊖⊖ | Moderate | Transcranial Ultrasound | PET, SPECT, CT, MRI | ICU, neurology, emergency department | Allen[205] (2023) |

^1^ “X” indicates the DTA pair selected as the representative for the condition presented in main Tables 2 or 3. For conditions with multiple eligible DTA pairs, selection followed a predefined hierarchy prioritizing: (i) the highest AMSTAR rating, (ii) the highest GRADE certainty, and (iii) the strongest emergency department focus. ^2^ GRADE is detailed as i) Risk of Bias (⊝/⊕), ii) Consistency (⊝/⊕), iii) Directness (⊝/⊕), and iv) Precision (⊝/⊕).

**Abbreviations:** ATFL = Anterior Talofibulare ligament; CEUS = Contrast Enhanced Ultrasound; CT = Computer Tomography; CTA = Computer Tomography Angiography; DSA = Digital Subtraction Angiography; DTA = diagnostic test accuracy; IA = Intracranial Angiography; IAA = Intra-arterial Angiography; ICU = Intensive Care Unit), l = long; MRA = Magnetic Resonance Angiography; MRI = Magnetic Resonance Imaging; n = number: NA = Not documented; path = pathology; PLC = Posterior ligamentous complex; POCUS = Point Of Care Ultrasound; Sens. = Sensitivity; Spec. = Specificity

## Table S8i: Summary of pooled *ocular* DTA pairs (index test vs reference standard), including GRADE certainty of evidence for sensitivity and specificity, AMSTAR 2 assessments, and information on the diagnostic procedure. For each POCUS indication, one DTA pair with pooled estimates from the included studies is presented.

| **Topic^1^**  n patient (n studies) | | **Sensitivity [%] (95% CI)** | **Specificity [%] (95% CI)** | **GRADE^2^ (Sens./Spec.)** | **Method. quality (AMSTAR 2)** | **Index test** | **Reference standard** | **Remark** | **First author (year)** |
| --- | --- | --- | --- | --- | --- | --- | --- | --- | --- |
| **Retinal detachment** | | |  |  |  |  |  |  |  |
|  | Retinal detachment  517 (6) | 92.0 (67.2; 98.5) | 91.4 (84.9; 95.3) | Low ⊕⊖⊕⊖  Low ⊕⊖⊕⊖ | Moderate | POCUS of the eye | Ophthalmologist exam, CT |  | Gottlieb[206] (2019) |
| X | Retinal detachment 1189 (9) | 94.0 (88.0; 97.0) | 94.0 (85.0; 98.0) | Moderate ⊕⊖⊕⊕  Low ⊕⊖⊕⊖ | Moderate | POCUS of the eye | Ophthalmologist exam | Sensitivity analysis without significant change | Propst[207]  (2019) |
| **Ocular other** | | |  |  |  |  |  |  |  |
| X | Foreign body  400 (2) | 100  (81.0; 100) | 99.0 (99.0; 100) | Low ⊕⊖⊕⊖  Moderate ⊕⊖⊕⊕ | Moderate | POCUS of the eye | Ophthalmologist exam |  | Propst[207]  (2019) |
| X | Globe rupture  400 (2) | 100 (63.0; 100) | 99.0 (99.0; 100) | Low ⊕⊖⊕⊖  Moderate ⊕⊖⊕⊕ | Moderate | POCUS of the eye | Ophthalmologist exam |  | Propst[207]  (2019) |
| X | Lens dislocation  400 (2) | 97.0  (83.0; 99.0) | 99.0 (97.0; 100) | Low ⊕⊖⊕⊖  Moderate ⊕⊖⊕⊕ | Moderate | POCUS of the eye | Ophthalmologist exam |  | Propst[207]  (2019) |
| X | Posterior disorder 1281 (10) | 96.0 (91.0; 98.0) | 94.0 (87.0; 98.0) | Moderate ⊕⊖⊕⊕ Low ⊕⊖⊕⊖ | Moderate | B-mode ultrasound | Direct ophthalmoscopy |  | Wu[208] (2025) |
| X | Vitreous detachment  388 (4) | 67.0  (51.0; 81.0) | 89.0 (53.0; 98.0) | Low ⊕⊖⊕⊖  Low ⊕⊖⊕⊖ | Moderate | POCUS of the eye | Ophthalmologist exam |  | Propst[207]  (2019) |
| X | Vitreous haemorrhage  739 (5) | 90.0 (65.0; 98.0) | 92.0  (75.0; 98.0) | Low ⊕⊖⊕⊖  Low ⊕⊖⊕⊖ | Moderate | POCUS of the eye | Ophthalmologist exam |  | Propst[207]  (2019) |

^1^ “X” indicates the DTA pair selected as the representative for the condition presented in main Tables 2 or 3. For conditions with multiple eligible DTA pairs, selection followed a predefined hierarchy prioritizing: (i) the highest AMSTAR rating, (ii) the highest GRADE certainty, and (iii) the strongest emergency department focus. ^2^ GRADE is detailed as i) Risk of Bias (⊝/⊕), ii) Consistency (⊝/⊕), iii) Directness (⊝/⊕), and iv) Precision (⊝/⊕).

**Abbreviations:** CI = confidence interval; DTA = diagnostic test accuracy; n = number; POCUS = point of care ultrasound; Sens. = sensitivity; Spec. = specificity

## Table S8j: Summary of pooled *respiratory* DTA pairs (index test vs reference standard), including GRADE certainty of evidence for sensitivity and specificity, AMSTAR 2 assessments, and information on the diagnostic procedure. For each POCUS indication, one DTA pair with pooled estimates from the included studies is presented.

| **Topic^1^**  n patient (n studies) | | **Sensitivity [%] (95% CI)** | **Specificity [%] (95% CI)** | **GRADE^2^ (Sens./Spec.)** | **Method. quality (AMSTAR 2)** | **Index test** | **Reference standard** | **Remark** | **First author (year)** |
| --- | --- | --- | --- | --- | --- | --- | --- | --- | --- |
| **Disorders of the pleural space** | | |  |  |  |  |  |  |  |
| X | Hemothorax  451 (3) | 81.0  (71.0; 88.0) | 99.0  (96.0; 100.0) | Moderate ⊕⊕⊕⊖  High ⊕⊕⊕⊕ | Moderate | Lung ultrasound (anechoic area, free fluid) | CT, chest X-ray, chest tube observation |  | Staub[209]  (2018) |
| X | Iatrog. Pneumothorax  281 (2) | 93.0 (84.0; 97.0) | 97.0 (93.0; 99.0) | Very low ⊖⊖⊕⊖  Low ⊖⊖⊕⊕ | Low | Lung ultrasound | CT, release of air on chest tube insertion | Radiologists | Alrajhi[210]  (2012) |
| X | Pneumothorax  823 (3) | 87.4  (79.9; 94.8) | 99.0 (98.4; 100.0) | Moderate ⊕⊕⊕⊝  High ⊕⊕⊕⊕ | Moderate | Lung ultrasound (BLUE protocol) | Final diagnosis | Emergency department / Intensive care unit | Asmara[73]  (2022) |
|  | Pneumothorax  3171 (15) | 79.4 (68.2; 90.7) | 99.5 (99.0; 100) | Very low ⊝⊝⊕⊝  Low ⊝⊝⊕⊕ | Low | Lung ultrasound (lung sliding, comet-tails, lung point) | CT | Emergency department | Bouillon-Minois[211]  (2025) |
|  | Pneumothorax  2495 (14) | 88.0  (82.0; 94.0) | 99.0 (98.0; 100.0) | Very low ⊖⊖⊕⊖  Very low ⊖⊖⊕⊖ | Low | Lung ultrasound (lung sliding, comet-tails, lung point) | CT | Emergency physicians only | Ebrahimi[212]  (2014) |
|  | Pneumothorax  3855 (15) | 97.4  (91.3; 99.3) | 93.6 (83.5; 97.7) | Low ⊖⊖⊕⊕ Low ⊖⊕⊕⊖ | Low | eFAST or two pont Lung ultrasound | CT, chest X-ray |  | Ranganath[213] (2026) |
|  | Pneumothorax  1942 (13) | 81.0  (71.0; 88.0) | 98.0 (97.0; 99.0) | Low ⊕⊖⊕⊖  High ⊕⊕⊕⊕ | Moderate | Lung ultrasound (lung sliding, comet-tails) | CT, chest X-ray, chest tube observation |  | Staub[209]  (2018) |
|  | Pleural Effusion  924 (4) | 93.0  (89.0; 96.0) | 96.0  (95.0; 98.0) | Moderate ⊖⊕⊕⊕  Moderate ⊖⊕⊕⊕ | Critically low | Lung ultrasound | CT, thoracic drainage |  | Grimberg[214]  (2010) |
|  | Pleural Effusion  653 (12) | 94.5 (91.7; 97.3) | 97.9 (95.8; 100) | Moderate ⊕⊕⊖⊕  Moderate ⊕⊕⊖⊕ | Moderate | Lung ultrasound (POCUS) | CT, chest X-ray | With pocket device: Sensitivity 96%, Specificity 86% | Zaki[215]  (2024) |
| **Infectious lung diseases** | | |  |  |  |  |  |  |  |
|  | COVID-19  2410 (15) | 88.9  (84.9; 92.0) | 72.2  (58.8; 82.5) | Low ⊕⊖⊖⊕  Very low ⊕⊖⊖⊖ | Moderate | Lung ultrasound | RT-PCR, , serology, WHO- or CDC-criteria |  | Ebrahimzadeh[216] (2022) |
|  | COVID-19  2105 (16) | 86.9  (80.9; 91.3) | 62.4  (49.4; 73.9) | Low ⊕⊖⊕⊖  Low ⊕⊖⊕⊖ | Moderate | Lung ultrasound | RT-PCR | Max 6 regions: 80% Sensitivity, 48% Specificity | Jari[217]  (2022) |
| X | COVID-19  4314 (20) | 87.2  (83.6; 90.2) | 97.9  (95.8; 100) | Moderate ⊕⊖⊕⊕  Low ⊕⊖⊕⊖ | Moderate | Lung ultrasound | RT-PCR, CT, clinical diagnosis |  | Matthies[218]  (2023) |
|  | COVID-19  531 (9) | 96.0  (80.0; 99.0) | 80.0  (60.0; 92.0) | Very low ⊖⊖⊕⊖  Very low ⊖⊖⊕⊖ | Low | Lung ultrasound | CT |  | Wang[219]  (2021) |
|  | Exaz Asthma/COPD  680 (3) | 91.1  (79.0; 99.0) | 88.4 (76.5; 100.0) | Low ⊕⊝⊕⊝  Low ⊕⊝⊕⊝ | Moderate | Lung ultrasound (BLUE protocol) | Final diagnosis | Emergency department / Intensive care unit | Asmara[73]  (2022) |
| X | Exaz Asthma/COPD  527 (4) | 78.0  (67.0; 86.0) | 94.0 (89.0; 97.0) | Low ⊕⊝⊕⊝  Moderate ⊕⊝⊕⊕ | Moderate | Lung ultrasound (BLUE protocol) | Radiological image | Emergency department / Intensive care unit | Staub[69]  (2019) |
|  | Pneumonia  2897 (12) | 92.1 (84.1; 96.3) | 81.9  (72.7; 88.5) | Low ⊝⊕⊕⊝  Very low ⊝⊝⊕⊝ | Low | Lung ultrasound | NA |  | Abid[220]  (2024) |
|  | Pneumonia  2513 (20) | 85.0  (84.0; 87.0) | 93.0  (92.0; 95.0) | Moderate ⊕⊝⊕⊕  Moderate ⊕⊝⊕⊕ | Moderate | Lung ultrasound | CT, chest X-ray, clinical diagnosis | Half of studies on emergency department | Alzahrani[221]  (2017) |
| X | Pneumonia  860 (5) | 84.0  (76.0; 89.0) | 98.0 (93.0; 99.0) | Moderate ⊕⊕⊕⊝  High ⊕⊕⊕⊕ | Moderate | Lung ultrasound (BLUE protocol) | Final diagnosis | Emergency department / Intensive care unit | Asmara[73]  (2022) |
|  | Pneumonia  1172 (10) | 94.0 (92.0; 96.0) | 96.0  (94.0; 97.0) | Moderate ⊝⊕⊕⊕  Low ⊝⊝⊕⊕ | Low | Lung ultrasound | CT, chest X-ray | Half of studies on emergency department | Chavez[222]  (2014) |
|  | Pneumonia  6702 (29) | 92.0 (91.0; 93.0) | 94.0 (94.0; 95.0) | Low ⊝⊝⊕⊕  Low ⊝⊝⊕⊕ | Critically low | Lung ultrasound | CT, chest X-ray, clinical diagnosis |  | Desai[223] (2024) |
|  | Pneumonia  4901 (33) | 91.9 (87.9; 94.7) | 89.7 (81.3; 94.6) | Low ⊝⊝⊕⊕  Very low ⊝⊝⊕⊝ | Low | Lung ultrasound | CT, chest X-ray, clinical diagnosis, expert consensus | Emergency department | Gentilotti[224]  (2022) |
|  | Pneumonia  1080 (9) | 97.0 (93.0; 99.0) | 94.0 (85.0; 98.0) | Moderate ⊝⊕⊕⊕  Low ⊝⊕⊕⊝ | Low | Lung ultrasound | CT, chest X-ray, clinical diagnosis, microbiology |  | Hu[225]  (2014) |
|  | Pneumonia  1515 (12) | 88.0 (86.0; 90.0) | 86.0 (83.0; 88.0) | Low ⊝⊝⊕⊕  Low ⊝⊝⊕⊕ | Low | Lung ultrasound | CT, chest X-ray | Half of studies used X-ray as reference standard | Long[226]  (2017) |
|  | Pneumonia  5108 (17) | 92.0 (86.0; 95.0) | 93.0 (86.0; 97.0) | Moderate ⊕⊝⊕⊕  Low ⊕⊝⊕⊝ | Moderate | Lung ultrasound (Consolidation or focal B-lines) | CT, chest X-ray | Emergency department | Orso[227]  (2018) |
|  | Pneumonia  1390 (8) | 37.0 (19.0; 59.0) | 95.0  (73.0; 99.0) | Low ⊕⊖⊕⊖ Low ⊕⊖⊕⊖ | Moderate | Lung ultrasound (focal B-Lines) | CT, chest X-ray | Emergency department or hospital wards | Padrao[228]  (2025) |
|  | Pneumonia  NA (12) | 86.0 (84.0; 87.0) | 81.0 (79.0; 83.0) | Low ⊝⊝⊕⊕  Low ⊝⊝⊕⊕ | Critically low | Lung ultrasound | CT, chest X-ray, clinical diagnosis, microbiology | Emergency department | Sistani[229]  (2021) |
|  | Pneumonia  933 (6) | 82.0  (74.0; 88.0) | 94.0 (85.0; 98.0) | Low ⊕⊝⊕⊝  Low ⊕⊝⊕⊝ | Moderate | Lung ultrasound (Consolidation) | CT, final diagnosis | Emergency department / Intensive care unit | Staub[69]  (2019) |
|  | Pneumonia  1011 (8) | 90.0 (81.0; 96.0) | 91.0 (80.0; 98.0) | Low ⊕⊝⊕⊝  Low ⊕⊝⊕⊝ | Moderate | Lung ultrasound | CT | Emergency department / Intensive care unit | Vera-Ponce[230] (2025) |
|  | Pneumonia  1911 (14) | 90.4  (88.4; 92.1) | 88.4 (86.1; 90.4) | Low ⊝⊝⊕⊕  Low ⊝⊝⊕⊕ | Low | Lung ultrasound | CT, chest X-ray, clinical diagnosis, microbiology |  | Xia[231]  (2016) |
| **Respiratory other** | | |  |  |  |  |  |  |  |
| X | Acute dyspnea  9275 (19) | 85.6 (84.0; 87.2) | 80.8 (79.0; 82.5) | Low ⊖⊖⊕⊕ Low ⊖⊖⊕⊕ | Low | POCUS | CT, chest X-ray, clinical examination, biomarkers | Emergency department No specific condition | Seyala[232]  (2026) |
| X | Acute respiratory failure  3083 (20) | 89.0 (83.0; 94.0) | 94.0 (90.0; 97.0) | Low ⊕⊖⊕⊖ Moderate ⊕⊖⊕⊕ | Moderate | Lung ultrasound | CT, chest X-ray, composite reference | Mostly intensive care unit | Jianguo[233]  (2026) |
|  | Acute respiratory failure  1232 (11) | 92.0  (85.0; 96.0) | 98.0  (94.0; 99.0) | Very low ⊕⊖⊖⊖  Low ⊕⊖⊖⊕ | Moderate | Lung ultrasound | CT, chest X-ray | Intensive care unit | Yuan[234]  (2021) |
|  | Acute respiratory distress syndrome 2075 (11) | 63.1 (45.0; 78.2) | 94.2 (85.6; 97.8) | Very low ⊕⊝⊝⊝ Low ⊕⊝⊝⊕ | Moderate | Lung ultrasound | Berlin definition using CT or chest X-ray | Mainly intensive care unit | Boumans[235] (2024) |
| X | Acute respiratory distress syndrome 1885 (14) | 84.0 (69.0; 92.0) | 94.0 (83.0; 98.0) | Low ⊕⊖⊕⊖ Low ⊕⊖⊕⊖ | Moderate | Lung ultrasound | Berlin definition using CT or chest X-ray |  | Ji[236] (2026) |
|  | Acute respiratory distress syndrome 1938 (16) | 75.0 (62.0; 85.0) | 87.0 (80.0; 91.0) | Low ⊕⊖⊕⊖ Low ⊕⊖⊕⊖ | Moderate | Lung ultrasound | Berlin definition using CT |  | Sreedharan[237] (2026) |

^1^ “X” indicates the DTA pair selected as the representative for the condition presented in main Tables 2 or 3. For conditions with multiple eligible DTA pairs, selection followed a predefined hierarchy prioritizing: (i) the highest AMSTAR rating, (ii) the highest GRADE certainty, and (iii) the strongest emergency department focus. ^2^ GRADE is detailed as i) Risk of Bias (⊝/⊕), ii) Consistency (⊝/⊕), iii) Directness (⊝/⊕), and iv) Precision (⊝/⊕).

**Abbreviations:** CI = Confidence Interval; CT = Computer Tomography; DTA = diagnostic test accuracy; n = number: NA = Not documented; POCUS = point of care ultrasound; RT-PCR = Real-time polymerase chain reaction; Sens. = Sensitivity; Spec. = Specificity
