# Supplementary material for: Diagnostic accuracy of point-of-care ultrasound in adult non-traumatic emergency medicine: an umbrella review
Source: BMC Med. 2026 Jul 25;24:579. doi: 10.1186/s12916-026-05094-2 (PMC13689006; doi:10.1186/s12916-026-05094-2)
Supplement: Supplementary file 5 [file 12916_2026_5094_MOESM5_ESM.docx]

# Additional file 6: fact sheet for each included study condition

# Cardiac

## Aortic disease

| **Concannon (2014)**[41] – Abdominal aortic aneurysm | | | | | | | |
| --- | --- | --- | --- | --- | --- | --- | --- |
| **Clinical context** | ***Patient population*** | Not documented | | | | | |
|  | ***Clinical setting*** | Emergency department | | | | | |
|  | ***Prevalence [%] (CI)*** | 20.9 (18.4; 23.6) | | | | | |
|  | ***Noteworthy incl. crit.*** | Non radiologist performed ultrasound studies | | | | | |
|  | ***Noteworthy excl. crit.*** | RADUS as index test | | | | | |
| **Index test(s)** | Maximal antero-posterior aortic diameter measurement of > 3 cm on B-mode ultrasound | | | | | | |
| **Reference standard(s)** | Formal radiologist performed aortic imaging, intraoperative findings, findings post mortem | | | | | | |
| **DTA-SR characteristics** | ***No. of studies*** | 11 | | | | | |
|  | ***No. of participants*** | 944 | | | | | |
|  | ***Year range*** | 2000 - 2012 | | | | | |
|  | ***Quality assessment*** | QUADAS; mostly high quality, only 1/11 with score < 12/14 | | | | | |
|  | ***Special remark*** | 3 studies included patients with asymptomatic AAA | | | | | |
| **Diagnostic accuracy** | ***Measure*** | Sensitivity [%] (95% CI):  97.5 (91.2; 99.2) | | | Specificity [%] (95% CI):  98.9 (97.9; 99.5) | | |
|  | ***GRADE*** | High ⊕⊕⊕⊕ | | | High ⊕⊕⊕⊕ | | |
| **Method. quality**  **(mod AMSTAR2)** | **Overall** | Moderate | | | | | |
| **Further investigations, sub- or sensitivity analyses** | **Index test** | | **Reference standard** | **Studies (Particip.)** | | **Sens. [%], 95% CI** | **Spec. [%], 95% CI** |
|  |  | |  |  | |  |  |
|  |  | |  |  | |  |  |
|  |  | |  |  | |  |  |
|  |  | |  |  | |  |  |
| **Conclusions of study authors** | *“Non-radiologist performed ultrasound has been shown to achieve acceptable levels of sensitivity and specificity for the diagnosis of AAA according to the results of this meta-analysis. The risks associated with missed pathology in diagnosis of AAA in symptomatic and asymptomatic patients are substantial. Our results support the use of non-radiology-performed ultrasound as a diagnostic adjunct rather than a replacement for radiology-performed advanced imaging. Further research by way of a randomized controlled trial is needed to determine the potential direct effect of non-radiology-performed ultrasound on time to diagnosis, time to disposition for surgical intervention and on the mortality of patients presenting with ruptured and unruptured AAA.”* | | | | | | |

| **Rubano (2013)**[42] – Abdominal aortic aneurysm | | | | | | | |
| --- | --- | --- | --- | --- | --- | --- | --- |
| **Clinical context** | ***Patient population*** | Adults | | | | | |
|  | ***Clinical setting*** | Emergency department | | | | | |
|  | ***Prevalence [%] (CI)*** | 23.5 (20.2; 26.8) | | | | | |
|  | ***Noteworthy incl. crit.*** | Bedside ultrasound performed by emergency physicians, adult patients with symptoms/signs suggestive of AAAs | | | | | |
|  | ***Noteworthy excl. crit.*** | - | | | | | |
| **Index test(s)** | Aortic diameter of ≥3 cm on emergency department ultrasound | | | | | | |
| **Reference standard(s)** | CT, angiography, MRI, surgery, RADUS | | | | | | |
| **DTA-SR characteristics** | ***No. of studies*** | 7 | | | | | |
|  | ***No. of participants*** | 655 | | | | | |
|  | ***Year range*** | 2000 - 2005 | | | | | |
|  | ***Quality assessment*** | QUADAS (no high quality studies) | | | | | |
|  | ***Special remark*** | Indeterminate scans were coded as false positives | | | | | |
| **Diagnostic accuracy** | ***Measure*** | Sensitivity [%] (95% CI):  99.0 (95.0; 100.0) | | | Specificity [%] (95% CI):  99.0 (97.0; 99.0) | | |
|  | ***GRADE*** | Moderate ⊖⊕⊕⊕ | | | Moderate ⊖⊕⊕⊕ | | |
| **Method. quality**  **(mod AMSTAR2)** | **Overall** | Low | | | | | |
| **Further investigations, sub- or sensitivity analyses** | **Index test** | | **Reference standard** | **Studies (Particip.)** | | **Sens. [%], 95% CI** | **Spec. [%], 95% CI** |
|  |  | |  |  | |  |  |
|  |  | |  |  | |  |  |
|  |  | |  |  | |  |  |
|  |  | |  |  | |  |  |
| **Conclusions of study authors** | *“Emergency physician bedside ultrasound can be used to rule in (positive likelihood ratio 10.8 to infinity) or rule out (negative likelihood ratio 0 to 0.025) the need for emergent CT and/or vascular surgery consultation. Emergency bedside ultrasound can be used with great accuracy to detect the presence of abdominal aortic aneurysm in symptomatic patients.”* | | | | | | |

| **Shaban (2025)**[43] – Abdominal aortic aneurysm | | | | | | | |
| --- | --- | --- | --- | --- | --- | --- | --- |
| **Clinical context** | ***Patient population*** | Adults | | | | | |
|  | ***Clinical setting*** | Emergency department | | | | | |
|  | ***Prevalence [%] (CI)*** | Not documented | | | | | |
|  | ***Noteworthy incl. crit.*** | Bedside US/POCUS in the ED by EP | | | | | |
|  | ***Noteworthy excl. crit.*** | Case reports, case series, fewer than 10 participants | | | | | |
| **Index test(s)** | POCUS | | | | | | |
| **Reference standard(s)** | CT, RADUS, MRI, angiography, laparotomy, autopsy | | | | | | |
| **DTA-SR characteristics** | ***No. of studies*** | 9 | | | | | |
|  | ***No. of participants*** | Not documented | | | | | |
|  | ***Year range*** | 2000 - 2023 | | | | | |
|  | ***Quality assessment*** | QUADAS (8 studies with high risk of bias) | | | | | |
|  | ***Special remark*** | One included study doesn’t report No of participants | | | | | |
| **Diagnostic accuracy** | ***Measure*** | Sensitivity [%] (95% CI):  98.0 (96.0; 100) | | | Specificity [%] (95% CI):  100 (99.0; 100) | | |
|  | ***GRADE*** | Moderate ⊖⊕⊕⊕ | | | Moderate ⊖⊕⊕⊕ | | |
| **Method. quality**  **(mod AMSTAR2)** | **Overall** | Low | | | | | |
| **Further investigations, sub- or sensitivity analyses** | **Index test** | | **Reference standard** | **Studies (Particip.)** | | **Sens. [%], 95% CI** | **Spec. [%], 95% CI** |
|  |  | |  |  | |  |  |
|  |  | |  |  | |  |  |
|  |  | |  |  | |  |  |
|  |  | |  |  | |  |  |
| **Conclusions of study authors** | *“[…]this meta-analysis has shown that emergency physicians can accurately undertake POCUS to detect AAA even with limited training. Moreover, emergency physicians can utilize POCUS to identify patients with ruptured AAA, which allows appropriate management to be undertaken. Unfortunately, POCUS examinations performed by EPs are still subject to misdiagnosis and are limited in patients with bowel gas and obesity. Therefore, until these issues are resolved, POCUS cannot replace CT as the gold standard but can be considered an adjunct imaging modality.”* | | | | | | |

| **Fernando (2022)**[44] – Ruptured abdominal aortic aneurysm | | | | | | | |
| --- | --- | --- | --- | --- | --- | --- | --- |
| **Clinical context** | ***Patient population*** | Adults ≥ 16y | | | | | |
|  | ***Clinical setting*** | Emergency department | | | | | |
|  | ***Prevalence [%] (CI)*** | Not documented | | | | | |
|  | ***Noteworthy incl. crit.*** | Adult patients, acute symptoms or confirmed rAAA, POCUS by any provider | | | | | |
|  | ***Noteworthy excl. crit.*** | - | | | | | |
| **Index test(s)** | B-mode US (POCUS) | | | | | | |
| **Reference standard(s)** | CTA, surgical findings | | | | | | |
| **DTA-SR characteristics** | ***No. of studies*** | 5 | | | | | |
|  | ***No. of participants*** | 628 | | | | | |
|  | ***Year range*** | 2000 - 2019 | | | | | |
|  | ***Quality assessment*** | QUADAS-2 (all studies with high risk of bias for reference standard) | | | | | |
|  | ***Special remark*** | Not for rAAA but for AAA in the setting of suspected rAAA, absence of AAA on POCUS meant that rAAA is highly unlikely | | | | | |
| **Diagnostic accuracy** | ***Measure*** | Sensitivity [%] (95% CI):  97.8 (95.4; 100) | | | Specificity [%] (95% CI):  97.0 (93.9; 100) | | |
|  | ***GRADE*** | Moderate ⊕⊖⊕⊕ | | | Moderate ⊕⊖⊕⊕ | | |
| **Method. quality**  **(mod AMSTAR2)** | **Overall** | Moderate | | | | | |
| **Further investigations, sub- or sensitivity analyses** | **Index test** | | **Reference standard** | **Studies (Particip.)** | | **Sens. [%], 95% CI** | **Spec. [%], 95% CI** |
|  |  | |  |  | |  |  |
|  |  | |  |  | |  |  |
|  |  | |  |  | |  |  |
|  |  | |  |  | |  |  |
| **Conclusions of study authors** | *„Finally, point-of-care ultrasound had very high sensitivity and specificity for diagnosis of abdominal aortic aneurysm in patients presenting with acute symptoms, but cannot definitively evaluate for rupture, and therefore a high index of suspicion should still prompt further imaging or surgical evaluation”* | | | | | | |

| **Sutarjono (2023)**[45] – Aortic dissection | | | | | | | |
| --- | --- | --- | --- | --- | --- | --- | --- |
| **Clinical context** | ***Patient population*** | Not documented | | | | | |
|  | ***Clinical setting*** | Not documented | | | | | |
|  | ***Prevalence [%] (CI)*** | Not documented | | | | | |
|  | ***Noteworthy incl. crit.*** | Clearly defined reference standard | | | | | |
|  | ***Noteworthy excl. crit.*** | Case reports | | | | | |
| **Index test(s)** | TTE overall | | | | | | |
| **Reference standard(s)** | CT/CTA, TEE, MRI, surgery/autopsy | | | | | | |
| **DTA-SR characteristics** | ***No. of studies*** | 14 | | | | | |
|  | ***No. of participants*** | 9602 (examinations) | | | | | |
|  | ***Year range*** | 1989-2021 | | | | | |
|  | ***Quality assessment*** | QUADAS-2 (relatively low risk of bias in all studies) | | | | | |
|  | ***Special remark*** | heterogeneity is described as low, but visual inspection of forest plots suggests high heterogeneity | | | | | |
| **Diagnostic accuracy** | ***Measure*** | Sensitivity [%] (95% CI):  62.4 (60.8; 64.0) | | | Specificity [%] (95% CI):  87.5 (86.7; 88.3) | | |
|  | ***GRADE*** | Low ⊕⊖⊕⊖ | | | Moderate ⊕⊖⊕⊕ | | |
| **Method. quality**  **(mod AMSTAR2)** | **Overall** | Moderate | | | | | |
| **Further investigations, sub- or sensitivity analyses** | **Index test** | | **Reference standard** | **Studies (Particip.)** | | **Sens. [%], 95% CI** | **Spec. [%], 95% CI** |
|  | TTE any sonographic feature | | CT/CTA, TEE, MRI, surgery/autopsy | 12 (NA) | | 91 (88; 93) | NA |
|  | TTE intimal flap, tear, or intramural hematoma | | CT/CTA, TEE, MRI, surgery/autopsy | 9 (NA) | | 69 (65; 73) | 98 (96;98) |
|  | TTE pericardial effusion | | CT/CTA, TEE, MRI, surgery/autopsy | 8 (NA) | | NA | 95 (94; 96) |
|  |  | |  |  | |  |  |
| **Conclusions of study authors** | *“…we show that there is diagnostic value in the utilization of TTE for the diagnosis of proximal aortic dissection. In summary, the identification of an intimal flap, tear, or intramural hematoma effectively rules in the disease with a high level of confidence, while the absence of any sonographic feature following a systematic TTE scan greatly lowers suspicion. We suggest that employing this TTE search strategy can provide pivotal information early on during the patient’s presentation and should be considered as a routine tool for triage and assessment for proximal aortic dissection.”* | | | | | | |

| **Yeh (2025)**[47] – Acute aortic dissection Type A | | | | | | | |
| --- | --- | --- | --- | --- | --- | --- | --- |
| **Clinical context** | ***Patient population*** | Not documented | | | | | |
|  | ***Clinical setting*** | Emergency department, intensive care unit, wards | | | | | |
|  | ***Prevalence [%] (CI)*** | 21.4 (NA) | | | | | |
|  | ***Noteworthy incl. crit.*** | - | | | | | |
|  | ***Noteworthy excl. crit.*** | - | | | | | |
| **Index test(s)** | TTE, direct sign of dissection | | | | | | |
| **Reference standard(s)** | CT, MRA, TEE, aortography, autopsy | | | | | | |
| **DTA-SR characteristics** | ***No. of studies*** | 8 | | | | | |
|  | ***No. of participants*** | 2560 | | | | | |
|  | ***Year range*** | 1985 - 2023 | | | | | |
|  | ***Quality assessment*** | QUADAS-2 (no low risk of bias studies) | | | | | |
|  | ***Special remark*** | Rated certainty of evidence according to GRADE, sensitivity low, specificity moderate | | | | | |
| **Diagnostic accuracy** | ***Measure*** | Sensitivity [%] (95% CI):  58.0 (38.0; 76.0) | | | Specificity [%] (95% CI):  94.0 (89.0; 97.0) | | |
|  | ***GRADE*** | Low ⊕⊖⊕⊖ | | | Moderate ⊕⊖⊕⊕ | | |
| **Method. quality**  **(mod AMSTAR2)** | **Overall** | Moderate | | | | | |
| **Further investigations, sub- or sensitivity analyses** | **Index test** | | **Reference standard** | **Studies (Particip.)** | | **Sens. [%], 95% CI** | **Spec. [%], 95% CI** |
|  | TTE, any sign | | CT, MRA, TEE, aortography, autopsy | 5 (2011) | | 91 (85; 94) | 74 (61; 84) |
|  | TTE, direct sign or root dilation | | CT, MRA, TEE, aortography, autopsy | 2 (235) | | 89 (37; 99) | 78 (59; 90) |
|  | TTE, root dilation | | CT, MRA, TEE, aortography, autopsy | 5 (2250) | | 74 (65; 82) | 78 (72; 83) |
|  | TTE, pericardial effusion | | CT, MRA, TEE, aortography, autopsy | 4 (1741) | | 35 (26; 45) | 92 (89; 95) |
|  | TTE, aortic regurgitation | | CT, MRA, TEE, aortography, autopsy | 4 (1741) | | 35 (16; 59) | 88 (74; 95) |
| **Conclusions of study authors** | *“Our study proposes that TTE could be considered as the initial imaging method in the diagnostic process for patients with suspected AAAS. Given its high specificity, the presence of direct TTE signs acts as a rule-in test, providing a more straightforward path to establishing a definitive diagnosis. Moreover, combining the absence of any TTE sign with low clinical suspicion could offer a more secure basis for excluding AAAS.”* | | | | | | |

| **Zaki (2023)**[46] – Acute aortic dissection Type A | | | | | | | |
| --- | --- | --- | --- | --- | --- | --- | --- |
| **Clinical context** | ***Patient population*** | Adults | | | | | |
|  | ***Clinical setting*** | Not documented | | | | | |
|  | ***Prevalence [%] (CI)*** | 32 % | | | | | |
|  | ***Noteworthy incl. crit.*** | Observational or randomized studies, any AAD classification, evaluated TTE or TEE | | | | | |
|  | ***Noteworthy excl. crit.*** | Studies that only included patients with chronic or asymptomatic aortic disease or did not differentiate between AD and AAD, studies with sample size < 30 | | | | | |
| **Index test(s)** | TTE for type A AAD (conventional and contrast-enhanced) | | | | | | |
| **Reference standard(s)** | Chest x-ray, CT/CTA, coronary angiography, MRI, surgical findings, autopsy | | | | | | |
| **DTA-SR characteristics** | ***No. of studies*** | 8 | | | | | |
|  | ***No. of participants*** | 2240 | | | | | |
|  | ***Year range*** | 1992 - 2022 | | | | | |
|  | ***Quality assessment*** | QUADAS-2 (all low risk studies) | | | | | |
|  | ***Special remark*** | The pooled data from studies evaluating the role of TTE in diagnosing AAD shows that false negatives occur at a rate of 28.6%, while false positives occur at a rate of 18.6%. More studies evaluated sensitivity than specificity (8 vs 6) | | | | | |
| **Diagnostic accuracy** | ***Measure*** | Sensitivity [%] (95% CI):  86.7 (80.5; 92.8) | | | Specificity [%] (95% CI):  86.5 (77.4; 95.6) | | |
|  | ***GRADE*** | Low ⊕⊖⊕⊖ | | | Low ⊕⊖⊕⊖ | | |
| **Method. Quality**  **(mod AMSTAR2)** | **Overall** | Moderate | | | | | |
| **Further investigations, sub- or sensitivity analyses** | **Index test** | | **Reference standard** | **Studies (Particip.)** | | **Sens. [%], 95% CI** | **Spec. [%], 95% CI** |
|  | TTE for type A AAD conventional | | Chest x-ray, CT/CTA, coronary angiography, MRI, surgical findings, autopsy | 8 (NA) | | 85 (78; 93) | 85 (74; 96) |
|  |  | |  |  | |  |  |
|  |  | |  |  | |  |  |
|  |  | |  |  | |  |  |
| **Conclusions of study authors** | *“…in emergency settings where TEE is not readily available, this imaging modality [TTE] can be used for the diagnosis.”* | | | | | | |

| **Zaki (2023)**[46] – Acute aortic dissection Type B | | | | | | | |
| --- | --- | --- | --- | --- | --- | --- | --- |
| **Clinical context** | ***Patient population*** | Adults | | | | | |
|  | ***Clinical setting*** | Not documented | | | | | |
|  | ***Prevalence [%] (CI)*** | Not documented | | | | | |
|  | ***Noteworthy incl. crit.*** | Observational or randomized studies, any AAD classification, evaluated TTE or TEE | | | | | |
|  | ***Noteworthy excl. crit.*** | Studies that only included patients with chronic or asymptomatic aortic disease or did not differentiate between AD and AAD, studies with sample size < 30 | | | | | |
| **Index test(s)** | TTE for type B AAD (conventional and contrast-enhanced) | | | | | | |
| **Reference standard(s)** | Chest x-ray, CT/CTA, coronary angiography, MRI, surgical findings, autopsy | | | | | | |
| **DTA-SR characteristics** | ***No. of studies*** | 4 | | | | | |
|  | ***No. of participants*** | Not documented | | | | | |
|  | ***Year range*** | 1992 - 2022 | | | | | |
|  | ***Quality assessment*** | QUADAS-2 (all low risk studies) | | | | | |
|  | ***Special remark*** | More studies in sensitivity than specificity | | | | | |
| **Diagnostic accuracy** | ***Measure*** | Sensitivity [%] (95% CI):  56.8 (32.5; 81.1)) | | | Specificity [%] (95% CI):  92.4 (81.6; 103.3) | | |
|  | ***GRADE*** | Low ⊕⊖⊕⊖ | | | Low ⊕⊖⊕⊖ | | |
| **Method. Quality**  **(mod AMSTAR2)** | **Overall** | Moderate | | | | | |
| **Further investigations, sub- or sensitivity analyses** | **Index test** | | **Reference standard** | **Studies (Particip.)** | | **Sens. [%], 95% CI** | **Spec. [%], 95% CI** |
|  | TTE for type B AAD  conventional | | Chest x-ray, CT/CTA, coronary angiography, MRI, surgical findings, autopsy | 3 (NA) | | 46 (21, 71) | 87 (66; 107) |
|  |  | |  |  | |  |  |
|  |  | |  |  | |  |  |
|  |  | |  |  | |  |  |
| **Conclusions of study authors** | “…in emergency settings where TEE is not readily available, this imaging modality [TTE] can be used for the diagnosis.” | | | | | | |

| **Mani (2025)**[48] – Acute thoracic aortic syndrome | | | | | | | |
| --- | --- | --- | --- | --- | --- | --- | --- |
| **Clinical context** | ***Patient population*** | Adults | | | | | |
|  | ***Clinical setting*** | Emergency department (acute health care setting) | | | | | |
|  | ***Prevalence [%] (CI)*** | 11.0 (1.4; 45.7) | | | | | |
|  | ***Noteworthy incl. crit.*** | Cross-sectional studies, any echocardiography technology, any user, any definition of a positive test | | | | | |
|  | ***Noteworthy excl. crit.*** | Case reports / series, systematic reviews, MA’s | | | | | |
| **Index test(s)** | Bedside focused TTE, direct sign of dissection, Type A dissection | | | | | | |
| **Reference standard(s)** | CTA, TEE, MRI angiography, surgery, autopsy, follow-up | | | | | | |
| **DTA-SR characteristics** | ***No. of studies*** | 7 | | | | | |
|  | ***No. of participants*** | NA | | | | | |
|  | ***Year range*** | 1981 - 2023 | | | | | |
|  | ***Quality assessment*** | QUADAS-2 (only low risk of bias studies included) | | | | | |
|  | ***Special remark*** |  | | | | | |
| **Diagnostic accuracy** | ***Measure*** | Sensitivity [%] (95% CI):  89.0 (82.0; 84.0) | | | Specificity [%] (95% CI):  92.0 (88.0; 95.0) | | |
|  | ***GRADE*** | Moderate ⊕⊕⊖⊕ | | | High ⊕⊕⊕⊕ | | |
| **Method. quality**  **(mod AMSTAR2)** | **Overall** | Moderate | | | | | |
| **Further investigations, sub- or sensitivity analyses** | **Index test** | | **Reference standard** | **Studies (Particip.)** | | **Sens. [%], 95% CI** | **Spec. [%], 95% CI** |
|  | TTE, direct sign of dissection, Type B dissection | | CTA, TEE, MRI angiography, surgery, autopsy, follow-up | 5 (NA) | | 65 (45; 80) | 100 (0; 100) |
|  | TTE, indirect sign of Dissection, aortic valve regurgitation | |  | 3 (NA) | | 64 (4; 99) | 94 (92; 96) |
|  | TTE, indirect sign of Dissection, thoracic aortic aneurysm | |  | 4 (NA) | | 92 (52; 99) | 87 (62; 97) |
|  | TTE, indirect sign of Dissection, pericardial effusion | |  | 4 (NA) | | 39 (34; 45) | 94 (92; 95) |
| **Conclusions of study authors** | *“In this systematic review and meta-analysis, bedside- focused TTE has high specificity for type A and B dis section, a moderate to high sensitivity for type A but poor for type B, but unclear diagnostic accuracy for intramural hematoma and penetrating aortic ulcer. Bedside-focused TTE may also improve time to diagnosis and facilitating early specialty consultation, transfer to a tertiary hospital and definitive treatment. However, routine and unselected deployment of bedside-focused TTE in the hands of novice operators, particularly in ruling out thoracic aortic syndrome, is not clinically recommended.”* | | | | | | |

###### CPR

| **Albaroudi (2024)**[49] – Cardiac arrest outcome / death | | | | | | | |
| --- | --- | --- | --- | --- | --- | --- | --- |
| **Clinical context** | ***Patient population*** | Adults | | | | | |
|  | ***Clinical setting*** | Mostly emergency department (out of hospital cardiac arrest) | | | | | |
|  | ***Prevalence [%] (CI)*** | Not documented | | | | | |
|  | ***Noteworthy incl. crit.*** | Prehospital or emergency department | | | | | |
|  | ***Noteworthy excl. crit.*** | In hospital cardiac arrest, transoesophageal echocardiography | | | | | |
| **Index test(s)** | POCUS, absence of cardiac activity | | | | | | |
| **Reference standard(s)** | Termination of resuscitation / no return of circulation | | | | | | |
| **DTA-SR characteristics** | ***No. of studies*** | 15 | | | | | |
|  | ***No. of participants*** | 2239 | | | | | |
|  | ***Year range*** | 2001 - 2021 | | | | | |
|  | ***Quality assessment*** | QUADAS-2, mostly at risk in patient selection | | | | | |
|  | ***Special remark*** | Analyses only for medical cardiac arrest | | | | | |
| **Diagnostic accuracy** | ***Measure*** | Sensitivity [%] (95% CI):  87.0 (75.0; 94.0) | | | Specificity [%] (95% CI):  70.0 (56.0; 82.0) | | |
|  | ***GRADE*** | Low ⊕⊖⊕⊖ | | | Low ⊕⊖⊕⊖ | | |
| **Method. quality (mod AMSTAR2)** | **Overall** | Moderate | | | | | |
| **Further investigations, sub- or sensitivity analyses** | **Index test** | | **Reference standard** | **Studies (Particip.)** | | **Sens. [%], 95% CI** | **Spec. [%], 95% CI** |
|  | POCUS | | Death before hospital admission | 15 (2239) | | 82 (72, 88) | 82 (64, 92) |
|  | POCUS | | Death before hospital discharge / 30 days | 15 (2239) | | 72 (62, 80) | 80 (58, 92) |
|  |  | |  |  | |  |  |
|  |  | |  |  | |  |  |
| **Conclusions of study authors** | *“The absence of cardiac activity on intra-arrest point of care echocardiography for medical cardiac arrest predicts a poor prognosis but is not a stand-alone tool to predict death and thus guide the cessation or continuation of a resuscitation.”* | | | | | | |

| **Blyth (2012)**[50] – Cardiac arrest outcome / survival | | | | | | | |
| --- | --- | --- | --- | --- | --- | --- | --- |
| **Clinical context** | ***Patient population*** | Adults | | | | | |
|  | ***Clinical setting*** | Not documented | | | | | |
|  | ***Prevalence [%] (CI)*** | Not documented | | | | | |
|  | ***Noteworthy incl. crit.*** | Clinician performed transthoracic echo, adults in cardiac arrest during CPR, outcome reported | | | | | |
|  | ***Noteworthy excl. crit.*** | TEE | | | | | |
| **Index test(s)** | POCUS, presence of cardiac activity | | | | | | |
| **Reference standard(s)** | Survival / ROSC | | | | | | |
| **DTA-SR characteristics** | ***No. of studies*** | 8 | | | | | |
|  | ***No. of participants*** | 568 | | | | | |
|  | ***Year range*** | 2001 - 2012 | | | | | |
|  | ***Quality assessment*** | Modified QUADAS, overall low risk of bias | | | | | |
|  | ***Special remark*** | Mixed population of traumatic and medical cardiac arrest patients; not all studies had ROSC as primary endpoint | | | | | |
| **Diagnostic accuracy** | ***Measure*** | Sensitivity [%] (95% CI):  91.6 (84.6; 96.1) | | | Specificity [%] (95% CI):  80.0 (76.1; 83.6) | | |
|  | ***GRADE*** | Low ⊕⊖⊕⊖ | | | Moderate ⊕⊖⊕⊕ | | |
| **Method. quality**  **(mod AMSTAR2)** | **Overall** | Moderate | | | | | |
| **Further investigations, sub- or sensitivity analyses** | **Index test** | | **Reference standard** | **Studies (Particip.)** | | **Sens. [%], 95% CI** | **Spec. [%], 95% CI** |
|  |  | |  |  | |  |  |
|  |  | |  |  | |  |  |
|  |  | |  |  | |  |  |
|  |  | |  |  | |  |  |
| **Conclusions of study authors** | *“In selected patients with a higher likelihood of survival from cardiac arrest at presentation, based on established predictors of survival, point-of-care ultrasound should not be the sole basis for the decision to cease resuscitative efforts. Presence of cardiac activity on ultrasound is a weak predictor of return of spontaneous circulation.”* | | | | | | |

| **Breglia (2025)**[51] – Cardiac arrest outcome / death | | | | | | | |
| --- | --- | --- | --- | --- | --- | --- | --- |
| **Clinical context** | ***Patient population*** | Adults | | | | | |
|  | ***Clinical setting*** | Intra-hospital, pre-hospital | | | | | |
|  | ***Prevalence [%] (CI)*** | Not documented | | | | | |
|  | ***Noteworthy incl. crit.*** | Traumatic and non-traumatic cases | | | | | |
|  | ***Noteworthy excl. crit.*** | Studies that used transesophageal echocardiography, POCUS performed post-ROSC, or that included pediatric populations. | | | | | |
| **Index test(s)** | POCUS, presence of cardiac activity | | | | | | |
| **Reference standard(s)** | Clinical outcome (ROSC, death) | | | | | | |
| **DTA-SR characteristics** | ***No. of studies*** | 24 | | | | | |
|  | ***No. of participants*** | 3684 | | | | | |
|  | ***Year range*** | 2001 - 2024 | | | | | |
|  | ***Quality assessment*** | QUADAS-2 | | | | | |
|  | ***Special remark*** | No significant difference in subgroups | | | | | |
| **Diagnostic accuracy** | ***Measure*** | Sensitivity [%] (95% CI):  86.5 (78.9; 90.4) | | | Specificity [%] (95% CI):  79.0 (67.1; 87.4) | | |
|  | ***GRADE*** | Low ⊕⊖⊕⊖ | | | Low ⊕⊖⊕⊖ | | |
| **Method. quality (mod AMSTAR2)** | ***Overall*** | Moderate | | | | | |
| **Further investigations, sub- or sensitivity analyses** | **Index test** | | **Reference standard** | **Studies (Particip.)** | | **Sens. [%], 95% CI** | **Spec. [%], 95% CI** |
|  | POCUS by long-term trained, certified examiner | | Survival (ROSC) | 11 (1486) | | 83 (69; 92) | 81 (65; 91) |
|  | No information regarding operator training | | Survival (ROSC) | 6 (1443) | | 90 (82; 95) | 70 (49; 85) |
|  | POCUS by hands-on or short-term session trained examiner | | Survival (ROSC) | 7 (755) | | 84 (72; 92) | 82 (51; 95) |
|  |  | |  |  | |  |  |
| **Conclusions of study authors** | *“This systematic review and meta-analysis demonstrate that cardiac standstill on early point-of-care focused TTE during cardiac arrest is significantly associated with an absence of ROSC, exhibiting good diagnostic accuracy.”* | | | | | | |

| **Dudek (2023)**[52] – Cardiac arrest outcome / survival | | | | | | | |
| --- | --- | --- | --- | --- | --- | --- | --- |
| **Clinical context** | ***Patient population*** | Adults | | | | | |
|  | ***Clinical setting*** | Emergency department (out of hospital cardiac arrest) | | | | | |
|  | ***Prevalence [%] (CI)*** | Not documented | | | | | |
|  | ***Noteworthy incl. crit.*** | Point of care echo during cardiopulmonary resuscitation | | | | | |
|  | ***Noteworthy excl. crit.*** | Children, insufficient data for 2x2 table generation | | | | | |
| **Index test(s)** | POCUS, presence of cardiac activity | | | | | | |
| **Reference standard(s)** | ROSC | | | | | | |
| **DTA-SR characteristics** | ***No. of studies*** | 9 | | | | | |
|  | ***No. of participants*** | Not documented | | | | | |
|  | ***Year range*** | 2001 - 2019 | | | | | |
|  | ***Quality assessment*** | ROBINS-I and RoB tool, overall moderate risk of bias | | | | | |
|  | ***Special remark*** | Discussion seems faulty, talks of lung fields when the index test was cardiac POCUS | | | | | |
| **Diagnostic accuracy** | ***Measure*** | Sensitivity [%] (95% CI):  19.4 (13.9; 26.0) | | | Specificity [%] (95% CI):  73.5 (58.9; 85.1) | | |
|  | ***GRADE*** | Very low ⊖⊖⊕⊖ | | | Very low ⊖⊖⊕⊖ | | |
| **Method. quality**  **(mod AMSTAR2)** | **Overall** | Critically low | | | | | |
| **Further investigations, sub- or sensitivity analyses** | **Index test** | | **Reference standard** | **Studies (Particip.)** | | **Sens. [%], 95% CI** | **Spec. [%], 95% CI** |
|  | POCUS | | Survivals to admission | 14 (NA) | | 12 (9; 15) | 26 (17; 37) |
|  | POCUS | | Survival to hospital discharge | 6 (NA) | | 4 (2; 8) | 2 (0; 13) |
|  |  | |  |  | |  |  |
|  |  | |  |  | |  |  |
| **Conclusions of study authors** | *“The results do not allow unambiguous recommendation of POCUS as a predictor of resuscitation outcomes and further studies based on a large number of patients with full standardization of operators, their training and procedures performed are necessary.”* | | | | | | |

| **Lalande (2019)**[53] – Cardiac arrest outcome (atraumatic) / survival | | | | | | | |
| --- | --- | --- | --- | --- | --- | --- | --- |
| **Clinical context** | ***Patient population*** | Adults | | | | | |
|  | ***Clinical setting*** | Out-of-/pre-hospital | | | | | |
|  | ***Prevalence [%] (CI)*** | 16.6 (14.6; 18.8) | | | | | |
|  | ***Noteworthy incl. crit.*** | TTE on non-traumatic cardiac arrest patients during CPR, out of hospital cardiac arrest and emergency department cardiac arrest | | | | | |
|  | ***Noteworthy excl. crit.*** | TEE, traumatic cardiac arrest | | | | | |
| **Index test(s)** | POCUS, presence of cardiac activity | | | | | | |
| **Reference standard(s)** | Survival / ROSC | | | | | | |
| **DTA-SR characteristics** | ***No. of studies*** | 6 | | | | | |
|  | ***No. of participants*** | 1174 | | | | | |
|  | ***Year range*** | 2005 – 2017 | | | | | |
|  | ***Quality assessment*** | QUADAS-2, overall low risk or bias | | | | | |
|  | ***Special remark*** | Better sensitivity for ROSC in PEA than asystole | | | | | |
| **Diagnostic accuracy** | ***Measure*** | Sensitivity [%] (95% CI):  60.3 (38.1; 78.9) | | | Specificity [%] (95% CI):  91.5 (80.8; 96.5) | | |
|  | ***GRADE*** | Low ⊕⊖⊕⊖ | | | Low ⊕⊖⊕⊖ | | |
| **Method. quality**  **(mod AMSTAR2)** | **Overall** | Moderate | | | | | |
| **Further investigations, sub- or sensitivity analyses** | **Index test** | | **Reference standard** | **Studies (Particip.)** | | **Sens. [%], 95% CI** | **Spec. [%], 95% CI** |
|  | POCUS | | Death before hospital admission | 6 (1437) | | 75 (61; 85) | 81 (72; 87) |
|  | POCUS | | Death before hospital discharge | 9 (1127) | | 75 (53; 89) | 75 (60; 85) |
|  |  | |  |  | |  |  |
|  |  | |  |  | |  |  |
| **Conclusions of study authors** | *“Although overall outcomes for survival were poor, the presence of cardiac activity on POCUS was associated with improved odds and increased likelihood of ROSC, sustained hospital admission, and sustained hospital discharge among adults with non-traumatic asystole or PEA. The absence of cardiac activity on POCUS was less likely to predict unsuccessful resuscitations. While POCUS may provide valuable information in the management of non-traumatic PEA or asystole, it should not be viewed as the sole predictor in determining the possibility of a successful or unsuccessful resuscitation effort in these patients.”* | | | | | | |

| **Lalande (2021)**[54] – Cardiac arrest outcome (traumatic) / death | | | | | | | |
| --- | --- | --- | --- | --- | --- | --- | --- |
| **Clinical context** | ***Patient population*** | Mixed (1 patient < 18y) | | | | | |
|  | ***Clinical setting*** | Preclinical, Emergency department (out of hospital cardiac arrest) | | | | | |
|  | ***Prevalence [%] (CI)*** | Not documented | | | | | |
|  | ***Noteworthy incl. crit.*** | POCUS on patients with traumatic cardiac arrest | | | | | |
|  | ***Noteworthy excl. crit.*** | Low energy trauma, in-hospital cardiac arrest, TEE, POCUS post-ROSC | | | | | |
| **Index test(s)** | POCUS (mostly subxiphoidal window), absence of cardiac activity | | | | | | |
| **Reference standard(s)** | ROSC | | | | | | |
| **DTA-SR characteristics** | ***No. of studies*** | 5 | | | | | |
|  | ***No. of participants*** | 82 | | | | | |
|  | ***Year range*** | 2009 - 2019 | | | | | |
|  | ***Quality assessment*** | QUADAS-2, overall low risk of bias | | | | | |
|  | ***Special remark*** | Meta analysis only performed on very small subpopulation | | | | | |
| **Diagnostic accuracy** | ***Measure*** | Sensitivity [%] (95% CI):  91.0 (67.0; 98.0) | | | Specificity [%] (95% CI):  98.0 (13.0; 100) | | |
|  | ***GRADE*** | Low ⊕⊖⊕⊖ | | | Low ⊕⊖⊕⊖ | | |
| **Method. quality**  **(mod AMSTAR2)** | **Overall** | Moderate | | | | | |
| **Further investigations, sub- or sensitivity analyses** | **Index test** | | **Reference standard** | **Studies (Particip.)** | | **Sens. [%], 95% CI** | **Spec. [%], 95% CI** |
|  |  | |  |  | |  |  |
|  |  | |  |  | |  |  |
|  |  | |  |  | |  |  |
|  |  | |  |  | |  |  |
| **Conclusions of study authors** | *“The finding of cardiac activity is associated with an increased probability of ROSC, and POCUS may direct the resuscitation team to reversible causes such as tension pneumothorax or cardiac tamponade. The numbers of patients included in published studies remains too low for practice recommendations...”* | | | | | | |

| **Tsou (2017)**[55] – Cardiac arrest (Survival to hospital admission) | | | | | | | |
| --- | --- | --- | --- | --- | --- | --- | --- |
| **Clinical context** | ***Patient population*** | Adults with cardiac arrest | | | | | |
|  | ***Clinical setting*** | Preclinical (3), Emergency department (7) | | | | | |
|  | ***Prevalence [%] (CI)*** | Not documented | | | | | |
|  | ***Noteworthy incl. crit.*** | Studies that evaluated transthoracic point-of-care echocardiography | | | | | |
|  | ***Noteworthy excl. crit.*** | Case reports, comments, reviews, guidelines and animal studies | | | | | |
| **Index test(s)** | POCUS (different windows), absence of cardiac activity | | | | | | |
| **Reference standard(s)** | Survival | | | | | | |
| **DTA-SR characteristics** | ***No. of studies*** | 10 | | | | | |
|  | ***No. of participants*** | NA | | | | | |
|  | ***Year range*** | 2001 - 2016 | | | | | |
|  | ***Quality assessment*** | QUADAS-2, overall low risk of bias | | | | | |
|  | ***Special remark*** |  | | | | | |
| **Diagnostic accuracy** | ***Measure*** | Sensitivity [%] (95% CI):  90.0 (83.0; 94.0) | | | Specificity [%] (95% CI):  78.0 (64.0; 88.0) | | |
|  | ***GRADE*** | Very low ⊖⊖⊕⊖ | | | Very low ⊖⊖⊕⊖ | | |
| **Method. quality**  **(mod AMSTAR2)** | **Overall** | Low | | | | | |
| **Further investigations, sub- or sensitivity analyses** | **Index test** | | **Reference standard** | **Studies (Particip.)** | | **Sens. [%], 95% CI** | **Spec. [%], 95% CI** |
|  |  | |  |  | |  |  |
|  |  | |  |  | |  |  |
|  |  | |  |  | |  |  |
|  |  | |  |  | |  |  |
| **Conclusions of study authors** | *“Point-of-care focused echocardiography can be used to identify reversible causes and predict short-term outcome in patients with cardiac arrest. In patients with a low pretest probability for return of spontaneous circulation, absence of spontaneous cardiac movement on echocardiography can predict a low likelihood of survival and guide the decision of resuscitation termination.”* | | | | | | |

| **Jian (2025)**[56] – Pulseless electrical activity | | | | | | | |
| --- | --- | --- | --- | --- | --- | --- | --- |
| **Clinical context** | ***Patient population*** | Adults and children with pulseless electric activity | | | | | |
|  | ***Clinical setting*** | Mostly out of hospital cardiac arrest | | | | | |
|  | ***Prevalence [%] (CI)*** | Not documented | | | | | |
|  | ***Noteworthy incl. crit.*** | Prospective or retrospective diagnostic studies, conducted in pre-hospital or hospital settings, utilize transthoracic echocardiography to predict resuscitation outcomes, specifically one of the following: return of spontaneous circulation, sudden hemodynamic arrest, severe hemodynamic deterioration | | | | | |
|  | ***Noteworthy excl. crit.*** | Case reports, case series, conference abstracts, animal studies, reviews, and studies with duplicate subjects | | | | | |
| **Index test(s)** | TTE | | | | | | |
| **Reference standard(s)** | Follow-up (return of spontaneous circulation) | | | | | | |
| **DTA-SR characteristics** | ***No. of studies*** | 10 | | | | | |
|  | ***No. of participants*** | 732 | | | | | |
|  | ***Year range*** | 2005 - 2021 | | | | | |
|  | ***Quality assessment*** | QUADAS-2 (7 studies with high risk of bias) | | | | | |
|  | ***Special remark*** |  | | | | | |
| **Diagnostic accuracy** | ***Measure*** | Sensitivity [%] (95% CI):  86.0 (67.0; 95.0) | | | Specificity [%] (95% CI):  64.0 (51.0; 75.0) | | |
|  | ***GRADE*** | Very low ⊕⊖⊖⊖ | | | Very low ⊕⊖⊖⊖ | | |
| **Method. quality**  **(mod AMSTAR2)** | **Overall** | Moderate | | | | | |
| **Further investigations, sub- or sensitivity analyses** | **Index test** | | **Reference standard** | **Studies (Particip.)** | | **Sens. [%], 95% CI** | **Spec. [%], 95% CI** |
|  | TTE | | Follow-up (sudden hemodynamic arrest) | 13 (975) | | 89 (80; 94) | 73 (63; 81) |
|  | TTE | | Follow-up (sever hemodynamic deterioration) | 9 (82) | | 79 (58; 91) | 58 (47; 61) |
|  |  | |  |  | |  |  |
|  |  | |  |  | |  |  |
| **Conclusions of study authors** | *“Our study suggests that POCUS may serve as a vital component of a multimodal approach for early termination of resuscitation.”* | | | | | | |

| **Lee (2019)**[57] – Post-cardiac arrest neurological outcome | | | | | | | |
| --- | --- | --- | --- | --- | --- | --- | --- |
| **Clinical context** | ***Patient population*** | Adults | | | | | |
|  | ***Clinical setting*** | Inhospital | | | | | |
|  | ***Prevalence [%] (CI)*** | Not documented | | | | | |
|  | ***Noteworthy incl. crit.*** | Use of mean optical nerve ultrasound, ocular ultrasound or brain CT as index test, sufficient data for 2x2 tables, original research articles | | | | | |
|  | ***Noteworthy excl. crit.*** | - | | | | | |
| **Index test(s)** | Optical ultrasound | | | | | | |
| **Reference standard(s)** | Glasgow-Pittsburg Cerebral Performance Categories, Glascow Outcome Scale | | | | | | |
| **DTA-SR characteristics** | ***No. of studies*** | 3 | | | | | |
|  | ***No. of participants*** | Not documented | | | | | |
|  | ***Year range*** | 2015 - 2018 | | | | | |
|  | ***Quality assessment*** | QUADAS-2, overall low risk of bias | | | | | |
|  | ***Special remark*** | Absolute numbers and free-text results don’t match | | | | | |
| **Diagnostic accuracy** | ***Measure*** | Sensitivity [%] (95% CI):  77.0 (53.0; 100) | | | Specificity [%] (95% CI):  98.0 (83.0; 100) | | |
|  | ***GRADE*** | Low ⊕⊖⊕⊖ | | | Low ⊕⊖⊕⊖ | | |
| **Method. quality**  **(mod AMSTAR2)** | **Overall** | Moderate | | | | | |
| **Further investigations, sub- or sensitivity analyses** | **Index test** | | **Reference standard** | **Studies (Particip.)** | | **Sens. [%], 95% CI** | **Spec. [%], 95% CI** |
|  |  | |  |  | |  |  |
|  |  | |  |  | |  |  |
|  |  | |  |  | |  |  |
|  |  | |  |  | |  |  |
| **Conclusions of study authors** | *“The present meta-analysis revealed that the optic nerve sheath diameter may be a useful method for predicting neurologic outcomes in post–cardiac arrest patients. For increased accuracy, measurement of the optic nerve sheath diameter should be performed using ocular ultrasound, the method should be limited to patients with cardiac-origin cardiac arrest, and the Cerebral Performance Category should be used for neurologic outcome evaluation.”* | | | | | | |

###### Endocarditis

| **Bai (2017)**[58] – Infective endocarditis | | | | | | | |
| --- | --- | --- | --- | --- | --- | --- | --- |
| **Clinical context** | ***Patient population*** | Adults | | | | | |
|  | ***Clinical setting*** | Not documented | | | | | |
|  | ***Prevalence [%] (CI)*** | 28 (-) | | | | | |
|  | ***Noteworthy incl. crit.*** | Patients with and without infective endocarditis, sufficient data for 2x2 table | | | | | |
|  | ***Noteworthy excl. crit.*** | - | | | | | |
| **Index test(s)** | TTE harmonic | | | | | | |
| **Reference standard(s)** | TEE | | | | | | |
| **DTA-SR characteristics** | ***No. of studies*** | 7 | | | | | |
|  | ***No. of participants*** | Not documented | | | | | |
|  | ***Year range*** | 2005 - 2016 | | | | | |
|  | ***Quality assessment*** | QUADAS; substantial risk of bias | | | | | |
|  | ***Special remark*** | Included patients with and without endocarditis | | | | | |
| **Diagnostic accuracy** | ***Measure*** | Sensitivity [%] (95% CI):  61.0 (45.0; 75.0) | | | Specificity [%] (95% CI):  94.0 (85.0; 98.0) | | |
|  | ***GRADE*** | Low ⊕⊕⊖⊖ | | | Low ⊕⊕⊖⊖ | | |
| **Method. quality**  **(mod AMSTAR2)** | **Overall** | Moderate | | | | | |
| **Further investigations, sub- or sensitivity analyses** | **Index test** | | **Reference standard** | **Studies (Particip.)** | | **Sens. [%], 95% CI** | **Spec. [%], 95% CI** |
|  | Fundamental TTE | | TEE | 9 (NA) | | 41 (29; 55) | 96 (90; 99) |
|  | Harmonic TTE, native valves only | | TEE | 6 (NA) | | 66 (51; 79) | 95 (85;98) |
|  | Harmonic TTE, positive or indeterminate TTE was rated as positive result | | TEE | 6 (NA) | | 90 (80; 96) | 58 (36; 78) |
|  |  | |  |  | |  |  |
| **Conclusions of study authors** | *“Modern harmonic TTE still has the potential to miss many vegetations compared with TEE. Conclusively negative results on TTE recorded with optimal imaging views in patients without prosthetic valves greatly decreases likelihood of IE and can aid in ruling out IE. All other transthoracic results are not useful for ruling out IE, and subsequent TEE is almost always required.”* | | | | | | |

| **Bonzi (2018)**[59] – Native valve infective endocarditis | | | | | | | |
| --- | --- | --- | --- | --- | --- | --- | --- |
| **Clinical context** | ***Patient population*** | Adults | | | | | |
|  | ***Clinical setting*** | Not documented | | | | | |
|  | ***Prevalence [%] (CI)*** | Not documented | | | | | |
|  | ***Noteworthy incl. crit.*** | Both TTE and TEE performed, sufficient data for 2x2 table, Studies that included both patients with native and prosthetic valves were included if data about native valves could be extracted separately | | | | | |
|  | ***Noteworthy excl. crit.*** | - | | | | | |
| **Index test(s)** | TTE (fundamental and harmonic) | | | | | | |
| **Reference standard(s)** | TEE | | | | | | |
| **DTA-SR characteristics** | ***No. of studies*** | 11 | | | | | |
|  | ***No. of participants*** | 2209 | | | | | |
|  | ***Year range*** | 1990 - 2016 | | | | | |
|  | ***Quality assessment*** | QUADAS-2; overall substantial risk of bias | | | | | |
|  | ***Special remark*** | Study presents a diagnostic algorithm for suspected IE based on pre-test probability and TTE results  Reported patients and 2x2 table results don’t match | | | | | |
| **Diagnostic accuracy** | ***Measure*** | Sensitivity [%] (95% CI):  71.0 (56.0; 82.0) | | | Specificity [%] (95% CI):  80.0 (58.0; 92.0) | | |
|  | ***GRADE*** | Very low ⊖⊖⊖⊖ | | | Very low ⊖⊖⊖⊖ | | |
| **Method. quality**  **(mod AMSTAR2)** | **Overall** | Low | | | | | |
| **Further investigations, sub- or sensitivity analyses** | **Index test** | | **Reference standard** | **Studies (Particip.)** | | **Sens. [%], 95% CI** | **Spec. [%], 95% CI** |
|  | TTE single cut-off | | TEE | 5 (NA) | | 73 (55; 86) | 94 (90; 97) |
|  | TTE strict negative criteria | | TEE | 6 (NA) | | 94 (88; 97) | 57 (46; 67) |
|  | TTE standard criteria | | TEE | 6 (NA) | | 64 (48; 77) | 98 (90; 99) |
|  |  | |  |  | |  |  |
| **Conclusions of study authors** | *“Our study results support guidelines and previous evidence-based data in considering a negative TTE as a single rule-out test in the presence of low pre-test probability of IE. The possibility also excluding IE in intermediate risk patients with either the adoption of strictly negative criteria or a clinical suspicion of right heart IE should be taken into account. On the other hand, a positive TTE might be considered as a single rule-in test in patients with a highly suggestive clinical picture, and in whom either the probability of complications is low, or the detection of complications would not change the clinical management.”* | | | | | | |

| **Habets (2015)**[60] – Prosthetic heart valve endocarditis | | | | | | | |
| --- | --- | --- | --- | --- | --- | --- | --- |
| **Clinical context** | ***Patient population*** | Not documented | | | | | |
|  | ***Clinical setting*** | Not documented | | | | | |
|  | ***Prevalence [%] (CI)*** | Not documented | | | | | |
|  | ***Noteworthy incl. crit.*** | - | | | | | |
|  | ***Noteworthy excl. crit.*** | Studies performed before 1985 | | | | | |
| **Index test(s)** | TTE, periannular complications | | | | | | |
| **Reference standard(s)** | Surgical inspection/autopsy or clinical follow-up | | | | | | |
| **DTA-SR characteristics** | ***No. of studies*** | 9 | | | | | |
|  | ***No. of participants*** | 172 | | | | | |
|  | ***Year range*** | 1989 - 2013 | | | | | |
|  | ***Quality assessment*** | QUADAS-2, several studies with high risk of bias | | | | | |
|  | ***Special remark*** | Compares TTE to TEE and multidetector-row computed tomography | | | | | |
| **Diagnostic accuracy** | ***Measure*** | Sensitivity [%] (95% CI):  36.0 (27.0; 46.0) | | | Specificity [%] (95% CI):  93.0 (84.0; 97.0) | | |
|  | ***GRADE*** | Very low ⊖⊖⊖⊖ | | | Very low ⊖⊖⊖⊖ | | |
| **Method. quality**  **(mod AMSTAR2)** | **Overall** | Low | | | | | |
| **Further investigations, sub- or sensitivity analyses** | **Index test** | | **Reference standard** | **Studies (Particip.)** | | **Sens. [%], 95% CI** | **Spec. [%], 95% CI** |
|  | TTE, vegetations | | Surgery, autopsy, follow-up | 6 (63) | | 29 (9;62) | 100 (86; 100) |
|  | TTE, general signs | | Surgery, autopsy, follow-up | NA (55) | | 33 (24; 42) | 100 (76; 100) |
|  | TTE, PHV dehiscence | | Surgery, autopsy, follow-up | NA (18) | | 11 (1; 73) | 100 (72; 100) |
|  |  | |  |  | |  |  |
| **Conclusions of study authors** | *“In this meta-analysis, TEE showed good sensitivity (86%), and because of its better spatial resolution and the close relationship between the TEE probe and the heart, it was proven to be superior to TTE (36%) for the detection of abscesses and mycotic aneurysms.”* | | | | | | |

###### Heart failure

| **Al Deeb (2014)**[61] – Acute heart failure | | | | | | | |
| --- | --- | --- | --- | --- | --- | --- | --- |
| **Clinical context** | ***Patient population*** | Not documented | | | | | |
|  | ***Clinical setting*** | Emergency department, intensive care unit, ward, prehospital emergency setting | | | | | |
|  | ***Prevalence [%] (CI)*** | Not documented | | | | | |
|  | ***Noteworthy incl. crit.*** | Prospective cohort and prospective case-control studies, patients with undifferentiated acute dyspnea, B-Lines for diagnosis, clinical follow-up as reference standard | | | | | |
|  | ***Noteworthy excl. crit.*** | - | | | | | |
| **Index test(s)** | LUS, B-Lines | | | | | | |
| **Reference standard(s)** | Clinical follow-up | | | | | | |
| **DTA-SR characteristics** | ***No. of studies*** | 7 | | | | | |
|  | ***No. of participants*** | 1075 | | | | | |
|  | ***Year range*** | 1998 - 2012 | | | | | |
|  | ***Quality assessment*** | QUADAS-2, all average to high quality studies | | | | | |
|  | ***Special remark*** | - | | | | | |
| **Diagnostic accuracy** | ***Measure*** | Sensitivity [%] (95% CI):  94.1 (81.3; 98.3) | | | Specificity [%] (95% CI):  92.4 (84.2; 96.4) | | |
|  | ***GRADE*** | Low ⊕⊖⊕⊖ | | | Low ⊕⊖⊕⊖ | | |
| **Method. quality**  **(mod AMSTAR2)** | **Overall** | Moderate | | | | | |
| **Further investigations, sub- or sensitivity analyses** | **Index test** | | **Reference standard** | **Studies (Particip.)** | | **Sens. [%], 95% CI** | **Spec. [%], 95% CI** |
|  | LUS, sensitivity analysis | | Clinical follow-up | 4 (575) | | 93 (82; 98) | 89 (79; 95) |
|  |  | |  |  | |  |  |
|  |  | |  |  | |  |  |
|  |  | |  |  | |  |  |
| **Conclusions of study authors** | *“The results of this meta-analysis suggest that POCUS using B-lines may aid clinicians in the diagnosis of acute cardiogenic pulmonary edema.”* | | | | | | |

| **Chiu (2022)**[62] – Acute heart failure | | | | | | | |
| --- | --- | --- | --- | --- | --- | --- | --- |
| **Clinical context** | ***Patient population*** | Adults | | | | | |
|  | ***Clinical setting*** | Emergency department, ward | | | | | |
|  | ***Prevalence [%] (CI)*** | 41 - 72% | | | | | |
|  | ***Noteworthy incl. crit.*** | Comparing both LUS and chest X-ray | | | | | |
|  | ***Noteworthy excl. crit.*** | - | | | | | |
| **Index test(s)** | Lung ultrasound, B-Lines | | | | | | |
| **Reference standard(s)** | Medical record audit | | | | | | |
| **DTA-SR characteristics** | ***No. of studies*** | 8 | | | | | |
|  | ***No. of participants*** | 2563 | | | | | |
|  | ***Year range*** | 2011 - 2020 | | | | | |
|  | ***Quality assessment*** | QUADAS-2; overall substantial risk of bias | | | | | |
|  | ***Special remark*** | Compares LUS to chest x-ray against medical record audit | | | | | |
| **Diagnostic accuracy** | ***Measure*** | Sensitivity [%] (95% CI):  91.8 (80.5; 96.7) | | | Specificity [%] (95% CI):  92.3 (86.6; 95.7) | | |
|  | ***GRADE*** | Very low ⊖⊖⊕⊖ | | | Low ⊖⊖⊕⊕ | | |
| **Method. quality**  **(mod AMSTAR2)** | **Overall** | Low | | | | | |
| **Further investigations, sub- or sensitivity analyses** | **Index test** | | **Reference standard** | **Studies (Particip.)** | | **Sens. [%], 95% CI** | **Spec. [%], 95% CI** |
|  | LUS, sensitivity analysis | | Medical record audit | 7 (2345) | | 92 (79; 98) | 92 (85; 96) |
|  |  | |  |  | |  |  |
|  |  | |  |  | |  |  |
|  |  | |  |  | |  |  |
| **Conclusions of study authors** | *„In conclusion, these findings suggest that LUS is more sensitive and specific than CXR in detecting pulmonary edema. This highlights the importance of sonographic B-lines, along with the accurate interpretation of clinical data, in the diagnosis of acute decongested heart failure. Therefore, LUS should be considered an alternative to chest x-ray for evaluating patients with dyspnea in the setting of acute decongested heart failure.* | | | | | | |

| **Gartlehner (2021)**[63] – Acute heart failure | | | | | | | |
| --- | --- | --- | --- | --- | --- | --- | --- |
| **Clinical context** | ***Patient population*** | Adult patients with acute dyspnea | | | | | |
|  | ***Clinical setting*** | Emergency department | | | | | |
|  | ***Prevalence [%] (CI)*** | 4 - 62 | | | | | |
|  | ***Noteworthy incl. crit.*** | At least 85% of study participants had to report acute dyspnea. The target diseases of interest were congestive heart failure. | | | | | |
|  | ***Noteworthy excl. crit.*** | - | | | | | |
| **Index test(s)** | POCUS ultrasound of the lung | | | | | | |
| **Reference standard(s)** | Independent chart review by experts | | | | | | |
| **DTA-SR characteristics** | ***No. of studies*** | 5 | | | | | |
|  | ***No. of participants*** | 708 | | | | | |
|  | ***Year range*** | 2013 – 2020 | | | | | |
|  | ***Quality assessment*** | QUADAS-2 (4 with low, one with unclear risk of bias) | | | | | |
|  | ***Special remark*** | Ultrasound performed by internists or emergency physicians | | | | | |
| **Diagnostic accuracy** | ***Measure*** | Sensitivity [%] (95% CI):  76.0 (48.0; 91.0) | | | Specificity [%] (95% CI):  96.0 (90.0; 98.0) | | |
|  | ***GRADE*** | Moderate ⊕⊕⊖⊕ | | | Moderate ⊕⊕⊖⊕ | | |
| **Method. quality**  **(mod AMSTAR2)** | **Overall** | Moderate | | | | | |
| **Further investigations, sub- or sensitivity analyses** | **Index test** | | **Reference standard** | **Studies (Particip.)** | | **Sens. [%], 95% CI** | **Spec. [%], 95% CI** |
|  |  | |  |  | |  |  |
|  |  | |  |  | |  |  |
|  |  | |  |  | |  |  |
|  |  | |  |  | |  |  |
| **Conclusions of study authors** | *“In conclusion, evidence from RCTs indicates that POCUS can improve the correctness of diagnosis in patients with acute dyspnea.”* | | | | | | |

| **Lian (2018)**[64] – Acute heart failure | | | | | | | |
| --- | --- | --- | --- | --- | --- | --- | --- |
| **Clinical context** | ***Patient population*** | Adults | | | | | |
|  | ***Clinical setting*** | Emergency department and prehospital emergency setting | | | | | |
|  | ***Prevalence [%] (CI)*** | Not documented | | | | | |
|  | ***Noteworthy incl. crit.*** | Patients with complaints of acute dyspnea or clinical suspected AHF, US in the emergency department or pre hospital emergency settings, ultrasound lung comets as index text, sufficient data for 2x2 table | | | | | |
|  | ***Noteworthy excl. crit.*** | - | | | | | |
| **Index test(s)** | LUS, ultrasound lung comets | | | | | | |
| **Reference standard(s)** | Comprehensive examination results, final diagnosis | | | | | | |
| **DTA-SR characteristics** | ***No. of studies*** | 15 | | | | | |
|  | ***No. of participants*** | 3309 | | | | | |
|  | ***Year range*** | 2006 - 2016 | | | | | |
|  | ***Quality assessment*** | QUADAS-2; overall low risk of bias | | | | | |
|  | ***Special remark*** | - | | | | | |
| **Diagnostic accuracy** | ***Measure*** | Sensitivity [%] (95% CI):  85.0 (84.0; 87.0) | | | Specificity [%] (95% CI):  91.0 (89.0; 92.0) | | |
|  | ***GRADE*** | Moderate ⊕⊖⊕⊕ | | | Moderate ⊕⊖⊕⊕ | | |
| **Method. quality**  **(mod AMSTAR2)** | **Overall** | Moderate | | | | | |
| **Further investigations, sub- or sensitivity analyses** | **Index test** | | **Reference standard** | **Studies (Particip.)** | | **Sens. [%], 95% CI** | **Spec. [%], 95% CI** |
|  | LUS > 90 min admission to examination | | Comprehensive examination results, final diagnosis | 5 (NA) | | 67 (61; 72 | 90 (87; 92) |
|  | LUS < 90 min admission to examination | | Comprehensive examination results, final diagnosis | 8 (NA) | | 91 (89; 92) | 90 (89; 92) |
|  |  | |  |  | |  |  |
|  |  | |  |  | |  |  |
| **Conclusions of study authors** | *“This study strongly supports the routine detection of AHF using TLS performed at the bedside and early test performance during the evaluation of patients presenting to the ED with acute dyspnea together with routine examinations.”* | | | | | | |

| **Lv (2024)**[65] – Acute heart failure | | | | | | | |
| --- | --- | --- | --- | --- | --- | --- | --- |
| **Clinical context** | ***Patient population*** | Adults with dyspnea or suspected heart failure | | | | | |
|  | ***Clinical setting*** | Not documented | | | | | |
|  | ***Prevalence [%] (CI)*** | 27.6 (26.3; 28.9) | | | | | |
|  | ***Noteworthy incl. crit.*** | English-language studies of the last 10 years, diagnostic studies with echocardiography, reliable reference standard (expert consensus or final diagnosis); sample size ≥25 | | | | | |
|  | ***Noteworthy excl. crit.*** | Case reports, abstracts, letters, editorials; reviews/meta-analyses, pediatric or animal studies; unclear diagnosis, missing data | | | | | |
| **Index test(s)** | POCUS (left ventricular ejection fracture, B-lines, inferior vena cava) | | | | | | |
| **Reference standard(s)** | Final diagnosis, expert consensus | | | | | | |
| **DTA-SR characteristics** | ***No. of studies*** | 18 | | | | | |
|  | ***No. of participants*** | 4390 | | | | | |
|  | ***Year range*** | 2012 - 2019 | | | | | |
|  | ***Quality assessment*** | QUADAS-2 (9 studies with high risk of bias) | | | | | |
|  | ***Special remark*** |  | | | | | |
| **Diagnostic accuracy** | ***Measure*** | Sensitivity [%] (95% CI):  92.0 (81.0; 97.0) | | | Specificity [%] (95% CI):  96.0 (93.0; 98.0) | | |
|  | ***GRADE*** | Very low ⊕⊖⊖⊖ | | | Low ⊕⊖⊖⊕ | | |
| **Method. quality**  **(mod AMSTAR2)** | **Overall** | Moderate | | | | | |
| **Further investigations, sub- or sensitivity analyses** | **Index test** | | **Reference standard** | **Studies (Particip.)** | | **Sens. [%], 95% CI** | **Spec. [%], 95% CI** |
|  | POCUS (LVEF < 40%, B-lines, IVC) | | Final diagnosis, expert consensus | 6 (NA) | | 93 (74; 98) | 96 (93; 98) |
|  | POCUS (LVEF < 45%, B-lines, IVC) | | Final diagnosis, expert consensus | 7 (NA) | | 79 (47; 94) | 97 (87; 99) |
|  |  | |  |  | |  |  |
|  |  | |  |  | |  |  |
| **Conclusions of study authors** | *“Echocardiography is highly accurate in diagnosing acute heart failure, underscored by its critical role in early treatment decisions and potential integration into standard care protocols, thereby preventing adverse outcomes and improving patient management.”* | | | | | | |

| **Martindale (2016)**[66] – Acute heart failure | | | | | | | |
| --- | --- | --- | --- | --- | --- | --- | --- |
| **Clinical context** | ***Patient population*** | Adults | | | | | |
|  | ***Clinical setting*** | Emergency department | | | | | |
|  | ***Prevalence [%] (CI)*** | 48.2 (46.0; 50.5) | | | | | |
|  | ***Noteworthy incl. crit.*** | Studies which focused on the diagnosis of acute heart failure in the emergency department (any diagnostic test) | | | | | |
|  | ***Noteworthy excl. crit.*** | Compensated, chronic heart failure or prognostic studies | | | | | |
| **Index test(s)** | Lung ultrasound (no protocol, but at least 3 B-lines in two bilateral lung zones) | | | | | | |
| **Reference standard(s)** | Clinical chart review (blinded) | | | | | | |
| **DTA-SR characteristics** | ***No. of studies*** | 8 | | | | | |
|  | ***No. of participants*** | 1918 | | | | | |
|  | ***Year range*** | Until March 2015 | | | | | |
|  | ***Quality assessment*** | QUADAS-2 (high risk of bias in 4 studies) | | | | | |
|  | ***Special remark*** |  | | | | | |
| **Diagnostic accuracy** | ***Measure*** | Sensitivity [%] (95% CI):  85.3 (82.8, 87.5) | | | Specificity [%] (95% CI):  92.7 (90.0, 94.3) | | |
|  | ***GRADE*** | Moderate ⊕⊖⊕⊕ | | | Moderate ⊕⊖⊕⊕ | | |
| **Method. quality**  **(mod AMSTAR2)** | ***Overall*** | Moderate | | | | | |
| **Further investigations, sub- or sensitivity analyses** | **Index test** | | **Reference standard** | **Studies (Particip.)** | | **Sens. [%], 95% CI** | **Spec. [%], 95% CI** |
|  |  | |  |  | |  |  |
|  |  | |  |  | |  |  |
|  |  | |  |  | |  |  |
|  |  | |  |  | |  |  |
| **Conclusions of study authors** | *“Lung ultrasound appears to have the best combination of test characteristics with the presence or absence of diffuse B-lines providing reliable information to confirm or exclude the emergency diagnosis of acute heart failure.”* | | | | | | |

| **Mc Givery (2018)**[67] –Acute heart failure | | | | | | | |
| --- | --- | --- | --- | --- | --- | --- | --- |
| **Clinical context** | ***Patient population*** | Not documented | | | | | |
|  | ***Clinical setting*** | Emergency department | | | | | |
|  | ***Prevalence [%] (CI)*** | Not documented | | | | | |
|  | ***Noteworthy incl. crit.*** | Studies including dyspneic ED patients | | | | | |
|  | ***Noteworthy excl. crit.*** | - | | | | | |
| **Index test(s)** | Lung ultrasound (Volpicelli method) | | | | | | |
| **Reference standard(s)** | Chart review based on all assessments besides lung ultrasound | | | | | | |
| **DTA-SR characteristics** | ***No. of studies*** | 7 | | | | | |
|  | ***No. of participants*** | 1861 | | | | | |
|  | ***Year range*** | Search until August 2016 | | | | | |
|  | ***Quality assessment*** | CASP-Checklist, low risk of bias in all included studies | | | | | |
|  | ***Special remark*** |  | | | | | |
| **Diagnostic accuracy** | ***Measure*** | Sensitivity [%] (95% CI):  82.5 (66.4; 91.8) | | | Specificity [%] (95% CI):  83.6 (72.4;90.8) | | |
|  | ***GRADE*** | Low ⊕⊖⊕⊖ | | | Moderate ⊕⊖⊕⊕ | | |
| **Method. quality**  **(mod AMSTAR2)** | ***Overall*** | Moderate | | | | | |
| **Further investigations, sub- or sensitivity analyses** | **Index test** | | **Reference standard** | **Studies (Particip.)** | | **Sens. [%], 95% CI** | **Spec. [%], 95% CI** |
|  | Specialist only ultrasound (no residents or students) | | Same as above | 5 (NA) | | 89 (80; 94) | 83 (63; 94) |
|  |  | |  |  | |  |  |
|  |  | |  |  | |  |  |
|  |  | |  |  | |  |  |
| **Conclusions of study authors** | *“The included studies suggest that bedside lung ultrasound may serve as a reliable diagnostic adjunct for emergency department patients with undifferentiated dyspnea for whom physicians have a clinical suspicion of acute decongested heart failure. Based on our meta-analysis, we suggest performing early bedside lung US and considering the initiation of treatment, while awaiting additional test results for dyspneic patients. For the skilled sonographer, we would recommend incorporating bedside echocardiography inferior vena cava measurement to delineate further the cause of pulmonary edema, as this may improve the accuracy of isolated B-lines.”* | | | | | | |

| **Russell (2024)**[68] – Acute heart failure | | | | | | | |
| --- | --- | --- | --- | --- | --- | --- | --- |
| **Clinical context** | ***Patient population*** | Adults and children with suspected acute decompensated heart failure | | | | | |
|  | ***Clinical setting*** | Prehospital | | | | | |
|  | ***Prevalence [%] (CI)*** | 42.4 (38.7; 46.2) | | | | | |
|  | ***Noteworthy incl. crit.*** | Lung ultrasound was performed and interpreted in the prehospital setting for the diagnosis of acute decompensated heart failure. | | | | | |
|  | ***Noteworthy excl. crit.*** | Concern for trauma, or if the study included under 10 patients | | | | | |
| **Index test(s)** | Lung ultrasound (5x more than 2 B-lines, 1x more than 3 B-lines, 2x unknown) | | | | | | |
| **Reference standard(s)** | Final hospital diagnosis, emergency department diagnosis | | | | | | |
| **DTA-SR characteristics** | ***No. of studies*** | 8 | | | | | |
|  | ***No. of participants*** | 692 | | | | | |
|  | ***Year range*** | 2011 – 2023 | | | | | |
|  | ***Quality assessment*** | QUADAS-2 (6 studies with high risk of bias) | | | | | |
|  | ***Special remark*** |  | | | | | |
| **Diagnostic accuracy** | ***Measure*** | Sensitivity [%] (95% CI):  86.7 (70.8; 94.6) | | | Specificity [%] (95% CI):  87.5 (78.2; 93.2) | | |
|  | ***GRADE*** | Low ⊕⊕⊖⊖ | | | Low ⊕⊕⊖⊖ | | |
| **Method. quality**  **(mod AMSTAR2)** | **Overall** | Moderate | | | | | |
| **Further investigations, sub- or sensitivity analyses** | **Index test** | | **Reference standard** | **Studies (Particip.)** | | **Sens. [%], 95% CI** | **Spec. [%], 95% CI** |
|  |  | |  |  | |  |  |
|  |  | |  |  | |  |  |
|  |  | |  |  | |  |  |
|  |  | |  |  | |  |  |
| **Conclusions of study authors** | *“In this systematic review and meta-analysis, we found prehospital lung ultrasound to have similar diagnostic test characteristics to emergency department performed lung ultrasound for the diagnosis of acute decompensated heart failure in dyspneic patients.”* | | | | | | |

| **Staub (2019)**[69] – Acute heart failure, pneumonia and exacerbated COPD | | | | | | | |
| --- | --- | --- | --- | --- | --- | --- | --- |
| **Clinical context** | ***Patient population*** | Adults (>16 years) | | | | | |
|  | ***Clinical setting*** | Emergency department and intensive care unit | | | | | |
|  | ***Prevalence [%] (CI)*** | 44.2 (41.4; 45.9) | | | | | |
|  | ***Noteworthy incl. crit.*** | Prospective diagnostic accuracy study, LUS with description of the sonographic signs of positivity; target disease: Pneumonia, acute heart failure or exacerbation of COPD or asthma | | | | | |
|  | ***Noteworthy excl. crit.*** | - | | | | | |
| **Index test(s)** | LUS, diffuse interstitial syndrome | | | | | | |
| **Reference standard(s)** | Final diagnosis by experts including radiological imaging | | | | | | |
| **DTA-SR characteristics** | ***No. of studies*** | 8 | | | | | |
|  | ***No. of participants*** | 1208 | | | | | |
|  | ***Year range*** | 2008 - 2016 | | | | | |
|  | ***Quality assessment*** | QUADAS-2 (All studies had a high or unclear risk of bias in at least one domain; mostly patient selection) | | | | | |
|  | ***Special remark*** |  | | | | | |
| **Diagnostic accuracy** | ***Measure*** | Sensitivity [%] (95% CI):  73.0 (48.0; 89.0) | | | Specificity [%] (95% CI):  84.0 (76.0; 90.0) | | |
|  | ***GRADE*** | Low ⊕⊖⊕⊖ | | | Low ⊕⊖⊕⊖ | | |
| **Method. quality**  **(mod AMSTAR2)** | **Overall** | Moderate | | | | | |
| **Further investigations, sub- or sensitivity analyses** | **Index test** | | **Reference standard** | **Studies (Particip.)** | | **Sens. [%], 95% CI** | **Spec. [%], 95% CI** |
|  | Lung ultrasound (looking for B profile/acute heart failure) | | Final diagnosis | 6 | | 82 (55; 94) | 90 (87;92) |
|  | Lung ultrasound (looking for consolidation/pneumonia) | | Final diagnosis | 6 | | 82 (74; 88) | 94 (85;98) |
|  | Lung ultrasound (looking for C-profile/pneumonia) | | Final diagnosis | 3 | | 24 (18; 32) | 97 (89; 99) |
|  | Lung ultrasound (looking for Exacerbation of COPD) | | Final diagnosis | 4 | | 78 (67, 86) | 94 (89;97) |
| **Conclusions of study authors** | *“LUS has high accuracy for pneumonia, acute heart failure, and exacerbation of COPD/asthma, and seems to be a valuable complement to other diagnostic methods in the emergency diagnosis of these diseases in adults with respiratory symptoms. The interpretation of the LUS examination should take into account the accuracy of the individual sonographic signs to diagnose pneumonia and acute heart failure.”* | | | | | | |

| **Squizzato (2021)**[70] – Acute heart failure | | | | | | | |
| --- | --- | --- | --- | --- | --- | --- | --- |
| **Clinical context** | ***Patient population*** | Adults and children | | | | | |
|  | ***Clinical setting*** | Emergency department | | | | | |
|  | ***Prevalence [%] (CI)*** | 50.8 (46.8; 54.9) | | | | | |
|  | ***Noteworthy incl. crit.*** | Studies evaluating IVC-Collapsibility index as stand-alone test of patients with acute dyspnea; only prospective cohort studies | | | | | |
|  | ***Noteworthy excl. crit.*** | - | | | | | |
| **Index test(s)** | Measurement of VCI | | | | | | |
| **Reference standard(s)** | At least two of the heart failure criteria by ESC (Clinical, TTE, Rx, ECG, proBNP) | | | | | | |
| **DTA-SR characteristics** | ***No. of studies*** | 7 | | | | | |
|  | ***No. of participants*** | 591 | | | | | |
|  | ***Year range*** | 2009 - 2018 | | | | | |
|  | ***Quality assessment*** | QUADAS-2 (Only one high risk o bias domain in one study) | | | | | |
|  | ***Special remark*** |  | | | | | |
| **Diagnostic accuracy** | ***Measure*** | Sensitivity [%] (95% CI):  79.1 (86.5; 86.8) | | | Specificity [%] (95% CI):  81.8 (75; 87) | | |
|  | ***GRADE*** | Low ⊕⊖⊕⊖ | | | Moderate ⊕⊖⊕⊕ | | |
| **Method. quality**  **(mod AMSTAR2)** | **Overall** | Moderate | | | | | |
| **Further investigations, sub- or sensitivity analyses** | **Index test** | | **Reference standard** | **Studies (Particip.)** | | **Sens. [%], 95% CI** | **Spec. [%], 95% CI** |
|  | IVC diameter in M-Mode; min | | Heart failure criteria by ESC | 2 | | 79 (70; 87) | 92 (85; 97) |
|  | IVC diameter in M-Mode; max | | Heart failure criteria by ESC | 3 | | 74 (61; 86) | 82 (74; 88) |
|  | Only low risk of bias | | Heart failure criteria by ESC | NA | | 80 (72; 87) | 77 (63; 89) |
|  | 50% IVC-CIx cut-off | | Heart failure criteria by ESC | 3 | | 84 (74; 90) | 73 (61; 83) |
| **Conclusions of study authors** | *“Our findings suggest that the sensitivity and specificity of IVC-Collapsibility index are suboptimal to rule in or rule out heart failure diagnosis in patients with acute dyspnea in the ED setting. Therefore, physicians should be discouraged to use IVC-Collapsibility index as a stand-alone test to support clinical decision-making. Further studies are needed to better define the role of IVC-Collapsibility index as part of a complex diagnostic algorithm for the differential diagnosis of acute dyspnea, in particular when acute heart failure is suspected”* | | | | | | |

| **Huang (2025)**[71] – Left ventricular diastolic dysfunction | | | | | | | |
| --- | --- | --- | --- | --- | --- | --- | --- |
| **Clinical context** | ***Patient population*** | Adults | | | | | |
|  | ***Clinical setting*** | Emergency Department | | | | | |
|  | ***Prevalence [%] (CI)*** | Not documented | | | | | |
|  | ***Noteworthy incl. crit.*** | adult patients (>18 years) presenting to the emergency department with acute dyspnea or other clinical symptoms suggestive of acute heart failure | | | | | |
|  | ***Noteworthy excl. crit.*** | Patients with indeterminate classification. | | | | | |
| **Index test(s)** | Emergency physician-performed focused cardiac ultrasound, utilizing any view or Doppler parameter specifically for assessing diastolic function. | | | | | | |
| **Reference standard(s)** | Focused cardiac ultrasound clips reviewed by a cardiologist or accredited sonographer, formal TTE, or invasive hemodynamic assessment performed during the same clinical encounter | | | | | | |
| **DTA-SR characteristics** | ***No. of studies*** | 4 | | | | | |
|  | ***No. of participants*** | 298 | | | | | |
|  | ***Year range*** | 2012 - 2021 | | | | | |
|  | ***Quality assessment*** | QUADAS-2 | | | | | |
|  | ***Special remark*** | Heterogeneity not statistically assessed | | | | | |
| **Diagnostic accuracy** | ***Measure*** | Sensitivity [%] (95% CI):  94.0 (87.0; 97.0) | | | Specificity [%] (95% CI):  59.0 (42.0; 74.0) | | |
|  | ***GRADE*** | High ⊕⊕⊕⊕ | | | Low ⊕⊖⊕⊖ | | |
| **Method. quality (mod AMSTAR2)** | **Overall** | Moderate | | | | | |
| **Further investigations, sub- or sensitivity analyses** | **Index test** | | **Reference standard** | **Studies (Particip.)** | | **Sens. [%], 95% CI** | **Spec. [%], 95% CI** |
|  |  | |  |  | |  |  |
|  |  | |  |  | |  |  |
|  |  | |  |  | |  |  |
|  |  | |  |  | |  |  |
| **Conclusions of study authors** | *“Emergency physician-performed focused cardiac ultrasound exhibited excellent sensitivity and low negative likelihood ratios, supporting its use as an effective initial triage tool for ruling out diastolic dysfunction in emergency department patients with suspected acute HF. However, its moderate specificity and limited positive likelihood ratios hamper its standalone use as a definitive diagnostic modality for ruling in diastolic dysfunction”* | | | | | | |

| **Albaroudi (2022)**[72] – Left ventricular function | | | | | | | |
| --- | --- | --- | --- | --- | --- | --- | --- |
| **Clinical context** | ***Patient population*** | Adults | | | | | |
|  | ***Clinical setting*** | Emergency department | | | | | |
|  | ***Prevalence [%] (CI)*** | Not documented | | | | | |
|  | ***Noteworthy incl. crit.*** | Studies that assessed LVSF using transthoracic echocardiography by visual estimation, E-point septal separation, or an assessment of stroke volume/cardiac output using velocity time integral | | | | | |
|  | ***Noteworthy excl. crit.*** | Use of fractional shortening as assessment | | | | | |
| **Index test(s)** | Visual estimation of LVEF with TTE by emergency physician | | | | | | |
| **Reference standard(s)** | Visual estimation of LVEF with TTE by expert echographer | | | | | | |
| **DTA-SR characteristics** | ***No. of studies*** | 7 | | | | | |
|  | ***No. of participants*** | 688 | | | | | |
|  | ***Year range*** | 2002-2019 | | | | | |
|  | ***Quality assessment*** | QUADAS-2 (the quality of the included studies was low, and all studies were assessed as high risk of bias) | | | | | |
|  | ***Special remark*** | No meta-analysis of EPSS or VTI possible | | | | | |
| **Diagnostic accuracy** | ***Measure*** | Sensitivity [%] (95% CI):  89.0 (80.0; 94.0) | | | Specificity [%] (95% CI):  85.0 (80.0; 89.0) | | |
|  | ***GRADE*** | Low ⊕⊖⊕⊖ | | | Moderate ⊕⊖⊕⊕ | | |
| **Method. quality**  **(mod AMSTAR2)** | **Overall** | Moderate | | | | | |
| **Further investigations, sub- or sensitivity analyses** | **Index test** | | **Reference standard** | **Studies (Particip.)** | | **Sens. [%], 95% CI** | **Spec. [%], 95% CI** |
|  |  | |  |  | |  |  |
|  |  | |  |  | |  |  |
|  |  | |  |  | |  |  |
|  |  | |  |  | |  |  |
| **Conclusions of study authors** | “*Clinician sonographer (trainee or attending) had a substantial agreement with an expert sonographer for the POC echocardiography assessment of left ventricular systolic function as ‘normal or abnormal’ and as ‘normal, reduced and severely reduced’ by visual estimation. Few studies assessed the agreement between clinician sonographer and expert sonographer for* *E-point septal separation and velocity time integral methods and meta-analysis was not possible.”* | | | | | | |

| **Asmara (2022)**[73] – Acute respiratory failure due to pulmonary edema | | | | | | | |
| --- | --- | --- | --- | --- | --- | --- | --- |
| **Clinical context** | ***Patient population*** | Adults | | | | | |
|  | ***Clinical setting*** | Intensive care unit, emergency department | | | | | |
|  | ***Prevalence [%] (CI)*** | Not documented | | | | | |
|  | ***Noteworthy incl. crit.*** | Cross-sectional design, comparing the diagnostic ability of the BLUE protocol with a gold standard, patients with acute respiratory failure | | | | | |
|  | ***Noteworthy excl. crit.*** | - | | | | | |
| **Index test(s)** | LUS (BLUE protocol) | | | | | | |
| **Reference standard(s)** | Echocardiography, radiography or clinical parameters | | | | | | |
| **DTA-SR characteristics** | ***No. of studies*** | 5 | | | | | |
|  | ***No. of participants*** | 770 | | | | | |
|  | ***Year range*** | 2008 - 2015 | | | | | |
|  | ***Quality assessment*** | QUADAS-2 (No studies with high risk of bias) | | | | | |
|  | ***Special remark*** |  | | | | | |
| **Diagnostic accuracy** | ***Measure*** | Sensitivity [%] (95% CI):  89.0 (81.0; 93.0) | | | Specificity [%] (95% CI):  94.0 (89.0; 96.0) | | |
|  | ***GRADE*** | Moderate ⊕⊕⊕⊖ | | | High ⊕⊕⊕⊕ | | |
| **Method. quality**  **(mod AMSTAR2)** | **Overall** | Moderate | | | | | |
| **Further investigations, sub- or sensitivity analyses** | **Index test** | | **Reference standard** | **Studies (Particip.)** | | **Sens. [%], 95% CI** | **Spec. [%], 95% CI** |
|  | BLUE protocol for pneumonia | | Echocardiography, radiography or clinic | 5 | | 84 (76; 89) | 98 (93; 99) |
|  | BLUE protocol for pneumothorax | | Echocardiography, radiography or clinic | 3 | | 87 (80; 95) | 99 (98; 100) |
|  | Blue protocol for pulmonary embolism | | Echocardiography, radiography or clinic | 3 | | 66 (31; 100) | 99 (98; 100) |
|  | Blue protocol for asthma/COPD | | Same as above | 3 | | 91 (79; 99) | 88 (77 100) |
| **Conclusions of study authors** | *“The BLUE protocol has high sensitivity and specificity in diagnosing pneumonia and pulmonary edema. These high diagnostic accuracy values came from a good quality study based on the GRADE approach. The BLUE protocol has high specificity in diagnose pneumothorax and pulmonary embolism but with varying sensitivity. This accuracy assessment comes from a poor-quality study based on the GRADE approach.”* | | | | | | |

| **Dong (2023)**[74] – Cardiogenic pulmonary edema | | | | | | | |
| --- | --- | --- | --- | --- | --- | --- | --- |
| **Clinical context** | ***Patient population*** | Adults | | | | | |
|  | ***Clinical setting*** | Not documented | | | | | |
|  | ***Prevalence [%] (CI)*** | 49.9 (47.7; 50.2) | | | | | |
|  | ***Noteworthy incl. crit.*** | Diagnostic cohort and case control studies on diagnostic accuracy of Lung ultrasound | | | | | |
|  | ***Noteworthy excl. crit.*** | Case reports, experiments, non-English | | | | | |
| **Index test(s)** | LUS, looking for B-Lines (no defined protocol) | | | | | | |
| **Reference standard(s)** | Discharge diagnosis, clinical diagnosis, expert panel, review of data | | | | | | |
| **DTA-SR characteristics** | ***No. of studies*** | 9 | | | | | |
|  | ***No. of participants*** | 2097 | | | | | |
|  | ***Year range*** | 2011 - 2019 | | | | | |
|  | ***Quality assessment*** | QUADAS-2 (all included were moderate or high) | | | | | |
|  | ***Special remark*** |  | | | | | |
| **Diagnostic accuracy** | ***Measure*** | Sensitivity [%] (95% CI):  92.0 (84.0; 97.0) | | | Specificity [%] (95% CI):  87.0 (82.0; 91.0) | | |
|  | ***GRADE*** | Very low ⊖⊖⊕⊖ | | | Low ⊖⊖⊕⊕ | | |
| **Method. quality**  **(mod AMSTAR2)** | ***Overall*** | Low | | | | | |
| **Further investigations, sub- or sensitivity analyses** | **Index test** | | **Reference standard** | **Studies (Particip.)** | | **Sens. [%], 95% CI** | **Spec. [%], 95% CI** |
|  | Four-zone method | | Same as above | 1 | | 97 (78;99) | 82 (46;96) |
|  | Six-Zone method | | Same as above | 1 | | 91 (55;99) | 93 (65;99) |
|  | Eight-Zone method | | Same as above | 3 | | 1 | 87 (75;95) |
|  | Twelve-Zone method | | Same as above | 4 | | 85 (63;96) | 83 (76;88) |
| **Conclusions of study authors** | *“LUS offers a good diagnostic efficacy for cardiogenic pulmonary edema. Further standardization of the examination method is required to provide a reference for the clinical use of LUS”* | | | | | | |

| **Maw (2019)**[75] – Cardiogenic pulmonary edema | | | | | | | |
| --- | --- | --- | --- | --- | --- | --- | --- |
| **Clinical context** | ***Patient population*** | Adults | | | | | |
|  | ***Clinical setting*** | Emergency department, medical ward | | | | | |
|  | ***Prevalence [%] (CI)*** | Not documented | | | | | |
|  | ***Noteworthy incl. crit.*** | Prospective cohorts of patients with acute dyspnea, both lung ultrasound and chest radiograph were performed on all patients | | | | | |
|  | ***Noteworthy excl. crit.*** | Overlapping or duplicate populations | | | | | |
| **Index test(s)** | Lung ultrasound | | | | | | |
| **Reference standard(s)** | Medical record (5 studies); echocardiography, BNP, and chest X-ray (1 study) | | | | | | |
| **DTA-SR characteristics** | ***No. of studies*** | 6 | | | | | |
|  | ***No. of participants*** | 1872 | | | | | |
|  | ***Year range*** | 2011 – 2017 | | | | | |
|  | ***Quality assessment*** | QUADAS-2 (no low risk of bias studies) | | | | | |
|  | ***Special remark*** |  | | | | | |
| **Diagnostic accuracy** | ***Measure*** | Sensitivity [%] (95% CI):  88.0 (75.0; 95.0) | | | Specificity [%] (95% CI):  90.0 (88.0; 92.0) | | |
|  | ***GRADE*** | Low ⊕⊖⊕⊖ | | | Moderate ⊕⊖⊕⊕ | | |
| **Method. quality**  **(mod AMSTAR2)** | **Overall** | Moderate | | | | | |
| **Further investigations, sub- or sensitivity analyses** | **Index test** | | **Reference standard** | **Studies (Particip.)** | | **Sens. [%], 95% CI** | **Spec. [%], 95% CI** |
|  |  | |  |  | |  |  |
|  |  | |  |  | |  |  |
|  |  | |  |  | |  |  |
|  |  | |  |  | |  |  |
| **Conclusions of study authors** | *"The findings suggest that lung ultrasound is as specific and more sensitive than chest X-ray in the identification of cardiogenic pulmonary edema. Given the potential advantages of its use, lung ultrasound should be considered as an adjunct imaging modality in the evaluation of patients with dyspnea at risk of acute decompensated heart failure.”* | | | | | | |

| **Wang (2018)**[76] – Pulmonary Edema | | | | | | | |
| --- | --- | --- | --- | --- | --- | --- | --- |
| **Clinical context** | ***Patient population*** | Patients with clinical suspicion of acute pulmonary edema and acute dyspnea | | | | | |
|  | ***Clinical setting*** | Emergency department (3 studies), intensive care unit (2), on a ward (2), prehospital (1) | | | | | |
|  | ***Prevalence [%] (CI)*** | 42.4 (38.7; 46.2) | | | | | |
|  | ***Noteworthy incl. crit.*** | Patient suspicion of acute pulmonary edema and acute dyspnea, bedside lung ultrasound by trained persons | | | | | |
|  | ***Noteworthy excl. crit.*** | - | | | | | |
| **Index test(s)** | LUS, (more than 2 B-lines) | | | | | | |
| **Reference standard(s)** | Final hospital diagnosis, emergency department diagnosis | | | | | | |
| **DTA-SR characteristics** | ***No. of studies*** | 8 | | | | | |
|  | ***No. of participants*** | 1301 | | | | | |
|  | ***Year range*** | 1998 – 2016 | | | | | |
|  | ***Quality assessment*** | QUADAS-2 (2 studies with high risk of bias) | | | | | |
|  | ***Special remark*** |  | | | | | |
| **Diagnostic accuracy** | ***Measure*** | Sensitivity [%] (95% CI):  97.0 (96.0; 98.0) | | | Specificity [%] (95% CI):  98.0 (97.0; 99.0) | | |
|  | ***GRADE*** | Low ⊖⊖⊕⊕ | | | Low ⊖⊖⊕⊕ | | |
| **Method. quality**  **(mod AMSTAR2)** | **Overall** | Critically low | | | | | |
| **Further investigations, sub- or sensitivity analyses** | **Index test** | | **Reference standard** | **Studies (Particip.)** | | **Sens. [%], 95% CI** | **Spec. [%], 95% CI** |
|  |  | |  |  | |  |  |
|  |  | |  |  | |  |  |
|  |  | |  |  | |  |  |
|  |  | |  |  | |  |  |
| **Conclusions of study authors** | *“In this systematic review and meta-analysis, we found prehospital lung ultrasound to have similar diagnostic test characteristics to emergency department performed lung ultrasound for the diagnosis of acute heart failure in dyspneic patients.”* | | | | | | |

###### Myocardial ischemia

| **Zarama (2024)**[77] – Myocardial ischemia | | | | | | | |
| --- | --- | --- | --- | --- | --- | --- | --- |
| **Clinical context** | ***Patient population*** | Adults with chest pain | | | | | |
|  | ***Clinical setting*** | Emergency department | | | | | |
|  | ***Prevalence [%] (CI)*** | 26.9 (15.2; 53.2) | | | | | |
|  | ***Noteworthy incl. crit.*** | Prospective cohort, cross-sectional, case–control studies and randomized controlled trials of interventions that included data on diagnostic accuracy. Adults with chest pain or chest pain equivalents of any duration presenting on the emergency department. | | | | | |
|  | ***Noteworthy excl. crit.*** | Case reports/series, animal studies, and retrospective studies were excluded. Trauma, postcardiac arrest, and mechanically ventilated patients were excluded as well as prehospital, primary/ambulatory care, hospital wards, and intensive/coronary care unit settings. Studies using only speckle-tracking and strain echocardiography as well as studies with ultrasounds performed by radiologists, nurses, or conducted inside a radiology suite were excluded. | | | | | |
| **Index test(s)** | Cardiac ultrasound (TTE, Limited TTE, POCUS) | | | | | | |
| **Reference standard(s)** | Chart review, final diagnosis, coronary angiography | | | | | | |
| **DTA-SR characteristics** | ***No. of studies*** | 6 | | | | | |
|  | ***No. of participants*** | 1790 | | | | | |
|  | ***Year range*** |  | | | | | |
|  | ***Quality assessment*** | QUADAS-2 (4 high risk of bias studies) | | | | | |
|  | ***Special remark*** | - | | | | | |
| **Diagnostic accuracy** | ***Measure*** | Sensitivity [%] (95% CI):  83.1 (60.2; 94.1) | | | Specificity [%] (95% CI):  89.2 (73.4; 96.1) | | |
|  | ***GRADE*** | Low ⊕⊖⊕⊖ | | | High ⊕⊕⊕⊕ | | |
| **Method. quality**  **(mod AMSTAR2)** | **Overall** | Moderate | | | | | |
| **Further investigations, sub- or sensitivity analyses** | **Index test** | | **Reference standard** | **Studies (Particip.)** | | **Sens. [%], 95% CI** | **Spec. [%], 95% CI** |
|  | Cardiac ultrasound (all studies) | | Chart review, final diagnosis, coronary angiography | 29 (5043) | | 79 (69; 87) | 87 (80; 92) |
|  | Cardiac ultrasound (by cardiologists) | | Chart review, final diagnosis, coronary angiography | 6 (727) | | 90 (74; 97) | 88 (72; 96) |
|  | TTE | | Chart review, final diagnosis, coronary angiography | 23 (3193) | | 79 (68; 87) | 88 (80; 93) |
|  | POCUS | | Chart review, final diagnosis, coronary angiography | 5 (1824) | | 76 (48; 92) | 85 (60; 95) |
|  | Cardiac ultrasound (all studies) | | Chart review | 11 (2968) | | 66 (48; 80) | 93 (87; 96) |
|  | Cardiac ultrasound (all studies) | | Coronary angiography | 7 (948) | | 87 (73; 94) | 89 (76; 95) |
| **Conclusions of study authors** | *“Cardiac ultrasound may have a potential role in the diagnostic pathway of myocardial ischemia in the ED; however, a pooled accuracy must be interpreted cautiously given substantial heterogeneity and that important patient and test characteristics affect its diagnostic performance.”* | | | | | | |

###### Shock related

| **Stickles (2019)**[78] – Shock etiology | | | | | | | |
| --- | --- | --- | --- | --- | --- | --- | --- |
| **Clinical context** | ***Patient population*** | Adult patients with acute undifferentiated shock | | | | | |
|  | ***Clinical setting*** | Emergency department | | | | | |
|  | ***Prevalence [%] (CI)*** | Not documented | | | | | |
|  | ***Noteworthy incl. crit.*** | RUSH exam or identical protocol | | | | | |
|  | ***Noteworthy excl. crit.*** | - | | | | | |
| **Index test(s)** | RUSH protocol or identical POCUS protocol; cardiogenic | | | | | | |
| **Reference standard(s)** | Chart review, expert consensus | | | | | | |
| **DTA-SR characteristics** | ***No. of studies*** | 4 | | | | | |
|  | ***No. of participants*** | 357 | | | | | |
|  | ***Year range*** | 2015 - 2018 | | | | | |
|  | ***Quality assessment*** | QUADAS-2 | | | | | |
|  | ***Special remark*** | - | | | | | |
| **Diagnostic accuracy** | ***Measure*** | Sensitivity [%] (95% CI):  83.0 (71.0; 92.0) | | | Specificity [%] (95% CI):  97.0 (95.0; 99.0) | | |
|  | ***GRADE*** | Low ⊕⊖⊕⊖ | | | High ⊕⊕⊕⊕ | | |
| **Method. quality**  **(mod AMSTAR2)** | **Overall** | Moderate | | | | | |
| **Further investigations, sub- or sensitivity analyses** | **Index test** | | **Reference standard** | **Studies (Particip.)** | | **Sens. [%], 95% CI** | **Spec. [%], 95% CI** |
|  | RUSH hypovolemic | | Chart review, expert consensus | 4 (357) | | 81 (73; 88) | 91 (87; 94) |
|  | RUSH distributive | | Chart review, expert consensus | 4 (357) | | 93 (68; 100) | 98 (96; 99) |
|  | RUSH mixed | | Chart review, expert consensus | 3 (332) | | 64 (56; 72) | 95 (91; 98) |
|  | RUSH obstructive | | Chart review, expert consensus | 3 (239) | | 75 (53;90) | 80 (73;85) |
| **Conclusions of study authors** | *“Our review suggests that the RUSH exam performs better when used to confirm suspected causes of shock, rather than to definitively exclude specific etiologies. The LR- values of the RUSH exam by subtype suggest that it most accurately rules out obstructive shock.”* | | | | | | |

| **Yoshida (2023)**[79] – Shock etiology | | | | | | | |
| --- | --- | --- | --- | --- | --- | --- | --- |
| **Clinical context** | ***Patient population*** | Adults with undifferentiated shock | | | | | |
|  | ***Clinical setting*** | Emergency department | | | | | |
|  | ***Prevalence [%] (CI)*** | Not documented | | | | | |
|  | ***Noteworthy incl. crit.*** | Undifferentiated shock | | | | | |
|  | ***Noteworthy excl. crit.*** | Diagnostic case-control studies | | | | | |
| **Index test(s)** | POCUS (TTE), cardiogenic shock | | | | | | |
| **Reference standard(s)** | Chart review | | | | | | |
| **DTA-SR characteristics** | ***No. of studies*** | 7 | | | | | |
|  | ***No. of participants*** | 743 | | | | | |
|  | ***Year range*** | 2015 - 2021 | | | | | |
|  | ***Quality assessment*** | QUADAS-2, overall low risk of bias | | | | | |
|  | ***Special remark*** |  | | | | | |
| **Diagnostic accuracy** | ***Measure*** | Sensitivity [%] (95% CI):  75.0 (49.0; 90.0) | | | Specificity [%] (95% CI):  94.0 (88.0; 97.0) | | |
|  | ***GRADE*** | Moderate ⊕⊕⊖⊕ | | | High ⊕⊕⊕⊕ | | |
| **Method. quality**  **(mod AMSTAR2)** | **Overall** | High | | | | | |
| **Further investigations, sub- or sensitivity analyses** | **Index test** | | **Reference standard** | **Studies (Particip.)** | | **Sens. [%], 95% CI** | **Spec. [%], 95% CI** |
|  | POCUS, obstructive shock | | Chart review | 7 (725) | | 83 (65; 92) | 97 (87; 99) |
|  | POCUS, hypovolemic shock | | Chart review | 7 (602) | | 91 (84; 95) | 92 (85; 96) |
|  | POCUS, distributive shock | | Chart review | 6 (509) | | 78 (69; 84) | 98 (95; 99) |
|  |  | |  |  | |  |  |
| **Conclusions of study authors** | *“In this study, the identification of the etiology of shock by POCUS was characterized by high sensitivity and a positive likelihood ratio, especially for obstructive shock. Hence, these findings should be considered in future diagnostic protocols for shock using POCUS. However, since this study only examined the diagnostic aspect, further interventional studies are necessary to assess the true impact of POCUS on shock patients.”* | | | | | | |

| **Basmaji (2025)**[80] – Shock etiology | | | | | | | |
| --- | --- | --- | --- | --- | --- | --- | --- |
| **Clinical context** | ***Patient population*** | Adult patients with hypotension and/or clinical manifestations of shock | | | | | |
|  | ***Clinical setting*** | Not documented | | | | | |
|  | ***Prevalence [%] (CI)*** | Not documented | | | | | |
|  | ***Noteworthy incl. crit.*** | Fulltext manuscripts or abstracts | | | | | |
|  | ***Noteworthy excl. crit.*** | Postcardiac surgical patients | | | | | |
| **Index test(s)** | RUSH protocol or identical POCUS protocol; mixed | | | | | | |
| **Reference standard(s)** | Chart review, expert consensus | | | | | | |
| **DTA-SR characteristics** | ***No. of studies*** | 8 | | | | | |
|  | ***No. of participants*** | 929 | | | | | |
|  | ***Year range*** | 2015 - 2022 | | | | | |
|  | ***Quality assessment*** | QUADAS-2 | | | | | |
|  | ***Special remark*** |  | | | | | |
| **Diagnostic accuracy** | ***Measure*** | Sensitivity [%] (95% CI):  85.0 (77.0; 91.0) | | | Specificity [%] (95% CI):  98.0 (91.0; 100) | | |
|  | ***GRADE*** | Low ⊝⊝⊕⊝ | | | Very low ⊝⊝⊕⊝ | | |
| **Method. quality**  **(mod AMSTAR2)** | **Overall** | Low | | | | | |
| **Further investigations, sub- or sensitivity analyses** | **Index test** | | **Reference standard** | **Studies (Particip.)** | | **Sens. [%], 95% CI** | **Spec. [%], 95% CI** |
|  | RUSH hypovolemic | | Chart review, expert consensus | 16 (1397) | | 90 (81; 95) | 95 (90, 97) |
|  | RUSH distributive | | Chart review, expert consensus | 15 (1327) | | 78 (69; 85) | 97 (94; 99) |
|  | RUSH obstructive | | Chart review, expert consensus | 13 (1132) | | 94 (85; 97) | 99 (98; 100) |
|  | RUSH cardiogenic | | Chart review, expert consensus | 14 (1217) | | 95 (84; 98) | 98 (97; 99) |
| **Conclusions of study authors** | *“On the basis of very low to moderate quality evidence, POCUS may perform better at ruling in shock subtypes and specific shock etiologies than ruling them out. Point-of-care ultrasound is a promising tool for the diagnosis of shock.”* | | | | | | |

| **Osawa (2025)**[81] – Cardiogenic shock | | | | | | | |
| --- | --- | --- | --- | --- | --- | --- | --- |
| **Clinical context** | ***Patient population*** | Adults with undifferentiated shock | | | | | |
|  | ***Clinical setting*** | Emergency department | | | | | |
|  | ***Prevalence [%] (CI)*** | Not documented | | | | | |
|  | ***Noteworthy incl. crit.*** | - | | | | | |
|  | ***Noteworthy excl. crit.*** | - | | | | | |
| **Index test(s)** | POCUS | | | | | | |
| **Reference standard(s)** | Final diagnosis | | | | | | |
| **DTA-SR characteristics** | ***No. of studies*** | 8 | | | | | |
|  | ***No. of participants*** | 783 | | | | | |
|  | ***Year range*** | 2015 - 2022 | | | | | |
|  | ***Quality assessment*** | QUADAS-2 (mostly low risk of bias studies) | | | | | |
|  | ***Special remark*** | Rating of evidence according to GRADE, heterogeneity noted as I2=0% | | | | | |
| **Diagnostic accuracy** | ***Measure*** | Sensitivity [%] (95% CI):  86.0 (72.0; 94.0) | | | Specificity [%] (95% CI):  96.0 (94.0; 97.0) | | |
|  | ***GRADE*** | Moderate ⊖⊕⊕⊕ | | | Moderate ⊖⊕⊕⊕ | | |
| **Method. quality**  **(mod AMSTAR2)** | **Overall** | Moderate | | | | | |
| **Further investigations, sub- or sensitivity analyses** | **Index test** | | **Reference standard** | **Studies (Particip.)** | | **Sens. [%], 95% CI** | **Spec. [%], 95% CI** |
|  |  | |  |  | |  |  |
|  |  | |  |  | |  |  |
|  |  | |  |  | |  |  |
|  |  | |  |  | |  |  |
| **Conclusions of study authors** | *“The diagnostic accuracy of POCUS for patients with cardiogenic shock was reasonable, especially as a rule-in test.”* | | | | | | |

| **Osawa (2025)**[81] – Obstructive shock | | | | | | | |
| --- | --- | --- | --- | --- | --- | --- | --- |
| **Clinical context** | ***Patient population*** | Adults with undifferentiated shock | | | | | |
|  | ***Clinical setting*** | Emergency department | | | | | |
|  | ***Prevalence [%] (CI)*** | Not documented | | | | | |
|  | ***Noteworthy incl. crit.*** | - | | | | | |
|  | ***Noteworthy excl. crit.*** | - | | | | | |
| **Index test(s)** | POCUS | | | | | | |
| **Reference standard(s)** | Final diagnosis | | | | | | |
| **DTA-SR characteristics** | ***No. of studies*** | 8 | | | | | |
|  | ***No. of participants*** | 863 | | | | | |
|  | ***Year range*** | 2015 - 2022 | | | | | |
|  | ***Quality assessment*** | QUADAS-2 (only low risk of bias studies) | | | | | |
|  | ***Special remark*** | Rating of evidence according to GRADE, heterogeneity noted as I2=0% | | | | | |
| **Diagnostic accuracy** | ***Measure*** | Sensitivity [%] (95% CI):  78.0 (63.0; 88.0) | | | Specificity [%] (95% CI):  98.0 (94.0; 99.0) | | |
|  | ***GRADE*** | Low ⊖⊕⊕⊖ | | | Moderate ⊖⊕⊕⊕ | | |
| **Method. quality**  **(mod AMSTAR2)** | **Overall** | Moderate | | | | | |
| **Further investigations, sub- or sensitivity analyses** | **Index test** | | **Reference standard** | **Studies (Particip.)** | | **Sens. [%], 95% CI** | **Spec. [%], 95% CI** |
|  |  | |  |  | |  |  |
|  |  | |  |  | |  |  |
|  |  | |  |  | |  |  |
|  |  | |  |  | |  |  |
| **Conclusions of study authors** | *“The diagnostic accuracy of POCUS for patients with cardiogenic shock was reasonable, especially as a rule-in test.”* | | | | | | |

###### Venous thromboembolism

| **Burnside (2008)**[82] – Deep vein thrombosis | | | | | | | |
| --- | --- | --- | --- | --- | --- | --- | --- |
| **Clinical context** | ***Patient population*** | Adults and children | | | | | |
|  | ***Clinical setting*** | Predominantly outpatients (>50%) | | | | | |
|  | ***Prevalence [%] (CI)*** | 14.1 (12.0; 16.5) | | | | | |
|  | ***Noteworthy incl. crit.*** | Original research of emergency department patients with signs/symptoms of DVT | | | | | |
|  | ***Noteworthy excl. crit.*** | Asymptomatic patients (Surveillance) | | | | | |
| **Index test(s)** | Ultrasound of one or both legs by emergency physician | | | | | | |
| **Reference standard(s)** | Ultrasound by radiology or vascular department | | | | | | |
| **DTA-SR characteristics** | ***No. of studies*** | 6 | | | | | |
|  | ***No. of participants*** | 936 | | | | | |
|  | ***Year range*** | 2001 - 2007 | | | | | |
|  | ***Quality assessment*** | QUADAS (Two studies not adequately blinded) | | | | | |
|  | ***Special remark*** |  | | | | | |
| **Diagnostic accuracy** | ***Measure*** | Sensitivity [%] (95% CI):  95.0 (87.0; 99.0) | | | Specificity [%] (95% CI):  96.0 (87.0; 99.0) | | |
|  | ***GRADE*** | Low ⊕⊖⊕⊖ | | | Low ⊕⊖⊕⊖ | | |
| **Method. quality**  **(mod AMSTAR2)** | **Overall** | Moderate | | | | | |
| **Further investigations, sub- or sensitivity analyses** | **Index test** | | **Reference standard** | **Studies (Particip.)** | | **Sens. [%], 95% CI** | **Spec. [%], 95% CI** |
|  |  | |  |  | |  |  |
|  |  | |  |  | |  |  |
|  |  | |  |  | |  |  |
|  |  | |  |  | |  |  |
| **Conclusions of study authors** | “*Our data show promise for ultrasound by emergency physician as a method to evaluate for deep vein thrombosis. However, the limitations in the literature comprising this systematic review point toward the need for further research before widespread adoption.”* | | | | | | |

| **Goodacre (2006)**[83] – Deep vein thrombosis | | | | | | | |
| --- | --- | --- | --- | --- | --- | --- | --- |
| **Clinical context** | ***Patient population*** | Adults and children | | | | | |
|  | ***Clinical setting*** | Outpatient, inpatient and emergency department | | | | | |
|  | ***Prevalence [%] (CI)*** | 48 | | | | | |
|  | ***Noteworthy incl. crit.*** | - | | | | | |
|  | ***Noteworthy excl. crit.*** | - | | | | | |
| **Index test(s)** | Ultrasound (compression ultrasonography, color Doppler, continuous wave doppler, or a combination thereof) | | | | | | |
| **Reference standard(s)** | Venography | | | | | | |
| **DTA-SR characteristics** | ***No. of studies*** | 100 | | | | | |
|  | ***No. of participants*** | Not documented | | | | | |
|  | ***Year range*** | Not documented | | | | | |
|  | ***Quality assessment*** | 3 criteria: 1. reference standard (fulfilled in all studies: venography) 2. Ultrasound blinded to venography (fulfilled in 62 cohorts) 3. Venography blinded to ultrasound result (fulfilled in 56 cohorts) | | | | | |
|  | ***Special remark*** |  | | | | | |
| **Diagnostic accuracy** | ***Measure*** | Sensitivity [%] (95% CI):  89.7 (88.8, 90.5) | | | Specificity [%] (95% CI):  93.8 (93.1; 94.4) | | |
|  | ***GRADE*** | Moderate ⊕⊕⊖⊕ | | | Moderate ⊕⊕⊖⊕ | | |
| **Method. quality**  **(mod AMSTAR2)** | **Overall** | Moderate | | | | | |
| **Further investigations, sub- or sensitivity analyses** | **Index test** | | **Reference standard** | **Studies (Particip.)** | | **Sens. [%], 95% CI** | **Spec. [%], 95% CI** |
|  | Compression only | | Same as above | 22 | | 90 (88; 92) | 98 (97; 98) |
|  | Proximal DVT only | | Same as above |  | | 94 (93; 95) | 94 (93; 94) |
|  | Distal DVT only | | Same as above |  | | 64 (60; 67) | 94 (93; 94) |
|  | Duplex | | Same as above | 28 | | 92 (98; 94) | 94 (93; 95) |
| **Conclusions of study authors** | *“In patients with clinically suspected DVT, ultrasound can rule out proximal but not distal DVT. In asymptomatic patients neither proximal nor distal DVT can be ruled out by ultrasound. In both patient groups specificity is good, but not perfect, so use of this technique in low-risk patients carries the risk of producing significant numbers of false-positive results. Optimal sensitivity (particularly for distal DVT) is achieved by the combined use of compression ultrasound and color Doppler, while optimal specificity is achieved by using compression ultrasound alone.”* | | | | | | |

| **Gottlieb (1999)**[84] – Deep vein thrombosis | | | | | | | |
| --- | --- | --- | --- | --- | --- | --- | --- |
| **Clinical context** | ***Patient population*** | Adults, children | | | | | |
|  | ***Clinical setting*** | Not described | | | | | |
|  | ***Prevalence [%] (CI)*** | 23.1 (17.9; 29.2) | | | | | |
|  | ***Noteworthy incl. crit.*** | Signs and Symptoms of DVT, n=mind. 5, only calf | | | | | |
|  | ***Noteworthy excl. crit.*** | Non-blinded | | | | | |
| **Index test(s)** | Ultrasound of all deep veins in the calf | | | | | | |
| **Reference standard(s)** | Contrast venography | | | | | | |
| **DTA-SR characteristics** | ***No. of studies*** | 5 | | | | | |
|  | ***No. of participants*** | 212 extremities | | | | | |
|  | ***Year range*** | 1990 - 1996 | | | | | |
|  | ***Quality assessment*** | No quality evaluation | | | | | |
|  | ***Special remark*** |  | | | | | |
| **Diagnostic accuracy** | ***Measure*** | Sensitivity [%] (95% CI):  92.5 (81.8; 97.9) | | | Specificity [%] (95% CI):  98.7 (95.5; 99.9) | | |
|  | ***GRADE*** | Very low ⊖⊕⊖⊖ | | | Very low ⊖⊕⊖⊖ | | |
| **Method. quality**  **(mod AMSTAR2)** | **Overall** | Critically low | | | | | |
| **Further investigations, sub- or sensitivity analyses** | **Index test** | | **Reference standard** | **Studies (Particip.)** | | **Sens. [%], 95% CI** | **Spec. [%], 95% CI** |
|  |  | |  |  | |  |  |
|  |  | |  |  | |  |  |
|  |  | |  |  | |  |  |
|  |  | |  |  | |  |  |
| **Conclusions of study authors** | “*In summary, sonography is highly accurate in detecting DVT in the calf in patients with lower extremity symptoms or physical signs suggestive of DVT. However, indeterminate examinations occur relatively frequently, with a wide variation in reported rates. Each ultrasound laboratory should review its own rate of indeterminate studies in the calf.”* | | | | | | |

| **Hercz (2024)**[85] – Deep vein thrombosis | | | | | | | |
| --- | --- | --- | --- | --- | --- | --- | --- |
| **Clinical context** | ***Patient population*** | Adults | | | | | |
|  | ***Clinical setting*** | Emergency department | | | | | |
|  | ***Prevalence [%] (CI)*** | Not documented | | | | | |
|  | ***Noteworthy incl. crit.*** | Cross-sectional and longitudinal (cohort and randomized controlled trials) | | | | | |
|  | ***Noteworthy excl. crit.*** | Review articles, editorials or letters, expert opinions, comments, and animal experiments | | | | | |
| **Index test(s)** | Ultrasound by emergency physician (50% 2-Point, 50% 3-Point) | | | | | | |
| **Reference standard(s)** | RADUS or angiology | | | | | | |
| **DTA-SR characteristics** | ***No. of studies*** | 15 | | | | | |
|  | ***No. of participants*** | 2311 | | | | | |
|  | ***Year range*** | 2012-2020 | | | | | |
|  | ***Quality assessment*** | QUADAS-2 (40% of studies were considered high or unclear risk of bias of patient selection due to the use of convenience, non-consecutive sampling.) | | | | | |
|  | ***Special remark*** |  | | | | | |
| **Diagnostic accuracy** | ***Measure*** | Sensitivity [%] (95% CI):  90.0 (82.0; 95.0) | | | Specificity [%] (95% CI):  95.0 (91.0; 97.0) | | |
|  | ***GRADE*** | Low ⊕⊖⊕⊖ | | | Moderate ⊕⊖⊕⊕ | | |
| **Method. quality**  **(mod AMSTAR2)** | **Overall** | Moderate | | | | | |
| **Further investigations, sub- or sensitivity analyses** | **Index test** | | **Reference standard** | **Studies (Particip.)** | | **Sens. [%], 95% CI** | **Spec. [%], 95% CI** |
|  | Exclusion of 6 outliers | | RADUS or angiology | 9 | | 89 (85; 92) | 96 (93; 97) |
|  | Trainee | | RADUS or angiology | NA | | 77 | 87 |
|  | Attending | | RADUS or angiology | NA | | 93 | 97 |
|  |  | |  |  | |  |  |
| **Conclusions of study authors** | *“This meta-analysis of studies reported since 2012 demonstrated excellent performance of EM specialist performed three-point point-of-care ultrasound for the diagnosis of deep vein thrombosis. Both the pooled sensitivity and specificity were 95%. We recommend that POCUS-trained attending EPs perform a three-point examination in the emergency departement to effectively and accurately diagnose DVT”* | | | | | | |

| **Lee (2019)**[86] –Deep vein thrombosis | | | | | | | |
| --- | --- | --- | --- | --- | --- | --- | --- |
| **Clinical context** | ***Patient population*** | Not documented | | | | | |
|  | ***Clinical setting*** | Emergency department | | | | | |
|  | ***Prevalence [%] (CI)*** | Not documented | | | | | |
|  | ***Noteworthy incl. crit.*** | Patients with suspected deep vein thrombosis | | | | | |
|  | ***Noteworthy excl. crit.*** | Duplex as index test | | | | | |
| **Index test(s)** | 2-Point POCUS by emergency physician | | | | | | |
| **Reference standard(s)** | Contrast venography, follow-up or additional ultrasound performed by radiologist | | | | | | |
| **DTA-SR characteristics** | ***No. of studies*** | 9 | | | | | |
|  | ***No. of participants*** | 1337 | | | | | |
|  | ***Year range*** | 2001 - 2018 | | | | | |
|  | ***Quality assessment*** | QUADAS-2 (no studies were considered to be seriously flawed according to the QUADAS-2 tool. All the studies satisfied ≥4 of the 7 item) | | | | | |
|  | ***Special remark*** |  | | | | | |
| **Diagnostic accuracy** | ***Measure*** | Sensitivity [%] (95% CI):  91.0 (68.0, 98.0) | | | Specificity [%] (95% CI):  98.0 (96.0; 99.0) | | |
|  | ***GRADE*** | Low ⊕⊖⊕⊖ | | | Low ⊕⊖⊕⊖ | | |
| **Method. quality**  **(mod AMSTAR2)** | **Overall** | Moderate | | | | | |
| **Further investigations, sub- or sensitivity analyses** | **Index test** | | **Reference standard** | **Studies (Particip.)** | | **Sens. [%], 95% CI** | **Spec. [%], 95% CI** |
|  | 3-Point POCUS by emergency physician | | Same as above | 8 | | 90 (83-95) | 95 (83;99) |
|  |  | |  |  | |  |  |
|  |  | |  |  | |  |  |
|  |  | |  |  | |  |  |
| **Conclusions of study authors** | “*In summary, the present meta-analysis revealed that both 2point and 3-point POCUS showed excellent performance for the diagnosis of DVT. The effect of the isolated superficial femoral vein thrombosis in 2-point POCUS was minimal. We recommend that POCUS trained attending emergency physicians perform the initial 2point POCUS to effectively and accurately diagnose DVT.”* | | | | | | |

| **Pomero (2013)**[87] – Deep vein thrombosis | | | | | | | |
| --- | --- | --- | --- | --- | --- | --- | --- |
| **Clinical context** | ***Patient population*** | Not documented | | | | | |
|  | ***Clinical setting*** | Emergency department | | | | | |
|  | ***Prevalence [%] (CI)*** | 23.1 (21.3; 24.7) | | | | | |
|  | ***Noteworthy incl. crit.*** | Any article evaluating the diagnostic performance of emergency physician performed ultrasound in the diagnosis of DVT | | | | | |
|  | ***Noteworthy excl. crit.*** | - | | | | | |
| **Index test(s)** | Ultrasound by emergency physician: Duplex (1 study), proximal venous ultrasound (1 study), whole leg ultrasound (1 study) | | | | | | |
| **Reference standard(s)** | RADUS, angiology lab or angiography | | | | | | |
| **DTA-SR characteristics** | ***No. of studies*** | 16 | | | | | |
|  | ***No. of participants*** | 2379 | | | | | |
|  | ***Year range*** | 1990 - 2011 | | | | | |
|  | ***Quality assessment*** | QUADAS-2 (There were 11 of high quality) | | | | | |
|  | ***Special remark*** |  | | | | | |
| **Diagnostic accuracy** | ***Measure*** | Sensitivity [%] (95% CI):  96.1 (90.6; 98.5) | | | Specificity [%] (95% CI):  96.8 (94.6, 98.1) | | |
|  | ***GRADE*** | High ⊕⊕⊕⊕ | | | High ⊕⊕⊕⊕ | | |
| **Method. quality**  **(mod AMSTAR2)** | **Overall** | Moderate | | | | | |
| **Further investigations, sub- or sensitivity analyses** | **Index test** | | **Reference standard** | **Studies (Particip.)** | | **Sens. [%], 95% CI** | **Spec. [%], 95% CI** |
|  | Proximal B-mode compression ultrasound | | RADUS, angiology | 13 (1806) | | 96 (90; 99) | 97 (95; 98) |
|  | Only high quality studies | | RADUS, angiology |  | | 98 (90; 100) | 97 (94, 98) |
|  | Only peer reviewed | | RADUS, angiology |  | | 97 (90; 99) | 97 (94; 99) |
|  |  | |  |  | |  |  |
| **Conclusions of study authors** | *“Our findings suggest that* emergency physician performed ultrasound *may be useful in the management of patients with suspected DVT. Future prospective studies are warranted to confirm our preliminary findings and to evaluate this technique together with algorithms incorporating pre-test probability assessment with a sensitive D-dimer assay.”* | | | | | | |

| **Zaki (2024)**[88] – Deep vein thrombosis | | | | | | | |
| --- | --- | --- | --- | --- | --- | --- | --- |
| **Clinical context** | ***Patient population*** | Adults | | | | | |
|  | ***Clinical setting*** | Emergency department | | | | | |
|  | ***Prevalence [%] (CI)*** | Not documented | | | | | |
|  | ***Noteworthy incl. crit.*** | Diagnosis carried out by emergency physician or in the emergency department | | | | | |
|  | ***Noteworthy excl. crit.*** | Radiology department ultrasound as index test, case reports, sample size less than 50 participants | | | | | |
| **Index test(s)** | 2-point POCUS | | | | | | |
| **Reference standard(s)** | RADUS, venography | | | | | | |
| **DTA-SR characteristics** | ***No. of studies*** | 12 | | | | | |
|  | ***No. of participants*** | 1689 | | | | | |
|  | ***Year range*** | Not documented | | | | | |
|  | ***Quality assessment*** | QUADAS-2 (overall low risk of bias) | | | | | |
|  | ***Special remark*** | Reduced time to diagnosis compared to RADUS, Metaregression shows that level of training was NOT a significant source of heterogeneity | | | | | |
| **Diagnostic accuracy** | ***Measure*** | Sensitivity [%] (95% CI):  92.0 (88.0; 97.0) | | | Specificity [%] (95% CI):  97.0 (95.0; 99.0) | | |
|  | ***GRADE*** | Moderate ⊕⊖⊕⊕ | | | Moderate ⊕⊖⊕⊕ | | |
| **Method. quality**  **(mod AMSTAR2)** | **Overall** | Moderate | | | | | |
| **Further investigations, sub- or sensitivity analyses** | **Index test** | | **Reference standard** | **Studies (Particip.)** | | **Sens. [%], 95% CI** | **Spec. [%], 95% CI** |
|  | 3-point POCUS | | RADUS, venography | 12 (1662) | | 89 (83; 95) | 93 (90; 96) |
|  | Complete compression ultrasound/whole leg ultrasound | | RADUS | 2 (511) | | 100 (98; 100) | 97 (92; 100) |
|  |  | |  |  | |  |  |
|  |  | |  |  | |  |  |
| **Conclusions of study authors** | *“The current meta-analysis has shown that the 2-point, 3-point, complete compression ultrasound, and wholeleg duplex POCUS protocols are excellent in diagnosing DVT in the emergency department. Combining the high diagnostic performance with the fact that POCUS significantly reduces the time from triage to DVT diagnosis, we can recommend that POCUS be utilized as the first-line imaging tool for diagnosing DVT in the emergency department.”* | | | | | | |

| **Asmara (2022)**[73] –Pulmonary embolism | | | | | | | |
| --- | --- | --- | --- | --- | --- | --- | --- |
| **Clinical context** | ***Patient population*** | Adults | | | | | |
|  | ***Clinical setting*** | Intensive care unit, emergency department | | | | | |
|  | ***Prevalence [%] (CI)*** | Not documented | | | | | |
|  | ***Noteworthy incl. crit.*** | Cross-sectional design, comparing the diagnostic ability of the BLUE protocol with a gold standard, patients with acute respiratory failure | | | | | |
|  | ***Noteworthy excl. crit.*** | - | | | | | |
| **Index test(s)** | LUS (BLUE protocol) | | | | | | |
| **Reference standard(s)** | Echocardiography, radiography or clinical parameters | | | | | | |
| **DTA-SR characteristics** | ***No. of studies*** | 2 | | | | | |
|  | ***No. of participants*** | 643 | | | | | |
|  | ***Year range*** | 2008 - 2019 | | | | | |
|  | ***Quality assessment*** | Quadas-2 (No studies with high risk of bias) | | | | | |
|  | ***Special remark*** |  | | | | | |
| **Diagnostic accuracy** | ***Measure*** | Sensitivity [%] (95% CI):  65.5 (31.3; 99.6) | | | Specificity [%] (95% CI):  99 (98.3; 100) | | |
|  | ***GRADE*** | Low ⊕⊖⊕⊖ | | | High ⊕⊕⊕⊕ | | |
| **Method. quality**  **(mod AMSTAR2)** | **Overall** | Moderate | | | | | |
| **Further investigations, sub- or sensitivity analyses** | **Index test** | | **Reference standard** | **Studies (Particip.)** | | **Sens. [%], 95% CI** | **Spec. [%], 95% CI** |
|  |  | |  |  | |  |  |
|  |  | |  |  | |  |  |
|  |  | |  |  | |  |  |
|  |  | |  |  | |  |  |
| **Conclusions of study authors** | *“The BLUE protocol has high sensitivity and specificity in diagnosing pneumonia and pulmonary edema. These high diagnostic accuracy values came from a good quality study based on the GRADE approach. The BLUE protocol has high specificity in diagnose pneumothorax and pulmonary embolism but with varying sensitivity. This accuracy assessment comes from a poor-quality study based on the GRADE approach.”* | | | | | | |

| **Cao (2022)**[89] – Pulmonary embolism | | | | | | | |
| --- | --- | --- | --- | --- | --- | --- | --- |
| **Clinical context** | ***Patient population*** | Not documented | | | | | |
|  | ***Clinical setting*** | Emergency department and ICU | | | | | |
|  | ***Prevalence [%] (CI)*** | Not documented | | | | | |
|  | ***Noteworthy incl. crit.*** | Cardiopulmonary Ultrasound for pulmonary embolism among breathless adult patients | | | | | |
|  | ***Noteworthy excl. crit.*** | - | | | | | |
| **Index test(s)** | Cardiopulmonary ultrasound | | | | | | |
| **Reference standard(s)** | CT or clinically derived diagnostic criterion/expert opinion audit | | | | | | |
| **DTA-SR characteristics** | ***No. of studies*** | 10 | | | | | |
|  | ***No. of participants*** | 426 | | | | | |
|  | ***Year range*** | 2013 - 2021 | | | | | |
|  | ***Quality assessment*** | QUADAS-2 (All included studies rated low or unclear) | | | | | |
|  | ***Special remark*** |  | | | | | |
| **Diagnostic accuracy** | ***Measure*** | Sensitivity [%] (95% CI):  77.0 (50.0; 92.0) | | | Specificity [%] (95% CI):  99.0 (97.0;100) | | |
|  | ***GRADE*** | Very low ⊖⊖⊕⊖ | | | Low ⊖⊖⊕⊕ | | |
| **Method. quality**  **(mod AMSTAR2)** | **Overall** | Low | | | | | |
| **Further investigations, sub- or sensitivity analyses** | **Index test** | | **Reference standard** | **Studies (Particip.)** | | **Sens. [%], 95% CI** | **Spec. [%], 95% CI** |
|  | Prospective observational studies | | CT, clinic | NA | | 68 (42; 86) | 99 (96; 100) |
|  | Expert opinion only as reference | | CT, clinic | NA | | 84 (NA) | 99 (97; 100) |
|  |  | |  |  | |  |  |
|  |  | |  |  | |  |  |
| **Conclusions of study authors** | *“Our review provides important information and valuable implications for clinical evaluation of pulmonary embolism and informs that combined cardiac and lung ultrasound has the ability to be used as an adjunct to CT especially in resource constrained settings. The use of this imaging modality can aid the patients in having a significant decline in the time and money spent during these diagnostic procedures. Another important implication is that the early assessment of suspected patients can help in engaging the diagnosed patients early in the effective therapeutic management.”* | | | | | | |

| **Du (2024)**[90] – Pulmonary embolism | | | | | | | |
| --- | --- | --- | --- | --- | --- | --- | --- |
| **Clinical context** | ***Patient population*** | Adults clinically suspected of having pulmonary embolism | | | | | |
|  | ***Clinical setting*** | Various settings | | | | | |
|  | ***Prevalence [%] (CI)*** | Not documented | | | | | |
|  | ***Noteworthy incl. crit.*** | Patients with suspected pulmonary embolism, lung ultrasound as index test, comparison with CT or comprehensive diagnostic standard | | | | | |
|  | ***Noteworthy excl. crit.*** | - | | | | | |
| **Index test(s)** | Lung ultrasound (various, mostly including typical pleural-based/subpleural hypoechoic lesions) | | | | | | |
| **Reference standard(s)** | CT, MRI, angiography, V/Q scan | | | | | | |
| **DTA-SR characteristics** | ***No. of studies*** | 18 | | | | | |
|  | ***No. of participants*** | 2158 | | | | | |
|  | ***Year range*** | 1990 - 2022 | | | | | |
|  | ***Quality assessment*** | QUADAS-2 (12 studies with high risk of bias) | | | | | |
|  | ***Special remark*** |  | | | | | |
| **Diagnostic accuracy** | ***Measure*** | Sensitivity [%] (95% CI):  80.0 (71.0; 86.0) | | | Specificity [%] (95% CI):  87.0 (81.0; 92.0) | | |
|  | ***GRADE*** | Very low ⊕⊖⊖⊖ | | | Very low ⊕⊖⊖⊖ | | |
| **Method. quality**  **(mod AMSTAR2)** | **Overall** | Moderate | | | | | |
| **Further investigations, sub- or sensitivity analyses** | **Index test** | | **Reference standard** | **Studies (Particip.)** | | **Sens. [%], 95% CI** | **Spec. [%], 95% CI** |
|  |  | |  |  | |  |  |
|  |  | |  |  | |  |  |
|  |  | |  |  | |  |  |
|  |  | |  |  | |  |  |
| **Conclusions of study authors** | *“In conclusion, lung ultrasound is a rapid, radiation-free and bedside feasible diagnostic tool that shows promising advantages in the early diagnosis of pulmonary embolism. Although lung ultrasound is expected to be a useful complementary diagnostic method to CTA, methodological quality issues in the included studies and the generalisability limitations of patient populations restrict the broad application of this conclusion. Further research, particularly exploring data in specific populations, is still needed.”* | | | | | | |

| **Falster (2022)**[91] – Pulmonary embolism | | | | | | | |
| --- | --- | --- | --- | --- | --- | --- | --- |
| **Clinical context** | ***Patient population*** | Not documented | | | | | |
|  | ***Clinical setting*** | Not documented | | | | | |
|  | ***Prevalence [%] (CI)*** | 32.0 (26.8; 37.7) | | | | | |
|  | ***Noteworthy incl. crit.*** | Studies reporting diagnostic accuracy of deep venous, lung, or cardiac ultrasound, or any combination thereof, in patients with suspected pulmonary embolism. | | | | | |
|  | ***Noteworthy excl. crit.*** | Control group consisted of healthy volunteers or if transesophageal or endobronchial ultrasound | | | | | |
| **Index test(s)** | Multi organ ultrasound (“right ventricular dilatation or DVT”) | | | | | | |
| **Reference standard(s)** | Multimodal (CT, MRI, angiography, V/Q scan, autopsy) | | | | | | |
| **DTA-SR characteristics** | ***No. of studies*** | 3 | | | | | |
|  | ***No. of participants*** | 281 | | | | | |
|  | ***Year range*** | Not reported explicitly, search until 04/2020 | | | | | |
|  | ***Quality assessment*** | QUADAS-2 (All studies but one showed risk of bias in a least one domain, most often because the time interval between ultrasound examination and reference test was not reported or >24 hours (51 studies, 72.8%), or because of inappropriate or unclear exclusion criteria) | | | | | |
|  | ***Special remark*** |  | | | | | |
| **Diagnostic accuracy** | ***Measure*** | Sensitivity [%] (95% CI):  91.8 (84.5; 96.4) | | | Specificity [%] (95% CI):  63.9 (56.5; 70.9) | | |
|  | ***GRADE*** | Low ⊕⊖⊕⊖ | | | Low ⊕⊖⊕⊖ | | |
| **Method. quality**  **(mod AMSTAR2)** | **Overall** | Moderate | | | | | |
| **Further investigations, sub- or sensitivity analyses** | **Index test** | | **Reference standard** | **Studies (Particip.)** | | **Sens. [%], 95% CI** | **Spec. [%], 95% CI** |
|  | Cardiac ultrasound (dilation of the right ventricular, all signs) | | Multimodal | 35 | | 63 (55; 71) | 87 (83; 91) |
|  | Lung ultrasound | | Multimodal | 19 | | 81 (73; 88) | 87 (81; 92) |
|  | Deep venous ultrasound (compression) | | Multimodal | 22 | | 44 (36; 51) | 97 (95; 98) |
|  | Deep venous ultrasound (compression), unselected patients | | Multimodal | 10 | | 48 (40; 57) | 96 (5; 98) |
| **Conclusions of study authors** | *“We encourage the use of multiorgan POCUS, encompassing deep venous, lung and cardiac ultrasound, in patients where standard clinical evaluation does not rule out suspected pulmonary embolism and further radiation imaging is required. In this context, several ultrasound signs are highly predictive of pulmonary embolism and detection of a DVT or a right ventricular thrombus allows for diagnosis without further testing and adds valuable information regarding prognosis. Furthermore, multiorgan POCUS devoid of DVT, hypoechoic subpleural lesions or signs of right ventricular pressure overload has a high negative predictive value and findings consistent with an alternative explanation for the patient’s symptoms practically excludes pulmonary pressure suspicion.”* | | | | | | |

| **Fields (2017)**[92] – Pulmonary embolism | | | | | | | |
| --- | --- | --- | --- | --- | --- | --- | --- |
| **Clinical context** | ***Patient population*** | Adults and chields | | | | | |
|  | ***Clinical setting*** | Emergency department and inpatient setting | | | | | |
|  | ***Prevalence [%] (CI)*** | 24.7 (22.8; 26.6) | | | | | |
|  | ***Noteworthy incl. crit.*** | English language, research of any design that reported quantitative data, focus on diagnosis of acute pulmonary embolism using echocardiography, and reporting of data that allowed for calculation of both sensitivity and specificity. | | | | | |
|  | ***Noteworthy excl. crit.*** | Exclusion criteria included articles that were anecdotal or had no data; on >50% pregnant patients; prognostic studies; single case reports or had a sample size less than 10; studies that used healthy controls; or experimental studies using animals, animal tissue, or cadavers. | | | | | |
| **Index test(s)** | TTE | | | | | | |
| **Reference standard(s)** | CT, angiography, V/Q scan (+- confirmatory test for interm. Result), surgery, autopsy | | | | | | |
| **DTA-SR characteristics** | ***No. of studies*** | 16 | | | | | |
|  | ***No. of participants*** | 1986 | | | | | |
|  | ***Year range*** | 2003-2016 | | | | | |
|  | ***Quality assessment*** | QUADAS-2; Four studies had a high or unclear risk of bias in all four categories. | | | | | |
|  | ***Special remark*** |  | | | | | |
| **Diagnostic accuracy** | ***Measure*** | Sensitivity [%] (95% CI):  53.0 (45.0; 61.0) | | | Specificity [%] (95% CI):  83.0 (74.0; 90.0) | | |
|  | ***GRADE*** | Very low ⊖⊖⊕⊖ | | | Very low ⊖⊖⊕⊖ | | |
| **Method. quality**  **(mod AMSTAR2)** | **Overall** | Low | | | | | |
| **Further investigations, sub- or sensitivity analyses** | **Index test** | | **Reference standard** | **Studies (Particip.)** | | **Sens. [%], 95% CI** | **Spec. [%], 95% CI** |
|  | Right heart strain in emergency studies | | CT, angiography, V/Q scan | 6 | | 55 (32;76) | 87 (82;90) |
|  | Right heart strain for physician performed studies | | CT, angiography, V/Q scan | 5 | | 61 (32;84) | 88 (83;92) |
|  | Right heart strain in POCUS studies | | CT, angiography, V/Q scan | 3 | | 42 (28;57) | 87 (87;92) |
|  |  | |  |  | |  |  |
| **Conclusions of study authors** | *“Studies have consistently shown a high specificity for transthoracic echocardiography in diagnosing pulmonary embolism in patients with an adequate suspicion on the part of the clinician, making it useful as a rule-in test. This may be especially useful at the bedside in critical care settings for patients unable to get other confirmatory studies, especially the most critical patients.”* | | | | | | |

| **Jiang (2015)**[93] – Pulmonary embolism | | | | | | | |
| --- | --- | --- | --- | --- | --- | --- | --- |
| **Clinical context** | ***Patient population*** | Adults | | | | | |
|  | ***Clinical setting*** | Not documented | | | | | |
|  | ***Prevalence [%] (CI)*** | Not documented | | | | | |
|  | ***Noteworthy incl. crit.*** | Adult patients with acute suspected pulmonary embolism | | | | | |
|  | ***Noteworthy excl. crit.*** | - | | | | | |
| **Index test(s)** | LUS | | | | | | |
| **Reference standard(s)** | CT, V/Q scan or combination | | | | | | |
| **DTA-SR characteristics** | ***No. of studies*** | 13 | | | | | |
|  | ***No. of participants*** | 1356 | | | | | |
|  | ***Year range*** | 1991 - 2013 | | | | | |
|  | ***Quality assessment*** | QUADAS-2 (8 studies with more than 1 criterium for high risk) | | | | | |
|  | ***Special remark*** |  | | | | | |
| **Diagnostic accuracy** | ***Measure*** | Sensitivity [%] (95% CI):  85.0 (78.0; 90.0) | | | Specificity [%] (95% CI):  83.0 (73.0; 90.0) | | |
|  | ***GRADE*** | Very low ⊖⊖⊕⊖ | | | Very low ⊖⊖⊕⊖ | | |
| **Method. quality**  **(mod AMSTAR2)** | **Overall** | Low | | | | | |
| **Further investigations, sub- or sensitivity analyses** | **Index test** | | **Reference standard** | **Studies (Particip.)** | | **Sens. [%], 95% CI** | **Spec. [%], 95% CI** |
|  | Studies published pre 2000 | | Same as above | 5 | | 94 (89; 96) | 74 (61;85) |
|  | Studies published past 2000 | | Same as above | 8 | | 77 (70;83) | 88 (78;94) |
|  |  | |  |  | |  |  |
|  |  | |  |  | |  |  |
| **Conclusions of study authors** | “Transthoracic lung ultrasonography is helpful in diagnosing pulmonary embolism. Although, the positive transthoracic lung ultrasonography results significantly increase the post-test probability of pulmonary embolism and the negative transthoracic lung ultrasonography results significantly decrease the post-test probability of pulmonary embolism, which may change some patients’ diagnostic processes, it is inappropriate to generally use transthoracic ultrasonography in diagnosing pulmonary embolism due to the lack of appropriate tools used to calculate the pre-test probability of pulmonary embolism.” | | | | | | |

| **Kagima (2020)**[94] – Pulmonary embolism | | | | | | | |
| --- | --- | --- | --- | --- | --- | --- | --- |
| **Clinical context** | ***Patient population*** | Adults | | | | | |
|  | ***Clinical setting*** | Emergency department | | | | | |
|  | ***Prevalence [%] (CI)*** | 22 (CI nod documented) | | | | | |
|  | ***Noteworthy incl. crit.*** | Prospective and retrospective observational studies and clinical trials that recruited acutely breathless adults | | | | | |
|  | ***Noteworthy excl. crit.*** | Non-English papers, unpublished research, conference communications, paediatric studies and studies on animals | | | | | |
| **Index test(s)** | Multi organ POCUS (Cardiac and lung ultrasound) | | | | | | |
| **Reference standard(s)** | CT and clinically derived diagnosis | | | | | | |
| **DTA-SR characteristics** | ***No. of studies*** | 2 | | | | | |
|  | ***No. of participants*** | 453 | | | | | |
|  | ***Year range*** | 2013 - 2017 | | | | | |
|  | ***Quality assessment*** | QUADAS-2 (“the quality of all studies was generally high, had low risk of bias, and satisfied the majority of the risk of bias and applicability”) | | | | | |
|  | ***Special remark*** |  | | | | | |
| **Diagnostic accuracy** | ***Measure*** | Sensitivity [%] (95% CI):  91.0 (84.0; 95.0) | | | Specificity [%] (95% CI):  81.0 (77.0; 85.0) | | |
|  | ***GRADE*** | Low ⊕⊖⊕⊖ | | | Moderate ⊕⊖⊕⊕ | | |
| **Method. quality**  **(mod AMSTAR2)** | **Overall** | Moderate | | | | | |
| **Further investigations, sub- or sensitivity analyses** | **Index test** | | **Reference standard** | **Studies (Particip.)** | | **Sens. [%], 95% CI** | **Spec. [%], 95% CI** |
|  | Multi-Organ POCUS (Same as above) | | Clinically derived Diagnosis as reference standard | 5 | | 52 (43; 61) | 99 (99; 99) |
|  |  | |  |  | |  |  |
|  |  | |  |  | |  |  |
|  |  | |  |  | |  |  |
| **Conclusions of study authors** | *“Our findings suggest that cardiopulmonary ultrasonography has potential to be used in pulmonary embolism diagnostics when CT is unsuitable or unavailable. Cardiopulmonary ultrasound may be highly relevant in low resource settings as it is cheaper and is less human resource dependent compared to CT. Based on the current evidence, a global harmonization of ultrasound diagnostic criteria for pulmonary embolism is required, potentially within worldwide consensus guidelines that will allow specifications of essential practice standards for low resource settings.”* | | | | | | |

| **Melo (2025)**[95] – Pulmonary embolism | | | | | | | |
| --- | --- | --- | --- | --- | --- | --- | --- |
| **Clinical context** | ***Patient population*** | Adults | | | | | |
|  | ***Clinical setting*** | Emergency department, intensive care unit | | | | | |
|  | ***Prevalence [%] (CI)*** | Not documented | | | | | |
|  | ***Noteworthy incl. crit.*** | Patients ≥18 with suspected pulmonary embolism, reference standard CT or V/Q scan | | | | | |
|  | ***Noteworthy excl. crit.*** | Studies including pediatric populations, case reports, non-english articles, preclinical articles | | | | | |
| **Index test(s)** | Multi-organ ultrasound (combined lung, cardiac and venous POCUS) | | | | | | |
| **Reference standard(s)** | CTPA and/or V/Q scan | | | | | | |
| **DTA-SR characteristics** | ***No. of studies*** | 4 | | | | | |
|  | ***No. of participants*** | 594 | | | | | |
|  | ***Year range*** | 2014 - 2023 | | | | | |
|  | ***Quality assessment*** | QUADAS 2 (1 study with high risk of bias) | | | | | |
|  | ***Special remark*** | One study focused exclusively on patients with COVID-19. This was the only high risk of bias study | | | | | |
| **Diagnostic accuracy** | ***Measure*** | Sensitivity [%] (95% CI):  90.0 (85.0; 94.0) | | | Specificity [%] (95% CI):  69.0 (42.0; 87.0) | | |
|  | ***GRADE*** | High ⊕⊕⊕⊕ | | | Moderate ⊕⊕⊕⊖ | | |
| **Method. quality**  **(mod AMSTAR2)** | **Overall** | Moderate | | | | | |
| **Further investigations, sub- or sensitivity analyses** | **Index test** | | **Reference standard** | **Studies (Particip.)** | | **Sens. [%], 95% CI** | **Spec. [%], 95% CI** |
|  | Multi-organ ultrasound, only low risk of bias studies | | CTPA and/or V/Q scan | 3 (553) | | 91 (85; 94) | 84 (73; 90) |
|  |  | |  |  | |  |  |
|  |  | |  |  | |  |  |
|  |  | |  |  | |  |  |
| **Conclusions of study authors** | “In our study, multi-organ POCUS has demonstrated high accuracy for ruling in or out pulmonary embolism in critically ill patients, offering a valuable, cost-effective alternative to traditional imaging modalities, especially in resource-limited environments. Further research is needed to validated these findings across different patient populations. “ | | | | | | |

| **Niemann (2009)**[96] – Pulmonary embolism | | | | | | | |
| --- | --- | --- | --- | --- | --- | --- | --- |
| **Clinical context** | ***Patient population*** | Not documented | | | | | |
|  | ***Clinical setting*** | Not documented | | | | | |
|  | ***Prevalence [%] (CI)*** | 48.1 (44.3; 51.9) | | | | | |
|  | ***Noteworthy incl. crit.*** | Articles about the diagnostic performance of transthoracic ultrasound for patients with suspected pulmonary embolism | | | | | |
|  | ***Noteworthy excl. crit.*** | Review articles, case reports, letters and comments, published before 1990 | | | | | |
| **Index test(s)** | Lung ultrasound | | | | | | |
| **Reference standard(s)** | CT, MRI or CT/US/D-Dimers | | | | | | |
| **DTA-SR characteristics** | ***No. of studies*** | 5 | | | | | |
|  | ***No. of participants*** | 652 | | | | | |
|  | ***Year range*** | 1999 - 2005 | | | | | |
|  | ***Quality assessment*** | STARD | | | | | |
|  | ***Special remark*** |  | | | | | |
| **Diagnostic accuracy** | ***Measure*** | Sensitivity [%] (95% CI):  80.0 (72.0; 86.9) | | | Specificity [%] (95% CI):  93.0 (90.1; 95.8) | | |
|  | ***GRADE*** | Low ⊕⊖⊕⊖ | | | High ⊕⊕⊕⊕ | | |
| **Method. quality**  **(mod AMSTAR2)** | **Overall** | Moderate | | | | | |
| **Further investigations, sub- or sensitivity analyses** | **Index test** | | **Reference standard** | **Studies (Particip.)** | | **Sens. [%], 95% CI** | **Spec. [%], 95% CI** |
|  |  | |  |  | |  |  |
|  |  | |  |  | |  |  |
|  |  | |  |  | |  |  |
|  |  | |  |  | |  |  |
| **Conclusions of study authors** | *“Transthoracic ultrasound for the detection of pulmonary embolism should not be seen as the first-choice imaging modality in all consecutive patients, since other modalities such as CT are more sensitive and specific. However, there are special clinical settings, e. g. pregnant patients, when transthoracic ultrasound can be used to alter the pre-test probability of pulmonary embolism. As well described in the literature, different levels of probability are associated with different algorithms for further investigation and treatment. However, CT and V/Q scanning remain the imaging modalities of choice in the case of suspected pulmonary embolism”* | | | | | | |

| **Squizzato (2013)**[97] – Pulmonary embolism | | | | | | | |
| --- | --- | --- | --- | --- | --- | --- | --- |
| **Clinical context** | ***Patient population*** | Adults and children | | | | | |
|  | ***Clinical setting*** | Not documented | | | | | |
|  | ***Prevalence [%] (CI)*** | 61.0 (57.7; 64.1) | | | | | |
|  | ***Noteworthy incl. crit.*** | Suspected pulmonary embolism, no language restrictions | | | | | |
|  | ***Noteworthy excl. crit.*** | - | | | | | |
| **Index test(s)** | LUS looking for triangular/wedge or rounded, hypo-echogenic, homogenous, pleura-based lesions (5 studies did not report clear criteria) | | | | | | |
| **Reference standard(s)** | "Composite reference" / any imaging test ("seven studies used proper reference") | | | | | | |
| **DTA-SR characteristics** | ***No. of studies*** | 10 | | | | | |
|  | ***No. of participants*** | 887 | | | | | |
|  | ***Year range*** | 1990 - 2010 | | | | | |
|  | ***Quality assessment*** | QUADAS-2 (All studies had high or medium risk bias) | | | | | |
|  | ***Special remark*** |  | | | | | |
| **Diagnostic accuracy** | ***Measure*** | Sensitivity [%] (95% CI):  87.0 (79.0; 92.0) | | | Specificity [%] (95% CI):  81.0 (71.0; 89.0) | | |
|  | ***GRADE*** | Low ⊖⊖⊕⊕ | | | Low ⊖⊖⊕⊕ | | |
| **Method. quality**  **(mod AMSTAR2)** | **Overall** | Low | | | | | |
| **Further investigations, sub- or sensitivity analyses** | **Index test** | | **Reference standard** | **Studies (Particip.)** | | **Sens. [%], 95% CI** | **Spec. [%], 95% CI** |
|  | Low quality according to QUADAS-2 | | Composite reference | NA | | 91 (81; 96) | 86 (73; 93) |
|  | High quality studies | | Composite reference | NA | | 78 (68; 85) | 75 (65; 84) |
|  | LUS | | CT as single or main reference | 5 | | 80 (76; 88) | 87 (77; 93) |
|  |  | |  |  | |  |  |
| **Conclusions of study authors** | *“The results of our meta-analysis suggest that lung ultrasound may be a useful diagnostic tool in the management of patients with suspected pulmonary embolism when CT is contraindicated or not available. However, a high potential for bias in the included studies limits any definitive conclusion”* | | | | | | |

| **Zaki (2025)**[98] – Pulmonary embolism | | | | | | | |
| --- | --- | --- | --- | --- | --- | --- | --- |
| **Clinical context** | ***Patient population*** | Adults | | | | | |
|  | ***Clinical setting*** | Emergency department | | | | | |
|  | ***Prevalence [%] (CI)*** | Not documented | | | | | |
|  | ***Noteworthy incl. crit.*** | Deep venous, lung, cardiac or multiorgan ultrasound, any acceptable reference test | | | | | |
|  | ***Noteworthy excl. crit.*** | Case reports, control groups with healthy volunteers, outpatient or intensive care unit | | | | | |
| **Index test(s)** | Multiorgan ultrasound, overall | | | | | | |
| **Reference standard(s)** | CT, CTPA, MRA, autopsy | | | | | | |
| **DTA-SR characteristics** | ***No. of studies*** | 10 | | | | | |
|  | ***No. of participants*** | Not documented | | | | | |
|  | ***Year range*** | 1998 - 2023 | | | | | |
|  | ***Quality assessment*** | QUADAS-2; mostly moderate to high risk of bias | | | | | |
|  | ***Special remark*** |  | | | | | |
| **Diagnostic accuracy** | ***Measure*** | Sensitivity [%] (95% CI):  79.0 (64.0; 94.0) | | | Specificity [%] (95% CI):  78.0 (68.0; 90.0) | | |
|  | ***GRADE*** | Low ⊕⊖⊕⊖ | | | Low ⊕⊖⊕⊖ | | |
| **Method. quality**  **(mod AMSTAR2)** | **Overall** | Moderate | | | | | |
| **Further investigations, sub- or sensitivity analyses** | **Index test** | | **Reference standard** | **Studies (Particip.)** | | **Sens. [%], 95% CI** | **Spec. [%], 95% CI** |
|  | TTE, overall | | CT, CTPA, MRA, autopsy | 32 (NA) | | 39 (29; 48) | 94 (92; 96) |
|  | Lung ultrasound, overall | | CT, CTPA, MRA, autopsy | 9 (NA) | | 47 (26; 68) | 88 (81; 94) |
|  | Deep venous ultrasound, overall | | CT, CTPA, MRA, autopsy | 7 (NA) | | 56 (46; 65) | 96 (93; 99) |
|  |  | |  |  | |  |  |
| **Conclusions of study authors** | *“In summary, cardiac, lung, and deep venous ultrasounds have consistently shown high specificity, making them valuable diagnostic tools for ruling in pulmonary embolism at the patient’s bedside. Moreover, evidence has shown that multiorgan POCUS has a high diagnostic accuracy, making it a valuable tool for confirming or dismissing pulmonary embolism in emergency department. Therefore, multiorgan POCUS can be used as an alternative to CTPA in medical institutions where CTPA is not available or in cases where CTPA is not feasible.”* | | | | | | |

##### Ear, Nose, and Throat

| **Kim (2023)**[99] – Peritonsillar abscess | | | | | | | |
| --- | --- | --- | --- | --- | --- | --- | --- |
| **Clinical context** | ***Patient population*** | Adults and children | | | | | |
|  | ***Clinical setting*** | Emergency department, Ear – Nose- Throat clinic | | | | | |
|  | ***Prevalence [%] (CI)*** | Not documented | | | | | |
|  | ***Noteworthy incl. crit.*** | Reference standard CT or aspiration/drainage of pus | | | | | |
|  | ***Noteworthy excl. crit.*** | Case series with 10 or fewer patients | | | | | |
| **Index test(s)** | POCUS, transcervical and intraoral | | | | | | |
| **Reference standard(s)** | Drainage or CT | | | | | | |
| **DTA-SR characteristics** | ***No. of studies*** | 6 | | | | | |
|  | ***No. of participants*** | 452 | | | | | |
|  | ***Year range*** | 2005 - 2021 | | | | | |
|  | ***Quality assessment*** | QUADAS-2; all studies with high or unclear risk of bias | | | | | |
|  | ***Special remark*** | Overall small population except one study; no significant difference in sensitivity analysis | | | | | |
| **Diagnostic accuracy** | ***Measure*** | Sensitivity [%] (95% CI):  74.0 (67.0; 80.0) | | | Specificity [%] (95% CI):  79.0 (61.0; 90.0) | | |
|  | ***GRADE*** | Low ⊕⊖⊕⊖ | | | Low ⊕⊖⊕⊖ | | |
| **Method. quality**  **(mod AMSTAR2)** | **Overall** | Moderate | | | | | |
| **Further investigations, sub- or sensitivity analyses** | **Index test** | | **Reference standard** | **Studies (Particip.)** | | **Sens. [%], 95% CI** | **Spec. [%], 95% CI** |
|  | RADUS | | Drainage or CT | 12 (372) | | 89 (84; 92) | 71 (63; 79) |
|  | Intraoral any sonographer | | Drainage or CT | 8 (NA) | | 91 (82: 95) | 75 (63; 84) |
|  | Transcervical any sonographer | | Drainage or CT | 12 (NA) | | 80 (67; 89) | 81 (66; 91) |
|  |  | |  |  | |  |  |
| **Conclusions of study authors** | *“Ultrasound is an acceptable alternative to computed tomography and interventional aspiration or drainage for establishing the diagnosis of peritonsillar abscess. It has high sensitivity for ruling out peritonsillar abscess, but it demonstrates only moderate specificity for ruling in the diagnosis.”* | | | | | | |

##### Gastrointestinal

###### Appendicitis

| **Arruzza (2022)**[100] – Appendicitis | | | | | | | |
| --- | --- | --- | --- | --- | --- | --- | --- |
| **Clinical context** | ***Patient population*** | Adults | | | | | |
|  | ***Clinical setting*** | Mostly emergency department | | | | | |
|  | ***Prevalence [%] (CI)*** | Not documented | | | | | |
|  | ***Noteworthy incl. crit.*** | Quantitative studies that examined the outcomes of sensitivity and specificity in the diagnosis of adult appendicitis. Comparative accuracy studies, multiple test studies. | | | | | |
|  | ***Noteworthy excl. crit.*** | Pediatric or pregnant patients | | | | | |
| **Index test(s)** | Not documented | | | | | | |
| **Reference standard(s)** | Pathology, surgery, follow-up | | | | | | |
| **DTA-SR characteristics** | ***No. of studies*** | 16 | | | | | |
|  | ***No. of participants*** | 2549 | | | | | |
|  | ***Year range*** | 2007 - 2020 | | | | | |
|  | ***Quality assessment*** | QUADAS-2 | | | | | |
|  | ***Special remark*** |  | | | | | |
| **Diagnostic accuracy** | ***Measure*** | Sensitivity [%] (95% CI):  82.1 (73.8; 88.2) | | | Specificity [%] (95% CI):  85.9 (72.7.0;93.3) | | |
|  | ***GRADE*** | Low ⊕⊖⊕⊖ | | | Low ⊕⊖⊕⊖ | | |
| **Method. quality**  **(mod AMSTAR2)** | **Overall** | Moderate | | | | | |
| **Further investigations, sub- or sensitivity analyses** | **Index test** | | **Reference standard** | **Studies (Particip.)** | | **Sens. [%], 95% CI** | **Spec. [%], 95% CI** |
|  |  | |  |  | |  |  |
|  |  | |  |  | |  |  |
|  |  | |  |  | |  |  |
|  |  | |  |  | |  |  |
| **Conclusions of study authors** | *“CT is superior in diagnostic accuracy compared to ultrasound. Ultrasound findings may be overestimated if equivocal findings are excluded from analysis”* | | | | | | |

| **Cho (2023)**[101] – Appendicitis | | | | | | | |
| --- | --- | --- | --- | --- | --- | --- | --- |
| **Clinical context** | ***Patient population*** | Adults and children | | | | | |
|  | ***Clinical setting*** | Emergency department | | | | | |
|  | ***Prevalence [%] (CI)*** | Not documented | | | | | |
|  | ***Noteworthy incl. crit.*** | - | | | | | |
|  | ***Noteworthy excl. crit.*** | Only articles published in English. | | | | | |
| **Index test(s)** | POCUS | | | | | | |
| **Reference standard(s)** | Pathology, surgery, follow-up | | | | | | |
| **DTA-SR characteristics** | ***No. of studies*** | 21 | | | | | |
|  | ***No. of participants*** | 2735 | | | | | |
|  | ***Year range*** | 1998 - 2021 | | | | | |
|  | ***Quality assessment*** | QUADAS-2 | | | | | |
|  | ***Special remark*** |  | | | | | |
| **Diagnostic accuracy** | ***Measure*** | Sensitivity [%] (95% CI):  81.0 (78.0; 83.0) | | | Specificity [%] (95% CI):  87.0 (85.0; 88.0) | | |
|  | ***GRADE*** | Moderate ⊕⊖⊕⊕ | | | Moderate ⊕⊖⊕⊕ | | |
| **Method. quality**  **(mod AMSTAR2)** | **Overall** | Moderate | | | | | |
| **Further investigations, sub- or sensitivity analyses** | **Index test** | | **Reference standard** | **Studies (Particip.)** | | **Sens. [%], 95% CI** | **Spec. [%], 95% CI** |
|  | Ultrasound by emergency physician | | Histopathological findings, clinical follow up | 14 | | 86 (84; 88) | 84 (82; 86) |
|  |  | |  |  | |  |  |
|  |  | |  |  | |  |  |
|  |  | |  |  | |  |  |
| **Conclusions of study authors** | *“This systematic review and meta-analysis of studies on the use of ultrasound for the diagnosis of acute appendicitis in the emergency department showed that ultrasound has high overall sensitivity and specificity for the diagnosis of acute appendicitis; however, high heterogeneity among the included studies and potential publication bias were observed. Therefore, additional tests are necessary when the POCUS result is positive, to establish a final diagnosis. It is also important to acknowledge the limitations and constraints of POCUS examination, and to interpret test results carefully and professionally.”* | | | | | | |

| **Fields (2017)**[102] – Appendicitis | | | | | | | |
| --- | --- | --- | --- | --- | --- | --- | --- |
| **Clinical context** | ***Patient population*** | Adults and children | | | | | |
|  | ***Clinical setting*** | Emergency department | | | | | |
|  | ***Prevalence [%] (CI)*** | 29.8 (28.7; 30.9) | | | | | |
|  | ***Noteworthy incl. crit.*** | Reporting of quantitative data, nonradiologist physicians | | | | | |
|  | ***Noteworthy excl. crit.*** | Ultrasound performed by radiologist, not POCUS, sample <10, not in English, reviews or editorials with no data, >50% pregnant patients, experimental studies using animals | | | | | |
| **Index test(s)** | B-mode ultrasound | | | | | | |
| **Reference standard(s)** | Pathology, surgery, follow-up, CT, MRI | | | | | | |
| **DTA-SR characteristics** | ***No. of studies*** | 21 | | | | | |
|  | ***No. of participants*** | 6636 | | | | | |
|  | ***Year range*** | 1996 - 2015 | | | | | |
|  | ***Quality assessment*** | QUADAS-2 | | | | | |
|  | ***Special remark*** |  | | | | | |
| **Diagnostic accuracy** | ***Measure*** | Sensitivity [%] (95% CI):  91.0 (83.0;96.0) | | | Specificity [%] (95% CI):  97.0 (91.0;99.0) | | |
|  | ***GRADE*** | Low ⊕⊖⊕⊖ | | | Moderate ⊕⊖⊕⊕ | | |
| **Method. quality**  **(mod AMSTAR2)** | **Overall** | Moderate | | | | | |
| **Further investigations, sub- or sensitivity analyses** | **Index test** | | **Reference standard** | **Studies (Particip.)** | | **Sens. [%], 95% CI** | **Spec. [%], 95% CI** |
|  | Ultrasound performed by emergency physicians | | Pathology, surgery, follow-up, CT, MRI | NA | | 80 (76; 83) | 92 (90; 94) |
|  | No differentiation between adult and pediatric patients, emergency physicians | | Pathology, surgery, follow-up, CT, MRI | NA | | 76 (47; 92) | 89 (67; 97) |
|  | No differentiation between adult and pediatric patients, non-emergency physicians | | Pathology, surgery, follow-up, CT, MRI | NA | | 93 (83; 97) | 99 (97; 99) |
|  | Pediatric patients, emergency physicians | | Pathology, surgery, follow-up, CT, MRI | NA | | 89 (47; 99) | 97 (84; 99) |
|  | Pediatric patients, non-emergency physicians | | Pathology, surgery, follow-up, CT, MRI | NA | | 96 (92; 98) | 92 (56; 99) |
| **Conclusions of study authors** | *“... it does suggest that point-of-care ultrasonography in the hands of a trained operator is an appropriate initial imaging modality for diagnosing appendicitis. Ultrasonography is not a sufficient standalone nor rule-out test, and those patients with high clinical suspicion of appendicitis and a negative or non-diagnostic ultrasonography warrant further evaluation.”* | | | | | | |

| **Fu (2021)**[103] – Appendicitis | | | | | | | |
| --- | --- | --- | --- | --- | --- | --- | --- |
| **Clinical context** | ***Patient population*** | Adults and children | | | | | |
|  | ***Clinical setting*** | Not documented | | | | | |
|  | ***Prevalence [%] (CI)*** | Not documented | | | | | |
|  | ***Noteworthy incl. crit.*** | Acute appendicitis in all age groups from 2010 to 2021 | | | | | |
|  | ***Noteworthy excl. crit.*** | Reference standard other than pathology | | | | | |
| **Index test(s)** | B-mode ultrasound | | | | | | |
| **Reference standard(s)** | Pathology, surgery, follow-up, CT | | | | | | |
| **DTA-SR characteristics** | ***No. of studies*** | 18 | | | | | |
|  | ***No. of participants*** | 4600 | | | | | |
|  | ***Year range*** | 2008 - 2022 | | | | | |
|  | ***Quality assessment*** | QUADAS-2 (all judged as low risk of bias)) | | | | | |
| **Diagnostic accuracy** | ***Measure*** | Sensitivity [%] (95% CI):  77.2 (75.4; 78.9) | | | Specificity [%] (95% CI):  80.0 (60.0; 92.0) | | |
|  | ***GRADE*** | Moderate ⊕⊖⊕⊕ | | | Low ⊕⊖⊕⊖ | | |
| **Method. quality**  **(mod AMSTAR2)** | **Overall** | Moderate | | | | | |
| **Further investigations, sub- or sensitivity analyses** | **Index test** | | **Reference standard** | **Studies (Particip.)** | | **Sens. [%], 95% CI** | **Spec. [%], 95% CI** |
|  |  | |  |  | |  |  |
|  |  | |  |  | |  |  |
|  |  | |  |  | |  |  |
|  |  | |  |  | |  |  |
|  |  | |  |  | |  |  |
| **Conclusions of study authors** | *“Abdominal ultrasound shows significant accuracy of diagnosis in patients*  *with suspected acute appendicitis.”* | | | | | | |

| **Giljaca (2017)**[104] – Appendicitis | | | | | | | |
| --- | --- | --- | --- | --- | --- | --- | --- |
| **Clinical context** | ***Patient population*** | Adults and children | | | | | |
|  | ***Clinical setting*** | Not documented | | | | | |
|  | ***Prevalence [%] (CI)*** | Not documented | | | | | |
|  | ***Noteworthy incl. crit.*** | - | | | | | |
|  | ***Noteworthy excl. crit.*** | Studies and case series with less than 10 patients, period between index test and reference standard was longer than one week, studies older than year 1994. | | | | | |
| **Index test(s)** | B-mode ultrasound | | | | | | |
| **Reference standard(s)** | Pathology, surgery | | | | | | |
| **DTA-SR characteristics** | ***No. of studies*** | 17 | | | | | |
|  | ***No. of participants*** | 2841 | | | | | |
|  | ***Year range*** | 1994 - 2014 | | | | | |
|  | ***Quality assessment*** | QUADAS-2 | | | | | |
|  | ***Special remark*** |  | | | | | |
| **Diagnostic accuracy** | ***Measure*** | Sensitivity [%] (95% CI):  69.0 (59.0; 78.0) | | | Specificity [%] (95% CI):  81.0 (73.0; 88.0) | | |
|  | ***GRADE*** | Very low ⊝⊝⊝⊕ | | | Very low ⊝⊝⊝⊕ | | |
| **Method. quality**  **(mod AMSTAR2)** | **Overall** | Low | | | | | |
| **Further investigations, sub- or sensitivity analyses** | **Index test** | | **Reference standard** | **Studies (Particip.)** | | **Sens. [%], 95% CI** | **Spec. [%], 95% CI** |
|  |  | |  |  | |  |  |
|  |  | |  |  | |  |  |
|  |  | |  |  | |  |  |
|  |  | |  |  | |  |  |
| **Conclusions of study authors** | *“Based on the results of our study, the sensitivity and specificity of ultrasound do not seem to exceed that of the combination of history, physical examination and laboratory tests previously published in the literature. It may be considered by clinicians that patients with equivocal signs of AA may not benefit from ultrasound examination, and other imaging modalities may be more beneficial.”* | | | | | | |

| **Kamal (2026**)[105] –Appendicitis | | | | | | | |
| --- | --- | --- | --- | --- | --- | --- | --- |
| **Clinical context** | ***Patient population*** | Adults and children | | | | | |
|  | ***Clinical setting*** | Emergency department | | | | | |
|  | ***Prevalence [%] (CI)*** | Not documented | | | | | |
|  | ***Noteworthy incl. crit.*** | -. | | | | | |
|  | ***Noteworthy excl. crit.*** | Studies focusing solely on pregnant women were excluded unless separate data for the general population were available or the study specifically aimed to validate POCUS in pregnancy, | | | | | |
| **Index test(s)** | POCUS performed at the bedside by emergency physicians, including residents or attendings. | | | | | | |
| **Reference standard(s)** | Pathology, surgery, follow-up, CT, MRI | | | | | | |
| **DTA-SR characteristics** | ***No. of studies*** | 15 | | | | | |
|  | ***No. of participants*** | 2021 | | | | | |
|  | ***Year range*** | 2000 - 2024 | | | | | |
|  | ***Quality assessment*** | QUADAS-2 (5 studies with high risk of bias) | | | | | |
|  | ***Special remark*** |  | | | | | |
| **Diagnostic accuracy** | ***Measure*** | Sensitivity [%] (95% CI):  78.0 (69.0; 85.0) | | | Specificity [%] (95% CI):  89.0 (83.0; 93.0) | | |
|  | ***GRADE*** | Low ⊕⊖⊕⊖ | | | Moderate ⊕⊖⊕⊕ | | |
| **Method. quality (mod AMSTAR2)** | **Overall** | High | | | | | |
| **Further investigations, sub- or sensitivity analyses** | **Index test** | | **Reference standard** | **Studies (Particip.)** | | **Sens. [%], 95% CI** | **Spec. [%], 95% CI** |
|  |  | |  |  | |  |  |
|  |  | |  |  | |  |  |
|  |  | |  |  | |  |  |
|  |  | |  |  | |  |  |
| **Conclusions of study authors** | *“POCUS performed by emergency physicians is a highly specific and moderately sensitive tool for diagnosing acute appendicitis. A positive scan reliably rules in the disease, potentially reducing the need for further imaging. However, a negative scan is insufficient to rule out appendicitis, requiring secondary evaluation.”* | | | | | | |

| **Lee (2019)**[106] – Appendicitis | | | | | | | |
| --- | --- | --- | --- | --- | --- | --- | --- |
| **Clinical context** | ***Patient population*** | Adults and children | | | | | |
|  | ***Clinical setting*** | Emergency department | | | | | |
|  | ***Prevalence [%] (CI)*** | Not documented | | | | | |
|  | ***Noteworthy incl. crit.*** | Right lower quadrant pain, sufficient information to reconstruct 2x2 contingency tables, original research articles | | | | | |
|  | ***Noteworthy excl. crit.*** | Case reports, case series, review articles, guidelines, consensus statements, letters, editorials, clinical trials, conference abstracts, studies that only used the radiologists final report | | | | | |
| **Index test(s)** | POCUS (cut-off 6 or 7 mm) by emergency physician | | | | | | |
| **Reference standard(s)** | Pathology, surgery, follow-up | | | | | | |
| **DTA-SR characteristics** | ***No. of studies*** | 17 | | | | | |
|  | ***No. of participants*** | 2385 | | | | | |
|  | ***Year range*** | 2000 - 2018 | | | | | |
|  | ***Quality assessment*** | QUADAS-2 (all studies high risk of bias) | | | | | |
|  | ***Special remark*** |  | | | | | |
| **Diagnostic accuracy** | ***Measure*** | Sensitivity [%] (95% CI):  84.0 (72.0; 92.0) | | | Specificity [%] (95% CI):  91.0 (85.0; 95.0) | | |
|  | ***GRADE*** | Low ⊕⊖⊕⊖ | | | Moderate ⊕⊖⊕⊕ | | |
| **Method. quality**  **(mod AMSTAR2)** | **Overall** | Moderate | | | | | |
| **Further investigations, sub- or sensitivity analyses** | **Index test** | | **Reference standard** | **Studies (Particip.)** | | **Sens. [%], 95% CI** | **Spec. [%], 95% CI** |
|  | POCUS for pediatric patients by emergency physician | | Histopathological findings | 7 | | 95 (75; 99) | 95 (85; 98) |
|  | POCUS by emergency physician | | RADUS | 5 | | 81 (61; 90) | 89 (77; 95) |
|  |  | |  |  | |  |  |
|  |  | |  |  | |  |  |
| **Conclusions of study authors** | *“In conclusion, the present meta-analysis revealed that emergency physician-POCUS had excellent performance for diagnosing acute appendicitis, which was similar to that of RADUS, and even better diagnostic performance for pediatric acute appendicitis. We recommend that the attending emergency physicians perform the initial POCUS using a diameter cut-off value of 7 mm to more accurately diagnose acute appendicitis.””* | | | | | | |

| **Li (2022)**[107] – Appendicitis | | | | | | | |
| --- | --- | --- | --- | --- | --- | --- | --- |
| **Clinical context** | ***Patient population*** | Pregnant women | | | | | |
|  | ***Clinical setting*** | Not documented (mostly radiology) | | | | | |
|  | ***Prevalence [%] (CI)*** | Not documented | | | | | |
|  | ***Noteworthy incl. crit.*** | Original research articles, retrospective studies, prospective studies | | | | | |
|  | ***Noteworthy excl. crit.*** | Reviews, case reports | | | | | |
| **Index test(s)** | Not documented | | | | | | |
| **Reference standard(s)** | Pathology, surgery | | | | | | |
| **DTA-SR characteristics** | ***No. of studies*** | 5 | | | | | |
|  | ***No. of participants*** | 521 | | | | | |
|  | ***Year range*** | 2015 - 2019 | | | | | |
|  | ***Quality assessment*** | QUADAS-2 (all with high risk of bias) | | | | | |
|  | ***Special remark*** |  | | | | | |
| **Diagnostic accuracy** | ***Measure*** | Sensitivity [%] (95% CI):  62.0 (43.0; 78.0) | | | Specificity [%] (95% CI):  91.0 (74.0; 97.0) | | |
|  | ***GRADE*** | Very low ⊖⊖⊖⊖ | | | Very low ⊖⊖⊖⊖ | | |
| **Method. quality**  **(mod AMSTAR2)** | **Overall** | Low | | | | | |
| **Further investigations, sub- or sensitivity analyses** | **Index test** | | **Reference standard** | **Studies (Particip.)** | | **Sens. [%], 95% CI** | **Spec. [%], 95% CI** |
|  |  | |  |  | |  |  |
|  |  | |  |  | |  |  |
|  |  | |  |  | |  |  |
|  |  | |  |  | |  |  |
| **Conclusions of study authors** | *“In summary, data of this meta-analysis revealed that ultrasound had a medium-level sensitivity and high specificity on the diagnosis of acute appendicitis in pregnant women.”* | | | | | | |

| **Moghadam (2022)**[108] – Appendicitis | | | | | | | |
| --- | --- | --- | --- | --- | --- | --- | --- |
| **Clinical context** | ***Patient population*** | Pregnant women | | | | | |
|  | ***Clinical setting*** | Not documented | | | | | |
|  | ***Prevalence [%] (CI)*** | 54.7 (50.0; 59.0) | | | | | |
|  | ***Noteworthy incl. crit.*** |  | | | | | |
|  | ***Noteworthy excl. crit.*** | Case series, case reports, letters, abstracts, editorials, reviews, meta-analyses, comments, guidelines | | | | | |
| **Index test(s)** | Not documented | | | | | | |
| **Reference standard(s)** | Pathology, surgery, CT | | | | | | |
| **DTA-SR characteristics** | ***No. of studies*** | 8 | | | | | |
|  | ***No. of participants*** | 1593 | | | | | |
|  | ***Year range*** | 2010 - 2020 | | | | | |
|  | ***Quality assessment*** | QUADAS-2 (all studies with low risk of bias) | | | | | |
|  | ***Special remark*** |  | | | | | |
| **Diagnostic accuracy** | ***Measure*** | Sensitivity [%] (95% CI):  77.6 (75.4; 79.6) | | | Specificity [%] (95% CI):  75.3 (73.1; 77.5) | | |
|  | ***GRADE*** | Very low ⊖⊖⊖⊕ | | | Very low ⊖⊖⊖⊕ | | |
| **Method. quality**  **(mod AMSTAR2)** | **Overall** | Low | | | | | |
| **Further investigations, sub- or sensitivity analyses** | **Index test** | | **Reference standard** | **Studies (Particip.)** | | **Sens. [%], 95% CI** | **Spec. [%], 95% CI** |
|  | In retrospective studies | |  |  | | 78 (74; 82) | 85 (81; 88) |
|  | India | |  |  | | 92 | 100 |
|  |  | |  |  | |  |  |
|  |  | |  |  | |  |  |
| **Conclusions of study authors** | *“In a conclusion, ultrasound evaluation has a low diagnostic accuracy for acute appendicitis in pregnant women especially during the third trimester. These patients should be diagnosed early and accurately to prevent both complications and unnecessary operations. According to the previous studies and results of our study, we recommended that alternative imaging should be considered mostly after negative or uncertain USG.”* | | | | | | |

| **Montero (2025)**[109] – Appendicitis | | | | | | | |
| --- | --- | --- | --- | --- | --- | --- | --- |
| **Clinical context** | ***Patient population*** | Adults | | | | | |
|  | ***Clinical setting*** | Radiologist performed | | | | | |
|  | ***Prevalence [%] (CI)*** | Not documented | | | | | |
|  | ***Noteworthy incl. crit.*** | We included prospective or retrospective observational clinical studies evaluating the diagnostic performance of any Doppler ultrasound modality [in the main analysis]. | | | | | |
|  | ***Noteworthy excl. crit.*** | Studies in languages other than English or Spanish, and those not involving surgical confirmation or populations outside the scope of interest (e.g., immunocompromised patients, abdominal malignancy, hematological disorders). | | | | | |
| **Index test(s)** | Color Doppler (secondary DTA Meta-analysis) | | | | | | |
| **Reference standard(s)** | Pathology | | | | | | |
| **DTA-SR characteristics** | ***No. of studies*** | 12 | | | | | |
|  | ***No. of participants*** | Not documented for this group. | | | | | |
|  | ***Year range*** | 1992 - 2019 | | | | | |
|  | ***Quality assessment*** | QUADAS-2 | | | | | |
|  | ***Special remark*** | Not enough Data for meta-analysis of Power Doppler modality and to differentiate complicated with uncomplicated appendicitis. | | | | | |
| **Diagnostic accuracy** | ***Measure*** | Sensitivity [%] (95% CI):  82.0 (70.0; 90.0) | | | Specificity [%] (95% CI):  97.0 (92.0; 99.0) | | |
|  | ***GRADE*** | Very low ⊕⊖⊖⊖ | | | Low ⊕⊖⊖⊕ | | |
| **Method. quality (mod AMSTAR2)** | **Overall** | Moderate | | | | | |
| **Further investigations, sub- or sensitivity analyses** | **Index test** | | **Reference standard** | **Studies (Particip.)** | | **Sens. [%], 95% CI** | **Spec. [%], 95% CI** |
|  | Spectral Doppler | | Pathology | 5 (NA) | | 81 (68; 89) | 88 (73; 95) |
|  |  | |  |  | |  |  |
|  |  | |  |  | |  |  |
|  |  | |  |  | |  |  |
| **Conclusions of study authors** | *“Based on the current evidence, Doppler ultrasound is not a reliable tool for differentiating between complicated and non-complicated appendicitis, and its use for this purpose cannot be recommended. When the cecal appendix is adequately visualized, however, Doppler ultrasound —particularly Color Doppler and Spectral Doppler—demonstrates high diagnostic accuracy, with Color Doppler offering excellent specificity as a dependable rule-in test.”* | | | | | | |

| **Popat (2024)**[110] – Appendicitis | | | | | | | |
| --- | --- | --- | --- | --- | --- | --- | --- |
| **Clinical context** | ***Patient population*** | Adults and children | | | | | |
|  | ***Clinical setting*** | Emergency department | | | | | |
|  | ***Prevalence [%] (CI)*** | Not documented | | | | | |
|  | ***Noteworthy incl. crit.*** | - | | | | | |
|  | ***Noteworthy excl. crit.*** | - | | | | | |
| **Index test(s)** | POCUS | | | | | | |
| **Reference standard(s)** | Pathology, surgery, RADUS or CT | | | | | | |
| **DTA-SR characteristics** | ***No. of studies*** | 11 | | | | | |
|  | ***No. of participants*** | Not reported | | | | | |
|  | ***Year range*** | 2008 - 2022 | | | | | |
|  | ***Quality assessment*** | QUADAS-2 (Various with high risk of bias overall, not reported separately) | | | | | |
| **Diagnostic accuracy** | ***Measure*** | Sensitivity [%] (95% CI):  65.3 (52.7; 77.9) | | | Specificity [%] (95% CI):  95.6 (92.2; 99.0) | | |
|  | ***GRADE*** | Low ⊕⊖⊕⊖ | | | Low ⊕⊖⊕⊖ | | |
| **Method. quality**  **(mod AMSTAR2)** | **Overall** | Moderate | | | | | |
| **Further investigations, sub- or sensitivity analyses** | **Index test** | | **Reference standard** | **Studies (Particip.)** | | **Sens. [%], 95% CI** | **Spec. [%], 95% CI** |
|  |  | |  |  | |  |  |
|  |  | |  |  | |  |  |
|  |  | |  |  | |  |  |
|  |  | |  |  | |  |  |
|  |  | |  |  | |  |  |
| **Conclusions of study authors** | *“POCUS has increasingly been utilized in the emergency department over the past two decades. It also indicates that POCUS in the emergency department can accurately diagnose clinical conditions such as small bowel obstruction, retinal detachment, abscess, clavicle fractures, distal forearm fractures, metacarpal fractures, skull fractures, pleural effusion, and deep venous thrombosis. In addition, we have demonstrated that POCUS can aid in ruling in appendicitis, foreign bodies, and cholecystitis due to its high specificity.”* | | | | | | |

| **Terasava (2004)**[111] – Appendicitis | | | | | | | |
| --- | --- | --- | --- | --- | --- | --- | --- |
| **Clinical context** | ***Patient population*** | Adults and adolescents (≥ 14 years) | | | | | |
|  | ***Clinical setting*** | Emergency department, medical ward | | | | | |
|  | ***Prevalence [%] (CI)*** | Not documented | | | | | |
|  | ***Noteworthy incl. crit.*** | Prospective cohorts of patients with suspected appendicitis | | | | | |
|  | ***Noteworthy excl. crit.*** | pregnancy | | | | | |
| **Index test(s)** | B-mode ultrasound, curved and / or linear | | | | | | |
| **Reference standard(s)** | Surgery with histology and clinical follow-up | | | | | | |
| **DTA-SR characteristics** | ***No. of studies*** | 14 | | | | | |
|  | ***No. of participants*** | 1516 | | | | | |
|  | ***Year range*** | 1988 - 2002 | | | | | |
|  | ***Quality assessment*** | QUADAS-2 (no low risk of bias studies) | | | | | |
|  | ***Special remark*** | Non-diagnostic tests were excluded from main statistical analysis.  Includes both suspected and atypical presentation. | | | | | |
| **Diagnostic accuracy** | ***Measure*** | Sensitivity [%] (95% CI):  86.0 (83.0; 88.0) | | | Specificity [%] (95% CI):  81.0 (78.0; 84.0) | | |
|  | ***GRADE*** | Moderate ⊕⊖⊕⊕ | | | Moderate ⊕⊖⊕⊕ | | |
| **Method. quality**  **(mod AMSTAR2)** | **Overall** | Moderate | | | | | |
| **Further investigations, sub- or sensitivity analyses** | **Index test** | | **Reference standard** | **Studies (Particip.)** | | **Sens. [%], 95% CI** | **Spec. [%], 95% CI** |
|  | B-mode ultrasound, adults only | | Surgery with histology and clinical follow-up |  | | 81 (72; 88) | 80 (73; 86) |
|  |  | |  |  | |  |  |
|  |  | |  |  | |  |  |
|  |  | |  |  | |  |  |
| **Conclusions of study authors** | *"Computed tomography is probably more accurate than ultrasonography for diagnosing appendicitis in adults and adolescents. Prospective studies that apply gold standard diagnostic testing to all study participants would more reliably estimate the true diagnostic accuracy of these tests.”* | | | | | | |

| **Wang (2023)**[112] **–** Appendicitis | | | | | | | |
| --- | --- | --- | --- | --- | --- | --- | --- |
| **Clinical context** | ***Patient population*** | Pregnant women | | | | | |
|  | ***Clinical setting*** | Not documented | | | | | |
|  | ***Prevalence [%] (CI)*** | Not documented | | | | | |
|  | ***Noteworthy incl. crit.*** | - | | | | | |
|  | ***Noteworthy excl. crit.*** | - | | | | | |
| **Index test(s)** | Compression in B-mode ultrasound | | | | | | |
| **Reference standard(s)** | Pathology, surgery, follow-up | | | | | | |
| **DTA-SR characteristics** | ***No. of studies*** | 4 | | | | | |
|  | ***No. of participants*** | 239 | | | | | |
|  | ***Year range*** | 2015 - 2021 | | | | | |
|  | ***Quality assessment*** | QUADAS-2 | | | | | |
|  | ***Special remark*** |  | | | | | |
| **Diagnostic accuracy** | ***Measure*** | Sensitivity [%] (95% CI):  56.0 (45.0;65.0) | | | Specificity [%] (95% CI):  88.0 (64.0;97.0) | | |
|  | ***GRADE*** | Very low ⊖⊖⊖⊖ | | | Very low ⊖⊖⊖⊖ | | |
| **Method. quality**  **(mod AMSTAR2)** | **Overall** | Low | | | | | |
| **Further investigations, sub- or sensitivity analyses** | **Index test** | | **Reference standard** | **Studies (Particip.)** | | **Sens. [%], 95% CI** | **Spec. [%], 95% CI** |
|  |  | |  |  | |  |  |
|  |  | |  |  | |  |  |
|  |  | |  |  | |  |  |
|  |  | |  |  | |  |  |
| **Conclusions of study authors** | *“As a non-invasive and convenient method, ultrasonography showed moderate accuracy in detecting appendicitis in pregnant women. In many cases, it is a viable imaging technology that does not expose the fetus or the mother to ionizing radiation, making it a viable choice for pregnant patients with suspected appendicitis. In addition, it can be done at any stage of pregnancy and has been shown to have no negative effects on the fetus.”* | | | | | | |

| **Weston (2005)**[113] – Appendicitis | | | | | | | |
| --- | --- | --- | --- | --- | --- | --- | --- |
| **Clinical context** | ***Patient population*** | Adults and children | | | | | |
|  | ***Clinical setting*** | Not documented | | | | | |
|  | ***Prevalence [%] (CI)*** | 41.0 (39.6; 42.4) | | | | | |
|  | ***Noteworthy incl. crit.*** | Original publications reporting one or more clinical study suitably designed to evaluate the diagnostic performance of the test, 50 patients or more | | | | | |
|  | ***Noteworthy excl. crit.*** | Pregnant women only studies, children only studies or >50% were children, nonroutine operators conducting the test, languages other than English | | | | | |
| **Index test(s)** | Compression in B-mode ultrasound | | | | | | |
| **Reference standard(s)** | Pathology, surgery | | | | | | |
| **DTA-SR characteristics** | ***No. of studies*** | 21 | | | | | |
|  | ***No. of participants*** | 4801 | | | | | |
|  | ***Year range*** | 1987 - 2002 | | | | | |
|  | ***Quality assessment*** | Diagnostic-specific checklist published by the Cochrane Screening and Diagnostic Tests Methods group | | | | | |
|  | ***Special remark*** |  | | | | | |
| **Diagnostic accuracy** | ***Measure*** | Sensitivity [%] (95% CI):  87.1 (84.9; 88.9) | | | Specificity [%] (95% CI):  92.8(91.5 ;93.9) | | |
|  | ***GRADE*** | Very low ⊖⊖⊖⊕ | | | Very low ⊖⊖⊖⊕ | | |
| **Method. quality**  **(mod AMSTAR2)** | **Overall** | Low | | | | | |
| **Further investigations, sub- or sensitivity analyses** | **Index test** | | **Reference standard** | **Studies (Particip.)** | | **Sens. [%], 95% CI** | **Spec. [%], 95% CI** |
|  |  | |  |  | |  |  |
|  |  | |  |  | |  |  |
|  |  | |  |  | |  |  |
|  |  | |  |  | |  |  |
| **Conclusions of study authors** | *“The current systematic review suggests that CT is a sensitive and specific diagnostic modality, it does not support the routine use of CT in the diagnosis of acute appendicitis. Other factors, such as the cost and time delay associated with obtaining a CT scan, are essential considerations.”* | | | | | | |

| **Yu (2005)**[114] - Appendicitis | | | | | | | |
| --- | --- | --- | --- | --- | --- | --- | --- |
| **Clinical context** | ***Patient population*** | All ages | | | | | |
|  | ***Clinical setting*** | unclear | | | | | |
|  | ***Prevalence [%] (CI)*** | Not documented | | | | | |
|  | ***Noteworthy incl. crit.*** | Original articles, The US criteria (41 42) for the diagnosis of appendicitis were an appendiceal diameter greater than 6 mm, a lack of compressibility, inflammation, echogenic periappendiceal  fat, appendicolith, adjacent fluid collections (and  hyperemia on color Doppler imaging). This study included  the articles that presented over three of the US criteria | | | | | |
|  | ***Noteworthy excl. crit.*** | - | | | | | |
| **Index test(s)** | Compression in B-mode ultrasound | | | | | | |
| **Reference standard(s)** | Pathology, surgery, follow-up | | | | | | |
| **DTA-SR characteristics** | ***No. of studies*** | 22 | | | | | |
|  | ***No. of participants*** | 2643 | | | | | |
|  | ***Year range*** | 1987 - 2001 | | | | | |
|  | ***Quality assessment*** |  | | | | | |
|  | ***Special remark*** |  | | | | | |
| **Diagnostic accuracy** | ***Measure*** | Sensitivity [%] (95% CI):  86.6 (85.4;88.0) | | | Specificity [%] (95% CI):  90.0 (88.9;91.2) | | |
|  | ***GRADE*** | Very low ⊝⊝⊝⊕ | | | Very low ⊝⊝⊝⊕ | | |
| **Method. quality**  **(mod AMSTAR2)** | **Overall** | Low | | | | | |
| **Further investigations, sub- or sensitivity analyses** | **Index test** | | **Reference standard** | **Studies (Particip.)** | | **Sens. [%], 95% CI** | **Spec. [%], 95% CI** |
|  | Dominant age: young | |  |  | | 90 (88; 93) | 94 (92; 96) |
|  | Dominant age: adult | |  |  | | 83 (78; 87) | 88 (85; 92) |
|  | Dominant age: old | |  |  | | 58 (51; 65) | 83 (77; 88) |
|  | Highly suggestive clinical status | |  |  | | 93 (89; 97) | 92 (88; 97) |
|  | Intermediate suggestive | |  |  | | 80 (74; 86) | 89 (85; 94) |
|  | Less likely | |  |  | | 75 (64; 87) | 94 (87; 100) |
|  | Radiologist | |  |  | | 85 (83; 87) | 93 (92; 94) |
|  | Non-radiologist | |  |  | | 98 (96; 100) | 73 (66; 80) |
|  | Gray scale Ultrasound | |  |  | | 82 (78; 86) | 94 (92; 97) |
|  | Colo-doppler ultrasound | |  |  | | 80 (76; 84) | 93 (90; 95) |
| **Conclusions of study authors** | *“In conclusion, ultrasound may be suggested as a useful diagnostic method for acute appendicitis, especially when the symptomatic patients are younger age, male and have clear clinical suggestions of disease. This procedure is evidently user-dependent and it has to be performed by a well-trained physician prior to the decision-making regarding an appendectomy.”* | | | | | | |

###### Biliary system

| **Childs (2023)**[115] – Acute cholecystitis | | | | | | | |
| --- | --- | --- | --- | --- | --- | --- | --- |
| **Clinical context** | ***Patient population*** | Adults suspected to have acute cholecystitis not in critical care unit | | | | | |
|  | ***Clinical setting*** | Mainly radiology | | | | | |
|  | ***Prevalence [%] (CI)*** | 48.0 (30.0; 52.7) | | | | | |
|  | ***Noteworthy incl. crit.*** | Clear diagnostic criteria, surgery or clinical follow-up as reference standard, sufficient data reported | | | | | |
|  | ***Noteworthy excl. crit.*** | Case reports, patients in critical care unit, suspected cases of acalculous cholecystitis or gallstone pancreatitis | | | | | |
| **Index test(s)** | Ultrasound (heterogeneity of combination of signs) | | | | | | |
| **Reference standard(s)** | Surgery or follow-up | | | | | | |
| **DTA-SR characteristics** | ***No. of studies*** | 18 | | | | | |
|  | ***No. of participants*** | 5714 | | | | | |
|  | ***Year range*** | 2000 - 2020 | | | | | |
|  | ***Quality assessment*** | QUADAS, studies included despite insufficient quality | | | | | |
|  | ***Special remark*** | 17 different diagnostic criteria provided in the 18 studies investigating the performance of ultrasound. Prevalence of acute cholecystitis in the included articles ranged from 9.4 to 98.0%. | | | | | |
| **Diagnostic accuracy** | ***Measure*** | Sensitivity [%] (95% CI):  69.0 (62.0; 76.0) | | | Specificity [%] (95% CI):  79.0 (71.0; 85.0) | | |
|  | ***GRADE*** | Low ⊕⊖⊕⊖ | | | Low ⊕⊖⊕⊖ | | |
| **Method. quality**  **(mod AMSTAR2)** | **Overall** | Moderate | | | | | |
| **Further investigations, sub- or sensitivity analyses** | **Index test** | | **Reference standard** | **Studies (Particip.)** | | **Sens. [%], 95% CI** | **Spec. [%], 95% CI** |
|  |  | |  |  | |  |  |
|  |  | |  |  | |  |  |
|  |  | |  |  | |  |  |
|  |  | |  |  | |  |  |
| **Conclusions of study authors** | *“Recent data suggests that CT may have a higher sensitivity than US for diagnosing acute cholecystitis, with similar specificity.”* | | | | | | |

| **De Oliveira (2024)**[116] – Acute cholecystitis | | | | | | | |
| --- | --- | --- | --- | --- | --- | --- | --- |
| **Clinical context** | ***Patient population*** | Adults and children | | | | | |
|  | ***Clinical setting*** | Not documented despite exclusion of intensive care units | | | | | |
|  | ***Prevalence [%] (CI)*** | 29.9 (28.8; 31.0) | | | | | |
|  | ***Noteworthy incl. crit.*** | Diagnostic accuracy of ultrasound and CT for acute cholecystitis, defined imaging criteria, surgery or follow-up as reference standard | | | | | |
|  | ***Noteworthy excl. crit.*** | Non-English, abstracts/case reports, traumatic patients, intensive care unit, outdated CT, POCUS evaluation | | | | | |
| **Index test(s)** | B-mode ultrasound (gallstone, thickening of the wall, sonographic Murphy's sign or not defined threshold) | | | | | | |
| **Reference standard(s)** | Surgery, follow-up | | | | | | |
| **DTA-SR characteristics** | ***No. of studies*** | 44 | | | | | |
|  | ***No. of participants*** | 2075 | | | | | |
|  | ***Year range*** | 1979 - 2023 | | | | | |
|  | ***Quality assessment*** | QUADAS-2 (assessment only for studies included in the head to head analysis [primary analysis]) | | | | | |
|  | ***Special remark*** |  | | | | | |
| **Diagnostic accuracy** | ***Measure*** | Sensitivity [%] (95% CI):  77.4 (69.9; 83.5) | | | Specificity [%] (95% CI):  88.4 (83.1; 92.2) | | |
|  | ***GRADE*** | Low ⊝⊝⊝⊝ | | | Low ⊕⊝⊝⊕ | | |
| **Method. quality**  **(mod AMSTAR2)** | **Overall** | Low | | | | | |
| **Further investigations, sub- or sensitivity analyses** | **Index test** | | **Reference standard** | **Studies (Particip.)** | | **Sens. [%], 95% CI** | **Spec. [%], 95% CI** |
|  |  | |  |  | |  |  |
|  |  | |  |  | |  |  |
|  |  | |  |  | |  |  |
|  |  | |  |  | |  |  |
| **Conclusions of study authors** | *“The diagnostic performance of CT is comparable to US for the diagnosis of acute cholecystitis, with a high rate of concordance between the two modalities.”* | | | | | | |

| **Huang (2023)**[117] – Acute cholecystitis | | | | | | | |
| --- | --- | --- | --- | --- | --- | --- | --- |
| **Clinical context** | ***Patient population*** | Adults | | | | | |
|  | ***Clinical setting*** | Not documented | | | | | |
|  | ***Prevalence [%] (CI)*** | Not documented | | | | | |
|  | ***Noteworthy incl. crit.*** | Articles investigating the diagnostic performance of ultrasound for acute cholecystitis | | | | | |
|  | ***Noteworthy excl. crit.*** | Studies focused on acalculous cholecystitis | | | | | |
| **Index test(s)** | Ultrasound (any sign) | | | | | | |
| **Reference standard(s)** | Surgical pathology, discharge diagnosis, radiology-performed ultrasound, and telephone follow-up | | | | | | |
| **DTA-SR characteristics** | ***No. of studies*** | 14 (> 25% with high risk of bias) | | | | | |
|  | ***No. of participants*** | 2638 | | | | | |
|  | ***Year range*** | 2001 - 2023 | | | | | |
|  | ***Quality assessment*** | QUADAS-2, studies included despite insufficient quality | | | | | |
|  | ***Special remark*** |  | | | | | |
| **Diagnostic accuracy** | ***Measure*** | Sensitivity [%] (95% CI):  71.0 (67.0; 74.0) | | | Specificity [%] (95% CI):  92.0 (90.0; 93.0) | | |
|  | ***GRADE*** | Very low ⊖⊖⊕⊖ | | | Low ⊖⊖⊕⊕ | | |
| **Method. quality**  **(mod AMSTAR2)** | **Overall** | Low | | | | | |
| **Further investigations, sub- or sensitivity analyses** | **Index test** | | **Reference standard** | **Studies (Particip.)** | | **Sens. [%], 95% CI** | **Spec. [%], 95% CI** |
|  | Ultrasound by anyone | | Surgery, clinical follow-up, radiology-performed ultras. | 40 (8652) | | 71 (69; 72) | 85 (84; 86) |
|  | Ultrasound by surgeon | | Surgery, performed ultrasound. | 3 (369) | | 79 (71; 85) | 76 (69; 81) |
|  | Ultrasound by radiologist | | Surgery, clinical follow-up | 18 (4899) | | 68 (66;71) | 87 (86; 88) |
|  | Ultrasound (Gallbladder wall thickness) | | NA | 8 | | 45 (40; 50) | 84 (81;87) |
|  | Ultrasound (Gallstone) | | NA | 7 | | 88 (84; 91) | 71 (65; 76) |
|  | Ultrasound (Peri-Gallbladder fluid) | | NA | 7 | | 26 (22; 31) | 92 (89; 93) |
|  | Sonographic (Murphy sign) | | NA | 7 | | 51 (46; 46) | 75 (72; 78) |
| **Conclusions of study authors** | “*Ultrasound is a good imaging modality for the diagnosis of acute cholecystitis with discriminative power. Emergency physician-performed ultrasound has a similar diagnostic performance to those by radiologists. Further investigations would be needed for the impact of US on the clinical management process and patient-centered outcomes.”* | | | | | | |

| **Kiewiet (2012)**[118] – Acute Cholecystitis | | | | | | | |
| --- | --- | --- | --- | --- | --- | --- | --- |
| **Clinical context** | ***Patient population*** | Adults | | | | | |
|  | ***Clinical setting*** | Not documented | | | | | |
|  | ***Prevalence [%] (CI)*** | Not documented | | | | | |
|  | ***Noteworthy incl. crit.*** | - | | | | | |
|  | ***Noteworthy excl. crit.*** | Case reports, ICU setting | | | | | |
| **Index test(s)** | B-mode ultrasound | | | | | | |
| **Reference standard(s)** | Surgery and/or clinical follow-up | | | | | | |
| **DTA-SR characteristics** | ***No. of studies*** | 26 | | | | | |
|  | ***No. of participants*** | 2847 | | | | | |
|  | ***Year range*** | 1978 - 2010 | | | | | |
|  | ***Quality assessment*** | QUADAS (poor to moderate quality) | | | | | |
|  | ***Special remark*** | Studies implemented different ultrasound protocols and  Scanning systems | | | | | |
| **Diagnostic accuracy** | ***Measure*** | Sensitivity [%] (95% CI):  81.0 (75.0; 87.0) | | | Specificity [%] (95% CI):  83.0 (74.0; 89.0) | | |
|  | ***GRADE*** | Low ⊕⊖⊕⊖ | | | Low ⊕⊖⊕⊖ | | |
| **Method. quality**  **(mod AMSTAR2)** | **Overall** | Moderate | | | | | |
| **Further investigations, sub- or sensitivity analyses** | **Index test** | | **Reference standard** | **Studies (Particip.)** | | **Sens. [%], 95% CI** | **Spec. [%], 95% CI** |
|  | B-mode ultrasound, before 01/01/1990 | | Surgery and/or clinical follow-up | NA | | 87 (78; 92) | 75 (64; 83) |
|  | B-mode ultrasound, after 01/01/1990 | | Surgery and/or clinical follow-up | NA | | 80 (70; 87) | 82 (68; 91) |
|  | Presence of gallstones | | Surgery and/or clinical follow-up | NA | | 80 (56; 93) | 79 (68; 87) |
|  | Multiple criteria | | Surgery and/or clinical follow-up | NA | | 81 (74; 87) | 84 (73; 91) |
| **Conclusions of study authors** | *"Cholescintigraphy has the highest diagnostic accuracy of all imaging modalities in detection of acute cholecystitis. The diagnostic accuracy of ultrasound has a substantial margin of error, comparable to that of MR imaging, while CT is still underevaluated.”* | | | | | | |

| **Popat (2024)**[110] – Acute Cholecystitis | | | | | | | |
| --- | --- | --- | --- | --- | --- | --- | --- |
| **Clinical context** | ***Patient population*** | Adults and children | | | | | |
|  | ***Clinical setting*** | Emergency department | | | | | |
|  | ***Prevalence [%] (CI)*** | Not documented | | | | | |
|  | ***Noteworthy incl. crit.*** | - | | | | | |
|  | ***Noteworthy excl. crit.*** | - | | | | | |
| **Index test(s)** | POCUS | | | | | | |
| **Reference standard(s)** | Pathology, surgery, RADUS, CT | | | | | | |
| **DTA-SR characteristics** | ***No. of studies*** | 4 | | | | | |
|  | ***No. of participants*** | Not documented | | | | | |
|  | ***Year range*** | 2010 - 2021 | | | | | |
|  | ***Quality assessment*** | QUADAS-2 (Various with high risk of bias overall, not reported separately) | | | | | |
| **Diagnostic accuracy** | ***Measure*** | Sensitivity [%] (95% CI):  74.7 (58.1; 91.4) | | | Specificity [%] (95% CI):  95.6 (92.2; 99.0) | | |
|  | ***GRADE*** | Low ⊕⊖⊕⊖ | | | Moderate ⊕⊖⊕⊕ | | |
| **Method. quality**  **(mod AMSTAR2)** | **Overall** | Moderate | | | | | |
| **Further investigations, sub- or sensitivity analyses** | **Index test** | | **Reference standard** | **Studies (Particip.)** | | **Sens. [%], 95% CI** | **Spec. [%], 95% CI** |
|  |  | |  |  | |  |  |
|  |  | |  |  | |  |  |
|  |  | |  |  | |  |  |
|  |  | |  |  | |  |  |
|  |  | |  |  | |  |  |
| **Conclusions of study authors** | *“POCUS has increasingly been utilized in the emergency department over the past two decades. It also indicates that POCUS in the emergency department can accurately diagnose clinical conditions such as small bowel obstruction, retinal detachment, abscess, clavicle fractures, distal forearm fractures, metacarpal fractures, skull fractures, pleural effusion, and deep venous thrombosis. In addition, we have demonstrated that POCUS can aid in ruling in appendicitis, foreign bodies, and cholecystitis due to its high specificity.”* | | | | | | |

| **Wilson (2023)**[119] – Acute cholecystitis | | | | | | | |
| --- | --- | --- | --- | --- | --- | --- | --- |
| **Clinical context** | ***Patient population*** | Adults | | | | | |
|  | ***Clinical setting*** | Emergency department | | | | | |
|  | ***Prevalence [%] (CI)*** | 16.9 (15.4; 18.5) | | | | | |
|  | ***Noteworthy incl. crit.*** | Adult patients with clinical suspicion of acute cholecystitis,  who underwent emergency physician–performed point-of care  ultrasound in the ED for the same indication | | | | | |
|  | ***Noteworthy excl. crit.*** | Nothing special | | | | | |
| **Index test(s)** | Ultrasound | | | | | | |
| **Reference standard(s)** | Surgical pathology, discharge diagnosis, radiology-performed ultrasound, and telephone follow-up | | | | | | |
| **DTA-SR characteristics** | ***No. of studies*** | 10 (9 with high risk of bias) | | | | | |
|  | ***No. of participants*** | 2356 | | | | | |
|  | ***Year range*** | 2001 - 2023 | | | | | |
|  | ***Quality assessment*** | QUADAS-2 | | | | | |
|  | ***Special remark*** | Diagnosis for acute cholecystitis: presence of gallstones plus any one of: 1) positive sonographic Murphy sign, 2) gallbladder wall thickness more than 3 mm, or 3) pericholecystic fluid. | | | | | |
| **Diagnostic accuracy** | ***Measure*** | Sensitivity [%] (95% CI):  78.6 (67.8; 86.5) | | | Specificity [%] (95% CI):  94.9 (89.7.0; 97.5) | | |
|  | ***GRADE*** | Low ⊕⊖⊕⊖ | | | High ⊕⊕⊕⊕ | | |
| **Method. quality**  **(mod AMSTAR2)** | **Overall** | Moderate | | | | | |
| **Further investigations, sub- or sensitivity analyses** | **Index test** | | **Reference standard** | **Studies (Particip.)** | | **Sens. [%], 95% CI** | **Spec. [%], 95% CI** |
|  | Ultrasound | | Only surgical pathology, discharge diagnosis, and then diagnostic imaging results | 7 (1772) | | 71 (62; 78) | 94 (88; 98) |
|  |  | |  |  | |  |  |
|  |  | |  |  | |  |  |
|  |  | |  |  | |  |  |
| **Conclusions of study authors** | *“In conclusion, our systematic review and meta-analysis demonstrated that emergency physician–performed point of-care ultrasound is moderately sensitive and highly specific in diagnosing acute cholecystitis.”* | | | | | | |

| **Gurusamy (2015)**[115] – Choledocholithiasis | | | | | | | |
| --- | --- | --- | --- | --- | --- | --- | --- |
| **Clinical context** | ***Patient population*** | People suspected of having common bile duct stones. | | | | | |
|  | ***Clinical setting*** | Surgery or gastroenterology in different parts of the world | | | | | |
|  | ***Prevalence [%] (CI)*** | Not documented | | | | | |
|  | ***Noteworthy incl. crit.*** | Studies providing cross-sectional information comparing one or more of the index tests against a reference standard in the appropriate patient population | | | | | |
|  | ***Noteworthy excl. crit.*** | Diagnostic case-control studies | | | | | |
| **Index test(s)** | B-mode ultrasound | | | | | | |
| **Reference standard(s)** | Endoscopic or surgical extraction of stones in people with a positive index test result or clinical follow-up (minimum 6 months) in people with a negative index test result. | | | | | | |
| **DTA-SR characteristics** | ***No. of studies*** | 5 (all low quality) | | | | | |
|  | ***No. of participants*** | 523 | | | | | |
|  | ***Year range*** | 1989 - 2006 | | | | | |
|  | ***Quality assessment*** | QUADAS-2, only low-quality studies | | | | | |
|  | ***Special remark*** |  | | | | | |
| **Diagnostic accuracy** | ***Measure*** | Sensitivity [%] (95% CI):  73.0 (44.0; 90.0) | | | Specificity [%] (95% CI):  91.0 (84.0; 95.0) | | |
|  | ***GRADE*** | Low ⊕⊖⊕⊖ | | | Moderate ⊕⊕⊕⊖ | | |
| **Method. quality**  **(mod AMSTAR2)** | **Overall** | Moderate | | | | | |
| **Further investigations, sub- or sensitivity analyses** | **Index test** | | **Reference standard** | **Studies (Particip.)** | | **Sens. [%], 95% CI** | **Spec. [%], 95% CI** |
|  |  | |  |  | |  |  |
|  |  | |  |  | |  |  |
|  |  | |  |  | |  |  |
|  |  | |  |  | |  |  |
| **Conclusions of study authors** | *“Many people may have common bile duct stones in spite of having a negative ultrasound or liver function test. Such people may have to be re-tested with other modalities if the clinical suspicion of common bile duct stones is very high because of their symptoms. False positive results are also possible and further non-invasive testing is recommended to confirm common bile duct stones to avoid the risks of invasive testing.”* | | | | | | |

| **Ross (2011)**[122] – Cholelithiasis | | | | | | | |
| --- | --- | --- | --- | --- | --- | --- | --- |
| **Clinical context** | ***Patient population*** | Patients with symptoms of suspected biliary colic including right upper quadrant pain, epigastric pain, or flank pain. | | | | | |
|  | ***Clinical setting*** | Emergency department | | | | | |
|  | ***Prevalence [%] (CI)*** | 60.0 (range: 46.0; 80.0) | | | | | |
|  | ***Noteworthy incl. crit.*** | Studies with patients with symptoms of suspected biliary colic, with ultrasound performed by an emergency physician and acceptable reference standard. Prospective studies. | | | | | |
|  | ***Noteworthy excl. crit.*** | No data could be obtained through author contact. | | | | | |
| **Index test(s)** | Ultrasound performed by an emergency physician | | | | | | |
| **Reference standard(s)** | Ultrasound performed by a radiologist, CT, MRI, surgery | | | | | | |
| **DTA-SR characteristics** | ***No. of studies*** | 8 | | | | | |
|  | ***No. of participants*** | 710 | | | | | |
|  | ***Year range*** | 2001 - 2010 | | | | | |
|  | ***Quality assessment*** | Modified QUADAS | | | | | |
|  | ***Special remark*** | > 80% of patients with radiological ultrasound as reference | | | | | |
| **Diagnostic accuracy** | ***Measure*** | Sensitivity [%] (95% CI):  89.8 (86.4; 92.5) | | | Specificity [%] (95% CI):  88.0 (83.7; 91.4) | | |
|  | ***GRADE*** | High ⊕⊕⊕⊕ | | | High ⊕⊕⊕⊕ | | |
| **Method. quality**  **(mod AMSTAR2)** | **Overall** | Moderate | | | | | |
| **Further investigations, sub- or sensitivity analyses** | **Index test** | | **Reference standard** | **Studies (Particip.)** | | **Sens. [%], 95% CI** | **Spec. [%], 95% CI** |
|  |  | |  |  | |  |  |
|  |  | |  |  | |  |  |
|  |  | |  |  | |  |  |
|  |  | |  |  | |  |  |
| **Conclusions of study authors** | *“This systematic review suggests that emergency bedside ultrasound is a useful adjunct for the diagnosis or exclusion of cholelithiasis in the emergency department. Based on the results of eight studies (n = 710) of variable quality, the estimates for emergency bedside ultrasound sensitivity and specificity for cholelithiasis are 89.8 and 88.0%, respectively. In patients presenting to the emergency department with a high pretest probability of symptomatic cholelithiasis, a positive emergency bedside ultrasound scan (likelihood ratio = 7.5) may be used to confirm the diagnosis and arrange for appropriate outpatient follow-up if symptoms have resolved. In patients with a low pretest probability, a negative emergency bedside ultrasound scan (likelihood ratio = 0.12) should prompt the clinician to consider an alternative diagnosis or further diagnostic testing.”* | | | | | | |

| **Wu (2023)**[120] – Gallbladder disease | | | | | | | |
| --- | --- | --- | --- | --- | --- | --- | --- |
| **Clinical context** | ***Patient population*** | Patients with suspected gallbladder disease | | | | | |
|  | ***Clinical setting*** | Not documented | | | | | |
|  | ***Prevalence [%] (CI)*** | Not documented | | | | | |
|  | ***Noteworthy incl. crit.*** | Suspected gallbladder disease, reference standard defined | | | | | |
|  | ***Noteworthy excl. crit.*** | - | | | | | |
| **Index test(s)** | B-mode ultrasound | | | | | | |
| **Reference standard(s)** | Pathology, surgery, ultrasound performed by a radiologist, CT | | | | | | |
| **DTA-SR characteristics** | ***No. of studies*** | 7 | | | | | |
|  | ***No. of participants*** | 1464 | | | | | |
|  | ***Year range*** | 2015 - 2023 | | | | | |
|  | ***Quality assessment*** | QUADAS-2 | | | | | |
|  | ***Special remark*** |  | | | | | |
| **Diagnostic accuracy** | ***Measure*** | Sensitivity [%] (95% CI):  86.0 (75.0; 93.0) | | | Specificity [%] (95% CI):  92.0 (87.0; 96.0) | | |
|  | ***GRADE*** | Very low ⊖⊖⊕⊖ | | | Very low ⊖⊖⊕⊖ | | |
| **Method. quality**  **(mod AMSTAR2)** | **Overall** | Low | | | | | |
| **Further investigations, sub- or sensitivity analyses** | **Index test** | | **Reference standard** | **Studies (Particip.)** | | **Sens. [%], 95% CI** | **Spec. [%], 95% CI** |
|  | Cholecystitis in B-Mode Ultrasound | | Surgery, pathology, ultrasound by radiologist, CT | 5 (1320) | | 73 (55; 86) | 93 (82; 98) |
|  | Cholelithiasis in B-Mode Ultrasound | | Ultrasound performed by a radiologist | 2 (144) | | 94 (91; 98) | 93 (91; 95) |
|  |  | |  |  | |  |  |
|  |  | |  |  | |  |  |
| **Conclusions of study authors** | *“Compared to conventional ultrasound, POCUS is characterized by decreased cost, resource utilization, and de-creased hospital length of stay. The findings of this meta-analysis indicate that POCUS was a favourable modality for the detection of patients suspected of gallbladder disease especially for cholelithiasis.”* | | | | | | |

###### Intestinal obstruction

| **Gottlieb (2017)**[123] – Small bowel obstruction | | | | | | | |
| --- | --- | --- | --- | --- | --- | --- | --- |
| **Clinical context** | ***Patient population*** | Adults and children | | | | | |
|  | ***Clinical setting*** | Emergency department | | | | | |
|  | ***Prevalence [%] (CI)*** | Not documented | | | | | |
|  | ***Noteworthy incl. crit.*** | Prospective studies assessing accuracy of ultrasound for detecting small bowel obstruction with dedicated reference standard | | | | | |
|  | ***Noteworthy excl. crit.*** | Retrospective studies, case series, abstracts | | | | | |
| **Index test(s)** | Fluid-filled dilated small bowel (diameter ≥25 mm) in B-mode Ultrasound | | | | | | |
| **Reference standard(s)** | Enteroclysis, surgery, discharge diagnosis, follow-up, CT | | | | | | |
| **DTA-SR characteristics** | ***No. of studies*** | 5 | | | | | |
|  | ***No. of participants*** | 625 | | | | | |
|  | ***Year range*** | 1996 - 2010 | | | | | |
|  | ***Quality assessment*** | QUADAS-2 | | | | | |
|  | ***Special remark*** |  | | | | | |
| **Diagnostic accuracy** | ***Measure*** | Sensitivity [%] (95% CI):  93.0 (89.0; 95.0) | | | Specificity [%] (95% CI):  96.0 (86.0; 99.0) | | |
|  | ***GRADE*** | Moderate ⊕⊖⊕⊕ | | | Low ⊕⊖⊕⊖ | | |
| **Method. quality**  **(mod AMSTAR2)** | **Overall** | Moderate | | | | | |
| **Further investigations, sub- or sensitivity analyses** | **Index test** | | **Reference standard** | **Studies (Particip.)** | | **Sens. [%], 95% CI** | **Spec. [%], 95% CI** |
|  | B-mode ultrasound (in every setting) | | Enteroclysis, surgery, discharge diagnosis, FUP, CT | 11 (1178) | | 92 (89; 95) | 97 (88; 99) |
|  |  | |  |  | |  |  |
|  |  | |  |  | |  |  |
|  |  | |  |  | |  |  |
| **Conclusions of study authors** | *“The existing literature suggests that ultrasound is a valuable tool in the diagnosis of small bowel obstruction with a sensitivity and specificity comparable to that of CT. Ultrasound may save time and radiation exposure, while also allowing for serial examinations of patients to assess for resolution of the small bowel obstruction. It may be particularly valuable in settings with limited or no access to CT. Future studies should include more studies in the Emergency Department setting, comparison of probe choices, and inclusion of more paediatric patients.”* | | | | | | |

| **Lin (2021)**[124] – Small bowel obstruction | | | | | | | |
| --- | --- | --- | --- | --- | --- | --- | --- |
| **Clinical context** | ***Patient population*** | Adults | | | | | |
|  | ***Clinical setting*** | Emergency department | | | | | |
|  | ***Prevalence [%] (CI)*** | 33.0 (30.1; 35.9) | | | | | |
|  | ***Noteworthy incl. crit.*** | Prospective and retrospective studies evaluating ultrasound for the diagnosis of bowel obstruction in adults. | | | | | |
|  | ***Noteworthy excl. crit.*** | - | | | | | |
| **Index test(s)** | Small bowel diameter ≥25 mm in B-mode ultrasound, free fluid or abnormal peristalsis | | | | | | |
| **Reference standard(s)** | Surgery, CT, Follow-up | | | | | | |
| **DTA-SR characteristics** | ***No. of studies*** | 7 | | | | | |
|  | ***No. of participants*** | 1004 | | | | | |
|  | ***Year range*** | 2006 - 2019 | | | | | |
|  | ***Quality assessment*** | QUADAS-2 (mostly studies with unclear or high risk of bias included) | | | | | |
|  | ***Special remark*** | Individual patient-level meta-analysis | | | | | |
| **Diagnostic accuracy** | ***Measure*** | Sensitivity [%] (95% CI):  95.0 (92.0; 96.0) | | | Specificity [%] (95% CI):  77.0.0 (73.0; 80.0) | | |
|  | ***GRADE*** | Moderate ⊕⊖⊕⊕ | | | Moderate ⊕⊖⊕⊕ | | |
| **Method. quality**  **(mod AMSTAR2)** | **Overall** | Moderate | | | | | |
| **Further investigations, sub- or sensitivity analyses** | **Index test** | | **Reference standard** | **Studies (Particip.)** | | **Sens. [%], 95% CI** | **Spec. [%], 95% CI** |
|  | Ultrasound (all Settings) | | Surgery, CT, follow-up | 15 (1536) | | 92 (89; 95) | 93 (85; 97) |
|  | Ultrasound (non ED Setting) | | Surgery, CT, follow-up | 7 (531) | | 90 (86; 93) | 98 (96; 100) |
|  | Ultrasound (North America) | | Surgery, CT, follow-up | 5 (524) | | 90 (85; 94) | 71 (65; 76) |
|  | Ultrasound (Europe) | | Surgery, CT, follow-up | 5 (352) | | 93 (88; 96) | 954 (90; 98) |
|  | Ultrasound (Asia) | | Surgery, CT, follow-up | 4 (562) | | 96 (94; 98) | 87 (82; 91) |
|  | Ultrasound (ED residents) | | Surgery, CT, follow-up | 4 (351) | | 94 (89; 97) | 84 (78; 89) |
|  | Ultrasound (ED attendings) | | Surgery, CT, follow-up | 2 (237) | | 89 (80; 94) | 61 (52; 69) |
| **Conclusions of study authors** | *“In conclusion, the results indicated that, overall, ultrasound is a reliable tool with good sensitivity and specificity for the diagnosis of small bowel obstruction. Because specificity was affected by the operator, setting, reference standard, and study location, some concerns remain regarding using ultrasound as the gold standard diagnostic modality for small bowel obstruction.”* | | | | | | |

| **Popat (2024)**[110] – Small bowel obstruction | | | | | | | |
| --- | --- | --- | --- | --- | --- | --- | --- |
| **Clinical context** | ***Patient population*** | Adults | | | | | |
|  | ***Clinical setting*** | Emergency department | | | | | |
|  | ***Prevalence [%] (CI)*** | Not documented | | | | | |
|  | ***Noteworthy incl. crit.*** | - | | | | | |
|  | ***Noteworthy excl. crit.*** | - | | | | | |
| **Index test(s)** | POCUS | | | | | | |
| **Reference standard(s)** | CT, surgery, pathology | | | | | | |
| **DTA-SR characteristics** | ***No. of studies*** | 6 | | | | | |
|  | ***No. of participants*** | Not documented | | | | | |
|  | ***Year range*** | 2016 – 2022 | | | | | |
|  | ***Quality assessment*** | QUADAS-2 (Various with high risk of bias overall, not reported separately) | | | | | |
| **Diagnostic accuracy** | ***Measure*** | Sensitivity [%] (95% CI):  93.0 (89.0; 98.0) | | | Specificity [%] (95% CI):  82.0 (69.0; 94.0) | | |
|  | ***GRADE*** | Low ⊕⊖⊕⊖ | | | Low ⊕⊖⊕⊖ | | |
| **Method. quality**  **(mod AMSTAR2)** | **Overall** | Moderate | | | | | |
| **Further investigations, sub- or sensitivity analyses** | **Index test** | | **Reference standard** | **Studies (Particip.)** | | **Sens. [%], 95% CI** | **Spec. [%], 95% CI** |
|  |  | |  |  | |  |  |
|  |  | |  |  | |  |  |
|  |  | |  |  | |  |  |
|  |  | |  |  | |  |  |
| **Conclusions of study authors** | *“POCUS has increasingly been utilized in the emergency department over the past two decades. It also indicates that POCUS in the emergency department can accurately diagnose clinical conditions such as small bowel obstruction, retinal detachment, abscess, clavicle fractures, distal forearm fractures, metacarpal fractures, skull fractures, pleural effusion, and deep venous thrombosis. In addition, we have demonstrated that POCUS can aid in ruling in appendicitis, foreign bodies, and cholecystitis due to its high specificity.”* | | | | | | |

| **Shokoohi (2023)**[125] – Small bowel obstruction | | | | | | | |
| --- | --- | --- | --- | --- | --- | --- | --- |
| **Clinical context** | ***Patient population*** | Adults with suspected small bowel obstruction | | | | | |
|  | ***Clinical setting*** | Emergency department | | | | | |
|  | ***Prevalence [%] (CI)*** | 33.0 (28.6; 37.4) | | | | | |
|  | ***Noteworthy incl. crit.*** | Prospective studies with adults that assessed POCUS by emergency physician to identify small bowel obstruction | | | | | |
|  | ***Noteworthy excl. crit.*** | Authors could not provide access to the original  clinical and sonographic data needed for analysis | | | | | |
| **Index test(s)** | Fluid-filled dilated small bowel (diameter ≥25 mm) in B-mode ultrasound | | | | | | |
| **Reference standard(s)** | Surgery, CT | | | | | | |
| **DTA-SR characteristics** | ***No. of studies*** | 5 | | | | | |
|  | ***No. of participants*** | 433 | | | | | |
|  | ***Year range*** | 2011 - 2022 | | | | | |
|  | ***Quality assessment*** | QUADAS-2 | | | | | |
|  | ***Special remark*** | Individual patient-level meta-analysis | | | | | |
| **Diagnostic accuracy** | ***Measure*** | Sensitivity [%] (95% CI):  83.0 (71.7; 90.4) | | | Specificity [%] (95% CI):  93.0 (55.3; 99.3) | | |
|  | ***GRADE*** | Low ⊕⊖⊕⊖ | | | Low ⊕⊖⊕⊖ | | |
| **Method. quality**  **(mod AMSTAR2)** | **Overall** | Moderate | | | | | |
| **Further investigations, sub- or sensitivity analyses** | **Index test** | | **Reference standard** | **Studies (Particip.)** | | **Sens. [%], 95% CI** | **Spec. [%], 95% CI** |
|  | Ultrasound by residents | | Surgery, CT | 4 (131) | | 73 (57; 85) | 88 (59; 98) |
|  | Ultrasound by fellows or attendings | | Surgery, CT | 4 (302) | | 88 (71; 95) | 91 (57; 99) |
|  | Ultrasound in patients with BMI ≥ 30 | | Surgery, CT | 4 (63) | | 72 (51; 88) | 90 (75; 97) |
|  | Ultrasound in patients with BMI < 30 | | Surgery, CT | 4 (179) | | 89 (80; 95) | 84 (75; 91) |
| **Conclusions of study authors** | *“In conclusion, this meta-analysis provides encouraging evidence supporting the use of POCUS by emergency physicians in evaluating small bowel obstruction. POCUS demonstrates high sensitivity and specificity in diagnosing small bowel obstruction, making it a valuable tool in the Emergency department.”* | | | | | | |

| **Taylor (2013)**[126] – Small bowel obstruction | | | | | | | |
| --- | --- | --- | --- | --- | --- | --- | --- |
| **Clinical context** | ***Patient population*** | Adults with suspected small bowel obstruction | | | | | |
|  | ***Clinical setting*** | Emergency department | | | | | |
|  | ***Prevalence [%] (CI)*** | 2-15% of all abdominal pain on emergency department | | | | | |
|  | ***Noteworthy incl. crit.*** | Studies focusing primarily on small bowel obstruction in an adult population. | | | | | |
|  | ***Noteworthy excl. crit.*** | Studies with tests not readily be available to the EP, studies focusing on a single radiographic sign, focusing on treatment | | | | | |
| **Index test(s)** | Small bowel diameter ≥25 mm in B-mode Ultrasound with absent or decreased peristalsis | | | | | | |
| **Reference standard(s)** | Surgery, CT | | | | | | |
| **DTA-SR characteristics** | ***No. of studies*** | 2 | | | | | |
|  | ***No. of participants*** | 250 | | | | | |
|  | ***Year range*** | 2011 | | | | | |
|  | ***Quality assessment*** | QUADAS-2 (1 low, 1 high risk of bias study included) | | | | | |
|  | ***Special remark*** |  | | | | | |
| **Diagnostic accuracy** | ***Measure*** | Sensitivity [%] (95% CI):  97.0 (92.0; 99.0) | | | Specificity [%] (95% CI):  90.0 (84.0; 95.0) | | |
|  | ***GRADE*** | Moderate ⊖⊕⊕⊕ | | | Low ⊖⊖⊕⊕ | | |
| **Method. quality**  **(mod AMSTAR2)** | **Overall** | Low | | | | | |
| **Further investigations, sub- or sensitivity analyses** | **Index test** | | **Reference standard** | **Studies (Particip.)** | | **Sens. [%], 95% CI** | **Spec. [%], 95% CI** |
|  | Ultrasound (all settings) | | Surgery, follow-up | 5 (449) | | 90 (86; 93) | 96 (91; 98) |
|  |  | |  |  | |  |  |
|  |  | |  |  | |  |  |
| **Conclusions of study authors** | *“Although lacking in study numbers, the findings for those studies involving ultrasound suggest that it is a good diagnostic imaging modality that could potentially improve the emergency department diagnosis. More emergency medicine-specific studies are needed in this area. In terms of the test-treatment threshold, emergency physicians should not hesitate to use nasogastric intubation for symptomatic relief early on, as there is very low risk of adverse events with this intervention.”* | | | | | | |

###### Gastrointestinal other

| **Van Hoving (2019)**[127] – Tuberculosis | | | | | | | |
| --- | --- | --- | --- | --- | --- | --- | --- |
| **Clinical context** | ***Patient population*** | HIV-positive adults | | | | | |
|  | ***Clinical setting*** | No restrictions | | | | | |
|  | ***Prevalence [%] (CI)*** | 41.9 (38.6; 45.2) | | | | | |
|  | ***Noteworthy incl. crit.*** | HIV-positive individuals) with a clinical suspicion of abdominal tuberculosis. Accuracy of abdominal ultrasound for M. tuberculosis | | | | | |
|  | ***Noteworthy excl. crit.*** | - | | | | | |
| **Index test(s)** | B-mode ultrasound of the abdomen | | | | | | |
| **Reference standard(s)** | Bacteriological confirmation of mycobacterium tuberculosis | | | | | | |
| **DTA-SR characteristics** | ***No. of studies*** | 5 | | | | | |
|  | ***No. of participants*** | 879 | | | | | |
|  | ***Year range*** | 2008 - 2019 | | | | | |
|  | ***Quality assessment*** | QUADAS-2 | | | | | |
| **Diagnostic accuracy** | ***Measure*** | Sensitivity [%] (95% CI):  63.0 (43.0; 79.0) | | | Specificity [%] (95% CI):  68.0 (42.0; 87.0) | | |
|  | ***GRADE*** | Very low ⊕⊖⊖⊖ | | | Very low ⊕⊖⊖⊖ | | |
| **Method. quality**  **(mod AMSTAR2)** | **Overall** | High | | | | | |
| **Further investigations, sub- or sensitivity analyses** | **Index test** | | **Reference standard** | **Studies (Particip.)** | | **Sens. [%], 95% CI** | **Spec. [%], 95% CI** |
|  |  | |  |  | |  |  |
|  |  | |  |  | |  |  |
|  |  | |  |  | |  |  |
|  |  | |  |  | |  |  |
|  |  | |  |  | |  |  |
| **Conclusions of study authors** | *“In HIV-positive individuals thought to have abdominal tuberculosis or disseminated tuberculosis with abdominal involvement, abdominal ultrasound appears to have 63% sensitivity and 68% specificity when tuberculosis was bacteriologically confirmed. These estimates are based on data that is limited, varied, and low-certainty.”* | | | | | | |

| **Fei (2017)**[128] – Acute Pancreatitis | | | | | | | |
| --- | --- | --- | --- | --- | --- | --- | --- |
| **Clinical context** | ***Patient population*** | Not documented | | | | | |
|  | ***Clinical setting*** | Not documented | | | | | |
|  | ***Prevalence [%] (CI)*** | 61.5 (56.9; 66.3) | | | | | |
|  | ***Noteworthy incl. crit.*** | Evaluation of CEUS for acute pancreatitis, all cases were compared with CT as the reference standard | | | | | |
|  | ***Noteworthy excl. crit.*** | Less than 20 patients | | | | | |
| **Index test(s)** | Contrast-enhanced Ultrasound (CEUS) | | | | | | |
| **Reference standard(s)** | CT | | | | | | |
| **DTA-SR characteristics** | ***No. of studies*** | 7 | | | | | |
|  | ***No. of participants*** | 421 | | | | | |
|  | ***Year range*** | 2006 - 2016 | | | | | |
|  | ***Quality assessment*** | QUADAS-2 (all studies with moderate risk of bias) | | | | | |
|  | ***Special remark*** |  | | | | | |
| **Diagnostic accuracy** | ***Measure*** | Sensitivity [%] (95% CI):  92.0 (88.0; 95.0) | | | Specificity [%] (95% CI):  84.0 (78.0; 90.0) | | |
|  | ***GRADE*** | Very Low ⊖⊖⊖⊕ | | | Very Low ⊖⊕⊖⊖ | | |
| **Method. quality**  **(mod AMSTAR2)** | **Overall** | Low | | | | | |
| **Further investigations, sub- or sensitivity analyses** | **Index test** | | **Reference standard** | **Studies (Particip.)** | | **Sens. [%], 95% CI** | **Spec. [%], 95% CI** |
|  |  | |  |  | |  |  |
|  |  | |  |  | |  |  |
|  |  | |  |  | |  |  |
| **Conclusions of study authors** | *“CEUS is a reliable, non-invasive imaging modality with no radiation exposure and a high*  *sensitivity and specificity for the assessment of severity of acute pancreatitis. Nonetheless, it should be applied cautiously, and large-scale, well-designed trials are necessary to assess its clinical value.”* | | | | | | |

| **Gupta (2020)**[129] – Budd Chiari syndrome | | | | | | | |
| --- | --- | --- | --- | --- | --- | --- | --- |
| **Clinical context** | ***Patient population*** | Not documented | | | | | |
|  | ***Clinical setting*** | Not documented | | | | | |
|  | ***Prevalence [%] (CI)*** | 84.4 (81.1; 88.0) | | | | | |
|  | ***Noteworthy incl. crit.*** | Studies of Doppler ultrasound, CT, and MRI with reference standard of catheter venography or surgery | | | | | |
|  | ***Noteworthy excl. crit.*** | - | | | | | |
| **Index test(s)** | Doppler ultrasound | | | | | | |
| **Reference standard(s)** | Digital subtraction angiography | | | | | | |
| **DTA-SR characteristics** | ***No. of studies*** | 6 | | | | | |
|  | ***No. of participants*** | 461 | | | | | |
|  | ***Year range*** | 1987 - 2014 | | | | | |
|  | ***Quality assessment*** | QUADAS-2 (5 high risk of bias studies included) | | | | | |
|  | ***Special remark*** | The retrospective study of Zhou et al. recruited 338 patients with confirmed Budd Chiari syndrome, there were no true-negative cases on Doppler ultrasound | | | | | |
| **Diagnostic accuracy** | ***Measure*** | Sensitivity [%] (95% CI):  89.0 (81.0; 94.0) | | | Specificity [%] (95% CI):  68.0 (3.0; 99.0) | | |
|  | ***GRADE*** | Low ⊕⊕⊖⊖ | | | Very low ⊕⊖⊖⊖ | | |
| **Method. quality**  **(mod AMSTAR2)** | **Overall** | Moderate | | | | | |
| **Further investigations, sub- or sensitivity analyses** | **Index test** | | **Reference standard** | **Studies (Particip.)** | | **Sens. [%], 95% CI** | **Spec. [%], 95% CI** |
|  |  | |  |  | |  |  |
|  |  | |  |  | |  |  |
|  |  | |  |  | |  |  |
| **Conclusions of study authors** | *“These results suggest that Doppler is an excellent first-line imaging test for patients suspected to have Budd Chiari syndrome. The advantages of Doppler ultrasound are easy availability and lower cost. Doppler ultrasound also provides functional information regarding the stenosis. However, the subjective nature of assessment and lack of evaluation of the extrahepatic collateral pathways is a significant limitation. However, this meta-analysis also highlights the need for prospective studies exploring the diagnostic performance of the imaging tests based on the site and type of venous obstruction as well as the duration of disease.”* | | | | | | |

| **Celik (2025)**[130] – Diverticulitis | | | | | | | |
| --- | --- | --- | --- | --- | --- | --- | --- |
| **Clinical context** | ***Patient population*** | Adults | | | | | |
|  | ***Clinical setting*** | Mostly in emergency settings, followed by surgical settings | | | | | |
|  | ***Prevalence [%] (CI)*** | 56.0 (CI Not documented) | | | | | |
|  | ***Noteworthy incl. crit.*** | Done by an emergency physician or another non-radiologist clinician. | | | | | |
|  | ***Noteworthy excl. crit.*** | Non-english articles | | | | | |
| **Index test(s)** | POCUS | | | | | | |
| **Reference standard(s)** | CT, or follow-up–based final diagnosis | | | | | | |
| **DTA-SR characteristics** | ***No. of studies*** | 6 | | | | | |
|  | ***No. of participants*** | 1420 | | | | | |
|  | ***Year range*** | 2020-2023 | | | | | |
|  | ***Quality assessment*** | QUADAS-2 (2 studies with high risk of bias) | | | | | |
| **Diagnostic accuracy** | ***Measure*** | Sensitivity [%] (95% CI):  93.9 (92.0; 95.3) | | | Specificity [%] (95% CI):  85.9 (72.0; 93.6) | | |
|  | ***GRADE*** | High ⊕⊕⊕⊕ | | | Low ⊕⊖⊕⊖ | | |
| **Method. quality**  **(mod AMSTAR2)** | **Overall** | Moderate | | | | | |
| **Further investigations, sub- or sensitivity analyses** | **Index test** | | **Reference standard** | **Studies (Particip.)** | | **Sens. [%], 95% CI** | **Spec. [%], 95% CI** |
|  | Only performed by ED physicians | | CT, follow-up | 3 (927) | | 94 (91; 95) | 91 (88; 94) |
|  |  | |  |  | |  |  |
|  |  | |  |  | |  |  |
|  |  | |  |  | |  |  |
|  |  | |  |  | |  |  |
| **Conclusions of study authors** | *“PoCUS as a highly effective diagnostic tool for diverticulitis, demonstrating high sensitivity and specificity, particularly in emergency settings. When performed by experienced and well-trained operators, specificity improves further. For complicated diverticulitis, PoCUS exhibits excellent specificity but only moderate sensitivity, making it useful for confirming complications but not excluding.”* | | | | | | |

| **Vijfschagt (2024)**[131] – Diverticulitis | | | | | | | |
| --- | --- | --- | --- | --- | --- | --- | --- |
| **Clinical context** | ***Patient population*** | Adults suspected of acute diverticulitis | | | | | |
|  | ***Clinical setting*** | Emergency department, radiology or surgery | | | | | |
|  | ***Prevalence [%] (CI)*** | 28 (26.4; 29.6) | | | | | |
|  | ***Noteworthy incl. crit.*** | Adults with suspected acute diverticulitis investigated with ultrasound and adequate reference standard | | | | | |
|  | ***Noteworthy excl. crit.*** | Population with known acute diverticulitis, healthy control | | | | | |
| **Index test(s)** | B-mode ultrasound | | | | | | |
| **Reference standard(s)** | Usually a combination of clinical outcomes, surgery, CT, follow-up, clinical experts | | | | | | |
| **DTA-SR characteristics** | ***No. of studies*** | 12 | | | | | |
|  | ***No. of participants*** | 2999 | | | | | |
|  | ***Year range*** | 2015 - 2023 | | | | | |
|  | ***Quality assessment*** | QUADAS-2 (10 studies with high risk of bias in at least 1 domain) | | | | | |
|  | ***Special remark*** |  | | | | | |
| **Diagnostic accuracy** | ***Measure*** | Sensitivity [%] (95% CI):  92.0 (86.0; 96.0) | | | Specificity [%] (95% CI):  94.0 (88.0; 97.0) | | |
|  | ***GRADE*** | Moderate ⊕⊕⊖⊕ | | | Low ⊕⊖⊖⊕ | | |
| **Method. quality**  **(mod AMSTAR2)** | **Overall** | Moderate | | | | | |
| **Further investigations, sub- or sensitivity analyses** | **Index test** | | **Reference standard** | **Studies (Particip.)** | | **Sens. [%], 95% CI** | **Spec. [%], 95% CI** |
|  | B-mode Ultrasound (After 2020) | | Surgery, pathology, ultrasound by radiologist, CT | 5 (1320) | | 92 (84; 96) | 96 (90; 99) |
|  |  | |  |  | |  |  |
|  |  | |  |  | |  |  |
|  |  | |  |  | |  |  |
| **Conclusions of study authors** | *“Ultrasound had a high diagnostic accuracy“* | | | | | | |

| **Pan (2024)**[132] **–** Gastric content and volume | | | | | | | |
| --- | --- | --- | --- | --- | --- | --- | --- |
| **Clinical context** | ***Patient population*** | Adults and children | | | | | |
|  | ***Clinical setting*** | Not documented | | | | | |
|  | ***Prevalence [%] (CI)*** | Not documented | | | | | |
|  | ***Noteworthy incl. crit.*** | - | | | | | |
|  | ***Noteworthy excl. crit.*** | Reviews, case reports, animal experiments, conference abstracts, duplicated data | | | | | |
| **Index test(s)** | B-mode ultrasound of the antrum in supine and right position | | | | | | |
| **Reference standard(s)** | Endoscopic findings, randomization to fluid intake, nature and volume of stomach contents through nasogastric tube | | | | | | |
| **DTA-SR characteristics** | ***No. of studies*** | 9 | | | | | |
|  | ***No. of participants*** | 523 | | | | | |
|  | ***Year range*** | 2014 - 2022 | | | | | |
|  | ***Quality assessment*** | QUADAS-2 | | | | | |
|  | ***Special remark*** |  | | | | | |
| **Diagnostic accuracy** | ***Measure*** | Sensitivity [%] (95% CI):  95.0 (84.0; 99.0) | | | Specificity [%] (95% CI):  88.0 (72.0; 95.0) | | |
|  | ***GRADE*** | Very low ⊕⊖⊖⊖ | | | Very low ⊕⊖⊖⊖ | | |
| **Method. quality**  **(mod AMSTAR2)** | **Overall** | Moderate | | | | | |
| **Further investigations, sub- or sensitivity analyses** | **Index test** | | **Reference standard** | **Studies (Particip.)** | | **Sens. [%], 95% CI** | **Spec. [%], 95% CI** |
|  |  | |  |  | |  |  |
|  |  | |  |  | |  |  |
|  |  | |  |  | |  |  |
|  |  | |  |  | |  |  |
|  |  | |  |  | |  |  |
|  |  | |  |  | |  |  |
|  |  | |  |  | |  |  |
|  |  | |  |  | |  |  |
| **Conclusions of study authors** | *“The results of this systematic review and meta-analysis show that ultrasound has high diagnostic accuracy in evaluating patients’ stomach contents. However, most of the existing studies on the determination of gastric contents by ultrasonic technology are feasibility studies with small sample sizes, lack standardization, and had high risk of bias. Therefore, more relevant research articles are needed to improve and expand the sample size. Ultrasonic evaluation of gastric contents is a convenient, feasible, and accurate imaging technology, which can be widely used in clinical work in the future.”* | | | | | | |

##### Genitourinary

###### Testicular torsion

| **Alexander (2025)**[133] – Testicular torsion | | | | | | | |
| --- | --- | --- | --- | --- | --- | --- | --- |
| **Clinical context** | ***Patient population*** | Male adults and children with suspected testicular torsion | | | | | |
|  | ***Clinical setting*** | Secondary or tertiary setting | | | | | |
|  | ***Prevalence [%] (CI)*** | 30.7 (29.4; 32.1) | | | | | |
|  | ***Noteworthy incl. crit.*** | Suspected testicular torsion in secondary or tertiary setting | | | | | |
|  | ***Noteworthy excl. crit.*** | Scrotal trauma, recent inguinal surgery | | | | | |
| **Index test(s)** | Color doppler ultrasound | | | | | | |
| **Reference standard(s)** | Surgery, follow-up | | | | | | |
| **DTA-SR characteristics** | ***No. of studies*** | 42 | | | | | |
|  | ***No. of participants*** | 4422 | | | | | |
|  | ***Year range*** | 1992 - 2023 | | | | | |
|  | ***Quality assessment*** | QUADAS-2; 49.2% judged to be high risk of bias | | | | | |
|  | ***Special remark*** |  | | | | | |
| **Diagnostic accuracy** | ***Measure*** | Sensitivity [%] (95% CI):  95.3 (91.4; 97.5) | | | Specificity [%] (95% CI):  98.3 (96.2; 99.3) | | |
|  | ***GRADE*** | Moderate ⊕⊖⊕⊕ | | | Moderate ⊕⊖⊕⊕ | | |
| **Method. quality**  **(mod AMSTAR2)** | **Overall** | Moderate | | | | | |
| **Further investigations, sub- or sensitivity analyses** | **Index test** | | **Reference standard** | **Studies (Particip.)** | | **Sens. [%], 95% CI** | **Spec. [%], 95% CI** |
|  |  | |  |  | |  |  |
|  |  | |  |  | |  |  |
|  |  | |  |  | |  |  |
|  |  | |  |  | |  |  |
| **Conclusions of study authors** | *“There is evidence to suggest that ultrasound is highly sensitive and specific for testicular torsion detection. The ideal patient pathway for suspected testicular torsion should integrate timely access to ultrasound alongside clinical assessment, with careful patient counselling. The application of POCUS in the emergency department by appropriately trained users does not compromise diagnostic accuracy and offers an important opportunity to achieve imaging in a time-sensitive fashion.”* | | | | | | |

| **McDowall (2018)**[134] – Testicular torsion | | | | | | | |
| --- | --- | --- | --- | --- | --- | --- | --- |
| **Clinical context** | ***Patient population*** | Children and adults | | | | | |
|  | ***Clinical setting*** | Mainly radiology department | | | | | |
|  | ***Prevalence [%] (CI)*** | Not documented | | | | | |
|  | ***Noteworthy incl. crit.*** | “Whirlpool sign” with relation to the topic of testicular torsion | | | | | |
|  | ***Noteworthy excl. crit.*** | Single-case studies | | | | | |
| **Index test(s)** | Whirlpool sign assessed during ultrasound | | | | | | |
| **Reference standard(s)** | Not mentioned | | | | | | |
| **DTA-SR characteristics** | ***No. of studies*** | 4 (3 with high risk of bias) | | | | | |
|  | ***No. of participants*** | 147 | | | | | |
|  | ***Year range*** | 2006 - 2016 | | | | | |
|  | ***Quality assessment*** | Oxford 2010 Critical Appraisal Skills Program (CASP) studies included despite insufficient quality | | | | | |
|  | ***Special remark*** |  | | | | | |
| **Diagnostic accuracy** | ***Measure*** | Sensitivity [%] (95% CI):  73.0 (65.0; 79.0) | | | Specificity [%] (95% CI):  99.0 (95.0; 100.0) | | |
|  | ***GRADE*** | Very low ⊖⊖⊖⊖ | | | Low ⊖⊕⊖⊕ | | |
| **Method. quality**  **(mod AMSTAR2)** | **Overall** | Low | | | | | |
| **Further investigations, sub- or sensitivity analyses** | **Index test** | | **Reference standard** | **Studies (Particip.)** | | **Sens. [%], 95% CI** | **Spec. [%], 95% CI** |
|  |  | |  |  | |  |  |
|  |  | |  |  | |  |  |
|  |  | |  |  | |  |  |
|  |  | |  |  | |  |  |
| **Conclusions of study authors** | *“The whirlpool sign is a valuable sign in diagnosing testicular torsion in the post-neonatal age group. Like all ultrasound interpretations and reports, it is reliant on operator skill and experience. The whirlpool sign should, however, not be utilized in isolation, but must be*  *interpreted in combination with clinical findings as well as other ultrasound features that have been described.”* | | | | | | |

| **Ota (2019)**[135] – Testicular torsion | | | | | | | |
| --- | --- | --- | --- | --- | --- | --- | --- |
| **Clinical context** | ***Patient population*** | Adults | | | | | |
|  | ***Clinical setting*** | Not documented | | | | | |
|  | ***Prevalence [%] (CI)*** | Not documented | | | | | |
|  | ***Noteworthy incl. crit.*** | Doppler ultrasound or ultrasound with suspected testicular torsion | | | | | |
|  | ***Noteworthy excl. crit.*** | Studies including only trauma patients | | | | | |
| **Index test(s)** | Color doppler ultrasound | | | | | | |
| **Reference standard(s)** | Surgical exploration or clinical follow-up for size of the testes | | | | | | |
| **DTA-SR characteristics** | ***No. of studies*** | 26 | | | | | |
|  | ***No. of participants*** | 2116 | | | | | |
|  | ***Year range*** | 1990 - 2016 | | | | | |
|  | ***Quality assessment*** | QUADAS-2, studies included despite insufficient quality | | | | | |
|  | ***Special remark*** |  | | | | | |
| **Diagnostic accuracy** | ***Measure*** | Sensitivity [%] (95% CI):  86.4 (79.4; 91.3) | | | Specificity [%] (95% CI):  95.3 (92.3; 97.2) | | |
|  | ***GRADE*** | Moderate ⊕⊕⊕⊖ | | | High ⊕⊕⊕⊕ | | |
| **Method. quality**  **(mod AMSTAR2)** | **Overall** | Moderate | | | | | |
| **Further investigations, sub- or sensitivity analyses** | **Index test** | | **Reference standard** | **Studies (Particip.)** | | **Sens. [%], 95% CI** | **Spec. [%], 95% CI** |
|  | Color Doppler ultrasound 1990 - 1999 | | Surgery or clinical follow-up | 8 (221) | | 83 (72; 91) | 91 (85; 95) |
|  | Color Doppler ultrasound 2000 - 2009 | | Surgery or clinical follow-up | 10 (931) | | 83 (69; 92) | 93 (87; 97) |
|  | Color Doppler ultrasound 2010 and later | | Surgery or clinical follow-up | 8 (964) | | 95 (84; 99) | 98 (93; 99) |
|  |  | |  |  | |  |  |
| **Conclusions of study authors** | “This meta-analysis demonstrated that ultrasound represents an effective imaging modality for diagnosing testicular torsion in adult patients with acute scrotal pain.” | | | | | | |

###### Urolithiasis

| **Laher (2020)**[136] – Urolithiasis | | | | | | | |
| --- | --- | --- | --- | --- | --- | --- | --- |
| **Clinical context** | ***Patient population*** | Children and adults | | | | | |
|  | ***Clinical setting*** | Not documented | | | | | |
|  | ***Prevalence [%] (CI)*** | Not documented | | | | | |
|  | ***Noteworthy incl. crit.*** | Clinical studies investigating twinkling artefact | | | | | |
|  | ***Noteworthy excl. crit.*** | Not documented | | | | | |
| **Index test(s)** | Twinkling artefact | | | | | | |
| **Reference standard(s)** | Nonenhanced computed tomography (15 studies). All other (7) used more than one modality, which included CT, abdominal radiography, excretory urography, stone passage, extracorporeal shock wave lithotripsy, intravenous urography/pyelography, ultrasonography and endoscopic visualization. | | | | | | |
| **DTA-SR characteristics** | ***No. of studies*** | 21 | | | | | |
|  | ***No. of participants*** | 4005 | | | | | |
|  | ***Year range*** | 2001 - 2017 | | | | | |
|  | ***Quality assessment*** | QUADAS-2, studies included despite insufficient quality | | | | | |
|  | ***Special remark*** |  | | | | | |
| **Diagnostic accuracy** | ***Measure*** | Sensitivity [%] (95% CI):  88.2 (87.1; 89.2) | | | Specificity [%] (95% CI):  79.2 (74.3; 84.3) | | |
|  | ***GRADE*** | Low ⊖⊖⊕⊕ | | | Low ⊖⊖⊕⊕ | | |
| **Method. quality**  **(mod AMSTAR2)** | **Overall** | Critically low | | | | | |
| **Further investigations, sub- or sensitivity analyses** | **Index test** | | **Reference standard** | **Studies (Particip.)** | | **Sens. [%], 95% CI** | **Spec. [%], 95% CI** |
|  | Twinkling artefact | | Computed tomography only | 12 (2061) | | 87 (85; 88) | 73 (65; 81) |
|  |  | |  |  | |  |  |
|  |  | |  |  | |  |  |
|  |  | |  |  | |  |  |
| **Conclusions of study authors** | *“Despite the suboptimal pooled sensitivity and specificity of the twinkling artefact sign and the large heterogeneity between published studies, the current body of evidence suggests that the Color Doppler ultrasound twinkling artefact sign may be useful as a complementary tool in the diagnostic workup of patients with suspected urolithiasis.”* | | | | | | |

| **Wong (2018)**[137] – Urolithiasis | | | | | | | |
| --- | --- | --- | --- | --- | --- | --- | --- |
| **Clinical context** | ***Patient population*** | Adults | | | | | |
|  | ***Clinical setting*** | Emergency department | | | | | |
|  | ***Prevalence [%] (CI)*** | Ranged from 35 to 84 | | | | | |
|  | ***Noteworthy incl. crit.*** | Suggestive symptom, emergency department physician or trainee | | | | | |
|  | ***Noteworthy excl. crit.*** | Not documented | | | | | |
| **Index test(s)** | POCUS | | | | | | |
| **Reference standard(s)** | Computer tomography, direct stone visualization, surgical findings | | | | | | |
| **DTA-SR characteristics** | ***No. of studies*** | 5 (all high quality) | | | | | |
|  | ***No. of participants*** | 1773 | | | | | |
|  | ***Year range*** | 2005 - 2016 | | | | | |
|  | ***Quality assessment*** | QUADAS-2, only high-quality studies included | | | | | |
|  | ***Special remark*** |  | | | | | |
| **Diagnostic accuracy** | ***Measure*** | Sensitivity [%] (95% CI):  70.2 (67.1; 73.2) | | | Specificity [%] (95% CI):  75.4 (72.5; 78.2) | | |
|  | ***GRADE*** | Low ⊕⊖⊕⊖ | | | High ⊕⊕⊕⊕ | | |
| **Method. quality**  **(mod AMSTAR2)** | **Overall** | Moderate | | | | | |
| **Further investigations, sub- or sensitivity analyses** | **Index test** | | **Reference standard** | **Studies (Particip.)** | | **Sens. [%], 95% CI** | **Spec. [%], 95% CI** |
|  | POCUS all 5 high + biggest low quality | | CT, Visualization, Surgery | 6 (1550) | | 65 (62; 68) | 75 (73; 77) |
|  | POCUS with moderate or greater hydronephrosis | | CT, Visualization, Surgery | 2 (1505) | | 29 (25; 32) | 94 (92; 96) |
|  |  | |  |  | |  |  |
|  |  | |  |  | |  |  |
| **Conclusions of study authors** | *“Point-of-care ultrasound has modest diagnostic accuracy for diagnosing nephrolithiasis. The finding of moderate or greater hydronephrosis is highly specific for the presence of any stone, and the presence of any hydronephrosis is suggestive of a larger (>5 mm) stone in those presenting with renal colic.”* | | | | | | |

###### Genitourinary other

| **Chen (2023)**[138] – Bladder outlet obstruction | | | | | | | |
| --- | --- | --- | --- | --- | --- | --- | --- |
| **Clinical context** | ***Patient population*** | Adults with lower urinary tract symptoms | | | | | |
|  | ***Clinical setting*** | Not defined | | | | | |
|  | ***Prevalence [%] (CI)*** | Not documented | | | | | |
|  | ***Noteworthy incl. crit.*** | Suggestive symptom, emergency department physician or trainee | | | | | |
|  | ***Noteworthy excl. crit.*** | Not meeting technical standards for measuring ultrasound-estimated bladder weight / detrusor wall thickness | | | | | |
| **Index test(s)** | Detrusor wall thickness | | | | | | |
| **Reference standard(s)** | Urodynamic pressure studies | | | | | | |
| **DTA-SR characteristics** | ***No. of studies*** | 10 | | | | | |
|  | ***No. of participants*** | 1204 | | | | | |
|  | ***Year range*** | 1997 - 2016 | | | | | |
|  | ***Quality assessment*** | QUADAS-2, studies included despite insufficient quality | | | | | |
|  | ***Special remark*** |  | | | | | |
| **Diagnostic accuracy** | ***Measure*** | Sensitivity [%] (95% CI):  68.0 (56.0; 78.0) | | | Specificity [%] (95% CI):  91.0 (82.0; 96.0) | | |
|  | ***GRADE*** | Very low ⊖⊖⊖⊖ | | | Very low ⊖⊖⊖⊕ | | |
| **Method. quality**  **(mod AMSTAR2)** | **Overall** | Critical low | | | | | |
| **Further investigations, sub- or sensitivity analyses** | **Index test** | | **Reference standard** | **Studies (Particip.)** | | **Sens. [%], 95% CI** | **Spec. [%], 95% CI** |
|  | Ultrasound-estimated bladder weight | | Urodynamic pressure studies | 6 (643) | | 88 (78; 93) | 81 (67; 90) |
|  |  | |  |  | |  |  |
|  |  | |  |  | |  |  |
|  |  | |  |  | |  |  |
| **Conclusions of study authors** | *“Detrusor wall thickness shows high specificity, and ultrasound-estimated bladder weight performs high sensitivity of diagnosing bladder outlet obstruction. Further studies are needed before these 2 indicators become routine tests. Detrusor wall thickness shows and ultrasound-estimated bladder weight are recommended for bladder outlet obstruction diagnosis before routine tests.”* | | | | | | |

| **Wei (2022)**[139] – Acute kidney injury | | | | | | | |
| --- | --- | --- | --- | --- | --- | --- | --- |
| **Clinical context** | ***Patient population*** | Not documented | | | | | |
|  | ***Clinical setting*** | Intensive care unit (21 studies), ward (2 studies) | | | | | |
|  | ***Prevalence [%] (CI)*** | Not documented | | | | | |
|  | ***Noteworthy incl. crit.*** | Critically illness or postoperative, only prospective studies | | | | | |
|  | ***Noteworthy excl. crit.*** | Not documented | | | | | |
| **Index test(s)** | Renal Resistive Index | | | | | | |
| **Reference standard(s)** | Development of acute kidney injury | | | | | | |
| **DTA-SR characteristics** | ***No. of studies*** | 23 | | | | | |
|  | ***No. of participants*** | 2400 | | | | | |
|  | ***Year range*** | 2011 - 2021 | | | | | |
|  | ***Quality assessment*** | QUADAS-2, studies included despite insufficient quality | | | | | |
|  | ***Special remark*** |  | | | | | |
| **Diagnostic accuracy** | ***Measure*** | Sensitivity [%] (95% CI):  76.0 (70.0; 81.0) | | | Specificity [%] (95% CI):  79.0 (72.0; 85.0) | | |
|  | ***GRADE*** | Very low ⊖⊖⊖⊖ | | | Very low ⊖⊖⊖⊕ | | |
| **Method. quality**  **(mod AMSTAR2)** | **Overall** | Low | | | | | |
| **Further investigations, sub- or sensitivity analyses** | **Index test** | | **Reference standard** | **Studies (Particip.)** | | **Sens. [%], 95% CI** | **Spec. [%], 95% CI** |
|  | Power Doppler | | Acute kidney injury | 23 (2400) | | 64 (48; 77) | 90 (81; 95) |
|  |  | |  |  | |  |  |
|  |  | |  |  | |  |  |
|  |  | |  |  | |  |  |
| **Conclusions of study authors** | *“This study showed that the predictive parameters of the RRI and Power doppler for subsequent AKI risk are comparable, while the specificity of the Power doppler was higher than that of the RRI for predicting AKI risk.”* | | | | | | |

##### Gynecological

###### Ectopic pregnancy

| **Han (2025)**[140] –Cesarean scar pregnancy | | | | | | | |
| --- | --- | --- | --- | --- | --- | --- | --- |
| **Clinical context** | ***Patient population*** | Women with suspected cesarean scar pregnancy | | | | | |
|  | ***Clinical setting*** | Not documented | | | | | |
|  | ***Prevalence [%] (CI)*** | Not documented | | | | | |
|  | ***Noteworthy incl. crit.*** | Studies testing MRI and transvaginal ultrasound simultaneously | | | | | |
|  | ***Noteworthy excl. crit.*** | Abdominal ultrasound, sample size <20 | | | | | |
| **Index test(s)** | Transvaginal ultrasound | | | | | | |
| **Reference standard(s)** | Surgery, follow-up | | | | | | |
| **DTA-SR characteristics** | ***No. of studies*** | 9 | | | | | |
|  | ***No. of participants*** | 713 | | | | | |
|  | ***Year range*** | 2014 - 2023 | | | | | |
|  | ***Quality assessment*** | QUADAS-2 | | | | | |
|  | ***Special remark*** |  | | | | | |
| **Diagnostic accuracy** | ***Measure*** | Sensitivity [%] (95% CI):  83.0 (77.0; 87.0) | | | Specificity [%] (95% CI):  74.0 (63.0; 83.0) | | |
|  | ***GRADE*** | Low ⊕⊕⊖⊕ | | | Very low ⊕⊕⊖⊖ | | |
| **Method. quality (mod AMSTAR2)** | **Overall** | Low | | | | | |
| **Further investigations, sub- or sensitivity analyses** | **Index test** | | **Reference standard** | **Studies (Particip.)** | | **Sens. [%], 95% CI** | **Spec. [%], 95% CI** |
|  | MRI | | Surgery | 9 | | 96 (94; 97) | 90 (84; 94) |
|  |  | |  |  | |  |  |
|  |  | |  |  | |  |  |
|  |  | |  |  | |  |  |
| **Conclusions of study authors** | *„In conclusion, the meta-analysis suggests that both MRI and transvaginal ultrasound can effectively diagnose cesarean scar pregnancy. However, compared with transvaginal ultrasound, MRI has a better diagnostic value for cesarean scar pregnancy, with higher sensitivity and specificity. Regarding the good diagnostic performance of ultra-sound, it is useful for subsequent management that patients with ultrasound suspicion of cesarean scar pregnancy should be sent to a reference center where MRI can express its full diagnostic potential concerning depth, topography of invasion and myometrial residue.”* | | | | | | |

| **Crochet (2013)**[141] – Ectopic pregnancy | | | | | | | |
| --- | --- | --- | --- | --- | --- | --- | --- |
| **Clinical context** | ***Patient population*** | Women with pain and vaginal bleeding or positive urinary pregnancy test, but without a living embryo located outside the uterus | | | | | |
|  | ***Clinical setting*** | Not documented | | | | | |
|  | ***Prevalence [%] (CI)*** | Not documented | | | | | |
|  | ***Noteworthy incl. crit.*** | Sample size was 100 or more; the study used a reference standard of surgical visualization, clinical follow-up | | | | | |
|  | ***Noteworthy excl. crit.*** | Narrative and systematic reviews, studies without proper control groups, retrospective studies, studies limited to ruptured ectopic pregnancies, studies with a primary focus other than ectopic pregnancy, studies focusing on the effectiveness of contraceptive devices, and studies involving assisted reproduction and in vitro fertilization were excluded. | | | | | |
| **Index test(s)** | Transvaginal ultrasound | | | | | | |
| **Reference standard(s)** | Surgery, follow-up | | | | | | |
| **DTA-SR characteristics** | ***No. of studies*** | 3 | | | | | |
|  | ***No. of participants*** | 6885 | | | | | |
|  | ***Year range*** | 1989 - 2005 | | | | | |
|  | ***Quality assessment*** | Rational Clinical Examination | | | | | |
|  | ***Special remark*** |  | | | | | |
| **Diagnostic accuracy** | ***Measure*** | Sensitivity [%] (95% CI):  88.0 (52.0; 69.0) | | | Specificity [%] (95% CI):  99.0 (96.0; 100) | | |
|  | ***GRADE*** | Very low ⊖⊖⊖⊖ | | | Very low ⊖⊖⊖⊕ | | |
| **Method. quality**  **(mod AMSTAR2)** | **Overall** | Low | | | | | |
| **Further investigations, sub- or sensitivity analyses** | **Index test** | | **Reference standard** | **Studies (Particip.)** | | **Sens. [%], 95% CI** | **Spec. [%], 95% CI** |
|  |  | |  |  | |  |  |
|  |  | |  |  | |  |  |
|  |  | |  |  | |  |  |
|  |  | |  |  | |  |  |
| **Conclusions of study authors** | *“Transvaginal sonography is the single best diagnostic modality for evaluating women with suspected ectopic pregnancy. The presence of abdominal pain or vaginal bleeding during early pregnancy should prompt a transvaginal sonogram and quantitative serum hCG testing.”* | | | | | | |

| **Richardson (2016)**[142] –Ectopic pregnancy | | | | | | | |
| --- | --- | --- | --- | --- | --- | --- | --- |
| **Clinical context** | ***Patient population*** | Women with pain and vaginal bleeding or positive urinary pregnancy test, but without a living embryo located outside the uterus | | | | | |
|  | ***Clinical setting*** | Not documented | | | | | |
|  | ***Prevalence [%] (CI)*** | 54.5 (51.4; 57.6) | | | | | |
|  | ***Noteworthy incl. crit.*** | Primary studies that reported original data regarding the  ultrasonographic diagnosis of an ectopic pregnancy | | | | | |
|  | ***Noteworthy excl. crit.*** | Case reports, case studies in which the sample size was fewer than 10 cases, commentaries, narrative reviews and letters | | | | | |
| **Index test(s)** | Transabdominal (TAU) or -vaginal (TVU) ultrasound (adnexal mass and free fluid) | | | | | | |
| **Reference standard(s)** | Surgery, final diagnosis | | | | | | |
| **DTA-SR characteristics** | ***No. of studies*** | 7 | | | | | |
|  | ***No. of participants*** | 1023 | | | | | |
|  | ***Year range*** | 1985 - 1993 | | | | | |
|  | ***Quality assessment*** | QUADAS-2 (1 study with high risk of bias, 4 with unclear) | | | | | |
|  | ***Special remark*** | 2 of 7 studies didn’t define clearly the reference standard | | | | | |
| **Diagnostic accuracy** | ***Measure*** | Sensitivity [%] (95% CI):  45.0 (34.0; 56.0) | | | Specificity [%] (95% CI):  96.0 (93.0; 98.0) | | |
|  | ***GRADE*** | Very low ⊕⊖⊖⊖ | | | Moderate ⊕⊕⊖⊕ | | |
| **Method. quality**  **(mod AMSTAR2)** | **Overall** | Moderate | | | | | |
| **Further investigations, sub- or sensitivity analyses** | **Index test** | | **Reference standard** | **Studies (Particip.)** | | **Sens. [%], 95% CI** | **Spec. [%], 95% CI** |
|  | TAU or TVU (Empty uterus) | | Surgery, final diagnosis | 13 (2499) | | 81 (42; 96) | 80 (69; 87) |
|  | TAU or TVU (Pseudosac) | | Surgery, final diagnosis | 8 (1838) | | 6 (3; 9) | 94 (76; 99) |
|  | TAU or TVU (Adnexal mass) | | Surgery, final diagnosis | 21 (2787) | | 64 (49; 76) | 91 (84; 96) |
|  | TAU or TVU (Free fluid) | | Surgery, final diagnosis | 19 (3232) | | 47 (33; 62) | 92 (86; 96) |
| **Conclusions of study authors** | *“In conclusion, this review is the first to collate comprehensively evidence of the accuracy of various ultrasonographic features to accurately confirm the presence of an ectopic pregnancy in the absence of a live extrauterine embryo. When an obvious extrauterine pregnancy is not present, the commonly used ultrasound features have poor sensitivity for identifying a tubal pregnancy, but they have good specificity. We can therefore infer ultrasound features are more useful for ruling in a tubal ectopic pregnancy, than for ruling one out. The findings are limited by the small number and poor quality of the included studies and by the considerable variation in index test and reference standard among the different studies.”* | | | | | | |

| **Stein (2010)**[143] – Ectopic pregnancy | | | | | | | |
| --- | --- | --- | --- | --- | --- | --- | --- |
| **Clinical context** | ***Patient population*** | Adults | | | | | |
|  | ***Clinical setting*** | Emergency department | | | | | |
|  | ***Prevalence [%] (CI)*** | 8.1 (3.8; 11.6) | | | | | |
|  | ***Noteworthy incl. crit.*** | Emergency department patients at risk for ectopic pregnancy, emergency physician performed ultrasound, single or combination criterion standard | | | | | |
|  | ***Noteworthy excl. crit.*** | Asymptomatic pelvic mass, only pediatric, pregnant, or non-pregnant population | | | | | |
| **Index test(s)** | Transabdominal or -vaginal ultrasound | | | | | | |
| **Reference standard(s)** | RADUS, gynaecology ultrasound, expert review, follow up | | | | | | |
| **DTA-SR characteristics** | ***No. of studies*** | 10 | | | | | |
|  | ***No. of participants*** | 2057 | | | | | |
|  | ***Year range*** | 1989 – 2009 | | | | | |
|  | ***Quality assessment*** | QUADAS (no low risk studies) | | | | | |
|  | ***Special remark*** | Random-effects model pools only sensitivity data; presented pooled data is from SROC curve | | | | | |
| **Diagnostic accuracy** | ***Measure*** | Sensitivity [%] (95% CI):  97.0 (92.0; 99.0) | | | Specificity [%] (95% CI):  76.0 (60.0; 80.0) | | |
|  | ***GRADE*** | Moderate ⊖⊕⊕⊕ | | | Very low ⊖⊖⊕⊖ | | |
| **Method. quality**  **(mod AMSTAR2)** | **Overall** | Low | | | | | |
| **Further investigations, sub- or sensitivity analyses** | **Index test** | | **Reference standard** | **Studies (Particip.)** | | **Sens. [%], 95% CI** | **Spec. [%], 95% CI** |
|  |  | |  |  | |  |  |
|  |  | |  |  | |  |  |
|  |  | |  |  | |  |  |
|  |  | |  |  | |  |  |
| **Conclusions of study authors** | *"This systematic review demonstrates that the use of bedside ultrasonography performed by emergency physicians consistently exhibits excellent test characteristics for ruling out ectopic pregnancy in a wide variety of clinical settings. These findings support the appropriate use of pelvic ultrasonography by emergency physicians in clinical practice.”* | | | | | | |

| **Celik (2026)**[144] – Intrauterine pregnancy | | | | | | | |
| --- | --- | --- | --- | --- | --- | --- | --- |
| **Clinical context** | ***Patient population*** | Women | | | | | |
|  | ***Clinical setting*** | Emergency department | | | | | |
|  | ***Prevalence [%] (CI)*** | Not documented | | | | | |
|  | ***Noteworthy incl. crit.*** | All prospective and retrospective studies and randomized controlled trials published in English | | | | | |
|  | ***Noteworthy excl. crit.*** | Studies with less than 20 Patients | | | | | |
| **Index test(s)** | POCUS by emergency physician (or non-radiologist, non-obstetrician) | | | | | | |
| **Reference standard(s)** | Ultrasound by obstetrician or radiologist | | | | | | |
| **DTA-SR characteristics** | ***No. of studies*** | 7 | | | | | |
|  | ***No. of participants*** | 1716 | | | | | |
|  | ***Year range*** | 1996 - 2002 | | | | | |
|  | ***Quality assessment*** | QUADAS-2 | | | | | |
|  | ***Special remark*** |  | | | | | |
| **Diagnostic accuracy** | ***Measure*** | Sensitivity [%] (95% CI):  86.0 (76.0; 93.0) | | | Specificity [%] (95% CI):  98.0 (87.0; 100) | | |
|  | ***GRADE*** | Low ⊕⊖⊕⊖ | | | Moderate ⊕⊕⊕⊖ | | |
| **Method. Quality**  **(mod AMSTAR2)** | **Overall** | Moderate | | | | | |
| **Further investigations, sub- or sensitivity analyses** | **Index test** | | **Reference standard** | **Studies (Particip.)** | | **Sens. [%], 95% CI** | **Spec. [%], 95% CI** |
|  | «Leave one out» (exclusion of one study with substantial heterogeneity) | | | 6 (NA) | | 89 (79; 94) | 99 (92; 100) |
|  | POCUS by ED physician | | Comprehensive transvaginal ultrasonography performed by radiologists or obstetricians | 4 (1017) | | 93 (86; 96) | 97 (83; 99) |
|  | POCUS by ED physician | | Additional ultrasonographic follow-up | 3 (699) | | 72 (67; 77) | 96 (92; 98) |
|  |  | |  |  | |  |  |
| **Conclusions of study authors** | *«Our systematic review and meta-analysis demonstrates that PoCUS performed by emergency physicians is a highly effective tool for diagnosing IUP in first-trimester patients presenting with pain and vaginal bleeding. These findings underscore the reliability of PoCUS as a diagnostic method in clinical practice, particularly for con- firming IUP and aiding in the exclusion of ectopic preg- nancy. However, caution is warranted when interpreting negative results. In such cases, additional follow-up eval- uations are recommended to ensure patient safety.»* | | | | | | |

| **Li (2024)**[145] – Retained products of conception | | | | | | | |
| --- | --- | --- | --- | --- | --- | --- | --- |
| **Clinical context** | ***Patient population*** | Women with suspected symptoms of retained products of conception | | | | | |
|  | ***Clinical setting*** | Not documented | | | | | |
|  | ***Prevalence [%] (CI)*** | Not documented | | | | | |
|  | ***Noteworthy incl. crit.*** | Diagnostic test accuracy studies comparing endometrial thickness alone, serum hCG alone, or their combination with a reference standard for retained products of conception in English or Chinese | | | | | |
|  | ***Noteworthy excl. crit.*** | Case reports, reviews, letters, studies without clear reference standard, studies with overlapping populations, studies focusing on ectopic pregnancy or trophoblastic neoplasms | | | | | |
| **Index test(s)** | Transabdominal or -vaginal ultrasound (Endometrial thickness) | | | | | | |
| **Reference standard(s)** | Pathology | | | | | | |
| **DTA-SR characteristics** | ***No. of studies*** | 15 | | | | | |
|  | ***No. of participants*** | 3268 | | | | | |
|  | ***Year range*** | 2014 - 2025 | | | | | |
|  | ***Quality assessment*** | QUADAS-2 (performance not documented) | | | | | |
|  | ***Special remark*** |  | | | | | |
| **Diagnostic accuracy** | ***Measure*** | Sensitivity [%] (95% CI):  72.0 (67.0; 77.0) | | | Specificity [%] (95% CI):  86.0 (82.0; 89.0) | | |
|  | ***GRADE*** | Very low ⊖⊖⊖⊕ | | | Low ⊖⊕⊖⊕ | | |
| **Method. quality**  **(mod AMSTAR2)** | **Overall** | Low | | | | | |
| **Further investigations, sub- or sensitivity analyses** | **Index test** | | **Reference standard** | **Studies (Particip.)** | | **Sens. [%], 95% CI** | **Spec. [%], 95% CI** |
|  | Transvaginal abdominal (Endometrial thickness) | | Pathology | 12 (2752) | | 76 (71; 80) | 87 (83; 90) |
|  | Transvaginal vaginal (Endometrial thickness) | | Pathology | 3 (516) | | 58 (49; 66) | 84 (78; 89)) |
|  |  | |  |  | |  |  |
|  |  | |  |  | |  |  |
| **Conclusions of study authors** | *“We suggest the clinical use of the combined protocol: “serum hCG (cutoff: 50 mIU/*  *mL) + Transvaginal-ultrasound-measured endometrial thickness (cutoff: 8 mm)” for retained products of conception diagnosis, with recognition of study limitations (e.g., heterogeneity in endometrial thickness measurement, limited cutoff exploration).”* | | | | | | |

| **Sundararajan (2024)**[146] – Retained products of conception | | | | | | | |
| --- | --- | --- | --- | --- | --- | --- | --- |
| **Clinical context** | ***Patient population*** | Women with suspected symptoms of retained products of conception after a full-term or preterm vaginal delivery or caesarean delivery, a miscarriage, or termination of pregnancy | | | | | |
|  | ***Clinical setting*** | Not documented | | | | | |
|  | ***Prevalence [%] (CI)*** | 27.2 (24.7; 29.7) | | | | | |
|  | ***Noteworthy incl. crit.*** | Prospective and retrospective cross-sectional or cohort studies that evaluated both the ultrasound findings (before management of retained products of conception) and the histopathologic results at all gestational ages. | | | | | |
|  | ***Noteworthy excl. crit.*** | Case-control studies, reviews, case reports, case series, and conference abstracts. Furthermore, studies evaluating hysteroscopy, magnetic resonance imaging, incomplete miscarriage outcomes, or reporting only hysteroscopic findings without corresponding sonographic assessment were also excluded. | | | | | |
| **Index test(s)** | Transvaginal ultrasound (echogenic mass) | | | | | | |
| **Reference standard(s)** | Pathology | | | | | | |
| **DTA-SR characteristics** | ***No. of studies*** | 9 | | | | | |
|  | ***No. of participants*** | 1237 | | | | | |
|  | ***Year range*** | 2005 - 2019 | | | | | |
|  | ***Quality assessment*** | QUADAS-2 (4 studies with high risk of bias) | | | | | |
|  | ***Special remark*** |  | | | | | |
| **Diagnostic accuracy** | ***Measure*** | Sensitivity [%] (95% CI):  91.5 (84.4; 95.5) | | | Specificity [%] (95% CI):  84.3 (61.5; 94.7) | | |
|  | ***GRADE*** | Very low ⊕⊖⊖⊖ | | | Very low ⊕⊖⊖⊖ | | |
| **Method. quality**  **(mod AMSTAR2)** | **Overall** | High | | | | | |
| **Further investigations, sub- or sensitivity analyses** | **Index test** | | **Reference standard** | **Studies (Particip.)** | | **Sens. [%], 95% CI** | **Spec. [%], 95% CI** |
|  | Transvaginal ultrasound (Endometrial thickness) | | Pathology | 4 (504) | | 67 (7; 98) | 87 (38; 99) |
|  | Transvaginal ultrasound (Color Doppler) | | Pathology | 8 (1838) | | 85 (75; 91) | 41 (21; 66) |
|  |  | |  |  | |  |  |
|  |  | |  |  | |  |  |
| **Conclusions of study authors** | *“Our review concluded that an echogenic mass is the most sensitive and specific predictor of retained products of conception after any pregnancy event. The most important limitation of our review is that the design of the studies included led to significant statistical heterogeneity.”* | | | | | | |

| **Garde (2023)**[147] – Adnexal torsion | | | | | | | |
| --- | --- | --- | --- | --- | --- | --- | --- |
| **Clinical context** | ***Patient population*** | Women of all ages (from premenarchal to postmenopausal) with suspected adnexal torsion | | | | | |
|  | ***Clinical setting*** | Not documented | | | | | |
|  | ***Prevalence [%] (CI)*** | 47.0 (12.0; 82.0) | | | | | |
|  | ***Noteworthy incl. crit.*** | Clinical suspicion of adnexal torsion | | | | | |
|  | ***Noteworthy excl. crit.*** | Studies with <20 patients | | | | | |
| **Index test(s)** | Ultrasound (transvaginal, transrectal, abdominal) using ovarian doppler-flow | | | | | | |
| **Reference standard(s)** | Surgery | | | | | | |
| **DTA-SR characteristics** | ***No. of studies*** | 14 (for adnexal torsion) | | | | | |
|  | ***No. of participants*** | 1742 | | | | | |
|  | ***Year range*** | 1998 - 2022 | | | | | |
|  | ***Quality assessment*** | QUADAS 2 | | | | | |
|  | ***Special remark*** |  | | | | | |
| **Diagnostic accuracy** | ***Measure*** | Sensitivity [%] (95% CI):  53.0 (34.0; 72.0)  53 (34 – 72) | | | Specificity [%] (95% CI):  95.0 (86.0; 98.0) | | |
|  | ***GRADE*** | Very low ⊖⊖⊖⊖ | | | Very low ⊖⊖⊖⊖ | | |
| **Method. quality**  **(mod AMSTAR2)** | **Overall** | Low | | | | | |
| **Further investigations, sub- or sensitivity analyses** | **Index test** | | **Reference standard** | **Studies (Particip.)** | | **Sens. [%], 95% CI** | **Spec. [%], 95% CI** |
|  | Ovarian edema | | Surgery | 8 | | 58 (38; 76) | 86 (61; 96) |
|  | Adnexal mass | | Surgery | 8 | | 69 (55; 81) | 46 (22; 71) |
|  | Whirlpool sign | | Surgery | 6 | | 65 (12; 96) | 91 (81; 96) |
|  | Pelvic fluid | | Surgery | 7 | | 55 (38; 71) | 69 (54; 80) |
| **Conclusions of study authors** | *«Ovarian edema, the whirlpool sign and absent intrao- varian blood flow as assessed by Doppler ultrasound are highly specific sonographic signs for diagnosing adnexal torsion. The presence of an adnexal mass or pelvic fluid have moderate diagnostic performance for detecting adnexal torsion. However, the quality of the evidence is limited»* | | | | | | |

| **Shakeel (2021)**[148] – Adnexal torsion | | | | | | | |
| --- | --- | --- | --- | --- | --- | --- | --- |
| **Clinical context** | ***Patient population*** | Pediatric or adult women with lower abdominal pain | | | | | |
|  | ***Clinical setting*** | Not documented | | | | | |
|  | ***Prevalence [%] (CI)*** | Not documented | | | | | |
|  | ***Noteworthy incl. crit.*** | Observational (cross-sectional) studies | | | | | |
|  | ***Noteworthy excl. crit.*** | Randomized control/clinical trials, and non-matched  Retrospective and prospective studies and chart reviews | | | | | |
| **Index test(s)** | Ultrasound and / or Doppler ultrasound | | | | | | |
| **Reference standard(s)** | Clinical analysis, clinical or radiological follow-up | | | | | | |
| **DTA-SR characteristics** | ***No. of studies*** | 12 | | | | | |
|  | ***No. of participants*** | 1187 | | | | | |
|  | ***Year range*** | 2000 - 2020 | | | | | |
|  | ***Quality assessment*** | No quality assessment | | | | | |
|  | ***Special remark*** |  | | | | | |
| **Diagnostic accuracy** | ***Measure*** | Sensitivity [%] (95% CI):  79.0 (63.0; 92.0) | | | Specificity [%] (95% CI):  76.0 (54.0; 93.0) | | |
|  | ***GRADE*** | Very low ⊖⊖⊖⊖ | | | Very low ⊖⊖⊖⊖ | | |
| **Method. quality**  **(mod AMSTAR2)** | **Overall** | Critically low | | | | | |
| **Further investigations, sub- or sensitivity analyses** | **Index test** | | **Reference standard** | **Studies (Particip.)** | | **Sens. [%], 95% CI** | **Spec. [%], 95% CI** |
|  | Doppler ultrasound | | clinical analysis, clinical or radiological follow-up | 7 (845) | | 80 (67; 93) | 88 (72; 100) |
|  |  | |  |  | |  |  |
|  |  | |  |  | |  |  |
|  |  | |  |  | |  |  |
| **Conclusions of study authors** | *“As a first line analytic examination for alleged adnexal torsion, ultrasound has good performance. Increment in quantity of adnexal torsion cases needs precise, rapid, as well as correct analytic extent. It has been utmost popular method as allied with further Imaging Modalities. MRI might compromise better specificity for investigating intricate morphology of ovary, nonetheless further proof is wanted. Beside with medical doubt as well as approximations. This study will offer sufficient appreciated evidence to understand sonographic markers impact during OT analysis precisely.”* | | | | | | |

| **Wattar (2021)**[149] – Adnexal torsion | | | | | | | |
| --- | --- | --- | --- | --- | --- | --- | --- |
| **Clinical context** | ***Patient population*** | Adults and children | | | | | |
|  | ***Clinical setting*** | Not documented | | | | | |
|  | ***Prevalence [%] (CI)*** | Not documented | | | | | |
|  | ***Noteworthy incl. crit.*** | Female patients presenting with symptoms of adnexal torsion | | | | | |
|  | ***Noteworthy excl. crit.*** | Asymptomatic pelvic mass, only pediatric, pregnant, or non-pregnant population | | | | | |
| **Index test(s)** | Ultrasound and / or Doppler ultrasound | | | | | | |
| **Reference standard(s)** | Surgical exploration with / without follow up | | | | | | |
| **DTA-SR characteristics** | ***No. of studies*** | 12 | | | | | |
|  | ***No. of participants*** | 1187 | | | | | |
|  | ***Year range*** | 1988 – 2018 | | | | | |
|  | ***Quality assessment*** | QUADAS-2 (overall moderate quality) | | | | | |
|  | ***Special remark*** |  | | | | | |
| **Diagnostic accuracy** | ***Measure*** | Sensitivity [%] (95% CI):  79.0 (63.0; 92.0) | | | Specificity [%] (95% CI):  76.0 (54.0; 93.0) | | |
|  | ***GRADE*** | Very low ⊕⊖⊖⊖ | | | Very low ⊕⊖⊖⊖ | | |
| **Method. quality**  **(mod AMSTAR2)** | **Overall** | Moderate | | | | | |
| **Further investigations, sub- or sensitivity analyses** | **Index test** | | **Reference standard** | **Studies (Particip.)** | | **Sens. [%], 95% CI** | **Spec. [%], 95% CI** |
|  | Doppler ultrasound only | | Surgical exploration ± Follow up | 7 (845) | | 80 (67; 97) | 88 (72; 100) |
|  | Ultrasound, adults only | | Surgical exploration ± Follow up | 3 (NA) | | 84 (34; 98) | 78 (42; 94) |
|  |  | |  |  | |  |  |
|  |  | |  |  | |  |  |
| **Conclusions of study authors** | *“Ultrasound has good performance as a first-line diagnostic test for women with suspected adnexal torsion. Magnetic resonance imaging could offer improved specificity to investigate complex ovarian morphology, but more evidence is needed.”* | | | | | | |

| **Adu-Bredu (2021)**[150] – Ovarian torsion | | | | | | | |
| --- | --- | --- | --- | --- | --- | --- | --- |
| **Clinical context** | ***Patient population*** | Children and women (pregnant and non-pregnant) | | | | | |
|  | ***Clinical setting*** | Not documented | | | | | |
|  | ***Prevalence [%] (CI)*** | 59.6 (55.9; 63.3) | | | | | |
|  | ***Noteworthy incl. crit.*** | Observational and randomized controlled studies were eligible. Studies reporting the ultrasonographic whirlpool sign with surgical or follow-up confirmation of ovarian torsion. Only studies in humans published in English were considered. | | | | | |
|  | ***Noteworthy excl. crit.*** | - | | | | | |
| **Index test(s)** | Whirlpool sign in transabdominal or -vaginal ultrasound | | | | | | |
| **Reference standard(s)** | Surgery, follow-up | | | | | | |
| **DTA-SR characteristics** | ***No. of studies*** | 7 | | | | | |
|  | ***No. of participants*** | 1023 | | | | | |
|  | ***Year range*** | 1998 - 2020 | | | | | |
|  | ***Quality assessment*** | QUADAS-2 (probably only low risk of bias included) | | | | | |
|  | ***Special remark*** |  | | | | | |
| **Diagnostic accuracy** | ***Measure*** | Sensitivity [%] (95% CI):  82.0 (78.0; 86.0) | | | Specificity [%] (95% CI):  81.0 (70.0; 90.0) | | |
|  | ***GRADE*** | Very low ⊖⊖⊖⊖ | | | Very low ⊖⊕⊖⊖ | | |
| **Method. quality**  **(mod AMSTAR2)** | **Overall** | Low | | | | | |
| **Further investigations, sub- or sensitivity analyses** | **Index test** | | **Reference standard** | **Studies (Particip.)** | | **Sens. [%], 95% CI** | **Spec. [%], 95% CI** |
|  |  | |  |  | |  |  |
|  |  | |  |  | |  |  |
|  |  | |  |  | |  |  |
|  |  | |  |  | |  |  |
| **Conclusions of study authors** | *“The whirlpool sign is a valuable sonographic feature in the diagnosis of ovarian torsion with a high sensitivity and specificity when performed using both transabdominal and endocavitary techniques. Efforts to identify the sonographic whirlpool sign should therefore be encouraged in cases of suspected ovarian torsion.”* | | | | | | |

##### Intervention related

###### Airway related

| **Alsabri (2026)**[151] – Endotracheal tube placement | | | | | | | |
| --- | --- | --- | --- | --- | --- | --- | --- |
| **Clinical context** | ***Patient population*** | Adults | | | | | |
|  | ***Clinical setting*** | ED and ICU | | | | | |
|  | ***Prevalence [%] (CI)*** | Not documented | | | | | |
|  | ***Noteworthy incl. crit.*** | The inclusion criteria [were expanded] to studies using capnography, chest auscultation chest X-ray, or direct laryngoscopy as acceptable reference standards | | | | | |
|  | ***Noteworthy excl. crit.*** | Studies without sufficient data for 2 × 2 table construction were analyzed separately for secondary outcomes (e.g., time-to confirmation) but not included in pooled diagnostic accuracy estimates. | | | | | |
| **Index test(s)** | Transtracheal ultrasound, bilateral lung sliding assessment, and diaphragmatic motion analysis | | | | | | |
| **Reference standard(s)** | Capnography, chest auscultation, chest X-ray, or direct laryngoscopy | | | | | | |
| **DTA-SR characteristics** | ***No. of studies*** | 30 | | | | | |
|  | ***No. of participants*** | 2588 | | | | | |
|  | ***Year range*** | 2009-2024 | | | | | |
|  | ***Quality assessment*** | QUADAS-2 | | | | | |
|  | ***Special remark*** |  | | | | | |
| **Diagnostic accuracy** | ***Measure*** | Sensitivity [%] (95% CI):  91.0 (82.0; 95.0) | | | Specificity [%] (95% CI):  98.0 (96.0; 99.0) | | |
|  | ***GRADE*** | Low ⊕⊖⊕⊖ | | | Moderate ⊕⊖⊕⊕ | | |
| **Method. quality**  **(mod AMSTAR2)** | **Overall** | Moderate | | | | | |
| **Further investigations, sub- or sensitivity analyses** | **Index test** | | **Reference standard** | **Studies (Particip.)** | | **Sens. [%], 95% CI** | **Spec. [%], 95% CI** |
|  | Unchanged | | Capnography as the sole reference standard | 12 | | 92 (NA) | 99 (NA) |
|  | Unchanged | | Other standards or composite | 8 | | 90 (NA) | 97 (NA) |
|  | ED Setting | | Unchanged | 7 | | 91 (NA) | 98 (NA) |
|  | ICU Setting | | Unchanged | 13 | | 91 (NA) | 98 (NA) |
| **Conclusions of study authors** | *“POCUS is a highly accurate, rapid, and versatile tool for confirming endotracheal tube placement, with performance metrics that support its use as a strong adjunct to standard methods. Its ability to confirm placement before ventilation, combined with portability and cost-effectiveness, makes it especially valuable as part of a multimodal verification strategy, in resource-limited settings, and in clinical situations where capnography is unreliable.”* | | | | | | |

| **Chou (2015)**[152] – Endotracheal tube placement | | | | | | | |
| --- | --- | --- | --- | --- | --- | --- | --- |
| **Clinical context** | ***Patient population*** | | Adults | | | | |
|  | ***Clinical setting*** | | Emergency department | | | | |
|  | ***Prevalence [%] (CI)*** | | Not documented | | | | |
|  | ***Noteworthy incl. crit.*** | | Cohort design or case–control design with appropriate controls | | | | |
|  | ***Noteworthy excl. crit.*** | | Case reports, comments, reviews, guidelines and animal studies. Studies enrolling pediatric patients were excluded as well | | | | |
| **Index test(s)** | Tracheal ultrasound, lung ultrasound, diaphragm movement | | | | | | |
| **Reference standard(s)** | Capnography, direct laryngoscopy, chest auscultation, chest X-ray | | | | | | |
| **DTA-SR characteristics** | ***No. of studies*** | | 9 | | | | |
|  | ***No. of participants*** | | 665 | | | | |
|  | ***Year range*** | | 2009 - 2014 | | | | |
|  | ***Quality assessment*** | | QUADAS | | | | |
|  | ***Special remark*** | | Authors contribute decreased sensitivity of emergency department ultrasound to the emergent nature of many emergency department intubations | | | | |
| **Diagnostic accuracy** | ***Measure*** | | Sensitivity [%] (95% CI):  88.0 (76.0; 94.0) | | Specificity [%] (95% CI):  98.0 (96.0; 99.0) | | |
|  | ***GRADE*** | | Moderate ⊕⊕⊕⊖ | | High ⊕⊕⊕⊕ | | |
| **Method. quality**  **(mod AMSTAR2)** | **Overall** | | Moderate | | | | |
| **Further investigations, sub- or sensitivity analyses** | **Index test** | **Reference standard** | | **Studies (Particip.)** | | **Sens. [%], 95% CI** | **Spec. [%], 95% CI** |
|  | Ultrasonography overall | capnography, direct laryngoscopy, chest auscultation, chest X-ray | | 12 (1656) | | 93 (86; 96) | 97 (95; 98) |
|  | Tracheal ultrasonography | capnography, direct laryngoscopy, chest auscultation, chest X-ray | | NA | | 92 (84; 97) | 97 (95; 98) |
|  | Resident-performed | capnography, direct laryngoscopy, chest auscultation, chest X-ray | | NA | | 92 (78; 96) | 97 (95; 98) |
|  | Physician performed | capnography, direct laryngoscopy, chest auscultation, chest X-ray | | NA | | 98 (96; 99) | 96 (94; 98) |
|  | Real-time | capnography, direct laryngoscopy, chest auscultation, chest X-ray | | NA | | 94 (86; 98) | 96 (94; 98) |
|  | Static | capnography, direct laryngoscopy, chest auscultation, chest X-ray | | NA | | 91 (10; 98) | 98 (94; 99) |
| **Conclusions of study authors** | *"Current evidence supports that ultrasonography has high diagnostic value for identifying esophageal intubation. With optimal sensitivity and specificity, ultrasonography can be a valuable adjunct in this aspect of airway assessment, especially in situations where capnography may be unreliable.”* | | | | | | |

| **Das (2015)**[153] – Endotracheal tube placement | | | | | | | |
| --- | --- | --- | --- | --- | --- | --- | --- |
| **Clinical context** | ***Patient population*** | Adults | | | | | |
|  | ***Clinical setting*** | Emergency department, elective | | | | | |
|  | ***Prevalence [%] (CI)*** | Not documented | | | | | |
|  | ***Noteworthy incl. crit.*** | Transtracheal ultrasound, capnography as reference standard | | | | | |
|  | ***Noteworthy excl. crit.*** | Pediatric studies, cadaver studies | | | | | |
| **Index test(s)** | Transtracheal ultrasound, emergency intubations | | | | | | |
| **Reference standard(s)** | Capnography | | | | | | |
| **DTA-SR characteristics** | ***No. of studies*** | 8 | | | | | |
|  | ***No. of participants*** | 713 | | | | | |
|  | ***Year range*** | 2011 - 2014 | | | | | |
|  | ***Quality assessment*** | QUADAS-2 (mostly low risk of bias) | | | | | |
|  | ***Special remark*** | Only included transtracheal ultrasound with capnography as reference standard | | | | | |
| **Diagnostic accuracy** | ***Measure*** | Sensitivity [%] (95% CI):  98.0 (97.0; 99.0) | | | Specificity [%] (95% CI):  94.0 (86.0; 98.0) | | |
|  | ***GRADE*** | High ⊕⊕⊕⊕ | | | Moderate ⊕⊕⊕⊖ | | |
| **Method. quality**  **(mod AMSTAR2)** | **Overall** | Moderate | | | | | |
| **Further investigations, sub- or sensitivity analyses** | **Index test** | | **Reference standard** | **Studies (Particip.)** | | **Sens. [%], 95% CI** | **Spec. [%], 95% CI** |
|  | Overall | | Capnography | 11 (969) | | 98 (97; 99) | 98 (95; 99) |
|  | Low risk of bias | | Capnography | 7 (NA) | | 99 (98; 100) | 97 (94; 99) |
|  |  | |  |  | |  |  |
|  |  | |  |  | |  |  |
| **Conclusions of study authors** | *“…transtracheal ultrasound is a novel technique with an acceptable degree of accuracy to confirm tracheal placement of the ETT in reasonably less time and without ventilation.”* | | | | | | |

| **Gottlieb (2018)**[154] – Endotracheal tube placement | | | | | | | |
| --- | --- | --- | --- | --- | --- | --- | --- |
| **Clinical context** | ***Patient population*** | Adults | | | | | |
|  | ***Clinical setting*** | Emergency department, operation room | | | | | |
|  | ***Prevalence [%] (CI)*** | 91.3 (89.7; 92.9) | | | | | |
|  | ***Noteworthy incl. crit.*** | Prospective studies or randomized controlled trials | | | | | |
|  | ***Noteworthy excl. crit.*** | Retrospective studies, case series, pediatric studies, cadaver studies | | | | | |
| **Index test(s)** | Transtracheal ultrasound, emergency department performed | | | | | | |
| **Reference standard(s)** | Direct visualization, capnography, auscultation, aspiration, oxygen saturation monitoring | | | | | | |
| **DTA-SR characteristics** | ***No. of studies*** | 13 | | | | | |
|  | ***No. of participants*** | 1152 | | | | | |
|  | ***Year range*** | 2009 – 2017 | | | | | |
|  | ***Quality assessment*** | QUADAS-2, overall low risk of bias with unclear risk of bias mostly in the domain of patient selection | | | | | |
|  | ***Special remark*** | Includes emergency department, operation room patients | | | | | |
| **Diagnostic accuracy** | ***Measure*** | Sensitivity [%] (95% CI):  98.7 (97.6; 99.3) | | | Specificity [%] (95% CI):  96.0 (89.8; 98.5) | | |
|  | ***GRADE*** | High ⊕⊕⊕⊕ | | | Moderate ⊕⊖⊕⊕ | | |
| **Method. quality**  **(mod AMSTAR2)** | **Overall** | Moderate | | | | | |
| **Further investigations, sub- or sensitivity analyses** | **Index test** | | **Reference standard** | **Studies (Particip.)** | | **Sens. [%], 95% CI** | **Spec. [%], 95% CI** |
|  | Overall | | Visualization, capnography, auscultation, aspiration, | 17 (1595) | | 99 (98; 99) | 97 (92; 99) |
|  | ED patients | | Visualization, capnography, auscultation, aspiration, | 12 (1148) | | 99 (98; 99) | 94 (87; 97) |
|  | Non-ED patients | | Visualization, capnography, auscultation, aspiration, | 5 (449) | | 99 (95; 100) | 100 (58; 100) |
|  | Attending physicians | | Visualization, capnography, auscultation, aspiration, | 5 (478) | | 99 (94; 100) | 98 (84; 100) |
|  | Resident physicians | | Visualization, capnography, auscultation, aspiration, | 9 (878) | | 99 (98; 99) | 94 (86; 97) |
|  | Linear transducer | | Visualization, capnography, auscultation, aspiration, | 12 (1158) | | 99 (97; 99) | 99 (91; 100) |
|  | Curvilinear transducer | | Visualization, capnography, auscultation, aspiration, | 5 (437) | | 99 (98; 100) | 93 (80; 98) |
|  | Static technique | | Visualization, capnography, auscultation, aspiration, | 9 (891) | | 98 (97; 99) | 94 (86; 97) |
|  | Dynamic technique | | Visualization, capnography, auscultation, aspiration, | 8 (603) | | 99 (98; 100) | 99 (84; 100) |
| **Conclusions of study authors** | *“Overall, our study demonstrated that transtracheal sonography has an acceptable degree of sensitivity and specificity for the confirmation of endotracheal intubation. Ultrasonography is a valuable adjunct and should be considered when quantitative capnography is unavailable or unreliable.”* | | | | | | |

| **Sahu (2020)**[155] – Endotracheal tube placement | | | | | | | |
| --- | --- | --- | --- | --- | --- | --- | --- |
| **Clinical context** | ***Patient population*** | Adults | | | | | |
|  | ***Clinical setting*** | Emergency department, operation room | | | | | |
|  | ***Prevalence [%] (CI)*** | Not documented | | | | | |
|  | ***Noteworthy incl. crit.*** | POCUS transtracheal or lung | | | | | |
|  | ***Noteworthy excl. crit.*** | Retrospective studies, case series, pediatric studies, cadaver studies | | | | | |
| **Index test(s)** | POCUS, any sign, emergency department-performed | | | | | | |
| **Reference standard(s)** | Capnography or fiberoptic bronchoscopy | | | | | | |
| **DTA-SR characteristics** | ***No. of studies*** | 20 | | | | | |
|  | ***No. of participants*** | 2534 | | | | | |
|  | ***Year range*** | 2007 - 2019 | | | | | |
|  | ***Quality assessment*** | QUADAS-2 (overall low or unclear risk of bias) | | | | | |
|  | ***Special remark*** | No statistically significant differences in accuracy of POCUS with respect to operator specialty, study location | | | | | |
| **Diagnostic accuracy** | ***Measure*** | Sensitivity [%] (95% CI):  98.0 (97.0; 99.0) | | | Specificity [%] (95% CI):  95.0 (90.0; 100) | | |
|  | ***GRADE*** | High ⊕⊕⊕⊕ | | | High ⊕⊕⊕⊕ | | |
| **Method. quality**  **(mod AMSTAR2)** | **Overall** | Moderate | | | | | |
| **Further investigations, sub- or sensitivity analyses** | **Index test** | | **Reference standard** | **Studies (Particip.)** | | **Sens. [%], 95% CI** | **Spec. [%], 95% CI** |
|  | Double tract sign or similar | | Capnography or fiberoptic bronchoscopy | 20 (NA) | | 98 (97; 99) | 95 (88; 98) |
|  | Snwo-storm sign | | Capnography or fiberoptic bronchoscopy | 2 (NA) | | 97 (81; 99) | 100 (0; 100) |
|  | Bullet sign | | Capnography or fiberoptic bronchoscopy | 2 (NA) | | 98 (93; 99) | 100 (0; 100) |
|  | Lung sliding sign | | Capnography or fiberoptic bronchoscopy | 3 (NA) | | 97 (93; 99) | 96 (56; 99) |
|  | All signs, cardiac arrest | | Capnography or fiberoptic bronchoscopy | 5 (NA) | | 99 (98; 100) | 84 (67; 100) |
|  | All signs, non-cardiac arrest | | Capnography or fiberoptic bronchoscopy | 13 (NA) | | 98 (97; 99) | 97 (94; 100) |
|  | Location (ED) | | Capnography or fiberoptic bronchoscopy | 18 | | 98 (97;99) | 93 (88;99) |
|  | Location (Non-ED) | | Capnography or fiberoptic bronchoscopy | 12 | | 98 (97;100) | 98 (95;100) |
|  | Sonographer specialty (Non-Emergency medical) | | Capnography or fiberoptic bronchoscopy | 11 | | 98 (97;100) | 98 (94;100) |
|  | USG sign (Other USG signs) | | Capnography or fiberoptic bronchoscopy | 10 | | 98 (96;99) | 98 (94;100) |
|  | Transducer (Linear) | | Capnography or fiberoptic bronchoscopy | 23 | | 98 (97;99) | 96 (92;100) |
|  | Transducer (Curvilinear) | | Capnography or fiberoptic bronchoscopy | 7 | | 99 (99;100) | 99 (92;100) |
|  | Risk of bias (Low risk) | | Capnography or fiberoptic bronchoscopy | 7 | | 99 (98;100) | 95 (89;100) |
|  | Risk of bias (At risk of bias) | | Capnography or fiberoptic bronchoscopy | 23 | | 98 (97;99) | 97 (93;100) |
|  | Technique (Dynamic) | | Capnography or fiberoptic bronchoscopy | 19 | | 98 (97;99) | 97 (94;100) |
|  | Technique (Static) | | Capnography or fiberoptic bronchoscopy | 11 | | 98 (97;99) | 93 (85;100) |
| **Conclusions of study authors** | *“Ultrasonography is a useful adjunct to confirm endotracheal intubation with excellent sensitivity and specificity. It should be considered when capnography is not available or unreliable. The incorporation of USG in resuscitation will not only help in dynamic assessment of airway, but also it will serve as a time-saving step.”* | | | | | | |

| **Soni (2025)**[156] – Endotracheal tube placement | | | | | | | |
| --- | --- | --- | --- | --- | --- | --- | --- |
| **Clinical context** | ***Patient population*** | Adults | | | | | |
|  | ***Clinical setting*** | Pre- and intraoperative | | | | | |
|  | ***Prevalence [%] (CI)*** | Not documented | | | | | |
|  | ***Noteworthy incl. crit.*** | - | | | | | |
|  | ***Noteworthy excl. crit.*** | Case series, pediatric populations | | | | | |
| **Index test(s)** | B-mode ultrasound, distance from skin to epiglottis | | | | | | |
| **Reference standard(s)** | Cormack Lehane score, > 2 attempts, > 10 minutes duration | | | | | | |
| **DTA-SR characteristics** | ***No. of studies*** | 8 | | | | | |
|  | ***No. of participants*** | 2112 | | | | | |
|  | ***Year range*** | Not documented | | | | | |
|  | ***Quality assessment*** | QUADAS-2 | | | | | |
|  | ***Special remark*** | Rated certainty of evidence with GRADE, judged moderate to high | | | | | |
| **Diagnostic accuracy** | ***Measure*** | Sensitivity [%] (95% CI):  78.0 (60.0; 89.0) | | | Specificity [%] (95% CI):  81.0 (63.0; 91.0) | | |
|  | ***GRADE*** | Low ⊕⊕⊖⊖ | | | Low ⊕⊕⊖⊖ | | |
| **Method. quality**  **(mod AMSTAR2)** | **Overall** | Moderate | | | | | |
| **Further investigations, sub- or sensitivity analyses** | **Index test** | | **Reference standard** | **Studies (Particip.)** | | **Sens. [%], 95% CI** | **Spec. [%], 95% CI** |
|  | B-mode ultrasound, distans from skin to hyoid bone | | Cormack Lehane score, > 2 attempts, > 10 minutes duration | 3 (1493 | | 80 (74; 85) | 86 (74; 91) |
|  | B-mode ultrasound, distance from skin to vocal cords | | Cormack Lehane score, > 2 attempts, > 10 minutes duration | 3 (300) | | 69 (52; 91) | 80 (73; 86) |
|  | Hyomental distans ratio of 1 | | Cormack Lehane score, > 2 attempts, > 10 minutes duration | 3 (345) | | 80 (63; 91) | 62 (55; 70) |
|  | Pre-E/E-VC ratio | | Cormack Lehane score, > 2 attempts, > 10 minutes duration | 4 (469) | | 56 (6; 97) | 70 (31; 92) |
| **Conclusions of study authors** | *“In summary, our study supports the potential for the incorporation of POCUS as part of the standard of care in airway assessment and management.”* | | | | | | |

| **Yang (2022)**[157] – Endotracheal tube placement | | | | | | | |
| --- | --- | --- | --- | --- | --- | --- | --- |
| **Clinical context** | ***Patient population*** | Adults and children | | | | | |
|  | ***Clinical setting*** | Emergency department, intensive care unit, anesthesiology | | | | | |
|  | ***Prevalence [%] (CI)*** | 80.4 (77.3; 83.5) | | | | | |
|  | ***Noteworthy incl. crit.*** | Sufficient data to calculate 2x2 table, ethics committee approval | | | | | |
|  | ***Noteworthy excl. crit.*** | Case series witch <10 cases, overlapping data | | | | | |
| **Index test(s)** | LUS | | | | | | |
| **Reference standard(s)** | Fiberoptic bronchoscopy, chest radiography | | | | | | |
| **DTA-SR characteristics** | ***No. of studies*** | 9 | | | | | |
|  | ***No. of participants*** | 617 | | | | | |
|  | ***Year range*** | 2010 - 2019 | | | | | |
|  | ***Quality assessment*** | QUADAS-2, overall low risk of bias | | | | | |
|  | ***Special remark*** | Authors report no heterogeneity even though I2-, Q-Test, and forest plots show significant; no 95% CI for subgroup analysis presented; supplementary material incomplete | | | | | |
| **Diagnostic accuracy** | ***Measure*** | Sensitivity [%] (95% CI):  93.0 (87.0; 96.0) | | | Specificity [%] (95% CI):  75.0 (57.0; 87.0) | | |
|  | ***GRADE*** | Moderate ⊕⊖⊕⊕ | | | Low ⊕⊖⊕⊖ | | |
| **Method. quality**  **(mod AMSTAR2)** | **Overall** | Moderate | | | | | |
| **Further investigations, sub- or sensitivity analyses** | **Index test** | | **Reference standard** | **Studies (Particip.)** | | **Sens. [%], 95% CI** | **Spec. [%], 95% CI** |
|  | LUS adults | | Fiberoptic bronchoscopy, chest x-ray | 6 | | 88.4 (NA) | 74.4 (NA) |
|  | LUS children | | Fiberoptic bronchoscopy, chest x-ray | 3 | | 94.5 (NA) | 60.0 (NA) |
|  |  | |  |  | |  |  |
|  |  | |  |  | |  |  |
| **Conclusions of study authors** | *“This study suggests that lung ultrasound has high accuracy in confirming endotracheal tube placement.”* | | | | | | |

| **Benavides-Zora (2023)**[158] – Predicting difficult intubation | | | | | | | |
| --- | --- | --- | --- | --- | --- | --- | --- |
| **Clinical context** | ***Patient population*** | Adults | | | | | |
|  | ***Clinical setting*** | preoperative | | | | | |
|  | ***Prevalence [%] (CI)*** | 12.0 (9.3; 14.7) | | | | | |
|  | ***Noteworthy incl. crit.*** | Observational studies | | | | | |
|  | ***Noteworthy excl. crit.*** | Anatomic airway abnormalities, obstetric populations, alternative imaging methods | | | | | |
| **Index test(s)** | Hyomental-distance ratio | | | | | | |
| **Reference standard(s)** | Direct laryngoscopy (Cormack-Lehane score ≥ 3) | | | | | | |
| **DTA-SR characteristics** | ***No. of studies*** | 4 | | | | | |
|  | ***No. of participants*** | 544 | | | | | |
|  | ***Year range*** | 2016 - 2018 | | | | | |
|  | ***Quality assessment*** | QUADAS-2, GRADEpro; most studies with low risk of bias and good quality | | | | | |
|  | ***Special remark*** | Obese patients were excluded in 3 studies; large variability in cutoff points | | | | | |
| **Diagnostic accuracy** | ***Measure*** | Sensitivity [%] (95% CI):  61.0 (45.0; 75.0) | | | Specificity [%] (95% CI):  88.0 (77.0; 94.0) | | |
|  | ***GRADE*** | Very low ⊕⊖⊖⊖ | | | Very low ⊕⊖⊖⊖ | | |
| **Method. quality**  **(mod AMSTAR2)** | **Overall** | Moderate | | | | | |
| **Further investigations, sub- or sensitivity analyses** | **Index test** | | **Reference standard** | **Studies (Particip.)** | | **Sens. [%], 95% CI** | **Spec. [%], 95% CI** |
|  | Skin-to-Epiglottis Distance | | Direct laryngoscopy | 2 (375) | | 75 (55; 88) | 86 (74; 93) |
|  | Ratio of preepiglottic distance to the epiglottic distance at the midpoint of the vocal chords | | Direct laryngoscopy | 2 (320) | | 82 (68; 91) | 83 (78; 87) |
|  |  | |  |  | |  |  |
|  |  | |  |  | |  |  |
| **Conclusions of study authors** | *“The 3 commonly used airway POCUS measures have high variability in reported studies. Hence, although pooled data in the authors’ review suggested that POCUS may have higher sensitivity and specificity than clinical measures in predicting difficult laryngoscopy, future data may change their confidence in these conclusions.”* | | | | | | |

| **Bhargava (2023)**[159] – Predicting difficult intubation | | | | | | | |
| --- | --- | --- | --- | --- | --- | --- | --- |
| **Clinical context** | ***Patient population*** | Adults | | | | | |
|  | ***Clinical setting*** | Elective surgery | | | | | |
|  | ***Prevalence [%] (CI)*** | 9 (NA) | | | | | |
|  | ***Noteworthy incl. crit.*** | Retrospective or prospective studies, | | | | | |
|  | ***Noteworthy excl. crit.*** | Case series < 10 cases, | | | | | |
| **Index test(s)** | Ultrasound, anterior neck soft tissue thickness domain | | | | | | |
| **Reference standard(s)** | Direct laryngoscopy (Cormack-Lehane score ≥ 3) or difficult intubation | | | | | | |
| **DTA-SR characteristics** | ***No. of studies*** | 23 | | | | | |
|  | ***No. of participants*** | Not documented | | | | | |
|  | ***Year range*** | 2014 - 2021 | | | | | |
|  | ***Quality assessment*** | QUADAS-2 (overall low risk of bias) | | | | | |
|  | ***Special remark*** | Most studies excluded morbidly obese patients; one study evaluated pregnant patients. Studies were grouped into three domains before metaanalysis | | | | | |
| **Diagnostic accuracy** | ***Measure*** | Sensitivity [%] (95% CI):  76.0 (71.0; 81.0) | | | Specificity [%] (95% CI):  77.0 (72.0; 82.0) | | |
|  | ***GRADE*** | Low ⊕⊖⊖⊕ | | | Low ⊕⊖⊖⊕ | | |
| **Method. quality**  **(mod AMSTAR2)** | **Overall** | Moderate | | | | | |
| **Further investigations, sub- or sensitivity analyses** | **Index test** | | **Reference standard** | **Studies (Particip.)** | | **Sens. [%], 95% CI** | **Spec. [%], 95% CI** |
|  | Anatomical position domain | | Direct laryngoscopy | 10 (NA) | | 74 (61; 84) | 86 (78; 91) |
|  | Oral space domains | | Direct laryngoscopy | 10 (NA) | | 53 (36; 69) | 77 (67; 85) |
|  |  | |  |  | |  |  |
|  |  | |  |  | |  |  |
| **Conclusions of study authors** | *“Ultrasound measurements in three domains may serve as predictors for difficult laryngoscopy. The Anterior-posterior diameter, which includes sonographic assessment of hyomental distance, may be more accurate than other domains.”* | | | | | | |

| **Carsetti (2022)**[160] – Predicting difficult intubation | | | | | | | |
| --- | --- | --- | --- | --- | --- | --- | --- |
| **Clinical context** | ***Patient population*** | Adults | | | | | |
|  | ***Clinical setting*** | Elective surgery | | | | | |
|  | ***Prevalence [%] (CI)*** | 15.4 (13.9; 16.9) | | | | | |
|  | ***Noteworthy incl. crit.*** | Any ultrasound of neck soft tissue, adults, elective surgery under general anesthesia, observational trials | | | | | |
|  | ***Noteworthy excl. crit.*** | Anatomical abnormalities, previous difficult intubation or expected difficult laryngoscopy | | | | | |
| **Index test(s)** | Upper airway ultrasound, distance from skin to epiglottis | | | | | | |
| **Reference standard(s)** | Direct laryngoscopy (Cormack-Lehane score ≥ 3) | | | | | | |
| **DTA-SR characteristics** | ***No. of studies*** | 15 | | | | | |
|  | ***No. of participants*** | 2097 | | | | | |
|  | ***Year range*** | 2014 – 2020 | | | | | |
|  | ***Quality assessment*** | QUADAS-2, GRADE | | | | | |
|  | ***Special remark*** | Obese patients were excluded in some studies; publication bias investigation planned but not reported | | | | | |
| **Diagnostic accuracy** | ***Measure*** | Sensitivity [%] (95% CI):  82.0 (74.0; 87.0) | | | Specificity [%] (95% CI):  79.0 (70.0; 86.0) | | |
|  | ***GRADE*** | Very low ⊕⊖⊖⊖ | | | Very low ⊕⊖⊖⊖ | | |
| **Method. quality**  **(mod AMSTAR2)** | **Overall** | Moderate | | | | | |
| **Further investigations, sub- or sensitivity analyses** | **Index test** | | **Reference standard** | **Studies (Particip.)** | | **Sens. [%], 95% CI** | **Spec. [%], 95% CI** |
|  | Distance from skin to hyoid bone | | Direct laryngoscopy | 7 (1064) | | 71 (58; 82) | 71 (57; 82) |
|  | Distance from skin to vocal cords | | Direct laryngoscopy | 6 (824) | | 75 (62; 84) | 72 (45; 89) |
|  | Distance from the epiglottis to the midpoint of the distance between the vocal cords | | Direct laryngoscopy | 5 (574) | | 65 (22; 93) | 68 (43; 85) |
|  |  | |  |  | |  |  |
| **Conclusions of study authors** | *“[…] our study demonstrates that upper airway ultrasound index tests are significantly different between patients with easy versus difficult direct laryngoscopy, and the distance from skin to epiglottis is the most studied index test in literature to predict difficult direct laryngoscopy.”* | | | | | | |

| **Ji (2017)**[161] – Predicting difficult intubation | | | | | | | |
| --- | --- | --- | --- | --- | --- | --- | --- |
| **Clinical context** | ***Patient population*** | Adults | | | | | |
|  | ***Clinical setting*** | Operation room | | | | | |
|  | ***Prevalence [%] (CI)*** | 8.3 (7.4; 9.2) | | | | | |
|  | ***Noteworthy incl. crit.*** | CT, MRI, X-ray, ultrasound, modified Mallampati score for DTA, adults, surgery under general anesthesia, not pregnant, ASA I-III | | | | | |
|  | ***Noteworthy excl. crit.*** | Insufficient data for 2x2 table | | | | | |
| **Index test(s)** | Ultrasound, multiple signs | | | | | | |
| **Reference standard(s)** | Direct laryngoscopy (Cormack-Lehane score ≥ 3) | | | | | | |
| **DTA-SR characteristics** | ***No. of studies*** | 8 | | | | | |
|  | ***No. of participants*** | 3485 | | | | | |
|  | ***Year range*** | 2014 - 2017 | | | | | |
|  | ***Quality assessment*** | QUADAS-2; overall substantial risk of bias | | | | | |
|  | ***Special remark*** | Sample size might be the major source of heterogeneity | | | | | |
| **Diagnostic accuracy** | ***Measure*** | Sensitivity [%] (95% CI):  69.0 (63.0; 74.0) | | | Specificity [%] (95% CI):  84.0 (82.0; 85.0) | | |
|  | ***GRADE*** | Very low ⊕⊖⊖⊖ | | | Low ⊕⊖⊖⊕ | | |
| **Method. quality**  **(mod AMSTAR2)** | **Overall** | Moderate | | | | | |
| **Further investigations, sub- or sensitivity analyses** | **Index test** | | **Reference standard** | **Studies (Particip.)** | | **Sens. [%], 95% CI** | **Spec. [%], 95% CI** |
|  |  | |  |  | |  |  |
|  |  | |  |  | |  |  |
|  |  | |  |  | |  |  |
|  |  | |  |  | |  |  |
| **Conclusions of study authors** | *“…provided evidence that CT, X-ray and ultrasound could be regarded as effective methods and significantly improve the sensitivity and specificity in terms of predicting difficult airway. The diagnostic value of US is similar to that of CT and X-ray but much better than that of Modified Mallampati Score*.” | | | | | | |

###### Central venous catheter placement

| **Ablordeppey (2017)**[162] – Central venous catheter placement | | | | | | | |
| --- | --- | --- | --- | --- | --- | --- | --- |
| **Clinical context** | ***Patient population*** | Adults | | | | | |
|  | ***Clinical setting*** | Emergency department, intensive care unit, ward | | | | | |
|  | ***Prevalence [%] (CI)*** | 17.6 (15.6; 19.6) | | | | | |
|  | ***Noteworthy incl. crit.*** | Adults, direct comparison of US to chest radiography, internal jugular vein or subclavian vein | | | | | |
|  | ***Noteworthy excl. crit.*** | Inadequate data for 2x2 tables | | | | | |
| **Index test(s)** | Cardiac and vascular ultrasound and/or CEUS | | | | | | |
| **Reference standard(s)** | Chest radiography | | | | | | |
| **DTA-SR characteristics** | ***No. of studies*** | 13 | | | | | |
|  | ***No. of participants*** | 1469 | | | | | |
|  | ***Year range*** | 2001 - 2015 | | | | | |
|  | ***Quality assessment*** | QUADAS-2 (no study fulfilled all QUADAS-2 criteria for low risk of bias) | | | | | |
|  | ***Special remark*** | Secondary outcome: DTA for pneumothorax, no meta-analysis for this outcome | | | | | |
| **Diagnostic accuracy** | ***Measure*** | Sensitivity [%] (95% CI):  82.0 (77.0; 86.0) | | | Specificity [%] (95% CI):  98.0 (97.0; 99.0) | | |
|  | ***GRADE*** | Moderate ⊕⊖⊕⊕ | | | Moderate ⊕⊖⊕⊕ | | |
| **Method. quality**  **(mod AMSTAR2)** | **Overall** | Moderate | | | | | |
| **Further investigations, sub- or sensitivity analyses** | **Index test** | | **Reference standard** | **Studies (Particip.)** | | **Sens. [%], 95% CI** | **Spec. [%], 95% CI** |
|  | Experienced | | Chest radiography | 6 (NA) | | 69 (58; 79) | 98 (96; 99) |
|  | Inexperienced | | Chest radiography | 3 (NA) | | 68 (45; 86) | 99 (97; 100) |
|  | Both experienced and inexperienced | | Chest radiography | 4 (NA) | | 89 (82; 94) | 98 (96; 99) |
|  | Contrast | | Chest radiography | NA (NA) | | 79 (64; 90) | 99 (98; 100) |
|  | No contrast | | Chest radiography | NA (NA) | | 80 (73; 85) | 97 (95; 99) |
|  | Cardiac and vascular ultrasound | | Chest radiography | 5 (NA) | | 82 (76; 87) | 95 (92; 97) |
|  | Cardiac ultrasound only | | Chest radiography | 8 (NA) | | 64 (43; 82) | 100 (99; 100) |
| **Conclusions of study authors** | *“When a central venous catheter malposition exists, bedside ultrasound will identify four out of every five.”* | | | | | | |

| **Bou Chebl (2017)**[163] – Central venous catheter placement | | | | | | | |
| --- | --- | --- | --- | --- | --- | --- | --- |
| **Clinical context** | ***Patient population*** | Adults | | | | | |
|  | ***Clinical setting*** | Emergency department, intensive care unit | | | | | |
|  | ***Prevalence [%] (CI)*** | 7.1 (5.0; 9.2) | | | | | |
|  | ***Noteworthy incl. crit.*** | Directly compare ultrasound to chest radiography, internal jugular vein or subclavian vein | | | | | |
|  | ***Noteworthy excl. crit.*** | - | | | | | |
| **Index test(s)** | CEUS | | | | | | |
| **Reference standard(s)** | Chest radiography | | | | | | |
| **DTA-SR characteristics** | ***No. of studies*** | 5 | | | | | |
|  | ***No. of participants*** | 572 | | | | | |
|  | ***Year range*** | 2010 - 2016 | | | | | |
|  | ***Quality assessment*** | QUADAS-2, possible risk of bias in the domain of patient selection | | | | | |
|  | ***Special remark*** | Consistent standard ultrasound technique and study design across all articles included  2/5 studies excluded populations with indwelling mechanical devices and difficult 4-chameber cardiac views | | | | | |
| **Diagnostic accuracy** | ***Measure*** | Sensitivity [%] (95% CI):  72.0 (44.0; 91.0) | | | Specificity [%] (95% CI):  100 (99.0; 100) | | |
|  | ***GRADE*** | Low ⊖⊕⊕⊖ | | | Moderate ⊖⊕⊕⊕ | | |
| **Method. quality**  **(mod AMSTAR2)** | **Overall** | Low | | | | | |
| **Further investigations, sub- or sensitivity analyses** | **Index test** | | **Reference standard** | **Studies (Particip.)** | | **Sens. [%], 95% CI** | **Spec. [%], 95% CI** |
|  |  | |  |  | |  |  |
|  |  | |  |  | |  |  |
|  |  | |  |  | |  |  |
|  |  | |  |  | |  |  |
| **Conclusions of study authors** | *“In the setting of adult internal jugular or subclavian central venous catheter placement, compared with chest radiography, postprocedural contrast-enhanced ultrasound imaging is a safe, efficient, and highly specific confirmatory test for catheter tip location.”* | | | | | | |

| **Smit (2018)**[164] – Central venous catheter placement | | | | | | | |
| --- | --- | --- | --- | --- | --- | --- | --- |
| **Clinical context** | ***Patient population*** | Mixed, mostly adults | | | | | |
|  | ***Clinical setting*** | Emergency department, intensive care unit, operation room, nephrology department | | | | | |
|  | ***Prevalence [%] (CI)*** | - | | | | | |
|  | ***Noteworthy incl. crit.*** | Central venous catheter in subclavian or internal jugular vein | | | | | |
|  | ***Noteworthy excl. crit.*** | Case reports/series, index test TEE, no reference standard, language non-english/spanish/portuguese | | | | | |
| **Index test(s)** | Vascular US, TTE and/or CEUS | | | | | | |
| **Reference standard(s)** | Chest radiography, TEE +/- intrafluoroscopy | | | | | | |
| **DTA-SR characteristics** | ***No. of studies*** | 25 | | | | | |
|  | ***No. of participants*** | 2548 | | | | | |
|  | ***Year range*** | 2000 - 2016 | | | | | |
|  | ***Quality assessment*** | QUADAS-2; no study scored low in all domains of the bias assessment | | | | | |
|  | ***Special remark*** | Malposition wasn’t defined equally in all studies  For pneumothorax prevalence is reported instead of pooled sn/sp | | | | | |
| **Diagnostic accuracy** | ***Measure*** | Sensitivity [%] (95% CI):  68.2 (54.4; 79.4) | | | Specificity [%] (95% CI):  98.9 (97.8; 99.4) | | |
|  | ***GRADE*** | Low ⊕⊖⊕⊖ | | | Moderate ⊕⊖⊕⊕ | | |
| **Method. quality**  **(mod AMSTAR2)** | **Overall** | Moderate | | | | | |
| **Further investigations, sub- or sensitivity analyses** | **Index test** | | **Reference standard** | **Studies (Particip.)** | | **Sens. [%], 95% CI** | **Spec. [%], 95% CI** |
|  | TTE and CEUS, total | | Chest Radiography, TEE +/- intrafluoroscopy | 12 (NA) | | 65 (51; 78) | 99 (97; 99) |
|  | TTE and CEUS, intraatrial malposition | | Chest Radiography, TEE +/- intrafluoroscopy | 12 (NA) | | 74 (57; 85) | 97 (95; 99) |
|  | TTE and CEUS, extraatrial malposition | | Chest Radiography, TEE +/- intrafluoroscopy | 12 (NA) | | 56 (21; 86) | 100 (98; 100) |
|  |  | |  |  | |  |  |
| **Conclusions of study authors** | *„…ultrasound is an accurate and feasible diagnostic modality to detect central venous catheter malposition and iatrogenic pneumothorax.“* | | | | | | |

| **Zhu (2026**)[165] – Central venous catheter placement | | | | | | | |
| --- | --- | --- | --- | --- | --- | --- | --- |
| **Clinical context** | ***Patient population*** | Adult population with planned insertion of central venous catheter | | | | | |
|  | ***Clinical setting*** | Emergency department | | | | | |
|  | ***Prevalence [%] (CI)*** |  | | | | | |
|  | ***Noteworthy incl. crit.*** | Prospective or retrospective observational studies and randomized controlled trials | | | | | |
|  | ***Noteworthy excl. crit.*** | - | | | | | |
| **Index test(s)** | Ultrasound (vascular, cardiac, contrast-enhanced, and/or lung ultrasound) for malplacement | | | | | | |
| **Reference standard(s)** | Chest X-ray or CT | | | | | | |
| **DTA-SR characteristics** | ***No. of studies*** | 8 | | | | | |
|  | ***No. of participants*** | 801 | | | | | |
|  | ***Year range*** | 2013 - 2021 | | | | | |
|  | ***Quality assessment*** | QUADAS-2 | | | | | |
|  | ***Special remark*** |  | | | | | |
| **Diagnostic accuracy** | ***Measure*** | Sensitivity [%] (95% CI):  81.0 (50.0; 95.0) | | | Specificity [%] (95% CI):  99.0 (97.0; 99.0) | | |
|  | ***GRADE*** | Moderate ⊕⊕⊕⊖ | | | Moderate ⊕⊖⊕⊕ | | |
| **Method. Quality**  **(mod AMSTAR2)** | **Overall** | Moderate | | | | | |
| **Further investigations, sub- or sensitivity analyses** | **Index test** | | **Reference standard** | **Studies (Particip.)** | | **Sens. [%], 95% CI** | **Spec. [%], 95% CI** |
|  | Ultrasound for pneumothorax detection | | CT/Chest xray |  | | 97-100 (no Meta-analysis) | 25-100 (no meta-analysis) |
|  |  | |  |  | |  |  |
|  |  | |  |  | |  |  |
|  |  | |  |  | |  |  |
| **Conclusions of study authors** | *“In this diagnostic test accuracy meta- analysis of eight prospective studies (801 CVC insertions), POCUS showed high specificity (0.99) and overall discrimination ability (AUC 0.99) for detecting post- procedural CVC malposition. The large, pooled PLR and DOR support strong rule-in performance. Sensitivity was 0.81 with substantial s, and the pooled NLR of 0.19 suggests that a negative examination reduces—yet does not eliminate—the prob- ability of malposition.”* | | | | | | |

###### Gastric tube placement

| **Lin (2017)**[166] – Nasogastric tube placement | | | | | | | |
| --- | --- | --- | --- | --- | --- | --- | --- |
| **Clinical context** | ***Patient population*** | Adults | | | | | |
|  | ***Clinical setting*** | Prehospital, emergency department intensive care unit | | | | | |
|  | ***Prevalence [%] (CI)*** | 91.9 (89.3; 94.5) | | | | | |
|  | ***Noteworthy incl. crit.*** | Reference standard X-ray | | | | | |
|  | ***Noteworthy excl. crit.*** | Uncontrolled reports, studies on ultrasound-guided nasogastric tube placement | | | | | |
| **Index test(s)** | Ultrasound of stomach, neck, and esophagus, direct visualization or saline/air injection | | | | | | |
| **Reference standard(s)** | Chest or abdominal radiography | | | | | | |
| **DTA-SR characteristics** | ***No. of studies*** | 5 | | | | | |
|  | ***No. of participants*** | 420 | | | | | |
|  | ***Year range*** | 2012 - 2014 | | | | | |
|  | ***Quality assessment*** | QUADAS-2, overall good quality | | | | | |
|  | ***Special remark*** | Meta-regression and subgroup analysis were planned but not possible | | | | | |
| **Diagnostic accuracy** | ***Measure*** | Sensitivity [%] (95% CI):  93.0 (87.0; 97.0) | | | Specificity [%] (95% CI):  97.0 (23.0; 100.0) | | |
|  | ***GRADE*** | High ⊕⊕⊕⊕ | | | Low ⊕⊖⊕⊖ | | |
| **Method. quality**  **(mod AMSTAR2)** | **Overall** | Moderate | | | | | |
| **Further investigations, sub- or sensitivity analyses** | **Index test** | | **Reference standard** | **Studies (Particip.)** | | **Sens. [%], 95% CI** | **Spec. [%], 95% CI** |
|  |  | |  |  | |  |  |
|  |  | |  |  | |  |  |
|  |  | |  |  | |  |  |
|  |  | |  |  | |  |  |
| **Conclusions of study authors** | *„Our findings partly support the use of ultrasound to confirm nasogastric tube placement.“* | | | | | | |

| **Peng (2022)**[176] – Nasogastric tube placement | | | | | | | |
| --- | --- | --- | --- | --- | --- | --- | --- |
| **Clinical context** | ***Patient population*** | Adults and children | | | | | |
|  | ***Clinical setting*** | Emergency department | | | | | |
|  | ***Prevalence [%] (CI)*** | 95.2 (91.9; 98.4) | | | | | |
|  | ***Noteworthy incl. crit.*** | Reference standard X-ray | | | | | |
|  | ***Noteworthy excl. crit.*** | Uncontrolled reports, studies on ultrasound-guided NGT placement | | | | | |
| **Index test(s)** | Ultrasound of stomach, neck, and esophagus, direct visualization or saline/air injection | | | | | | |
| **Reference standard(s)** | Chest or abdominal radiography | | | | | | |
| **DTA-SR characteristics** | ***No. of studies*** | 3 | | | | | |
|  | ***No. of participants*** | 168 | | | | | |
|  | ***Year range*** | 2012 - 2021 | | | | | |
|  | ***Quality assessment*** | QUADAS-2, overall good quality | | | | | |
|  | ***Special remark*** | Authors planned to explore factors that affected, but were unable to because the required information was unavailable or limited | | | | | |
| **Diagnostic accuracy** | ***Measure*** | Sensitivity [%] (95% CI):  93.0 (88.0; 97.0) | | | Specificity [%] (95% CI):  88.0 (47.0; 100) | | |
|  | ***GRADE*** | Moderate ⊕⊖⊕⊕ | | | Low ⊕⊖⊕⊖ | | |
| **Method. quality**  **(mod AMSTAR2)** | **Overall** | Moderate | | | | | |
| **Further investigations, sub- or sensitivity analyses** | **Index test** | | **Reference standard** | **Studies (Particip.)** | | **Sens. [%], 95% CI** | **Spec. [%], 95% CI** |
|  | Ultrasound, overall | | Chest or abdominal radiography | 10 (1812) | | 96 (94; 97) | 91 (85; 96) |
|  | Ultrasound, physician-performed | | Chest or abdominal radiography |  | | 96 (94; 97) | 91 (84; 95) |
|  | Ultrasound, nurse-performed | | Chest or abdominal radiography |  | | 96 (91; 98) | 100 (48; 100) |
|  | Ultrasound, ICU setting | | Chest or abdominal radiography |  | | 96 (95; 97) | 89 (81; 95) |
|  | Ultrasound, prehospital setting | | Chest or abdominal radiography |  | | 95 (92 ;98) | 100 (86; 100) |
| **Conclusions of study authors** | *„Our findings support the use of ultrasonography to confirm gastric tube placement. “* | | | | | | |

##### Musculoskeletal

###### Calcium Pyrophosphate Dehydrate Deposition Disease

| **Filippou (2016)**[168] – Calcium Pyrophosphate Dehydrate Deposition Disease (CPPD) | | | | | | | |
| --- | --- | --- | --- | --- | --- | --- | --- |
| **Clinical context** | ***Patient population*** | Patients with suspected CPPD | | | | | |
|  | ***Clinical setting*** | Not documented | | | | | |
|  | ***Prevalence [%] (CI)*** | Not documented | | | | | |
|  | ***Noteworthy incl. crit.*** | All study types included | | | | | |
|  | ***Noteworthy excl. crit.*** | - | | | | | |
| **Index test(s)** | B-mode ultrasound of fibrocartilage or hyaline cartilage | | | | | | |
| **Reference standard(s)** | Synovial fluid analysis, microscopic analysis | | | | | | |
| **DTA-SR characteristics** | ***No. of studies*** | 5 | | | | | |
|  | ***No. of participants*** | 184 at least (one study didn’t report amount of participants) | | | | | |
|  | ***Year range*** | 2007 - 2014 | | | | | |
|  | ***Quality assessment*** | QUADAS-2 (only low risk of bias studies) | | | | | |
|  | ***Special remark*** | - | | | | | |
| **Diagnostic accuracy** | ***Measure*** | Sensitivity [%] (95% CI):  89.0 (72.0; 97.0) | | | Specificity [%] (95% CI):  94.0 (87.0; 98.0) | | |
|  | ***GRADE*** | Very low ⊖⊖⊖⊖ | | | Very low ⊖⊖⊖⊖ | | |
| **Method. quality**  **(mod AMSTAR2)** | **Overall** | Low | | | | | |
| **Further investigations, sub- or sensitivity analyses** | **Index test** | | **Reference standard** | **Studies (Particip.)** | | **Sens. [%], 95% CI** | **Spec. [%], 95% CI** |
|  | Ultrasound of Hyaline cartilage | | Synovial fluid analysis, microscopic analysis | 7 | | 77 (63; 87) | 92 (16; 100) |
|  | Ultrasound of Fibrocartilage | | Synovial fluid analysis, microscopic analysis | 5 | | 77 (31; 96) | 96 (75; 100) |
|  | Ultrasound of Tendons | | Synovial fluid analysis, microscopic analysis | 2 | | 34 (16; 58) | 100 (89; 100) |
|  | Ultrasound of Fibrocartilage or Hyaline cartilage | | Synovial fluid analysis | 5 | | 87 (76; 99) | 98 (96; 100) |
|  | Ultrasound of Fibrocartilage or Hyaline cartilage | | Radiography | 2 | | 58 (9; 100) | 84 (52; 100) |
|  | Ultrasound of Fibrocartilage or Hyaline cartilage | | McCarty criteria | 3 | | 34 (2; 65) | 100 (99; 100) |
|  | Ultrasound of Fibrocartilage or Hyaline cartilage | | Microscopic analysis | 2 | | 84 (61; 100) | 93 (77; 100) |
|  | Publication bias was evaluated by the symmetry of the funnel plot of logDOR. This diagram and forma testing showed a marginally significant small study effect, but not in the direction of publication bias. | | | | | | |
| **Conclusions of study authors** | “*Ultrasound is potentially a useful tool for the diagnosis of CPPD.*” | | | | | | |

| **Gamon (2015)**[169] – Calcium Pyrophosphate Dehydrate Deposition Disease (CPPD) | | | | | | | |
| --- | --- | --- | --- | --- | --- | --- | --- |
| **Clinical context** | ***Patient population*** | Patients with diagnosed CPPD and control | | | | | |
|  | ***Clinical setting*** | Not documented | | | | | |
|  | ***Prevalence [%] (CI)*** | Not documented | | | | | |
|  | ***Noteworthy incl. crit.*** | Ultrasound on patients with CPPD diagnosed by McCarthy criteria or by crystal microscopy or X-rays | | | | | |
|  | ***Noteworthy excl. crit.*** | Case reports, concise reports, review articles and letters to the editor that were purely commentary or non-human studies | | | | | |
| **Index test(s)** | B-mode and Doppler ultrasound | | | | | | |
| **Reference standard(s)** | Calcium pyrophosphate crystal in synovial fluid analysis | | | | | | |
| **DTA-SR characteristics** | ***No. of studies*** | 8 | | | | | |
|  | ***No. of participants*** | 597 | | | | | |
|  | ***Year range*** | 2007-2014 | | | | | |
|  | ***Quality assessment*** | QUADAS | | | | | |
|  | ***Special remark*** | - | | | | | |
| **Diagnostic accuracy** | ***Measure*** | Sensitivity [%] (95% CI):  87.9 (80.9, 94.9) | | | Specificity [%] (95% CI):  91.5 (85.5, 97.2) | | |
|  | ***GRADE*** | Very low ⊖⊖⊖⊖ | | | Very low ⊖⊖⊖⊕ | | |
| **Method. quality**  **(mod AMSTAR2)** | **Overall** | Critically low | | | | | |
| **Further investigations, sub- or sensitivity analyses** | **Index test** | | **Reference standard** | **Studies (Particip.)** | | **Sens. [%], 95% CI** | **Spec. [%], 95% CI** |
|  |  | |  |  | |  |  |
|  |  | |  |  | |  |  |
|  |  | |  |  | |  |  |
|  |  | |  |  | |  |  |
| **Conclusions of study authors** | “*In conclusion, ultrasound is a promising tool for the diagnosis and management of CPPD to improve outcomes for patients; however, further work is required, particularly in defining the pathology and establishing the validity and reliability of the ultrasound -detected signs.*” | | | | | | |

| **Chen (2017)**[170] – Gout | | | | | | | |
| --- | --- | --- | --- | --- | --- | --- | --- |
| **Clinical context** | ***Patient population*** | Patients with gout and control | | | | | |
|  | ***Clinical setting*** | Not documented | | | | | |
|  | ***Prevalence [%] (CI)*** | 60.0 (54.2; 65.3) | | | | | |
|  | ***Noteworthy incl. crit.*** | Examining the performance of ultrasound. Crystal confirmation of gout after joint aspiration. | | | | | |
|  | ***Noteworthy excl. crit.*** | Studies not in English. Case reports, lectures reports, conference papers. | | | | | |
| **Index test(s)** | Echogenic foci, hyperechoic areas, the double contour, snow storm, tophus presence, hyperechoic cloudy area (Ultrasound of joints) | | | | | | |
| **Reference standard(s)** | Monosodium urate crystal in synovial fluid analysis | | | | | | |
| **DTA-SR characteristics** | ***No. of studies*** | 6 | | | | | |
|  | ***No. of participants*** | 299 | | | | | |
|  | ***Year range*** | 2008 – 2014 | | | | | |
|  | ***Quality assessment*** | QUADAS-2, at least 3 with high risk of bias | | | | | |
|  | ***Special remark*** |  | | | | | |
| **Diagnostic accuracy** | ***Measure*** | Sensitivity [%] (95% CI):  93.0 (90.0; 96.0) | | | Specificity [%] (95% CI):  73.0 (67.0; 78.0) | | |
|  | ***GRADE*** | Very low ⊖⊖⊖⊕ | | | Very low ⊖⊖⊖⊖ | | |
| **Method. quality**  **(mod AMSTAR2)** | **Overall** | Low | | | | | |
| **Further investigations, sub- or sensitivity analyses** | **Index test** | | **Reference standard** | **Studies (Particip.)** | | **Sens. [%], 95% CI** | **Spec. [%], 95% CI** |
|  |  | |  |  | |  |  |
|  |  | |  |  | |  |  |
|  |  | |  |  | |  |  |
|  |  | |  |  | |  |  |
| **Conclusions of study authors** | “*Based on current evidence, both ultrasound and dual energy CT can be used as major diagnostic methods for gouty arthritis, although the overall sensitivity of ultrasound is higher than that of dual energy CT. When physicians and radiologists come across patients with gouty arthritic attacks, ultrasound should be considered as the first choice and dual energy CT should act as a further verification tool.*” | | | | | | |

| **Lee (2018)**[171] – Gout | | | | | | | |
| --- | --- | --- | --- | --- | --- | --- | --- |
| **Clinical context** | ***Patient population*** | Patients with gout and control | | | | | |
|  | ***Clinical setting*** | Not documented | | | | | |
|  | ***Prevalence [%] (CI)*** | 38.1 (35.5; 40.8) | | | | | |
|  | ***Noteworthy incl. crit.*** | Studies which included subjects with suspicious gout, investigated with ultrasound and reference standard defined as classification criteria or joint aspirates for gout. | | | | | |
|  | ***Noteworthy excl. crit.*** | - | | | | | |
| **Index test(s)** | Double contour sign (ultrasound of joints) | | | | | | |
| **Reference standard(s)** | Classification criteria or monosodium urate crystal in synovial fluid analysis | | | | | | |
| **DTA-SR characteristics** | ***No. of studies*** | 11 | | | | | |
|  | ***No. of participants*** | 1726 | | | | | |
|  | ***Year range*** | 2007 - 2017 | | | | | |
|  | ***Quality assessment*** | QUADAS-2, 8 studies with high, 3 with unclear risk of bias | | | | | |
|  | ***Special remark*** | - | | | | | |
| **Diagnostic accuracy** | ***Measure*** | Sensitivity [%] (95% CI):  65.1 (62; 68.2) | | | Specificity [%] (95% CI):  89.0 (86.6; 91.1) | | |
|  | ***GRADE*** | Very low ⊕⊖⊖⊖ | | | Low ⊕⊖⊖⊕ | | |
| **Method. quality**  **(mod AMSTAR2)** | **Overall** | Moderate | | | | | |
| **Further investigations, sub- or sensitivity analyses** | **Index test** | | **Reference standard** | **Studies (Particip.)** | | **Sens. [%], 95% CI** | **Spec. [%], 95% CI** |
|  | Tophus | | Synovial fluid analysis | 7 (1341) | | 54 (51; 58) | 93 (91; 95) |
|  | Snowstorm | | Synovial fluid analysis | 2 (878) | | 31 (27: 35) | 91 (88; 93) |
|  | Bony erosion | | Synovial fluid analysis | 1 (92) | | 52 (39; 65) | 93 (78; 99) |
|  | Meta-regression showed that sample size, study design, and diagnostic criteria were not sources of heterogeneity in the meta-analysis. | | | | | | |
| **Conclusions of study authors** | “*In conclusion, our meta-analysis demonstrates that ultrasound offers good diagnostic accuracy. It plays an important role in the diagnosis of gout and can assist in differentiating gout from other arthritic diseases. Ultrasound is therefore an important component of the noninvasive assessment of gout. However, considering the limitations and the heterogeneity in this study, further studies with homogenous clinical characteristics and large populations are warranted to definitively validate the diagnostic value of ultrasound in gout. Ultrasound features of tophus, snowstorm, or bony erosion besides the double contour sign had limited sensitivity, but they had high specificity for diagnosis of gout.*” | | | | | | |

| **Ogdie (2015)**[172] – Gout | | | | | | | |
| --- | --- | --- | --- | --- | --- | --- | --- |
| **Clinical context** | ***Patient population*** | Patients with gout and control | | | | | |
|  | ***Clinical setting*** | Secondary care clinics | | | | | |
|  | ***Prevalence [%] (CI)*** | Not documented | | | | | |
|  | ***Noteworthy incl. crit.*** | Two groups of participants, one group had gout as determined by monosodium urate crystal identification as reference test | | | | | |
|  | ***Noteworthy excl. crit.*** | Asymptomatic hyperuricemia | | | | | |
| **Index test(s)** | Double contour sign (ultrasound of joints) | | | | | | |
| **Reference standard(s)** | Monosodium urate crystal in synovial fluid analysis | | | | | | |
| **DTA-SR characteristics** | ***No. of studies*** | 5 | | | | | |
|  | ***No. of participants*** | 363 | | | | | |
|  | ***Year range*** | 2003-2014 | | | | | |
|  | ***Quality assessment*** | QUADAS | | | | | |
|  | ***Special remark*** | - | | | | | |
| **Diagnostic accuracy** | ***Measure*** | Sensitivity [%] (95% CI):  83.0 (72.0, 91.0) | | | Specificity [%] (95% CI):  76.0 (68.0, 83.0) | | |
|  | ***GRADE*** | Very low ⊖⊖⊖⊖ | | | Very low ⊖⊖⊖⊖ | | |
| **Method. quality**  **(mod AMSTAR2)** | **Overall** | Low | | | | | |
| **Further investigations, sub- or sensitivity analyses** | **Index test** | | **Reference standard** | **Studies (Particip.)** | | **Sens. [%], 95% CI** | **Spec. [%], 95% CI** |
|  | Tophus | | Monosodium urate crystal in synovial fluid analysis | 334 (5) | | 65 (34; 87) | 80 (38; 96) |
| **Conclusions of study authors** | *“The results available suggest that ultrasound may be useful to include in revised gout clinical classification criteria.*” | | | | | | |

| **Shang (2022)**[173] – Gout | | | | | | | |
| --- | --- | --- | --- | --- | --- | --- | --- |
| **Clinical context** | ***Patient population*** | Subjects with suspicious gout | | | | | |
|  | ***Clinical setting*** | Not documented | | | | | |
|  | ***Prevalence [%] (CI)*** | Not documented | | | | | |
|  | ***Noteworthy incl. crit.*** | Patients with suspicious gout. Imaging modality of dual energy CT and ultrasound. | | | | | |
|  | ***Noteworthy excl. crit.*** | Asymptomatic hyperuricemia. | | | | | |
| **Index test(s)** | Double contour sign (ultrasound of joints) | | | | | | |
| **Reference standard(s)** | Monosodium urate crystal in synovial fluid analysis | | | | | | |
| **DTA-SR characteristics** | ***No. of studies*** | 11 | | | | | |
|  | ***No. of participants*** | Not documented | | | | | |
|  | ***Year range*** | 2007 - 2017 | | | | | |
|  | ***Quality assessment*** | QUADAS-2 (8 studies included with high, 3 with unclear quality) | | | | | |
|  | ***Special remark*** | - | | | | | |
| **Diagnostic accuracy** | ***Measure*** | Sensitivity [%] (95% CI):  70.0 (58.0; 79.0) | | | Specificity [%] (95% CI):  95.0 (87.0; 98.0) | | |
|  | ***GRADE*** | Very low ⊖⊖⊖⊖ | | | Very low ⊖⊖⊖⊖ | | |
| **Method. quality**  **(mod AMSTAR2)** | **Overall** | Critically low | | | | | |
| **Further investigations, sub- or sensitivity analyses** | **Index test** | | **Reference standard** | **Studies (Particip.)** | | **Sens. [%], 95% CI** | **Spec. [%], 95% CI** |
|  | Tophus | | Synovial fluid analysis or clinical diagnosis criteria | 8 (1441) | | 57 (38; 74) | 99 (88; 100) |
|  | Double contour sign and Tophus | | Synovial fluid analysis or clinical diagnosis criteria | 11 (1668) | | 84 (73; 91) | 84 (78; 89) |
|  | Meta-regression showed that sample size, study design, and diagnostic criteria were not sources of heterogeneity in the meta-analysis. No significant publication bias was found with Deek’s tests in the quantitative analyses ultrasound methods | | | | | | |
| **Conclusions of study authors** | “*Ultrasound showed promising accuracy for gout diagnosis.*” | | | | | | |

| **Stewart (2021)**[174] – Gout | | | | | | | |
| --- | --- | --- | --- | --- | --- | --- | --- |
| **Clinical context** | ***Patient population*** | Adults with suspicion of gout | | | | | |
|  | ***Clinical setting*** | Outpatient setting | | | | | |
|  | ***Prevalence [%] (CI)*** | 53.5 (50.8; 56.1) | | | | | |
|  | ***Noteworthy incl. crit.*** | Two groups of participants, one group had gout as determined by monosodium urate crystal identification as reference test | | | | | |
|  | ***Noteworthy excl. crit.*** | Long standing, established, or tophaceous gout | | | | | |
| **Index test(s)** | Tophus (ultrasound of joints) | | | | | | |
| **Reference standard(s)** | Monosodium urate crystal in synovial fluid analysis | | | | | | |
| **DTA-SR characteristics** | ***No. of studies*** | 7 | | | | | |
|  | ***No. of participants*** | 1363 | | | | | |
|  | ***Year range*** | 2007-2021 | | | | | |
|  | ***Quality assessment*** | QUADAS-2 | | | | | |
|  | ***Special remark*** | Network analysis | | | | | |
| **Diagnostic accuracy** | ***Measure*** | Sensitivity [%] (95% CI):  60.0 (35.0, 82.0) | | | Specificity [%] (95% CI):  94.0 (89.0, 97.0) | | |
|  | ***GRADE*** | Very low ⊖⊖⊖⊖ | | | Very low ⊖⊖⊖⊕ | | |
| **Method. quality**  **(mod AMSTAR2)** | **Overall** | Low | | | | | |
| **Further investigations, sub- or sensitivity analyses** | **Index test** | | **Reference standard** | **Studies (Particip.)** | | **Sens. [%], 95% CI** | **Spec. [%], 95% CI** |
|  | Double contour sign | | Monosodium urate crystal in synovial fluid analysis | 6 (1317) | | 71 (58, 82) | 87 (80, 92 |
|  | Aggregates | | Monosodium urate crystal in synovial fluid analysis | 2 (171) | | 71 (12, 98) | 96 (2, 100) |
|  | Snowstorm | | Monosodium urate crystal in synovial fluid analysis | 2 (878) | | 31 (27, 35) | 91 (87, 93) |
|  | Any ultrasound features | | Monosodium urate crystal in synovial fluid analysis | 4 (1049) | | 90 (55-99) | 84 (81-87) |
| **Conclusions of study authors** | “*In conclusion, this analysis has shown that DECT evidence of monosodium urate crystal deposition has similar sensitivity for the diagnosis of gout compared to ultrasound double contour sign, while tophus on ultrasound is ranked the most specific for gout. Overall, DECT and ultrasound are both useful modalities for the detection of imaging features of monosodium urate deposition, and have a similar overall diagnostic accuracy in patients with unclear diagnoses or suspected gout.*” | | | | | | |

**Abbreviations:** CI = Confidence Interval; DTA = Diagnostic test accuracy systematic review; DECT = Dual Energy Computer Tomography

| **Zhang (2018)**[175] – Gout | | | | | | | |
| --- | --- | --- | --- | --- | --- | --- | --- |
| **Clinical context** | ***Patient population*** | Not documented | | | | | |
|  | ***Clinical setting*** | Not documented | | | | | |
|  | ***Prevalence [%] (CI)*** | 53.3 (50.6; 56.0) | | | | | |
|  | ***Noteworthy incl. crit.*** | If any articles included overlapping data, the most comprehensive or recent article was selected | | | | | |
|  | ***Noteworthy excl. crit.*** | Patients with asymptomatic hyperuricemia | | | | | |
| **Index test(s)** | Double contour sign (DCS, ultrasound of joints person-based) | | | | | | |
| **Reference standard(s)** | Monosodium urate crystal in synovial fluid analysis | | | | | | |
| **DTA-SR characteristics** | ***No. of studies*** | 6 | | | | | |
|  | ***No. of participants*** | 1272 | | | | | |
|  | ***Year range*** | 2003-2017 | | | | | |
|  | ***Quality assessment*** | QUADAS-1 | | | | | |
|  | ***Special remark*** | Person-based vs joint-/location-based assessment | | | | | |
| **Diagnostic accuracy** | ***Measure*** | Sensitivity [%] (95% CI):  66.0 (62.0, 69.0) | | | Specificity [%] (95% CI):  92.0 (89.0,94.0) | | |
|  | ***GRADE*** | Very low ⊖⊖⊖⊖ | | | Very low ⊖⊖⊖⊕ | | |
| **Method. quality**  **(mod AMSTAR2)** | **Overall** | Low | | | | | |
| **Further investigations, sub- or sensitivity analyses** | **Index test** | | **Reference standard** | **Studies (Particip.)** | | **Sens. [%], 95% CI** | **Spec. [%], 95% CI** |
|  | DCS joint-/location-based | | Monosodium urate crystal in synovial fluid analysis | 4 (523) | | 75 (68,80) | 65 (59, 70) |
|  | Tophus person-based | | Monosodium urate crystal in synovial fluid analysis | 5 (1201) | | 56 (52, 60) | 94 (92, 96) |
|  | Snowstorm person-based | | Monosodium urate crystal in synovial fluid analysis | 3 (941) | | 31 (27, 36) | 91 (88, 93) |
|  | Overall consideration | | Monosodium urate crystal in synovial fluid analysis | 4 (1120) | | 80 (76, 83) | 83 (79, 86) |
| **Conclusions of study authors** | *“[…] our comprehensive meta-analysis demonstrates that ultrasound is a useful tool in the diagnosis of gout. The double contour sign, the presence of tophi and the snowstorm sign on ultrasound have high specificity but modest to low sensitivity in the diagnosis of gout for person-based evaluations. Simultaneously considering these features may improve the diagnostic sensitivity of ultrasound for gout. However, the double contour sign alone is weak in the differentiation of gout and non-gout for joint-/location-based evaluations.*” | | | | | | |

###### Vasculitis

| **El Yaman (2026)**[176] – Giant cell arteritis | | | | | | | |
| --- | --- | --- | --- | --- | --- | --- | --- |
| **Clinical context** | ***Patient population*** | Adults | | | | | |
|  | ***Clinical setting*** | Fast-track/secondary/tertiary care | | | | | |
|  | ***Prevalence [%] (CI)*** | Not documented | | | | | |
|  | ***Noteworthy incl. crit.*** | Clinical features suggestive of GCA (for example, new temporal headache, jaw claudication, or acute visual symptoms). Studies | | | | | |
|  | ***Noteworthy excl. crit.*** | Patients received high-dose corticosteroids for more than two weeks before imaging. | | | | | |
| **Index test(s)** | Color Doppler (different signs) | | | | | | |
| **Reference standard(s)** | Biopsy and clinical diagnosis based on accepted criteria and clinical adjudication (commonly using American College of Rheumatology/European League Against Rheumatism (ACR/EULAR) clinical classification and local diagnostic pathways). | | | | | | |
| **DTA-SR characteristics** | ***No. of studies*** | 31 | | | | | |
|  | ***No. of participants*** | 2766 | | | | | |
|  | ***Year range*** | 1997 - 2024 | | | | | |
|  | ***Quality assessment*** | QUADAS-2 | | | | | |
|  | ***Special remark*** |  | | | | | |
| **Diagnostic accuracy** | ***Measure*** | Sensitivity [%] (95% CI):  83.0 (79.0; 87.0) | | | Specificity [%] (95% CI):  88.0 (84.0; 91.0) | | |
|  | ***GRADE*** | Low ⊕⊖⊖⊕ | | | Low ⊕⊖⊖⊕ | | |
| **Method. quality (mod AMSTAR2)** | **Overall** | Moderate | | | | | |
| **Further investigations, sub- or sensitivity analyses** | **Index test** | | **Reference standard** | **Studies (Particip.)** | | **Sens. [%], 95% CI** | **Spec. [%], 95% CI** |
|  | Unchanged | | Temporal artery biopsy | 17 (NA) | | 77 (82; 83) | 83 (77; 90) |
|  |  | |  |  | |  |  |
|  |  | |  |  | |  |  |
|  |  | |  |  | |  |  |
| **Conclusions of study authors** | *“Ultrasound remains an effective and scalable first-line test in experienced centers, particularly within fast-track pathways, while MRI provides useful confirmatory information when ultrasound is inconclusive or expertise is limited.”* | | | | | | |

| **Pouncey (2024)**[177] – Giant cell arteritis | | | |
| --- | --- | --- | --- |
| **Clinical context** | ***Patient population*** | Clinically suspected giant cell arteritis in every age | |
|  | ***Clinical setting*** | Outpatient or inpatient clinical setting | |
|  | ***Prevalence [%] (CI)*** | 41.7; (39.4; 44.2) | |
|  | ***Noteworthy incl. crit.*** | - | |
|  | ***Noteworthy excl. crit.*** | All studies in which ultrasound was performed more than two weeks following formal diagnosis, to exclude patients with two or more weeks of steroid treatment | |
| **Index test(s)** | Halo sign (temporal artery ultrasound) | | |
| **Reference standard(s)** | Clinical diagnosis (ACR classification) | | |
| **DTA-SR characteristics** | ***No. of studies*** | 15 | |
|  | ***No. of participants*** | 1479 | |
|  | ***Year range*** | 1997 - 2020 | |
|  | ***Quality assessment*** | QUADAS-2 (14 low quality studies) | |
|  | ***Special remark*** | - | |
| **Diagnostic accuracy** | ***Measure*** | Sensitivity [%] (95% CI):  78.0 (74.6; 81.3) | Specificity [%] (95% CI):  91.0 (89.0; 92.9) |
|  | ***GRADE*** | Very low ⊕⊖⊖⊖ | Very low ⊕⊖⊖⊖ |
| **Method. quality**  **(mod AMSTAR2)** | **Overall** | High | |

| **Further investigations, sub- or sensitivity analyses** | **Index test** | **Reference standard** | **Studies (Particip.)** | **Sens. [%], 95% CI** | **Spec. [%], 95% CI** |
| --- | --- | --- | --- | --- | --- |
|  |  |  |  |  |  |
|  |  |  |  |  |  |
|  |  |  |  |  |  |
|  |  |  |  |  |  |

| **Conclusions of study authors** | “*There is limited published evidence on the accuracy of temporal artery ultrasound for detecting giant cell arteritis. Ultrasound seems to be moderately sensitive when the specificity is good, but data were heterogeneous across studies and either did not use the same halo thickness threshold or did not report it. We can draw no conclusions from accuracy studies on whether ultrasound can replace temporal artery biopsy for diagnosing giant cell arteritis given the very low certainty of the evidence. Future research could consider using the 2016 revision of the ACR criteria as a reference standard, which will limit incorporation bias of temporal artery biopsy into the reference standard.*” |
| --- | --- |

| **Rinagel (2019)**[178] – Giant cell arteritis | | | |
| --- | --- | --- | --- |
| **Clinical context** | ***Patient population*** | Adults | |
|  | ***Clinical setting*** | Not documented | |
|  | ***Prevalence [%] (CI)*** | Not documented | |
|  | ***Noteworthy incl. crit.*** | - | |
|  | ***Noteworthy excl. crit.*** | - | |
| **Index test(s)** | Any ultrasound sign (temporal artery ultrasound) | | |
| **Reference standard(s)** | Temporal artery biopsy | | |
| **DTA-SR characteristics** | ***No. of studies*** | 11 | |
|  | ***No. of participants*** | 1061 | |
|  | ***Year range*** | 1997 – 2013 | |
|  | ***Quality assessment*** | QUADAS-2 | |
|  | ***Special remark*** | - | |
| **Diagnostic accuracy** | ***Measure*** | Sensitivity [%] (95% CI):  78.0 (64.0, 87.0) | Specificity [%] (95% CI):  79.0 (73.0, 85.0) |
|  | ***GRADE*** | Very low ⊖⊖⊖⊖ | Very low ⊖⊖⊖⊖ |
| **Method. quality**  **(mod AMSTAR2)** | **Overall** | Low | |

| **Further investigations, sub- or sensitivity analyses** | **Index test** | **Reference standard** | **Studies (Particip.)** | **Sens. [%], 95% CI** | **Spec. [%], 95% CI** |
| --- | --- | --- | --- | --- | --- |
|  | Halo sign | Temporal artery biopsy | 20 (1046) | 68 (57, 78) | 81 (75, 86) |
|  |  |  |  |  |  |
|  |  |  |  |  |  |
|  |  |  |  |  |  |

| **Conclusions of study authors** | “*This systematic review and meta-analysis provides valuable estimates of positive and negative likelihood ratios for abnormal temporal artery ultrasound compared to temporal artery biopsy for the diagnosis of giant cell arteritis. These summary parameters may prove very useful to assess the risk of giant cell arteritis routinely at the bed-side. Another important finding it that taking into account any colour duplex sonography abnormalities increased only slightly the sensitivity compared to using the halo sign, with a very similar specificity are largely overlapping 95% confidence interval area, which may have some very practical implications.*” |
| --- | --- |

| **Sebastian (2021)**[179] – Giant cell arteritis | | | | | | | |
| --- | --- | --- | --- | --- | --- | --- | --- |
| **Clinical context** | ***Patient population*** | Clinically suspected giant cell arteritis, 50 year and older | | | | | |
|  | ***Clinical setting*** | Not documented | | | | | |
|  | ***Prevalence [%] (CI)*** | Not documented | | | | | |
|  | ***Noteworthy incl. crit.*** | Retrospective and prospective studies, ultrasound was performed at any time from the clinical suspicion of giant cell arteritis | | | | | |
|  | ***Noteworthy excl. crit.*** | - | | | | | |
| **Index test(s)** | Halo sign (temporal artery ultrasound) | | | | | | |
| **Reference standard(s)** | Clinical diagnosis or temporal artery biopsy | | | | | | |
| **DTA-SR characteristics** | ***No. of studies*** | 18 | | | | | |
|  | ***No. of participants*** | 1502 | | | | | |
|  | ***Year range*** | 1997 - 2020 | | | | | |
|  | ***Quality assessment*** | QUADAS-2, 11 studies with high risk of bias | | | | | |
|  | ***Special remark*** | - | | | | | |
| **Diagnostic accuracy** | ***Measure*** | Sensitivity [%] (95% CI):  67.0 (51.0; 80.0) | | | Specificity [%] (95% CI):  95.0 (89.0; 98.0) | | |
|  | ***GRADE*** | Very low ⊕⊖⊖⊖ | | | Low ⊕⊖⊖⊕ | | |
| **Method. quality**  **(mod AMSTAR2)** | **Overall** | Moderate | | | | | |
| **Further investigations, sub- or sensitivity analyses** | **Index test** | | **Reference standard** | **Studies (Particip.)** | | **Sens. [%], 95% CI** | **Spec. [%], 95% CI** |
|  | Halo sign or stenosis or occlusion | | Physician’s clinical diagnosis | 4 (270) | | 52 (18; 84) | 81 (64; 91) |
|  | Halo sign or stenosis | | Physician’s clinical diagnosis | 4 (230) | | 43 (12; 80) | 85 (66; 94) |
|  | Halo sign | | Temporal artery biopsy | 15 (1209) | | 67 (15; 81) | 95 (89; 98) |
| **Conclusions of study authors** | “*This meta-analysis shows that the ultrasound halo sign has a significant role in the assessment and diagnosis of giant cell arteritis. Ultrasound is a sensitive and specific approach for giant cell arteritis assessment, which seems to be improving with better equipment and user familiarity with ultrasound techniques. However, the studies analysed showed heterogeneity in their design and outcomes. Therefore, it is recommended that future researchers conduct multicentre prospective studies for analysing the effectiveness of the halo sign in the assessment of giant cell arteritis, with a standardized study protocol.*” | | | | | | |

| **Duftner (2018)**[180] – Large vessel vasculitis | | | | | | | |
| --- | --- | --- | --- | --- | --- | --- | --- |
| **Clinical context** | ***Patient population*** | Adults and children | | | | | |
|  | ***Clinical setting*** | Not documented | | | | | |
|  | ***Prevalence [%] (CI)*** | 38.7 (34.8; 42.6) | | | | | |
|  | ***Noteworthy incl. crit.*** | Suspected large vessel vasculitis, prospective studies | | | | | |
|  | ***Noteworthy excl. crit.*** | - | | | | | |
| **Index test(s)** | Halo sign (temporal artery) | | | | | | |
| **Reference standard(s)** | Physician’s clinical diagnosis | | | | | | |
| **DTA-SR characteristics** | ***No. of studies*** | 8 (3 with low risk of bias) | | | | | |
|  | ***No. of participants*** | 605 | | | | | |
|  | ***Year range*** | 1997 - 2014 | | | | | |
|  | ***Quality assessment*** | QUADAS-2, 5 studies included despite insufficient quality | | | | | |
|  | ***Special remark*** | No studies on takayasu arteritis were identified | | | | | |
| **Diagnostic accuracy** | ***Measure*** | Sensitivity [%] (95% CI):  77.0 (62.0; 87.0) | | | Specificity [%] (95% CI):  96.0 (85.0; 99.0) | | |
|  | ***GRADE*** | Very low ⊖⊖⊖⊖ | | | Very low ⊖⊕⊖⊖ | | |
| **Method. quality**  **(mod AMSTAR2)** | **Overall** | Low | | | | | |
| **Further investigations, sub- or sensitivity analyses** | **Index test** | | **Reference standard** | **Studies (Particip.)** | | **Sens. [%], 95% CI** | **Spec. [%], 95% CI** |
|  | Halo sign or stenosis or occlusion | | Physician’s clinical diagnosis | 3 (560) | | 78 (57; 90) | 89 (78; 95) |
|  | Halo sign | | Temporal artery biopsy | 7 (289) | | 70 (65; 81) | 84 (73;91) |
|  | Halo sign or stenosis | | Temporal artery biopsy | 2 (50) | | 77 (23; 97) | 91 (75; 97) |
|  | Halo sign or stenosis or occlusion | | Temporal artery biopsy | 5 (611) | | 78 (48; 93) | 91 (70; 98) |
| **Conclusions of study authors** | “*Ultrasound provides a high diagnostic value for cranial giant cell arteritis.*” | | | | | | |

| **Bosch (2023)**[181] – Large vessel vasculitis | | | | | | | |
| --- | --- | --- | --- | --- | --- | --- | --- |
| **Clinical context** | ***Patient population*** | Adults with suspected large vessel vasculitis | | | | | |
|  | ***Clinical setting*** | Not documented | | | | | |
|  | ***Prevalence [%] (CI)*** | 49.5 (45.4; 53.6) | | | | | |
|  | ***Noteworthy incl. crit.*** |  | | | | | |
|  | ***Noteworthy excl. crit.*** |  | | | | | |
| **Index test(s)** | Halo sign with/without compression sign or thickened wall (axillary, carotid, facial and mostly temporal arteries) | | | | | | |
| **Reference standard(s)** | Clinical diagnosis or temporal artery biopsy | | | | | | |
| **DTA-SR characteristics** | ***No. of studies*** | 8 | | | | | |
|  | ***No. of participants*** | 584 | | | | | |
|  | ***Year range*** | 2006 - 2022 | | | | | |
|  | ***Quality assessment*** | QUADAS-2 (4 low, 7 unclear, 1 high risk of bias) | | | | | |
|  | ***Special remark*** |  | | | | | |
| **Diagnostic accuracy** | ***Measure*** | Sensitivity [%] (95% CI):  88.0 (82.0; 92.0) | | | Specificity [%] (95% CI):  96.0 (86.0; 99.0) | | |
|  | ***GRADE*** | Very low ⊝⊝⊝⊕ | | | Very low ⊝⊝⊝⊕ | | |
| **Method. quality**  **(mod AMSTAR2)** | **Overall** | Low | | | | | |
| **Further investigations, sub- or sensitivity analyses** | **Index test** | | **Reference standard** | **Studies (Particip.)** | | **Sens. [%], 95% CI** | **Spec. [%], 95% CI** |
|  | Halo sign with/without compression sign (all studies) | | Clinical diagnosis or temporal artery biopsy | 23 (1981) | | 75 (66; 83) | 91 (86; 94)) |
|  |  | |  |  | |  |  |
|  |  | |  |  | |  |  |
|  |  | |  |  | |  |  |
| **Conclusions of study authors** | *“Ultrasound revealed a good performance for the diagnosis of giant cell arthritis. Cranial and extracranial vascular ultrasound had a higher pooled sensitivity with similar specificity compared with limited cranial ultrasound.”* | | | | | | |

###### Musculoskeletal other

| **Popat (2024)**[110] – Abscess | | | | | | | |
| --- | --- | --- | --- | --- | --- | --- | --- |
| **Clinical context** | ***Patient population*** | Adults and children | | | | | |
|  | ***Clinical setting*** | Emergency department | | | | | |
|  | ***Prevalence [%] (CI)*** | Not documented | | | | | |
|  | ***Noteworthy incl. crit.*** | - | | | | | |
|  | ***Noteworthy excl. crit.*** | - | | | | | |
| **Index test(s)** | POCUS | | | | | | |
| **Reference standard(s)** | Needle aspiration or incision-drainage | | | | | | |
| **DTA-SR characteristics** | ***No. of studies*** | 5 | | | | | |
|  | ***No. of participants*** | Not documented | | | | | |
|  | ***Year range*** | 2009 - 2020 | | | | | |
|  | ***Quality assessment*** | QUADAS-2 (Various with high risk of bias overall, not reported separately) | | | | | |
| **Diagnostic accuracy** | ***Measure*** | Sensitivity [%] (95% CI):  94.8 (90.6; 98.9) | | | Specificity [%] (95% CI):  85.2 (72.2; 98.1) | | |
|  | ***GRADE*** | Moderate ⊖⊕⊕⊕ | | | Low ⊖⊕⊕⊖ | | |
| **Method. quality**  **(mod AMSTAR2)** | **Overall** | Moderate | | | | | |
| **Further investigations, sub- or sensitivity analyses** | **Index test** | | **Reference standard** | **Studies (Particip.)** | | **Sens. [%], 95% CI** | **Spec. [%], 95% CI** |
|  |  | |  |  | |  |  |
|  |  | |  |  | |  |  |
|  |  | |  |  | |  |  |
|  |  | |  |  | |  |  |
|  |  | |  |  | |  |  |
| **Conclusions of study authors** | *“POCUS has increasingly been utilized in the emergency department over the past two decades. It also indicates that POCUS in the emergency department can accurately diagnose clinical conditions such as small bowel obstruction, retinal detachment, abscess, clavicle fractures, distal forearm fractures, metacarpal fractures, skull fractures, pleural effusion, and deep venous thrombosis. In addition, we have demonstrated that POCUS can aid in ruling in appendicitis, foreign bodies, and cholecystitis due to its high specificity.”* | | | | | | |

| **Barbic (2017)**[182] – Abscess | | | | | | | |
| --- | --- | --- | --- | --- | --- | --- | --- |
| **Clinical context** | ***Patient population*** | Adults and children with skin and soft tissue infection | | | | | |
|  | ***Clinical setting*** | Emergency department | | | | | |
|  | ***Prevalence [%] (CI)*** | 80.7 (77.7; 83.4) | | | | | |
|  | ***Noteworthy incl. crit.*** | Prospective cohort and case-control studies. | | | | | |
|  | ***Noteworthy excl. crit.*** | Primary care or inpatient setting. Case reports, retrospective studies and other types of case–control studies | | | | | |
| **Index test(s)** | POCUS | | | | | | |
| **Reference standard(s)** | CT, purulent discharge, follow-up | | | | | | |
| **DTA-SR characteristics** | ***No. of studies*** | 8 | | | | | |
|  | ***No. of participants*** | 747 | | | | | |
|  | ***Year range*** | 1997 - 2016 | | | | | |
|  | ***Quality assessment*** | QUADAS-2 (2 high risk of bias) | | | | | |
|  | ***Special remark*** |  | | | | | |
| **Diagnostic accuracy** | ***Measure*** | Sensitivity [%] (95% CI):  96.2 (91.1; 98.4) | | | Specificity [%] (95% CI):  82.9 (60.4; 93.9) | | |
|  | ***GRADE*** | High ⊕⊕⊕⊕ | | | Low ⊕⊝⊕⊝ | | |
| **Method. quality**  **(mod AMSTAR2)** | **Overall** | Moderate | | | | | |
| **Further investigations, sub- or sensitivity analyses** | **Index test** | | **Reference standard** | **Studies (Particip.)** | | **Sens. [%], 95% CI** | **Spec. [%], 95% CI** |
|  |  | |  |  | |  |  |
|  |  | |  |  | |  |  |
|  |  | |  |  | |  |  |
|  |  | |  |  | |  |  |
| **Conclusions of study authors** | *“A total of 8 studies of good-to-excellent quality were included in this review. The use of POCUS helps differentiate abscess from cellulitis in emergency department patients with skin and soft tissue infection”* | | | | | | |

| **Meyer (2022)**[183] – Knee Effusions | | | | | | | |  |
| --- | --- | --- | --- | --- | --- | --- | --- | --- |
| **Clinical context** | ***Patient population*** | Adults and children | | | | | |  |
|  | ***Clinical setting*** | Not documented | | | | | |  |
|  | ***Prevalence [%] (CI)*** | 43.0 (38.0; 48.0) | | | | | |  |
|  | ***Noteworthy incl. crit.*** | Knee pain, swelling, or effusion | | | | | |  |
|  | ***Noteworthy excl. crit.*** | Case reports of single patients | | | | | |  |
| **Index test(s)** | B-mode ultrasound for synovial effusion | | | | | | |  |
| **Reference standard(s)** | MRI | | | | | | |  |
| **DTA-SR characteristics** | ***No. of studies*** | 8 | | | | | |  |
|  | ***No. of participants*** | 370 | | | | | |  |
|  | ***Year range*** | 1990 - 2019 | | | | | |  |
|  | ***Quality assessment*** | QUADAS-2 | | | | | |  |
|  | ***Special remark*** | This study compared 3 groups: physical examination vs Ultrasound, physical examination vs MRI, ultrasound vs MRI. 4 studies include pediatric populations, however fluid measurements have been shown to not be affected by age groups | | | | | |  |
| **Diagnostic accuracy** | ***Measure*** | Sensitivity [%] (95% CI):  84.0 (78.0, 89.0) | | | Specificity [%] (95% CI):  93.0 (73.0, 99.0) | | |  |
|  | ***GRADE*** | Low ⊕⊖⊕⊖ | | | Low ⊕⊖⊕⊖ | | |  |
| **Method. quality (mod AMSTAR2)** | **Overall** | Moderate | | | | | |  |
| **Further investigations, sub- or sensitivity analyses** | **Index test** | | **Reference standard** | **Studies (Particip.)** | | **Sens. [%], 95% CI** | **Spec. [%], 95% CI** | |
|  |  | |  |  | |  |  | |
|  |  | |  |  | |  |  | |
|  |  | |  |  | |  |  | |
|  |  | |  |  | |  |  | |
| **Conclusions of study authors** | *“Ultrasound has a higher sensitivity and similar specificity compared to the bulge sign and patellar tap in detecting knee effusion. If using ultrasound instead of these single physical examination maneuvers, positive and negative predictive value can be improved by up to 30%”* | | | | | | |  |

| **Götz (2023)**[184] – Necrotizing skin infection | | | | | | | |
| --- | --- | --- | --- | --- | --- | --- | --- |
| **Clinical context** | ***Patient population*** | Adults and children | | | | | |
|  | ***Clinical setting*** | Not documented | | | | | |
|  | ***Prevalence [%] (CI)*** | 62.1 (60.2; 64.1) | | | | | |
|  | ***Noteworthy incl. crit.*** | - | | | | | |
|  | ***Noteworthy excl. crit.*** | - | | | | | |
| **Index test(s)** | POCUS | | | | | | |
| **Reference standard(s)** | Surgery | | | | | | |
| **DTA-SR characteristics** | ***No. of studies*** | 13 | | | | | |
|  | ***No. of participants*** | 2522 | | | | | |
|  | ***Year range*** | 1997 - 2019 | | | | | |
|  | ***Quality assessment*** | QUADAS-2 | | | | | |
|  | ***Special remark*** | - | | | | | |
| **Diagnostic accuracy** | ***Measure*** | Sensitivity [%] (95% CI):  93.2 (90.3; 96.2) | | | Specificity [%] (95% CI):  87.9 (84.4; 91.1) | | |
|  | ***GRADE*** | Low ⊖⊖⊕⊕ | | | Low ⊖⊖⊕⊕ | | |
| **Method. quality**  **(mod AMSTAR2)** | **Overall** | Critically low | | | | | |
| **Further investigations, sub- or sensitivity analyses** | **Index test** | | **Reference standard** | **Studies (Particip.)** | | **Sens. [%], 95% CI** | **Spec. [%], 95% CI** |
|  |  | |  |  | |  |  |
|  |  | |  |  | |  |  |
|  |  | |  |  | |  |  |
|  |  | |  |  | |  |  |
| **Conclusions of study authors** | “*POCUS is a relatively accurate diagnostic test for necrotizing skin infection when compared to other methods. Its easy availability, high accuracy, and ability to be used without delaying surgical consultation make it a preferred diagnostic tool for confirming necrotising skin infection in clinical practice.*” | | | | | | |

| **Davis (2015)**[185] – Retained foreign bodies in skin and soft tissue | | | | | | | |
| --- | --- | --- | --- | --- | --- | --- | --- |
| **Clinical context** | ***Patient population*** | Adults and children with suspected foreign body | | | | | |
|  | ***Clinical setting*** | Not documented | | | | | |
|  | ***Prevalence [%] (CI)*** | Not documented | | | | | |
|  | ***Noteworthy incl. crit.*** | - | | | | | |
|  | ***Noteworthy excl. crit.*** | Foreign bodies that were intraocular, respiratory, or penetrated deeper than skin and soft tissue; on the topic of retained surgical equipment | | | | | |
| **Index test(s)** | B-mode ultrasound | | | | | | |
| **Reference standard(s)** | CT, surgery, follow-up | | | | | | |
| **DTA-SR characteristics** | ***No. of studies*** | 17 | | | | | |
|  | ***No. of participants*** | Not documented | | | | | |
|  | ***Year range*** | 1986 - 2014 | | | | | |
|  | ***Quality assessment*** | QUADAS-2 (all high risk of bias) | | | | | |
|  | ***Special remark*** |  | | | | | |
| **Diagnostic accuracy** | ***Measure*** | Sensitivity [%] (95% CI):  72.0 (57.0; 83.0) | | | Specificity [%] (95% CI):  92.0 (88.0; 95.0) | | |
|  | ***GRADE*** | Very low ⊖⊖⊕⊖ | | | Low ⊖⊖⊕⊕ | | |
| **Method. quality**  **(mod AMSTAR2)** | **Overall** | Low | | | | | |
| **Further investigations, sub- or sensitivity analyses** | **Index test** | | **Reference standard** | **Studies (Particip.)** | | **Sens. [%], 95% CI** | **Spec. [%], 95% CI** |
|  | Ultrasound for radiolucent or wooden foreign bodies | | CT, surgery, follow-up | 4 (NA) | | 97 (90; 99) | 84 (73; 92) |
|  | Ultrasound on emergency department | | CT, surgery, follow-up | 4 (NA) | | 91 (79; 96) | 91 (71; 97) |
|  |  | |  |  | |  |  |
|  |  | |  |  | |  |  |
|  |  | |  |  | |  |  |
|  |  | |  |  | |  |  |
|  |  | |  |  | |  |  |
|  |  | | | | | | |
| **Conclusions of study authors** | “*Ultrasonography can be a useful diagnostic tool in the evaluation of skin and soft tissue foreign bodies. It may complement traditional radiography in cases of suspected retained soft tissue foreign bodies of unknown origin. It should be strongly considered in high-risk cases and cases of radiolucent foreign bodies.”* | | | | | | |

| **Dinnes (2003)**[186] – Rotator cuff tear | | | | | | | |
| --- | --- | --- | --- | --- | --- | --- | --- |
| **Clinical context** | ***Patient population*** | Adults | | | | | |
|  | ***Clinical setting*** | Mostly radiology and orthopedics | | | | | |
|  | ***Prevalence [%] (CI)*** | 53% | | | | | |
|  | ***Noteworthy incl. crit.*** | All causes of shoulder pain, any reference test | | | | | |
|  | ***Noteworthy excl. crit.*** | Studies with healthy control groups | | | | | |
| **Index test(s)** | Any rotator cuff tear (shoulder ultrasound) | | | | | | |
| **Reference standard(s)** | Arthrography, arthroscopy, surgery, combination, other | | | | | | |
| **DTA-SR characteristics** | ***No. of studies*** | 29 | | | | | |
|  | ***No. of participants*** | Not documented | | | | | |
|  | ***Year range*** | 1985-2001 | | | | | |
|  | ***Quality assessment*** | Adapted from the QUADAS tool, developed by University of York | | | | | |
|  | ***Special remark*** | Subgroup analyses by lesion type, age, prevalence, publication year, ultrasound frequency, reference test, design, biases | | | | | |
| **Diagnostic accuracy** | ***Measure*** | Sensitivity [%] (95% CI):  80.0 (78.0, 83.0) | | | Specificity [%] (95% CI):  85.0 (82.0, 87.0) | | |
|  | ***GRADE*** | Low ⊖⊕⊕⊖ | | | Low ⊖⊕⊕⊖ | | |
| **Method. quality**  **(mod AMSTAR2)** | **Overall** | Moderate | | | | | |
| **Further investigations, sub- or sensitivity analyses** | **Index test** | | **Reference standard** | **Studies (Particip.)** | | **Sens. [%], 95% CI** | **Spec. [%], 95% CI** |
|  | Full-thickness rotator cuff tear | | Arthrography, arthroscopy, surgery, combination, other | 19 (NR) | | 87 (84, 89) | 96 (94, 97) |
|  | Partial-thickness rotator cuff tear | | Arthroscopy, arthroscopy/surgery, MRI | 11 (NR) | | 67 (61, 73) | 94 (92, 96) |
| **Conclusions of study authors** | *“Ultrasound may be able to rule in full-thickness tears and therefore rapidly identify those requiring surgical treatment, but this has as yet only been demonstrated in studies conducted in specialist settings where the prevalence of disease is high. Its diagnostic capabilities in more routine settings and its ability to rule out disease effectively when a negative result is shown is questionable and remains to be determined. There is as yet no conclusive evidence for its ability to rule in or out the presence of partial-thickness tears, but it seems likely that its ability to do either is low.”* | | | | | | |

| **Liu (2024)**[187] – Synovial abnormalities | | | | | | | |
| --- | --- | --- | --- | --- | --- | --- | --- |
| **Clinical context** | ***Patient population*** | Not documented | | | | | |
|  | ***Clinical setting*** | Not documented | | | | | |
|  | ***Prevalence [%] (CI)*** | Not documented | | | | | |
|  | ***Noteworthy incl. crit.*** | Knee pain, ultrasound to assess synovia abnormalities (ie, synovitis or synovial effusion) | | | | | |
|  | ***Noteworthy excl. crit.*** | Knee pain of non-musculoskeletal origin | | | | | |
| **Index test(s)** | Synovial effusion (knee ultrasound) | | | | | | |
| **Reference standard(s)** | MRI | | | | | | |
| **DTA-SR characteristics** | ***No. of studies*** | 8 | | | | | |
|  | ***No. of participants*** | 640 knees | | | | | |
|  | ***Year range*** | 2007 - 2021 | | | | | |
|  | ***Quality assessment*** | QUADAS-2 | | | | | |
|  | ***Special remark*** |  | | | | | |
| **Diagnostic accuracy** | ***Measure*** | Sensitivity [%] (95% CI):  90.0 (81.0; 95.0) | | | Specificity [%] (95% CI):  86.0 (77.0; 92.0) | | |
|  | ***GRADE*** | Very low ⊕⊖⊖⊖ | | | Very low ⊕⊖⊖⊖ | | |
| **Method. quality**  **(mod AMSTAR2)** | **Overall** | Moderate | | | | | |
| **Further investigations, sub- or sensitivity analyses** | **Index test** | | **Reference standard** | **Studies (Particip.)** | | **Sens. [%], 95% CI** | **Spec. [%], 95% CI** |
|  | Synovitis (knee ultrasound) | | MRI | 6 (199 knees) | | 88 (65; 96) | 70 (51; 84) |
| **Conclusions of study authors** | *“Overall, this meta-analysis implies that ultrasound is a valid approach for assessing synovitis and synovial effusion among patients with knee pain. Given that ultrasound is a more widely available, cost-effective, and real-time imaging procedure, it is therefore recommended as an adjuvant, especially for etiological diagnosis and individualized management among patients with knee pain.”* | | | | | | |

##### Neurological

###### Arterial Stenosis

| **Cassola (2022)**[188] **– Carotid artery stenosis (> 50%)** | | | | | | | |
| --- | --- | --- | --- | --- | --- | --- | --- |
| **Clinical context** | ***Patient population*** | Symptomatic adults (sudden visual loss, hemispheric TIA, and ischemic stroke) with suspected carotid artery stenosis | | | | | |
|  | ***Clinical setting*** | Not documented | | | | | |
|  | ***Prevalence [%] (CI)*** | 51.0 (47.9; 54.1) | | | | | |
|  | ***Noteworthy incl. crit.*** | Symptomatic patient (sudden visual loss, hemispheric TIA, and ischemic stroke) | | | | | |
|  | ***Noteworthy excl. crit.*** | Studies that included < 70% of symptomatic patients; or if the time between the index test and the reference standard was longer than four weeks or not described | | | | | |
| **Index test(s)** | Doppler ultrasound | | | | | | |
| **Reference standard(s)** | Digital subtraction angiography | | | | | | |
| **DTA-SR characteristics** | ***No. of studies*** | 5 | | | | | |
|  | ***No. of participants*** | 1007 | | | | | |
|  | ***Year range*** | 1993 - 2012 | | | | | |
|  | ***Quality assessment*** | QUADAS-2 (2 high risk of bias studies). | | | | | |
|  | ***Special remark*** |  | | | | | |
| **Diagnostic accuracy** | ***Measure*** | Sensitivity [%] (95% CI):  97.0 (95.0; 98.0) | | | Specificity [%] (95% CI):  70.0 (67.0; 73.0) | | |
|  | ***GRADE*** | Moderate ⊕⊕⊖⊕ | | | Low ⊕⊕⊖⊖ | | |
| **Method. quality**  **(mod AMSTAR2)** | **Overall** | High | | | | | |
| **Further investigations, sub- or sensitivity analyses** | **Index test** | | **Reference standard** | **Studies (Particip.)** | | **Sens. [%], 95% CI** | **Spec. [%], 95% CI** |
|  |  | |  |  | |  |  |
|  |  | |  |  | |  |  |
|  |  | |  |  | |  |  |
|  |  | |  |  | |  |  |
| **Conclusions of study authors** | *“The diagnostic accuracy of Doppler ultrasound is high, especially at discriminating between the presence or absence of significant carotid artery stenosis. This evidence, plus its less invasive nature, supports the early use of Doppler ultrasound for the detection of carotid artery stenosis.”* | | | | | | |

| **Cassola (2022)**[188] **– Carotid artery stenosis (> 70%)** | | | | | | | |
| --- | --- | --- | --- | --- | --- | --- | --- |
| **Clinical context** | ***Patient population*** | Symptomatic adults (sudden visual loss, hemispheric TIA, and ischemic stroke) with suspected carotid artery stenosis | | | | | |
|  | ***Clinical setting*** | Not documented | | | | | |
|  | ***Prevalence [%] (CI)*** | 45 (43.1; 46.9) | | | | | |
|  | ***Noteworthy incl. crit.*** | Symptomatic patient (sudden visual loss, hemispheric TIA, and ischemic stroke) | | | | | |
|  | ***Noteworthy excl. crit.*** | Studies that included < 70% of symptomatic patients; or if the time between the index test and the reference standard was longer than four weeks or not described | | | | | |
| **Index test(s)** | Doppler ultrasound | | | | | | |
| **Reference standard(s)** | Digital subtraction angiography | | | | | | |
| **DTA-SR characteristics** | ***No. of studies*** | 9 | | | | | |
|  | ***No. of participants*** | 2770 | | | | | |
|  | ***Year range*** | 1994- 2006 | | | | | |
|  | ***Quality assessment*** | QUADAS-2 (2 high risk of bias studies) | | | | | |
|  | ***Special remark*** |  | | | | | |
| **Diagnostic accuracy** | ***Measure*** | Sensitivity [%] (95% CI):  85.0 (77.0; 91.0) | | | Specificity [%] (95% CI):  98.0 (74.0; 90.0) | | |
|  | ***GRADE*** | Very low ⊕⊖⊖⊖ | | | Very low ⊕⊖⊖⊖ | | |
| **Method. quality**  **(mod AMSTAR2)** | **Overall** | High | | | | | |
| **Further investigations, sub- or sensitivity analyses** | **Index test** | | **Reference standard** | **Studies (Particip.)** | | **Sens. [%], 95% CI** | **Spec. [%], 95% CI** |
|  |  | |  |  | |  |  |
|  |  | |  |  | |  |  |
|  |  | |  |  | |  |  |
| **Conclusions of study authors** | *“The diagnostic accuracy of Doppler ultrasound is high, especially at discriminating between the presence or absence of significant carotid artery stenosis. This evidence, plus its less invasive nature, supports the early use of Doppler ultrasound for the detection of carotid artery stenosis.”* | | | | | | |

| **Liu (2023)**[189] **–Carotid artery occlusion (extracranial)** | | | | | | | |
| --- | --- | --- | --- | --- | --- | --- | --- |
| **Clinical context** | ***Patient population*** | Adults | | | | | |
|  | ***Clinical setting*** | Not documented (probably outpatient) | | | | | |
|  | ***Prevalence [%] (CI)*** | 57.3 (52.1; 62.5) | | | | | |
|  | ***Noteworthy incl. crit.*** | *-* | | | | | |
|  | ***Noteworthy excl. crit.*** | Occlusion of intracranial segment of the internal carotid artery (from the external aperture of carotid canal to the bifurcation of middle cerebral artery and anterior cerebral artery); Studies < 10 patients. | | | | | |
| **Index test(s)** | CEUS | | | | | | |
| **Reference standard(s)** | DSA, CTA, angiography, or surgery | | | | | | |
| **DTA-SR characteristics** | ***No. of studies*** | 8 | | | | | |
|  | ***No. of participants*** | 354 arteries | | | | | |
|  | ***Year range*** | 1999 - 2020 | | | | | |
|  | ***Quality assessment*** | QUADAS-2 (4 high risk of bias studies) | | | | | |
|  | ***Special remark*** | High applicability concerns | | | | | |
| **Diagnostic accuracy** | ***Measure*** | Sensitivity [%] (95% CI):  99.0 (83.0; 100) | | | Specificity [%] (95% CI):  97.0 (90.0; 99.0) | | |
|  | ***GRADE*** | Low ⊕⊕⊖⊖ | | | Moderate ⊕⊕⊖⊕ | | |
| **Method. quality**  **(mod AMSTAR2)** | **Overall** | Moderate | | | | | |
| **Further investigations, sub- or sensitivity analyses** | **Index test** | | **Reference standard** | **Studies (Particip.)** | | **Sens. [%], 95% CI** | **Spec. [%], 95% CI** |
|  |  | |  |  | |  |  |
|  |  | |  |  | |  |  |
|  |  | |  |  | |  |  |
| **Conclusions of study authors** | *“Our study shows that CEUS has a high sensitivity and specificity for the diagnosis of total carotid artery occlusion. It can not only accurately distinguish between occlusive and pseudo-occlusive lesions of carotid artery, but can also accurately evaluate the shape of the residual lumen of pseudo-occlusive vessels, which is conducive to the formulation of treatment plans.”* | | | | | | |

| **Wardlaw (2006)**[190] **- Carotid artery stenosis (>70%)** | | | | | | | |
| --- | --- | --- | --- | --- | --- | --- | --- |
| **Clinical context** | ***Patient population*** | Adults with symptoms consistent with TIA, minor stroke, amaurosis fugax or retinal artery occlusion | | | | | |
|  | ***Clinical setting*** | Not explicitly stated; likely outpatient or imaging departments | | | | | |
|  | ***Prevalence [%] (CI)*** | Not documented | | | | | |
|  | ***Noteworthy incl. crit.*** | Studies that compared duplex ultrasound with intra-arterial angiography | | | | | |
|  | ***Noteworthy excl. crit.*** | Studies of carotid imaging in trauma, cancer, healthy volunteers, children | | | | | |
| **Index test(s)** | Doppler ultrasound | | | | | | |
| **Reference standard(s)** | Intra-arterial angiography (IAA) | | | | | | |
| **DTA-SR characteristics** | ***No. of studies*** | 11 | | | | | |
|  | ***No. of participants*** | 316 | | | | | |
|  | ***Year range*** | 1989 - 2003 | | | | | |
|  | ***Quality assessment*** | QUADAS-2 (1 study with low quality) | | | | | |
|  | ***Special remark*** | No report of RoB per study | | | | | |
| **Diagnostic accuracy** | ***Measure*** | Sensitivity [%] (95% CI):  89.0 (85.0; 92.0) | | | Specificity [%] (95% CI):  84.0 (77.0; 89.0) | | |
|  | ***GRADE*** | Low ⊖⊕⊖⊕ | | | Very low ⊖⊕⊖⊖ | | |
| **Method. quality**  **(mod AMSTAR2)** | **Overall** | Low | | | | | |
| **Further investigations, sub- or sensitivity analyses** | **Index test** | | **Reference standard** | **Studies (Particip.)** | | **Sens. [%], 95% CI** | **Spec. [%], 95% CI** |
|  |  | |  |  | |  |  |
|  |  | |  |  | |  |  |
|  |  | |  |  | |  |  |
| **Conclusions of study authors** | “*Non-invasive tests, used cautiously, could replace intra-arterial carotid angiography for 70–99% stenosis”* | | | | | | |

| **Liu (2023)**[191] **- Stenosis or occlusion of any intracranial arteries** | | | | | | | |
| --- | --- | --- | --- | --- | --- | --- | --- |
| **Clinical context** | ***Patient population*** | Adults with acute ischemic stroke | | | | | |
|  | ***Clinical setting*** | Not documented | | | | | |
|  | ***Prevalence [%] (CI)*** | Not documented | | | | | |
|  | ***Noteworthy incl. crit.*** | Studies with patients with confirmed stroke by IA, CTA, MRA | | | | | |
|  | ***Noteworthy excl. crit.*** | - | | | | | |
| **Index test(s)** | Transcranial Doppler and transcranial color-coded Duplex | | | | | | |
| **Reference standard(s)** | IA, CTA, MRA | | | | | | |
| **DTA-SR characteristics** | ***No. of studies*** | 12 | | | | | |
|  | ***No. of participants*** | 812 | | | | | |
|  | ***Year range*** | 1989 - 2020 | | | | | |
|  | ***Quality assessment*** | QUADAS-2 (3 high risk of bias studies) | | | | | |
|  | ***Special remark*** |  | | | | | |
| **Diagnostic accuracy** | ***Measure*** | Sensitivity [%] (95% CI): 96.0 (88.0; 99.0) | | | Specificity [%] (95% CI):  94.0 (82.0; 98.0) | | |
|  | ***GRADE*** | Very low ⊕⊖⊖⊖ | | | Very low ⊕⊖⊖⊖ | | |
| **Method. quality**  **(mod AMSTAR2)** | **Overall** | Moderate | | | | | |
| **Further investigations, sub- or sensitivity analyses** | **Index test** | | **Reference standard** | **Studies (Particip.)** | | **Sens. [%], 95% CI** | **Spec. [%], 95% CI** |
|  | TCD | | IA, CTA, MRA | 8 | | 95 (88; 100) | 92 (82; 100) |
|  | TCCD | | IA, CTA, MRA | 4 | | 98 (95; 100) | 98 (93; 100) |
|  | Unchanged | | CTA | 7 | | 99 (97; 100) | 94 (86; 100) |
|  | Unchanged | | IAA, MRA | 5 | | 84 (77; 92) | 93 (81; 100) |
| **Conclusions of study authors** | *“TCD and TCCD in the detection of intracranial artery stenosis in patients with a history of stroke are comparable to the conventional detection methods of IA, CTA, and MRA. This study suggests that the selection of reference standards affects the results of sensitivity and specificity.”* | | | | | | |

| **Zhu (2025)**[193] – Intracranial artery | | | | | | | |
| --- | --- | --- | --- | --- | --- | --- | --- |
| **Clinical context** | ***Patient population*** | Adults | | | | | |
|  | ***Clinical setting*** | Not documented | | | | | |
|  | ***Prevalence [%] (CI)*** | Not documented | | | | | |
|  | ***Noteworthy incl. crit.*** | Patients with acute ischemic stroke | | | | | |
|  | ***Noteworthy excl. crit.*** | Case reports / series, unpublished grey literature | | | | | |
| **Index test(s)** | Transcranial Doppler and transcranial color-coded Duplex | | | | | | |
| **Reference standard(s)** | MRA, CTA, angiography, diagnosis at discharge | | | | | | |
| **DTA-SR characteristics** | ***No. of studies*** | 17 | | | | | |
|  | ***No. of participants*** | 1496 | | | | | |
|  | ***Year range*** | 1989 - 2019 | | | | | |
|  | ***Quality assessment*** | QUADAS-2, mixed risk of bias | | | | | |
|  | ***Special remark*** | - | | | | | |
| **Diagnostic accuracy** | ***Measure*** | Sensitivity [%] (95% CI):  90.0 (82.0; 95.0) | | | Specificity [%] (95% CI):  95.0 (91.0; 98.0) | | |
|  | ***GRADE*** | Very low ⊖⊖⊖⊖ | | | Low ⊖⊖⊖⊕ | | |
| **Method. quality**  **(mod AMSTAR2)** | **Overall** | Low | | | | | |
| **Further investigations, sub- or sensitivity analyses** | **Index test** | | **Reference standard** | **Studies (Particip.)** | | **Sens. [%], 95% CI** | **Spec. [%], 95% CI** |
|  | TCD / TCCD | | CTA, MRA, angiography | 11 (1180) | | 87 (81; 91) | 93 (87; 97) |
|  | TCD / TCCD | | CTA Angiography, hospital discharge | 6 (316) | | 98 (58; 100) | 97 (91; 99) |
|  |  | |  |  | |  |  |
|  |  | |  |  | |  |  |
| **Conclusions of study authors** | *“This review demonstrates the promise of transcranial ultrasonography as a potent tool in detecting stenosis in patients with acute ischemic stroke.”* | | | | | | |

| **Mattioni (2020)**[192] **- Stenosis or occlusion of any intracranial arteries** | | | | | | | |
| --- | --- | --- | --- | --- | --- | --- | --- |
| **Clinical context** | ***Patient population*** | Adults with acute ischemic stroke | | | | | |
|  | ***Clinical setting*** | Emergency department and specialist stroke or neurological units | | | | | |
|  | ***Prevalence [%] (CI)*** | 51.5 (47.1; 55.9) | | | | | |
|  | ***Noteworthy incl. crit.*** | Studies where all participants underwent imaging < 24h from onset | | | | | |
|  | ***Noteworthy excl. crit.*** | - | | | | | |
| **Index test(s)** | Transcranial Doppler (5 studies) and transcranial color-coded Duplex (4 studies) | | | | | | |
| **Reference standard(s)** | IA, CTA, MRA | | | | | | |
| **DTA-SR characteristics** | ***No. of studies*** | 9 | | | | | |
|  | ***No. of participants*** | 493 | | | | | |
|  | ***Year range*** | 1989 - 2016 | | | | | |
|  | ***Quality assessment*** | QUADAS-2 (4 high risk of bias studies) | | | | | |
|  | ***Special remark*** |  | | | | | |
| **Diagnostic accuracy** | ***Measure*** | Sensitivity [%] (95% CI): 95 (83; 99) | | | Specificity [%] (95% CI): 95 (90; 98) | | |
|  | ***GRADE*** | Very low ⊕⊖⊖⊖ | | | Moderate ⊕⊕⊖⊕ | | |
| **Method. quality**  **(mod AMSTAR2)** | **Overall** | High | | | | | |
| **Further investigations, sub- or sensitivity analyses** | **Index test** | | **Reference standard** | **Studies (Particip.)** | | **Sens. [%], 95% CI** | **Spec. [%], 95% CI** |
|  | Transcranial Doppler or color-coded Duplex (Only MCA) | | IA, CTA, MRA | 8 (393) | | 95 (82; 99) | 96 (90; 99) |
|  | Transcranial Doppler | | IA, CTA, MRA | 5 (NA) | | 90 (78; 96) | 95 (88; 98) |
|  | Transcranial color-coded Duplex | | IA, CTA, MRA | 4 (NA) | | 100 (32; 100) | 98 (40; 100) |
|  |  | |  |  | |  |  |
| **Conclusions of study authors** | *“This review provides evidence that transcranial Doppler or transcranial or color-coded Duplex, administered by professionals with adequate experience and skills, can provide useful diagnostic information for detecting stenosis or occlusion of intracranial vessels in people with acute ischemic stroke, or guide the request for more invasive vascular neuroimaging, especially where CT or MR-based vascular imaging are not immediately available.”* | | | | | | |

| **Jazayeri (2025)**[194] – Large vessel occlusion | | | | | | | |
| --- | --- | --- | --- | --- | --- | --- | --- |
| **Clinical context** | ***Patient population*** | Adults with ischemic stroke | | | | | |
|  | ***Clinical setting*** | Not documented | | | | | |
|  | ***Prevalence [%] (CI)*** | 28.0 (25.6; 30.4) | | | | | |
|  | ***Noteworthy incl. crit.*** | Ischemic stroke patients, non-invasive prehospital large vessel occlusion detection tools, comparison with CTA or MRA | | | | | |
|  | ***Noteworthy excl. crit.*** | Case reports/series <10 patients, non-original studies, pediatric populations | | | | | |
| **Index test(s)** | Transcranial Doppler and color-coded Duplex | | | | | | |
| **Reference standard(s)** | CTA, MRA | | | | | | |
| **DTA-SR characteristics** | ***No. of studies*** | 6 | | | | | |
|  | ***No. of participants*** | 1343 | | | | | |
|  | ***Year range*** | 2013 - 2020 | | | | | |
|  | ***Quality assessment*** | QUADAS-2 | | | | | |
|  | ***Special remark*** |  | | | | | |
| **Diagnostic accuracy** | ***Measure*** | Sensitivity [%] (95% CI):  87.5 (79.0; 92.8) | | | Specificity [%] (95% CI):  95.0 (91.8; 97.0) | | |
|  | ***GRADE*** | Very low ⊕⊖⊖⊖ | | | Low ⊕⊕⊖⊖ | | |
| **Method. quality**  **(mod AMSTAR2)** | **Overall** | Moderate | | | | | |
| **Further investigations, sub- or sensitivity analyses** | **Index test** | | **Reference standard** | **Studies (Particip.)** | | **Sens. [%], 95% CI** | **Spec. [%], 95% CI** |
|  |  | |  |  | |  |  |
|  |  | |  |  | |  |  |
|  |  | |  |  | |  |  |
|  |  | |  |  | |  |  |
| **Conclusions of study authors** | *“Non-invasive prehospital large vessel occlusion detection tools show promising diagnostic performance, particularly transcranial Doppler-based methods. Future studies should focus on validating these tools in larger, diverse populations to enhance prehospital stroke*  *triage and improve patient outcomes.”* | | | | | | |

| **Pinto-Villalba (2026)**[195] – Ischemic and hemorrhagic stroke | | | | | | | |
| --- | --- | --- | --- | --- | --- | --- | --- |
| **Clinical context** | ***Patient population*** | Adults | | | | | |
|  | ***Clinical setting*** | Stroke suspected in prehospital or in-hospital setting | | | | | |
|  | ***Prevalence [%] (CI)*** | Not documented | | | | | |
|  | ***Noteworthy incl. crit.*** | CTA served primarily to confirm stroke etiology or to exclude vascular mimics such as dissection or aneurysm, rather than to classify the stroke type (ischemic vs. hemorrhagic). | | | | | |
|  | ***Noteworthy excl. crit.*** | pediatric populations, animal studies, vasospasm assessments, and studies with <10 participants | | | | | |
| **Index test(s)** | Transcranial Doppler and color-coded Duplex | | | | | | |
| **Reference standard(s)** | CTA, MRA, DSA | | | | | | |
| **DTA-SR characteristics** | ***No. of studies*** | 13 | | | | | |
|  | ***No. of participants*** | 802 | | | | | |
|  | ***Year range*** | 1993 - 2022 | | | | | |
|  | ***Quality assessment*** | Study Quality Assessment Tools from the National Institutes of Health (NIH) | | | | | |
|  | ***Special remark*** |  | | | | | |
| **Diagnostic accuracy** | ***Measure*** | Sensitivity [%] (95% CI):  81.1 (73.1; 87.2) | | | Specificity [%] (95% CI):  85.5 (71.4; 93.3) | | |
|  | ***GRADE*** | Very low ⊖⊖⊖⊖ | | | Very low ⊖⊖⊖⊖ | | |
| **Method. quality (mod AMSTAR2)** | **Overall** | Low | | | | | |
| **Further investigations, sub- or sensitivity analyses** | **Index test** | | **Reference standard** | **Studies (Particip.)** | | **Sens. [%], 95% CI** | **Spec. [%], 95% CI** |
|  | TCCD (ischemic stroke) | | CTA, MRA, DSA | 8 (419) | | 77 (NA) | 88 (NA) |
|  | TCCD and POCUS (hemorrhagic stroke) | | CTA, MRA, DSA | 4 (267) | | 88 (65; 97) | 93 (72; 98) |
|  |  | |  |  | |  |  |
|  |  | |  |  | |  |  |
| **Conclusions of study authors** | *“Transcranial ultrasonography demonstrates good diagnostic accuracy for stroke detection. While operator-dependent and limited by acoustic window availability, these techniques, after thorough training and validation, may be prioritized in low-resource setting were computed tomography is not readily available through the means of formal capacitation and feasibility studies.”* | | | | | | |

| **Khan (2007)**[196] **- Vertebral artery stenosis (> 50 %)** | | | | | | | |
| --- | --- | --- | --- | --- | --- | --- | --- |
| **Clinical context** | ***Patient population*** | Adults | | | | | |
|  | ***Clinical setting*** | Not documented | | | | | |
|  | ***Prevalence [%] (CI)*** | Not documented | | | | | |
|  | ***Noteworthy incl. crit.*** | Studies that used IAA as reference standard to validate the accuracy of DUS, MRA and CTA to determine vertebral artery stenosis or occlusion. | | | | | |
|  | ***Noteworthy excl. crit.*** | No categorization of stenosis, merged vertebral artery data with other vessels | | | | | |
| **Index test(s)** | Color Doppler ultrasound | | | | | | |
| **Reference standard(s)** | Intra-arterial angiography (IAA) | | | | | | |
| **DTA-SR characteristics** | ***No. of studies*** | 2 | | | | | |
|  | ***No. of participants*** | 322 arteries | | | | | |
|  | ***Year range*** | 2001 - 2004 | | | | | |
|  | ***Quality assessment*** | No formal bias assessment | | | | | |
|  | ***Special remark*** |  | | | | | |
| **Diagnostic accuracy** | ***Measure*** | Sensitivity [%] (95% CI):  70.2 (54.2; 83.3) | | | Specificity [%] (95% CI):  97.7 (95.2; 99.1) | | |
|  | ***GRADE*** | Very low ⊖⊕⊖⊖ | | | Very low ⊖⊖⊖⊕ | | |
| **Method. quality**  **(mod AMSTAR2)** | **Overall** | Low | | | | | |
| **Further investigations, sub- or sensitivity analyses** | **Index test** | | **Reference standard** | **Studies (Particip.)** | | **Sens. [%], 95% CI** | **Spec. [%], 95% CI** |
|  | Duplex ultrasound without color (> 50% stenosis) | | Intra-arterial angiography | 3 (269) | | 70 (57; 82) | 93 (89; 96) |
|  | Duplex ultrasound without color (vertebral occlusion) | | Intra-arterial angiography | 3 (372) | | 99 (89; 100) | 91 (87; 94) |
|  |  | |  |  | |  |  |
|  |  | |  |  | |  |  |
| **Conclusions of study authors** | *“Our analysis suggests that Doppler ultrasound has a lower sensitivity than MRA and CTA. This is not surprising because ultrasound imaging cannot visualize the full length of the vertebral artery, and therefore detection of stenosis may have to rely on flow disturbance which is only present with more severe stenosis and does not directly show the site of stenosis. A further limitation of ultrasound is the difficulty of differentiating between dissection and atherosclerotic disease.”* | | | | | | |

###### Raised intracranial pressure

| **Aletreby (2022)**[197] **–** Raised intracranial pressure | | | | | | | |
| --- | --- | --- | --- | --- | --- | --- | --- |
| **Clinical context** | ***Patient population*** | Critically ill adults | | | | | |
|  | ***Clinical setting*** | Intensive care unit | | | | | |
|  | ***Prevalence [%] (CI)*** | Not documented | | | | | |
|  | ***Noteworthy incl. crit.*** | Studies of critically ill adults that measured ONSD by ultrasound and measured the ICP by an invasive procedure | | | | | |
|  | ***Noteworthy excl. crit.*** | Lumbar puncture measurements | | | | | |
| **Index test(s)** | Transorbital sonography (optic nerve sheath diameter) | | | | | | |
| **Reference standard(s)** | Invasive measurements of ICP | | | | | | |
| **DTA-SR characteristics** | ***No. of studies*** | 16 | | | | | |
|  | ***No. of participants*** | 619 | | | | | |
|  | ***Year range*** | 2007 - 2019 | | | | | |
|  | ***Quality assessment*** | QUADAS-2 (1 study with low quality) | | | | | |
|  | ***Special remark*** |  | | | | | |
| **Diagnostic accuracy** | ***Measure*** | Sensitivity [%] (95% CI):  90.0 (85.0; 94.0) | | | Specificity [%] (95% CI):  85.0 (80.0; 89.0) | | |
|  | ***GRADE*** | Moderate ⊖⊕⊕⊕ | | | Moderate ⊖⊕⊕⊕ | | |
| **Method. quality**  **(mod AMSTAR2)** | **Overall** | Low | | | | | |
| **Further investigations, sub- or sensitivity analyses** | **Index test** | | **Reference standard** | **Studies (Particip.)** | | **Sens. [%], 95% CI** | **Spec. [%], 95% CI** |
|  |  | |  |  | |  |  |
|  |  | |  |  | |  |  |
|  |  | |  |  | |  |  |
| ***Conclusions of study authors*** | *“Sonographically determined ONSD had reasonable performance indicators and a highly positive correlation with invasively measured ICP. The method is a useful adjunctive monitoring tool in emergency medicine and critical care, but it cannot replace invasive*  *methods as currently used in traditional situations.”* | | | | | | |

| **Bakola (2024)**[198] – Raised intracranial pressure | | | | | | | |
| --- | --- | --- | --- | --- | --- | --- | --- |
| **Clinical context** | ***Patient population*** | Adults and children idiopathic intracranial hypertension | | | | | |
|  | ***Clinical setting*** | Neurological department | | | | | |
|  | ***Prevalence [%] (CI)*** | 53.3 (49.8; 56.8) | | | | | |
|  | ***Noteworthy incl. crit.*** | Observational studies evaluating adult patients with idiopathic intracranial hypertension using transorbital sonography and obtaining values of optic nerve sheath diameter and/or optic disc elevation compared to controls | | | | | |
|  | ***Noteworthy excl. crit.*** | Noncontrolled studies, case series presenting less than 10 cases | | | | | |
| **Index test(s)** | Transorbital sonography (optic nerve sheath diameter and/or optic disc elevation) | | | | | | |
| **Reference standard(s)** | Follow-up | | | | | | |
| **DTA-SR characteristics** | ***No. of studies*** | 14 | | | | | |
|  | ***No. of participants*** | 779 | | | | | |
|  | ***Year range*** | 2011 – 2023 | | | | | |
|  | ***Quality assessment*** | QUADAS-2 (6 high risk of bias, 7 moderate risk of bias) | | | | | |
|  | ***Special remark*** | Case-control studies | | | | | |
| **Diagnostic accuracy** | ***Measure*** | Sensitivity [%] (95% CI):  85.5 (77.9; 90.8) | | | Specificity [%] (95% CI):  90.7 (84.6; 94.5) | | |
|  | ***GRADE*** | Very low ⊖⊖⊖⊖ | | | Very low ⊖⊖⊖⊕ | | |
| **Method. quality**  **(mod AMSTAR2)** | **Overall** | Low | | | | | |
| **Further investigations, sub- or sensitivity analyses** | **Index test** | | **Reference standard** | **Studies (Particip.)** | | **Sens. [%], 95% CI** | **Spec. [%], 95% CI** |
|  |  | |  |  | |  |  |
|  |  | |  |  | |  |  |
|  |  | |  |  | |  |  |
|  |  | |  |  | |  |  |
| **Conclusions of study authors** | *“In conclusion, transorbital sonography through optic nerve sheath diameter measurements has a high diagnostic utility for the noninvasive diagnosis of increased intracranial pressure in the context of idiopathic intracranial hypertension. Transorbital sonography represents a very promising bedside diagnostic tool that may deserve wider implementation in everyday clinical practice.”* | | | | | | |

| **Chen (2023)**[199] **–** Raised intracranial pressure | | | | | | | |
| --- | --- | --- | --- | --- | --- | --- | --- |
| **Clinical context** | ***Patient population*** | Adults | | | | | |
|  | ***Clinical setting*** | Not documented | | | | | |
|  | ***Prevalence [%] (CI)*** | Not documented | | | | | |
|  | ***Noteworthy incl. crit.*** | - | | | | | |
|  | ***Noteworthy excl. crit.*** | - | | | | | |
| **Index test(s)** | Transorbital sonography (optic nerve sheath diameter) | | | | | | |
| **Reference standard(s)** | Invasive measurements of intracranial pressure | | | | | | |
| **DTA-SR characteristics** | ***No. of studies*** | 8 | | | | | |
|  | ***No. of participants*** | 222 | | | | | |
|  | ***Year range*** | 2007 - 2020 | | | | | |
|  | ***Quality assessment*** | QUADAS-2 (Only low risk of bias studies) | | | | | |
|  | ***Special remark*** |  | | | | | |
| **Diagnostic accuracy** | ***Measure*** | Sensitivity [%] (95% CI):  82.0 (75.0; 88.0) | | | Specificity [%] (95% CI):  82.0 (71.0; 90.0) | | |
|  | ***GRADE*** | Low⊖⊕⊕⊖ | | | Low⊖⊕⊕⊖ | | |
| **Method. quality**  **(mod AMSTAR2)** | **Overall** | Low | | | | | |
| **Further investigations, sub- or sensitivity analyses** | **Index test** | | **Reference standard** | **Studies (Particip.)** | | **Sens. [%], 95% CI** | **Spec. [%], 95% CI** |
|  |  | |  |  | |  |  |
|  |  | |  |  | |  |  |
|  |  | |  |  | |  |  |
| **Conclusions of study authors** | *“An ultrasound‑determined elevated intracranial pressure has reasonable performance indicators with high sensitivity and specificity in patients with traumatic brain injury. As such, this method may be a useful complementary monitoring tool in acute care.”* | | | | | | |

| **Koziarz (2019)**[200] **–** Raised intracranial pressure | | | | | | | |
| --- | --- | --- | --- | --- | --- | --- | --- |
| **Clinical context** | ***Patient population*** | Adults and children | | | | | |
|  | ***Clinical setting*** | Not documented | | | | | |
|  | ***Prevalence [%] (CI)*** | Not documented | | | | | |
|  | ***Noteworthy incl. crit.*** | Prospective diagnostic test accuracy studies of optic nerve  ultrasonography for increased intracranial pressure | | | | | |
|  | ***Noteworthy excl. crit.*** | Patients presenting with globe trauma or ophthalmologic diseases | | | | | |
| **Index test(s)** | Transorbital sonography (optic nerve sheath diameter) | | | | | | |
| **Reference standard(s)** | CT, ICP Monitor, Lumbar puncture | | | | | | |
| **DTA-SR characteristics** | ***No. of studies*** | 71 | | | | | |
|  | ***No. of participants*** | 4551 | | | | | |
|  | ***Year range*** | 1994 – 2019 | | | | | |
|  | ***Quality assessment*** | QUADAS-2 (36 high risk of bias studies) | | | | | |
|  | ***Special remark*** |  | | | | | |
| **Diagnostic accuracy** | ***Measure*** | Sensitivity [%] (95% CI):  94.0 (91.0; 96.0) | | | Specificity [%] (95% CI):  87.0 (82.0; 91.0) | | |
|  | ***GRADE*** | Moderate ⊕⊖⊕⊕ | | | Moderate ⊕⊖⊕⊕ | | |
| **Method. quality**  **(mod AMSTAR2)** | **Overall** | High | | | | | |
| **Further investigations, sub- or sensitivity analyses** | **Index test** | | **Reference standard** | **Studies (Particip.)** | | **Sens. [%], 95% CI** | **Spec. [%], 95% CI** |
|  | OSND (Low risk of bias studies) | | CT, ICP Monitor, Lumbar puncture | 35 (NA) | | 95 (91; 97) | 89 (82; 93) |
|  | OSND (Emergency physician) | | CT, ICP Monitor, Lumbar puncture | NA | | 96 (88; 99) | 85 (69; 94) |
|  | OSND (Traumatic brain injury) | | CT, ICP Monitor, Lumbar puncture | 18 (NA) | | 97 (92; 99) | 86 (74; 93) |
|  | OSND (Atraumatic brain injury) | | CT, ICP Monitor, Lumbar puncture | 26 (NA) | | 92 (86; 96) | 86 (77; 92) |
| **Conclusions of study authors** | *“Optic nerve ultrasonography is an accurate diagnostic tool that may be useful for various patient populations and situations. It is noninvasive, quick, and easy to use and can enable health care professionals to triage patients with suspected increased intracranial pressure.”* | | | | | | |

| **Melo (2025)**[201] – Raised intracranial pressure | | | | | | | |
| --- | --- | --- | --- | --- | --- | --- | --- |
| **Clinical context** | ***Patient population*** | Adults | | | | | |
|  | ***Clinical setting*** | Not documented | | | | | |
|  | ***Prevalence [%] (CI)*** | Not documented | | | | | |
|  | ***Noteworthy incl. crit.*** | Patients with suspected increased ICP according to physical examination or imaging results | | | | | |
|  | ***Noteworthy excl. crit.*** | Traumatic brain injury, Case reports/series, preclinical studies | | | | | |
| **Index test(s)** | Optic nerve ultrasonography | | | | | | |
| **Reference standard(s)** | CT, MRI, IPP, IVC, lumbar puncture, external ventricular drainage | | | | | | |
| **DTA-SR characteristics** | ***No. of studies*** | 18 | | | | | |
|  | ***No. of participants*** | 1484 | | | | | |
|  | ***Year range*** | 2009 - 2024 | | | | | |
|  | ***Quality assessment*** | QUADAS-2 (overall low risk of bias) | | | | | |
|  | ***Special remark*** | No of participants doesn’t match 2x2 table | | | | | |
| **Diagnostic accuracy** | ***Measure*** | Sensitivity [%] (95% CI):  92.0 (85.0; 95.0) | | | Specificity [%] (95% CI):  90.0 (84.0; 94.0) | | |
|  | ***GRADE*** | Moderate ⊕⊕⊖⊕ | | | Moderate ⊕⊕⊖⊕ | | |
| **Method. quality**  **(mod AMSTAR2)** | **Overall** | Moderate | | | | | |
| **Further investigations, sub- or sensitivity analyses** | **Index test** | | **Reference standard** | **Studies (Particip.)** | | **Sens. [%], 95% CI** | **Spec. [%], 95% CI** |
|  | Optic nerve ultrasonography | | Invasive measurement | 9 (NA) | | 92 (85; 96) | 87 (80, 92) |
|  | Optic nerve ultrasonography | | Non-invasive measurement | 9 (NA) | | 91 (77; 97) | 92 (82; 97) |
|  |  | |  |  | |  |  |
|  |  | |  |  | |  |  |
| **Conclusions of study authors** | *“Our findings suggest that sonographic measurement of Optic nerve ultrasonography holds potential as a noninvasive technique for assessing elevated ICP in patients with nontraumatic brain injury. However, because of the heterogeneity of the conditions in the mixed patient poupulations studied, these results should not be extrapolated uncritically to specific etiologies. This underscores the need for large-scale studies in diverse, non-TBI populations to better define the role of ultrasound ONSD in diagnosing intracranial hypertension and its integration into clinical workflows.”* | | | | | | |

| **Wang (2026)**[202] – Raised intracranial pressure | | | | | | | |
| --- | --- | --- | --- | --- | --- | --- | --- |
| **Clinical context** | ***Patient population*** | Adults | | | | | |
|  | ***Clinical setting*** | Not documented | | | | | |
|  | ***Prevalence [%] (CI)*** | Not documented | | | | | |
|  | ***Noteworthy incl. crit.*** | Standardized ONSD measurement of 3 mm behind the globe | | | | | |
|  | ***Noteworthy excl. crit.*** | Studies with methodological constraints such as inadequate sample sizes (< 10 participants per group), nonstandardized optic nerve sheath diameter measurements | | | | | |
| **Index test(s)** | Optic nerve sheath diameter (5.1 mm defined as threshold by decision tree analysis) | | | | | | |
| **Reference standard(s)** | Invasive measurements of ICP, lumbar punction (cut-off 20mmHg) | | | | | | |
| **DTA-SR characteristics** | ***No. of studies*** | 42 | | | | | |
|  | ***No. of participants*** | 3213 | | | | | |
|  | ***Year range*** | 2009 - 2025 | | | | | |
|  | ***Quality assessment*** | QUADAS-2 | | | | | |
|  | ***Special remark*** |  | | | | | |
| **Diagnostic accuracy** | ***Measure*** | Sensitivity [%] (95% CI):  87.7 (83.1; 91.5) | | | Specificity [%] (95% CI):  86.3 (81.9; 90.1) | | |
|  | ***GRADE*** | Moderate ⊕⊖⊕⊕ | | | Moderate ⊕⊖⊕⊕ | | |
| **Method. quality (mod AMSTAR2)** | **Overall** | Moderate | | | | | |
| **Further investigations, sub- or sensitivity analyses** | **Index test** | | **Reference standard** | **Studies (Particip.)** | | **Sens. [%], 95% CI** | **Spec. [%], 95% CI** |
|  | Single plane | | ICP, lumbar punction | NA | | 87 (83; 92) | 86 (82; 90) |
|  | Traumatic brain injury | | ICP, lumbar punction | 11 (783) | | 85 (80; 90) | 84 (79; 88) |
|  | Non-traumatic brain injury | | ICP, lumbar punction | 31 (2430) | | 92 (88; 95) | 85 (81; 89) |
|  | Asian | | ICP, lumbar punction | 27 (2350) | | 88 (84; 91) | 85 (81; 88) |
|  | Non-Asian | | ICP, lumbar punction | 15 (863) | | 90 (83; 94) | 83 (77; 87) |
|  | High seniority of operator | | ICP, lumbar punction | NA | | 88 (83; 92) | 83 (78; 87) |
|  | Low seniority of operator | | ICP, lumbar punction | NA | | 87 (83; 96) | 82 (78; 86) |
| **Conclusions of study authors** | *“The findings from this study suggest that an ONSD threshold of ≥ 5.1 mm shows promise as a diagnostic marker for elevated ICP (> 20 cmH₂O/mmHg or acute etiology), although external validation is needed. Multiplane evaluation improves its predictive accuracy for favorable outcomes..”* | | | | | | |

| **Xu (2023)**[203] **–**Raised intracranial pressure | | | | | | | |
| --- | --- | --- | --- | --- | --- | --- | --- |
| **Clinical context** | ***Patient population*** | Critically ill adults after traumatic brain injury | | | | | |
|  | ***Clinical setting*** | Intensive care unit | | | | | |
|  | ***Prevalence [%] (CI)*** | Not documented | | | | | |
|  | ***Noteworthy incl. crit.*** | Only traumatic brain injury | | | | | |
|  | ***Noteworthy excl. crit.*** | Sample size less than 10. | | | | | |
| **Index test(s)** | Transorbital sonography (optic nerve sheath diameter) | | | | | | |
| **Reference standard(s)** | Invasive measurements of intracranial pressure | | | | | | |
| **DTA-SR characteristics** | ***No. of studies*** | 10 | | | | | |
|  | ***No. of participants*** | 512 | | | | | |
|  | ***Year range*** | 2007 - 2022 | | | | | |
|  | ***Quality assessment*** | QUADAS-2 (1 study with low quality) | | | | | |
|  | ***Special remark*** |  | | | | | |
| **Diagnostic accuracy** | ***Measure*** | Sensitivity [%] (95% CI):  85.0 (79.0; 89.0) | | | Specificity [%] (95% CI):  88.0 (80.0; 93.0) | | |
|  | ***GRADE*** | High⊕⊕⊕⊕ | | | Moderate ⊕⊕⊕⊖ | | |
| **Method. quality**  **(mod AMSTAR2)** | **Overall** | Moderate | | | | | |
| **Further investigations, sub- or sensitivity analyses** | **Index test** | | **Reference standard** | **Studies (Particip.)** | | **Sens. [%], 95% CI** | **Spec. [%], 95% CI** |
|  |  | |  |  | |  |  |
|  |  | |  |  | |  |  |
|  |  | |  |  | |  |  |
| **Conclusions of study authors** | “*For patients with traumatic brain injury, ONSD ultrasound is a potentially sensitive method to exclude elevated intracranial pressure in low-prevalence populations, as well as a specific testing method to determine elevated intracranial pressure in high prevalence traumatic brain injury populations.”* | | | | | | |

###### Neurology other

| **Boktor (2023)**[204] – Cauda equina syndrome | | | | | | | |
| --- | --- | --- | --- | --- | --- | --- | --- |
| **Clinical context** | ***Patient population*** | Adults with clinical suspicion of cauda equina syndrome | | | | | |
|  | ***Clinical setting*** | Not documented | | | | | |
|  | ***Prevalence [%] (CI)*** | 16.2 (12.9; 19.5) | | | | | |
|  | ***Noteworthy incl. crit.*** | Original studies, clinical suspicion of cauda equina syndrome, clinical findings which included post-void residual volume, confirmation or exclusion of cauda equina syndrome with cross-sectional imaging (e.g. MRI) | | | | | |
|  | ***Noteworthy excl. crit.*** | Studies reporting only urinary retention or bladder dysfunction without a clinical suspicion or imaging confirmation, post-void residual volume without a quantifiable volume | | | | | |
| **Index test(s)** | Post-void residual volume (200ml as threshold) | | | | | | |
| **Reference standard(s)** | MRI, CT | | | | | | |
| **DTA-SR characteristics** | ***No. of studies*** | 3 | | | | | |
|  | ***No. of participants*** | 487 | | | | | |
|  | ***Year range*** | 2019 - 2020 | | | | | |
|  | ***Quality assessment*** | QUADAS-2 (all low risk of bias) | | | | | |
|  | ***Special remark*** |  | | | | | |
| **Diagnostic accuracy** | ***Measure*** | Sensitivity [%] (95% CI):  82.3 (72.0; 90.0) | | | Specificity [%] (95% CI):  65.0 (60.0; 70.0) | | |
|  | ***GRADE*** | Very low ⊝⊝⊕⊝ | | | Low ⊝⊕⊕⊝ | | |
| **Method. quality**  **(mod AMSTAR2)** | **Overall** | Low | | | | | |
| **Further investigations, sub- or sensitivity analyses** | **Index test** | | **Reference standard** | **Studies (Particip.)** | | **Sens. [%], 95% CI** | **Spec. [%], 95% CI** |
|  | Post-void residual volume (100ml as threshold) | | CT, MRI | 1 (65) | | 67 (38; 88) | 52 (37; 66) |
|  | Post-void residual volume (300ml as threshold) | | CT, MRI | 4 (86) | | 80 (44; 97) | 59 (47; 70) |
|  | Post-void residual volume (500ml as threshold) | | CT, MRI | 1 (296) | | 38 (26; 51) | 77 (71; 82) |
|  |  | |  |  | |  |  |
| **Conclusions of study authors** | *“Amount of post-void residual volume is a useful tool when assessing patients in whom the diagnosis of cauda equina syndrome is suspected. Compared with other values an amount of post-void residual volume of 200 ml had superior sensitivity, specificity, and positive and negative predictive values. In a patient with a suggestive history and clinical findings, an amount of post-void residual of > 200 ml should further raise the suspicion of cauda equina syndrome. Caution is recommended when considering the amount of post-void residual in isolation and using it as an ‘exclusion tool’, and it should only be used as an adjunct to a full clinical assessment.”* | | | | | | |

| **Allen (2023)**[205] –Neurodegenerative disorder | | | | | | | |
| --- | --- | --- | --- | --- | --- | --- | --- |
| **Clinical context** | ***Patient population*** | Adults and children with suspected neurodegenerative disorder | | | | | |
|  | ***Clinical setting*** | Intensive care unit, neurology, emergency department | | | | | |
|  | ***Prevalence [%] (CI)*** | Not documented | | | | | |
|  | ***Noteworthy incl. crit.*** | RCTs, comparative cohort studies, case-control studies, case series with >3 patients and cross-sectional studies. | | | | | |
|  | ***Noteworthy excl. crit.*** | Fontanelle windows, skull disorders, ultrasound performed through surgical skull defects or craniectomy. | | | | | |
| **Index test(s)** | Transcranial ultrasound | | | | | | |
| **Reference standard(s)** | PET, SPECT, CT, MRI | | | | | | |
| **DTA-SR characteristics** | ***No. of studies*** | 20 (15 about Parkinsons’ disease 1 only about patients with Wilson’s and 1 study for each of amyotrophic lateral sclerosis,  Huntington’s disease, idiopathic rapid eye movement disorder and  progressive supranuclear palsy). | | | | | |
|  | ***No. of participants*** | 1402 | | | | | |
|  | ***Year range*** | 1999 - 2018 | | | | | |
|  | ***Quality assessment*** | QUADAS-2 (6 high risk of bias studies) | | | | | |
|  | ***Special remark*** |  | | | | | |
| **Diagnostic accuracy** | ***Measure*** | Sensitivity [%] (95% CI):  80.0 (72.0; 89.0) | | | Specificity [%] (95% CI):  71.0 (59.0; 82.0) | | |
|  | ***GRADE*** | Very Low ⊕⊖⊖⊖ | | | Very Low ⊕⊖⊖⊖ | | |
| **Method. quality**  **(mod AMSTAR2)** | **Overall** | Moderate | | | | | |
| **Further investigations, sub- or sensitivity analyses** | **Index test** | | **Reference standard** | **Studies (Particip.)** | | **Sens. [%], 95% CI** | **Spec. [%], 95% CI** |
|  | TCU (low risk of bias) | | PET, SPECT, CT, MRI | NA | | 79 (63; 95) | 80 (74; 87) |
|  |  | |  |  | |  |  |
|  |  | |  |  | |  |  |
| **Conclusions of study authors** | *“TCU has […] limited utility in detecting neurodegenerative disease”* | | | | | | |

| **Allen (2023)**[205] – Intracranial masses | | | | | | | |
| --- | --- | --- | --- | --- | --- | --- | --- |
| **Clinical context** | ***Patient population*** | Adults and children wit already diagnosed intracranial masses | | | | | |
|  | ***Clinical setting*** | Intensive care unit, neurology, emergency department | | | | | |
|  | ***Prevalence [%] (CI)*** | Not documented | | | | | |
|  | ***Noteworthy incl. crit.*** | RCTs, comparative cohort studies, case-control studies, case series with >3 patients and cross-sectional studies. | | | | | |
|  | ***Noteworthy excl. crit.*** | Fontanelle windows, skull disorders, ultrasound performed through surgical skull defects or craniectomy, germinalmatrix hemorrhages | | | | | |
| **Index test(s)** | Transcranial ultrasound | | | | | | |
| **Reference standard(s)** | CT, MRI | | | | | | |
| **DTA-SR characteristics** | ***No. of studies*** | 2 | | | | | |
|  | ***No. of participants*** | 113 | | | | | |
|  | ***Year range*** | 2008 | | | | | |
|  | ***Quality assessment*** | QUADAS-2 (no high risk of bias studies) | | | | | |
|  | ***Special remark*** |  | | | | | |
| **Diagnostic accuracy** | ***Measure*** | Sensitivity [%] (95% CI):  97.0 (92.0; 100) | | | Specificity [%] (95% CI):  99.0 (96.0, 100) | | |
|  | ***GRADE*** | Low ⊕⊖⊖⊕ | | | Low ⊕⊖⊖⊕ | | |
| **Method. quality**  **(mod AMSTAR2)** | **Overall** | Moderate | | | | | |
| **Further investigations, sub- or sensitivity analyses** | **Index test** | | **Reference standard** | **Studies (Particip.)** | | **Sens. [%], 95% CI** | **Spec. [%], 95% CI** |
|  |  | |  |  | |  |  |
|  |  | |  |  | |  |  |
|  |  | |  |  | |  |  |
| **Conclusions of study authors** | *“TCU has high sensitivity for the detection of the brain masses with low specificity. The heterogeneous nature of brainmasses included in the studies that varied in terms of size, vascularity, calcification, or echogenicity does not allow a meaningful clinical translation of the applicability of TCU in this setting”.* | | | | | | |

| **Allen (2023)**[205] –Intracranial hemorrhage | | | | | | | |
| --- | --- | --- | --- | --- | --- | --- | --- |
| **Clinical context** | ***Patient population*** | | Adults and children with mostly already diagnosed bleeding | | | | |
|  | ***Clinical setting*** | | Intensive care unit, neurology, emergency department | | | | |
|  | ***Prevalence [%] (CI)*** | | Not documented | | | | |
|  | ***Noteworthy incl. crit.*** | | RCTs, comparative cohort studies, case-control studies, case series with >3 patients and cross-sectional studies. | | | | |
|  | ***Noteworthy excl. crit.*** | | Fontanelle windows, skull disorders, ultrasound performed through surgical skull defects or craniectomy, germinalmatrix hemorrhages | | | | |
| **Index test(s)** | Transcranial ultrasound | | | | | | |
| **Reference standard(s)** | CT, MRI | | | | | | |
| **DTA-SR characteristics** | ***No. of studies*** | | 18 | | | | |
|  | ***No. of participants*** | | 803 | | | | |
|  | ***Year range*** | | 1991 - 2019 | | | | |
|  | ***Quality assessment*** | | QUADAS-2 (1 high risk of bias studies) | | | | |
|  | ***Special remark*** | |  | | | | |
| **Diagnostic accuracy** | ***Measure*** | | Sensitivity [%] (95% CI):  88.0 (74.0; 100) | | Specificity [%] (95% CI):  81.0 (50.0, 100) | | |
|  | ***GRADE*** | | Low ⊕⊖⊕⊖ | | Low ⊕⊖⊕⊖ | | |
| **Method. quality**  **(mod AMSTAR2)** | **Overall** | | Moderate | | | | |
| **Further investigations, sub- or sensitivity analyses** | **Index test** | **Reference standard** | | **Studies (Particip.)** | | **Sens. [%], 95% CI** | **Spec. [%], 95% CI** |
|  |  |  | |  | |  |  |
|  |  |  | |  | |  |  |
|  |  |  | |  | |  |  |
| **Conclusions of study authors** | *“TCU has a high sensitivity for acute lobar and basal ganglia intracranial hemorrhage less than 4 weeks old […]. Our meta-analysis revealed the best sensitivity, specificity, and accuracy of TCU for use in intracerebral hemorrhage. There was inconsistency in reporting intracranial hemorrhage features, which is likely to affect TCU’s accuracy in detecting intracranial hemorrhage”* | | | | | | |

##### Ocular

| **Gottlieb (2019)**[206] – Retinal detachment | | | | | | | |
| --- | --- | --- | --- | --- | --- | --- | --- |
| **Clinical context** | ***Patient population*** | Adults | | | | | |
|  | ***Clinical setting*** | Emergency department, radiology department, ophthalmology clinic | | | | | |
|  | ***Prevalence [%] (CI)*** | 21.4 (17.9; 25.0) | | | | | |
|  | ***Noteworthy incl. crit.*** | Portable ultrasound machine | | | | | |
|  | ***Noteworthy excl. crit.*** | Static ophthalmologic ultrasound devices | | | | | |
| **Index test(s)** | POCUS, emergency physician performed | | | | | | |
| **Reference standard(s)** | Ophthalmologist exam, CT | | | | | | |
| **DTA-SR characteristics** | ***No. of studies*** | 6 | | | | | |
|  | ***No. of participants*** | 517 | | | | | |
|  | ***Year range*** | 2011 - 2018 | | | | | |
|  | ***Quality assessment*** | QUADAS-2 (unclear risk of bias for patient selection and reference standard) | | | | | |
|  | ***Special remark*** | - | | | | | |
| **Diagnostic accuracy** | ***Measure*** | Sensitivity [%] (95% CI):  92.0 (67.2; 98.5) | | | Specificity [%] (95% CI):  91.4 (84.9; 95.3) | | |
|  | ***GRADE*** | Low ⊕⊖⊕⊖ | | | Low ⊕⊖⊕⊖ | | |
| **Method. quality**  **(mod AMSTAR2)** | **Overall** | Moderate | | | | | |
| **Further investigations, sub- or sensitivity analyses** | **Index test** | | **Reference standard** | **Studies (Particip.)** | | **Sens. [%], 95% CI** | **Spec. [%], 95% CI** |
|  | POCUS, overall | | Ophthalmologist exam, CT, surgical findings, clinical FUP | 11 (844) | | 94 (78; 99) | 96 (89; 99) |
|  | POCUS, non emergency physician performed | | Ophthalmologist exam, CT, surgical findings, clinical FUP | 4 (227) | | 91 (68; 98) | 99 (82; 100) |
|  | POCUS, emergency department patients | | Ophthalmologist exam, CT, surgical findings, clinical FUP | 5 (455) | | 94 (79; 99) | 92 (86; 96) |
|  | POCUS, non emergency department patients | | Ophthalmologist exam, CT, surgical findings, clinical FUP | 3 (133) | | 74 (61; 85) | 85 (75; 92) |
| **Conclusions of study authors** | *“POCUS ocular ultrasound is both sensitive and specific for the diagnosis of retinal detachment. This may be a valuable adjunct when ophthalmology is not immediately available.”* | | | | | | |

| **Propst (2019)**[207] – Posterior chamber abnormalities | | | | | | | |
| --- | --- | --- | --- | --- | --- | --- | --- |
| **Clinical context** | ***Patient population*** | Adults | | | | | |
|  | ***Clinical setting*** | Emergency department | | | | | |
|  | ***Prevalence [%] (CI)*** | 17.1 (14.9; 19.2) | | | | | |
|  | ***Noteworthy incl. crit.*** | Emergency physician-performed POCUS, ophthalmologist exam as reference standard, sufficient data for 2x2 table | | | | | |
|  | ***Noteworthy excl. crit.*** | Patients < 18y, non-emergency sonographers, case series of fewer than 10 patients | | | | | |
| **Index test(s)** | POCUS, retinal detachment | | | | | | |
| **Reference standard(s)** | Ophthalmologist exam | | | | | | |
| **DTA-SR characteristics** | ***No. of studies*** | 9 | | | | | |
|  | ***No. of participants*** | 1189 (eyes) | | | | | |
|  | ***Year range*** | 2002 - 2019 | | | | | |
|  | ***Quality assessment*** | QUADAS-2; all studies of unclear or high risk of bias | | | | | |
|  | ***Special remark*** | Sensitivity analysis without significant change in sensitivity/specificity | | | | | |
| **Diagnostic accuracy** | ***Measure*** | Sensitivity [%] (95% CI):  94.0 (88.0; 97.0) | | | Specificity [%] (95% CI):  94.0 (85.0; 98.0) | | |
|  | ***GRADE*** | Moderate ⊕⊖⊕⊕ | | | Low ⊕⊖⊕⊖ | | |
| **Method. quality**  **(mod AMSTAR2)** | **Overall** | Moderate | | | | | |
| **Further investigations, sub- or sensitivity analyses** | **Index test** | | **Reference standard** | **Studies (Particip.)** | | **Sens. [%], 95% CI** | **Spec. [%], 95% CI** |
|  | POCUS, vitreous haemorrhage | | Ophthalmologist exam | 5 (739) | | 90 (65; 98) | 92 (75; 98) |
|  | POCUS, vitreous detachment | | Ophthalmologist exam | 4 (388) | | 67 (51; 81) | 89 (53; 98) |
|  | POCUS, Lens dislocation | | Ophthalmologist exam | 2 (400) | | 97 (83; 99) | 99 (97; 100) |
|  | POCUS, foreign body | | Ophthalmologist exam | 2 (400) | | 100  (81; 100) | 99 (99; 100) |
|  | POCUS, globe rupture | | Ophthalmologist exam | 2 (400) | | 100  (63; 100) | 99 (99; 100) |
| **Conclusions of study authors** | *“Trained emergency physician performs ocular POCUS with high enough sensitivity and specificity to rule out retinal detachment in low-risk patients and to rule in the diagnosis in those at high risk. Limited data suggest that emergency physician-performed POCUS is moderately accurate for diagnosing vitreous hemorrhage and poorly accurate for diagnosing vitreous detachment. Preliminary data suggest that emergency physician-performed POCUS is highly accurate to diagnose lens dislocation, intraocular foreign body, and globe rupture, but further evidence is required before confidently recommending its routine use for these indications.”* | | | | | | |

| **Wu (2025)**[208] – Posterior segment ocular disorder | | | | | | | |
| --- | --- | --- | --- | --- | --- | --- | --- |
| **Clinical context** | ***Patient population*** | Adults and children | | | | | |
|  | ***Clinical setting*** | Not documented | | | | | |
|  | ***Prevalence [%] (CI)*** | Not documented | | | | | |
|  | ***Noteworthy incl. crit.*** | - | | | | | |
|  | ***Noteworthy excl. crit.*** | Case reports, sample size fewer than 10 participants | | | | | |
| **Index test(s)** | B-mode ultrsound, any posterior segment ocular disorder (retinal detachment, vitreous hemorrhage, posterior vitreous detachment and choroidal detachment) | | | | | | |
| **Reference standard(s)** | Direct ophthalmoscopy | | | | | | |
| **DTA-SR characteristics** | ***No. of studies*** | 10 | | | | | |
|  | ***No. of participants*** | 1281 | | | | | |
|  | ***Year range*** | 2009 - 2024 | | | | | |
|  | ***Quality assessment*** | QUADAS-2 (6 low, 2 moderate, 2 high risk of bias) | | | | | |
|  | ***Special remark*** | Includes one pediatric and one mixed population | | | | | |
| **Diagnostic accuracy** | ***Measure*** | Sensitivity [%] (95% CI):  96.0 (91.0; 98.0) | | | Specificity [%] (95% CI):  94.0 (87.0; 98.0) | | |
|  | ***GRADE*** | Moderate ⊕⊖⊕⊕ | | | Low ⊕⊖⊕⊖ | | |
| **Method. quality**  **(mod AMSTAR2)** | **Overall** | Moderate | | | | | |
| **Further investigations, sub- or sensitivity analyses** | **Index test** | | **Reference standard** | **Studies (Particip.)** | | **Sens. [%], 95% CI** | **Spec. [%], 95% CI** |
|  |  | |  |  | |  |  |
|  |  | |  |  | |  |  |
|  |  | |  |  | |  |  |
|  |  | |  |  | |  |  |
| **Conclusions of study authors** | *“This meta-analysis underscores the high diagnostic accuracy of B-scan ultrasound for detecting posterior segment ocular disorders, evidenced by its high sensitivity, specificity, and diagnostic odds ratio. The results advocate for its inclusion in diagnostic protocols, especially in settings where advanced imaging techniques may not be available.”* | | | | | | |

##### Respiratory

###### Disorders of the pleural space

| **Staub (2018)**[209] – Haemothorax | | | | | | | |
| --- | --- | --- | --- | --- | --- | --- | --- |
| **Clinical context** | ***Patient population*** | Adult trauma patients | | | | | |
|  | ***Clinical setting*** | Emergency department | | | | | |
|  | ***Prevalence [%] (CI)*** | 19.1 (15.5; 22.7) | | | | | |
|  | ***Noteworthy incl. crit.*** | Adult trauma patients on emergency department, ultrasonography with description of the sonographic sign, | | | | | |
|  | ***Noteworthy excl. crit.*** | Studies using units of analysis other than the hemithorax and patient (e.g. chest region) | | | | | |
| **Index test(s)** | Lung ultrasound (anechoic area in pleural space, free fluid in the pleural space) | | | | | | |
| **Reference standard(s)** | CT, chest X-ray, chest tube observation | | | | | | |
| **DTA-SR characteristics** | ***No. of studies*** | 3 | | | | | |
|  | ***No. of participants*** | 451 | | | | | |
|  | ***Year range*** | 1995 - 2014 | | | | | |
|  | ***Quality assessment*** | QUADAS-2 | | | | | |
|  | ***Special remark*** |  | | | | | |
| **Diagnostic accuracy** | ***Measure*** | Sensitivity [%] (95% CI):  81.0 (71.0; 88.0) | | | Specificity [%] (95% CI):  99.0 (96.0; 100.0) | | |
|  | ***GRADE*** | Moderate ⊕⊕⊕⊖ | | | High ⊕⊕⊕⊕ | | |
| **Method. quality**  **(mod AMSTAR2)** | **Overall** | Moderate | | | | | |
| **Further investigations, sub- or sensitivity analyses** | **Index test** | | **Reference standard** | **Studies (Particip.)** | | **Sens. [%], 95% CI** | **Spec. [%], 95% CI** |
|  |  | |  |  | |  |  |
|  |  | |  |  | |  |  |
|  |  | |  |  | |  |  |
|  |  | |  |  | |  |  |
| **Conclusions of study authors** | “*Notwithstanding the limitations of the included studies, this systematic review and metaanalysis suggested that chest ultrasonography is an accurate tool for the diagnostic assessment of traumatic pneumothorax and haemothorax in adults.”* | | | | | | |

| **Alrajhi (2012)**[210] – Iatrogenic pneumothorax | | | | | | | |
| --- | --- | --- | --- | --- | --- | --- | --- |
| **Clinical context** | ***Patient population*** | Adults | | | | | |
|  | ***Clinical setting*** | Emergency Department | | | | | |
|  | ***Prevalence [%] (CI)*** | Not documented | | | | | |
|  | ***Noteworthy incl. crit.*** | - | | | | | |
|  | ***Noteworthy excl. crit.*** | Pediatric populations | | | | | |
| **Index test(s)** | Lung ultrasound | | | | | | |
| **Reference standard(s)** | CT, release of air on chest tube insertion | | | | | | |
| **DTA-SR characteristics** | ***No. of studies*** | 2 | | | | | |
|  | ***No. of participants*** | 281 | | | | | |
|  | ***Year range*** | 2005, 2006 | | | | | |
|  | ***Quality assessment*** | QUADAS; moderate to high quality, detailed information not available | | | | | |
|  | ***Special remark*** | One study investigating radiologists, one with unknown operator | | | | | |
| **Diagnostic accuracy** | ***Measure*** | Sensitivity [%] (95% CI):  92.6 (83.5; 96.8) | | | Specificity [%] (95% CI):  96.6 (92.8; 98.5) | | |
|  | ***GRADE*** | Very low ⊖⊖⊕⊖ | | | Low ⊖⊖⊕⊕ | | |
| **Method. quality**  **(mod AMSTAR2)** | **Overall** | Low | | | | | |
| **Further investigations, sub- or sensitivity analyses** | **Index test** | | **Reference standard** | **Studies (Particip.)** | | **Sens. [%], 95% CI** | **Spec. [%], 95% CI** |
|  | Lung ultrasound; any cause | | CT, release of air on chest tube insertion | 8 (1048) | | 91 (87; 94) | 98 (97; 99) |
|  | Lung ultrasound; traumatic | | CT, release of air on chest tube insertion | 6 (767) | | 90 (85; 94) | 99 (97; 100) |
|  |  | |  |  | |  |  |
|  |  | |  |  | |  |  |
| **Conclusions of study authors** | *"This study demonstrates that chest ultrasonography can help recognize pneumothoraces with a sensitivity significantly superior and a specificity clinically similar to that of supine chest radiography. Our findings support the adoption of chest ultrasonography for routine use in patients with clinically suspected pneumothoraces.”* | | | | | | |

| **Bouillon-Minois (2025)**[211] – Pneumothorax | | | | | | | |
| --- | --- | --- | --- | --- | --- | --- | --- |
| **Clinical context** | ***Patient population*** | Adults after trauma | | | | | |
|  | ***Clinical setting*** | Emergency department | | | | | |
|  | ***Prevalence [%] (CI)*** | 27.6 (20.9; 34.3) | | | | | |
|  | ***Noteworthy incl. crit.*** | Studies performing lung ultrasound and chest X-ray comparing to CT-scan as a gold standard in the diagnosis of pneumothorax. | | | | | |
|  | ***Noteworthy excl. crit.*** | Articles without description about the ultrasound examination and the imaging results. | | | | | |
| **Index test(s)** | Lung ultrasound with signs of lung sliding, comet-tail artifact, lung point par emergency physician | | | | | | |
| **Reference standard(s)** | CT | | | | | | |
| **DTA-SR characteristics** | ***No. of studies*** | 15 | | | | | |
|  | ***No. of participants*** | 3171 | | | | | |
|  | ***Year range*** | 2005 - 2023 | | | | | |
|  | ***Quality assessment*** | QUADAS-2 (13 high risk of bias) | | | | | |
|  | ***Special remark*** |  | | | | | |
| **Diagnostic accuracy** | ***Measure*** | Sensitivity [%] (95% CI):  79.4 (68.2; 90.7) | | | Specificity [%] (95% CI):  99.5 (99.0; 100) | | |
|  | ***GRADE*** | Very low ⊝⊝⊕⊝ | | | Low ⊝⊝⊕⊕ | | |
| **Method. quality**  **(mod AMSTAR2)** | **Overall** | Low | | | | | |
| **Further investigations, sub- or sensitivity analyses** | **Index test** | | **Reference standard** | **Studies (Particip.)** | | **Sens. [%], 95% CI** | **Spec. [%], 95% CI** |
|  |  | |  |  | |  |  |
|  |  | |  |  | |  |  |
|  |  | |  |  | |  |  |
|  |  | |  |  | |  |  |
| **Conclusions of study authors** | *“In this meta-analysis, emergency healthcare workers performed chest ultrasound was better than chest X-ray for the detection of a pneumothorax in emergency departments patients.”* | | | | | | |

| **Ebrahimi (2014)**[212] – Pneumothorax | | | | | | | |
| --- | --- | --- | --- | --- | --- | --- | --- |
| **Clinical context** | ***Patient population*** | Adult trauma patients | | | | | |
|  | ***Clinical setting*** | Emergency department | | | | | |
|  | ***Prevalence [%] (CI)*** | Not documented | | | | | |
|  | ***Noteworthy incl. crit.*** | Adult trauma patients on emergency department, ultrasonography with description of the sonographic sign, | | | | | |
|  | ***Noteworthy excl. crit.*** | Studies using units of analysis other than the hemithorax and patient (e.g. chest region) | | | | | |
| **Index test(s)** | Lung ultrasound with signs of lung sliding, comet-tail artifact, lung point par emergency physician | | | | | | |
| **Reference standard(s)** | CT | | | | | | |
| **DTA-SR characteristics** | ***No. of studies*** | 14 | | | | | |
|  | ***No. of participants*** | 2495 | | | | | |
|  | ***Year range*** | 1999 - 2014 | | | | | |
|  | ***Quality assessment*** | QUADAS-2 (excluded all low value studies) | | | | | |
|  | ***Special remark*** | Subanalysis | | | | | |
| **Diagnostic accuracy** | ***Measure*** | Sensitivity [%] (95% CI):  88.0 (82.0; 94.0) | | | Specificity [%] (95% CI):  99.0 (98.0; 100.0) | | |
|  | ***GRADE*** | Very low ⊖⊖⊕⊖ | | | Very low ⊖⊖⊕⊖ | | |
| **Method. quality**  **(mod AMSTAR2)** | **Overall** | Low | | | | | |
| **Further investigations, sub- or sensitivity analyses** | **Index test** | | **Reference standard** | **Studies (Particip.)** | | **Sens. [%], 95% CI** | **Spec. [%], 95% CI** |
|  | Lung ultrasound (lung sliding, comet-tail artifact, lung point) | | CT | 28 (5314) | | 87 (81; 92) | 99 (98;99) |
|  |  | |  |  | |  |  |
|  |  | |  |  | |  |  |
|  |  | |  |  | |  |  |
| **Conclusions of study authors** | *“The present meta-analysis showed that the diagnostic accuracy of chest ultrasound was higher than supine chest X-ray for detection of pneumothorax. It seems that chest ultrasound is superior to chest X-ray in detection of pneumothorax, even after adjusting for possible sources of heterogeneity.”* | | | | | | |

| **Ranganath (2026)**[213] – Pneumothorax | | | | | | | |
| --- | --- | --- | --- | --- | --- | --- | --- |
| **Clinical context** | ***Patient population*** | Mixed, but mostly adults | | | | | |
|  | ***Clinical setting*** | Acute care and emgergency department | | | | | |
|  | ***Prevalence [%] (CI)*** | 14% | | | | | |
|  | ***Noteworthy incl. crit.*** | - | | | | | |
|  | ***Noteworthy excl. crit.*** | - | | | | | |
| **Index test(s)** | POCUS (eFAST or two points ultrasound of the lung) | | | | | | |
| **Reference standard(s)** | CT, chest X-ray | | | | | | |
| **DTA-SR characteristics** | ***No. of studies*** | 15 | | | | | |
|  | ***No. of participants*** | 3855 | | | | | |
|  | ***Year range*** | 2006 - 2025 | | | | | |
|  | ***Quality assessment*** | QUADAS (11 low risk, 4 high risk) | | | | | |
|  | ***Special remark*** |  | | | | | |
| **Diagnostic accuracy** | ***Measure*** | Sensitivity [%] (95% CI):  97.4 (91.3; 99.3) | | | Specificity [%] (95% CI):  93.6 (83.5; 97.7) | | |
|  | ***GRADE*** | Low ⊖⊖⊕⊕ | | | Low ⊖⊕⊕⊖ | | |
| **Method. quality**  **(mod AMSTAR2)** | **Overall** | Low | | | | | |
| **Further investigations, sub- or sensitivity analyses** | **Index test** | | **Reference standard** | **Studies (Particip.)** | | **Sens. [%], 95% CI** | **Spec. [%], 95% CI** |
|  |  | |  |  | |  |  |
|  |  | |  |  | |  |  |
|  |  | |  |  | |  |  |
|  |  | |  |  | |  |  |
| **Conclusions of study authors** | *“This systematic review and meta-analysis demonstrate that thoracic POCUS has high pooled sensitivity and specificity for the detection of pneumothorax in acute care settings. These findings are consistent with previous systematic reviews showing superior sensitivity of ultrasound compared with supine chest radiography, while maintaining high specificity”* | | | | | | |

| **Staub (2018)**[209] – Pneumothorax | | | | | | | |
| --- | --- | --- | --- | --- | --- | --- | --- |
| **Clinical context** | ***Patient population*** | Adult trauma patients | | | | | |
|  | ***Clinical setting*** | Emergency department | | | | | |
|  | ***Prevalence [%] (CI)*** | 19.8 (18.0; 21.6) | | | | | |
|  | ***Noteworthy incl. crit.*** | Adult trauma patients on emergency department, ultrasonography with description of the sonographic sign, | | | | | |
|  | ***Noteworthy excl. crit.*** | Studies using units of analysis other than the hemithorax and patient (e.g. chest region) | | | | | |
| **Index test(s)** | Lung ultrasound (absence of lung sliding and comet tail artefacts) | | | | | | |
| **Reference standard(s)** | CT, chest X-ray, chest tube observation | | | | | | |
| **DTA-SR characteristics** | ***No. of studies*** | 13 | | | | | |
|  | ***No. of participants*** | 1942 | | | | | |
|  | ***Year range*** | Not documented | | | | | |
|  | ***Quality assessment*** | QUADAS-2 | | | | | |
|  | ***Special remark*** |  | | | | | |
| **Diagnostic accuracy** | ***Measure*** | Sensitivity [%] (95% CI):  81.0 (71.0; 88.0) | | | Specificity [%] (95% CI):  98.0 (97.0; 99.0) | | |
|  | ***GRADE*** | Low ⊕⊖⊕⊖ | | | High ⊕⊕⊕⊕ | | |
| **Method. quality**  **(mod AMSTAR2)** | **Overall** | Moderate | | | | | |
| **Further investigations, sub- or sensitivity analyses** | **Index test** | | **Reference standard** | **Studies (Particip.)** | | **Sens. [%], 95% CI** | **Spec. [%], 95% CI** |
|  |  | |  |  | |  |  |
|  |  | |  |  | |  |  |
|  |  | |  |  | |  |  |
|  |  | |  |  | |  |  |
| **Conclusions of study authors** | “*Notwithstanding the limitations of the included studies, this systematic review and meta-analysis suggested that chest ultrasonography is an accurate tool for the diagnostic assessment of traumatic pneumothorax and haemothorax in adults.”* | | | | | | |

| **Grimberg (2010)**[214] – Pleural effusion | | | | | | | |
| --- | --- | --- | --- | --- | --- | --- | --- |
| **Clinical context** | ***Patient population*** | Various (trauma victims, exacerbated heart failure, hospitalized patients with acute respiratory distress syndrome) | | | | | |
|  | ***Clinical setting*** | Various (1 study with emergency setting) | | | | | |
|  | ***Prevalence [%] (CI)*** | 27.6 (24.8; 30.6) | | | | | |
|  | ***Noteworthy incl. crit.*** | - | | | | | |
|  | ***Noteworthy excl. crit.*** | - | | | | | |
| **Index test(s)** | Lung ultrasound | | | | | | |
| **Reference standard(s)** | CT, thoracic drainage | | | | | | |
| **DTA-SR characteristics** | ***No. of studies*** | 4 | | | | | |
|  | ***No. of participants*** | 924 | | | | | |
|  | ***Year range*** | 1997 - 2008 | | | | | |
|  | ***Quality assessment*** | QUADAS | | | | | |
|  | ***Special remark*** | - | | | | | |
| **Diagnostic accuracy** | ***Measure*** | Sensitivity [%] (95% CI):  93.0 (89.0; 96.0) | | | Specificity [%] (95% CI):  96.0 (95.0; 98.0) | | |
|  | ***GRADE*** | Moderate ⊖⊕⊕⊕ | | | Moderate ⊖⊕⊕⊕ | | |
| **Method. quality**  **(mod AMSTAR2)** | **Overall** | Critically low | | | | | |
| **Further investigations, sub- or sensitivity analyses** | **Index test** | | **Reference standard** | **Studies (Particip.)** | | **Sens. [%], 95% CI** | **Spec. [%], 95% CI** |
|  |  | |  |  | |  |  |
|  |  | |  |  | |  |  |
|  |  | |  |  | |  |  |
|  |  | |  |  | |  |  |
| **Conclusions of study authors** | *“Sonography, which is a portable low-cost radiation-free method, showed consistently high sensitivity, specificity and accuracy in detecting fluid in the pleural space, in different populations and clinical settings.”* | | | | | | |

| **Zaki (2024)**[134] – Pleural effusion | | | | | | | |
| --- | --- | --- | --- | --- | --- | --- | --- |
| **Clinical context** | ***Patient population*** | Not documented | | | | | |
|  | ***Clinical setting*** | Not documented | | | | | |
|  | ***Prevalence [%] (CI)*** | Not documented | | | | | |
|  | ***Noteworthy incl. crit.*** | Studies comparing POCUS to chest X-ray or assessed the role of these imaging tests in the diagnosis of pleural effusion | | | | | |
|  | ***Noteworthy excl. crit.*** | Studies investigating underlying diseases associated with pleural effusion | | | | | |
| **Index test(s)** | POCUS of the lung | | | | | | |
| **Reference standard(s)** | CT, chest X-ray | | | | | | |
| **DTA-SR characteristics** | ***No. of studies*** | 12 | | | | | |
|  | ***No. of participants*** | 653 | | | | | |
|  | ***Year range*** | 2001 – 2022 | | | | | |
|  | ***Quality assessment*** | QUADAS-2 | | | | | |
|  | ***Special remark*** |  | | | | | |
| **Diagnostic accuracy** | ***Measure*** | Sensitivity [%] (95% CI):  94.5 (91.7; 97.3) | | | Specificity [%] (95% CI):  97.9 (95.8; 100) | | |
|  | ***GRADE*** | Moderate ⊕⊕⊖⊕ | | | Moderate ⊕⊕⊖⊕ | | |
| **Method. quality**  **(mod AMSTAR2)** | **Overall** | Moderate | | | | | |
| **Further investigations, sub- or sensitivity analyses** | **Index test** | | **Reference standard** | **Studies (Particip.)** | | **Sens. [%], 95% CI** | **Spec. [%], 95% CI** |
|  | POCUS by experienced investigators | | CT, chest X-ray | 10 | | 94 (91; 98) | 98 (97; 100) |
|  | POCUS by inexperienced investigators | | CT, chest X-ray | 2 | | 94 (85; 100) | 86 (57; 100) |
|  | POCUS with pocket-size ultrasound | | CT, chest X-ray | 2 | | 96 (91; 100) | 86 (57; 100) |
|  | POCUS with usual ultrasound | | CT, chest X-ray | 10 | | 94 (91; 97) | 98 (97; 100) |
|  | POCUS ultrasound of the lung | | CT | 8 | | 95 (91; 98) | 98 (96; 100) |
|  | POCUS ultrasound of the lung | | Chest X-ray | 4 | | 94 (90; 99) | 95 (87; 100) |
| **Conclusions of study authors** | *“POCUS as an imaging modality has higher diagnostic accuracy than chest X-ray in detecting pleural effusion. Moreover, the accuracy is still high even when performed by physicians with less POCUS training. Therefore, we suggest it is considered a first-line imaging tool for diagnosing pleural effusion at the patients’ bedside.”* | | | | | | |

###### Infectious lung disease

| **Ebrahimzadeh (2022)**[216] – COVID-19 | | | | | | | |
| --- | --- | --- | --- | --- | --- | --- | --- |
| **Clinical context** | ***Patient population*** | Children or adults suspected of having COVID-19 | | | | | |
|  | ***Clinical setting*** | Not documented | | | | | |
|  | ***Prevalence [%] (CI)*** | 48.0 (46.0; 50.0) | | | | | |
|  | ***Noteworthy incl. crit.*** | Studies assessing ultrasound of the lungs for the diagnosis of COVID-19 and use a reference standard that included RT-PCR | | | | | |
|  | ***Noteworthy excl. crit.*** | Studies with image as part of the reference standard | | | | | |
| **Index test(s)** | Lung ultrasound | | | | | | |
| **Reference standard(s)** | RT-PCR test for SARS-CoV-2, positive WHO criteria, positive China CDC criteria or positive serology for SARS-CoV-2 antibodies | | | | | | |
| **DTA-SR characteristics** | ***No. of studies*** | 15 | | | | | |
|  | ***No. of participants*** | 2410 | | | | | |
|  | ***Year range*** | 2020 - 2021 | | | | | |
|  | ***Quality assessment*** | QUADAS-2 (10 studies with high risk of bias) | | | | | |
|  | ***Special remark*** |  | | | | | |
| **Diagnostic accuracy** | ***Measure*** | Sensitivity [%] (95% CI):  88.9 (84.9; 92.0) | | | Specificity [%] (95% CI):  72.2 (58.8; 82.5) | | |
|  | ***GRADE*** | Low ⊕⊖⊖⊕ | | | Very low ⊕⊖⊖⊖ | | |
| **Method. quality**  **(mod AMSTAR2)** | **Overall** | Moderate | | | | | |
| **Further investigations, sub- or sensitivity analyses** | **Index test** | | **Reference standard** | **Studies (Particip.)** | | **Sens. [%], 95% CI** | **Spec. [%], 95% CI** |
|  |  | |  |  | |  |  |
|  |  | |  |  | |  |  |
|  |  | |  |  | |  |  |
|  |  | |  |  | |  |  |
| **Conclusions of study authors** | *“Chest CT and ultrasound of the lungs are sensitive and moderately specific in diagnosing COVID-19.* *Thus, chest CT and ultrasound may have more utility for ruling out COVID-19 than for differentiation SARS-CoV-2 infection from other causes of respiratory illness. The uncertainty resulting from high or unclear risk of bias and the heterogeneity of included studies limit our ability to confidently draw conclusions based on our results.”* | | | | | | |

| **Jari (2022)**[217] – COVID-19 | | | | | | | |
| --- | --- | --- | --- | --- | --- | --- | --- |
| **Clinical context** | ***Patient population*** | Adults suspected of having COVID-19 | | | | | |
|  | ***Clinical setting*** | Emergency department | | | | | |
|  | ***Prevalence [%] (CI)*** | 50.9 (48.8; 53.1) | | | | | |
|  | ***Noteworthy incl. crit.*** | Adult patients (≥8 years), old), lung ultrasound was performed in the emergency department  equivalent | | | | | |
|  | ***Noteworthy excl. crit.*** | Studies limiting their analysis to the samples with only positive RT-PCR | | | | | |
| **Index test(s)** | Ultrasound of the lung (positive if: > 2 B lines per intercostal space, pleural line irregularity, or consolidations) | | | | | | |
| **Reference standard(s)** | RT-PCR test for SARS-CoV-2 | | | | | | |
| **DTA-SR characteristics** | ***No. of studies*** | 16 | | | | | |
|  | ***No. of participants*** | 2105 | | | | | |
|  | ***Year range*** | 2020 - 2021 | | | | | |
|  | ***Quality assessment*** | QUADAS-2 (some studies with high or unclear risk of bias) | | | | | |
|  | ***Special remark*** | Ten studies followed the 12-zone lung protocol, two studies used BLUE protocol, three studies did not scan the posterior lung areas, as in the 12-zone protocol, and two were scanned four lungs' areas in each hemithorax | | | | | |
| **Diagnostic accuracy** | ***Measure*** | Sensitivity [%] (95% CI):  86.9 (80.9; 91.3) | | | Specificity [%] (95% CI):  62.4 (49.4; 73.9) | | |
|  | ***GRADE*** | Low ⊕⊖⊕⊖ | | | Low ⊕⊖⊕⊖ | | |
| **Method. quality**  **(mod AMSTAR2)** | **Overall** | Moderate | | | | | |
| **Further investigations, sub- or sensitivity analyses** | **Index test** | | **Reference standard** | **Studies (Particip.)** | | **Sens. [%], 95% CI** | **Spec. [%], 95% CI** |
|  | Ultrasound of lungs with a four or six region protocol | | RT-PCR test for SARS-CoV-2 | 3 (214) | | 80 (64;90) | 48 (21; 76) |
|  |  | |  |  | |  |  |
|  |  | |  |  | |  |  |
|  |  | |  |  | |  |  |
| **Conclusions of study authors** | *“This is the first systematic review and meta-analysis done to evaluate the diagnostic performance of lung ultrasound for detecting COVID-19 pneumonia in the emergency setting. It was found that lung ultrasound had a moderate sensitivity and poor specificity. These results suggest that it should not be used independently to diagnose COVID-19, but could perform a triage process for patients with COVID-19 pneumonia (or other causes of pneumonia). Scanning a fewer number of lung regions can worsen the diagnostic accuracy.”* | | | | | | |

| **Matthies (2023)**[218] – COVID-19 | | | | | | | |
| --- | --- | --- | --- | --- | --- | --- | --- |
| **Clinical context** | ***Patient population*** | Adults | | | | | |
|  | ***Clinical setting*** | NA | | | | | |
|  | ***Prevalence [%] (CI)*** | 52.7 (51.2; 53.8) | | | | | |
|  | ***Noteworthy incl. crit.*** | Studies comparing lung ultrasound to RT-PCR, CT, or a final clinical diagnosis of COVID-19  COVID-19 | | | | | |
|  | ***Noteworthy excl. crit.*** | - | | | | | |
| **Index test(s)** | Lung ultrasound | | | | | | |
| **Reference standard(s)** | RT-PCR test for SARS-CoV-2, CT, final clinical diagnosis | | | | | | |
| **DTA-SR characteristics** | ***No. of studies*** | 20 | | | | | |
|  | ***No. of participants*** | 4314 | | | | | |
|  | ***Year range*** | 2020 – 2021 | | | | | |
|  | ***Quality assessment*** | QUADAS-2 (all studies with high risk of bias in patient selection) | | | | | |
|  | ***Special remark*** |  | | | | | |
| **Diagnostic accuracy** | ***Measure*** | Sensitivity [%] (95% CI):  87.2 (83.6; 90.2) | | | Specificity [%] (95% CI):  97.9 (95.8; 100) | | |
|  | ***GRADE*** | Moderate ⊕⊖⊕⊕ | | | Low ⊕⊖⊕⊖ | | |
| **Method. quality**  **(mod AMSTAR2)** | **Overall** | Moderate | | | | | |
| **Further investigations, sub- or sensitivity analyses** | **Index test** | | **Reference standard** | **Studies (Particip.)** | | **Sens. [%], 95% CI** | **Spec. [%], 95% CI** |
|  | Ultrasound of the lung (no further specification) | | RT-PCR test for SARS-CoV-2 | 15 (3913) | | 87 (83; 90) | 69 (60; 75) |
|  | Ultrasound of the lung (no further specification) | | CT | 5 (469) | | 90 (64; 98) | 71 (44; 88) |
|  | Ultrasound of the lung (no further specification) | | Final clinical diagnosis | 2 (160) | | 88 (82; 92) | 78 (71; 83) |
|  |  | |  |  | |  |  |
| **Conclusions of study authors** | *“During a period of high prevalence, lung ultrasound is a highly sensitive diagnostic test for COVID-19.”* | | | | | | |

| **Wang (2021)**[219] – COVID-19 | | | | | | | |
| --- | --- | --- | --- | --- | --- | --- | --- |
| **Clinical context** | ***Patient population*** | Patients with confirmed or suspected COVID-19 or asymptomatic SARS-CoV-2 infection | | | | | |
|  | ***Clinical setting*** | Emergency department (7), hospital (2) | | | | | |
|  | ***Prevalence [%] (CI)*** | 56.9 (52.7; 61.0) | | | | | |
|  | ***Noteworthy incl. crit.*** | Studies comparing lung ultrasound with CT in the diagnosis of COVID-19  interstitial pneumonia caused by COVID-199  COVID-19 | | | | | |
|  | ***Noteworthy excl. crit.*** | - | | | | | |
| **Index test(s)** | Ultrasound of the lung (no further specification) | | | | | | |
| **Reference standard(s)** | CT | | | | | | |
| **DTA-SR characteristics** | ***No. of studies*** | 9 | | | | | |
|  | ***No. of participants*** | 531 | | | | | |
|  | ***Year range*** | 2020 – 2021 | | | | | |
|  | ***Quality assessment*** | QUADAS-2 | | | | | |
|  | ***Special remark*** |  | | | | | |
| **Diagnostic accuracy** | ***Measure*** | Sensitivity [%] (95% CI):  96.0 (80.0; 99.0) | | | Specificity [%] (95% CI):  80.0 (60.0; 92.0)  (95.8; 100) | | |
|  | ***GRADE*** | Very low ⊖⊖⊕⊖ | | | Very low ⊖⊖⊕⊖ | | |
| **Method. quality**  **(mod AMSTAR2)** | **Overall** | Low | | | | | |
| **Further investigations, sub- or sensitivity analyses** | **Index test** | | **Reference standard** | **Studies (Particip.)** | | **Sens. [%], 95% CI** | **Spec. [%], 95% CI** |
|  |  | |  |  | |  |  |
|  |  | |  |  | |  |  |
|  |  | |  |  | |  |  |
|  |  | |  |  | |  |  |
| **Conclusions of study authors** | *“The level of diagnostic agreement between lung ultrasound and CT in the diagnosis of interstitial pneumonia caused by COVID-19 is high. Lung ultrasound can be therefore considered as an equally accurate alternative for CT in situations where molecular tests are not available.”* | | | | | | |

| **Abid (2024)**[220] – Pneumonia | | | | | | | |
| --- | --- | --- | --- | --- | --- | --- | --- |
| **Clinical context** | ***Patient population*** | Adults with clinically suspected pneumonia | | | | | |
|  | ***Clinical setting*** | Not documented | | | | | |
|  | ***Prevalence [%] (CI)*** | 37.3 (35.6; 39.1) | | | | | |
|  | ***Noteworthy incl. crit.*** | English original research, systematic reviews and meta-analysis published between 2017 and 2021, comparing X-rays to POCUS LUS | | | | | |
|  | ***Noteworthy excl. crit.*** | Caser reports, studies performed on COVID-19 | | | | | |
| **Index test(s)** | Lung ultrasound | | | | | | |
| **Reference standard(s)** | Not documented | | | | | | |
| **DTA-SR characteristics** | ***No. of studies*** | 12 | | | | | |
|  | ***No. of participants*** | 2897 | | | | | |
|  | ***Year range*** | 2017 – 2020 | | | | | |
|  | ***Quality assessment*** | QUADAS-2 (8 Studies with high risk of bias) | | | | | |
|  | ***Special remark*** |  | | | | | |
| **Diagnostic accuracy** | ***Measure*** | Sensitivity [%] (95% CI):  92.1 (84.1; 96.3) | | | Specificity [%] (95% CI):  81.9 (72.7; 88.5) | | |
|  | ***GRADE*** | Low ⊖⊕⊕⊖ | | | Very Low ⊖⊖⊕⊖ | | |
| **Method. quality**  **(mod AMSTAR2)** | **Overall** | Low | | | | | |
| **Further investigations, sub- or sensitivity analyses** | **Index test** | | **Reference standard** | **Studies (Particip.)** | | **Sens. [%], 95% CI** | **Spec. [%], 95% CI** |
|  |  | |  |  | |  |  |
|  |  | |  |  | |  |  |
|  |  | |  |  | |  |  |
|  |  | |  |  | |  |  |
| **Conclusions of study authors** | *“Our review supports that lung ultrasound can play a valuable role in detecting pneumonia early with high sensitivity, specificity, and diagnostic accuracy across diverse patient demographics, including paediatric, adult, and geriatric populations. Since it overcomes most of the limitations of chest X-ray and other diagnostic modalities, it can be utilized as a diagnostic tool for pneumonia for all age groups as it is a safe, readily available, and cost-effective.”* | | | | | | |

| **Alzahrani (2017)**[221] – Pneumonia | | | | | | | |
| --- | --- | --- | --- | --- | --- | --- | --- |
| **Clinical context** | ***Patient population*** | Adults and children | | | | | |
|  | ***Clinical setting*** | Community- and hospital acquired. 9 of 20 studies on emergency department | | | | | |
|  | ***Prevalence [%] (CI)*** | 63.9 (62.0; 65.8) | | | | | |
|  | ***Noteworthy incl. crit.*** | Lung ultrasound as index test, radiological imaging as reference standard, community- or hospital acquired pneumonia  ultrasound in the ED for the same indication | | | | | |
|  | ***Noteworthy excl. crit.*** | - | | | | | |
| **Index test(s)** | Lung ultrasound | | | | | | |
| **Reference standard(s)** | CT, chest X-ray, clinical diagnosis | | | | | | |
| **DTA-SR characteristics** | ***No. of studies*** | 20 (9 with high risk of bias) | | | | | |
|  | ***No. of participants*** | 2513 | | | | | |
|  | ***Year range*** | 1996 - 2015 | | | | | |
|  | ***Quality assessment*** | QUADAS-2 | | | | | |
|  | ***Special remark*** |  | | | | | |
| **Diagnostic accuracy** | ***Measure*** | Sensitivity [%] (95% CI):  85.0 (84.0; 87.0) | | | Specificity [%] (95% CI):  93.0 (92.0; 95.0) | | |
|  | ***GRADE*** | Moderate ⊕⊖⊕⊕ | | | Moderate ⊕⊖⊕⊕ | | |
| **Method. quality**  **(mod AMSTAR2)** | **Overall** | Moderate | | | | | |
| **Further investigations, sub- or sensitivity analyses** | **Index test** | | **Reference standard** | **Studies (Particip.)** | | **Sens. [%], 95% CI** | **Spec. [%], 95% CI** |
|  |  | |  |  | |  |  |
|  |  | |  |  | |  |  |
|  |  | |  |  | |  |  |
|  |  | |  |  | |  |  |
| **Conclusions of study authors** | *“Lung ultrasound can play a major and valuable role in the diagnosis of pneumonia with high diagnostic accuracy. Moreover, it can be an alternative to chest X-ray and thoracic CT in several conditions. Lung ultrasound can be used at the bedside easily, safely, and repetitively. Using lung ultrasound in emergency department, intensive care units, and medical wards after adequate training can be considered as a disruptive technology in this field.”* | | | | | | |

| **Chavez (2014)**[222] – Pneumonia | | | | | | | |
| --- | --- | --- | --- | --- | --- | --- | --- |
| **Clinical context** | ***Patient population*** | Adults with clinically suspected pneumonia or respiratory failure | | | | | |
|  | ***Clinical setting*** | 6 of 10 studies on emergency departments | | | | | |
|  | ***Prevalence [%] (CI)*** | 41.1 (39.7; 42.4) | | | | | |
|  | ***Noteworthy incl. crit.*** | Adults with suspected pneumonia or respiratory failure, chest x-ray or CT as reference standard | | | | | |
|  | ***Noteworthy excl. crit.*** | Studies that enrolled children. | | | | | |
| **Index test(s)** | Lung ultrasound | | | | | | |
| **Reference standard(s)** | CT, chest X-ray | | | | | | |
| **DTA-SR characteristics** | ***No. of studies*** | 10 | | | | | |
|  | ***No. of participants*** | 1172 | | | | | |
|  | ***Year range*** | 1996 - 2013 | | | | | |
|  | ***Quality assessment*** | QUADAS-2 (only studies with low risk of bias) | | | | | |
|  | ***Special remark*** |  | | | | | |
| **Diagnostic accuracy** | ***Measure*** | Sensitivity [%] (95% CI):  94.0 (92.0; 96.0) | | | Specificity [%] (95% CI):  96.0 (94.0; 97.0) | | |
|  | ***GRADE*** | Moderate ⊖⊕⊕⊕ | | | Low ⊖⊖⊕⊕ | | |
| **Method. quality**  **(mod AMSTAR2)** | **Overall** | Low | | | | | |
| **Further investigations, sub- or sensitivity analyses** | **Index test** | | **Reference standard** | **Studies (Particip.)** | | **Sens. [%], 95% CI** | **Spec. [%], 95% CI** |
|  |  | |  |  | |  |  |
|  |  | |  |  | |  |  |
|  |  | |  |  | |  |  |
|  |  | |  |  | |  |  |
| **Conclusions of study authors** | *“Our meta-analysis supports that lung ultrasound, when conducted by highly-skilled sonographers, performs well for the diagnosis of pneumonia. General practitioners and Emergency Medicine physicians should be encouraged to learn lung ultrasound since it appears to be an established diagnostic tool in the hands of experienced physicians.”* | | | | | | |

| **Desai (2024)**[223] – Pneumonia | | | | | | | |
| --- | --- | --- | --- | --- | --- | --- | --- |
| **Clinical context** | ***Patient population*** | Adults with either clinical suspicion or confirmed diagnosis of pneumonia or acute respiratory failure | | | | | |
|  | ***Clinical setting*** | 20 studies within emergency setting, 8 on intensive care unit, 1 on stroke unit | | | | | |
|  | ***Prevalence [%] (CI)*** | Not documented | | | | | |
|  | ***Noteworthy incl. crit.*** | Suspected/confirmed pneumonia or acute respiratory failure, reference method for diagnosing pneumonia was based on clinical data, laboratory results and confirmation by chest radiology/CT scan or both | | | | | |
|  | ***Noteworthy excl. crit.*** | Pediatric studies; <20 participants; diagnosis based on clinical data only | | | | | |
| **Index test(s)** | Lung ultrasound | | | | | | |
| **Reference standard(s)** | CT, X-ray, laboratory, clinical data | | | | | | |
| **DTA-SR characteristics** | ***No. of studies*** | 29 | | | | | |
|  | ***No. of participants*** | 6702 | | | | | |
|  | ***Year range*** | 1996 - 2017 | | | | | |
|  | ***Quality assessment*** | QUADAS-2 (10 studies with high risk of bias) | | | | | |
|  | ***Special remark*** |  | | | | | |
| **Diagnostic accuracy** | ***Measure*** | Sensitivity [%] (95% CI):  92.0 (91.0; 93.0) | | | Specificity [%] (95% CI):  94.0 (94.0; 95.0) | | |
|  | ***GRADE*** | Low ⊝⊝⊕⊕ | | | Low ⊝⊝⊕⊕ | | |
| **Method. quality**  **(mod AMSTAR2)** | **Overall** | Critically low | | | | | |
| **Further investigations, sub- or sensitivity analyses** | **Index test** | | **Reference standard** | **Studies (Particip.)** | | **Sens. [%], 95% CI** | **Spec. [%], 95% CI** |
|  |  | |  |  | |  |  |
|  |  | |  |  | |  |  |
|  |  | |  |  | |  |  |
|  |  | |  |  | |  |  |
| **Conclusions of study authors** | *“All aspects duly considered, ultrasound as a modality promises efficiency, efficacy and prudence in reaching an early diagnosis and can be safely employed for this purpose in patients suffering from pneumonia and a spectrum of cardiorespiratory conditions of varying etiologic and epidemiological factors.”* | | | | | | |

| **Gentilotti (2022)**[224] – Pneumonia | | | | | | | |
| --- | --- | --- | --- | --- | --- | --- | --- |
| **Clinical context** | ***Patient population*** | Adults and children with clinical suspicion of community acquired pneumonia | | | | | |
|  | ***Clinical setting*** | Emergency department | | | | | |
|  | ***Prevalence [%] (CI)*** | NA | | | | | |
|  | ***Noteworthy incl. crit.*** | Diagnostic test accuracy studies assessing pathogens that are commonly implicated in acute low respiratory tract infections CA-LRTI pathogenesis have been included | | | | | |
|  | ***Noteworthy excl. crit.*** | SARS-CoV-2 studies | | | | | |
| **Index test(s)** | Lung ultrasound | | | | | | |
| **Reference standard(s)** | CT, chest X-ray, expert consensus, diagnosis at discharge | | | | | | |
| **DTA-SR characteristics** | ***No. of studies*** | 33 | | | | | |
|  | ***No. of participants*** | 4901 | | | | | |
|  | ***Year range*** | 2008 - 2021 | | | | | |
|  | ***Quality assessment*** | QUADAS-2 (18 with high risk of bias) | | | | | |
|  | ***Special remark*** |  | | | | | |
| **Diagnostic accuracy** | ***Measure*** | Sensitivity [%] (95% CI):  91.9 (87.9; 94.7) | | | Specificity [%] (95% CI):  89.7 (81.3; 94.6) | | |
|  | ***GRADE*** | Low ⊖⊖⊕⊕ | | | Very low ⊖⊖⊕⊖ | | |
| **Method. quality**  **(mod AMSTAR2)** | **Overall** | Low | | | | | |
| **Further investigations, sub- or sensitivity analyses** | **Index test** | | **Reference standard** | **Studies (Particip.)** | | **Sens. [%], 95% CI** | **Spec. [%], 95% CI** |
|  |  | |  |  | |  |  |
|  |  | |  |  | |  |  |
|  |  | |  |  | |  |  |
|  |  | |  |  | |  |  |
| **Conclusions of study authors** | *“Lung ultrasound shows higher accuracy than chest X-ray for bacterial pneumonia at emergency department.”* | | | | | | |

| **Hu (2014)**[225] – Pneumonia | | | | | | | |
| --- | --- | --- | --- | --- | --- | --- | --- |
| **Clinical context** | ***Patient population*** | Adults with clinically suspected pneumonia | | | | | |
|  | ***Clinical setting*** | Not documented | | | | | |
|  | ***Prevalence [%] (CI)*** | 62.5 (59.6; 65.4) | | | | | |
|  | ***Noteworthy incl. crit.*** | Patients with suspected pneumonia, clearly defined reference standard, English | | | | | |
|  | ***Noteworthy excl. crit.*** | - | | | | | |
| **Index test(s)** | Lung ultrasound | | | | | | |
| **Reference standard(s)** | CT, chest X-ray, clinical presentation or microbiology | | | | | | |
| **DTA-SR characteristics** | ***No. of studies*** | 9 | | | | | |
|  | ***No. of participants*** | 1080 | | | | | |
|  | ***Year range*** | 2008 – 2013 | | | | | |
|  | ***Quality assessment*** | QUADAS (5 Studies with moderate risk of bias) | | | | | |
|  | ***Special remark*** |  | | | | | |
| **Diagnostic accuracy** | ***Measure*** | Sensitivity [%] (95% CI):  97.0 (93.0; 99.0) | | | Specificity [%] (95% CI):  94.0 (85.0; 98.0) | | |
|  | ***GRADE*** | Moderate ⊖⊕⊕⊕ | | | Low ⊖⊕⊕⊖ | | |
| **Method. quality**  **(mod AMSTAR2)** | **Overall** | Low | | | | | |
| **Further investigations, sub- or sensitivity analyses** | **Index test** | | **Reference standard** | **Studies (Particip.)** | | **Sens. [%], 95% CI** | **Spec. [%], 95% CI** |
|  |  | |  |  | |  |  |
|  |  | |  |  | |  |  |
|  |  | |  |  | |  |  |
|  |  | |  |  | |  |  |
| **Conclusions of study authors** | *“In summary, lung ultrasound plays a valuable role in the diagnosis of pneumonia, which is a promising attractive alternative to chest radiography and thoracic CT scan. The results should be interpreted in parallel with clinical findings and the results of conventional tests.”* | | | | | | |

| **Long (2017)**[226] – Pneumonia | | | | | | | |
| --- | --- | --- | --- | --- | --- | --- | --- |
| **Clinical context** | ***Patient population*** | Adults with clinically suspected pneumonia | | | | | |
|  | ***Clinical setting*** | NA | | | | | |
|  | ***Prevalence [%] (CI)*** | 56.8 (54.3; 59.3) | | | | | |
|  | ***Noteworthy incl. crit.*** | Adults with suspected pneumonia, chest X-ray or CT as reference standard | | | | | |
|  | ***Noteworthy excl. crit.*** | Studies that enrolled children. | | | | | |
| **Index test(s)** | Lung ultrasound | | | | | | |
| **Reference standard(s)** | CT, chest X-ray | | | | | | |
| **DTA-SR characteristics** | ***No. of studies*** | 12 | | | | | |
|  | ***No. of participants*** | 1515 | | | | | |
|  | ***Year range*** | 2009 - 2015 | | | | | |
|  | ***Quality assessment*** | QUADAS-2 (all studies with unclear risk of bias) | | | | | |
|  | ***Special remark*** | Six studies used chest X-ray as reference standard | | | | | |
| **Diagnostic accuracy** | ***Measure*** | Sensitivity [%] (95% CI):  88.0 (86.0; 90.0) | | | Specificity [%] (95% CI):  86.0 (83.0; 88.0) | | |
|  | ***GRADE*** | Low ⊖⊖⊕⊕ | | | Low ⊖⊖⊕⊕ | | |
| **Method. quality**  **(mod AMSTAR2)** | **Overall** | Low | | | | | |
| **Further investigations, sub- or sensitivity analyses** | **Index test** | | **Reference standard** | **Studies (Particip.)** | | **Sens. [%], 95% CI** | **Spec. [%], 95% CI** |
|  |  | |  |  | |  |  |
|  |  | |  |  | |  |  |
|  |  | |  |  | |  |  |
|  |  | |  |  | |  |  |
| **Conclusions of study authors** | *“Our meta-analysis indicates that lung ultrasound has high accuracy in the diagnosis of pneumonia. Moreover, lung ultrasound has bedside availability and feasibility. Therefore, lung ultrasound may be a promising alternative to chest X-ray and chest CT when it is not inconvenient.”* | | | | | | |

| **Orso (2018)**[227] – Pneumonia | | | | | | | |
| --- | --- | --- | --- | --- | --- | --- | --- |
| **Clinical context** | ***Patient population*** | Adults with suspected community acquired pneumonia | | | | | |
|  | ***Clinical setting*** | Emergency department | | | | | |
|  | ***Prevalence [%] (CI)*** | 41.1 (39.7; 42.4) | | | | | |
|  | ***Noteworthy incl. crit.*** | Adults with suspected pneumonia, chest x-ray or CT as reference standard and on emergency departments. | | | | | |
|  | ***Noteworthy excl. crit.*** | Studies that enrolled children. | | | | | |
| **Index test(s)** | Lung ultrasound (consultation and/or focal B lines) | | | | | | |
| **Reference standard(s)** | CT, chest X-ray | | | | | | |
| **DTA-SR characteristics** | ***No. of studies*** | 17 | | | | | |
|  | ***No. of participants*** | 5108 | | | | | |
|  | ***Year range*** | 2008 - 2017 | | | | | |
|  | ***Quality assessment*** | QUADAS-2 (only one study with high risk of bias) | | | | | |
|  | ***Special remark*** |  | | | | | |
| **Diagnostic accuracy** | ***Measure*** | Sensitivity [%] (95% CI):  92.0 (86.0; 95.0) | | | Specificity [%] (95% CI):  93.0 (86.0; 97.0) | | |
|  | ***GRADE*** | Moderate ⊕⊖⊕⊕ | | | Low ⊕⊖⊕⊖ | | |
| **Method. quality**  **(mod AMSTAR2)** | **Overall** | Moderate | | | | | |
| **Further investigations, sub- or sensitivity analyses** | **Index test** | | **Reference standard** | **Studies (Particip.)** | | **Sens. [%], 95% CI** | **Spec. [%], 95% CI** |
|  |  | |  |  | |  |  |
|  |  | |  |  | |  |  |
|  |  | |  |  | |  |  |
|  |  | |  |  | |  |  |
| **Conclusions of study authors** | “*The lung ultrasound proved to be a sufficiently useful and accurate tool to diagnose community acquired pneumonia in an adult population in the emergency department. Some concerns have been raised on the robustness of the obtained results, because of the lack of a well standardized methodology in the studies included (in particular on the choice of reference standards, the experience of the sonographer, and the ultrasound patterns considered significant for community acquired pneumonia). More methodologically rigorous studies are needed.”* | | | | | | |

| **Padrao (2025)**[228] – Pneumonia | | | | | | | |
| --- | --- | --- | --- | --- | --- | --- | --- |
| **Clinical context** | ***Patient population*** | Adults | | | | | |
|  | ***Clinical setting*** | Emergency departments or hospital wards | | | | | |
|  | ***Prevalence [%] (CI)*** | Not documented | | | | | |
|  | ***Noteworthy incl. crit.*** | - | | | | | |
|  | ***Noteworthy excl. crit.*** | Case series / reports, studies with overlapping cohorts (including only the bigger cohort | | | | | |
| **Index test(s)** | Lung ultrasound (focal B-Lines) | | | | | | |
| **Reference standard(s)** | CT, chest X-ray | | | | | | |
| **DTA-SR characteristics** | ***No. of studies*** | 8 | | | | | |
|  | ***No. of participants*** | 1390 | | | | | |
|  | ***Year range*** | NA | | | | | |
|  | ***Quality assessment*** | QUADAS-2 (6 studies with high risk of bias) | | | | | |
|  | ***Special remark*** | Subgroup with intensive care patients, ventilator associated pneumonia, stratified by reference standard | | | | | |
| **Diagnostic accuracy** | ***Measure*** | Sensitivity [%] (95% CI):  37.0 (19.0; 59.0) | | | Specificity [%] (95% CI):  95.0 (73.0; 99.0) | | |
|  | ***GRADE*** | Low ⊕⊖⊕⊖ | | | Low ⊕⊖⊕⊖ | | |
| **Method. quality**  **(mod AMSTAR2)** | **Overall** | Moderate | | | | | |
| **Further investigations, sub- or sensitivity analyses** | **Index test** | | **Reference standard** | **Studies (Particip.)** | | **Sens. [%], 95% CI** | **Spec. [%], 95% CI** |
|  | Lung ultrasound (consolidation) | | CT, chest X-rax | 7 (1238) | | 81 (71; 88) | 96 (82; 99) |
|  | Lung ultrasound (subpleural consolidation) | | CT, chest X-rax | 3 (336) | | 60 (32; 83) | 94 (38; 100) |
|  | Lung ultrasound (dynamic air bronchograms) | | CT, chest X-rax | 3 (198) | | 53 (15; 87) | 100 (0; 100) |
|  | Lung ultrasound (BLUE protocol) | | CT, chest X-rax | 2 (537) | | 85 (78; 90) | 92 (59; 99) |
| **Conclusions of study authors** | *“Lung ultrasound had good performance in diagnosing community aquired pneumonia or hospital aquired pneumonia. Dynamic air bronchograms and the BLUE protocol had the highest positive likelihood ratio for non-ventilator associated pneumonia patients.”* | | | | | | |

| **Sistani (2021)**[229] – Pneumonia | | | | | | | |
| --- | --- | --- | --- | --- | --- | --- | --- |
| **Clinical context** | ***Patient population*** | Adults with clinically suspected pneumonia | | | | | |
|  | ***Clinical setting*** | Emergency department | | | | | |
|  | ***Prevalence [%] (CI)*** | 52.4 (50.1; 54.6) | | | | | |
|  | ***Noteworthy incl. crit.*** | Adults with suspected pneumonia, chest x-ray, CT, clinical presentation or microbiology as reference standard | | | | | |
|  | ***Noteworthy excl. crit.*** | Studies that enrolled children. | | | | | |
| **Index test(s)** | Lung ultrasound | | | | | | |
| **Reference standard(s)** | CT, chest X-ray, clinical presentation or microbiology | | | | | | |
| **DTA-SR characteristics** | ***No. of studies*** | 12 | | | | | |
|  | ***No. of participants*** | NA | | | | | |
|  | ***Year range*** | 2011 – 2020 | | | | | |
|  | ***Quality assessment*** | QUADAS-2 (4 with high risk of bias) | | | | | |
|  | ***Special remark*** |  | | | | | |
| **Diagnostic accuracy** | ***Measure*** | Sensitivity [%] (95% CI):  86.0 (84.0; 87.0) | | | Specificity [%] (95% CI):  81.0 (79.0; 83.0) | | |
|  | ***GRADE*** | Low ⊖⊖⊕⊕ | | | Low ⊖⊖⊕⊕ | | |
| **Method. quality**  **(mod AMSTAR2)** | **Overall** | Critically low | | | | | |
| **Further investigations, sub- or sensitivity analyses** | **Index test** | | **Reference standard** | **Studies (Particip.)** | | **Sens. [%], 95% CI** | **Spec. [%], 95% CI** |
|  | Lung ultrasound on all wards | | CT, chest X-ray, clinic | 16 (2040) | | 86 (84;87) | 81 (79; 83) |
|  |  | |  |  | |  |  |
|  |  | |  |  | |  |  |
|  |  | |  |  | |  |  |
| **Conclusions of study authors** | *“This study demonstrated that lung ultrasound is a useful diagnostic technique for the diagnosis of pneumonia. This method has many advantages, which can be used by emergency department physicians with high accuracy, sensitivity, and specificity. The solution enables a fast evaluation of unstable patients, without using ionizing radiation. Among the elderly population, this diagnostic method has the potential to produce better results than a chest X-ray for pneumonia. The quick performance of a lung ultrasound may facilitate the rapid, cost effective, and safe diagnosis of pneumonia which can be a fatal disease. Hence, the use of lung ultrasound has the opportunity to expedite a patient’s treatment and prognosis for those affected with pneumonia.”* | | | | | | |

| **Vera-Ponce (2025)**[230] – Pneumonia | | | | | | | |
| --- | --- | --- | --- | --- | --- | --- | --- |
| **Clinical context** | ***Patient population*** | Adults | | | | | |
|  | ***Clinical setting*** | Emergency department, intensive care unit | | | | | |
|  | ***Prevalence [%] (CI)*** | Not documented | | | | | |
|  | ***Noteworthy incl. crit.*** | Studies comparing lung ultrasound to chest X-ray | | | | | |
|  | ***Noteworthy excl. crit.*** | Case reports | | | | | |
| **Index test(s)** | Lung ultrasound | | | | | | |
| **Reference standard(s)** | CT | | | | | | |
| **DTA-SR characteristics** | ***No. of studies*** | 8 | | | | | |
|  | ***No. of participants*** | 1011 | | | | | |
|  | ***Year range*** | 2013 - 2020 | | | | | |
|  | ***Quality assessment*** | QUADAS-2 (generally low risk of bias) | | | | | |
|  | ***Special remark*** |  | | | | | |
| **Diagnostic accuracy** | ***Measure*** | Sensitivity [%] (95% CI):  90.0 (81.0; 96.0) | | | Specificity [%] (95% CI):  91.0 (80.0; 98.0) | | |
|  | ***GRADE*** | Low ⊕⊖⊕⊖ | | | Low ⊕⊖⊕⊖ | | |
| **Method. quality**  **(mod AMSTAR2)** | **Overall** | Moderate | | | | | |
| **Further investigations, sub- or sensitivity analyses** | **Index test** | | **Reference standard** | **Studies (Particip.)** | | **Sens. [%], 95% CI** | **Spec. [%], 95% CI** |
|  |  | |  |  | |  |  |
|  |  | |  |  | |  |  |
|  |  | |  |  | |  |  |
|  |  | |  |  | |  |  |
| **Conclusions of study authors** | *“The findings of this systematic reviedw and meta-analysis provide strong evidence that pulmonary ultrasound offers superior diagnostic accuracy compared to chest radiography for detecting pneumonia. Beyond the traditional metrics of sensitivity and specificity, the substantial difference in likelihood ratio demonstrates ultrasound’s superior clinical utility in both confirming and excluding pneumonia. This diagnostic advantage, combined with ultrasound’s non-invasive nature, absence of ionizing radiation, and bedside availability, positions it as a valuable first-line diagnostic tool across various clinical settings.”* | | | | | | |

| **Xia (2016)**[231] – Pneumonia | | | | | | | |
| --- | --- | --- | --- | --- | --- | --- | --- |
| **Clinical context** | ***Patient population*** | Adults with clinically suspected pneumonia | | | | | |
|  | ***Clinical setting*** | 11 of 14 studies on emergency department or medical ward | | | | | |
|  | ***Prevalence [%] (CI)*** | 52.4 (50.1; 54.6) | | | | | |
|  | ***Noteworthy incl. crit.*** | Adults with suspected pneumonia, chest x-ray, CT, clinical presentation or microbiology as reference standard | | | | | |
|  | ***Noteworthy excl. crit.*** | - | | | | | |
| **Index test(s)** | Lung ultrasound | | | | | | |
| **Reference standard(s)** | CT, chest X-ray, clinical presentation or microbiology | | | | | | |
| **DTA-SR characteristics** | ***No. of studies*** | 14 | | | | | |
|  | ***No. of participants*** | 1911 | | | | | |
|  | ***Year range*** | 1996 – 2015 | | | | | |
|  | ***Quality assessment*** | QUADAS-2 (only one study with unclear risk of bias) | | | | | |
|  | ***Special remark*** |  | | | | | |
| **Diagnostic accuracy** | ***Measure*** | Sensitivity [%] (95% CI):  90.4 (88.4; 92.1) | | | Specificity [%] (95% CI):  88.4 (86.1; 90.4) | | |
|  | ***GRADE*** | Low ⊖⊖⊕⊕ | | | Low ⊖⊖⊕⊕ | | |
| **Method. quality**  **(mod AMSTAR2)** | **Overall** | Low | | | | | |
| **Further investigations, sub- or sensitivity analyses** | **Index test** | | **Reference standard** | **Studies (Particip.)** | | **Sens. [%], 95% CI** | **Spec. [%], 95% CI** |
|  |  | |  |  | |  |  |
|  |  | |  |  | |  |  |
|  |  | |  |  | |  |  |
|  |  | |  |  | |  |  |
| **Conclusions of study authors** | *“In conclusion, based on current available evidence, lung ultrasound is a valid alternative for the diagnosing pneumonia in adults. This is just a start, a beginning of a new trend and a convenient bedside tool for diagnosis of a common health problem.”* | | | | | | |

###### Respiratory other

| **Seyala (2026)**[232] – Acute dyspnoe (no specific condition) | | | | | | | |
| --- | --- | --- | --- | --- | --- | --- | --- |
| **Clinical context** | ***Patient population*** | Adults | | | | | |
|  | ***Clinical setting*** | Emergency department | | | | | |
|  | ***Prevalence [%] (CI)*** | Not reported | | | | | |
|  | ***Noteworthy incl. crit.*** | Any study evaluating POCUS for dyspnea in the ED, regardless of identified disease and including various outcomes | | | | | |
|  | ***Noteworthy excl. crit.*** | - | | | | | |
| **Index test(s)** | POCUS | | | | | | |
| **Reference standard(s)** | Varying: Chest radiography, CT, clinical examination, or biomarkers such as brain natriuretic peptide (BNP)], precluding a unified quantitative synthesis | | | | | | |
| **DTA-SR characteristics** | ***No. of studies*** | 19 | | | | | |
|  | ***No. of participants*** | 9275 | | | | | |
|  | ***Year range*** | 2011 - 2025 | | | | | |
|  | ***Quality assessment*** | QUADAS-2 | | | | | |
|  | ***Special remark*** |  | | | | | |
| **Diagnostic accuracy** | ***Measure*** | Sensitivity [%] (95% CI):  85.6 (84.0; 87.2) | | | Specificity [%] (95% CI):  80.8 (79.0; 82.5) | | |
|  | ***GRADE*** | Low ⊖⊖⊕⊕ | | | Low ⊖⊖⊕⊕ | | |
| **Method. quality**  **(mod AMSTAR2)** | **Overall** | Low | | | | | |
| **Further investigations, sub- or sensitivity analyses** | **Index test** | | **Reference standard** | **Studies (Particip.)** | | **Sens. [%], 95% CI** | **Spec. [%], 95% CI** |
|  |  | |  |  | |  |  |
|  |  | |  |  | |  |  |
|  |  | |  |  | |  |  |
|  |  | |  |  | |  |  |
| **Conclusions of study authors** | *“This systematic review and meta-analysis offer a thorough assessment of the diagnostic accuracy of POCUS across a range of emergency deparment contexts. A key observation from the literature is the variability in how POCUS was assessed in relation to other diagnostic methods, including chest X-ray, chest CT, and physical examination.”* | | | | | | |

| **Jianguo (2026)**[233] – Acute respiratory failure | | | | | | | |
| --- | --- | --- | --- | --- | --- | --- | --- |
| **Clinical context** | ***Patient population*** | Adults | | | | | |
|  | ***Clinical setting*** | Mostly intensive care unit, some emergency department | | | | | |
|  | ***Prevalence [%] (CI)*** | Not documented | | | | | |
|  | ***Noteworthy incl. crit.*** | Studies were eligible if the reference standard was applied to all or a clearly defined subset of participants, and if the timing relative to the index test was sufficiently close to minimize misclassification due to rapid clinical change. Clearly described scanning protocol (e.g., BLUE protocol, 6–12 zone approaches, or equivalent) and predefined sonographic criteria for the target condition. | | | | | |
|  | ***Noteworthy excl. crit.*** | Case reports, case series with < 10 patients, narrative  reviews, editorials, conference abstracts without full data, pediatric-only studies, and studies in which lung ultrasound was used solely for monitoring or prognostication without a diagnostic accuracy comparison against a reference standard | | | | | |
| **Index test(s)** | Lung ultrasound (BLUE protocol, 6–12 zone approaches, or equivalent) | | | | | | |
| **Reference standard(s)** | CT, chest X-ray, echocardiography for cardiogenic pulmonary edema, composite reference (clinical course, microbiology, and imaging) | | | | | | |
| **DTA-SR characteristics** | ***No. of studies*** | 20 | | | | | |
|  | ***No. of participants*** | 3083 | | | | | |
|  | ***Year range*** | 2004 - 2024 | | | | | |
|  | ***Quality assessment*** | QUADAS-2 | | | | | |
|  | ***Special remark*** |  | | | | | |
| **Diagnostic accuracy** | ***Measure*** | Sensitivity [%] (95% CI):  89.0 (83.0; 94.0) | | | Specificity [%] (95% CI):  94.0 (90.0; 97.0) | | |
|  | ***GRADE*** | Low ⊕⊖⊕⊖ | | | Moderate ⊕⊖⊕⊕ | | |
| **Method. quality (mod AMSTAR2)** | **Overall** | Moderate | | | | | |
| **Further investigations, sub- or sensitivity analyses** | **Index test** | | **Reference standard** | **Studies (Particip.)** | | **Sens. [%], 95% CI** | **Spec. [%], 95% CI** |
|  | Blue protocol-type scanning approach | | CT, chest X-ray, composite reference | 8 (907) | | 84 (69; 93) | 96 (87; 99) |
|  | Multi-zone scanning schemes | | CT, chest X-ray, composite reference | 12 (2176) | | 92 (85; 96) | 94 (96; 98) |
|  | Scans were performed by expert operators | | CT, chest X-ray, composite reference | 13 (1760) | | 91 (82; 96) | 95 (92; 97) |
|  | Scans by trainees / fellows or recently trained operators | | CT, chest X-ray, composite reference | 7 (1323) | | 87 (75; 93) | 91 (76; 97) |
| **Conclusions of study authors** | *“Although substantial heterogeneity and some methodological limitations of the primary literature require cautious interpretation, the overall body of evidence strongly supports the use of lung ultrasound as a first-line bedside imaging modality in this population.”* | | | | | | |

| **Yuan (2021)**[234] – Acute respiratory failure | | | | | | | |
| --- | --- | --- | --- | --- | --- | --- | --- |
| **Clinical context** | ***Patient population*** | Adults with clinically suspected or confirmed acute respiratory failure  by any etiology | | | | | |
|  | ***Clinical setting*** | Intensive care unit | | | | | |
|  | ***Prevalence [%] (CI)*** | Not documented | | | | | |
|  | ***Noteworthy incl. crit.*** | Suspected or confirmed acute respiratory failure, comparing lung ultrasound with X-ray or CT | | | | | |
|  | ***Noteworthy excl. crit.*** | Pediatric studies | | | | | |
| **Index test(s)** | Lung ultrasound | | | | | | |
| **Reference standard(s)** | CT, chest X-ray | | | | | | |
| **DTA-SR characteristics** | ***No. of studies*** | 11 | | | | | |
|  | ***No. of participants*** | 1232 | | | | | |
|  | ***Year range*** | 2004 -2019 | | | | | |
|  | ***Quality assessment*** | QUADAS-2 | | | | | |
|  | ***Special remark*** | - | | | | | |
| **Diagnostic accuracy** | ***Measure*** | Sensitivity [%] (95% CI):  92.0 (85.0; 96.0) | | | Specificity [%] (95% CI):  98.0 (94.0; 99.0) | | |
|  | ***GRADE*** | Very low ⊕⊖⊖⊖ | | | Low ⊕⊖⊖⊕ | | |
| **Method. quality**  **(mod AMSTAR2)** | **Overall** | Moderate | | | | | |
| **Further investigations, sub- or sensitivity analyses** | **Index test** | | **Reference standard** | **Studies (Particip.)** | | **Sens. [%], 95% CI** | **Spec. [%], 95% CI** |
|  |  | |  |  | |  |  |
|  |  | |  |  | |  |  |
|  |  | |  |  | |  |  |
|  |  | |  |  | |  |  |
| **Conclusions of study authors** | *“This systematic review and meta-analysis demonstrated that lung ultrasound had moderate sensitivity and high specificity for diagnosing acute respiratory failure in critically ill patients when compared with X-ray or CT. Lung ultrasound seems to be a well-validated modality to investigate the cause of acute respiratory failure or thoracic pathology associated with the diagnosis.”* | | | | | | |

| **Boumans (2024)**[235] – Acute respiratory distress syndrome | | | | | | | |
| --- | --- | --- | --- | --- | --- | --- | --- |
| **Clinical context** | ***Patient population*** | Adults with acute respiratory failure or requiring mechanical ventilation | | | | | |
|  | ***Clinical setting*** | Mainly intensive care units (medical, surgical, or mixed), with additional studies in the emergency department and one on a medical ward | | | | | |
|  | ***Prevalence [%] (CI)*** | 28.8 (26.9; 30.8) | | | | | |
|  | ***Noteworthy incl. crit.*** | Adult patients and studies assessing the diagnostic accuracy of lung ultrasound compared to the Berlin definition | | | | | |
|  | ***Noteworthy excl. crit.*** | Pediatric studies, case reports/series, non-English publications, and studies without an appropriate reference standard | | | | | |
| **Index test(s)** | Lung ultrasound with signs of lung sliding, comet-tail artifact, lung point par emergency physician | | | | | | |
| **Reference standard(s)** | Berlin definition using CT or chest X-ray | | | | | | |
| **DTA-SR characteristics** | ***No. of studies*** | 11 | | | | | |
|  | ***No. of participants*** | 2075 | | | | | |
|  | ***Year range*** | 2014 - 2023 | | | | | |
|  | ***Quality assessment*** | QUADAS-2 (1 high risk of bias) | | | | | |
|  | ***Special remark*** |  | | | | | |
| **Diagnostic accuracy** | ***Measure*** | Sensitivity [%] (95% CI):  63.1 (45.0; 78.2) | | | Specificity [%] (95% CI):  94.2 (85.6; 97.8) | | |
|  | ***GRADE*** | Very low ⊕⊝⊝⊝ | | | Low ⊕⊝⊝⊕ | | |
| **Method. quality**  **(mod AMSTAR2)** | **Overall** | Moderate | | | | | |
| **Further investigations, sub- or sensitivity analyses** | **Index test** | | **Reference standard** | **Studies (Particip.)** | | **Sens. [%], 95% CI** | **Spec. [%], 95% CI** |
|  |  | |  |  | |  |  |
|  |  | |  |  | |  |  |
|  |  | |  |  | |  |  |
|  |  | |  |  | |  |  |
| **Conclusions of study authors** | *“This review confirms the hypothesis that lung ultrasound is a reliable method for diagnosing acute respiratory distress syndrome in adult patients.”* | | | | | | |

| **Ji (2026)**[236] – Acute respiratory distress syndrome | | | | | | | |
| --- | --- | --- | --- | --- | --- | --- | --- |
| **Clinical context** | ***Patient population*** | Adults | | | | | |
|  | ***Clinical setting*** | Emergency department, general ward, intensive care unit | | | | | |
|  | ***Prevalence [%] (CI)*** | 28.0 (NA) | | | | | |
|  | ***Noteworthy incl. crit.*** | Studies using CT alone or alternative consensus definitions (provided they match any standard and validated criteria) were included. | | | | | |
|  | ***Noteworthy excl. crit.*** | Case reports, small case series (<10 patients), conference abstracts, studies without a clear reference standard or without blinded interpretation of LUS and reference data were excluded | | | | | |
| **Index test(s)** | Lund ultrasound | | | | | | |
| **Reference standard(s)** | Berlin definition using CT or chest X-ray | | | | | | |
| **DTA-SR characteristics** | ***No. of studies*** | 14 | | | | | |
|  | ***No. of participants*** | 1885 | | | | | |
|  | ***Year range*** | 2008 - 2023 | | | | | |
|  | ***Quality assessment*** | QUADAS-2 | | | | | |
|  | ***Special remark*** | Heterogeneity not assessed specifically for sensibility or specificity | | | | | |
| **Diagnostic accuracy** | ***Measure*** | Sensitivity [%] (95% CI):  84.0 (69.0; 92.0) | | | Specificity [%] (95% CI):  94.0 (83.0; 98.0) | | |
|  | ***GRADE*** | Low ⊕⊖⊕⊖ | | | Low ⊕⊖⊕⊖ | | |
| **Method. quality (mod AMSTAR2)** | **Overall** | Moderate | | | | | |
| **Further investigations, sub- or sensitivity analyses** | **Index test** | | **Reference standard** | **Studies (Particip.)** | | **Sens. [%], 95% CI** | **Spec. [%], 95% CI** |
|  | Only pattern-based LUS protocol | | Berlin definition using CT or chest X-ray | 8 (1223) | | 82 (53; 95) | 96 (83; 99) |
|  | Only score-based LUS protocol | | Berlin definition using CT or chest X-ray | 5 (341) | | 90 (79; 96) | 83 (50; 96) |
|  |  | |  |  | |  |  |
|  |  | |  |  | |  |  |
| **Conclusions of study authors** | *“Lung ultrasound demonstrates excellent rule‑in and rule‑out performance for ARDS in critically ill adults, rivalling CT accuracy without its drawbacks. Adoption of standardized LUS protocols and integration into acute respiratory distress syndrome diagnostic pathways could enhance early detection, optimize management, and reduce reliance on ionizing imaging.”* | | | | | | |

| **Sreedharan (2026)**[237] – Acute respiratory distress syndrome | | | | | | | |
| --- | --- | --- | --- | --- | --- | --- | --- |
| **Clinical context** | ***Patient population*** | Suspected ARDS, not further specified | | | | | |
|  | ***Clinical setting*** | Intensive care unit und emergency department | | | | | |
|  | ***Prevalence [%] (CI)*** | Not documented | | | | | |
|  | ***Noteworthy incl. crit.*** | Studies using established ARDS criteria as reference standard | | | | | |
|  | ***Noteworthy excl. crit.*** | - | | | | | |
| **Index test(s)** | Lung ultrasound (various protocols, 4-12 regions) | | | | | | |
| **Reference standard(s)** | CT Imaging and Berlin Criteria for ARDS | | | | | | |
| **DTA-SR characteristics** | ***No. of studies*** | 16 | | | | | |
|  | ***No. of participants*** | 1938 | | | | | |
|  | ***Year range*** | 2014 - 2023 | | | | | |
|  | ***Quality assessment*** | QUADAS-2 | | | | | |
|  | ***Special remark*** |  | | | | | |
| **Diagnostic accuracy** | ***Measure*** | Sensitivity [%] (95% CI):  75.0 (62.0; 85.0) | | | Specificity [%] (95% CI):  87.0 (80.0; 91.0) | | |
|  | ***GRADE*** | Low ⊕⊖⊕⊖ | | | Low ⊕⊖⊕⊖ | | |
| **Method. quality**  **(mod AMSTAR2)** | **Overall** | Moderate | | | | | |
| **Further investigations, sub- or sensitivity analyses** | **Index test** | | **Reference standard** | **Studies (Particip.)** | | **Sens. [%], 95% CI** | **Spec. [%], 95% CI** |
|  | Lung ultrasound (common-effect model) | | CT Imaging and Berlin Criteria for ARDS | 15 | | 51 (49; 53) | 83 (82; 84) |
|  | Lung ultrasound (adequate blinding) | | CT Imaging and Berlin Criteria for ARDS | NA | | 70 (55; 82) | 83 (74; 90) |
|  |  | |  |  | |  |  |
|  |  | |  |  | |  |  |
|  |  | |  |  | |  |  |
| **Conclusions of study authors** | *“This meta-analysis of 16 studies encompassing 5888 patients demonstrated that lung ultrasound has good diagnostic accuracy for acute respiratory distress syndrome, with a pooled diagnostic odds ratio of 14.98, sensitivity of 0.75, specificity of 0.87, and an Area Under the Receiver Operating Characteristic Curve of 0.91. Diagnostic performance was significantly higher with comprehensive scanning protocols utilizing 8 or more zones, in intensive care unit settings, and for severe acute respiratory distress syndrome. The moderate sensitivity of 0.75 indicates that negative LUS findings occurred in approximately 25% of confirmed acute respiratory distress syndrome cases, suggesting that lung ultrasound functions more effectively as a “rule-in” rather than a “rule-out” diagnostic tool.”* | | | | | | |

# Abbreviations

AAA = abdominal aortic aneurysm

AAD = acute aortic dissection

ACR = American College of Rheumatology

AD = aortic dissection

ADHF = acute decompensated heart failure

ASA = American Society of Anesthesiologists Physical Status Classification

BLUE = Bed Lung Ultrasound in Emergency protocol

CEUS = Contrast enhanced ultrasound

China CDC = Chinese Center for Disease Control and Prevention

CI = confidence interval

COPD = Chronic Obstructive Pulmonary Disease

CPPD = Calcium Pyrophosphate Dehydrate Deposition Disease

CT = computed tomography

CTA = computed tomography angiography

CXR = chest x-ray

DCS = double contour sign

DECT = dual energy computer tomography

DSA = digital substraction angiography

DTA = diagnostic test accuracy systematic review

ED = emergency department

ESC = European Society of Cardiology

FUP = follow-up

IA = intra-cranial angiography

IAA = intra-arterial angiography

IVC = inferior vena cava

LVEF = left ventricular ejection fraction

LUS = lung ultrasound

MRA = magnetic resonance angiography

MRI = magnetic resonance imaging

NA = not documented

ONSD = optic nerve sheath diameter

PEA = pulseless electrical activity

PET = Positron Emission Tomography

PHV = Prosthetic heart valve

POCUS = point of care ultrasound

QUADAS = Quality Assessment of Diagnostic Accuracy Studies

RADUS = radiologist performed ultrasound

rAAA = ruptured abdominal aortic aneurysm

RCT = randomized controlled trials

ROB = Risk of Bias

ROBINS-I = Risk Of Bias In Non-Randomized Studies – Interventions

ROSC = return of spontaneous circulation

RT-PCR = real-time polymerase chain reaction

SPECT = Single Photon Emission Computed Tomography

STARD = Standards for Reporting of Diagnostic Accuracy Studies

TAU = transabdominal ultrasound

TCCD = transcranial color Doppler

TCD = transcranial Doppler

TCU = transcranial ultrasound

TEE = transesophageal echocardiography

TIA = transient ischemic attack

TTE = transthoracic echocardiography

TVU = transvaginal ultrasound
V/Q scan = ventilation/perfusion scan

WHO = World Health Organization
