# Supplementary material for: Diagnostic accuracy of point-of-care ultrasound in adult non-traumatic emergency medicine: an umbrella review
Source: BMC Med. 2026 Jul 25;24:579. doi: 10.1186/s12916-026-05094-2 (PMC13689006; doi:10.1186/s12916-026-05094-2)
Supplement: Supplementary file 6 [file 12916_2026_5094_MOESM6_ESM.docx]

## Table S6. Excluded reviews during assessment of emergency medicine POCUS condition

Abdelnaby R, Mohamed KA, Elgenidy A, Sonbol YT, Bedewy MM, Aboutaleb AM, et al. Muscle Sonography in Inclusion Body Myositis: A Systematic Review and Meta-Analysis of 944 Measurements. Cells. 2022;11(4):600. doi:10.3390/cells11040600

Abdulla J, Sivertsen J, Kofoed KF, Alkadhi H, LaBounty T, Abildstrom SZ, et al. Evaluation of aortic valve stenosis by cardiac multislice computed tomography compared with echocardiography: a systematic review and meta-analysis. J Heart Valve Dis. 2009;18(6):634–43.

Abraha I, Luchetta ML, De Florio R, Cozzolino F, Casazza G, Duca P, et al. Ultrasonography for endoleak detection after endoluminal abdominal aortic aneurysm repair. Cochrane Vascular Group, editor. Cochrane Database of Systematic Reviews. 2017;2017(6). doi:10.1002/14651858.CD010296.pub2

Adebajo CO, Talwalkar JA, Poterucha JJ, Kim WR, Charlton MR. Ultrasound-Based Transient Elastography for the Detection of Hepatic Fibrosis in Patients With Recurrent HCV After Liver Transplantation: Systematic Review and Meta-Analysis. Gastroenterology. 2011;140(5):S-960. doi:10.1016/S0016-5085(11)63976-0

Afandy AN, Afandy NO. Diagnostic accuracy of contrast-enhanced ultrasound in differentiating benign and malignant hepatic lesion: A meta-analysis. Journal of Medical Imaging and Radiation Sciences. 2023;54(2):S6–7. doi:10.1016/j.jmir.2023.05.033

Aimo A, Kollia E, Ntritsos G, Barison A, Masci PG, Figliozzi S, et al. Echocardiography versus computed tomography and cardiac magnetic resonance for the detection of left heart thrombosis: a systematic review and meta-analysis. Clin Res Cardiol. 2021;110(11):1697–703. doi:10.1007/s00392-020-01741-7

Al Shuhaimy A, Ababtain K, Sun Z. Diagnostic value of non-invasive imaging techniques in the detection of carotid artery stenosis: a systematic review. Radiographer. 2009;56(3):14–8. doi:10.1002/j.2051-3909.2009.tb00104.x

Albuck A, Attia A, Aboueisha M, Omar M, Munshi R, Cambell B, et al. THE DIAGNOSTIC PERFORMANCE OF ULTRASOUND AND COMPUTED TOMOGRAPHY IN DETECTING NECK LYMPH NODE METASTASIS: A SYSTEMATIC REVIEW AND META-ANALYSIS. Thyroid. 2022;32:31A-31A.

Albuck AL, Issa PP, Hussein M, Aboueisha M, Attia AS, Omar M, et al. A combination of computed tomography scan and ultrasound provides optimal detection of cervical lymph node metastasis in papillary thyroid carcinomas: A systematic review and meta‐analysis. Head & Neck. 2023;45(9):2173–84. doi:10.1002/hed.27451

Alcázar JL, Carriles I, Cajas MB, Costa S, Fabra S, Cabrero M, et al. Diagnostic Performance of Two-Dimensional Ultrasound, Two-Dimensional Sonohysterography and Three-Dimensional Ultrasound in the Diagnosis of Septate Uterus—A Systematic Review and Meta-Analysis. Diagnostics. 2023;13(4):807. doi:10.3390/diagnostics13040807

Alcázar JL, Eguez PM, Forcada P, Ternero E, Martínez C, Pascual MÁ, et al. Diagnostic accuracy of sliding sign for detecting pouch of Douglas obliteration and bowel involvement in women with suspected endometriosis: systematic review and meta-analysis. Ultrasound Obstet Gynecol. 2022;60(4):477–86. doi:10.1002/uog.24900

Alcàzar JL, Montoya C, Candau C, Catalan C, Orozco R, Pascual MA, et al. Transvaginal ultrasound for detecting parametrial involvement in suspected deep pelvic endometriosis: updated meta‐analysis. Ultrasound in Obstetrics and Gynecology. 2025.

Aleo E, Barbieri F, Sconfienza L, Zampogna G, Garlaschi G, Cimmino MA. Ultrasound versus low-field magnetic resonance imaging in rheumatic diseases: a systematic literature review. Clin Exp Rheumatol. 2014;32(1 Suppl 80):S91-98.

Alfirevic Z, Stampalija T, Dowswell T. Fetal and umbilical Doppler ultrasound in high-risk pregnancies. Cochrane Database Syst Rev. 2017;6(6):CD007529. doi:10.1002/14651858.CD007529.pub4

Alfirevic Z, Stampalija T, Gyte G. Fetal and umbilical Doppler ultrasound in high-risk pregnancies. Ultrasound in Obstetrics & Gynecology. 2010;36(6):779–80. doi:10.1002/uog.8863

Alfirevic Z, Stampalija T, Gyte GML. Fetal and umbilical Doppler ultrasound in high-risk pregnancies. Cochrane Database Syst Rev. 2013;2013(11):CD007529. doi:10.1002/14651858.CD007529.pub3

Alghamdi D, Kernohan N, Li C, Nabi G. Comparative Assessment of Different Ultrasound Technologies in the Detection of Prostate Cancer: A Systematic Review and Meta-Analysis. Cancers. 2023;15(16):4105. doi:10.3390/cancers15164105

Allan-Blitz LT, Yarbrough C, Ndayizigiye M, Wade C, Goldsmith AJ, Duggan NM. Point-of-care ultrasound for diagnosing extrapulmonary TB. Int J Tuberc Lung Dis. 2024;28(5):217–24. doi:10.5588/ijtld.23.0471

Asferg C, Usinger L, Kristensen TS, Abdulla J. Accuracy of multi-slice computed tomography for measurement of left ventricular ejection fraction compared with cardiac magnetic resonance imaging and two-dimensional transthoracic echocardiography: a systematic review and meta-analysis. Eur J Radiol. 2012;81(5):e757-762. doi:10.1016/j.ejrad.2012.02.002

Awad MW, Salem T, Aboutaleb AM, Abouelatta EH, Ibrahim A, Sayed Serour AI, et al. Can ultrasound be a valuable tool in diagnosis and follow-up of traumatic nerve injury? - A preliminary systematic review and meta-analysis. Neurologie und Rehabilitation. 2023;29(0):s5–6.

Azarpey N. Sensitivity and specificity of ultrasound and mammography for detection of breast malignancy: A systematic review and meta-analysis. 2023.

Babatunde OO, Forsyth JJ. Quantitative Ultrasound and bone’s response to exercise: A meta analysis. Bone. 2013;53(1):311–8. doi:10.1016/j.bone.2012.12.011

Behzadmehr R, Salarzaei M. Is contrast enhanced ultrasonography an accurate way to diagnose gallbladder adenoma? A systematic review and meta-analysis. Journal of Medical Imaging and Radiation Sciences. 2021;52(1):127–36. doi:10.1016/j.jmir.2020.09.014

Bell R, Petticrew M, Sheldon T. The performance of screening tests for ovarian cancer: results of a systematic review. BJOG. 1998;105(11):1136–47. doi:10.1111/j.1471-0528.1998.tb09966.x

Best LMJ, Webb AC, Gurusamy KS, Cheng SF, Richards T. Transcranial Doppler Ultrasound Detection of Microemboli as a Predictor of Cerebral Events in Patients with Symptomatic and Asymptomatic Carotid Disease: A Systematic Review and Meta-Analysis. European Journal of Vascular and Endovascular Surgery. 2016;52(5):565–80. doi:10.1016/j.ejvs.2016.05.019

Bhatia P, Deniwar A, Lahlouh M, Belmont J, Al-Qurayshi ZH, Kandil E. Comparing efficacy of elastography and ultrasound in determination of benign and malignant thyroid nodules: A pooled analysis. Thyroid. 2014;24(S1):P-1-A-123. doi:10.1089/thy.2014.2410.abstracts

Bignotti B, Signori A, Sormani MP, Molfetta L, Martinoli C, Tagliafico A. Ultrasound versus magnetic resonance imaging for Morton neuroma: systematic review and meta-analysis. Eur Radiol. 2015;25(8):2254–62. doi:10.1007/s00330-015-3633-3

Bin L, Huihui Y, Weiping Y, Changyuan W, Qinghong Q, Weiyu M. Value of Three-Dimensional Ultrasound in Differentiating Malignant From Benign Breast Tumors: A Systematic Review and Meta-Analysis. Ultrasound Quarterly. 2019;35(1):68–73. doi:10.1097/RUQ.0000000000000433

Bollegala N, Griller N, Bannerman H, Habal M, Nguyen GC. Ultrasound vs Endoscopy, Surgery, or Pathology for the Diagnosis of Small Bowel Crohn’s Disease and its Complications. Inflammatory Bowel Diseases. 2019;25(8):1313–38. doi:10.1093/ibd/izy392

Brandell K, Reynolds-Wright JJ, Boerma C, Gibson G, Hognert H, Tuladhar H, et al. Medical Abortion before Confirmed Intrauterine Pregnancy: A Systematic Review. Semin Reprod Med. 2022;40(5–06):258–63. doi:10.1055/s-0042-1760117

Brennan ME, Houssami N. Evaluation of the evidence on staging imaging for detection of asymptomatic distant metastases in newly diagnosed breast cancer. The Breast. 2012;21(2):112–23. doi:10.1016/j.breast.2011.10.005

Caliesch R, Hilfiker. Diagnostic test accuracy of clinical tests and ultrasound for the detection of cam and pincer morphology a systematic review. Swiss Medical Weekly. 149:21s–21s.

Cano J, Acosta KR, Bisnar J, Inocencio R, Bocobo J. Diagnostic Performance of Acoustic Radiation Force Impulse Versus Transient Elastography in Assessing Liver Fibrosis: A Meta-analysis. Clinical Gastroenterology and Hepatology. 2014;12(1):156. doi:10.1016/j.cgh.2013.09.030

Cao J, Wang H, Ling W. Compared with SonoVue® LR-5, Sonazoid® modified LR-5 has better diagnostic sensitivity for hepatocellular carcinoma: a systematic review and meta-analysis. Quant Imaging Med Surg. 2024;14(4):2978–92. doi:10.21037/qims-23-1616

Cao S, Wang C, Ma X, Wang X, Huang J, Zhang C. Imaging diagnosis for chronic lateral ankle ligament injury: a systemic review with meta-analysis. J Orthop Surg Res. 2018;13(1):122. doi:10.1186/s13018-018-0811-4

Castro A, Diaz J, Kumar S. Performance of musculoskeletal ultrasonography (MSUS) and optimal cutoff criteria for patients at risk for rheumatoid arthritis: a meta-analysis. Annals of the Rheumatic Diseases. 2018;77:550–1. doi:10.1136/annrheumdis-2018-eular.1752

Chang D, Wang Q. Diagnostic value of ultrasound elastography and conventional ultrasound for thyroid nodules: a meta-analysis. Quant Imaging Med Surg. 2023;13(3):1300–11. doi:10.21037/qims-22-505

Chen B, Chi J, Hou S, Zhang Y, Song S, Liu M, et al. Diagnostic value of contrast-enhanced ultrasound and contrast-enhanced CT for renal solid space-occupying lesions: A meta-analysis. Chinese Journal of Evidence-Based Medicine. 2015;15(9):1042. doi:10.7507/1672-2531.20150174

Chen IJ, Chang KV, Wu WT, Özçakar L. Ultrasound Parameters Other Than the Direct Measurement of Ulnar Nerve Size for Diagnosing Cubital Tunnel Syndrome: A Systemic Review and Meta-analysis. Archives of Physical Medicine and Rehabilitation. 2019;100(6):1114–30. doi:10.1016/j.apmr.2018.06.021

Chen S, Wang X, Zhang B, Liu B, Pan H. Meta-analysis of the diagnostic value of contrast-enhanced ultrasound for the detection of vascular complications after liver transplantation. Rev Esp Enferm Dig. 2018;111. doi:10.17235/reed.2018.5818/2018

Chen WT, Wang MY, Jiang TT, Tang M, Ye QH, Wang HY, et al. Transtracheal ultrasound for confirmation of endotracheal tube placement in the intensive care unit: a systematic review and meta-analysis. Eur Rev Med Pharmacol Sci. 2022;26(22):8224–33. doi:10.26355/eurrev_202211_30354

Chen X, Yu H, Wei N, Ozcan BB, An G, Wu Q, et al. Diagnostic performance of contrast-enhanced ultrasound combined with shear wave elastography in differentiating benign from malignant breast lesions: a systematic review and meta-analysis. Gland Surg. 2023;12(11):1610–23. doi:10.21037/gs-23-333

Chen Y, Zhu W, Yi Y, Cao H, Chen F, Zhu Q. Perfluorobutane (Sonazoid) Contrast-enhanced ultrasound to diagnose hepatocellular carcinoma: A systematic review and meta-analysis. Med Ultrason. 2024;26(2):187–96. doi:10.11152/mu-4334

Chen Y, Zhu Y, Chen K, Wang H, Zhang W, Bao J, et al. Differentiation between hepatocellular carcinoma and intrahepatic cholangiocarcinoma using contrast-enhanced ultrasound: A systematic review and meta-analysis. Clin Hemorheol Microcirc. 2021;79(2):293–309. doi:10.3233/CH-211145

Chen YH, Zhang YQ. Diagnostic potential of ultrasonography and computed tomography in differentiating cervical lymph node metastasis of thyroid cancer: a systematic review and meta-analysis. Arch Med Sci. 2020. doi:10.5114/aoms.2020.95104

Cheng Y, Wang M, Ma B, Ma X. Potential role of contrast-enhanced ultrasound for the differentiation of malignant and benign gallbladder lesions in East Asia: A meta-analysis and systematic review. Medicine. 2018;97(33):e11808. doi:10.1097/MD.0000000000011808

Cheung K, Wang TS, Farrokhyar F, Roman SA, Sosa JA. A meta-analysis of preoperative localization techniques for patients with primary hyperparathyroidism. Ann Surg Oncol. 2012;19(2):577–83. doi:10.1245/s10434-011-1870-5

Chien PF, Arnott N, Gordon A, Owen P, Khan KS. How useful is uterine artery Doppler flow velocimetry in the prediction of pre-eclampsia, intrauterine growth retardation and perinatal death? An overview. BJOG. 2000;107(2):196–208. doi:10.1111/j.1471-0528.2000.tb11690.x

Chowdhury SR, Datta PK, Maitra S, Rawat D, Baidya DK, Roy A, et al. The use of preoperative inferior vena cava ultrasound to predict anaesthesia-induced hypotension: a systematic review. Anaesthesiol Intensive Ther. 2023;55(1):18–31. doi:10.5114/ait.2023.125310

Chung J, Kordzadeh A, Prionidis I, Panayiotopoulos Y, Browne T. Contrast-enhanced ultrasound (CEUS) versus computed tomography angiography (CTA) in detection of endoleaks in post-EVAR patients. Are delayed type II endoleaks being missed? A systematic review and meta-analysis. J Ultrasound. 2015;18(2):91–9. doi:10.1007/s40477-014-0154-x

Chuter VH, Searle A, Barwick A, Golledge J, Leigh L, Oldmeadow C, et al. Estimating the diagnostic accuracy of the ankle–brachial pressure index for detecting peripheral arterial disease in people with diabetes: A systematic review and meta‐analysis. Diabet Med. 2021;38(2):e14379. doi:10.1111/dme.14379

Colli A, Fraquelli M, Casazza G, Massironi S, Colucci A, Conte D, et al. Accuracy of ultrasonography, spiral CT, magnetic resonance, and alpha-fetoprotein in diagnosing hepatocellular carcinoma: a systematic review. Am J Gastroenterol. 2006;101(3):513–23. doi:10.1111/j.1572-0241.2006.00467.x

Colli A, Nadarevic T, Miletic D, Giljaca V, Fraquelli M, Štimac D, et al. Abdominal ultrasound and alpha-foetoprotein for the diagnosis of hepatocellular carcinoma in adults with chronic liver disease. Cochrane Database Syst Rev. 2021;4(4):CD013346. doi:10.1002/14651858.CD013346.pub2

Colli A, Nadarevic T, Miletic D, Giljaca V, Fraquelli M, Štimac D, et al. Abdominal ultrasound and alpha-foetoprotein for the diagnosis of hepatocellular carcinoma. A diagnostic test accuracy Cochrane review. Digestive and Liver Disease. 2021;Abstracts of the 53rd Virtual A.I.S.F. – Italian Association for the Study of the Liver – Annual Meeting 2021, Rome, March 3rd-5th, 202153:S36. doi:10.1016/j.dld.2020.12.087

Collins R, Cranny G, Burch J, Aguiar-Ibáñez R, Craig D, Wright K, et al. A systematic review of duplex ultrasound, magnetic resonance angiography and computed tomography angiography for the diagnosis and assessment of symptomatic, lower limb peripheral arterial disease. Health Technol Assess. 2007;11(20):iii–iv, xi–xiii, 1–184. doi:10.3310/hta11200

Conde-Agudelo A, Romero R. Predictive accuracy of changes in transvaginal sonographic cervical length over time for preterm birth: a systematic review and metaanalysis. Am J Obstet Gynecol. 2015;213(6):789–801. doi:10.1016/j.ajog.2015.06.015

Conti CB, Giunta M, Gridavilla D, Conte D, Fraquelli M. Role of Bowel Ultrasound in the Diagnosis and Follow-up of Patients with Crohn’s Disease. Ultrasound in Medicine & Biology. 2017;43(4):725–34. doi:10.1016/j.ultrasmedbio.2016.12.014

Dai X, Sui X, Chen S, Zhao B, Liu Z, Wang X. The diagnostic performance of salivary gland ultrasound elastography in Sjögren’s syndrome and sicca symptoms: a systematic review and meta-analysis. Eur Radiol. 2023;34(3):1545–55. doi:10.1007/s00330-023-10166-5

Darnis B, Mohkam K, Cauchy F, Cazauran JB, Bancel B, Rode A, et al. A systematic review of the anatomical findings of multiple gallbladders. HPB. 2018;20(11):985–91. doi:10.1016/j.hpb.2018.04.002

Darvish L, Khezri M, Teshnizi SH, Roozbeh N, Dehkordi JG, Amraee A. Color Doppler ultrasonography diagnostic value in detection of malignant nodules in cysts with pathologically proven thyroid malignancy: a systematic review and meta-analysis. Clin Transl Oncol. 2019;21(12):1712–29. doi:10.1007/s12094-019-02105-y

de Melo PFMV, Roever L, Mendonça TMS, da Silva Costa F, Rolnik DL, Diniz ALD. Ophthalmic artery Doppler in the complementary diagnosis of preeclampsia: a systematic review and meta-analysis. BMC Pregnancy Childbirth. 2023;23(1):343. doi:10.1186/s12884-023-05656-9

Delli K, Dijkstra P, Stel A, Bootsma H, Vissink A, Spijkervet F. Diagnostic properties of ultrasound of major salivary glands in Sjögren’s syndrome: a meta‐analysis. Oral Diseases. 2015;21(6):792–800. doi:10.1111/odi.12349

Deng H, Lei J, Jin L, Shi H. Diagnostic efficacy of sentinel lymph node in breast cancer under percutaneous contrast‐enhanced ultrasound: An updated meta‐analysis. Thoracic Cancer. 2021;12(21):2849–56. doi:10.1111/1759-7714.14139

Deng H, Shi H, Lei J, Hu Y, Li G, Wang C. A meta-analysis of contrast-enhanced ultrasound for small hepatocellular carcinoma diagnosis. J Cancer Res Ther. 2016;12(Supplement):C274–6. doi:10.4103/0973-1482.200756

Desai D, Patel S, Shah H, Shah A, Verma A, Malafi ME. Diagnostic accuracy of CEUS in identifying Solid Pancreatic Lesions:- A meta analysis. Surgery; 2023. doi:10.1101/2023.08.12.23294023

Descatha A, Huard L, Aubert F, Barbato B, Gorand O, Chastang JF. Meta-Analysis on the Performance of Sonography for the Diagnosis of Carpal Tunnel Syndrome. Seminars in Arthritis and Rheumatism. 2012;41(6):914–22. doi:10.1016/j.semarthrit.2011.11.006

Ding Y, Peng Y, Zhang J, Pan X, Huang X, Zhang CQ. Diagnostic value of contrast-enhanced ultrasound in the diagnosis of papillary thyroid microcarcinoma: A systematic review and meta-analysis. Medicine (Baltimore). 2024;103(15):e37768. doi:10.1097/MD.0000000000037768

Dong J, Wang H, Zhao J, Zhu W, Zhang L, Gong J, et al. Ultrasound as a diagnostic tool in detecting active Crohn’s disease: a meta-analysis of prospective studies. Eur Radiol. 2014;24(1):26–33. doi:10.1007/s00330-013-2973-0

Dong S, Hou J, Zhang C, Lu G, Qin W, Huang L, et al. Diagnostic Performance of Atherosclerotic Carotid Plaque Neovascularization with Contrast-Enhanced Ultrasound: A Meta-Analysis. Tang M, editor. Computational and Mathematical Methods in Medicine. 2022;2022:1–8. doi:10.1155/2022/7531624

Drew MK, Osmotherly PG, Chiarelli PE. Imaging and clinical tests for the diagnosis of long-standing groin pain in athletes. A systematic review. Physical Therapy in Sport. 2014;15(2):124–9. doi:10.1016/j.ptsp.2013.11.002

Duan Y, Dong B, Li S, Zhang C. Diagnostic accuracy of contrast-enhanced ultrasound for detecting clinically significant portal hypertension and severe portal hypertension in chronic liver disease: a meta-analysis. Expert Rev Gastroenterol Hepatol. 2023;17(5):489–97. doi:10.1080/17474124.2023.2196402

Duarte ML, Iared W, Oliveira ASB, Santos LRD, Peccin MS. Ultrasound versus electromyography for the detection of fasciculation in amyotrophic lateral sclerosis: systematic review and meta-analysis. Radiol Bras. 2020;53(2):116–21. doi:10.1590/0100-3984.2019.0055

ELgenidy A, Abdelwahab A, Jalal PK. GALLBLADDER WALL THICKENING MEASUREMENT AS NON-INVASIVE SCREENING FOR ESOPHAGEAL VARICES: SYSTEMATIC REVIEW AND META-ANALYSIS. Gastroenterology. 2022;162(7):S-1180-S-1181. doi:10.1016/S0016-5085(22)63513-3

Elkaryoni A, Altibi AM, Hassan A, Elgebaly A, Elsabbagh EM, Nanda NC. Diagnostic Accuracy of Dobutamine Stress Echocardiography in the Detection of Cardiac Allograft Vasculopathy in Heart Transplant Recipients: A Systematic Review and Meta-Analysis Study. Circulation. 2018;138(Suppl_1):A11295–A11295. doi:10.1161/circ.138.suppl_1.11295

Fei F, Zhuang H, Shi W, Yu H. Diagnostic value of lung ultrasound in diagnosing postoperative atelectasis, pleural effusion and pneumothorax in adult patients: a systematic review and meta-analysis. Chinese Journal of Anesthesiology. 2023;43(07):802. doi:10.3760/cma.j.cn131073.20230309.00708

Figueroa Córdoba AV, Bertazzo BB, Gonzalez Grima J, Martinez ML, Konicoff M, Martina L, et al. [Effectiveness of transthoracic echocardiogram in patent foramen ovale diagnosis. Systematic review of last ten years.]. Rev Fac Cien Med Univ Nac Cordoba. 2019;76(4):211–6. doi:10.31053/1853.0605.v76.n4.23988

Filho RHC, Pereira FL, Iared W. Diagnostic Accuracy Evaluation of Two-Dimensional Shear Wave Elastography in the Differentiation Between Benign and Malignant Thyroid Nodules: Systematic Review and Meta-analysis. J Ultrasound Med. 2020;39(9):1729–41. doi:10.1002/jum.15271

Filipe MD, Patuleia SIS, De Jong VMT, Vriens MR, Van Diest PJ, Witkamp AJ. Network Meta-analysis for the Diagnostic Approach to Pathologic Nipple Discharge. Clinical Breast Cancer. 2020;20(6):e723–48. doi:10.1016/j.clbc.2020.05.015

Floriani I, D’Onofrio M, Rulli E, Chen MH, Li R, Musicco L. Performance of Imaging Modalities in the Diagnosis of Hepatocellular Carcinoma: a Systematic Review and Meta-Analysis. Ultraschall in Med. 2012;34(05):454–62. doi:10.1055/s-0032-1330358

Forsythe RO, Apelqvist J, Boyko EJ, Fitridge R, Hong JP, Katsanos K, et al. Effectiveness of bedside investigations to diagnose peripheral artery disease among people with diabetes mellitus: A systematic review. Diabetes/Metabolism Research and Reviews. 2020;36(S1):e3277. doi:10.1002/dmrr.3277

Fosser C, Hutmacher MM, Gupta P, Krishnaswami S, Tortorici MA. An assessment of the operating characteristics (OCS) of time-to-event (TTE) exposure-response (ER) analyses of adverse events (AES). Clinical Pharmacology and Therapeutics. 95(0):s58–9.

Frank E, Ale‐Salvo D, Park J, Liu Y, Simental A, Inman JC. Preoperative imaging for parathyroid localization in patients with concurrent thyroid disease: A systematic review. Head & Neck. 2018;40(7):1577–87. doi:10.1002/hed.25111

Fraquelli M, Nadarevic T, Colli A, Manzotti C, Giljaca V, Miletic D, et al. Contrast-enhanced ultrasound for the diagnosis of hepatocellular carcinoma in adults with chronic liver disease. Cochrane Database Syst Rev. 2022;9(9):CD013483. doi:10.1002/14651858.CD013483.pub2

Friedrich-Rust M, Klopffleisch T, Nierhoff J, Herrmann E, Schneider M, Vermehren J, et al. Meta-analysis of contrast-enhanced ultrasound for the differentiation of benign and malignant focal liver lesions. Hepatology. 2012;56(S1):191A-1144A. doi:10.1002/hep.26040

Friedrich‐Rust M, Klopffleisch T, Nierhoff J, Herrmann E, Vermehren J, Schneider MD, et al. Contrast‐Enhanced Ultrasound for the differentiation of benign and malignant focal liver lesions: a meta‐analysis. Liver International. 2013;33(5):739–55. doi:10.1111/liv.12115

Fu H, Wang L, Zhang W, Lu J, Yang M. Diagnostic test accuracy of ultrasound for sarcopenia diagnosis: A systematic review and meta-analysis. J Cachexia Sarcopenia Muscle. 2023;14(1):57–70. doi:10.1002/jcsm.13149

Fung Kee Fung M, Bryson P, Johnston M, Chambers A. Screening Postmenopausal Women for Ovarian Cancer: A Systematic Review. Journal of Obstetrics and Gynaecology Canada. 2004;26(8):717–28. doi:10.1016/S1701-2163(16)30643-0

Furrer MA, Spycher SCJ, Büttiker SM, Gross T, Bosshard P, Thalmann GN, et al. Comparison of the Diagnostic Performance of Contrast-enhanced Ultrasound with That of Contrast-enhanced Computed Tomography and Contrast-enhanced Magnetic Resonance Imaging in the Evaluation of Renal Masses: A Systematic Review and Meta-analysis. European Urology Oncology. 2020;3(4):464–73. doi:10.1016/j.euo.2019.08.013

Gao LY, Wang Y, Jiang YX, Yang X, Liu RY, Xi XH, et al. Ultrasound is helpful to differentiate Bethesda class III thyroid nodules: A PRISMA-compliant systematic review and meta-analysis. Medicine. 2017;96(16):e6564. doi:10.1097/MD.0000000000006564

Gaughran J, Naji O, Murad MA, Sayasneh A. Can ultrasound reliably assess ovarian endometriomas in pregnancy? A systematic review. BJOG: An International Journal of Obstetrics and Gynaecology. 129:91–91.

Gaughran JE, Naji O, Al Sabbagh MQ, Sayasneh A. Is Ultrasound a Reliable and Reproducible Method for Assessing Adnexal Masses in Pregnancy? A Systematic Review. Cureus. 2021. doi:10.7759/cureus.19079

Ge X, Lan ZK, Chen J, Zhu SY. Effectiveness of contrast-enhanced ultrasound for detecting the staging and grading of bladder cancer: a systematic review and meta-analysis. Medical Ultrasonography. 2021;23(1):29–35. doi:10.11152/mu-2730

Geng J, Tang J. Contrast-enhanced ultrasound in the diagnosis of endometrial carcinoma: A meta-analysis. Exp Ther Med. 2018;16(6):5310–4. doi:10.3892/etm.2018.6889

Giri S, Vaidya A, Agrawal D, Varghese J, Patel RK, Tripathy T, et al. Role of contrast‐enhanced ultrasound for differentiation of benign vs. malignant portal vein thrombosis in hepatocellular carcinoma – A systematic review a meta‐analysis. Australas J Ultrason Med. 2024;27(1):56–64. doi:10.1002/ajum.12375

Goodsall TM, Nguyen TM, Parker CE, Ma C, Andrews JM, Jairath V, et al. Systematic Review: Gastrointestinal Ultrasound Scoring Indices for Inflammatory Bowel Disease. Journal of Crohn’s and Colitis. 2021;15(1):125–42. doi:10.1093/ecco-jcc/jjaa129

Groeneveld NS, Guglielmi V, Leeflang MMG, Matthijs Boekholdt S, Nils Planken R, Roos YBWEM, et al. CT angiography vs echocardiography for detection of cardiac thrombi in ischemic stroke: a systematic review and meta-analysis. J Neurol. 2020;267(6):1793–801. doi:10.1007/s00415-020-09766-8

Guang Y, Xie L, Ding H, Cai A, Huang Y. Diagnosis value of focal liver lesions with SonoVue®-enhanced ultrasound compared with contrast-enhanced computed tomography and contrast-enhanced MRI: a meta-analysis. J Cancer Res Clin Oncol. 2011;137(11):1595–605. doi:10.1007/s00432-011-1035-8

Guang Y, Xie. Contrast-enhanced ultrasound and contrast-enhanced CT for diagnosis of focal liver lesions: Meta-analysis. Chin J Med Imaging Technol. 2009.

Gunda ST, Yip JHY, Ng VTK, Chen Z, Han X, Chen X, et al. The Diagnostic Accuracy of Transcranial Color-Coded Doppler Ultrasound Technique in Stratifying Intracranial Cerebral Artery Stenoses in Cerebrovascular Disease Patients: A Systematic Review and Meta-Analysis. J Clin Med. 2024;13(5):1507. doi:10.3390/jcm13051507

Gutierrez M, Rodriguez S, Soto-Fajardo C, Santos-Moreno P, Sandoval H, Bertolazzi C, et al. Ultrasound of sacroiliac joints in spondyloarthritis: a systematic review. Rheumatol Int. 2018;38(10):1791–805. doi:10.1007/s00296-018-4126-x

Gyftopoulos S, Cardoso MDS, Rodrigues TC, Qian K, Chang CY. Postoperative Imaging of the Rotator Cuff: A Systematic Review and Meta-Analysis. American Journal of Roentgenology. 2022;219(5):717–23. doi:10.2214/AJR.22.27847

Hai Y, Chong W, Eisenbrey JR, Forsberg F. Network Meta-Analysis: Noninvasive Imaging Modalities for Identifying Clinically Significant Portal Hypertension. Dig Dis Sci. 2022;67(7):3313–26. doi:10.1007/s10620-021-07168-y

Hai Y, Chong W, Liu J bin, Forsberg F, Eisenbrey J. The Diagnostic Value of Contrast-Enhanced Ultrasound for Monitoring Complications After Kidney Transplantation—A Systematic Review and Meta-Analysis. Academic Radiology. 2021;28(8):1086–93. doi:10.1016/j.acra.2020.05.009

Hai Y, Savsani E, Chong W, Eisenbrey J, Lyshchik A. Meta-analysis and systematic review of contrast-enhanced ultrasound in evaluating the treatment response after locoregional therapy of hepatocellular carcinoma. Abdom Radiol. 2021;46(11):5162–79. doi:10.1007/s00261-021-03248-9

Hanna RF, Miloushev VZ, Tang A, Finklestone LA, Brejt SZ, Sandhu RS, et al. Comparative 13-year meta-analysis of the sensitivity and positive predictive value of ultrasound, CT, and MRI for detecting hepatocellular carcinoma. Abdom Radiol (NY). 2016;41(1):71–90. doi:10.1007/s00261-015-0592-8

Harky A, Zywicka E, Santoro G, Jullian L, Joshi M, Dimitri S. Is contrast-enhanced ultrasound (CEUS) superior to computed tomography angiography (CTA) in detection of endoleaks in post-EVAR patients? A systematic review and meta-analysis. J Ultrasound. 2019;22(1):65–75. doi:10.1007/s40477-019-00364-7

Heim B, Peball M, Hammermeister J, Djamshidian A, Krismer F, Seppi K. Differentiating Parkinson’s Disease from Essential Tremor Using Transcranial Sonography: A Systematic Review and Meta-Analysis. JPD. 2022;12(4):1115–23. doi:10.3233/JPD-213012

Hernaez R, Lazo M, Bonekamp S, Kamel I, Brancati FL, Guallar E, et al. Diagnostic accuracy and reliability of ultrasonography for the detection of fatty liver: A meta-analysis. Hepatology. 2011;54(3):1082–90. doi:10.1002/hep.24452

Hirooka M, Koizumi Y, Sunago K, Nakamura Y, Hirooka K, Watanabe T, et al. Efficacy of B-mode ultrasound-based attenuation for the diagnosis of hepatic steatosis: a systematic review/meta-analysis. J Med Ultrasonics. 2022;49(2):199–210. doi:10.1007/s10396-022-01196-5

Hirooka M, Koizumi Y, Yukimoto A, Sunago K, Watanabe T, Yoshida O, et al. Efficacy of the b-mode ultrasound-based attenuation coefficient for the diagnosis of hepatic steatosis: A meta-analysis. Hepatology. 2021;74(S1):157–1288. doi:10.1002/hep.32188

Holmes M, Rathbone J, Littlewood C, Rawdin A, Stevenson M, Stevens J, et al. Routine echocardiography in the management of stroke and transient ischaemic attack: a systematic review and economic evaluation. Health Technol Assess. 2014;18(16):1–176. doi:10.3310/hta18160

Hong S, Li W, Gao W, Liu M, Song D, Dong Y, et al. Diagnostic performance of elastography for breast non-mass lesions: A systematic review and meta-analysis. European Journal of Radiology. 2021;144:109991. doi:10.1016/j.ejrad.2021.109991

Hosseini RS, Ebrahimi PS, Shokrani A. Diagnostic value of enhanced-contrast ultrasound for cesarean scar pregnancy: A systematic review. Eur J Obstet Gynecol Reprod Biol. 2024;293:119–24. doi:10.1016/j.ejogrb.2023.12.028

Hosseini SM, Gity M, Talebi Boroujeni P, Asghari Ahmadabad M, Jahanshahi A. Application of Elastography in the Diagnosis of Idiopathic Granulomatous Mastitis (IGM): A Systematic Review. Iran J Radiol. 2023;20(3). doi:10.5812/iranjradiol-136102

Hu Q, Wang XY, Zhu SY, Kang LK, Xiao YJ, Zheng HY. Meta-analysis of contrast-enhanced ultrasound for the differentiation of benign and malignant breast lesions. Acta Radiol. 2015;56(1):25–33. doi:10.1177/0284185113517115

Hu Y, Liu J, Quan F. Diagnostic value of transcranial sonography for patients with Parkinson disease in China: Meta-analysis. Chinese Journal of Medical Imaging Technology. 2018;34(8):1173–7.

Huang J, Chen W, Yao S. Assessing diagnostic value of contrast-enhanced ultrasound and contrast-enhanced computed tomography in detecting small hepatocellular carcinoma: A meta-analysis. Medicine (Baltimore). 2017;96(30):e7555. doi:10.1097/MD.0000000000007555

Huang R, Abdelmoneim SS, Ball CA, Nhola LF, Farrell AM, Feinstein S, et al. Detection of Carotid Atherosclerotic Plaque Neovascularization Using Contrast Enhanced Ultrasound: A Systematic Review and Meta-Analysis of Diagnostic Accuracy Studies. J Am Soc Echocardiogr. 2016;29(6):491–502. doi:10.1016/j.echo.2016.02.012

Huang R, Jiang L, Xu Y, Gong Y, Ran H, Wang Z, et al. Comparative Diagnostic Accuracy of Contrast-Enhanced Ultrasound and Shear Wave Elastography in Differentiating Benign and Malignant Lesions: A Network Meta-Analysis. Front Oncol. 2019;9:102. doi:10.3389/fonc.2019.00102

Huang S, Zhao Y, Jiang X, Lin N, Zhang M, Wang H, et al. Clinical Utility of Contrast-enhanced Ultrasound for the Diagnosis of Lymphadenopathy. Ultrasound in Medicine & Biology. 2021;47(4):869–79. doi:10.1016/j.ultrasmedbio.2020.12.020

Huang X wen, Huang Q xiu, Huang H, Cheng M qing, Tong W juan, Xian M fei, et al. Diagnostic Performance of Quantitative and Qualitative Elastography for Axillary Lymph Node Metastasis in Breast Cancer: A Systematic Review and Meta-Analysis. Front Oncol. 2020;10:552177. doi:10.3389/fonc.2020.552177

Huang Y, Pang Y, Wang Q, Li JY. [Evaluation on the accuracy of high-frequency ultrasound being used in the breast cancer screening program in women from Asian countries: a systematic review]. Zhonghua Liu Xing Bing Xue Za Zhi. 2010;31(11):1296–9.

Hung KC, Huang YT, Tsai WW, Tan PH, Wu JY, Huang PY, et al. Diagnostic Efficacy of Carotid Ultrasound for Predicting the Risk of Perioperative Hypotension or Fluid Responsiveness: A Meta-Analysis. Diagnostics. 2023;13(13):2290. doi:10.3390/diagnostics13132290

Huo YL, Jiao D, Yang DY, Zheng B, Song XT. A meta analysis on diagnostic effectiveness of ultrasound in diagnosis of acute pulmonary edema in ICU.

Jahromi AS, Cinà CS, Liu Y, Clase CM. Sensitivity and specificity of color duplex ultrasound measurement in the estimation of internal carotid artery stenosis: A systematic review and meta-analysis. Journal of Vascular Surgery. 2005;41(6):962–72. doi:10.1016/j.jvs.2005.02.044

Jang JK, Choi SH, Lee JS, Kim SY, Lee SS, Kim KW. Accuracy of the ultrasound attenuation coefficient for the evaluation of hepatic steatosis: a systematic review and meta-analysis of prospective studies. Ultrasonography. 2022;41(1):83–92. doi:10.14366/usg.21076

Jawli A, Nabi G, Huang Z. The Performance of Different Parametric Ultrasounds in Prostate Cancer Diagnosis: Correlation with Radical Prostatectomy Specimens. Cancers. 2024;16(8):1502. doi:10.3390/cancers16081502

Jayanth A, Benabbas R, Chao J, Sinert R. Diagnostic modalities to determine ventriculoperitoneal shunt malfunction: A systematic review and meta-analysis. Am J Emerg Med. 2021;39:180–9. doi:10.1016/j.ajem.2020.09.024

Jerjes W, Hamdoon Z, Abbas S, Algamal Z. The efficacy of preoperative neck ultrasound in the detection of cervical lymph node metastasis: a systematic review. British Journal of Oral and Maxillofacial Surgery. 2011;49:S68. doi:10.1016/j.bjoms.2011.03.125

Jia K, Li L, Wu XJ, Hao MJ, Xue HY. Contrast-enhanced ultrasound for evaluating the pathologic response of breast cancer to neoadjuvant chemotherapy: A meta-analysis. Medicine (Baltimore). 2019;98(4):e14258. doi:10.1097/MD.0000000000014258

Jiang H, Chen J, Jiang J, Ye Z. [Diagnostic value of contrast-enhanced ultrasound for small hepatocellular carcinoma in cirrhotic patients: a meta-analysis]. Chinese journal of hepatology. 2012;20(11):828. doi:10.3760/cma.j.issn.1007-3418.2012.11.006

Jiang L, Zhang D, Chen YN, Yu XJ, Pan MF, Lian L. The value of conventional ultrasound combined with superb microvascular imaging and color Doppler flow imaging in the diagnosis of thyroid malignant nodules: a systematic review and meta-analysis. Front Endocrinol. 2023;14:1182259. doi:10.3389/fendo.2023.1182259

Jiang Y, Wu H, Yang D, Wang W, Chu J, Tang J, et al. Ultrasound for Osteoporosis in Elderly Women: A Meta-Analysis. Alternative therapies in health and medicine. 2023.

Jin H, Wang C, Jin X. Superb microvascular imaging for distinguishing thyroid nodules: A meta-analysis (PRISMA) [RETRACTED]. Medicine. 2022;101(24):e29505. doi:10.1097/MD.0000000000029505

Jones CM, Athanasiou T, Tekkis PP, Malinovski V, Purkayastha S, Haq A, et al. Does Doppler echography have a diagnostic role in patency assessment of internal thoracic artery grafts? Eur J Cardiothorac Surg. 2005;28(5):692–700. doi:10.1016/j.ejcts.2005.07.013

Jones SA, Arezina J. What is the diagnostic accuracy of 3D ultrasound in comparison to MRI for uterine anomalies? Ultrasound. 2019;27(2). doi:10.1177/1742271X19840271

Kapetanios D, Kontopodis N, Mavridis D, McWilliams RG, Giannoukas AD, Antoniou GA. Meta-analysis of the accuracy of contrast-enhanced ultrasound for the detection of endoleak after endovascular aneurysm repair. Journal of Vascular Surgery. 2019;69(1):280-294.e6. doi:10.1016/j.jvs.2018.07.044

Karthikesalingam A, Al-Jundi W, Jackson D, Boyle JR, Beard JD, Holt PJE, et al. Systematic review and meta-analysis of duplex ultrasonography, contrast-enhanced ultrasonography or computed tomography for surveillance after endovascular aneurysm repair. British Journal of Surgery. 2012;99(11):1514–23. doi:10.1002/bjs.8873

Katsanos A. Transcranial Doppler versus transthoracic echocardiography for the detection of patent foramen ovale in cryptogenic cerebral ischemia: A systematic review and diagnostic test accuracy meta‐analysis. 2015.

Katsanos AH, Psaltopoulou T, Sergentanis TN, Frogoudaki A, Vrettou AR, Ikonomidis I, et al. Transcranial Doppler versus transthoracic echocardiography for the detection of patent foramen ovale in patients with cryptogenic cerebral ischemia: A systematic review and diagnostic test accuracy meta-analysis. Annals of Neurology. 2016;79(4):625–35. doi:10.1002/ana.24609

Khan R, Karim MN, Hosseini F, Fine N. Diagnostic Accuracy of Transthoracic Echocardiography With Contrast for Detection of Right-to-Left Shunt: A Systematic Review and Meta-analysis. Can J Cardiol. 2022;38(12):1948–58. doi:10.1016/j.cjca.2022.08.007

Khazaaleh S, Alomari M, Musa R, Momani LA, Alnasarat A, Rahman AU. ACCURACY OF BOWEL ULTRASOUND AS A DIAGNOSTIC TOOL FOR DETECTING POST-SURGICAL CROHN’S DISEASE RECURRENCE: A SYSTEMATIC REVIEW AND UPDATED META-ANALYSIS. Gastroenterology. 2023;164(6):S-297. doi:10.1016/S0016-5085(23)01670-0

Khouri S. Meta-Analysis of the Accuracy of Doppler Echocardiography in the Assessment of Pulmonary Artery Systolic Pressure. Journal of Cardiac Failure. 2010;16(8):s114.

Kickel C, Liberto J, Quigley B, Tahir M. Utility of Intestinal Ultrasound (IUS) and Its Comparison to Other Diagnostic Modalities in Patients With Crohn’s Disease: A Systematic Review and Meta-Analysis. Am J Gastroenterol. 2022;117(10S):e606–7. doi:10.14309/01.ajg.0000859996.88100.32

Kim JY, Suh YJ, Han K, Kim YJ, Choi BW. Diagnostic Value of Advanced Imaging Modalities for the Detection and Differentiation of Prosthetic Valve Obstruction: A Systematic Review and Meta-Analysis. JACC Cardiovasc Imaging. 2019;12(11 Pt 1):2182–92. doi:10.1016/j.jcmg.2018.11.033

Kinkel K, Lu Y, Both M, Warren RS, Thoeni RF. Detection of hepatic metastases from cancers of the gastrointestinal tract by using noninvasive imaging methods (US, CT, MR imaging, PET): a meta-analysis. Radiology. 2002;224(3):748–56. doi:10.1148/radiol.2243011362

Klarich S, White H. Can ultrasound strain elastography (USE) improve management of suspicious thyroid nodules measuring <10 mm? A systematic review. Radiography. 2023;29(3):661–7. doi:10.1016/j.radi.2023.04.015

Kobayashi N, Kamono E, Yamamoto Y, Yukizawa Y, Honda H, Takagawa S, et al. Imaging Diagnosis, Prevalence, and Clinical Outcomes of Arthroscopic Surgery for Anterior Inferior Iliac Spine Impingement: A Systematic Review and Meta-analysis. Orthopaedic Journal of Sports Medicine. 2022;10(11):23259671221131341. doi:10.1177/23259671221131341

Kruse KK, Dilisio MF, Wang WL, Schmidt CC. Do we really need to order magnetic resonance imaging? Shoulder surgeon ultrasound practice patterns and beliefs. JSES Open Access. 2019;3(2):93–8. doi:10.1016/j.jses.2019.01.004

Lacerda Macedo AC, Carvalho G, Uggioni MLR, Bavaresco DV, Simon CS, Cruz M, et al. Accuracy of Ultrasonography in Breast Implant Rupture Diagnosis: Systematic Review and Meta-Analysis. Plast Reconstr Surg. 2021;148(5):939–47. doi:10.1097/PRS.0000000000008408

Lastoria DA. Comparing Modalities for the Detection of Lymph Node Metastasis in Papillary Thyroid Carcinoma: A Systematic Review and Meta-Analysis. British Journal of Surgery. 2023;110(Supplement_7):znad258.636. doi:10.1093/bjs/znad258.636

Le Boulc’h M, Gilhodes J, Steinmeyer Z, Molière S, Mathelin C. Pretherapeutic Imaging for Axillary Staging in Breast Cancer: A Systematic Review and Meta-Analysis of Ultrasound, MRI and FDG PET. JCM. 2021;10(7):1543. doi:10.3390/jcm10071543

Lee DI, You MW, Park SH, Seo M, Park SJ. Comparison of Diagnostic Performance of Ultrasonography and Magnetic Resonance Enterography in the Assessment of Active Bowel Lesions in Patients with Crohn’s Disease: A Systematic Review and Meta-Analysis. Diagnostics. 2022;12(8):2008. doi:10.3390/diagnostics12082008

Lee TM, Wu WT, Chiu YH, Chang KV, Özçakar L. Ultrasound Imaging in Predicting the Autograft Size in Anterior Cruciate Ligament Reconstruction: A Systematic Review and Meta-Analysis. JCM. 2022;11(13):3876. doi:10.3390/jcm11133876

Leng XF, Zhu Y, Wang GP, Jin J, Xian L, Zhang YH. Accuracy of ultrasound for the diagnosis of cervical lymph node metastasis in esophageal cancer: a systematic review and meta-analysis. J Thorac Dis. 2016;8(8):2146–57. doi:10.21037/jtd.2016.07.71

Leng XF, Zhu Y, Wang GP, Jin J, Xian L, Zhang YH. Accuracy of ultrasound for the diagnosis of cervical lymph node metastasis in esophageal cancer: a systematic review and meta-analysis. Journal of Thoracic Disease. 2016;8(8). doi:10.21037/jtd.2016.07.71

Leong A, Johnson A, Sugrue M. A meta-analysis: Nipple discharge probabilities and diagnostic accuracy of investigations. Ir J Med Sci. 2016;185(S2):57–137. doi:10.1007/s11845-015-1396-0

Li C, Zhang C, Li N, Li J. Compression Real-time Elastography for Evaluation of Salivary Gland Lesions: A Meta-analysis. J Ultrasound Med. 2016;35(5):999–1007. doi:10.7863/ultra.15.08043

Li DH, He YC, Liu J, Chen SD. Diagnostic Accuracy of Transcranial Sonography of the Substantia Nigra in Parkinson’s disease: A Systematic Review and Meta-analysis. Sci Rep. 2016;6(1):20863. doi:10.1038/srep20863

Li J, Wang SR, Li QL, Zhu T, Zhu PS, Chen M, et al. Diagnostic value of multiple ultrasound diagnostic techniques for axillary lymph node metastases in breast cancer: A systematic analysis and network meta-analysis. Front Oncol. 2023;12:1043185. doi:10.3389/fonc.2022.1043185

Li L, Hu Y, Han J, Li Q, Peng C, Zhou J. Clinical Application of Liver Imaging Reporting and Data System for Characterizing Liver Neoplasms: A Meta-Analysis. Diagnostics. 2021;11(2):323. doi:10.3390/diagnostics11020323

Li L, Xia Z, Zeng X, Tang A, Wang L, Su Y. The agreement of different techniques for muscle measurement in diagnosing sarcopenia: a systematic review and meta-analysis. Quant Imaging Med Surg. 2024;14(3):2177–92. doi:10.21037/qims-23-1089

Li Q, Cai M, Wang H, Chen L. Diagnostic Performance of Contrast‐Enhanced Ultrasound and High‐Resolution Magnetic Resonance Imaging for Carotid Atherosclerotic Plaques: A Systematic Review and Meta‐Analysis. J of Ultrasound Medicine. 2023;42(3):739–49. doi:10.1002/jum.16122

Li Q, Hu M, Chen Z, Li C, Zhang X, Song Y, et al. Meta-Analysis: Contrast-Enhanced Ultrasound Versus Conventional Ultrasound for Differentiation of Benign and Malignant Breast Lesions. Ultrasound in Medicine & Biology. 2018;44(5):919–29. doi:10.1016/j.ultrasmedbio.2018.01.022

Li XZ, Song J, Sun ZX, Yang YY, Wang H. Diagnostic performance of contrast-enhanced ultrasound for pancreatic neoplasms: A systematic review and meta-analysis. Dig Liver Dis. 2018;50(2):132–8. doi:10.1016/j.dld.2017.10.012

Li Y, Cao Q, Hou Z, Tang B, Shen Y. Transcranial Sonography as a Diagnostic Tool for Depressive Disorders. J of Ultrasound Medicine. 2023;42(3):687–99. doi:10.1002/jum.16081

Li Y, Tang J, Fei X, Gao Y. Diagnostic Performance of Contrast Enhanced Ultrasound in Patients with Prostate Cancer. Academic Radiology. 2013;20(2):156–64. doi:10.1016/j.acra.2012.09.018

Li Z, Gao Y, Gong H, Feng W, Ma Q, Li J, et al. Different Imaging Modalities for the Diagnosis of Axillary Lymph Node Metastases in Breast Cancer: A Systematic Review and Network Meta‐Analysis of Diagnostic Test Accuracy. Magnetic Resonance Imaging. 2023;57(5):1392–403. doi:10.1002/jmri.28399

Liberatore M, Calandri E, Ciccariello G, Fioravanti M, Megna V, Rampin L, et al. The Labeled-Leukocyte Scan in the Study of Abdominal Abscesses. Mol Imaging Biol. 2010;12(6):563–9. doi:10.1007/s11307-010-0314-0

Libson K, Sheridan C, Carr DR, Shahwan KT. Use of Imaging in Cutaneous Squamous Cell Carcinoma to Detect High-Risk Tumor Features, Nodal Metastasis, and Distant Metastasis: A Systematic Review. Dermatol Surg. 2024;50(8):705–9. doi:10.1097/DSS.0000000000004191

Lin LZ, Li F, Liu Y, Xing LX, Du LF. Contrast-Enhanced Ultrasound for differential diagnosis of pancreatic mass lesions: a meta-analysis. Med Ultrason. 2016;18(2):163. doi:10.11152/mu.2013.2066.182.ceu

Lin P, Chen M, Liu B, Wang S, Li X. Diagnostic performance of shear wave elastography in the identification of malignant thyroid nodules: a meta-analysis. Eur Radiol. 2014;24(11):2729–38. doi:10.1007/s00330-014-3320-9

Lin Y, Li H, Jin C, Wang H, Jiang B. The diagnostic accuracy of liver fibrosis in non-viral liver diseases using acoustic radiation force impulse elastography: A systematic review and meta-analysis. Alizad A, editor. PLoS ONE. 2020;15(1):e0227358. doi:10.1371/journal.pone.0227358

Lipszyc AC, Walker SCD, Beech AP, Wilding H, Akhlaghi H. Predicting Fluid Responsiveness Using Carotid Ultrasound in Mechanically Ventilated Patients: A Systematic Review and Meta-Analysis of Diagnostic Test Accuracy Studies. Anesth Analg. 2024;138(6):1174–86. doi:10.1213/ANE.0000000000006820

Liu B, Zheng Y, Huang G, Lin M, Shan Q, Lu Y, et al. Breast Lesions: Quantitative Diagnosis Using Ultrasound Shear Wave Elastography—A Systematic Review and Meta-Analysis. Ultrasound in Medicine & Biology. 2016;42(4):835–47. doi:10.1016/j.ultrasmedbio.2015.10.024

Liu J, Chen X, Li P, Dian H, Cheng X, Zhou Q, et al. Diagnostic value of HHUS versus ABVS for benign and malignant breast lesions: a meta-analysis. Chinese Journal of Evidence-Based Medicine. 2020;20(0):0. doi:10.7507/1672-2531.201909054

Liu PL, Tang SS. Contrast enhanced ultrasound and contrast enhanced CT for diagnosis of focal pancreas lesions: A meta-analysis. Chinese Journal of Medical Imaging Technology. 2011.

Liu Q, Cheng J, Li J, Gao X, Li H. The diagnostic accuracy of contrast-enhanced ultrasound for the differentiation of benign and malignant thyroid nodules: A PRISMA compliant meta-analysis. Medicine. 2018;97(49):e13325. doi:10.1097/MD.0000000000013325

Liu W, Zhang X, Liu K, Kang Z. Lung ultrasound for the diagnosis of pulmonary atelectasis in both adults and pediatrics: A protocol for systematic review and meta-analysis. Medicine. 2022;101(46):e28397. doi:10.1097/MD.0000000000031519

Liu X, Wang M, Wang Q, Zhang H. Diagnostic value of contrast-enhanced ultrasound for sentinel lymph node metastasis in breast cancer: an updated meta-analysis. Breast Cancer Res Treat. 2023;202(2):221–31. doi:10.1007/s10549-023-07063-2

Liu Y xia, Wang J zhen, Liu J ping, Pang C kun, Jia H ying. [Imaging diagnosis for fatty liver: a systematic review]. Zhongguo Yi Xue Ke Xue Yuan Xue Bao. 2004;26(5):580–4.

Liu Y, Kan Y, Zhang J, Li N, Wang Y. Characteristics of contrast‐enhanced ultrasound for diagnosis of solid clear cell renal cell carcinomas ≤4 cm: A meta‐analysis. Cancer Medicine. 2021;10(23):8288–99. doi:10.1002/cam4.4365

Liu Y, Xie Z, Huang Y, Lu X, Yin F. Uterine arteries pulsatility index by Doppler ultrasound in the prediction of preeclampsia: an updated systematic review and meta-analysis. Arch Gynecol Obstet. 2023;309(2):427–37. doi:10.1007/s00404-023-07044-2

Liu Z, Yang F, Zhang Y, Yu H, Zhu H, Yang R, et al. Conventional, Doppler and Contrast-Enhanced Ultrasonography in Differential Diagnosis of Ovarian Masses. Cell Physiol Biochem. 2016;39(6):2398–408. doi:10.1159/000452508

Losurdo G, De Bellis M, Rima R, Palmisano CM, Dell’Aquila P, Iannone A, et al. Small Intestinal Contrast Ultrasonography (SICUS) in Crohn’s Disease: Systematic Review and Meta-Analysis. J Clin Med. 2023;12(24):7714. doi:10.3390/jcm12247714

Luis Alcázar J, Ramón Pérez-Vidal J, Tameish S, Chacón E, Manzour N, Ángel Mínguez J. Ultrasound for assessing tumor spread in ovarian cancer. A systematic review of the literature and meta-analysis. European Journal of Obstetrics & Gynecology and Reproductive Biology. 2024;292:194–200. doi:10.1016/j.ejogrb.2023.11.017

Luo J, Cao Y, Nian W, Zeng X, Zhang H, Yue Y, et al. Benefit of Shear-wave Elastography in the differential diagnosis of breast lesion: a diagnostic meta-analysis. Med Ultrason. 2018;1(1):43. doi:10.11152/mu-1209

Luo K, Wang H, Huang F, Tang J. Optimal timing and cutoff range of lung ultrasound in predicting surfactant administration in neonates: A meta-analysis and systematic review. Tekleab AM, editor. PLoS ONE. 2023;18(7):e0287758. doi:10.1371/journal.pone.0287758

Ma C, Liu A, Liu A. Effectiveness of ultrasound in acute pulmonary edema diagnosis in ICU: a meta-analysis.

Ma X, Li Y, Jia H, Zhang J, Wang G, Liu X, et al. Contrast-Enhanced Ultrasound in the Diagnosis of Patients Suspected of Having Active Crohn’s Disease: Meta-analysis. Ultrasound in Medicine & Biology. 2015;41(3):659–68. doi:10.1016/j.ultrasmedbio.2014.09.013

Ma X, Liu R, Zhu C, Zhang J, Ling W. Diagnostic Value of Contrast-Enhanced Sonography for Differentiation of Breast Lesions: A Meta-analysis. J Ultrasound Med. 2016;35(10):2095–102. doi:10.7863/ultra.15.10005

Ma X, Zhang B, Ling W, Liu R, Jia H, Zhu F, et al. Contrast‐enhanced sonography for the identification of benign and malignant thyroid nodules: Systematic review and meta‐analysis. J of Clinical Ultrasound. 2016;44(4):199–209. doi:10.1002/jcu.22311

Ma X, Zhao Y, Zhang B, Ling W, Zhuo H, Jia H, et al. Contrast-enhanced ultrasound for differential diagnosis of malignant and benign ovarian tumors: systematic review and meta-analysis. Ultrasound Obstet Gynecol. 2015;46(3):277–83. doi:10.1002/uog.14800

Mahmood S, Anjum MN, Farooq F, Gilani SA, Fatima M, Andlib S, et al. Comparison of Mammography and Ultrasonography for Early Detection of Breast Cancer. PJMHS. 2021;15(7):1450–5. doi:10.53350/pjmhs211571450

Malik P, Pillai S, Agarwal K, Abdelwahed S, Bhandari R, Singh A, et al. Diagnostic Accuracy of Elastography and Liver Disease: A Meta-Analysis. Gastroenterology Res. 2022;15(5):232–9. doi:10.14740/gr1557

Man V, Luk WP, Fung LH, Kwong A. The role of pre-operative axillary ultrasound in assessment of axillary tumor burden in breast cancer patients: a systematic review and meta-analysis. Breast Cancer Res Treat. 2022;196(2):245–54. doi:10.1007/s10549-022-06699-w

Manatsathit W, Samant H, Panjawatanan P, Braseth A, Suh J, Esmadi M, et al. Performance of ultrasound for detection of transjugular intrahepatic portosystemic shunt dysfunction: a meta-analysis. Abdom Radiol. 2019;44(7):2392–402. doi:10.1007/s00261-019-01981-w

Martin E, Gill R, Debru E. A systematic review of transabdominal ultrasound in the diagnosis of gallbladder polyps. cjs. 2017;60(4):S109–81. doi:10.1503/cjs.010417

Martin E, Gill R, Debru E. Diagnostic accuracy of transabdominal ultrasonography for gallbladder polyps: systematic review. Can J Surg. 2018;61(3):200–7. doi:10.1503/cjs.011617

Masala I. Transcranial Doppler Ultrasound for the Diagnosis of Cerebral Vasospasm After Aneurysmal Subarachnoid Haemorrhage. 2022.

Maybury CM, Samarasekera E, Douiri A, Barker JN, Smith CH. Diagnostic accuracy of noninvasive markers of liver fibrosis in patients with psoriasis taking methotrexate: a systematic review and meta-analysis. Br J Dermatol. 2014;170(6):1237–47. doi:10.1111/bjd.12905

Mei M, Ye L, Quan J, Huang P. Contrast-enhanced ultrasound for the differential diagnosis between benign and metastatic superficial lymph nodes: a meta-analysis. CMAR. 2018;Volume 10:4987–97. doi:10.2147/CMAR.S174751

Mei YL, Yang J, Wu ZR, Yang Y, Xu YM. Transcranial Sonography of the Substantia Nigra for the Differential Diagnosis of Parkinson’s Disease and Other Movement Disorders: A Meta-Analysis. Colosimo C, editor. Parkinson’s Disease. 2021;2021:1–9. doi:10.1155/2021/8891874

Meinel TR, Eggimann A, Brignoli K, Wustmann K, Buffle E, Meinel FG, et al. Cardiovascular MRI Compared to Echocardiography to Identify Cardioaortic Sources of Ischemic Stroke: A Systematic Review and Meta-Analysis. Front Neurol. 2021;12:699838. doi:10.3389/fneur.2021.699838

Migda B, Migda M, Migda MS, Slapa RZ. Use of the Kwak Thyroid Image Reporting and Data System (K-TIRADS) in differential diagnosis of thyroid nodules: systematic review and meta-analysis. Eur Radiol. 2018;28(6):2380–8. doi:10.1007/s00330-017-5230-0

Mirza TA, Karthikesalingam A, Jackson D, Walsh SR, Holt PJ, Hayes PD, et al. Duplex Ultrasound and Contrast-Enhanced Ultrasound Versus Computed Tomography for the Detection of Endoleak after EVAR: Systematic Review and Bivariate Meta-Analysis. European Journal of Vascular and Endovascular Surgery. 2010;39(4):418–28. doi:10.1016/j.ejvs.2010.01.001

Miura Y, Tamai N, Kitamura A, Yoshida M, Takahashi T, Mugita Y, et al. Diagnostic accuracy of ultrasound examination in detecting aspiration and pharyngeal residue in patients with dysphagia: A systematic review and meta‐analysis. Japan Journal Nursing Sci. 2021;18(2):e12396. doi:10.1111/jjns.12396

Mojadidi MK, Gevorgyan R, Romero J, Goodman-Meza D, States U, Winoker J, et al. Comparison of transcranial doppler, transthoracic echo and transthoracic echo with harmonic imaging for the diagnosis of intracardiac right-to-left shunt: A meta-analysis of prospective studies. Catheterization and Cardiovascular Interventions. 2014.

Mojadidi MK, Winoker JS, Roberts SC, Msaouel P, Gevorgyan R, Zolty R. Two-dimensional echocardiography using second harmonic imaging for the diagnosis of intracardiac right-to-left shunt: A meta-analysis of prospective studies. International Journal of Cardiovascular Imaging. 2014;30(5):911–23. doi:10.1007/s10554-014-0426-8

Mojadidi MK, Winoker JS, Roberts SC, Msaouel P, Zaman MO, Gevorgyan R, et al. Accuracy of Conventional Transthoracic Echocardiography for the Diagnosis of Intracardiac Right-to-Left Shunt: A Meta-Analysis of Prospective Studies. Echocardiography. 2014;31(9):1036–48. doi:10.1111/echo.12583

Moore R, Mullner D, Nichols G, Scomacao I, Herrera F. Color Doppler Ultrasound versus Computed Tomography Angiography for Preoperative Anterolateral Thigh Flap Perforator Imaging: A Systematic Review and Meta-Analysis. J Reconstr Microsurg. 2022;38(7):563–70. doi:10.1055/s-0041-1740958

Mu J, Cao Y, Zhong X, Diao W, Jia Z. Prediction of Lymph Node Metastasis in Differentiated Thyroid Cancer Based on Radiomics Models: A Metaanalysis and Systematic Review. Eur J Nucl Med Mol Imaging. 2023;50(S1):1–898. doi:10.1007/s00259-023-06333-x

Myers ER, Bastian LA, Havrilesky LJ, Kulasingam SL, Terplan MS, Cline KE, et al. Management of adnexal mass. Evid Rep Technol Assess (Full Rep). 2006;(130):1–145.

Nafisi Moghadam R, Amelshahbaz AP, Namiranian N, Sobhan-Ardekani M, Emami-Meybodi M, Dehghan A, et al. Comparative Diagnostic Performance of Ultrasonography and 99mTc-Sestamibi Scintigraphy for Parathyroid Adenoma in Primary Hyperparathyroidism; Systematic Review and Meta- Analysis. Asian Pacific Journal of Cancer Prevention. 2017;18(12):3195–200. doi:10.22034/APJCP.2017.18.12.3195

Nayak S, Olkin I, Liu H, Grabe M, Gould MK, Allen IE, et al. Meta-Analysis: Accuracy of Quantitative Ultrasound for Identifying Patients with Osteoporosis. Ann Intern Med. 2006;144(11):832–41. doi:10.7326/0003-4819-144-11-200606060-00009

Nell S, Kist JW, Debray TPA, De Keizer B, Van Oostenbrugge TJ, Borel Rinkes IHM, et al. Qualitative elastography can replace thyroid nodule fine-needle aspiration in patients with soft thyroid nodules. A systematic review and meta-analysis. European Journal of Radiology. 2015;84(4):652–61. doi:10.1016/j.ejrad.2015.01.003

Newhouse I, Spacey A, Scragg B, Szczepura K. The diagnostic value and accuracy of ultrasound in diagnosing hydatidiform mole: A systematic review and meta-analysis of the literature. Radiography. 2022;28(4):897–905. doi:10.1016/j.radi.2022.06.005

Ng SJ, Lau HC, Kim V, Revheim ME, Werner T, Alavi A, et al. Advances in Characterization of Cardiac Tumors by PET/CT and PET/MRI. Journal of Nuclear Medicine. 2023;64(supplement 1):P1067–P1067.

Ni JR, Yan PJ, Liu SD, Hu Y, Yang KH, Song B, et al. Diagnostic accuracy of transthoracic echocardiography for pulmonary hypertension: a systematic review and meta-analysis. BMJ Open. 2019;9(12):e033084. doi:10.1136/bmjopen-2019-033084

Nie W, Zhu L, Yan P, Sun J. Thyroid nodule ultrasound accuracy in predicting thyroid malignancy based on TIRADS system. Adv Clin Exp Med. 2022;31(6):597–606. doi:10.17219/acem/146776

Niu J, Chen H, Peng J, Yuan H. A Systematic Review and Meta-analysis on Ultrasound Detection of Thyroid Cancer in China. Iranian Red Crescent Medical Journal (IRCMJ). 2023;25(10):e189157. doi:10.32592/ircmj.2023.25.10.2899

Niu Z, Xiao M, Ma L, Qin J, Li W, Zhang J, et al. The value of contrast-enhanced ultrasound enhancement patterns for the diagnosis of sentinel lymph node status in breast cancer: systematic review and meta-analysis. Quant Imaging Med Surg. 2022;12(2):936–48. doi:10.21037/qims-21-416

Nozaki S, Shiroshita A, Tanaka Y, Luo Y, Kataoka Y, Nakashima K. Utility of thoracic ultrasound for detecting malignant pleural effusion: a systematic review and meta-analysis. In: Ultrasound. European Respiratory Society; 2020. p. 2786. doi:10.1183/13993003.congress-2020.2786

Oh KE, Vasandani N, Anwar A. Radiomics to Differentiate Malignant and Benign Breast Lesions: A Systematic Review and Diagnostic Test Accuracy Meta-Analysis. Cureus. 2023. doi:10.7759/cureus.49015

Otify M, Laios A, Elshamy T, D’Angelo A, Amso NN. A systematic review and meta-analysis of the use of ultrasound to diagnose borderline ovarian tumours. European Journal of Obstetrics & Gynecology and Reproductive Biology. 2020;244:120–7. doi:10.1016/j.ejogrb.2019.11.016

Pakizer D, Kozel J, Taffé P, Elmers J, Feber J, Michel P, et al. Diagnostic accuracy of non-invasive imaging methods in detection of intraplaque hemorrhage in carotid atherosclerotic plaques: A systematic review and meta-analysis. Journal of the Neurological Sciences. 2023;455. doi:10.1016/j.jns.2023.121122

Pakizer D, Tafe P, Kozel J, Elmers J, Feber J, Michel P, et al. CHARACTERISTICS of ATHEROSCLEROTIC PLAQUES in CAROTID ARTERIES on NONINVASIVE IMAGING MODALITIES COMPARED to HISTOLOGY: A SYSTEMATIC REVIEW and DIAGNOSTIC TEST ACCURACY META-ANALYSIS. Neuroradiology. 2023;65:S121–2.

Pan KH, Jian L, Chen WJ, Nikzad AA, Kong FQ, Bin X, et al. Diagnostic Performance of Contrast-Enhanced Ultrasound in Renal Cancer: A Meta-Analysis. Front Oncol. 2020;10:586949. doi:10.3389/fonc.2020.586949

Pan L, Han Y, Sun X, Liu J, Gang H. FDG-PET and other imaging modalities for the evaluation of breast cancer recurrence and metastases: a meta-analysis. J Cancer Res Clin Oncol. 2010;136(7):1007–22. doi:10.1007/s00432-009-0746-6

Pang KH, Campi R, Arlandis S, Bo K, Chapple CR, Costantini E, et al. Diagnostic Tests for Female Bladder Outlet Obstruction: A Systematic Review from the European Association of Urology Non-neurogenic Female LUTS Guidelines Panel. Eur Urol Focus. 2022;8(4):1015–30. doi:10.1016/j.euf.2021.09.003

Park S, Hwang J, Kim S, Choi W. Differentiating value of shear wave elastography in breast ultrasound: A systematic review and meta-analysis. Value in Health. 2017;20(5):A236–7.

Patel A, Spychalski P, Aszkiełowicz A, Mikaszewski B, Kobiela J. Transcutaneous Laryngeal Ultrasound for Vocal Cord Paralysis Assessment in Patients Undergoing Thyroid and Parathyroid Surgery—A Systematic Review and Meta-Analysis. JCM. 2021;10(22):5393. doi:10.3390/jcm10225393

Phuah YZ, Ahmad M, Sim S, Tan YX, Wong J. Non-contrast versus contrast transthoracic echocardiography for the detection of left ventricular thrombus, using cardiac magnetic resonance imaging as a reference - a systematic review and meta-analysis. Heart. 2023;109(Suppl 3):A208–10. doi:10.1136/heartjnl-2023-BCS.177

Pinho JM, Coelho DA. Confirming identification of the epidural space: a systematic review of electric stimulation, pressure waveform analysis, and ultrasound and a meta-analysis of diagnostic accuracy in acute pain. J Clin Monit Comput. 2023;37(6):1593–605. doi:10.1007/s10877-023-01056-8

Pinto SPS, Huang DY, Dinesh AA, Sidhu PS, Ahmed K. A Systematic Review on the Use of Qualitative and Quantitative Contrast-enhanced Ultrasound in Diagnosing Testicular Abnormalities. Urology. 2021;154:16–23. doi:10.1016/j.urology.2021.02.012

Pruijt M, De Voogd F, Montazeri N, Jamaludin F, D’Haens G, Gecse K. Intestinal ultrasound is accurate for detecting intra-abdominal complications in Crohn’s disease: a meta-analysis. Journal of Crohn’s and Colitis. 2023;17(Supplement_1):i342–3. doi:10.1093/ecco-jcc/jjac190.0317

Pruijt M, De Voogd F, Montazeri NS, Jamaludin F, D’Haens G, Gecse KB. A META-ANALYSIS: INTESTINAL ULTRASOUND IS AN ACCURATE TOOL FOR THE DIAGNOSIS OF INTRA-ABDOMINAL COMPLICATIONS RELATED TO CROHN’S DISEASE. Gastroenterology. 2023;164(6):S-672. doi:10.1016/S0016-5085(23)02564-7

Pruijt MJ, de Voogd FAE, Montazeri NSM, van Etten-Jamaludin FS, D’Haens GR, Gecse KB. Diagnostic Accuracy of Intestinal Ultrasound in the Detection of Intra-Abdominal Complications in Crohn’s Disease: A Systematic Review and Meta-Analysis. J Crohns Colitis. 2024;18(6):958–72. doi:10.1093/ecco-jcc/jjad215

Qin Z, Zhou Y, Ding J, Wen J, Chen Y, Zhou H, et al. Risk stratification for hepatocellular carcinoma of contrast-enhanced ultrasound Liver Imaging Reporting and Data System (LI-RADS) and the diagnostic performance of LR-5 and LR-M: a systematic review and meta-analysis. Clinical Radiology. 2022;77(4):e280–6. doi:10.1016/j.crad.2022.01.041

Qiu Y, Xing Z, Liu J, Peng Y, Zhu J, Su A. Diagnostic reliability of elastography in thyroid nodules reported as indeterminate at prior fine-needle aspiration cytology (FNAC): a systematic review and Bayesian meta-analysis. Eur Radiol. 2020;30(12):6624–34. doi:10.1007/s00330-020-07023-0

Raffone A, Raimondo D, Neola D, Travaglino A, Raspollini A, Giorgi M, et al. Diagnostic Accuracy of Ultrasound in the Diagnosis of Uterine Leiomyomas and Sarcomas. J Minim Invasive Gynecol. 2024;31(1):28-36.e1. doi:10.1016/j.jmig.2023.09.013

Rafique J, Gilani SA, Murrium SKTS, Ahmad T, Gilani A, Jameel W. Evaluation of Different Diagnostic Modalities in Breast Cancer - A Systematic Review. PJMHS. 2022;16(1):17–8. doi:10.53350/pjmhs2216116

Rahmani G, McCarthy P, Bergin D. The diagnostic accuracy of ultrasonography for soft tissue lipomas: a systematic review. Acta Radiologica Open. 2017;6(6):205846011771670. doi:10.1177/2058460117716704

Ramsubeik K, Motilal S, Sanchez-Ramos L, Ramrattan LA, Kaeley GS, Singh JA. Diagnostic accuracy of salivary gland ultrasound in Sjögren’s syndrome: A systematic review and meta-analysis. Ther Adv Musculoskelet Dis. 2020;12:1759720X20973560. doi:10.1177/1759720X20973560

Ran L, Zhao W, Zhao Y, Bu H. Value of contrast-enhanced ultrasound in differential diagnosis of solid lesions of pancreas (SLP): A systematic review and a meta-analysis. Medicine. 2017;96(28):e7463. doi:10.1097/MD.0000000000007463

Rana P, Gupta P, Kalage D, Soundararajan R, Kumar-M P, Dutta U. Grayscale ultrasonography findings for characterization of gallbladder wall thickening in non-acute setting: a systematic review and meta-analysis. Expert Review of Gastroenterology & Hepatology. 2022;16(1):59–71. doi:10.1080/17474124.2021.2011210

Ray A, Bajaj N, Auer D, Schwarz S. Measuring substantia nigra echogenicity with transcranial sonography as an adjuvant to diagnosis of parkinson’s disease: A systematic review and meta-analysis. Neuroradiology. 2012;54(S1):1–194. doi:10.1007/s00234-012-1062-4

Razavi SA, Hadduck TA, Sadigh G, Dwamena BA. Comparative effectiveness of elastographic and B-mode ultrasound criteria for diagnostic discrimination of thyroid nodules: a meta-analysis. AJR Am J Roentgenol. 2013;200(6):1317–26. doi:10.2214/AJR.12.9215

Remonti LR, Kramer CK, Leitão CB, Pinto LCF, Gross JL. Thyroid Ultrasound Features and Risk of Carcinoma: A Systematic Review and Meta-Analysis of Observational Studies. Thyroid®. 2015;25(5):538–50. doi:10.1089/thy.2014.0353

Ren J, Lu Q, Fei X, Dong Y, D Onofrio M, Sidhu PS, et al. Assessment of arterial-phase hyperenhancement and late-phase washout of hepatocellular carcinoma-a meta-analysis of contrast-enhanced ultrasound (CEUS) with SonoVue® and Sonazoid®. Eur Radiol. 2024;34(6):3795–812. doi:10.1007/s00330-023-10371-2

Ren P, Li K, Lu X, Xie M. Diagnostic Value of Transthoracic Echocardiography for Patent Foramen Ovale: A Meta-analysis. Ultrasound in Medicine & Biology. 2013;39(10):1743–50. doi:10.1016/j.ultrasmedbio.2013.03.016

Ren PP, Xie MX. Diagnostic accuracy of transthoracic echocardiography for patent foramen ovale: A systematic review and meta-analysis. International Journal of Cardiology. 2013;164(2):s14.

Rispo A, Imperatore N, Testa A, Nardone OM, Luglio G, Caporaso N, et al. Diagnostic Accuracy of Ultrasonography in the Detection of Postsurgical Recurrence in Crohn’s Disease: A Systematic Review with Meta-analysis. Inflamm Bowel Dis. 2018;24(5):977–88. doi:10.1093/ibd/izy012

Roifman I, Connelly KA, Wright GA, Wijeysundera HC. Echocardiography vs. Cardiac Magnetic Resonance Imaging for the Diagnosis of Left Ventricular Thrombus: A Systematic Review. Can J Cardiol. 2015;31(6):785–91. doi:10.1016/j.cjca.2015.01.011

Sadigh G, Carlos RC, Neal CH, Dwamena BA. Accuracy of quantitative ultrasound elastography for differentiation of malignant and benign breast abnormalities: a meta-analysis. Breast Cancer Res Treat. 2012;134(3):923–31. doi:10.1007/s10549-012-2020-x

Sadigh G, Carlos RC, Neal CH, Dwamena BA. Ultrasonographic differentiation of malignant from benign breast lesions: a meta-analytic comparison of elasticity and BIRADS scoring. Breast Cancer Res Treat. 2012;133(1):23–35. doi:10.1007/s10549-011-1857-8

Sadigh G, Carlos RC, Neal CH, Wojcinski S, Dwamena BA. Impact of breast mass size on accuracy of ultrasound elastography vs. conventional B-mode ultrasound: a meta-analysis of individual participants. Eur Radiol. 2013;23(4):1006–14. doi:10.1007/s00330-012-2682-0

Sagami S, Kobayashi T, Miyatani Y, Okabayashi S, Yamazaki H, Takada T, et al. Accuracy of Ultrasound for Evaluation of Colorectal Segments in Patients With Inflammatory Bowel Diseases: A Systematic Review and Meta-analysis. Clinical Gastroenterology and Hepatology. 2021;19(5):908-921.e6. doi:10.1016/j.cgh.2020.07.067

Sakellariou G, Schiepatti A, Scalvini D, Lusetti F, Fazzino E, Biagi F, et al. Musculoskeletal Ultrasound to Identify Subclinical Joint and Periarticular Involvement in Patients With Inflammatory Bowel Disease: A Systematic Literature Review. Front Med. 2022;9:919521. doi:10.3389/fmed.2022.919521

Samiei S, De Mooij K, Lobbes M, Keymeulen K, Van Nijnatten T, Smidt M. Diagnostic performance of noninvasive imaging for assessment of axillary pathologic complete response after neoadjuvant systemic therapy in clinically node-positive breast cancer: a meta-analysis. European Journal of Surgical Oncology. 2021;47(2):e16. doi:10.1016/j.ejso.2020.11.187

Sanei Sistani S, Parooie F. Breast Ultrasound Versus MRI in Prediction of Pathologic Complete Response to Neoadjuvant Chemotherapy for Breast Cancer: A Systematic Review and Meta-Analysis. Journal of Diagnostic Medical Sonography. 2021;37(1):47–57. doi:10.1177/8756479320964102

Saranya H, Afifah M, Hayati JN. DIAGNOSTIC ACCURACY OF ULTRASOUND AS AN IMAGING TOOL IN PREOPERATIVE ASSESSMENT FOR THYROIDECTOMY: A SYSTEMATIC LITERATURE REVIEW. Journal of Medical Imaging and Radiation Sciences. 2023;54(3):S33. doi:10.1016/j.jmir.2023.06.119

Schmid E, Thomschewski A, Taylor A, Zimmermann G, Kirschner M, Kobulashvili T, et al. Diagnostic accuracy of functional magnetic resonance imaging, Wada test, magnetoencephalography, and functional transcranial Doppler sonography for memory and language outcome after epilepsy surgery: A systematic review. Epilepsia. 2018;59(12):2305–17. doi:10.1111/epi.14588

Selvaraj E, Zamarfand H, Mozes F, Jayaswal A, Levick C, Palaniyappan N, et al. Diagnostic accuracy of acoustic radiation force impulse elastography for the staging of hepatic fibrosis in non-alcoholic fatty liver disease: a systematic review and meta-analysis. Journal of Hepatology. 2020;73:S410. doi:10.1016/S0168-8278(20)31305-2

Senthilkumar S, Nagarayan V, Jiwa N. Diagnostic accuracy of nipple discharge fluid cytology: A metaanalysis and systematic review of the literature. BMC Proc. 2021;15(S4):9, s12919-021-00213–8. doi:10.1186/s12919-021-00213-8

Serafin Z, Białecki M, Białecka A, Sconfienza LM, Kłopocka M. Contrast-enhanced Ultrasound for Detection of Crohn’s Disease Activity: Systematic Review and Meta-analysis. ECCOJC. 2016;10(3):354–62. doi:10.1093/ecco-jcc/jjv196

Serodio M, Soares MF, Muxagata T, Almeida V, Fonseca AC. Sensitivity and agreement rate of Doppler Ultrasound compared to CT angiography for Carotid Web diagnosis: systematic review. European Stroke Journal. 2023;8(2):296–296.

Serra-Arbeloa P, Rabines-Juarez AO, Alvarez-Ruiz S, Guillen-Grima F. The role of preoperative lymph node ultrasound in patients with primary cutaneous melanoma: A meta-analysis. Eur J Nucl Med Mol Imaging. 2015;42(S1):1–924. doi:10.1007/s00259-015-3198-z

Shafieesabet A, Fereshtehnejad SM, Shafieesabet A, Delbari A, Baradaran HR, Postuma RB, et al. Hyperechogenicity of substantia nigra for differential diagnosis of Parkinson’s disease: A meta-analysis. Parkinsonism & Related Disorders. 2017;42:1–11. doi:10.1016/j.parkreldis.2017.06.006

Shah S, Malik P, Patel U, Wang Y, Gronseth GS. Diagnostic Yield of TEE in Patients with Cryptogenic Stroke and TIA with Normal TTE: A Systematic Review and Meta-Analysis. Neurol Int. 2021;13(4):659–70. doi:10.3390/neurolint13040063

Shi JT, Zhang Y, She Y, Goyal H, Wu ZQ, Xu HG. Diagnostic Utility of Non-invasive Tests for Inflammatory Bowel Disease: An Umbrella Review. Front Med. 2022;9:920732. doi:10.3389/fmed.2022.920732

Shi KQ, Tang JZ, Zhu XL, Ying L, Li DW, Gao J, et al. Controlled attenuation parameter for the detection of steatosis severity in chronic liver disease: a meta-analysis of diagnostic accuracy. J Gastroenterol Hepatol. 2014;29(6):1149–58. doi:10.1111/jgh.12519

Shi M, Nong D, Xin M, Lin L. Accuracy of Ultrasound Diagnosis of Benign and Malignant Thyroid Nodules: A Systematic Review and Meta‐Analysis. Yang X, editor. International Journal of Clinical Practice. 2022;2022(1):5056082. doi:10.1155/2022/5056082

Shi M, Zhang X, Shi L, Wang J, Zhou X. Diagnostic value of automated breast volume scanning versus hand - held ultrasound for benign and malignant lesions in breast: a Meta-analysis. Cancer Research and Clinic. 2022;34(10):774–80.

Shin J, Lee S, Bae H, Chung YE, Choi J, Huh Y, et al. Contrast‐enhanced ultrasound liver imaging reporting and data system for diagnosing hepatocellular carcinoma: A meta‐analysis. Liver International. 2020;40(10):2345–52. doi:10.1111/liv.14617

Shiroshita A, Nakashima K, Takeshita M, Kataoka Y. Preoperative Lung Ultrasound to Detect Pleural Adhesions: A Systematic Review and Meta-Analysis. Cureus. 2021. doi:10.7759/cureus.14866

Shiroshita A, Nozaki S, Tanaka Y, Luo Y, Kataoka Y. Thoracic ultrasound for malignant pleural effusion: a systematic review and meta-analysis. ERJ Open Res. 2020;6(4):00464–2020. doi:10.1183/23120541.00464-2020

Shivgulam ME, Liu H, Kivell MJ, MacLeod JR, O’Brien MW. Effectiveness of contrast-enhanced duplex ultrasound for detecting renal artery stenosis: A systematic review. J Clin Ultrasound. 2024;52(6):763–72. doi:10.1002/jcu.23684

Shook SJ, Ginsberg M, Narayanaswami P, Beekman R, Dubin AH, Katirji B, et al. Evidence‐based guideline: Neuromuscular ultrasound for the diagnosis of ulnar neuropathy at the elbow. Muscle and Nerve. 2022;65(2):147–53. doi:10.1002/mus.27460

Simpson E, Stevenson M, Scope A, Poku E, Minton J, Evans P. Echocardiography in newly diagnosed atrial fibrillation patients: a systematic review and economic evaluation. Health Technol Assess. 2013;17(36):1–263, v–vi. doi:10.3310/hta17360

Singal A, Haaland B, Parikh N, Ozbay B, Kirshner C, Chakankar S, et al. Comparison of a novel multi-target blood test with ultrasound and alpha-fetoprotein for hepatocellular carcinoma surveillance: Systematic review and network meta-analysis. Hepatology. 2021;74(6):1383A-1420A. doi:10.1002/hep.32218

Singal AG, Haaland B, Parikh ND, Ozbay AB, Kirshner C, Chakankar S, et al. Comparison of a multitarget blood test to ultrasound and alpha-fetoprotein for hepatocellular carcinoma surveillance: Results of a network meta-analysis. Hepatol Commun. 2022;6(10):2925–36. doi:10.1002/hep4.2045

Singh M, Tuteja A, Wong DT, Goel A, Trivedi A, Tomlinson G, et al. Point-of-Care Ultrasound for Obstructive Sleep Apnea Screening: Are We There Yet? A Systematic Review and Meta-analysis. Anesthesia & Analgesia. 2019;129(6):1673–91. doi:10.1213/ANE.0000000000004350

Singla D, Gupta B, Varshney P, Mangla M, Walikar BN, Jamir T. Role of carotid corrected flow time and peak velocity variation in predicting fluid responsiveness: a systematic review and meta-analysis. Korean J Anesthesiol. 2023;76(3):183–93. doi:10.4097/kja.22385

Son JH, Choi SH, Kim SY, Lee SJ, Park SH, Kim KW, et al. Accuracy of contrast-enhanced ultrasound liver imaging reporting and data system: a systematic review and meta-analysis. Hepatol Int. 2020;14(6):1104–13. doi:10.1007/s12072-020-10102-5

Song GG, Bae SC, Lee YH. Diagnostic accuracy of lung ultrasound for interstitial lung disease in patients with connective tissue diseases: a meta-analysis. Clin Exp Rheumatol. 2016;34(1):11–6.

Song J, Huang J, Huang H, Liu S, Luo Y. Performance of spleen stiffness measurement in prediction of clinical significant portal hypertension: A meta-analysis. Clinics and Research in Hepatology and Gastroenterology. 2018;42(3):216–26. doi:10.1016/j.clinre.2017.11.002

Songtanin B, Brittan K, Sanchez S, Le M, Schmidt C, Ingviya T, et al. Diagnostic performance of contrast-enhanced ultrasound in diagnosing hepatic artery occlusion after liver transplantation: A systematic review and meta-analysis. Clin Transplant. 2023;37(11):e15070. doi:10.1111/ctr.15070

Sood R, Rositch AF, Ambinder E, Pool KL, Shakoor D, Pollack E, et al. Ultrasound for Breast Cancer Detection in Low-Resource Settings: Systematic Review and Meta-Analysis. JGO. 2018;4(Supplement 3):43s–43s. doi:10.1200/JGO.18.10510

Sood R, Rositch AF, Shakoor D, Ambinder E, Pool KL, Pollack E, et al. Ultrasound for Breast Cancer Detection Globally: A Systematic Review and Meta-Analysis. JGO. 2019;(5):1–17. doi:10.1200/JGO.19.00127

Spiesecke P, Neumann K, Wakonig K, Lerchbaumer MH. Contrast-enhanced ultrasound (CEUS) in characterization of inconclusive cervical lymph nodes: a meta-analysis and systematic review. Sci Rep. 2022;12(1):7804. doi:10.1038/s41598-022-11542-9

Staibano P, Forner D, Noel CW, Zhang H, Gupta M, Monteiro E, et al. Ultrasonography and Fine‐Needle Aspiration in Indeterminate Thyroid Nodules: A Systematic Review of Diagnostic Test Accuracy. The Laryngoscope. 2022;132(1):242–51. doi:10.1002/lary.29778

Staub Jonck L, Biscaro R, Maurici R. Accuracy and Applications of Lung Ultrasound to Diagnose Ventilator-Associated Pneumonia: A Systematic Review. Journal of intensive care medicine. 2018;33:447–55.

Su E, Hamilton C, Tawfik DS, Mariano K, Ginsburg S, Conlon T, et al. Laryngeal Ultrasound Detects Vocal Fold Immobility in Adults: A Systematic Review. J of Ultrasound Medicine. 2022;41(8):1873–88. doi:10.1002/jum.15884

Suh CH, Baek JH, Choi YJ, Lee JH. Performance of CT in the Preoperative Diagnosis of Cervical Lymph Node Metastasis in Patients with Papillary Thyroid Cancer: A Systematic Review and Meta-Analysis. AJNR Am J Neuroradiol. 2017;38(1):154–61. doi:10.3174/ajnr.A4967

Sumak Hazir M, Unal D, Ambarcioglu P. Distance from skin to epiglottis measurement with ultrasonography as a predictor of difficult laryngoscopy: A diagnostic test accuracy meta-analysis. Trends in Anaesthesia and Critical Care. 2023;52:101291. doi:10.1016/j.tacc.2023.101291

Summers JA, Radhakrishnan M, Morris E, Chalkidou A, Rua T, Patel A, et al. Virtual TouchTM Quantification to Diagnose and Monitor Liver Fibrosis in Hepatitis B and Hepatitis C: A NICE Medical Technology Guidance. Appl Health Econ Health Policy. 2017;15(2):139–54. doi:10.1007/s40258-016-0277-7

Sun B, Lang L, Zhu X, Jiang F, Hong Y, He L. Accuracy of contrast-enhanced ultrasound in the identification of thyroid nodules: a meta-analysis. Int J Clin Exp Med. 2015;8(8):12882–9.

Sun C, Lin S, Zhao L, Xin S. A meta-analysis of ultrasound imaging in diagnosis of endoleak among patients after endovascular abdominal aortic aneurysm repair. Ultrasound imaging.

Sun J, Cai J, Wang X. Real‐time Ultrasound Elastography for Differentiation of Benign and Malignant Thyroid Nodules: A Meta‐analysis. J of Ultrasound Medicine. 2014;33(3):495–502. doi:10.7863/ultra.33.3.495

Tadesse GF, Tegaw EM, Abdisa EK. Diagnostic performance of mammography and ultrasound in breast cancer: a systematic review and meta-analysis. J Ultrasound. 2023;26(2):355–67. doi:10.1007/s40477-022-00755-3

Takase-Minegishi K, Horita N, Kobayashi K, Yoshimi R, Kirino Y, Ohno S, et al. Diagnostic test accuracy of ultrasound for synovitis in rheumatoid arthritis: systematic review and meta-analysis. Rheumatology (Oxford). 2018;57(1):49–58. doi:10.1093/rheumatology/kex036

Taleb M, Khuder S, Tinkel J, Khouri SJ. The Diagnostic Accuracy of D oppler Echocardiography in Assessment of Pulmonary Artery Systolic Pressure: A Meta‐Analysis. Echocardiography. 2013;30(3):258–65. doi:10.1111/echo.12061

Talwalkar JA, Kurtz DM, Schoenleber SJ, West CP, Montori VM. Ultrasound-Based Transient Elastography for the Detection of Hepatic Fibrosis: Systematic Review and Meta-analysis. Clinical Gastroenterology and Hepatology. 2007;5(10):1214–20. doi:10.1016/j.cgh.2007.07.020

Tang GX, Xiao XY, Xu XL, Yang HY, Cai YC, Liu XD, et al. Diagnostic value of ultrasound elastography for differentiation of benign and malignant axillary lymph nodes: a meta-analysis. Clin Radiol. 2020;75(6):481.e9-481.e16. doi:10.1016/j.crad.2020.03.021

Tang Y, Li X, Jiang Q, Zhai L. Diagnostic accuracy of multiparametric ultrasound in the diagnosis of prostate cancer: systematic review and meta-analysis. Insights Imaging. 2023;14(1):203. doi:10.1186/s13244-023-01543-1

Tang YN, Yaghoobi M, Calo NC, Mbuagbaw L, Albashir SM. INTESTINAL ULTRASOUND USE IN DETECTING POST-OPERATIVE RECURRENCE OF CROHN’S DISEASE: A DIAGNOSTIC TEST ACCURACY REVIEW AND META-ANALYSIS. Gastroenterology. 2021;160(6):S-514-S-515. doi:10.1016/S0016-5085(21)01935-1

Tao A, Chen G, Deng Y, Xu R. Accuracy of Transcranial Sonography of the Substantia Nigra for Detection of Parkinson’s Disease: A Systematic Review and Meta-analysis. Ultrasound in Medicine & Biology. 2019;45(3):628–41. doi:10.1016/j.ultrasmedbio.2018.11.010

Tat J, Tat J, Theodoropoulos J. Clinical applications of ultrasonography in the shoulder for the Orthopedic Surgeon: A systematic review. Orthop Traumatol Surg Res. 2020;106(6):1141–51. doi:10.1016/j.otsr.2020.06.005

Taylor JI, Souter LH, Barocas DA, Boorjian SA, Raman JD, Lotan Y. Diagnostic Imaging in the Evaluation of Asymptomatic Microhematuria: Systematic Review and Meta-analysis. J Urol. 2023;209(6):1099–106. doi:10.1097/JU.0000000000003395

Teng A, Liu F, Zhou D, He T, Chevalier Y, Klar RM. Effectiveness of 3-dimensional shoulder ultrasound in the diagnosis of rotator cuff tears: A meta-analysis. Medicine. 2018;97(37):e12405. doi:10.1097/MD.0000000000012405

Tian L, Wang L, Qin Y, Cai J. Systematic Review and Meta‐analysis of the Malignant Ultrasound Features of Triple‐Negative Breast Cancer. J of Ultrasound Medicine. 2020;39(10):2013–25. doi:10.1002/jum.15309

Timmermans A, Opmeer BC, Khan KS, Bachmann LM, Epstein E, Clark TJ, et al. Endometrial Thickness Measurement for Detecting Endometrial Cancer in Women With Postmenopausal Bleeding: A Systematic Review and Meta-Analysis. Obstetrics & Gynecology. 2010;116(1):160–7. doi:10.1097/AOG.0b013e3181e3e7e8

Toft J, Hadden WJ, Laurence JM, Lam V, Yuen L, Janssen A, et al. Imaging modalities in the diagnosis of pancreatic adenocarcinoma: A systematic review and meta-analysis of sensitivity, specificity and diagnostic accuracy. European Journal of Radiology. 2017;92:17–23. doi:10.1016/j.ejrad.2017.04.009

Trimboli P, Castellana M, Virili C, Havre RF, Bini F, Marinozzi F, et al. Performance of contrast-enhanced ultrasound (CEUS) in assessing thyroid nodules: a systematic review and meta-analysis using histological standard of reference. Radiol med. 2020;125(4):406–15. doi:10.1007/s11547-019-01129-2

Tsai WW, Hung KC, Huang YT, Yu CH, Lin CH, Chen IW, et al. Diagnostic efficacy of sonographic measurement of laryngeal air column width difference for predicting the risk of post-extubation stridor: A meta-analysis of observational studies. Front Med (Lausanne). 2023;10:1109681. doi:10.3389/fmed.2023.1109681

Tsili AC, Alexiou G, Naka C, Argyropoulou MI. Imaging of colorectal cancer liver metastases using contrast-enhanced US, multidetector CT, MRI, and FDG PET/CT: a meta-analysis. Acta Radiol. 2021;62(3):302–12. doi:10.1177/0284185120925481

Tsujimoto Y, Kumasawa J, Shimizu S, Nakano Y, Kataoka Y, Tsujimoto H, et al. Doppler trans-thoracic echocardiography for detection of pulmonary hypertension in adults. Cochrane Database Syst Rev. 2022;5(5):CD012809. doi:10.1002/14651858.CD012809.pub2

Tu FF, Hahn D, Steege JF. Pelvic congestion syndrome-associated pelvic pain: a systematic review of diagnosis and management. Database of Abstracts of Reviews of Effects (DARE): Quality-assessed Reviews. 2010.

Tufano A, Antonelli L, Di Pierro GB, Flammia RS, Minelli R, Anceschi U, et al. Diagnostic Performance of Contrast-Enhanced Ultrasound in the Evaluation of Small Renal Masses: A Systematic Review and Meta-Analysis. Diagnostics. 2022;12(10):2310. doi:10.3390/diagnostics12102310

Tufano A, Rosati D, Moriconi M, Santarelli V, Canale V, Salciccia S, et al. Diagnostic Accuracy of Contrast-Enhanced Ultrasound (CEUS) in the Detection of Muscle-Invasive Bladder Cancer: A Systematic Review and Diagnostic Meta-Analysis. Current Oncology. 2024;31(2):818–27. doi:10.3390/curroncol31020060

Tzartzeva K, Obi J, Rich NE, Parikh ND, Marrero JA, Yopp A, et al. Surveillance Imaging and Alpha Fetoprotein for Early Detection of Hepatocellular Carcinoma in Patients With Cirrhosis: A Meta-analysis. Gastroenterology. 2018;154(6):1706-1718.e1. doi:10.1053/j.gastro.2018.01.064

Ullah W, Minalyan A, Saleem S, Nadeem N, Abdullah HM, Abdalla A, et al. Comparative accuracy of non-invasive imaging versus right heart catheterization for the diagnosis of pulmonary hypertension: A systematic review and meta-analysis. Int J Cardiol Heart Vasc. 2020;29:100568. doi:10.1016/j.ijcha.2020.100568

Umar Z, Haseeb-Ul-Rasool M, Abbas R, Ariyaratnam JE, Sandhu J, Iqbal H, et al. POINT-OF-CARE ULTRASOUND (POCUS) USE IN INTENSIVE CARE UNIT (ICU) SETTING FOR COVID-19 PATIENTS AND EVALUATION OF VENOUS THROMBOEMBOLISM. CHEST. 2023;164(4):A3986–7. doi:10.1016/j.chest.2023.07.2598

Umar Z, Haseeb-Ul-Rasool M, Ashfaq S, Foster A, Sandhu J, Ariyaratnam JE, et al. UTILIZATION OF POINT-OF-CARE ULTRASOUND (POCUS) FOR DIAGNOSIS OF COVID-19 PNEUMONIA IN INTENSIVE CARE UNIT (ICU) SETTING. CHEST. 2023;164(4):A3988–9. doi:10.1016/j.chest.2023.07.2599

Valizadeh P, Jannatdoust P, Tahamtan M, Ghorani H, Dorcheh SS, Farnoud K, et al. Diagnostic performance of different imaging modalities for splenic malignancies: A comparative meta-analysis. Eur J Radiol Open. 2024;12:100566. doi:10.1016/j.ejro.2024.100566

Van Den Berg R, Ohrndorf S, Kortekaas MC, Van Der Helm-van Mil AHM. What is the value of musculoskeletal ultrasound in patients presenting with arthralgia to predict inflammatory arthritis development? A systematic literature review. Arthritis Res Ther. 2018;20(1):228. doi:10.1186/s13075-018-1715-8

Van Gruting IMA, Stankiewicz A, Thakar R, Santoro GA, IntHout J, Sultan AH. Imaging modalities for the detection of posterior pelvic floor disorders in women with obstructed defaecation syndrome. Cochrane Colorectal Group, editor. Cochrane Database of Systematic Reviews. 2021;2021(9). doi:10.1002/14651858.CD011482.pub2

Van Leeuwen FHP, Timmer MA, De Jong PA, Fischer K, Foppen W. Screening for subclinical synovial proliferation in haemophilia: A systematic review and meta‐analysis comparing physical examination and ultrasound. Haemophilia. 2023;29(2):445–55. doi:10.1111/hae.14737

Verburg N, Hoefnagels F, Pouwels P, Boellaard R, Barkhof F, Koekstra O, et al. The diagnostic accuracy of neuro-imaging to detect infiltrative glioma within the brain: A meta-analysis based on 1598 patients in 58 publications. Neuro-Oncology. 2013;15:iii194–iii194.

Verri D, Moro F, Fragomeni SM, Zaçe D, Bove S, Pozzati F, et al. The Role of Ultrasound in the Evaluation of Inguinal Lymph Nodes in Patients with Vulvar Cancer: A Systematic Review and Meta-Analysis. Cancers. 2022;14(13):3082. doi:10.3390/cancers14133082

Visser K, Hunink MG. Peripheral arterial disease: gadolinium-enhanced MR angiography versus color-guided duplex US--a meta-analysis. Radiology. 2000;216(1):67–77. doi:10.1148/radiology.216.1.r00jl0367

Vogel C, Ziegelmüller B, Ljungberg B, Bensalah K, Bex A, Canfield S, et al. Imaging in Suspected Renal-Cell Carcinoma: Systematic Review. Clinical Genitourinary Cancer. 2019;17(2):e345–55. doi:10.1016/j.clgc.2018.07.024

Wan Q, Cao P, Liu J. Meta-Analysis of Contrast Enhanced Ultrasound in Judging Benign and Malignant Thyroid Tumors. Khalaf OI, editor. Computational and Mathematical Methods in Medicine. 2021;2021:1–9. doi:10.1155/2021/2577113

Wang C, Yu C, Yang F, Yang G. Diagnostic accuracy of contrast-enhanced ultrasound for renal cell carcinoma: a meta-analysis. Tumour Biol. 2014;35(7):6343–50. doi:10.1007/s13277-014-1815-2

Wang F, Wang H. Diagnostic value of ultrasound elastography in triple negative breast cancer: A meta-analysis. Medicine (Baltimore). 2023;102(6):e32879. doi:10.1097/MD.0000000000032879

Wang H, Cao J, Fan H, Huang J, Zhang H, Ling W. Compared with CT/MRI LI-RADS, whether CEUS LI-RADS is worth popularizing in diagnosis of hepatocellular carcinoma?—a direct head-to-head meta-analysis. Quant Imaging Med Surg. 2023;13(8):4919–32. doi:10.21037/qims-22-1383

Wang J, Zhao R, Cheng J. Diagnostic accuracy of contrast-enhanced ultrasound to differentiate benign and malignant breast lesions: A systematic review and meta-analysis. European Journal of Radiology. 2022;149:110219. doi:10.1016/j.ejrad.2022.110219

Wang J, Zheng S, Ding L, Liang X, Wang Y, Greuter MJW, et al. Is Ultrasound an Accurate Alternative for Mammography in Breast Cancer Screening in an Asian Population? A Meta-Analysis. Diagnostics. 2020;10(11):985. doi:10.3390/diagnostics10110985

Wang L, Qi ZH. Automatic Breast Volume Scanner versus Handheld Ultrasound in Differentiation of Benign and Malignant Breast Lesions: A Systematic Review and Meta-analysis. Ultrasound Med Biol. 2019;45(8):1874–81. doi:10.1016/j.ultrasmedbio.2019.04.028

Wang P, Wu M, Li A, Ye X, Li C, Xu D. Diagnostic Value of Contrast-Enhanced Ultrasound for Differential Diagnosis of Malignant and Benign Soft Tissue Masses: A Meta-Analysis. Ultrasound Med Biol. 2020;46(12):3179–87. doi:10.1016/j.ultrasmedbio.2020.08.011

Wang PK, Lin TY, Su IM, Chang KV, Wu WT, Özçakar L. Preoperative lung ultrasound for confirming the double-lumen endotracheal tube position for one-lung ventilation: A systematic review and meta-analysis. Heliyon. 2023;9(4):e15458. doi:10.1016/j.heliyon.2023.e15458

Wang Y, Chen M, Chen P, Tong J, Zhang Y, Yang G. Diagnostic performance of ultrasound and computed tomography in parallel for the diagnosis of lymph node metastasis in patients with thyroid cancer: a systematic review and meta-analysis. Gland Surg. 2022;11(7):1212–23. doi:10.21037/gs-22-347

Wang Y, Na DR, Mu Y, Yao LH. Value of ultrasound contrast agents injected subcutaneously for diagnosing sentinel lymph nodes of breast cancer: A meta-analysis. Chinese Journal of Evidence-Based Medicine. 2015;15(5):537–41.

Wang YC, Huang CH, Tu YK. Pulmonary Hypertension and Pulmonary Artery Acceleration Time: A Systematic Review and Meta-Analysis. Journal of the American Society of Echocardiography. 2018 [cited 2026 May 11];(2).

Wells PS, Lensing AW, Davidson BL, Prins MH, Hirsh J. Accuracy of ultrasound for the diagnosis of deep venous thrombosis in asymptomatic patients after orthopedic surgery. A meta-analysis. Ann Intern Med. 1995;122(1):47–53. doi:10.7326/0003-4819-122-1-199501010-00008

Wen Y, Zhou H, Xia M, Liu Q, Quan H, Fang L. Differentiating progressive supranuclear palsy from other movement disorders using transcranial sonography: a systematic review and meta-analysis. Neurol Sci. 2024;45(2):455–65. doi:10.1007/s10072-023-07107-x

Wennmacker S, Lamberts M, Di Martino M, Drenth J, Gurusamy K, Van Laarhoven C. Systematic review and meta-analysis of transabdominal and endoscopic ultrasound for gallbladder polyps. United European Gastroenterology Journal. 2017;5(5_suppl):A161–836. doi:10.1177/2050640617725676

Wennmacker SZ, Lamberts MP, Di Martino M, Drenth JP, Gurusamy KS, Van Laarhoven CJ. Transabdominal ultrasound and endoscopic ultrasound for diagnosis of gallbladder polyps. Cochrane Hepato-Biliary Group, editor. Cochrane Database of Systematic Reviews. 2018;2018(8). doi:10.1002/14651858.CD012233.pub2

Westwood M, Joore M, Grutters J, Redekop K, Armstrong N, Lee K, et al. Contrast-enhanced ultrasound using SonoVue® (sulphur hexafluoride microbubbles) compared with contrast-enhanced computed tomography and contrast-enhanced magnetic resonance imaging for the characterisation of focal liver lesions and detection of liver metastases: a systematic review and cost-effectiveness analysis. Health Technol Assess. 2013;17(16):1–243. doi:10.3310/hta17160

Williams GJ, Macaskill P, Chan SF, Karplus TE, Yung W, Hodson EM, et al. Comparative accuracy of renal duplex sonographic parameters in the diagnosis of renal artery stenosis: paired and unpaired analysis. AJR Am J Roentgenol. 2007;188(3):798–811. doi:10.2214/AJR.06.0355

Winkler MH, Touw HR, Van De Ven PM, Twisk J, Tuinman PR. Diagnostic Accuracy of Chest Radiograph, and When Concomitantly Studied Lung Ultrasound, in Critically Ill Patients With Respiratory Symptoms: A Systematic Review and Meta-Analysis. Critical Care Medicine. 2018;46(7):e707–14. doi:10.1097/CCM.0000000000003129

Wu H, Tian H, Dong F, Liang W, Song D, Zeng J, et al. The role of grey-scale ultrasound in the diagnosis of adhesive capsulitis of the shoulder: a systematic review and meta-analysis. Med Ultrason. 2020;22(3):305. doi:10.11152/mu-2430

Wu J feng, Wu C, Wang Y lai, Wang Z ping. Diagnostic accuracy of contrast-enhanced ultrasound in the detection of small renal masses: A protocol of systematic review and meta-analysis. Medicine. 2020;99(29):e21262. doi:10.1097/MD.0000000000021262

Wu J, Zhou Z, Wang X, Jin Y, Wang Z, Jin G. Diagnostic performance of elastosonography in the differential diagnosis of benign and malignant salivary gland tumors: A meta-analysis. Front Oncol. 2022;12. doi:10.3389/fonc.2022.954751

Wu M, Li L, Wang J, Zhang Y, Guo Q, Li X, et al. Contrast-enhanced US for characterization of focal liver lesions: a comprehensive meta-analysis. Eur Radiol. 2018;28(5):2077–88. doi:10.1007/s00330-017-5152-x

Wu M, Ren A, Xu D, Peng X, Ye X, Li A. Diagnostic Performance of Elastography in Malignant Soft Tissue Tumors: A Systematic Review and Meta-analysis. Ultrasound in Medicine & Biology. 2021;47(4):855–68. doi:10.1016/j.ultrasmedbio.2020.12.017

Wu Y, Xia C, Chen J, Qin Q, Ye Z, Song B. Diagnostic performance of magnetic resonance imaging and contrast-enhanced ultrasound in differentiating intrahepatic cholangiocarcinoma from hepatocellular carcinoma: a meta-analysis. Abdom Radiol. 2023;49(1):34–48. doi:10.1007/s00261-023-04064-z

Wu Y, Zhou C, Shi B, Zeng Z, Wu X, Liu J. Systematic review and meta-analysis: diagnostic value of different ultrasound for benign and malignant thyroid nodules. Gland Surg. 2022;11(6):1067–77. doi:10.21037/gs-22-254

Wubulihasimu M, Maimaitusun M, Xu XL, Liu XD, Luo BM. The added value of contrast-enhanced ultrasound to conventional ultrasound in differentiating benign and malignant solid breast lesions: a systematic review and meta-analysis. Clinical Radiology. 2018;73(11):936–43. doi:10.1016/j.crad.2018.06.004

Xia B, Wang F, Friedrich-Rust M, Zhou F, Zhu J, Yang H, et al. Feasibility and Efficacy of Transient Elastography using the XL probe to diagnose liver fibrosis and cirrhosis: A meta-analysis. Medicine. 2018;97(39):e11816. doi:10.1097/MD.0000000000011816

Xiao G, Zhu S, Xiao X, Yan L, Yang J, Wu G. Comparison of laboratory tests, ultrasound, or magnetic resonance elastography to detect fibrosis in patients with nonalcoholic fatty liver disease: A meta-analysis. Hepatology. 2017;66(5):1486–501. doi:10.1002/hep.29302

Xiao Q, Zhu W, Tang H, Zhou L. Ultrasound radiomics in the prediction of microvascular invasion in hepatocellular carcinoma: A systematic review and meta-analysis. Heliyon. 2023;9(6):e16997. doi:10.1016/j.heliyon.2023.e16997

Xie HQ, Zhang WW, Sun DS, Chen XM, Yuan SF, Gong ZH, et al. A simplified lung ultrasound for the diagnosis of interstitial lung disease in connective tissue disease: a meta-analysis. Arthritis Res Ther. 2019;21(1):93. doi:10.1186/s13075-019-1888-9

Xie L, Guang Y, Ding H, Cai A, Huang Y. Diagnostic Value of Contrast-Enhanced Ultrasound, Computed Tomography and Magnetic Resonance Imaging for Focal Liver Lesions: A Meta-Analysis. Ultrasound in Medicine & Biology. 2011;37(6):854–61. doi:10.1016/j.ultrasmedbio.2011.03.006

Xiong R, Quan Q, Chen W, Peng G. Diagnostic value of perfluorobutane contrast-enhanced ultrasound in primary liver cancer: meta-analysis. Revista de Psiquiatria Clinica. 2022;49(3):87–91.

Xu P, Zhou AY, Zhang C, Xiao F. Contrast enhanced ultrasound for detecting invasive and noninvasive bladder cancer: Meta analysis. Chinese Journal of Interventional Imaging and Therapy. 2015;12(2):106–9.

Xu Z, Duan X, Yu X, Wang H, Dong X, Xiang Z. The accuracy of ultrasonography and magnetic resonance imaging for the diagnosis of Morton’s neuroma: a systematic review. Clinical Radiology. 2015;70(4):351–8. doi:10.1016/j.crad.2014.10.017

Xuan M, Zhou F, Ding Y, Zhu Q, Dong J, Zhou H, et al. Diagnostic accuracy of contrast-enhanced ultrasound in assessing the therapeutic response to radio frequency ablation for liver tumors: systematic review and meta-analysis. Surg Endosc. 2018;32(4):2067–75. doi:10.1007/s00464-017-5903-4

Xun L, Zhai L, Xu H. Comparison of conventional, doppler and contrast-enhanced ultrasonography in differential diagnosis of ovarian masses: a systematic review and meta-analysis. BMJ Open. 2021;11(12):e052830. doi:10.1136/bmjopen-2021-052830

Yamashita Y, Shimokawa T, Ashida R, Dietrich CF, D’Onofrio M, Hirooka Y, et al. Value of Low-Mechanical-Index Contrast-Enhanced Transabdominal Ultrasound for Diagnosis of Pancreatic Cancer: A Meta-analysis. Ultrasound in Medicine & Biology. 2021;47(12):3315–22. doi:10.1016/j.ultrasmedbio.2021.08.005

Yan X, Lü K, Chen T, Li J. Performance of Contrast-enhanced Ultrasound Liver Imaging Reporting and Data System LR-5 in the Diagnosis of Hepatocellular Carcinoma:A Meta-analysis. Acta Academiae Medicinae Sinicae. 2023;45(1):57–63.

Yang J, Huang J, Zhang Y, Zeng K, Liao M, Jiang Z, et al. Contrast-enhanced ultrasound and contrast-enhanced computed tomography for differentiating mass-forming pancreatitis from pancreatic ductal adenocarcinoma: a meta-analysis. Chin Med J (Engl). 2023;136(17):2028–36. doi:10.1097/CM9.0000000000002300

Yang J, Zhang F, Qiao Y. Diagnostic accuracy of ultrasound, CT and their combination in detecting cervical lymph node metastasis in patients with papillary thyroid cancer: a systematic review and meta-analysis. BMJ Open. 2022;12(7):e051568. doi:10.1136/bmjopen-2021-051568

Yang L, Wang S, Zhang L, Sheng C, Song F, Wang P, et al. Performance of ultrasonography screening for breast cancer: a systematic review and meta-analysis. BMC Cancer. 2020;20(1):499. doi:10.1186/s12885-020-06992-1

Yang Q, Zhang C, Zhang Z, Su B. Muscle ultrasound to diagnose sarcopenia in chronic kidney disease: a systematic review and bayesian bivariate meta-analysis. BMC Nephrol. 2024;25(1):12. doi:10.1186/s12882-023-03445-2

Yang X, Yang H, He Y. Diagnostic Values of CEUS, CECT and CEMRI for Renal Cystic Lesions on the Current Bosniak Criterion-A Meta-analysi. ADVANCED ULTRASOUND IN DIAGNOSIS AND THERAPY. 2022;6(4):165. doi:10.37015/AUDT.2022.210037

Yang Y, Liu C, Yan J, Liu K. Perfluorobutane contrast-enhanced ultrasonography for the diagnosis of HCC: a systematic review and meta-analysis. Abdom Radiol. 2021;46(10):4619–28. doi:10.1007/s00261-021-03141-5

Yao B, Liu J yu, Sun Y bo. RESPIRATORY VARIATION IN PERIPHERAL ARTERY VELOCITY TO PREDICT FLUID RESPONSIVENESS: A META-ANALYSIS. Critical Care Medicine. 2018;46(1):114–114. doi:10.1097/01.ccm.0000528283.10638.67

Ying L, Lin X, Xie ZL, Tang FY, Hu YP, Shi KQ. Clinical utility of acoustic radiation force impulse imaging for identification of malignant liver lesions: a meta-analysis. Eur Radiol. 2012;22(12):2798–805. doi:10.1007/s00330-012-2540-0

Yu D, Han Y, Chen T. Contrast‐Enhanced Ultrasound for Differentiation of Benign and Malignant Thyroid Lesions: Meta‐analysis. Otolaryngol--head neck surg. 2014;151(6):909–15. doi:10.1177/0194599814555838

Yung DE, Har-Noy O, Tham YS, Ben-Horin S, Eliakim R, Koulaouzidis A, et al. Capsule Endoscopy, Magnetic Resonance Enterography, and Small Bowel Ultrasound for Evaluation of Postoperative Recurrence in Crohn’s Disease: Systematic Review and Meta-Analysis. Inflamm Bowel Dis. 2017;24(1):93–100. doi:10.1093/ibd/izx027

Zaiem F, Almasri J, Tello M, Prokop LJ, Chaikof EL, Murad MH. A systematic review of surveillance after endovascular aortic repair. J Vasc Surg. 2018;67(1):320-331.e37. doi:10.1016/j.jvs.2017.04.058

Zaki HA, Shaban E, Salem W, Bilal F, Fayed M, Hendy M, et al. A Comparative Analysis Between Ultrasound and Electromyographic and Nerve Conduction Studies in Diagnosing Carpal Tunnel Syndrome (CTS): A Systematic Review and Meta-Analysis. Cureus. 2022;14(10):e30476. doi:10.7759/cureus.30476

Zhang B, Ma X, Zhan W, Zhu F, Li M, Huang J, et al. Real-Time Elastography in the Diagnosis of Patients Suspected of Having Prostate Cancer: A Meta-analysis. Ultrasound in Medicine & Biology. 2014;40(7):1400–7. doi:10.1016/j.ultrasmedbio.2014.02.020

Zhang D, Wang XN, Jiang L, Yu CX, Chen YN, Yu XJ, et al. Conventional ultrasonography and elastosonography in diagnosis of malignant thyroid nodules: A systematic review and meta-analysis. Front Endocrinol. 2023;13:1082881. doi:10.3389/fendo.2022.1082881

Zhang F, Li R, Li G, Jin L, Shi Q, Du L. Value of Contrast‐Enhanced Ultrasound in the Diagnosis of Renal Cancer and in Comparison With Contrast‐Enhanced Computed Tomography: A Meta‐analysis. J of Ultrasound Medicine. 2019;38(4):903–14. doi:10.1002/jum.14769

Zhang H, Hu J, Meng R, Liu F, Xu F, Huang M. A systematic review and meta-analysis comparing the diagnostic capability of automated breast ultrasound and contrast-enhanced ultrasound in breast cancer. Front Oncol. 2023;13:1305545. doi:10.3389/fonc.2023.1305545

Zhang J, Yu Y, Li Y, Wei L. Diagnostic value of contrast-enhanced ultrasound in hepatocellular carcinoma: a meta-analysis with evidence from 1998 to 2016. Oncotarget. 2017;8(43):75418–26. doi:10.18632/oncotarget.20049

Zhang J, Zhang X, Meng Y, Chen Y. Contrast-enhanced ultrasound for the differential diagnosis of thyroid nodules: An updated meta-analysis with comprehensive heterogeneity analysis. Cohen O, editor. PLoS ONE. 2020;15(4):e0231775. doi:10.1371/journal.pone.0231775

Zhang L, Zhang L, Wang H, Chen L, Sui G. Diagnostic performance of contrast-enhanced ultrasound and magnetic resonance imaging for detecting colorectal liver metastases: A systematic review and meta-analysis. Digestive and Liver Disease. 2019;51(9):1241–8. doi:10.1016/j.dld.2019.06.004

Zhang M, Wang R, Wu Y, Jing J, Chen S, Zhang G, et al. Micro-Ultrasound Imaging for Accuracy of Diagnosis in Clinically Significant Prostate Cancer: A Meta-Analysis. Front Oncol. 2019;9:1368. doi:10.3389/fonc.2019.01368

Zhang RF, Zhou L, Ma GF, Shao FC, Wu XH, Ying KJ. Diagnostic value of transthoracic Doppler echocardiography in pulmonary hypertension: a meta-analysis. Am J Hypertens. 2010;23(12):1261–4. doi:10.1038/ajh.2010.188

Zhang X, Lu D, Hu J, Banaei A, Abedi-Firouzjah R. The role of ultrasound and MRI in diagnosing of obstetrics cardiac disorders: A systematic review. Journal of Radiation Research and Applied Sciences. 2022;15(3):261–9. doi:10.1016/j.jrras.2022.06.007

Zhang X, Yao J, Zhang Y, Huang X, Wang W, Huang H. Updated Evaluation of the Diagnostic Performance of Double Contrast-Enhanced Ultrasonography in the Preoperative T Staging of Gastric Cancer: A Meta-Analysis and Systematic Review. Front Oncol. 2022;12:844390. doi:10.3389/fonc.2022.844390

Zhang X, Zhang X, Palida P, Zhang Y, Nadire T. Value of contrast-enhanced ultrasound in differential diagnosis of preoperative benign and malignant breast mass: A systematic review. Chinese Journal of Evidence-Based Medicine. 2014;14(1):79. doi:10.7507/1672-2531.20140014

Zhang XG, Palida P, Zhang Y, Nadire T. Diagnostic value of color Doppler ultrasonic for female breast cancer: A meta-analysis. Chinese Journal of Evidence-Based Medicine. 2013;13(9):1073–9.

Zhang XH, Xiao C. Diagnostic Value of Nineteen Different Imaging Methods for Patients with Breast Cancer: a Network Meta-Analysis. Cell Physiol Biochem. 2018;46(5):2041–55. doi:10.1159/000489443

Zhang Y, Sun X, Li J, Gao Q, Guo X, Liu J xin, et al. The diagnostic value of contrast-enhanced ultrasound and superb microvascular imaging in differentiating benign from malignant solid breast lesions: A systematic review and meta-analysis. CH. 2022;81(2):109–21. doi:10.3233/CH-211367

Zhang Y, Zhang J, Yang L, Huang S. A meta-analysis of the utility of transabdominal ultrasound for evaluation of gastric cancer. Medicine. 2021;100(32):e26928. doi:10.1097/MD.0000000000026928

Zhang Y, Zhu D, Feng R. Breast-specific gamma imaging versus ultrasound and mammography for breast cancer diagnosis: A meta-analysis. Int J Radiat Res. 2024;22(1):27–33. doi:10.61186/ijrr.22.1.27

Zhang Z, Ma C, Luo Y. Diagnostic value of liver contrast-enhanced ultrasound in early hepatocellular carcinoma: a systematic review and meta-analysis. J Gastrointest Oncol. 2023;14(2):626–35. doi:10.21037/jgo-23-211

Zhao D, Jing Y, Lin X, Zhang B. The value of color Doppler ultrasound in the diagnosis of thyroid nodules: a systematic review and meta-analysis. Gland Surg. 2021;10(12):3369–77. doi:10.21037/gs-21-752

Zhao F, Yang Q, Meng C, Jiang T. Comparison of the practicability of ultrasound and spiral computed tomography in the diagnosis of colon cancer: A protocol for systematic review and meta-analysis. Medicine. 2020;99(50):e23381. doi:10.1097/MD.0000000000023381

Zhao H, Li H. Meta-analysis of ultrasound for cervical lymph nodes in papillary thyroid cancer: Diagnosis of central and lateral compartment nodal metastases. European Journal of Radiology. 2019;112:14–21. doi:10.1016/j.ejrad.2019.01.006

Zhao YL, Wang Y. Value of ultrasound in diagnosis of breast cancer with axillary sentinel lymph nodes: A meta-analysis. Chinese Journal of Evidence-Based Medicine. 2014;14(9):1077–82.

Zhong L, Wang C. Diagnostic accuracy of ultrasound superb microvascular imaging for breast tumor: a meta-analysis. Med Ultrason. 2020;22(3):313–8. doi:10.11152/mu-2460

Zhou J, Tan J, Yang W, Huang T. False-negativity of preoperative ultrasound pose risk to active surveillance of ‘CN0’ papillary thyroid microcarcinoma. Thyroid. 2019;29(s1):P-1-A-166. doi:10.1089/thy.2019.29085.abstracts

Zhou L, Tang L, Yang T, Chen W. Comparison of contrast-enhanced ultrasound with MRI in the diagnosis of complex cystic renal masses: a meta-analysis. Acta Radiol. 2018;59(10):1254–63. doi:10.1177/0284185118755575

Zhou SC, Le J, Zhou J, Huang YX, Qian L, Chang C. The Role of Contrast-Enhanced Ultrasound in the Diagnosis and Pathologic Response Prediction in Breast Cancer: A Meta-analysis and Systematic Review. Clinical Breast Cancer. 2020;20(4):e490–509. doi:10.1016/j.clbc.2020.03.002

Zhou Y, Chen H, Qiang J, Wang D. Systematic review and meta-analysis of ultrasonic elastography in the diagnosis of benign and malignant thyroid nodules. Gland Surg. 2021;10(9):2734–44. doi:10.21037/gs-21-492

Zhou Y, Wu J. Which combination of different ultrasonography modalities is more appropriate to diagnose breast cancer?: A network meta-analysis (a PRISMA-compliant article). Medicine. 2022;101(31):e29955. doi:10.1097/MD.0000000000029955

Zhu L, Chen P, Sun X, Zhang S. Musculoskeletal ultrasound for ankylosing spondylitis: A systematic review and meta-analysis. Medicine. 2021;100(18):e25822. doi:10.1097/MD.0000000000025822
